# Synthesis of Diphenyl Sulfonamide Derivatives as Potential Inhibitors of V-ATPase

**DOI:** 10.2174/0115701794397334251126043618

**Published:** 2026-04-08

**Authors:** Mei Tian, Tao Guo, Jianjun Zhu, Yi Long, Ziyu Wang, Ziguang Shang, Jingxiu Zhang, Ruiyu Liu, Wenjun Wu, Zhaonong Hu, Jiwen Zhang

**Affiliations:** 1 College of Chemistry & Pharmacy, Northwest A&F University, Yangling, Shaanxi, 712100, People’s Republic of China;; 2 College of Life Science, Northwest A&F University, Yangling, Shaanxi, 712100, People’s Republic of China;; 3 Key Laboratory of Botanical Pesticide R&D in Shaanxi Province, Yangling, 712100, People’s Republic of China

**Keywords:** Structural optimization, sulfonamide derivatives, V-ATPase, insecticidal activity, electrophysiology, potential inhibitor

## Abstract

**Introduction:**

Sulfonamide compounds are widely used in pesticide chemistry. This study aims to synthesize novel diphenyl sulfonamide derivatives with improved pesticidal properties.

**Materials and Methods:**

Fifty-five diphenyl sulfonamide derivatives were designed and synthesized using Suzuki, Wittig, and Sonogashira couplings, as well as propargyl etherification. The compounds were characterized by spectroscopic techniques, and their insecticidal activities against *Mythimna separata* were evaluated. Their mechanisms of action were further explored through electrophysiological tests using a *Drosophila melanogaster* model and molecular docking analyses.

**Results:**

Compounds S6-3 and S6-4 demonstrated high insecticidal activity, with LC₅₀ values of 0.474 and 0.401 mg/mL, respectively, both superior to L-1. S6-4 affected *Drosophila melanogaster*neuromuscular junctions in a manner similar to bafilomycin A1, and molecular docking indicated binding to the H subunit of V-ATPase.

**Discussion:**

Structure-activity relationship analysis revealed the key structural features influencing insecticidal activity, providing guidance for further compound optimization.

**Conclusion:**

The synthesized derivatives targeting V-ATPase exhibited potent insecticidal activity and showed strong potential for the development of novel pesticides.

## 1 INTRODUCTION

Lepidopteran insects account for one-tenth of all globally described species, many of which are major agricultural and forest pests that cause significant economic damage worldwide [1-3]. Among these, *Mythimna separata* (Lepidoptera, Noctuidae) is a notorious polyphagous pest widely distributed across Asia, Australia, New Zealand, and several Pacific islands [4-6]. Due to its broad host range, this pest poses a severe threat to crops, particularly rice, corn, and wheat [4]. Currently, pesticide application remains the most common and effective method for controlling *M. separata*. However, excessive pesticide use can lead to increased resistance in pest populations and may also result in serious environmental issues [7, 8].

The extensive use of pesticides in agriculture and the subsequent development of resistance have rendered low-concentration pesticides ineffective, necessitating higher doses to achieve pest control. In some cases, such resistance has even resulted in complete pesticide failure. Moreover, pests frequently develop cross-resistance to pesticides targeting the same molecular mechanisms, further intensifying the challenge [9]. Therefore, identifying novel insecticidal targets and developing new pesticides is a critical strategy for addressing the growing issue of pesticide resistance.

Wu *et al.* (2016) reported that *Celastrus angulatus* Maxim. exhibits insecticidal activity, with its root bark containing a key component that shows unique insecticidal effects against *M. separata*. Furthermore, Lu *et al.* (2014) identified vacuolar-type H+-ATPase (V-ATPase) as the insecticidal target of dihydroagarofuran polyester, a pesticide with a novel mode of action [10-12]. V-ATPase generates a proton motive force in organelles and eukaryotic cell membranes, serving as the driving force for various secondary transport processes [13].

Yang *et al.* (2018) identified two compounds with insecticidal activity through homology modelling and molecular docking, using V-ATPase as the target. The structures of these compounds were then modified, and the results revealed that alkynyl propoxy and sulfonamide groups were the primary active moieties of these pesticides [14]. These compounds targeted the V-ATPase in the insect midgut, leading to midgut disruption, severe water loss, and eventual insect death.

Based on these previously identified pharmacophores (sulfonamide and alkynyl propoxy groups), the present study aimed to optimize insecticidal activity by exploring different linkages between benzene rings. Inspired by the structural features of pyrethroid pesticides, biphenyl, diphenylmethane, diphenylethene, and diphenylacetylene backbones were introduced to connect two benzene rings, with the goal of enhancing compound-target binding affinity and metabolic stability.

Several diphenyl sulfonamides with significant insecticidal activity, incorporating single benzene or naphthalene rings, have been reported to act on the H subunit of V-ATPase [15-17]. Consequently, chemical structures such as biphenyl, diphenylmethane, diphenylethene, and diphenylacetylene are frequently used in pesticide development (Fig. **1**) [18, 19]. In this study, compound design was informed by the development history of pyrethroid pesticides [20]. Specifically, two benzene rings were introduced and connected in various configurations to design and synthesize a series of diphenyl sulfonamides. Their insecticidal activity and structure-activity relationships were then evaluated to determine how different benzene-ring linkages influence the insecticidal efficacy of diphenyl sulfonamides. Additionally, the inhibitory mechanism of V-ATPase was examined through electrophysiological assays.

## 2 MATERIALS AND METHODS

### 2.1 Synthesis

All melting points were measured after column chromatography and are uncorrected. Column chromatography was performed using silica gel. All solvents and reagents were obtained from commercial sources and used without further purification. Triethylamine, toluene, *N, N*-dimethylfor-mamide, carbon tetrachloride, and tetrahydrofuran were purchased from Chengdu Chron Chemical Reagent Co., Ltd. Deuterated chloroform, deuterated dimethyl sulfoxide, bromopropyne, 4-bromobenzenesulfonyl chloride, 2-hydroxyphenylboronic acid, 3-hydroxyphenylboronic acid, 4-hydroxyphenylboronic acid, *p*-toluenesulfonyl chloride, 4-iodophenol, bromoacetonitrile, iodomethane, chlorosulfonic acid, dichlorosulfoxide, phenol, copper iodide, *N, N*-dimethylglycine, ultradry DMSO, boron tribromide, *N*-bromosuccinimide, tetrakis(triphenylphosphine)palladium, triphenylphosphine, potassium *tert*-butoxide, trimethylsilylacetylene, and dichlorobis(triphenylphosphine)palladium(II) were purchased from Anhui Zesheng Technology Co., Ltd. (Annegie Chemical).

### 2.2 Insecticidal Activity

The compounds and positive control drugs were dissolved in acetone. Fresh wheat leaves were cut into small squares (5 mm × 5 mm) for bioassays. Third-instar M. separata larvae and fresh wheat leaves were provided by the Key Laboratory of Botanical Pesticide R&D in Shaanxi Province, Northwest A&F University (Yangling, China).

### 2.3 Electrophysiological Recordings

For electrophysiological recordings, third-instar *Drosophila melanogaster* larvae were dissected and maintained in HL3 physiological solution. Glass microelectrodes and stimulating electrodes connected to a stimulator were used for the experiments. Data analysis was performed using SPSS 22.0 software.

## 3 EXPERIMENTAL SECTION: SYNTHESIS OF THE INTERMEDIATES AND TARGET COMPOUNDS

### 3.1 General Procedure for Preparing Intermediate 1b

To a round-bottom flask equipped with a magnetic stir bar were added 4-bromobenzenesulfonyl chloride (1a, 2 mmol), the corresponding substituted amine (3 mmol), Et₃N (5 mmol), and DCM (10 mL). After stirring at room temperature for 1 h, TLC indicated complete consumption of sulfonyl chloride 1a. The organic phase was washed with water and brine, dried over sodium sulfate, filtered, and concentrated under vacuum to afford intermediate 1b in 95-98% yield.

### 3.2 General Procedure for Preparing Intermediates 3a-3c

To a round-bottom flask equipped with a magnetic stir bar were added intermediate 1b (2 mmol), hydroxyphenylboronic acid (4 mmol), toluene (4 mL), ethanol (4 mL), sodium carbonate solution (4 mL, 2 M), and 5% Pd(PPh_3_)_4_ under argon protection. After reacting at 80°C for 8-10 h, the mixture was filtered and concentrated under vacuum to remove the solvent. Water was added to the residue, and the mixture was extracted three times with ethyl acetate. The combined organic layers were washed with water and brine, dried over sodium sulfate, filtered, and concentrated under vacuum. The crude product was then purified by column chromatography to afford intermediates 3a-3c in 40-65% yield.

### 3.3 General Procedure for Preparing the Target Compounds 4a-4c

To a round-bottom flask equipped with a magnetic stir bar were added intermediates 3a-3c (10 mmol), DMF (50 mL), K₂CO₃ (30 mmol), and 3-bromopropyne (15 mmol). After reacting at room temperature for 10-20 h, the mixture was poured into water and extracted three times with ethyl acetate. The combined organic layers were washed with brine and concentrated *in vacuo*. The products 4a-4c were obtained by column chromatography in 50-60% yields.

### 3.4 General Procedure for Preparing Intermediate 2b

To a round-bottom flask equipped with a magnetic stir bar were added α-bromo-*p*-toluenesulfonyl chloride (1b, 2 mmol), the corresponding substituted amine (3 mmol), Et₃N (5 mmol), and DCM (10 mL). After reacting at room temperature for 1 h, TLC indicated complete consumption of sulfonyl chloride 1b. The organic phase was washed with water and brine, dried over sodium sulfate, filtered, and concentrated under vacuum to afford intermediate 2b in 95-98% yield.

### 3.5 General Procedure for Preparing Intermediate 5

To a round-bottom flask equipped with a magnetic stir bar were added intermediate 2b (2 mmol), 4-hydroxyphenylboronic acid (4 mmol), toluene (4 mL), ethanol (4 mL), sodium carbonate solution (4 mL, 2 M), and 5% Pd(PPh₃)₄ under argon protection. After reacting at 80°C for 8-10 h, the mixture was filtered and concentrated under vacuum to remove the solvent. Water was added to the residue, and the mixture was extracted three times with ethyl acetate. The combined organic layers were washed with water and brine, dried over sodium sulfate, filtered, and concentrated under vacuum. The crude product was purified by column chromatography to afford intermediate 5 in 40-60% yield.

### 3.6 General Procedure for Preparing the Target Compound 6

To a round-bottom flask equipped with a magnetic stir bar were added intermediate 5 (10 mmol), DMF (50 mL), K₂CO₃ (30 mmol), and 3-bromopropyne (15 mmol). After reacting at room temperature for 10-20 h, the mixture was poured into water and extracted three times with ethyl acetate. The combined organic layers were washed with brine and concentrated *in vacuo*. Compound 6 was obtained by column chromatography in 45-60% yields.

### 3.7 General Procedure for Preparing Intermediate 7

To a round-bottom flask equipped with a magnetic stir bar were added intermediate 2b (10 mmol), toluene (50 mL), and triphenylphosphine (10.5 mmol). After reacting at 110°C for 7-10 h, TLC indicated complete consumption of intermediates 2b. The mixture was then filtered and concentrated under vacuum to remove the solvent, affording intermediate 7 in 70% yield.

### 3.8 General Procedure for Preparing the Target Compound 8

To a round-bottom flask equipped with a magnetic stir bar were added intermediate 7 (10 mmol), *t*-BuOK (10 mmol), and THF (50 mL). After stirring at 0°C for 1.5 h, 4-propargyl benzaldehyde (10 mmol) was slowly added. The reaction was continued at 0°C for an additional 2 h, after which the mixture was poured into water and extracted three times with ethyl acetate. The combined organic layers were washed with brine and concentrated *in vacuo*. Compound 8 was obtained by column chromatography in 40-65% yields.

### 3.9 General Procedure for Preparing Intermediate 9

To a round-bottom flask equipped with a magnetic stir bar were added intermediate 2a (2 mmol), CuI (0.2 mmol), Pd(PPh₃)₂Cl₂ (0.2 mmol), and THF (10 mL). After stirring at room temperature for 0.5 h, 4-ethynylphenol (2.4 mmol) was slowly added. The reaction was continued for 8 h, after which the mixture was concentrated under vacuum to remove the solvent. Water was added to the residue, and the mixture was extracted three times with ethyl acetate. The combined organic layers were washed with water and brine, dried over sodium sulfate, filtered, concentrated under vacuum, and purified by column chromatography to afford intermediate 9 in 25-50% yield.

### 3.10 General Procedure for Preparing the Target Compound 10

To a round-bottom flask equipped with a magnetic stir bar were added intermediate 9 (10 mmol), DMF (50 mL), K₂CO₃ (30 mmol), and 3-bromopropyne (15 mmol). After reacting at room temperature for 10-20 h, the mixture was poured into water and extracted three times with ethyl acetate. The combined organic layers were washed with brine and concentrated *in vacuo*. The target compound 10 was obtained by column chromatography in 40-50% yields.

### 3.11 General Procedure for Preparing Intermediate 12

To a round-bottom flask equipped with a magnetic stir bar were added compound 11 (10 mmol), DMF (50 mL), K₂CO₃ (30 mmol), and 3-bromopropyne (15 mmol). After reacting at room temperature for 10-20 h, the mixture was poured into water and extracted three times with ethyl acetate. The combined organic layers were washed with brine and concentrated *in vacuo*. Intermediate 12 was obtained by column chromatography in 88% yield.

### 3.12 General Procedure for Preparing Intermediate 13

To a round-bottom flask equipped with a magnetic stir bar were added intermediate 12 (8 mmol) and DCM (40 mL). After stirring for 0.5 h at 0°C, chlorosulfonic acid (24 mmol) was slowly added. The reaction was continued at 0°C for 5-8 h, after which the mixture was poured into water and extracted three times. The combined organic layers were washed with brine and concentrated *in vacuo*. Intermediate 13 was obtained by column chromatography in 70% yield.

### 3.13 General Procedures for Preparing Intermediate 14

To a round-bottom flask equipped with a magnetic stir bar were added intermediate 13 (2 mmol), the corresponding substituted amine (3 mmol), Et₃N (5 mmol), and DCM (10 mL). After reacting at room temperature for 1 h, TLC indicated complete consumption of sulfonyl chloride 13. The organic phase was washed with water and brine, dried over sodium sulfate, filtered, and concentrated under vacuum to afford intermediates 14 in 95-98% yield.

### 3.14 General Procedure for Preparing the Target Compound S4

To a round-bottom flask equipped with a magnetic stir bar were added intermediate 14 (2 mmol), phenylboronic acid (4 mmol), toluene (4 mL), ethanol (4 mL), a 2 M sodium carbonate solution (4 mL), and 5% Pd(PPh₃)₄ under argon protection. The mixture was stirred at 80°C for 8-10 h. After completion, the reaction mixture was filtered and concentrated under vacuum to remove the solvent. The residue was treated with water and extracted three times with ethyl acetate. The combined organic layers were washed with water and brine, dried over sodium sulfate, filtered, and concentrated under vacuum. The crude product was purified by column chromatography to afford S4 in 50-66% yield.

### 3.15 General Procedure for Preparing Intermediate 16

To a round-bottom flask equipped with a magnetic stir bar were added compound 15 (10 mmol), DMF (50 mL), K₂CO₃ (30 mmol), and 3-bromopropyne (15 mmol). After stirring for 10-20 h at room temperature, the reaction mixture was poured into water and extracted three times with ethyl acetate. The combined organic layers were washed with brine and concentrated *in vacuo***.** Intermediate 16 was obtained by column chromatography in 85% yield.

### 3.16 General Procedure for Preparing Intermediate 17

To a round-bottom flask equipped with a magnetic stir bar were added intermediate 16 (10 mmol) and DCM (40 mL). After stirring for 0.5 h at 0°C, chlorosulfonic acid (24 mmol) was slowly added. The reaction was continued for 5-8 h at 0°C. The crude product was used directly in the next step without further purification.

### 3.17 General Procedure for Preparing Intermediates 18

To a round-bottom flask equipped with a magnetic stir bar were added intermediate 17 (10 mmol) and DCM (40 mL). After stirring for 0.5 h at 0°C, PCl₅ (40 mmol) was slowly added. After reacting for 5-8 h at 0°C, the organic phase was washed with water and brine, dried over sodium sulfate, filtered, and concentrated under vacuum to give intermediate 18 in 65% yield.

### 3.18 General Procedure for Preparing the Target Compound S6

To a round-bottom flask equipped with a magnetic stir bar were added compound 18 (2 mmol), the appropriate substituted amine (3 mmol), Et₃N (5 mmol), and DCM (10 mL). After reacting for 1 h at room temperature, TLC confirmed complete consumption of sulfonyl chloride 18. The organic phase was washed with water and brine, dried over sodium sulfate, filtered, and the solvent was removed under vacuum to afford compound S6 in 95-98% yield.

### 3.19 General Procedure for Preparing Intermediate 20

To a round-bottom flask equipped with a magnetic stir bar were added compound 19 (10 mmol), DMF (50 mL), K₂CO₃ (30 mmol), and 3-bromopropyne (15 mmol). After stirring for 10-20 h at room temperature, the reaction mixture was poured into water and extracted three times with ethyl acetate. The combined organic layers were washed with brine and concentrated *in vacuo*. Intermediate 20 was obtained by column chromatography in 83% yield.

### 3.20 General Procedure for Preparing Intermediate 21

To a round-bottom flask equipped with a magnetic stir bar were added compound 20 (10 mmol) and DCM (40 mL). After stirring for 0.5 h at 0°C, chlorosulfonic acid (24 mmol) was slowly added. The reaction continued for 5-8 h at 0°C. The resulting mixture was poured into water and extracted three times with an appropriate organic solvent. The combined organic layers were washed with brine and concentrated *in vacuo*. Intermediate 21 was obtained by column chromatography in 75% yield.

### 3.21 General Procedure for Preparing Intermediate 22

To a round-bottom flask equipped with a magnetic stir bar were added compound 21 (2 mmol), various substituted amines (3 mmol), Et₃N (5 mmol), and DCM (10 mL). After stirring for 1 h at room temperature, TLC confirmed the complete consumption of sulfonyl chloride 21. The organic phase was washed with water and brine, dried over sodium sulfate, filtered, and the solvent was removed under vacuum to afford intermediate 22 in 90-95% yield.

### 3.22 General Procedure for Preparing Intermediate 23

To a round-bottom flask equipped with a magnetic stir bar were added intermediate 22 (1 mmol), sodium methoxide (3 mmol), and MeOH (5 mL). After stirring for 8-10 h at 65°C, the reaction mixture was concentrated *in vacuo*, poured into water, and extracted three times with ethyl acetate. The combined organic layers were washed with brine and concentrated *in vacuo*. Intermediate 23 was obtained by column chromatography in 40-60% yield.

### 3.23 General Procedure for Preparing the Target Compound S8

To a round-bottom flask equipped with a magnetic stir bar were added intermediate **23** (10 mmol), t-BuOK (10 mmol), and THF (50 mL). After stirring for 1.5 h at 0°C, benzyltriphenylphosphonium bromide (10 mmol) was slowly added. The reaction was continued for an additional 2 h at 0°C. The resulting mixture was then poured into water and extracted three times with ethyl acetate. The combined organic layers were washed with brine and concentrated *in vacuo*. Compound **S8** was obtained by column chromatography in 30-58% yield.

### 3.24 General Procedure for Preparing Intermediate 25

To a round-bottom flask equipped with a magnetic stir bar were added 2-iodophenol (10 mmol), DMF (30 mL), K₂CO₃ (30 mmol), and iodomethane (15 mmol). After reacting for 6-8 h at room temperature, the resulting mixture was poured into water and extracted three times with ethyl acetate. The combined organic layers were washed with brine and concentrated *in vacuo*. Intermediate **25** was obtained by column chromatography in 90% yield.

### 3.25 General Procedure for Preparing Intermediate 26

To a round-bottom flask equipped with a magnetic stir bar were added intermediate 25 (10 mmol) and DCM (40 mL). After stirring for 0.5 h at 0°C, chlorosulfonic acid (24 mmol) was slowly added. The reaction mixture was then stirred for 5-8 h at 0°C, poured into water, and extracted three times. The combined organic layers were washed with brine and concentrated *in vacuo*. Intermediate 26 was obtained by column chromatography in 70% yield.

### 3.26 General Procedure for Preparing Intermediate 27

To a round-bottom flask equipped with a magnetic stir bar were added intermediate 26 (2 mmol), various substituted amines (3 mmol), Et₃N (5 mmol), and DCM (10 mL). After reacting for 1 h at room temperature, TLC confirmed complete consumption of sulfonyl chloride 26. The organic phase was washed with water and brine, dried over sodium sulfate, filtered, and concentrated under vacuum to afford intermediates 27 in 90-95% yield.

### 3.27 General Procedure for Preparing Intermediate 28

To a round-bottom flask equipped with a magnetic stir bar were added intermediate 27 (4 mmol), CuI (0.4 mmol), Pd(PPh₃)₄ (0.12 mmol), Et₃N (5 mmol), and THF (10 mL). After stirring for 0.5 h at room temperature, phenylacetylene (4.3 mmol) was slowly added. The reaction mixture was stirred for an additional 20 h, then filtered through diatomite and concentrated under vacuum. The residue was added to water and extracted three times with ethyl acetate. The combined organic layers were washed with water and brine, dried over sodium sulfate, filtered, and concentrated under vacuum. Purification by column chromatography afforded intermediate 28 in 65-76% yield.

### 3.28 General Procedures for Preparing Intermediates 29

To a round-bottom flask equipped with a magnetic stir bar were added intermediate **28** (1 mmol), DCM (5 mL), and BBr₃ (2 mmol) under argon protection. After reacting for 10 h at 0°C, the organic phase was washed with water and brine, dried over sodium sulfate, filtered, and concentrated under vacuum to provide intermediate **29** in 70-80% yield.

### 3.29 General Procedure for Preparing the Target Compound S10

To a round-bottom flask equipped with a magnetic stir bar were added 29 (10 mmol), DMF (50 mL), K₂CO₃ (30 mmol), and 3-bromopropyne (15 mmol). After reacting for 10-20 h at room temperature, the resulting mixture was poured into water and extracted three times with ethyl acetate. The combined organic layers were washed with brine, then concentrated *in vacuo*. Purification by column chromatography afforded S10 in 50-68% yield.

## 4 STRUCTURAL CHARACTERIZATION OF COMPOUNDS

The chemical structure diagrams of the target compounds are provided in the Characterization Data section of **Supplementary Material S1.docx**. The complete NMR spectra (Figures N1-N110) and mass spectral data (Figs. **M1-M55**) for all compounds are also included in **Supporting Material S1**.

*N*-ethyl-2'-(prop-2-yn-1-yloxy)-[1,1'-biphenyl]-4-sulfonamide (**4a-1**). white solid, yield 27%. m.p.58.6~58.9℃. ^1^H NMR (500 MHz, Chloroform-*d*) δ 7.89 (d, *J* = 6.9 Hz, 2H), 7.68 (d, *J* = 7.3 Hz, 2H), 7.39 (t, *J* = 8.0 Hz, 1H), 7.34 (d, *J* = 7.6 Hz, 1H), 7.15 (d, *J* = 8.1 Hz, 1H), 7.11 (t, *J* = 7.1 Hz, 1H), 4.70 (d, *J* = 2.9 Hz, 2H), 4.46 (t, *J* = 6.2 Hz, 1H), 3.08 (pd, *J* = 7.2, 2.3 Hz, 2H), 2.50 (t, *J* = 2.6 Hz, 1H), 1.15 (t, *J* = 6.6 Hz, 3H). ^13^C NMR (126 MHz, Chloroform-*d*) δ 154.56, 143.01, 131.13, 130.38, 129.72, 129.66, 126.90, 122.26, 113.57, 78.54, 75.94, 56.41, 38.49, 15.34. ESI-MS (MeOH) m/z C_17_H_17_NO_3_S [M + H]^+^ calcd. for:316.10, found:316.16.

2'-(prop-2-yn-1-yloxy)-*N*-propyl-[1,1'-biphenyl]-4-sulfonamide (**4a-2**). white solid, yield 26%. m.p.59.4~61.7℃. ^1^H NMR (500 MHz, Chloroform-*d*) δ 7.89 (d, *J* = 6.7 Hz, 2H), 7.68 (d, *J* = 8.6 Hz, 2H), 7.38 (t, *J* = 8.1 Hz, 1H), 7.34 (d, *J* = 7.6 Hz, 1H), 7.15 (d, *J* = 7.9 Hz, 1H), 7.11 (t, *J* = 6.6 Hz, 1H), 4.69 (s, 2H), 4.56 (d, *J* = 6.7 Hz, 1H), 2.98 (td, *J* = 7.4, 3.6 Hz, 2H), 2.50 (d, *J* = 2.9 Hz, 1H), 1.54 (qd, *J* = 7.4, 2.3 Hz, 2H), 0.90 (t, *J* = 7.1 Hz, 3H). ^13^C NMR (126 MHz, Chloroform-*d*) δ 154.55, 142.95, 138.48, 131.12, 130.36, 129.70, 129.62, 126.86, 122.25, 113.57, 78.53, 75.92, 56.41, 45.20, 23.19, 11.25. ESI-MS (MeOH) m/z C_18_H_19_NO_3_S [M + H]^+^ calcd. for:330.12, found:330.16.

*N*-isopropyl-2'-(prop-2-yn-1-yloxy)-[1,1'-biphenyl]-4-sulfonamide (**4a-3**). white solid, yield 31%. m.p.82.1~83.5℃. ^1^H NMR (500 MHz, Chloroform-*d*) δ 7.96 (d, *J* = 7.2 Hz, 2H), 7.72 (d, *J* = 7.6 Hz, 2H), 7.43 (d, *J* = 7.6 Hz, 1H), 7.39 (d, *J* = 7.9 Hz, 1H), 7.19 (d, *J* = 8.1 Hz, 1H), 7.14 (d, *J* = 6.8 Hz, 1H), 4.73 (s, 2H), 4.66 (d, *J* = 7.5 Hz, 1H), 3.63 - 3.50 (m, 1H), 2.54 (d, *J* = 2.9 Hz, 1H), 1.17 (d, *J* = 7.0 Hz, 6H). ^13^C NMR (126 MHz, Chloroform-*d*) δ 154.54, 142.81, 139.47, 131.11, 130.31, 129.79, 129.66, 126.78, 122.22, 113.59, 78.53, 75.90, 56.40, 46.28, 23.97. ESI-MS (MeOH) m/z C_18_H_19_NO_3_S [M + H]^+^ calcd. for:330.12, found:330.14.

*N*-cyclopropyl-2'-(prop-2-yn-1-yloxy)-[1,1'-biphenyl]-4-sulfonamide (**4a-4**). white solid, yield 31%. m.p.77.4~76.6℃. ^1^H NMR (400 MHz, Chloroform-*d*) δ 7.93 (d, *J* = 8.5 Hz, 2H), 7.70 (d, J = 8.5 Hz, 2H), 7.39 (ddd, *J* = 8.3, 7.3, 1.7 Hz, 1H), 7.35 (dd, *J* = 7.6, 1.7 Hz, 1H), 7.15 (dd, *J* = 8.4, 1.0 Hz, 1H), 7.11 (td, *J* = 7.5, 1.1 Hz, 1H), 5.01 (s, 1H), 4.70 (d, *J* = 2.4 Hz, 2H), 2.50 (t, *J* = 2.4 Hz, 1H), 2.31 (dtdd, *J* = 9.3, 6.9, 4.7, 2.1 Hz, 1H), 0.73 - 0.58 (m, 4H). ^13^C NMR (101 MHz, Chloroform-*d*) δ 154.46, 143.14, 137.85, 131.12, 130.32, 130.07, 129.74, 127.19, 122.20, 113.43, 78.48, 75.96, 56.31, 24.52, 6.39. ESI-MS (MeOH) m/z C_18_H_19_NO_3_S [M + H]^+^ calcd. for:328.10, found:328.13.

*N*-isobutyl-2'-(prop-2-yn-1-yloxy)-[1,1'-biphenyl]-4-sulfonamide (**4a-5**). white solid, yield 24%. m.p.75.8~76.2℃. ^1^H NMR (500 MHz, Chloroform-*d*) δ 7.89 (d, *J* = 7.9 Hz, 2H), 7.68 (dd, *J* = 8.3, 2.7 Hz, 2H), 7.41 - 7.36 (m, 1H), 7.34 (d, *J* = 7.3 Hz, 1H), 7.15 (d, *J* = 8.1 Hz, 1H), 7.10 (d, *J* = 7.1 Hz, 1H), 4.80 (dd, *J* = 17.7, 10.3 Hz, 1H), 4.69 (s, 2H), 2.82 (q, *J* = 4.1 Hz, 2H), 2.50 (d, *J* = 2.9 Hz, 1H), 1.77 (dtd, *J* = 11.4, 6.8, 3.4 Hz, 1H), 0.90 (d, *J* = 5.7 Hz, 6H). ^13^C NMR (126 MHz, Chloroform-*d*) δ 154.50, 142.87, 138.46, 131.10, 130.33, 129.72, 129.67, 126.81, 122.21, 113.51, 75.93, 56.35, 50.76, 28.66, 20.01. ESI-MS (MeOH) m/z C_19_H_21_NO_3_S [M + H]^+^ calcd. for:344.13, found:344.16.

*N*-(sec-butyl)-2'-(prop-2-yn-1-yloxy)-[1,1'-biphenyl]-4-sulfonamide (**4a-6**). white solid, yield 28%. m.p. 82.6~86.4℃. ^1^H NMR (500 MHz, Chloroform-*d*) δ 7.91 (d, *J* = 7.6 Hz, 2H), 7.67 (d, *J* = 7.1 Hz, 2H), 7.38 (t, *J* = 7.8 Hz, 1H), 7.34 (d, *J* = 7.9 Hz, 1H), 7.14 (d, *J* = 8.1 Hz, 1H), 7.10 (d, *J* = 6.6 Hz, 1H), 4.67 (d, *J* = 2.5 Hz, 2H), 4.55 (dd, *J* = 23.4, 7.9 Hz, 1H), 3.32 (p, *J* = 7.0 Hz, 1H), 2.48 (t, *J* = 2.6 Hz, 1H), 1.44 (p, *J* = 7.7 Hz, 2H), 1.07 (d, *J* = 6.6 Hz, 3H), 0.83 (t, *J* = 7.3 Hz, 3H).^13^C NMR (126 MHz, Chloroform-*d*) δ 154.55, 142.77, 139.60, 131.09, 130.27, 129.83, 129.65, 126.77, 122.23, 113.64, 78.52, 75.87, 56.43, 51.59, 30.47, 21.36, 10.04. ESI-MS (MeOH) m/z C_19_H_21_NO_3_S [M + H]^+^ calcd. for:344.13, found:344.17.

*N*-(tert-butyl)-2'-(prop-2-yn-1-yloxy)-[1,1'-biphenyl]-4-sulfonamide (**4a-7**). white solid, yield 30%. m.p.110.4~110.9℃. ^1^H NMR (500 MHz, Chloroform-*d*) δ 7.98 (d, *J* = 7.3 Hz, 2H), 7.70 (d, *J* = 7.6 Hz, 2H), 7.42 (t, *J* = 8.0 Hz, 1H), 7.39 (d, *J* = 7.7 Hz, 1H), 7.19 (d, *J* = 8.3 Hz, 1H), 7.14 (t, *J* = 7.0 Hz, 1H), 5.14 (s, 1H), 4.72 (d, *J* = 2.5 Hz, 2H), 2.53 (t, *J* = 2.5 Hz, 1H), 1.31 (s, 9H). ^13^C NMR (126 MHz, Chloroform-*d*) δ 154.52, 142.40, 141.81, 131.08, 130.09, 129.84, 129.55, 126.67, 122.17, 113.60, 78.54, 75.86, 56.36, 54.79, 30.30. ESI-MS (MeOH) m/z C_19_H_21_NO_3_S [M + H]^+^ calcd. for:344.13, found:344.18.

*N*-pentyl-2'-(prop-2-yn-1-yloxy)-[1,1'-biphenyl]-4-sulfonamide (**4a-8**). white solid, yield 27%. m.p.58.2~59.7℃. ^1^H NMR (500 MHz, Chloroform-*d*) δ 7.94 (d, *J* = 7.4 Hz, 2H), 7.72 (d, *J* = 7.9 Hz, 2H), 7.43 (t, *J* = 8.0 Hz, 1H), 7.38 (d, *J* = 7.6 Hz, 1H), 7.20 (d, *J* = 8.3 Hz, 1H), 7.15 (t, *J* = 7.5 Hz, 1H), 4.76 (t, *J* = 6.6 Hz, 1H), 4.73 (d, *J* = 2.9 Hz, 2H), 3.05 (q, *J* = 6.5 Hz, 2H), 2.54 (t, *J* = 2.9 Hz, 1H), 1.60 - 1.49 (m, 2H), 1.36 - 1.24 (m, 4H), 0.89 (t, *J* = 3.8, 2.7 Hz, 3H). ^13^C NMR (126 MHz, Chloroform-*d*) δ 154.48, 142.90, 138.38, 131.10, 130.33, 129.70, 129.67, 126.84, 122.19, 113.47, 78.50, 75.93, 56.31, 43.42, 29.42, 28.77, 22.27, 13.99. ESI-MS (MeOH) m/z C_20_H_23_NO_3_S [M + H]^+^ calcd. for:358.15, found:358.19.

*N*-hexyl-2'-(prop-2-yn-1-yloxy)-[1,1'-biphenyl]-4-sulfonamide (**4a-9**). white solid, yield 29%. m.p.49.8~51.5℃^1^H NMR (400 MHz, Chloroform-*d*) δ 7.89 (d, *J* = 8.5 Hz, 2H), 7.68 (d, *J* = 8.5 Hz, 2H), 7.42 - 7.35 (m, 1H), 7.34 (dd, *J* = 7.6, 1.7 Hz, 1H), 7.15 (dd, *J* = 8.4, 1.0 Hz, 1H), 7.11 (td, *J* = 7.5, 1.1 Hz, 1H), 4.70 (d, *J* = 2.4 Hz, 3H), 3.00 (q, *J* = 6.8 Hz, 2H), 2.50 (t, *J* = 2.4 Hz, 1H), 1.56 - 1.38 (m, 2H), 1.35 - 1.17 (m, 6H), 0.85 (t, *J* = 6.8 Hz, 3H). ^13^C NMR (101 MHz, Chloroform-*d*) δ 154.44, 142.89, 138.30, 131.11, 130.33, 129.68, 129.64, 126.85, 122.18, 113.39, 78.48, 75.95, 56.27, 43.45, 31.38, 29.70, 26.32, 22.58, 14.09. ESI-MS (MeOH) m/z C_21_H_25_NO_3_S [M + H]^+^ calcd. for:372.16, found:372.21.

*N,N*-dimethyl-2'-(prop-2-yn-1-yloxy)-[1,1'-biphenyl]-4-sulfonamide (**4a-10**). white solid, yield 33%. m.p.91.5~93.8℃. ^1^H NMR (500 MHz, Chloroform-*d*) δ 7.85 (d, *J* = 7.2 Hz, 2H), 7.75 (d, *J* = 8.5 Hz, 2H), 7.43 (t, *J* = 8.0 Hz, 1H), 7.39 (d, *J* = 7.7 Hz, 1H), 7.20 (d, *J* = 9.0 Hz, 1H), 7.18 - 7.13 (m, 1H), 4.75 (d, *J* = 2.6 Hz, 2H), 2.80 (s, 6H), 2.55 (t, *J* = 2.5 Hz, 1H). ^13^C NMR (126 MHz, Chloroform-*d*) δ 154.45, 142.99, 133.93, 131.10, 130.23, 129.71, 129.58, 127.53, 122.20, 113.45, 78.48, 75.94, 56.30, 38.09. ESI-MS (MeOH) m/z C_17_H_17_NO_3_S [M + H]^+^ calcd. for:316.10, found:316.13.

*N,N*-diethyl-2'-(prop-2-yn-1-yloxy)-[1,1'-biphenyl]-4-sulfonamide (**4a-11**). white solid, yield 26%. m.p.85.1~88.4℃. ^1^H NMR (500 MHz, Chloroform-*d*) δ 7.87 (d, *J* = 7.2 Hz, 2H), 7.70 (d, *J* = 7.4 Hz, 2H), 7.42 (t, *J* = 8.0 Hz, 1H), 7.38 (d, *J* = 7.7 Hz, 1H), 7.19 (d, *J* = 8.1 Hz, 1H), 7.15 (t, *J* = 7.0 Hz, 1H), 4.73 (d, *J* = 2.8 Hz, 2H), 3.33 (qd, *J* = 7.2, 2.4 Hz, 4H), 2.54 (d, *J* = 2.6 Hz, 1H), 1.22 (t, *J* = 6.8 Hz, 6H). ^13^C NMR (126 MHz, Chloroform-*d*) δ 154.48, 142.52, 138.69, 131.09, 130.22, 129.76, 129.60, 126.79, 122.19, 113.52, 78.51, 75.88, 56.34, 42.33, 14.44. ESI-MS (MeOH) m/z C_19_H_21_NO_3_S [M + H]^+^ calcd. for:344.13, found:344.13.

*N*-cyclohexyl-2'-(prop-2-yn-1-yloxy)-[1,1'-biphenyl]-4-sulfonamide (**4a-12**). white solid, yield 23%. m.p.66.7~67.9℃. ^1^H NMR (400 MHz, Chloroform-*d*) δ 7.91 (d, *J* = 8.4 Hz, 2H), 7.67 (d, *J* = 8.4 Hz, 2H), 7.41 - 7.36 (m, 1H), 7.36 - 7.33 (m, 1H), 7.15 (d, *J* = 8.2 Hz, 1H), 7.11 (td, *J* = 7.5, 1.1 Hz, 1H), 4.74 (d, *J* = 7.6 Hz, 1H), 4.69 (d, *J* = 2.4 Hz, 2H), 3.20 (tdt, *J* = 11.3, 7.9, 3.9 Hz, 1H), 2.49 (t, *J* = 2.4 Hz, 1H), 1.81 (dt, *J* = 12.1, 3.7 Hz, 2H), 1.66 (dq, *J* = 12.8, 3.8 Hz, 2H), 1.57 - 1.12 (m, 6H). ^13^C NMR (101 MHz, Chloroform-*d*) δ 154.48, 142.68, 139.75, 131.12, 130.28, 129.72, 129.65, 126.67, 122.20, 113.50, 78.50, 75.91, 56.34, 52.78, 34.11, 25.28, 24.76. ESI-MS (MeOH) m/z C_21_H_23_NO_3_S [M + H]^+^ calcd. for:370.15, found:370.20.

*N*-allyl-2'-(prop-2-yn-1-yloxy)-[1,1'-biphenyl]-4-sulfona-mide (**4a-13**). white solid, yield 28%. m.p.120.7~125.1℃. ^1^H NMR (500 MHz, Chloroform-*d*) δ 7.94 (d, *J* = 8.5 Hz, 2H), 7.73 (d, *J* = 8.5 Hz, 2H), 7.47 - 7.40 (m, 1H), 7.38 (dd, *J* = 7.6, 1.8 Hz, 1H), 7.19 (d, *J* = 8.3 Hz, 1H), 7.15 (t, *J* = 7.3 Hz, 1H), 5.82 (ddt, *J* = 16.4, 10.1, 5.8 Hz, 1H), 5.30 - 5.13 (m, 2H), 4.80 (t, *J* = 6.3 Hz, 1H), 4.74 (d, *J* = 2.4 Hz, 2H), 3.70 (td, *J* = 5.2, 4.4, 3.0 Hz, 2H), 2.55 (t, *J* = 2.4 Hz, 1H).^13^C NMR (126 MHz, Chloroform-*d*) δ 154.48, 143.07, 138.27, 133.17, 131.09, 130.39, 129.72, 129.64, 126.90, 122.21, 117.87, 113.47, 78.48, 75.95, 56.33, 45.96. ESI-MS (MeOH) m/z C_18_H_17_NO_3_S [M + H]^+^ calcd. for:328.10, found:328.13.

3'-(prop-2-yn-1-yloxy)-*N*-propyl-[1,1'-biphenyl]-4-sulfo-namide (**4b-1**). white solid, yield 28%. m.p.49.8~51.5℃. ^1^H NMR (400 MHz, Chloroform-*d*) δ 7.93 (d, *J* = 8.6 Hz, 2H), 7.71 (d, *J* = 8.5 Hz, 2H), 7.41 (t, *J* = 7.9 Hz, 1H), 7.25 - 7.19 (m, 2H), 7.04 (ddd, *J* = 8.3, 2.6, 0.9 Hz, 1H), 4.77 (d, *J* = 2.4 Hz, 2H), 4.65 (q, *J* = 7.4, 6.2 Hz, 1H), 3.03 - 2.88 (m, 2H), 2.56 (t, *J* = 2.4 Hz, 1H), 1.52 (h, *J* = 7.3 Hz, 2H), 0.89 (t, *J* = 7.4 Hz, 3H). ^13^C NMR (101 MHz, Chloroform-*d*) δ 158.14, 145.23, 140.98, 138.93, 130.26, 127.89, 127.70, 120.76, 114.77, 114.31, 78.46, 75.99, 56.03, 45.16, 23.12, 11.25. ESI-MS (MeOH) m/z C_18_H_19_NO_3_S [M + H]^+^ calcd. for:330.12, found:330.15.

*N*-isopropyl-3'-(prop-2-yn-1-yloxy)-[1,1'-biphenyl]-4-sulfonamide (**4b-2**). white solid, yield 29%. m.p. 80.4~83.5℃. ^1^H NMR (500 MHz, Chloroform-*d*) δ 7.94 (dd, *J* = 8.2, 2.4 Hz, 2H), 7.74 - 7.67 (m, 2H), 7.41 (td, *J* = 8.0, 2.2 Hz, 1H), 7.23 (dd, *J* = 13.8, 5.2 Hz, 2H), 7.04 (dd, *J* = 8.3, 2.8 Hz, 1H), 4.76 (d, *J* = 2.5 Hz, 2H), 4.48 (d, *J* = 7.5 Hz, 1H), 3.52 (q, *J* = 7.0 Hz, 1H), 2.55 (t, *J* = 2.5 Hz, 1H), 1.12 (dd, *J* = 6.5, 2.3 Hz, 6H). ^13^C NMR (126 MHz, Chloroform-*d*) δ 158.20, 145.14, 141.02, 140.13, 130.25, 127.85, 127.65, 120.78, 114.83, 114.37, 78.51, 75.97, 56.09, 46.33, 23.98. ESI-MS (MeOH) m/z C_18_H_19_NO_3_S [M + H]^+^ calcd. for:330.12, found:330.12.

*N*-isobutyl-3'-(prop-2-yn-1-yloxy)-[1,1'-biphenyl]-4-sulfonamide （**4b-3**）. white solid, yield 26%. m.p.51.4~55.7℃. ^1^H NMR (500 MHz, Chloroform-*d*) δ 7.97 (d, *J* = 7.9 Hz, 2H), 7.75 (d, *J* = 6.1 Hz, 2H), 7.46 (td, *J* = 8.0, 2.3 Hz, 1H), 7.28 (dd, *J* = 15.6, 7.7 Hz, 2H), 7.09 (d, *J* = 8.1 Hz, 1H), 4.81 (d, *J* = 2.7 Hz, 2H), 4.66 (q, *J* = 10.0, 6.0 Hz, 1H), 2.86 (td, *J* = 6.7, 2.2 Hz, 2H), 2.60 (d, *J* = 2.6 Hz, 1H), 1.80 (dtt, *J* = 13.4, 6.8, 3.7 Hz, 1H), 0.94 (d, *J* = 5.7 Hz, 6H). ^13^C NMR (126 MHz, Chloroform-*d*) δ 158.20, 145.24, 141.02, 139.08, 130.26, 127.89, 127.69, 120.78, 114.83, 114.37, 78.49, 75.97, 56.08, 50.78, 28.66, 20.01. ESI-MS (MeOH) m/z C_19_H_21_NO_3_S [M + H]^+^ calcd. for:344.13, found:344.15.

*N*-(sec-butyl)-3'-(prop-2-yn-1-yloxy)-[1,1'-biphenyl]-4-sulfonamide (**4b-4**). white solid, yield 27%. m.p.81.7~84.4℃. ^1^H NMR (500 MHz, Chloroform-*d*) δ 7.99 (d, *J* = 7.5 Hz, 2H), 7.74 (d, *J* = 7.7 Hz, 2H), 7.45 (t, *J* = 7.8 Hz, 1H), 7.29 (d, *J* = 8.1 Hz, 2H), 7.08 (d, *J* = 8.2 Hz, 1H), 4.80 (d, *J* = 2.7 Hz, 2H), 4.72 (t, *J* = 10.4 Hz, 1H), 3.35 (p, *J* = 7.9 Hz, 1H), 2.60 (t, *J* = 2.8 Hz, 1H), 1.48 (pd, *J* = 7.2, 2.2 Hz, 2H), 1.10 (d, *J* = 5.9 Hz, 3H), 0.87 (d, *J* = 7.1 Hz, 3H). ^13^C NMR (126 MHz, Chloroform-*d*) δ 158.16, 145.01, 140.99, 140.25, 130.22, 127.76, 127.63, 120.74, 114.79, 114.33, 78.49, 75.96, 56.06, 51.63, 30.42, 21.32, 10.05. ESI-MS (MeOH) m/z C_19_H_21_NO_3_S [M + H]^+^ calcd. for:344.13, found:344.20.

*N*-pentyl-3'-(prop-2-yn-1-yloxy)-[1,1'-biphenyl]-4-sulfonamide (**4b-5**). white solid, yield 23%. m.p.52.2~55.3℃. ^1^H NMR (500 MHz, Chloroform-*d*) δ 7.93 (d, *J* = 7.5 Hz, 2H), 7.71 (d, *J* = 7.4 Hz, 2H), 7.41 (t, *J* = 7.8 Hz, 1H), 7.27 - 7.19 (m, 2H), 7.04 (d, *J* = 7.7 Hz, 1H), 4.76 (d, *J* = 2.8 Hz, 2H), 4.58 (d, *J* = 6.5 Hz, 1H), 3.04 - 2.94 (m, 2H), 2.56 (d, *J* = 2.9 Hz, 1H), 1.56 - 1.42 (m, 2H), 1.26 (dt, *J* = 8.1, 4.0 Hz, 4H), 0.84 (h, *J* = 2.8 Hz, 3H). ^13^C NMR (126 MHz, Chloroform-*d*) δ 158.19, 145.27, 141.03, 139.01, 130.26, 127.89, 127.72, 120.78, 114.81, 114.36, 78.49, 75.97, 56.07, 43.43, 29.44, 28.78, 22.27, 14.00. ESI-MS (MeOH) m/z C_20_H_23_NO_3_S [M + H]^+^ calcd. for:358.15, found:358.21.

*N,N*-dimethyl-3'-(prop-2-yn-1-yloxy)-[1,1'-biphenyl]-4-sulfonamide (**4b-6**). white solid, yield 29%. m.p.93.3~93.8℃. ^1^H NMR (500 MHz, Chloroform-*d*) δ 7.88 (d, *J* = 8.4 Hz, 2H), 7.78 (d, *J* = 7.6 Hz, 2H), 7.46 (t, *J* = 7.4 Hz, 1H), 7.30 - 7.24 (m, 2H), 7.09 (d, *J* = 8.1 Hz, 1H), 4.81 (d, *J* = 2.6 Hz, 2H), 2.80 (s, 6H), 2.60 (t, *J* = 2.6 Hz, 1H). ^13^C NMR (126 MHz, Chloroform-*d*) δ 158.21, 145.35, 140.97, 134.57, 130.28, 128.41, 127.81, 120.78, 114.85, 114.37, 78.49, 75.98, 56.08, 38.11. ESI-MS (MeOH) m/z C_17_H_17_NO_3_S [M + H]^+^ calcd. for:316.10, found:316.14.

*N,N*-diethyl-3'-(prop-2-yn-1-yloxy)-[1,1'-biphenyl]-4-sulfonamide (**4b-7**). white solid, yield 29%. m.p.85.5~87.4℃. ^1^H NMR (500 MHz, Chloroform-*d*) δ 7.90 (d, *J* = 8.0 Hz, 2H), 7.72 (d, *J* = 6.8 Hz, 2H), 7.44 (t, *J* = 7.9 Hz, 1H), 7.30 - 7.23 (m, 2H), 7.07 (d, *J* = 8.1 Hz, 1H), 4.80 (d, *J* = 2.7 Hz, 2H), 3.32 (qd, *J* = 7.2, 2.4 Hz, 4H), 2.60 (t, *J* = 2.7 Hz, 1H), 1.20 (t, *J* = 6.7 Hz, 6H). ^13^C NMR (126 MHz, Chloroform-*d*) δ 158.13, 144.82, 141.00, 139.34, 130.19, 127.74, 127.61, 120.70, 114.71, 114.27, 78.48, 75.94, 56.02, 42.23, 14.33. ESI-MS (MeOH) m/z C_19_H_21_NO_3_S [M + H]^+^ calcd. for:344.13, found:344.18.

*N*-ethyl-4'-(prop-2-yn-1-yloxy)-[1,1'-biphenyl]-4-sulfon-amide (**4c-1**). white solid, yield 28%. m.p.75.1~77.4℃. ^1^H NMR (500 MHz, Chloroform-*d*) δ 7.95 (d, *J* = 8.5 Hz, 2H), 7.72 (d, *J* = 8.5 Hz, 2H), 7.61 (d, *J* = 8.8 Hz, 2H), 7.13 (d, *J* = 8.8 Hz, 2H), 4.80 (d, *J* = 2.4 Hz, 2H), 4.64 (t, *J* = 6.0 Hz, 1H), 3.10 (qd, *J* = 7.2, 6.0 Hz, 2H), 2.61 (t, *J* = 2.4 Hz, 1H), 1.18 (t, *J* = 7.3 Hz, 3H). ^13^C NMR (126 MHz, Chloroform-*d*) δ 158.07, 145.08, 138.08, 132.72, 128.58, 127.76, 127.34, 115.58, 78.39, 75.98, 56.00, 38.43, 15.25. ESI-MS (MeOH) m/z C_17_H_17_NO_3_S [M + H]^+^ calcd. for:316.10, found:316.14.

4'-(prop-2-yn-1-yloxy)-*N*-propyl-[1,1'-biphenyl]-4-sulfo-namide (**4c-2**). white solid, yield 27%. m.p.65.1~66.4℃. ^1^H NMR (500 MHz, Chloroform-*d*) δ 7.95 (d, *J* = 8.5 Hz, 2H), 7.72 (d, *J* = 8.5 Hz, 2H), 7.61 (d, *J* = 8.8 Hz, 2H), 7.13 (d, *J* = 8.8 Hz, 2H), 4.80 (d, *J* = 2.4 Hz, 2H), 4.55 (t, *J* = 6.3 Hz, 1H), 3.01 (q, *J* = 6.8 Hz, 2H), 2.60 (t, *J* = 2.4 Hz, 1H), 1.56 (h, *J* = 7.3 Hz, 2H), 0.94 (t, *J* = 7.4 Hz, 3H). ^13^C NMR (126 MHz, Chloroform-*d*) δ 158.08, 145.06, 138.17, 132.75, 128.59, 127.74, 127.34, 115.59, 78.40, 75.98, 56.01, 45.16, 23.14, 11.26. ESI-MS (MeOH) m/z C_18_H_19_NO_3_S [M + H]^+^ calcd. for:330.12, found:330.16.

*N*-isopropyl-4'-(prop-2-yn-1-yloxy)-[1,1'-biphenyl]-4-sulfonamide (**4c-3**). white solid, yield 27%. m.p.83.4~83.9℃. ^1^H NMR (500 MHz, Chloroform-*d*) δ 7.96 (d, *J* = 8.4 Hz, 2H), 7.72 (d, *J* = 8.4 Hz, 2H), 7.61 (d, *J* = 8.8 Hz, 2H), 7.13 (d, *J* = 8.8 Hz, 2H), 4.80 (d, *J* = 2.4 Hz, 2H), 4.59 (d, *J* = 7.5 Hz, 1H), 3.62 - 3.47 (m, 1H), 2.60 (t, *J* = 2.4 Hz, 1H), 1.16 (d, *J* = 6.5 Hz, 6H). ^13^C NMR (126 MHz, Chloroform-*d*) δ 158.10, 144.93, 139.34, 132.79, 128.58, 127.68, 127.28, 115.62, 78.43, 75.95, 56.05, 46.29, 23.97. ESI-MS (MeOH) m/z C_18_H_19_NO_3_S [M + H]^+^ calcd. for:330.12, found:330.14.

*N*-cyclopropyl-4'-(prop-2-yn-1-yloxy)-[1,1'-biphenyl]-4-sulfonamide (**4c-4**). white solid, yield 26%. m.p.72.5~74.8℃. ^1^H NMR (500 MHz, Chloroform-*d*) δ 7.99 (d, *J* = 8.4 Hz, 2H), 7.74 (d, *J* = 8.5 Hz, 2H), 7.62 (d, *J* = 8.8 Hz, 2H), 7.14 (d, *J* = 8.8 Hz, 2H), 4.92 (s, 1H), 4.81 (d, *J* = 2.4 Hz, 2H), 2.61 (t, *J* = 2.4 Hz, 1H), 2.34 (dp, *J* = 10.4, 3.7 Hz, 1H), 0.68 (ddt, *J* = 12.0, 5.2, 2.5 Hz, 4H). ^13^C NMR (126 MHz, Chloroform-*d*) δ 158.12, 145.27, 137.76, 132.73, 128.62, 128.12, 127.29, 115.61, 78.39, 75.99, 56.02, 24.49, 6.39. ESI-MS (MeOH) m/z C_18_H_19_NO_3_S [M + H]^+^ calcd. for:328.10, found:328.10.

*N*-butyl-4'-(prop-2-yn-1-yloxy)-[1,1'-biphenyl]-4-sulfon-amide (**4c-5**). white solid, yield 29%. m.p.56.8~59.2℃. ^1^H NMR (500 MHz, Chloroform-*d*) δ 7.98 - 7.92 (m, 2H), 7.72 (d, *J* = 8.4 Hz, 2H), 7.64 - 7.59 (m, 2H), 7.13 (d, *J* = 8.7 Hz, 2H), 4.80 (d, *J* = 2.4 Hz, 2H), 4.60 (t, *J* = 6.1 Hz, 1H), 3.04 (q, *J* = 6.8 Hz, 2H), 2.60 (t, *J* = 2.4 Hz, 1H), 1.51 (p, *J* = 7.2 Hz, 2H), 1.36 (h, *J* = 7.4 Hz, 2H), 0.91 (t, *J* = 7.3 Hz, 3H). ^13^C NMR (126 MHz, Chloroform-*d*) δ 158.12, 145.07, 138.22, 132.79, 128.59, 127.75, 127.33, 115.63, 78.43, 75.96, 56.05, 43.13, 31.80, 19.84, 13.66. ESI-MS (MeOH) m/z C_19_H_21_NO_3_S [M + H]^+^ calcd. for:344.13, found:344.15.

*N*-isobutyl-4'-(prop-2-yn-1-yloxy)-[1,1'-biphenyl]-4-sulfonamide (**4c-6**). white solid, yield 29%. m.p.66.4~66.9℃. ^1^H NMR (500 MHz, Chloroform-*d*) δ 7.95 (d, *J* = 8.4 Hz, 2H), 7.72 (d, *J* = 8.5 Hz, 2H), 7.61 (d, *J* = 8.8 Hz, 2H), 7.13 (d, *J* = 8.8 Hz, 2H), 4.80 (d, *J* = 2.4 Hz, 2H), 4.73 (t, *J* = 6.5 Hz, 1H), 2.85 (t, *J* = 6.6 Hz, 2H), 2.60 (t, *J* = 2.4 Hz, 1H), 1.79 (dp, *J* = 13.4, 6.7 Hz, 1H), 0.94 (d, *J* = 6.7 Hz, 6H). ^13^C NMR (126 MHz, Chloroform-*d*) δ 158.12, 145.03, 138.30, 132.78, 128.59, 127.72, 127.33, 115.63, 78.43, 75.96, 56.05, 50.77, 28.65, 20.01. ESI-MS (MeOH) m/z C_19_H_21_NO_3_S [M + H]^+^ calcd. for:344.13, found:344.17.

*N*-(sec-butyl)-4'-(prop-2-yn-1-yloxy)-[1,1'-biphenyl]-4-sulfonamide (**4c-7**). white solid, yield 27%. m.p.74.2~77.4℃. ^1^H NMR (500 MHz, Chloroform-*d*) δ 7.97 (d, *J* = 8.5 Hz, 2H), 7.72 (d, *J* = 8.5 Hz, 2H), 7.62 (d, *J* = 8.7 Hz, 2H), 7.13 (d, *J* = 8.8 Hz, 2H), 4.80 (d, *J* = 2.4 Hz, 2H), 4.64 - 4.49 (m, 1H), 3.34 (dp, *J* = 7.9, 6.5 Hz, 1H), 2.60 (t, *J* = 2.4 Hz, 1H), 1.53 - 1.40 (m, 2H), 1.10 (d, *J* = 6.6 Hz, 3H), 0.87 (t, *J* = 7.4 Hz, 3H). ^13^C NMR (126 MHz, Chloroform-*d*) δ 158.06, 144.83, 139.45, 132.76, 128.56, 127.66, 127.21, 115.60, 78.41, 75.95, 56.03, 51.61, 30.44, 21.36, 21.35, 10.06. ESI-MS (MeOH) m/z C_19_H_21_NO_3_S [M + H]^+^ calcd. for:344.13, found:344.18.

*N*-(tert-butyl)-4'-(prop-2-yn-1-yloxy)-[1,1'-biphenyl]-4-sulfonamide (**4c-8**). white solid, yield 24%. m.p.92.5~97.7℃. ^1^H NMR (500 MHz, Chloroform-*d*) δ 7.98 (d, *J* = 8.4 Hz, 2H), 7.74 - 7.65 (m, 2H), 7.62 (d, *J* = 8.7 Hz, 2H), 7.13 (d, *J* = 8.7 Hz, 2H), 4.80 (d, *J* = 2.5 Hz, 3H), 2.60 (t, *J* = 2.4 Hz, 1H), 1.30 (s, 10H). ^13^C NMR (126 MHz, Chloroform-*d*) δ 157.99, 144.56, 141.59, 132.83, 128.55, 127.62, 127.11, 115.54, 78.42, 75.95, 56.00, 54.84, 30.33. ESI-MS (MeOH) m/z C_19_H_21_NO_3_S [M + H]^+^ calcd. for:344.13, found:344.13.

*N*-pentyl-4'-(prop-2-yn-1-yloxy)-[1,1'-biphenyl]-4-sulfo-namide (**4c-9**). white solid, yield 28%. m.p.55.7~58.4℃. ^1^H NMR (500 MHz, Chloroform-*d*) δ 7.95 (d, *J* = 8.5 Hz, 2H), 7.72 (d, *J* = 8.5 Hz, 2H), 7.61 (d, *J* = 8.7 Hz, 2H), 7.13 (d, *J* = 8.8 Hz, 2H), 4.80 (d, *J* = 2.4 Hz, 2H), 4.52 (t, *J* = 6.2 Hz, 1H), 3.03 (q, *J* = 6.9 Hz, 2H), 2.60 (t, *J* = 2.4 Hz, 1H), 1.59 - 1.44 (m, 2H), 1.36 - 1.24 (m, *J* = 2.3 Hz, 5H), 0.96 - 0.82 (m, 3H). ^13^C NMR (126 MHz, Chloroform-*d*) δ 158.08, 145.07, 138.15, 132.77, 128.59, 127.75, 127.34, 115.59, 78.40, 75.98, 56.01, 43.41, 29.44, 28.78, 22.28, 14.02. ESI-MS (MeOH) m/z C_20_H_23_NO_3_S [M + H]^+^ calcd. for:358.12, found:358.19.

*N*-hexyl-4'-(prop-2-yn-1-yloxy)-[1,1'-biphenyl]-4-sulfo-namide (**4c-10**). white solid, yield 26%. m.p.63.1~63.9℃. ^1^H NMR (400 MHz, Chloroform-*d*) δ 7.90 (d, *J* = 8.4 Hz, 2H), 7.68 (d, *J* = 8.4 Hz, 2H), 7.57 (d, *J* = 8.7 Hz, 2H), 7.09 (d, *J* = 8.7 Hz, 2H), 4.76 (d, *J* = 2.4 Hz, 2H), 4.61 (t, *J* = 6.2 Hz, 1H), 2.98 (q, *J* = 6.8 Hz, 2H), 2.56 (t, *J* = 2.4 Hz, 1H), 1.47 (p, *J* = 7.0 Hz, 2H), 1.31 - 1.15 (m, 6H), 0.84 (t, *J* = 6.8 Hz, 3H).^13^C NMR (101 MHz, Chloroform-*d*) δ 158.05, 145.03, 138.11, 132.74, 128.57, 127.74, 127.32, 115.56, 78.38, 75.98, 55.99, 43.42, 31.36, 29.68, 26.31, 22.58, 14.08. ESI-MS (MeOH) m/z C_21_H_25_NO_3_S [M + H]^+^ calcd. for:372.16, found:372.23.

*N,N*-dimethyl-4'-(prop-2-yn-1-yloxy)-[1,1'-biphenyl]-4-sulfonamide (**4c-11**). white solid, yield 27%. m.p.95.1~96.6℃. ^1^H NMR (500 MHz, Chloroform-*d*) δ 7.86 (d, *J* = 8.5 Hz, 2H), 7.75 (d, *J* = 8.4 Hz, 2H), 7.62 (d, *J* = 8.8 Hz, 2H), 7.14 (d, *J* = 8.8 Hz, 2H), 4.80 (d, *J* = 2.4 Hz, 2H), 2.79 (s, 6H), 2.61 (t, *J* = 2.4 Hz, 1H). ^13^C NMR (126 MHz, Chloroform-*d*) δ 158.11, 145.14, 135.65, 132.70, 128.60, 128.44, 127.24, 115.60, 78.39, 75.98, 56.01, 38.13. ESI-MS (MeOH) m/z C_17_H_17_NO_3_S [M + H]^+^ calcd. for:316.10, found:316.13.

*N,N*-diethyl-4'-(prop-2-yn-1-yloxy)-[1,1'-biphenyl]-4-sulfonamide (**4c-12**). white solid, yield 27%. m.p.88.4~89.7℃. ^1^H NMR (500 MHz, Chloroform-*d*) δ 7.89 (d, *J* = 8.4 Hz, 2H), 7.70 (d, *J* = 8.5 Hz, 2H), 7.61 (d, *J* = 8.7 Hz, 2H), 7.12 (d, *J* = 8.8 Hz, 2H), 4.80 (d, *J* = 2.4 Hz, 2H), 3.31 (q, *J* = 7.2 Hz, 5H), 2.60 (t, *J* = 2.4 Hz, 1H), 1.20 (t, *J* = 7.2 Hz, 7H). ^13^C NMR (126 MHz, Chloroform-*d*) δ 158.00, 144.64, 138.54, 132.78, 128.53, 127.66, 127.21, 115.54, 78.40, 75.95, 55.99, 42.22, 14.34. ESI-MS (MeOH) m/z C_19_H_21_NO_3_S [M + H]^+^ calcd. for:344.13, found:344.17.

*N*-cyclohexyl-4'-(prop-2-yn-1-yloxy)-[1,1'-biphenyl]-4-sulfonamide (**4c-13**). white solid, yield 29%. m.p.55.7~59.2℃. ^1^H NMR (400 MHz, Chloroform-*d*) δ 7.93 (d, *J* = 8.1 Hz, 2H), 7.67 (d, *J* = 8.0 Hz, 2H), 7.62 - 7.50 (m, 2H), 7.08 (d, *J* = 8.3 Hz, 2H), 4.87 - 4.77 (m, 1H), 4.75 (d, *J* = 1.7 Hz, 2H), 3.18 (dp, *J* = 14.1, 4.3 Hz, 1H), 2.56 (d, *J* = 2.6 Hz, 1H), 1.84 - 1.46 (m, 5H), 1.18 (dtd, *J* = 25.4, 14.0, 6.4 Hz, 5H). ^13^C NMR (101 MHz, Chloroform-*d*) δ 158.00, 144.75, 139.58, 132.71, 128.54, 127.55, 127.22, 115.53, 78.38, 75.97, 55.97, 52.78, 34.05, 25.24, 24.75. ESI-MS (MeOH) m/z C_21_H_23_NO_3_S [M + H]^+^ calcd. for:370.12, found:370.20.

*N*-allyl-4'-(prop-2-yn-1-yloxy)-[1,1'-biphenyl]-4-sulfon-amide (**4c-14**). white solid, yield 17%. m.p.114.5~117.7℃. ^1^H NMR (400 MHz, Chloroform-*d*) δ 7.95 - 7.87 (m, 2H), 7.70 - 7.67 (m, 2H), 7.61 - 7.53 (m, 2H), 7.13 - 7.02 (m, 2H), 5.75 (ddt, *J* = 16.4, 11.0, 5.9 Hz, 1H), 5.23 - 5.07 (m, 2H), 4.76 (d, *J* = 2.4 Hz, 2H), 4.66 (t, *J* = 6.3 Hz, 1H), 3.64 (t, *J* = 6.1 Hz, 2H), 2.56 (d, *J* = 2.4 Hz, 1H). ^13^C NMR (101 MHz, Chloroform-*d*) δ 158.07, 145.19, 137.99, 133.05, 132.64, 128.59, 127.79, 127.36, 117.98, 115.57, 78.37, 76.00, 55.99, 45.96. ESI-MS (MeOH) m/z C_18_H_17_NO_3_S [M + H]^+^ calcd. for:328.10, found:328.15.

*N*-ethyl-4-(4-(prop-2-yn-1-yloxy)benzyl)benzenesulfona-mide (**6-1**) . white solid, yield 20%. m.p.46.8~47.2℃. ^1^H NMR (400 MHz, Chloroform-*d*) δ 7.77 (d, *J* = 8.4 Hz, 2H), 7.30 (d, *J* = 8.2 Hz, 2H), 7.10 (d, *J* = 8.6 Hz, 2H), 6.92 (d, *J* = 8.7 Hz, 2H), 4.67 (d, *J* = 2.4 Hz, 2H), 4.59 (t, *J* = 6.1 Hz, 1H), 3.98 (s, 2H), 2.99 (qd, *J* = 7.2, 6.0 Hz, 2H), 2.52 (t, *J* = 2.4 Hz, 1H), 1.09 (t, *J* = 7.2 Hz, 3H). ^13^C NMR (101 MHz, Chloroform-*d*) δ 156.28, 146.69, 137.61, 132.71, 130.00, 129.47, 127.32, 115.12, 78.58, 75.58, 55.86, 40.86, 38.26, 15.08. ESI-MS (MeOH) m/z C_18_H_19_NO_3_S [M + K]^+^ calcd. for:368.07, found:368.50.

4-(4-(prop-2-yn-1-yloxy)benzyl)-*N*-propylbenzenesulfo-namide (**6-2**). white solid, yield 21%. m.p.54.6~55.7℃. ^1^H NMR (400 MHz, Chloroform-*d*) δ 7.77 (d, *J* = 8.4 Hz, 2H), 7.31 (d, *J* = 8.3 Hz, 2H), 7.10 (d, *J* = 8.6 Hz, 2H), 6.93 (d, *J* = 8.6 Hz, 2H), 4.68 (d, *J* = 2.4 Hz, 2H), 4.39 (t, *J* = 6.2 Hz, 1H), 3.99 (s, 2H), 2.91 (q, *J* = 6.8 Hz, 2H), 2.52 (t, *J* = 2.4 Hz, 1H), 1.49 (h, *J* = 7.3 Hz, 2H), 0.87 (t, *J* = 7.4 Hz, 3H). ^13^C NMR (101 MHz, Chloroform-*d*) δ 156.28, 146.65, 137.70, 132.69, 129.99, 129.46, 127.29, 115.11, 78.57, 75.55, 55.86, 44.99, 40.87, 22.99, 11.11. ESI-MS (MeOH) m/z C_19_H_21_NO_3_S [M + H]^+^ calcd. for:344.13, found:344.13.

*N*-butyl-4-(4-(prop-2-yn-1-yloxy)benzyl)benzenesulfon-amide (**6-3**) . white solid, yield 23%. m.p.48.7~49.5℃. ^1^H NMR (400 MHz, Chloroform-*d*) δ 7.77 (d, *J* = 8.3 Hz, 2H), 7.30 (d, *J* = 8.0 Hz, 2H), 7.10 (d, *J* = 8.6 Hz, 2H), 6.92 (d, *J* = 8.6 Hz, 2H), 4.67 (d, *J* = 2.4 Hz, 2H), 4.51 (t, *J* = 6.2 Hz, 1H), 3.99 (s, 2H), 2.93 (q, *J* = 6.8 Hz, 2H), 2.51 (t, *J* = 2.4 Hz, 1H), 1.43 (m, 2H), 1.33 - 1.26 (m, 2H), 0.84 (t, *J* = 7.3 Hz, 3H).^13^C NMR (101 MHz, Chloroform-*d*) δ 156.29, 146.63, 137.69, 132.72, 129.98, 129.46, 127.29, 115.13, 75.54, 55.87, 42.95, 40.86, 31.62, 19.68, 13.54. ESI-MS (MeOH) m/z C_20_H_23_NO_3_S [M + H]^+^ calcd. for:358.12, found:358.19.

(E)-*N*-ethyl-4-(4-(prop-2-yn-1-yloxy)styryl)benzenesulf-onamide (**8-1**). white solid, yield 25%. m.p.89.6~93.7℃. ^1^H NMR (400 MHz, Chloroform-*d*) δ 7.71 (d, *J* = 8.4 Hz, 2H), 7.38 (d, *J* = 8.2 Hz, 2H), 7.15 (d, *J* = 8.6 Hz, 2H), 6.85 (d, *J* = 8.8 Hz, 2H), 6.67 (d, *J* = 12.2 Hz, 1H), 6.51 (d, *J* = 12.2 Hz, 1H), 4.68 (d, *J* = 2.4 Hz, 2H), 4.34 (t, *J* = 6.3 Hz, 1H), 3.03 (qd, *J* = 7.2, 6.1 Hz, 2H), 2.53 (t, *J* = 2.4 Hz, 1H), 1.11 (t, *J* = 7.2 Hz, 3H). ^13^C NMR (101 MHz, Chloroform-*d*) δ 157.96, 142.13, 138.03, 131.51, 130.23, 128.30, 127.70, 126.76, 125.21, 115.38, 78.42, 75.92, 55.99, 38.42, 15.28. ESI-MS (MeOH) m/z C_19_H_19_NO_3_S [M + H]^+^ calcd. for:342.12, found:342.10.

(E)-4-(4-(prop-2-yn-1-yloxy)styryl)-*N*-propylbenzenesul-fonamide (**8-2**). white solid, yield 22%. m.p.84.7~87.2℃. ^1^H NMR (400 MHz, Chloroform-*d*) δ 7.71 (d, *J* = 8.4 Hz, 2H), 7.38 (d, *J* = 8.5 Hz, 2H), 7.15 (d, *J* = 8.7 Hz, 2H), 6.84 (d, *J* = 8.7 Hz, 2H), 6.67 (d, *J* = 12.1 Hz, 1H), 6.51 (d, *J* = 12.2 Hz, 1H), 4.68 (d, *J* = 2.3 Hz, 2H), 4.39 (t, *J* = 6.2 Hz, 1H), 2.94 (q, *J* = 6.8 Hz, 2H), 2.53 (t, *J* = 2.4 Hz, 1H), 1.49 (h, *J* = 7.2 Hz, 2H), 0.87 (t, *J* = 7.4 Hz, 3H). ^13^C NMR (101 MHz, Chloroform-*d*) δ 157.95, 142.08, 138.09, 131.49, 130.24, 128.29, 127.67, 126.75, 125.23, 115.38, 78.42, 75.92, 55.99, 45.13, 23.12, 11.26. ESI-MS (MeOH) m/z C_20_H_21_NO_3_S [M + H]^+^ calcd. for:356.13, found:356.12.

(E)-*N*-butyl-4-(4-(prop-2-yn-1-yloxy)styryl)benzenesul-fonamide (**8-3**). white solid, yield 21%. m.p.97.4~99.6℃. ^1^H NMR (400 MHz, Chloroform-*d*) δ 7.71 (d, *J* = 8.0 Hz, 2H), 7.38 (d, *J* = 7.9 Hz, 2H), 7.15 (d, *J* = 8.2 Hz, 2H), 6.93 - 6.77 (m, 2H), 6.67 (d, *J* = 12.2 Hz, 1H), 6.51 (d, *J* = 12.2 Hz, 1H), 4.68 (d, *J* = 2.4 Hz, 2H), 4.37 (t, *J* = 6.3 Hz, 1H), 2.96 (q, *J* = 6.6 Hz, 2H), 2.53 (d, *J* = 2.9 Hz, 1H), 1.44 (p, *J* = 7.1 Hz, 2H), 1.31 (q, *J* = 7.2 Hz, 2H), 0.86 (t, *J* = 7.3 Hz, 3H).^13^C NMR (101 MHz, Chloroform-*d*) δ 157.95, 142.08, 138.02, 131.48, 130.24, 128.29, 127.67, 126.75, 125.23, 115.37, 78.42, 75.92, 55.99, 43.10, 31.76, 19.84, 13.68. ESI-MS (MeOH) m/z C_21_H_23_NO_3_S [M + H]^+^ calcd. for:370.12, found:370.15.

(E)-*N*-(sec-butyl)-4-(4-(prop-2-yn-1-yloxy)styryl)benze-nesulfonamide (**8-4**). white solid, yield 19%. m.p.90.6~93.2℃. ^1^H NMR (400 MHz, Chloroform-*d*) δ 7.73 (d, *J* = 8.4 Hz, 2H), 7.37 (d, *J* = 8.3 Hz, 2H), 7.13 (d, *J* = 8.6 Hz, 2H), 6.83 (d, *J* = 8.7 Hz, 2H), 6.66 (d, *J* = 12.2 Hz, 1H), 6.53 (d, *J* = 12.2 Hz, 1H), 4.68 (d, *J* = 2.4 Hz, 2H), 4.18 (d, *J* = 8.3 Hz, 1H), 3.27 (dq, *J* = 8.3, 6.5 Hz, 1H), 2.53 (t, *J* = 2.4 Hz, 1H), 1.41 - 1.34 (m, 2H), 1.04 (d, *J* = 6.5 Hz, 4H), 0.79 (d, *J* = 7.5 Hz, 3H). ^13^C NMR (101 MHz, Chloroform-*d*) δ 157.96, 142.07, 138.13, 131.49, 130.25, 128.29, 127.65, 126.76, 125.24, 115.38, 78.44, 75.93, 55.99, 51.69, 30.42, 21.28, 10.24. ESI-MS (MeOH) m/z C_21_H_23_NO_3_S [M + H]^+^ calcd. for:370.12, found:370.16.

*N*-ethyl-4-((4-(prop-2-yn-1-yloxy)phenyl)ethynyl)benze-nesulfonamide (**10-1**). white solid, yield 21%. m.p.59.7~62.5℃. ^1^H NMR (500 MHz, Chloroform-*d*) δ 7.88 (d, *J* = 8.4 Hz, 2H), 7.67 (d, *J* = 8.5 Hz, 2H), 7.54 (d, *J* = 8.8 Hz, 2H), 7.02 (d, *J* = 8.8 Hz, 2H), 4.77 (d, *J* = 2.5 Hz, 2H), 4.53 (t, *J* = 6.0 Hz, 1H), 3.08 (qd, *J* = 7.3, 6.0 Hz, 2H), 2.60 (t, *J* = 2.4 Hz, 1H), 1.16 (t, *J* = 7.3 Hz, 3H). ^13^C NMR (126 MHz, Chloroform-*d*) δ 158.07, 138.91, 133.33, 131.95, 128.29, 127.09, 115.43, 115.11, 92.79, 87.00, 78.03,76.01, 55.87, 38.33, 15.17. ESI-MS (MeOH) m/z C_19_H_17_NO_3_S [M + H]^+^ calcd. for:340.10, found:340.16.

4-((4-(prop-2-yn-1-yloxy)phenyl)ethynyl)-*N*-propylbenzenesulfonamide (**10-2**). white solid, yield 18%. m.p.66.3~67.6℃. ^1^H NMR (400 MHz, Chloroform-*d*) δ 7.84 (d, *J* = 8.6 Hz, 2H), 7.62 (d, *J* = 8.5 Hz, 2H), 7.50 (d, *J* = 8.8 Hz, 2H), 6.98 (d, *J* = 8.9 Hz, 2H), 4.73 (d, *J* = 2.5 Hz, 2H), 4.40 (d, *J* = 7.7 Hz, 1H), 3.56 - 3.44 (m, 1H), 2.55 (t, *J* = 2.4 Hz, 1H), 1.09 (d, *J* = 6.5 Hz, 6H). ^13^C NMR (101 MHz, Chloroform-*d*) δ 158.03, 140.01, 133.31, 131.92, 128.11, 126.98, 115.42, 115.08, 92.69, 87.01,78.03, 76.00, 55.85, 46.23, 23.82. ESI-MS (MeOH) m/z C_20_H_19_NO_3_S [M + H]^+^ calcd. for:354.12, found:354.12.

*N*-butyl-4-((4-(prop-2-yn-1-yloxy)phenyl)ethynyl)benz-enesulfonamide (**10-3**). white solid, yield 17%. m.p.70.8~72.4℃. ^1^H NMR (400 MHz, Chloroform-*d*) δ 7.82 (d, *J* = 8.0 Hz, 2H), 7.62 (d, *J* = 8.0 Hz, 2H), 7.50 (d, *J* = 8.4 Hz, 2H), 6.98 (d, *J* = 8.4 Hz, 2H), 4.73 (d, *J* = 1.8 Hz, 2H), 4.39 (t, *J* = 6.2 Hz, 1H), 2.97 (q, *J* = 6.7 Hz, 2H), 2.59 - 2.52 (m, 1H), 1.45 (p, *J* = 7.2 Hz, 2H), 1.31 (dt, *J* = 15.0, 7.2 Hz, 2H), 0.86 (t, *J* = 7.3 Hz, 4H). ^13^C NMR (101 MHz, Chloroform-*d*) δ 158.05, 138.86, 133.31, 131.92, 128.25, 127.05, 115.42, 115.09, 92.76, 86.99, 78.03,75.99, 55.85, 42.99, 31.64, 19.69, 13.53. ESI-MS (MeOH) m/z C_21_H_21_NO_3_S [M + H]^+^ calcd. for:368.13, found:368.47.

*N*-(sec-butyl)-4-((4-(prop-2-yn-1-yloxy)phenyl)ethynyl)-benzenesulfonamide (**10-4**). white solid, yield 20%. m.p.71.7~74.3℃. ^1^H NMR (400 MHz, Chloroform-*d*) δ 7.85 (d, *J* = 8.5 Hz, 2H), 7.61 (d, *J* = 8.5 Hz, 2H), 7.49 (d, *J* = 8.8 Hz, 2H), 6.97 (d, *J* = 8.8 Hz, 2H), 4.72 (d, *J* = 2.4 Hz, 2H), 4.55 (d, *J* = 8.2 Hz, 1H), 2.55 (t, *J* = 2.4 Hz, 1H), 1.40 (p, *J* = 7.3 Hz, 2H), 1.03 (d, *J* = 6.5 Hz, 3H), 0.90 - 0.84 (m, 1H), 0.80 (t, *J* = 7.4 Hz, 3H).^13^C NMR (101 MHz, Chloroform-*d*) δ 158.15, 140.30, 133.40, 131.98, 128.13, 127.08, 115.55, 115.20, 92.75, 87.17, 78.15, 76.10, 55.96, 51.68, 30.39, 21.36, 10.07. ESI-MS (MeOH) m/z C_21_H_21_NO_3_S [M + H]^+^ calcd. for:368.13, found:368.14.

*N*-cyclopropyl-6-(prop-2-yn-1-yloxy)-[1,1'-biphenyl]]-3-sulfonamide (**S4-1**). white solid, yield 38%. m.p. 68.5~69.1℃. ^1^H NMR (400 MHz, Chloroform-*d*) δ 8.10 (d, *J* = 2.4 Hz, 1H), 7.85 (dq, *J* = 7.9, 2.3 Hz, 1H), 7.49 - 7.18 (m, 6H), 5.09 (d, *J* = 2.8 Hz, 2H), 4.90 (d, *J* = 3.7 Hz, 1H), 2.26 (q, *J* = 4.9 Hz, 1H), 1.58 (d, *J* = 3.7 Hz, 1H), 0.62 (s, 4H). ^13^C NMR (101 MHz, Chloroform-*d*) δ 157.71, 133.33, 132.90, 131.99, 129.26, 128.54, 128.37, 121.78, 113.41, 112.85, 88.87, 82.20, 58.08, 24.45, 6.37. ESI-MS (MeOH) m/z C_18_H_17_NO_3_S [M + H]^+^ calcd. for:328.10, found:328.10.

*N*-butyl-6-(prop-2-yn-1-yloxy)-[1,1'-biphenyl]]-3-sulfona-mide (**S4-2**). white solid, yield 47%. m.p. 55.4~57.2℃. ^1^H NMR (400 MHz, Chloroform-*d*) δ 8.07 (d, *J* = 2.2 Hz, 1H), 7.81 (dd, *J* = 8.7, 2.3 Hz, 1H), 7.45 - 7.39 (m, 2H), 7.32 (qd, *J* = 8.9, 7.5, 3.3 Hz, 3H), 7.24 (d, *J* = 8.8 Hz, 1H), 5.08 (s, 2H), 4.37 (t, *J* = 6.2 Hz, 1H), 2.96 (q, *J* = 6.7 Hz, 2H), 1.46 (p, *J* = 7.1 Hz, 2H), 1.29 (dp, *J* = 14.5, 7.3 Hz, 3H), 0.86 (t, *J* = 7.3 Hz, 3H). ^13^C NMR (101 MHz, Chloroform-*d*) δ 157.53, 133.75, 132.56, 131.98, 129.25, 128.54, 127.98, 121.78, 113.46, 112.87, 88.82, 82.22, 58.04, 43.10, 31.73, 19.81, 13.66. ESI-MS (MeOH) m/z C_19_H_21_NO_3_S [M + H]^+^ calcd. for:344.13, found:344.14.

*N*-(sec-butyl)-6-(prop-2-yn-1-yloxy)-[1,1'-biphenyl]]-3-sulfonamide (**S4-3**). white solid, yield 45%. m.p.73.5~76.1℃. ^1^H NMR (400 MHz, CDCl3) δ 8.02 (d, J = 2.3 Hz, 1H), 7.76 (dd, J = 8.7, 2.3 Hz, 1H), 7.37 - 7.31 (m, 1H), 7.30 - 7.19 (m, 1H), 7.15 (d, J = 8.7 Hz, 1H), 5.00 (s, 1H), 4.54 (d, J = 8.1 Hz, 1H), 3.29 - 3.07 (m, 1H), 1.43 - 1.26 (m, 1H), 0.97 (d, J = 6.6 Hz, 1H), 0.73 (t, J = 7.4 Hz, 1H).^13^C NMR (101 MHz, Chloroform-*d*) δ 157.35, 135.04, 132.47, 131.93, 129.19, 128.50, 127.81, 121.77, 113.46, 112.72, 88.78, 82.23, 58.04, 51.64, 30.35, 21.33, 10.07. ESI-MS (MeOH) m/z C_19_H_21_NO_3_S [M + H]^+^ calcd. for:344.13, found:344.15.

3-benzyl-*N*-cyclopropyl-4-(prop-2-yn-1-yloxy)benzene-sulfonamide (**S6-1**). white solid, yield 55%. m.p. 65.1~68.0℃. ^1^H NMR (400 MHz, Chloroform-*d*) δ 7.69 (dd, *J* = 8.6, 2.4 Hz, 1H), 7.56 (d, *J* = 2.4 Hz, 1H), 7.23 - 7.15 (m, 2H), 7.13 (d, *J* = 7.2 Hz, 3H), 6.99 (d, *J* = 8.6 Hz, 1H), 4.79 (s, 1H), 4.69 (d, *J* = 2.4 Hz, 2H), 3.94 (s, 2H), 2.48 (t, *J* = 2.4 Hz, 1H), 2.10 (tt, *J* = 6.8, 3.6 Hz, 1H), 0.45 (ddt, *J* = 11.8, 5.0, 2.3 Hz, 5H). ^13^C NMR (101 MHz, Chloroform-*d*) δ 158.59, 139.48, 131.89, 131.40, 129.77, 128.95, 128.50, 127.47, 126.31, 111.66, 77.64, 76.34, 56.21, 35.82, 24.32, 6.12. ESI-MS (MeOH) m/z C_19_H_19_NO_3_S [M + H]^+^ calcd. for:342.12, found:342.17.

3-benzyl-*N*-butyl-4-(prop-2-yn-1-yloxy)benzenesulfona-mide **(S6-2**). white solid, yield 49%. m.p. 72.6~72.9℃. ^1^H NMR (400 MHz, Chloroform-*d*) δ 7.66 (dd, *J* = 8.6, 2.4 Hz, 1H), 7.53 (d, *J* = 2.4 Hz, 1H), 7.22 - 7.16 (m, 2H), 7.12 (d, *J* = 7.2 Hz, 3H), 6.97 (d, *J* = 8.6 Hz, 1H), 4.68 (d, *J* = 2.4 Hz, 2H), 4.41 (t, *J* = 6.3 Hz, 1H), 3.92 (s, 2H), 2.80 (q, *J* = 6.2 Hz, 2H), 2.47 (t, *J* = 2.4 Hz, 1H), 1.31 (dtd, *J* = 8.4, 7.6, 7.1, 6.1 Hz, 2H), 1.23 - 1.12 (m, 2H), 0.75 (t, *J* = 7.3 Hz, 3H). ^13^C NMR (101 MHz, CDCl3) δ 158.59 (s), 139.62 (s), 132.45 (s), 131.71 (s), 129.54 (s), 129.08 (s), 128.65 (s), 127.34 (s), 126.46 (s), 112.87 - 111.99 (m), 77.82 (s), 76.45 (s), 56.34 (s), 43.06 (s), 36.03 (s), 31.68 (s), 19.85 (s), 13.70 (s).ESI-MS (MeOH) m/z C_20_H_23_NO_3_S [M + H]^+^ calcd. for:358.12, found:358.20.

3-benzyl-*N*-(sec-butyl)-4-(prop-2-yn-1-yloxy)benzenesu-lfonamide (**S6-3**). white solid, yield 45%. m.p. 81.5~82.0℃. ^1^H NMR (400 MHz, Chloroform-*d*) δ 7.77 (dd, *J* = 8.6, 2.4 Hz, 1H), 7.63 (d, *J* = 2.5 Hz, 1H), 7.27 (d, *J* = 7.2 Hz, 2H), 7.24 - 7.17 (m, 3H), 7.06 (d, *J* = 8.7 Hz, 1H), 4.78 (d, *J* = 2.2 Hz, 2H), 4.46 (s, 1H), 4.02 (s, 2H), 3.19 (p, *J* = 6.7 Hz, 1H), 2.57 (t, *J* = 2.4 Hz, 1H), 1.42 - 1.31 (m, 2H), 1.00 (d, *J* = 6.6 Hz, 3H), 0.77 (t, *J* = 7.4 Hz, 3H). ^13^C NMR (101 MHz, CDCl3) δ 158.32 (s), 139.52 (s), 133.51 (s), 131.40 (s), 129.30 (s), 128.93 (s), 128.48 (s), 127.13 (s), 126.28 (s), 111.58 (s), 77.68 (s), 76.28 (s), 56.21 (s), 51.32 (s), 35.83 (s), 30.22 (s), 21.18 (s), 9.95 (s).ESI-MS (MeOH) m/z C_20_H_23_NO_3_S [M + H]^+^ calcd. for:358.12, found:358.20.

*N*-allyl-3-benzyl-4-(prop-2-yn-1-yloxy)benzenesulfona-mide (**S6-4**). white solid, yield 36%. m.p. 105.2~108..4℃. ^1^H NMR (400 MHz, CDCl3) δ 7.73 (dd, J = 8.6, 2.2 Hz, 1H), 7.60 (d, J = 2.1 Hz, 1H), 7.31 - 7.15 (m, 5H), 7.05 (d, J = 8.7 Hz, 1H), 5.67 (dq, J = 10.6, 5.8 Hz, 1H), 5.08 (dd, J = 24.0, 13.7 Hz, 2H), 4.76 (d, J = 2.2 Hz, 2H), 4.52 (t, J = 6.2 Hz, 1H), 4.00 (s, 2H), 3.53 (t, J = 6.0 Hz, 2H), 2.55 (t, J = 2.2 Hz, 1H).^13^C NMR (101 MHz, CDCl3) δ 158.55 (s), 139.42 (s), 133.00 (s), 132.20 (s), 131.52 (s), 129.40 (s), 128.95 (s), 128.52 (s), 127.28 (s), 126.34 - 125.64 (m), 117.67 - 117.24 (m), 111.71 (s), 77.64 (s), 76.36 (s), 56.19 (s), 45.77 (s), 35.88 (s).ESI-MS (MeOH) m/z C_19_H_19_NO_3_S [M + H]^+^ calcd. for:342.12, found:342.17.

(E)-*N*-cyclopropyl-4-(prop-2-yn-1-yloxy)-3-styrylbenze-nesulfonamide（E:Z=1.47:1）(**S8-1**). white solid, yield 23%. m.p. 92.4~93.1℃. ^1^H NMR (400 MHz, Chloroform-*d*) δ 8.25 (d, *J* = 2.3 Hz, 1H), 7.89 (ddd, *J* = 8.7, 6.2, 2.4 Hz, 2H), 7.70 (d, *J* = 2.4 Hz, 1H), 7.68 - 7.62 (m, 1H), 7.49 (dd, *J* = 13.6, 6.1 Hz, 2H), 7.44 - 7.36 (m, 1H), 7.28 (d, *J* = 5.6 Hz, 1H), 7.25 - 7.19 (m, 3H), 6.88 (d, *J* = 12.1 Hz, 1H), 6.76 (d, *J* = 12.0 Hz, 1H), 5.04 (s, 1H), 4.96 (d, *J* = 2.4 Hz, 1H), 4.90 (d, *J* = 2.4 Hz, 2H), 4.68 (s, 1H), 2.70 (d, *J* = 2.4 Hz, 1H), 2.68 (t, *J* = 2.6 Hz, 1H), 2.39 (tt, *J* = 6.7, 3.6 Hz, 1H), 1.96 (tt, *J* = 6.8, 3.4 Hz, 1H), 0.80 - 0.67 (m, 3H), 0.63 (td, *J* = 4.6, 4.1 Hz, 2H), 0.50 (td, *J* = 7.1, 4.9 Hz, 2H). ^13^C NMR (101 MHz, Chloroform-*d*) δ 158.63, 157.80, 137.16, 136.83, 132.56, 132.50, 131.84, 131.69, 130.14, 129.04, 128.87, 128.36, 128.25, 128.06, 127.97, 127.92, 127.33, 126.97, 125.87, 124.58, 121.46, 112.47, 112.18, 77.72, 77.66, 76.75, 76.64, 56.56, 56.47, 24.48, 24.33, 6.34, 6.23. ESI-MS (MeOH) m/z C_20_H_19_NO_3_S [M + H]^+^ calcd. for:354.12, found:354.18.

(E)-*N*-butyl-4-(prop-2-yn-1-yloxy)-3-styrylbenzenesulfo-namide（E:Z=1.8:1）（**S8-2**）. white solid, yield 26%. m.p. 82.6~83.9℃. ^1^H NMR (400 MHz, Chloroform-*d*) δ 7.83 (td, *J* = 8.7, 2.4 Hz, 1H), 7.71 - 7.60 (m, 2H), 7.47 (dd, *J* = 13.1, 5.8 Hz, 1H), 7.38 (d, *J* = 7.3 Hz, 1H), 7.32 - 7.26 (m, 3H), 7.24 (t, *J* = 3.8 Hz, 1H), 7.19 (dd, *J* = 8.7, 6.1 Hz, 1H), 6.85 (d, *J* = 12.1 Hz, 1H), 6.73 (d, *J* = 12.1 Hz, 1H), 4.93 (d, *J* = 2.4 Hz, 1H), 4.85 (d, *J* = 2.4 Hz, 2H), 4.69 (t, *J* = 6.2 Hz, 1H), 4.26 (t, *J* = 6.3 Hz, 1H), 3.06 (q, *J* = 6.8 Hz, 1H), 2.76 (q, *J* = 6.7 Hz, 2H), 2.66 (t, *J* = 2.4 Hz, 1H), 1.71 (s, 1H), 1.62 - 1.26 (m, 8H), 0.95 (t, *J* = 7.2 Hz, 5H). ^13^C NMR (101 MHz, Chloroform-*d*) δ 158.38, 157.63, 137.17, 136.79, 132.86, 132.52, 132.25, 131.64, 129.68, 128.96, 128.85, 128.39, 128.23, 128.07, 127.90, 127.83, 127.54, 127.49, 126.96, 125.53, 124.33, 121.45, 112.51, 112.29, 77.74, 77.69, 76.70, 76.59, 56.53, 56.45, 43.09, 42.88, 31.71, 19.84, 19.80, 13.73, 13.69. ESI-MS (MeOH) m/z C_21_H_23_NO_3_S [M + H]^+^ calcd. for:370.15, found:370.22.

(E)-*N*-(sec-butyl)-4-(prop-2-yn-1-yloxy)-3-styrylbenzen-esulfonamide（E:Z=1.2:1）（**S8-3**）. white solid, yield 30%. m.p. 96.1~99.4℃. ^1^H NMR (400 MHz, Chloroform-*d*) δ 8.23 (d, *J* = 2.3 Hz, 1H), 7.84 (ddd, *J* = 11.7, 8.7, 2.4 Hz, 2H), 7.73 (d, *J* = 2.4 Hz, 1H), 7.66 - 7.58 (m, 2H), 7.46 (dd, *J* = 13.5, 6.0 Hz, 2H), 7.41 - 7.32 (m, 1H), 7.27 (s, 1H), 7.25 (s, 1H), 7.18 (d, *J* = 3.8 Hz, 1H), 7.16 (d, *J* = 3.7 Hz, 1H), 6.83 (d, *J* = 12.2 Hz, 1H), 6.71 (d, *J* = 12.2 Hz, 1H), 4.92 (d, *J* = 2.4 Hz, 2H), 4.83 (d, *J* = 2.5 Hz, 2H), 4.71 (d, *J* = 8.0 Hz, 1H), 4.29 (d, *J* = 7.9 Hz, 1H), 3.35 (dq, *J* = 8.0, 6.4 Hz, 1H), 3.17 - 3.03 (m, 1H), 2.67 (t, *J* = 2.4 Hz, 1H), 2.64 (t, *J* = 2.4 Hz, 1H), 1.50 (q, *J* = 7.1 Hz, 2H), 1.41 (p, *J* = 7.2 Hz, 2H), 1.14 (d, *J* = 6.6 Hz, 3H), 1.01 (d, *J* = 6.5 Hz, 3H), 0.91 (t, *J* = 7.4 Hz, 3H), 0.85 (t, *J* = 7.4 Hz, 3H). ^13^C NMR (101 MHz, Chloroform-*d*) δ 158.24, 157.51, 137.19, 136.76, 134.17, 133.59, 132.48, 131.53, 129.59, 128.85, 128.83, 128.41, 128.19, 127.95, 127.80, 127.70, 127.51, 127.44, 126.94, 125.46, 124.21, 121.50, 112.47, 112.36, 77.74, 77.69, 76.66, 76.53, 56.54, 56.46, 51.53, 51.22, 30.39, 30.32, 21.34, 21.23, 10.09, 10.01. ESI-MS (MeOH) m/z C_21_H_23_NO_3_S [M + H]^+^ calcd. for:370.15, found:370.21.

(E)-*N*-allyl-4-(prop-2-yn-1-yloxy)-3-styrylbenzenesulfo-namide（E:Z=1.6:1）（**S8-4**）. white solid, yield 22%. m.p. 126.1~130.7℃. ^1^H NMR (400 MHz, Chloroform-*d*) δ 8.23 (d, *J* = 2.3 Hz, 1H), 7.83 (ddd, *J* = 10.9, 8.7, 2.4 Hz, 2H), 7.67 (d, *J* = 2.4 Hz, 1H), 7.63 (d, *J* = 7.3 Hz, 1H), 7.47 (dd, *J* = 13.3, 5.8 Hz, 2H), 7.42 - 7.34 (m, 1H), 7.28 (d, *J* = 14.1 Hz, 2H), 7.25 (d, *J* = 3.2 Hz, 1H), 7.19 (dd, *J* = 8.7, 3.5 Hz, 2H), 6.85 (d, *J* = 12.1 Hz, 1H), 6.73 (d, *J* = 12.1 Hz, 1H), 5.84 (ddt, *J* = 16.2, 10.2, 5.8 Hz, 1H), 5.74 (ddt, *J* = 16.2, 10.3, 5.7 Hz, 1H), 4.97 (t, *J* = 6.3 Hz, 1H), 4.93 (d, *J* = 2.4 Hz, 1H), 4.85 (d, *J* = 2.4 Hz, 2H), 4.49 (t, *J* = 6.3 Hz, 1H), 3.70 (tt, *J* = 6.2, 1.7 Hz, 1H), 3.38 (tt, *J* = 6.2, 1.7 Hz, 2H), 2.68 (t, *J* = 2.4 Hz, 1H), 2.67 (t, *J* = 2.4 Hz, 1H).^13^C NMR (101 MHz, Chloroform-*d*) δ 158.47, 157.68, 137.11, 136.73, 133.08, 132.75, 132.55, 132.03, 131.64, 129.74, 128.91, 128.81, 128.41, 128.20, 128.06, 127.88, 127.78, 127.58, 127.51, 126.93, 125.53, 124.25, 121.35, 117.83, 117.59, 112.51, 112.35, 77.69, 77.65, 76.72, 76.62, 56.50, 56.43, 45.89, 45.68. ESI-MS (MeOH) m/z C_20_H_19_NO_3_S [M + H]^+^ calcd. for:354.12, found:354.18.

*N*-cyclopropyl-3-(phenylethynyl)-4-(prop-2-yn-1-yloxy)-benzenesulfonamide（**S10-1**）. white solid, yield 19%. m.p. 53.1~52.6℃. ^1^H NMR (400 MHz, Chloroform-*d*) δ 8.05 (d, *J* = 2.4 Hz, 1H), 7.84 (dd, *J* = 8.8, 2.4 Hz, 1H), 7.56 (dq, *J* = 7.1, 2.6, 2.2 Hz, 2H), 7.40 - 7.33 (m, 3H), 7.16 (d, *J* = 8.8 Hz, 1H), 4.96 (s, 1H), 4.88 (d, *J* = 2.4 Hz, 2H), 2.60 (t, *J* = 2.4 Hz, 1H), 2.28 (dddd, *J* = 7.1, 5.7, 4.4, 2.2 Hz, 1H), 0.62 (dt, *J* = 7.3, 1.9 Hz, 4H). ^13^C NMR (101 MHz, Chloroform-*d*) δ 160.92, 133.01, 132.42, 131.88, 129.01, 128.88, 128.51, 122.84, 114.46, 112.57, 95.61, 83.80, 77.40, 77.04, 56.86, 24.43, 6.33. ESI-MS (MeOH) m/z C_20_H_17_NO_3_S [M + H]^+^ calcd. for:352.10, found:352.19.

*N*-butyl-3-(phenylethynyl)-4-(prop-2-yn-1-yloxy)benzen-eesulfonamide (**S10-2**). white solid, yield 20%. m.p.47.6~49.2℃. ^1^H NMR (400 MHz, Chloroform-*d*) δ 8.01 (d, *J* = 2.4 Hz, 1H), 7.81 (dd, *J* = 8.8, 2.3 Hz, 1H), 7.56 (dq, *J* = 7.9, 3.1, 2.4 Hz, 2H), 7.37 (q, *J* = 2.9 Hz, 3H), 7.15 (d, *J* = 8.8 Hz, 1H), 4.87 (d, *J* = 2.4 Hz, 2H), 4.56 (t, *J* = 6.2 Hz, 1H), 2.96 (q, *J* = 6.8 Hz, 2H), 2.59 (t, *J* = 2.4 Hz, 1H), 1.46 (dq, *J* = 8.6, 7.1 Hz, 2H), 1.37 - 1.25 (m, 2H), 0.87 (t, *J* = 7.3 Hz, 3H). ^13^C NMR (101 MHz, Chloroform-*d*) δ 160.76, 132.83, 132.70, 131.88, 128.87, 128.63, 128.51, 122.86, 114.47, 112.65, 95.58, 83.83, 77.44, 76.99, 56.85, 43.09, 31.72, 19.83, 13.68.ESI-MS (MeOH) m/z C_21_H_21_NO_3_S [M + H]^+^ calcd. for:368.13, found:368.22.

*N*-(sec-butyl)-3-(phenylethynyl)-4-(prop-2-yn-1-yloxy)-benzenesulfonamide (**S10-3**). white solid, yield 25%. m.p.61.5~62.7℃. ^1^H NMR (400 MHz, Chloroform-*d*) δ 8.05 (d, *J* = 2.4 Hz, 1H), 7.83 (dd, *J* = 8.8, 2.4 Hz, 1H), 7.61 - 7.50 (m, 2H), 7.35 (p, *J* = 3.9, 3.4 Hz, 3H), 7.13 (d, *J* = 8.8 Hz, 1H), 4.87 - 4.80 (m, 3H), 3.26 (dq, *J* = 8.0, 6.4 Hz, 1H), 2.59 (t, *J* = 2.4 Hz, 1H), 1.42 (p, *J* = 7.2 Hz, 2H), 1.05 (d, *J* = 6.6 Hz, 3H), 0.82 (t, *J* = 7.4 Hz, 3H). ^13^C NMR (101 MHz, Chloroform-*d*) δ 160.59, 134.11, 132.54, 131.82, 128.80, 128.49, 128.46, 122.83, 114.26, 112.59, 95.42, 83.90, 77.40, 76.94, 56.81, 51.55, 30.30, 21.24, 10.06. ESI-MS (MeOH) m/z C_21_H_21_NO_3_S [M + H]^+^ calcd. for:368.13, found:368.21.

*N*-allyl-3-(phenylethynyl)-4-(prop-2-yn-1-yloxy) benzene-sulfonamide (**S10-4**). white solid, yield 20%. m.p.74.6~77.4℃. ^1^H NMR (400 MHz, Chloroform-*d*) δ 8.02 (d, *J* = 2.3 Hz, 1H), 7.81 (dd, *J* = 8.9, 2.4 Hz, 1H), 7.55 (dd, *J* = 6.6, 3.0 Hz, 2H), 7.41 - 7.30 (m, 3H), 7.15 (d, *J* = 8.8 Hz, 1H), 5.74 (ddt, *J* = 16.3, 10.9, 5.8 Hz, 1H), 5.25 - 5.08 (m, 2H), 4.87 (d, *J* = 2.4 Hz, 2H), 4.78 (t, *J* = 6.3 Hz, 1H), 3.61 (t, *J* = 6.1 Hz, 2H), 2.64 - 2.56 (m, 1H). ^13^C NMR (101 MHz, Chloroform-*d*) δ 160.86, 132.99, 132.75, 132.73, 131.88, 128.89, 128.70, 128.52, 122.82, 118.02, 114.53, 112.66, 95.64, 83.77, 77.40, 77.04, 56.85, 45.93. ESI-MS (MeOH) m/z C_20_H_17_NO_3_S [M + H]^+^ calcd. for:352.10, found:352.15.

### 4.1 Insecticidal Activity

Leaf squares were immersed in the test solutions for 3 s, air-dried, and placed in 12-well plates. One starved third-instar *M. separata* larva was added to each well, with three replicates per compound. Mortality was recorded after 48 h at 25°C. Six concentration gradients were tested to maintain mortality between 20%-80%, and LC₅₀ values were calculated.

### 4.2 Electrophysiological Recordings

Dissected larvae were immobilized, and microelectrodes were inserted into the abdominal longitudinal muscles to monitor the resting membrane potential (-80 to -50 mV). Excitatory junctional potentials (EJPs) were evoked by 0.1 Hz nerve stimulation. Test compounds and the BA1 stock were diluted to final concentrations of 20 μM and 2 μM, respectively, with DMSO used as the control. EJP recordings were initiated after stabilization of the resting membrane potential.

### 4.3 Molecular Docking

To determine whether the target compounds interact with the H subunit of the V-ATPase receptor in *M. separata* and to elucidate their mode of action, representative samples (S6-3 and S6-4) and a control sample (L-1) were docked with a homology model of the H subunit [21]. The ligand structures were generated using Discovery Studio, and their energies were minimized before docking with the *M. separata* V-ATPase H subunit model previously constructed in the laboratory. Docking results were analyzed using Discovery Studio 2019 Client [22].

## 5 RESULTS AND DISCUSSION

### 5.1 Synthesis

Figs. (**2**, **3**) summarize the synthetic routes for the target compounds. Initially, the corresponding diphenyl sulfonamide intermediates 2 were synthesized by reacting bromosulfonyl chloride with various substituted amines. Biphenyl and diphenylmethane backbones were then introduced into intermediates 2 *via* Suzuki coupling reactions, yielding intermediates 3 and 5, respectively.

Additionally, intermediates 2b reacted with triphenylphosphine to form a triphenylphosphine salt, which subsequently underwent a Wittig reaction with 4-alkynyl propoxy benzaldehyde to produce diphenylethene derivatives (compounds 8). Likewise, intermediates 2a underwent a Sonogashira reaction with 4-hydroxyphenylacetylene, introducing the diphenylacetylene backbone and generating intermediates 9.

Finally, intermediates 3, 5, and 9 reacted with 3-bromopropyne to incorporate alkynyl propoxy groups, affording the target compounds 4, 6, and 10.

The biological activity of compounds 4, 6, 8, and 10 was evaluated, after which structural optimization was performed to generate the target compounds (S4, S6, S8, and S10). These optimized molecules featured alkynyl propoxy and sulfonamide groups located on the same benzene ring within their respective backbones.

The synthesis of S4 began with 2-bromophenol as the starting material. Propargylation and chlorosulfonation reactions produced the intermediate sulfonyl chloride (intermediate 13). Subsequent substitution reactions between this intermediate and various small-molecule amines, followed by a Suzuki coupling reaction, ultimately afforded the target compounds S4.

For the synthesis of S6, 2-benzylphenol served as the starting material. Its reaction with 3-bromopropyne introduced an alkynyl propoxy group, forming intermediate 16. Sulfonation with chlorosulfonic acid and subsequent chlorination with phosphorus pentachloride yielded the benzenesulfonyl chloride intermediate 18. Final reactions with different amines completed the synthesis of S6, which possessed a diphenylmethane backbone.

To synthesize S8, 2-hydroxybenzaldehyde was used as the starting material. Propargyl and sulfonyl chloride groups were first introduced to generate intermediate 21, which was further modified by substituting its dichloromethyl group with various amines. Reaction with sodium methoxide then produced intermediates 23 containing an aldehyde group. A subsequent Wittig reaction with a triphenylphosphine salt introduced the diphenylethene backbone, ultimately yielding S8.

For S10, the starting material was 2-iodophenol. Methyl and sulfonyl chloride groups were introduced, followed by substitution reactions with different amines to obtain intermediates 27. A Sonogashira reaction with phenylacetylene then introduced the diphenylacetylene backbone, after which boron tribromide was used to remove protective groups and generate intermediates 29. Finally, an alkynyl propoxy group was introduced, yielding the target compounds S10, which featured a diphenylacetylene backbone with the active groups positioned on the same side of the benzene ring.


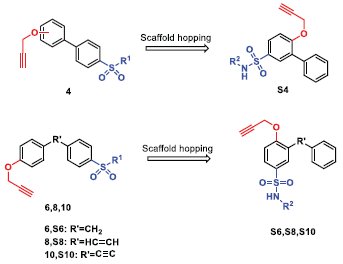


### 5.2 Insecticidal Activity

To evaluate the potential of the synthesized compounds as new pesticides targeting *M. separata*, their insecticidal activity was assessed. Specifically, the effects of the target compounds on symptom development and mortality in third-instar *M. separata* larvae within 48 h were recorded using the leaf-dipping method. Celangulin V, lead compound L-1, and indoxacarb were used as positive controls, while acetone served as the blank control.

First, the biphenyl compounds (compounds 4) were evaluated. As shown in Table **1**, several synthesized compounds exhibited good insecticidal activity against third-instar larvae at a concentration of 10 mg/mL. Notably, compounds 4b-7, 4c-1-7, 4c-12, and 4c-14 showed mortality rates exceeding 60%, while compounds 4c-7 and 4c-14 maintained mortality rates above 50% even at the reduced concentration of 1 mg/mL. The results further indicated that insecticidal activity was influenced by the positional relationship between the phenyl group and the alkynyl propoxy group. Specifically, compounds with substituents in the para position (*e.g.,* compound 4c) displayed higher activity than those with meta-position substituents and significantly higher activity than those with ortho-position substituents. Additionally, compounds containing amino groups with 2-4 carbon atoms and monosubstitution exhibited better insecticidal activity.

Compounds 6, 8, and 10 were synthesized by aligning the position of the phenyl group with that of the alkynyl propoxy group, and their bioassay results are summarized in Table **2**. In compounds 6, the two benzene rings were connected by a methylene group, and their activity showed little variation compared with compounds 4c, with mortality rates exceeding 90% at a concentration of 10 mg/mL. However, in compounds 8 and 10, the phenyl rings were connected by ethylene and ethynyl groups, respectively. When a double bond was introduced (compounds 8), insecticidal activity decreased, and only compound 8-4 exhibited a mortality rate above 60% at 10 mg/mL. Similarly, introducing an alkyne linkage produced compounds 10, which displayed even lower activity than compounds 8; in this series, only compound 10-4 achieved a mortality rate above 60% at 10 mg/mL. Moreover, compounds 6, 8, and 10 generally showed no detectable insecticidal activity at 1 mg/mL. Overall, insecticidal potency declined as the spatial distance between the alkynyl propoxy and sulfonamide groups increased.

Based on these findings, compounds S4, S6, S8, and S10-comprising 15 newly designed molecules-were synthesized by positioning the active groups (alkynyl propoxy and sulfonamide) on the same benzene ring. Their insecticidal activities were evaluated, and the results are presented in Table **3**. When both functional groups were located on the same benzene ring, several compounds exhibited enhanced insecticidal activity. Moreover, at a concentration of 1 mg/mL, structural modifications resulted in marked improvements in potency, with compounds S6-2, S8-3, and S10-3 showing superior activity compared with compounds 6-3, 8-4, and 10-4, respectively.

Among the tested compounds, those exhibiting a mortality rate above 50% at a concentration of 1 mg/mL were selected for LC₅₀ determination. As shown in Table **4**, the LC₅₀ values of compounds 4c-14, S6-3, S6-4, S8-3, and S10-1 were approximately three times lower (indicating higher potency) than that of the lead compound L-1, while the LC₅₀ value of compound 4c-7 was slightly higher than that of L-1. Nevertheless, the insecticidal activities of all these compounds were superior to that of celangulin V.

During the insecticidal activity tests, a series of poisoning symptoms were observed in the treated insects (Fig. **4**). Shortly after ingesting small amounts of treated leaves, the insects became unconscious and immobilized. This was followed by severe writhing and convulsions, accompanied by the exudation of bodily fluids from the posterior end, ultimately resulting in death. These symptoms closely resembled those induced by celangulin V and the lead compound, suggesting that the tested compounds may share a similar mode of action with these two controls.

### 5.3 Structure-activity Relationship

The structure-activity relationship of the compounds was analyzed based on the experimental results, leading to the following conclusions: (1) In biphenyl compounds, when the alkynyl propoxy and phenyl groups are positioned para to each other, insecticidal activity is higher than when the groups are in the meta position and significantly higher than when they are in the ortho position. (2) As the number of carbon atoms linking the benzene ring backbone increases, the spatial distance between the alkynyl propoxy and sulfonamide groups also increases, resulting in a decline in insecticidal activity [23]. (3) Compounds in which the alkynyl propoxy and sulfonamide groups occupy the para position on the same benzene ring are more likely to exhibit strong insecticidal activity. (4) Target compounds containing sulfonamide groups with 3-4 carbon atoms and mono-substitution tend to demonstrate higher insecticidal activity.

### 5.4 Electrophysiological Recordings

To determine whether the target compounds interacted with V-ATPase, bafilomycin A1 (BA1), a specific V-ATPase inhibitor, was used as a reference. Intracellular microelectrode technology was employed to measure the effects of both the target compounds and BA1 on the intensity of neuronal transmission signals [24]. As shown in Fig. (**5**), BA1 significantly reduced neuronal action potentials, confirming its inhibitory effect on interneuronal signaling. Similarly, the target compounds decreased the amplitude of neuronal action potentials. This reduction suggests that the target compounds may share a similar mode of action with BA1.

Electrophysiological tests further demonstrated that the amplitude of excitatory junction potentials (EJPs) in the control group of *Drosophila melanogaster* remained stable throughout the recording period (Fig. **6**). Neuromuscular junction specimens from dissected third-instar larvae were stimulated at 0.1 Hz for 60 min. In the control group, the EJP amplitude showed minimal decline, with reduction rates of 0%, 1%, 3% and 6% at 10, 20, 40, and 60 min, respectively. In contrast, specimens treated with BA1 exhibited significantly greater decreases in EJP amplitude, with reduction rates of 13%, 23%, 38% and 62% at the same time points.

Similarly, specimens treated with compound S6-4 showed a progressive decline in EJP amplitude of 10%, 19%, 23% and 35% at 10, 20, 40, and 60 min, respectively. Although the rate of decline induced by S6-4 was slower than that observed for BA1, the overall trend was comparable. These findings indicate that, similar to BA1, compound S6-4 reduces neuronal excitability and affects synaptic transmission. The comparable effects observed at the neuromuscular junction of Drosophila suggest that S6-4 may target the V-ATPase located on the presynaptic membrane at neuromuscular junctions.

### 5.5 Molecular Docking

The binding sites within the structural domains of the receptor were examined (Fig. **7**). The results indicated that compound S6-3 was fully accommodated within the binding pocket of the H subunit of V-ATPase in *M. separata*. Compounds S6-3, S6-4 and L-1 displayed similar binding patterns, interacting with the same amino acid residues: ALA278, GLU274, LYS330, LYS232 and CYS282. However, compared with the lead compound L-1, S6-3 and S6-4 formed additional interactions with three more amino acid residues, which may account for their higher insecticidal activity relative to L-1.

When these molecular docking results were considered alongside the observed poisoning symptoms in the test insects, it could be inferred that the target compounds likely exert their insecticidal effects by acting on the H subunit of V-ATPase.

## STUDY LIMITATIONS

This study has several limitations that warrant discussion. First, although the compounds were evaluated in *M. separata* and *Drosophila melanogaster*, their efficacy against other economically important pests and their potential toxicity to non-target organisms (*e.g.,* beneficial insects or mammals) remain uncharacterized. Second, while molecular docking and electrophysiological data support V-ATPase inhibition, the metabolic stability and pharmacokinetic properties of the compounds *in vivo* are not yet fully understood. Additionally, although structural optimization focused on biphenyl backbones, the exploration of heteroaromatic scaffolds or alternative linkers (*e.g.,* ether or thioether groups) was limited, potentially restricting the identification of more potent analogs. Finally, environmental fate studies, such as degradation kinetics in soil or water, are needed to assess the ecological safety of these derivatives for practical agricultural use. These aspects represent important directions for future research.

## CONCLUSION

A series of diphenyl sulfonamide derivatives containing alkynyl propoxy and sulfonamide groups, both associated with insecticidal activity, were designed, synthesized, and optimized in two stages, yielding a total of 55 compounds. Their insecticidal activity against *M. separata* was evaluated, with compounds S6-3 and S6-4 exhibiting LC₅₀ values of 0.474 mg/mL and 0.401 mg/mL, respectively, indicating strong insecticidal potency. Electrophysiological studies further revealed that S6-4 weakened excitatory signals in a manner similar to BA1, suggesting a comparable inhibitory effect on V-ATPase. Molecular docking demonstrated that S6-3 and S6-4, along with the lead compound L-1, were able to effectively bind within the activity pocket of the H subunit of V-ATPase.

Overall, the combined findings strongly indicate that the H subunit of V-ATPase is the likely target site for these compounds. This work also demonstrates that diphenyl sulfonamide derivatives represent promising candidates for the development of novel pesticides with potential commercial applications.

## AUTHORS' CONTRIBUTIONS

The authors confirm their contribution as follows: TG and MT contributed equally to this work. The study was conceived and designed by WW, ZH, and JZ. Data were collected by JZ, YL, ZW, ZS, JZ, and RL. TG performed the analysis and interpretation of the results. MT drafted the manuscript. All authors reviewed the results and approved the final version of the manuscript.

## Figures and Tables

**Fig. (1):**
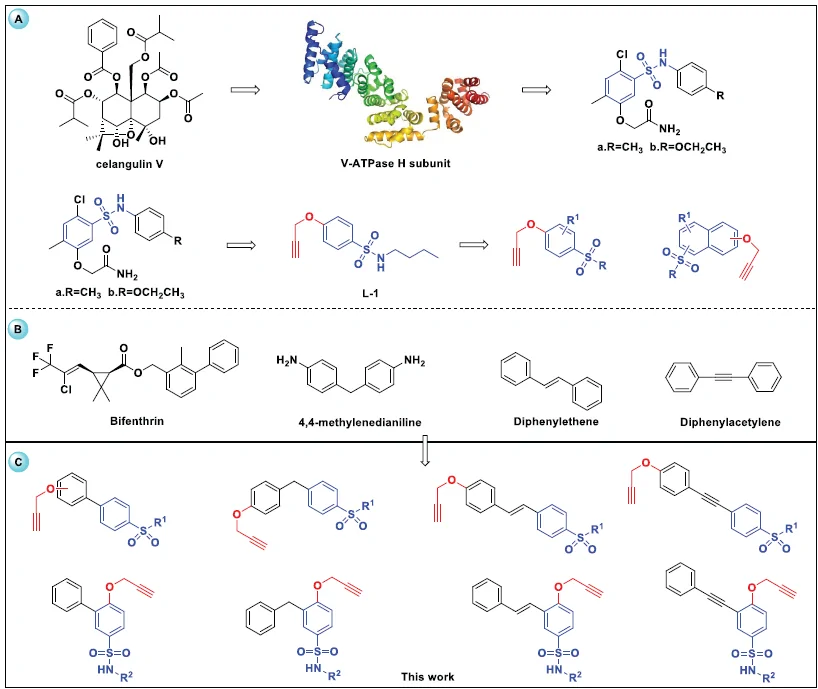
Design strategy of the target compounds. (**A**) Research on compounds based on V-ATPase; (**B**) Application of related compound structures in pesticide development; (**C**) Design of the present study.

**Fig. (2):**
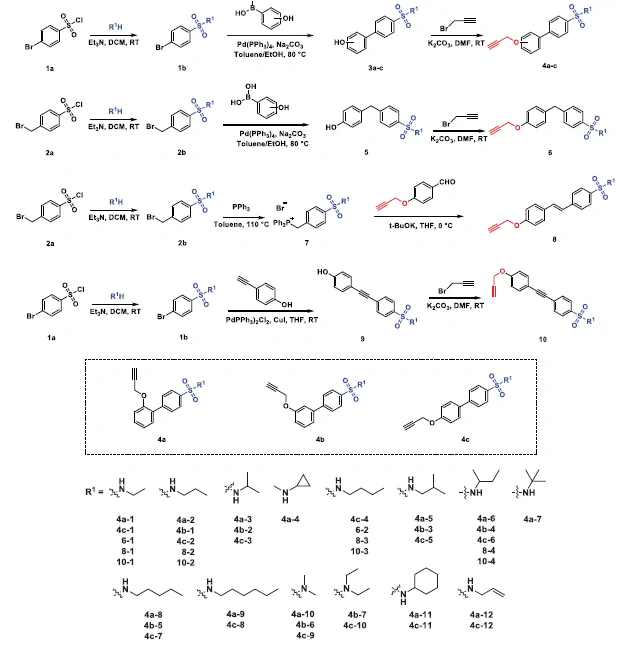
Synthetic route for synthesizing the target compounds.

**Fig. (3):**
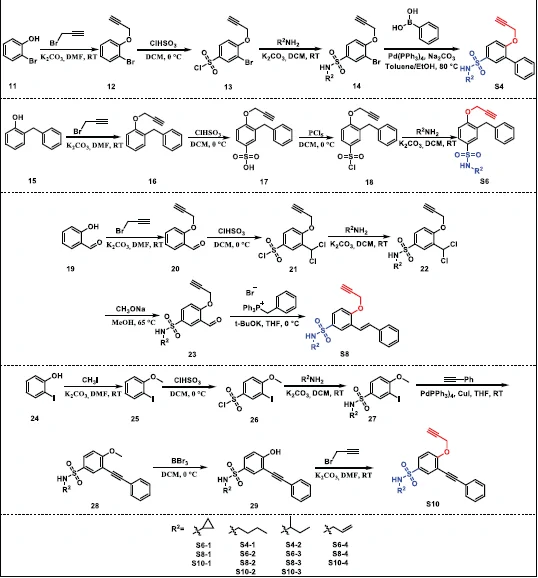
Synthetic route for the synthesis of the target compounds.

**Fig. (4):**
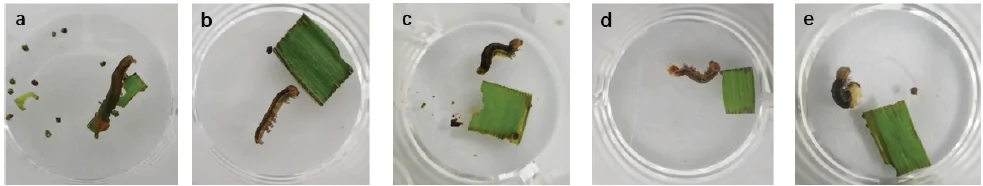
Different symptoms observed in the test insects: (**a**) blank control; (**b**) insects rendered unconscious and immobilized; (**c**) insects displaying contorted writhing movements; (**d**) bodily fluids exuding from the posterior ends; (**e**) desiccated and deceased insects.

**Fig. (5):**
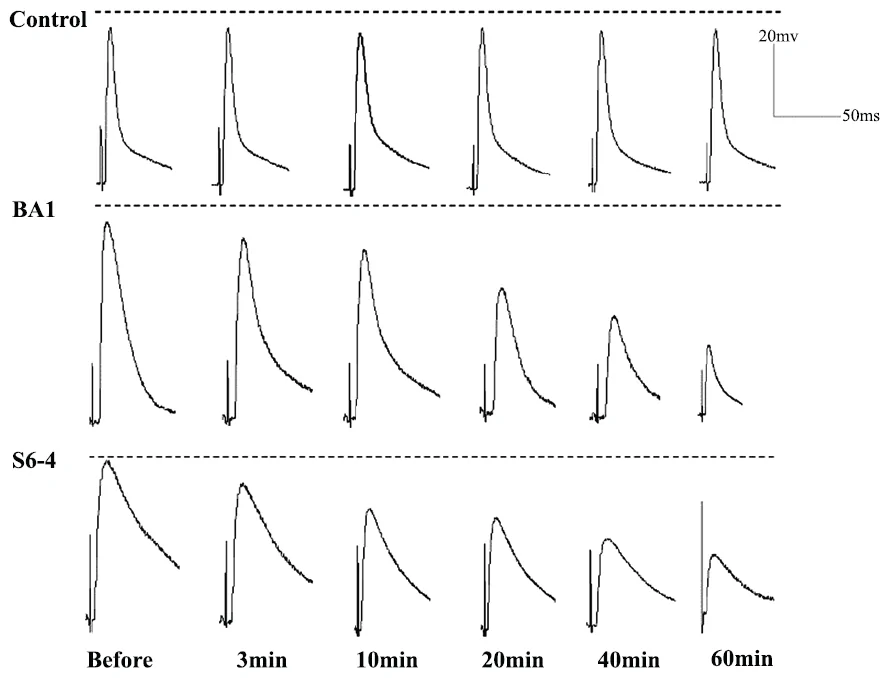
Representative traces of EJP (excitatory junctional potential) in fruit flies, recorded at various time points (0, 3, 10, 20, 40, and 60 min) after treatment with DMSO, 10 μM **S6-4,** and 2 μM BA1.

**Fig. (6):**
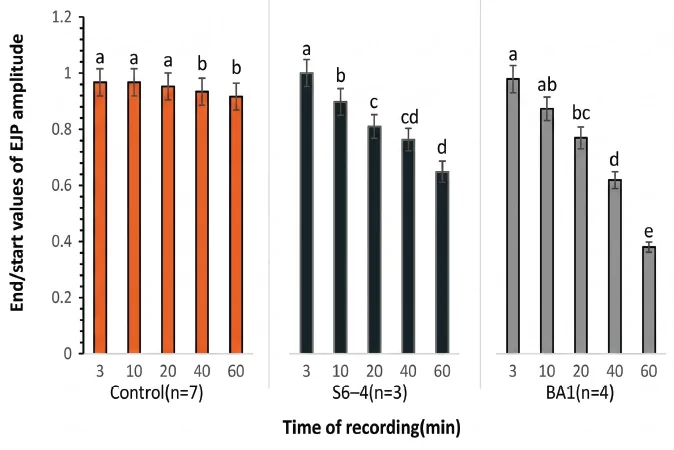
EJP amplitude statistical analysis chart. **Note:** Effects of DMSO (control), 10 μM **S6-4,** and 2 μM BA1 on evoked EJP amplitudes in w1118 flies at different time points (3, 10, 20, 40, and 60 min). Data are presented as normalized mean amplitudes.

**Fig. (7):**
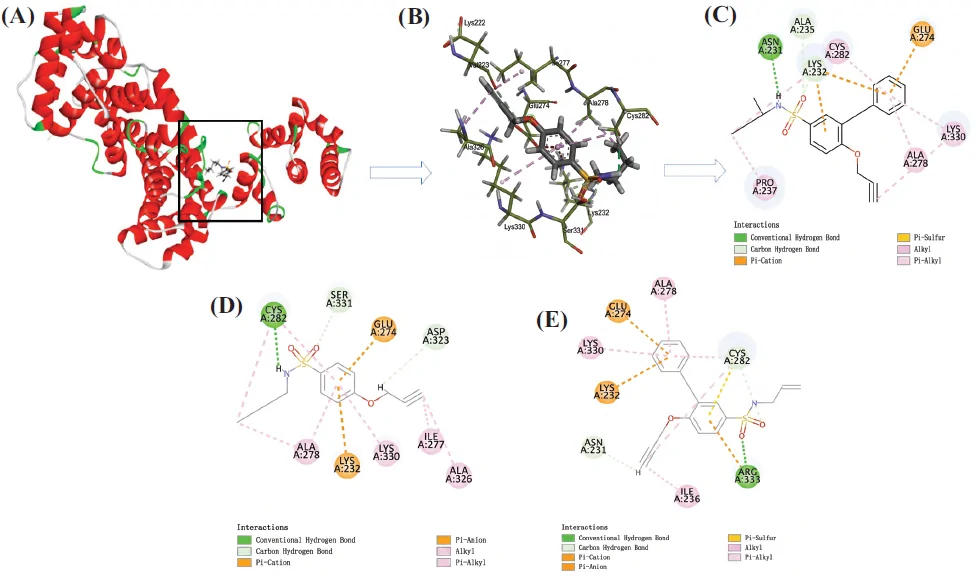
Binding of the H subunit of V-ATPase with compounds in the midgut of *M. separata* larvae. A: **S6-3**; B: **S6-3**; C: **S6-3**; D: **L-1**; E: **S6-4.**

**Table 1:** Insecticidal activity of the 4-series compounds against M. separata.

**Compounds**	**Mortality ± SD (%)**		**Compounds**	**Mortality ± SD (%)**	
	**10 mg/mL**	**1 mg/mL**		**10 mg/mL**	**1 mg/mL**
**4a-1**	15.2 ± 2.5	/	**4b-7**	66.7 ± 2.5	33.3 ± 2.5
**4a-2**	6.1 ± 2.5	/	**4c-1**	69.7 ± 2.5	27.3 ± 2.5
**4a-3**	6.1 ± 2.5	/	**4c-2**	87.9 ± 2.5	9.1 ± 4.3
**4a-4**	18.2 ± 4.3	/	**4c-3**	78.8 ± 4.3	12.1 ± 2.5
**4a-5**	15.2 ± 2.5	/	**4c-4**	81.9 ± 4.3	24.2 ± 4.3
**4a-6**	9.1 ± 4.3	/	**4c-5**	66.7 ± 2.5	12.1 ± 2.5
**4a-7**	9.1 ± 0.0	/	**4c-6**	87.9 ± 6.6	51.5 ± 2.5
**4a-8**	/	/	**4c-7**	51.5 ± 2.5	/
**4a-9**	3.0 ± 2.5	/	**4c-8**	30.3 ± 2.5	/
**4a-10**	6.1 ± 2.5	/	**4c-9**	33.3 ± 2.5	
**4a-11**	9.1 ± 4.3	/	**4c-10**	60.6 ± 2.5	9.1 ± 0.0
**4a-12**	48.5 ± 2.5	/	**4c-11**	45.5 ± 4.3	/
**4b-1**	27.3 ± 4.3	/	**4c-12**	80.6 ± 4.3	66.7 ± 2.5
**4b-2**	45.5 ± 4.3	/	**indoxacarb**	100	100
**4b-3**	3.0 ± 2.5	/	**celangulin V**	36.4 ± 4.3	/
**4b-4**	57.6 ± 2.5	/	**L-1**	75.8 ± 2.5	55.6 ± 2.5
**4b-5**	9.1 ± 4.3	/	**CK**	0	0
**4b-6**	24.2 ± 4.3	/	-	-	-

**Table 2:** Insecticidal activity of compounds 6, 8 and 10 against M. separate.

**Compounds**	**Mortality ± SD (%)**		**Compounds**	**Mortality ± SD (%)**	
	**10 mg/mL**	**1 mg/mL**		**10 mg/mL**	**1 mg/mL**
**6-1**	97.0 ± 2.5	36.1 ± 2.5	**10-2**	24.2 ± 2.5	/
**6-2**	90.9 ± 4.3	30.6 ± 4.3	**10-3**	27.3 ± 4.3	/
**8-1**	39.4 ± 2.5	/	**10-4**	60.6 ± 2.5	6.1 ± 2.5
**8-2**	18.2 ± 4.3	/	**indoxacarb**	100	100
**8-3**	39.4 ± 2.5	/	**celangulin V**	38.9 ± 4.3	/
**8-4**	78.8 ± 2.5	27.8 ± 2.5	**CK**	0	0
**10-1**	15.2 ± 4.3	/	**-**	-	-

**Table 3:** Insecticidal activity of S-series compounds against M. separate.

**Compounds**	**Mortality ± SD (%)**		**Compounds**	**Mortality ± SD (%)**	
	**10 mg/mL**	**1 mg/mL**		**10 mg/mL**	**1 mg/mL**
**S4-1**	51.5 ± 2.5	15.2 ± 2.5	**S8-4**	81.8 ± 4.3	30.3 ± 2.5
**S4-2**	57.6 ± 2.5	15.2 ± 4.3	**S10-1**	78.8 ± 2.5	51.5 ± 4.3
**S6-1**	60.6 ± 2.5	49.7 ± 2.5	**S10-2**	30.3 ± 6.6	/
**S6-2**	78.8 ± 4.3	42.4 ± 4.3	**S10-3**	97.0 ± 2.5	48.5 ± 2.5
**S6-3**	100	97.0 ± 2.5	**S10-4**	59.1 ± 4.3	30.3 ± 2.5
**S6-4**	100	60.6 ± 6.6	**indoxacarb**	100	100
**S8-1**	57.6 ± 2.5	39.4 ± 2.5	**celangulin V**	30.7 ± 2.5	/
**S8-2**	51.5 ± 4.3	18.2 ± 4.3	**CK**	0	0
**S8-3**	69.7 ± 6.6	57.6 ± 2.5	**-**	-	-

**Table 4:** LC50 of the selected compounds.

**Compounds**	**Toxicity regression equation**	**LC_50_ (mg/mL)**	**R^2^**
**4c-7**	y=1.2985x+0.9639	1.283	0.9736
**4c-14**	y=1.0595x+2.1237	0.519	0.9649
**S6-3**	y=2.2750x-1.0863	0.474	0.9776
**S6-4**	y=2.9020x-2.5527	0.401	0.9658
**S8-3**	y=2.1548x-1.2935	0.833	0.9856
**S10-1**	y=1.3526x+0.9996	0.907	0.9804
**celangulin V**	y=1.3066x-0.5628	18.091	0.9707
**L-1**	y=1.1391x+1.4681	1.261	0.9989
**indoxacarb**	y=1.7800x+4.1282	0.005	0.9476

## Data Availability

The authors confirm that the data supporting the findings of this study are available within the article and it’s supplementary material.

## LIST OF ABBREVIATIONS

V-ATPase: Vacuolar-type H⁺-ATPase
BA1: Bafilomycin A1
DMSO: Dimethyl Sulfoxide
EJP: Excitatory Junction Potential
LC₅₀: Lethal Concentration 50
NMR: Nuclear Magnetic Resonance
ESI-MS: Electrospray Ionization Mass Spectrometry
DCM: Dichloromethane
DMF: N, N-dimethylformamide
THF: Tetrahydrofuran
Pd (PPh₃)₄: Tetrakis (triphenylphosphine) Palladium
t-BuOK: Potassium tert-butoxide
PCl₅: Phosphorus Pentachloride
SPSS: Statistical Product and Service Solutions
