# Supplementary material for: Synthesis of Diphenyl Sulfonamide Derivatives as Potential Inhibitors of V-ATPase
Source: Curr Org Synth. 2026 Apr 8;23(3):193–212. doi: 10.2174/0115701794397334251126043618 (PMC13625036; doi:10.2174/0115701794397334251126043618)

Supplementary Materials

Synthesis of Diphenyl Sulfonamide Derivatives as Potential Inhibitors of V-ATPase

Mei Tian[1],Tao Guo[1], Jianjun Zhu[1], Yi Long[1], Ziyu Wang[1], Ziguang Shang[1], Jingxiu Zhang[1], Ruiyu Liu[2], Wenjun Wu[3], Zhaonong Hu[3] and Jiwen Zhang[1,3,*]

[1]College of Chemistry & Pharmacy, Northwest A&F University, Yangling, Shaanxi 712100, People's Republic of China; [2]College of Life Science, Northwest A&F University, Yangling, Shaanxi 712100, People's Republic of China; [3]Key Laboratory of Botanical Pesticide R&D in Shaanxi Province, Yangling 712100, People's Republic of China

1. Characterization data

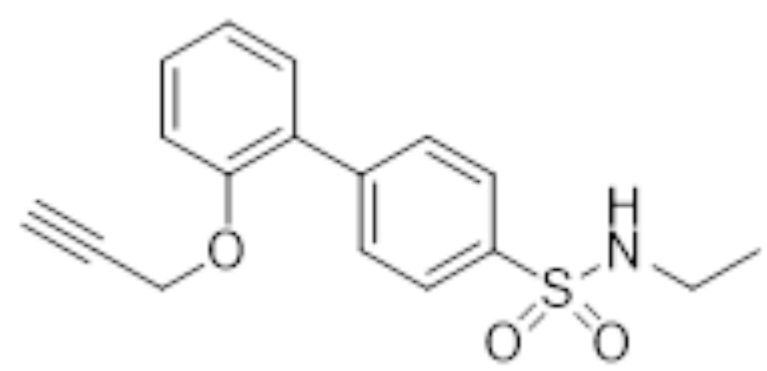

Fig. C1 The chemical structure of compound **4a-1**.

N-ethyl-2'-(prop-2-yn-1-yloxy)-[1,1'-biphenyl]-4-sulfonamide (**4a-1**). white solid, yield 27%. m.p.58.6~58.9□. ^{1}H NMR (500 MHz, Chloroform-*d*) δ 7.89 (d, *J* = 6.9 Hz, 2H), 7.68 (d, *J* = 7.3 Hz, 2H), 7.39 (t, *J* = 8.0 Hz, 1H), 7.34 (d, *J* = 7.6 Hz, 1H), 7.15 (d, *J* = 8.1 Hz, 1H), 7.11 (t, *J* = 7.1 Hz, 1H), 4.70 (d, *J* = 2.9 Hz, 2H), 4.46 (t, *J* = 6.2 Hz, 1H), 3.08 (pd, *J* = 7.2, 2.3 Hz, 2H), 2.50 (t, *J* = 2.6 Hz, 1H), 1.15 (t, *J* = 6.6 Hz, 3H). ^{13}C NMR (126 MHz, Chloroform-*d*) δ 154.56, 143.01, 131.13, 130.38, 129.72, 129.66, 126.90, 122.26, 113.57, 78.54, 75.94, 56.41, 38.49, 15.34. ESI-MS (MeOH) m/z $C_{17}H_{17}NO_3S$ $[M + H]^+$ calcd. for:316.10, found:316.16.

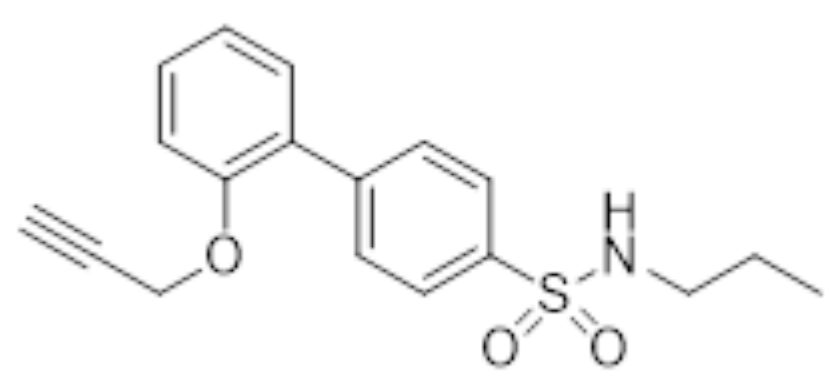

Fig. C2 The chemical structure of compound **4a-2**.

2'-(prop-2-yn-1-yloxy)-*N*-propyl-[1,1'-biphenyl]-4-sulfonamide (**4a-2**). white solid, yield 26%. m.p.59.4~61.7□. ^{1}H NMR (500 MHz, Chloroform-*d*) δ 7.89 (d, *J* = 6.7 Hz, 2H), 7.68 (d, *J* = 8.6 Hz, 2H), 7.38 (t, *J* = 8.1 Hz, 1H), 7.34 (d, *J* = 7.6 Hz, 1H), 7.15 (d, *J* = 7.9 Hz, 1H), 7.11 (t, *J* = 6.6 Hz, 1H), 4.69 (s, 2H), 4.56 (d, *J* = 6.7 Hz, 1H), 2.98 (td, *J* = 7.4, 3.6 Hz, 2H), 2.50 (d, *J* = 2.9 Hz, 1H), 1.54 (qd, *J* = 7.4, 2.3 Hz, 2H), 0.90 (t, *J* = 7.1 Hz, 3H). ^{13}C NMR (126 MHz, Chloroform-*d*) δ 154.55, 142.95, 138.48, 131.12, 130.36, 129.70, 129.62, 126.86, 122.25, 113.57, 78.53, 75.92, 56.41, 45.20, 23.19, 11.25. ESI-MS (MeOH) m/z $C_{18}H_{19}NO_3S$ $[M + H]^+$ calcd. for:330.12, found:330.16.

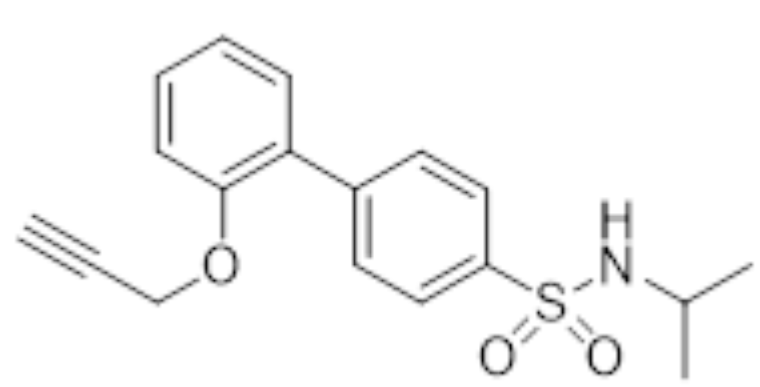

Fig. C3 The chemical structure of compound **4a-3**.

N-isopropyl-2'-(prop-2-yn-1-yloxy)-[1,1'-biphenyl]-4-sulfonamide (**4a-3**). white solid, yield 31%. m.p.82.1~83.5□. ^{1}H NMR (500 MHz, Chloroform-*d*) δ 7.96 (d, *J* = 7.2 Hz, 2H), 7.72 (d, *J* = 7.6 Hz, 2H), 7.43 (d, *J* = 7.6 Hz, 1H), 7.39 (d, *J* = 7.9 Hz,

 © 2026 Bentham Science Publishers

1H), 7.19 (d, *J* = 8.1 Hz, 1H), 7.14 (d, *J* = 6.8 Hz, 1H), 4.73 (s, 2H), 4.66 (d, *J* = 7.5 Hz, 1H), 3.63 – 3.50 (m, 1H), 2.54 (d, *J* = 2.9 Hz, 1H), 1.17 (d, *J* = 7.0 Hz, 6H). ^{13}C NMR (126 MHz, Chloroform-*d*) δ 154.54, 142.81, 139.47, 131.11, 130.31, 129.79, 129.66, 126.78, 122.22, 113.59, 78.53, 75.90, 56.40, 46.28, 23.97. ESI-MS (MeOH) m/z $C_{18}H_{19}NO_3S$ $[M + H]^+$ calcd. for:330.12, found:330.14.

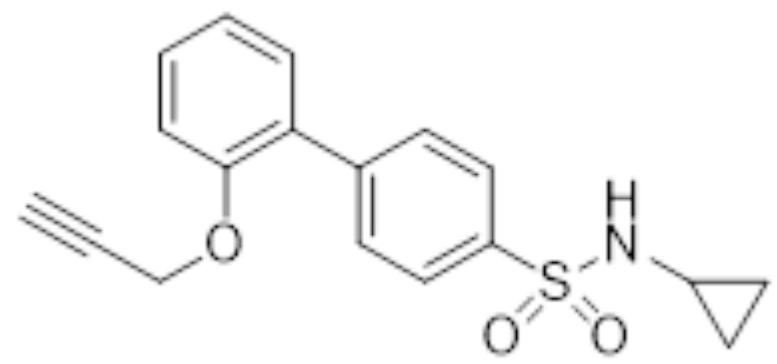

Fig. C4 The chemical structure of compound **4a-4**.

N-cyclopropyl-2'-(prop-2-yn-1-yloxy)-[1,1'-biphenyl]-4-sulfonamide (**4a-4**). white solid, yield 31%. m.p.77.4~76.6□. ^{1}H NMR (400 MHz, Chloroform-*d*) δ 7.93 (d, *J* = 8.5 Hz, 2H), 7.70 (d, J = 8.5 Hz, 2H), 7.39 (ddd, *J* = 8.3, 7.3, 1.7 Hz, 1H), 7.35 (dd, *J* = 7.6, 1.7 Hz, 1H), 7.15 (dd, *J* = 8.4, 1.0 Hz, 1H), 7.11 (td, *J* = 7.5, 1.1 Hz, 1H), 5.01 (s, 1H), 4.70 (d, *J* = 2.4 Hz, 2H), 2.50 (t, *J* = 2.4 Hz, 1H), 2.31 (dtdd, *J* = 9.3, 6.9, 4.7, 2.1 Hz, 1H), 0.73 – 0.58 (m, 4H). ^{13}C NMR (101 MHz, Chloroform-*d*) δ 154.46, 143.14, 137.85, 131.12, 130.32, 130.07, 129.74, 127.19, 122.20, 113.43, 78.48, 75.96, 56.31, 24.52, 6.39. ESI-MS (MeOH) m/z $C_{18}H_{19}NO_3S$ $[M + H]^+$ calcd. for:328.10, found:328.13.

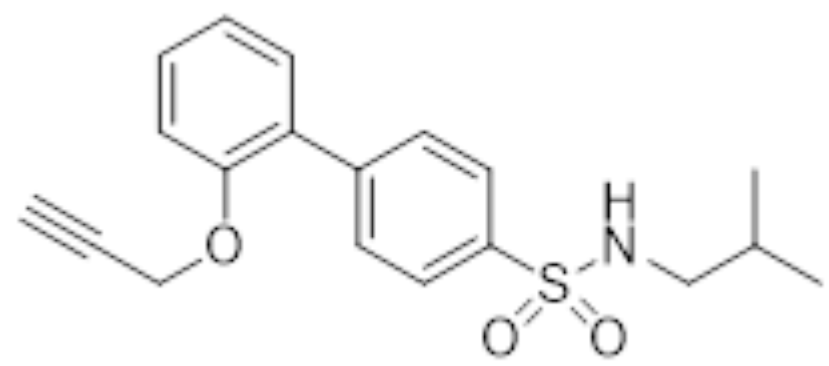

Fig. C5 The chemical structure of compound **4a-5**.

N-isobutyl-2'-(prop-2-yn-1-yloxy)-[1,1'-biphenyl]-4-sulfonamide (**4a-5**). white solid, yield 24%. m.p.75.8~76.2□. ^{1}H NMR (500 MHz, Chloroform-*d*) δ 7.89 (d, *J* = 7.9 Hz, 2H), 7.68 (dd, *J* = 8.3, 2.7 Hz, 2H), 7.41 – 7.36 (m, 1H), 7.34 (d, *J* = 7.3 Hz, 1H), 7.15 (d, *J* = 8.1 Hz, 1H), 7.10 (d, *J* = 7.1 Hz, 1H), 4.80 (dd, *J* = 17.7, 10.3 Hz, 1H), 4.69 (s, 2H), 2.82 (q, *J* = 4.1 Hz, 2H), 2.50 (d, *J* = 2.9 Hz, 1H), 1.77 (dtd, *J* = 11.4, 6.8, 3.4 Hz, 1H), 0.90 (d, *J* = 5.7 Hz, 6H). ^{13}C NMR (126 MHz, Chloroform-*d*) δ 154.50, 142.87, 138.46, 131.10, 130.33, 129.72, 129.67, 126.81, 122.21, 113.51, 75.93, 56.35, 50.76, 28.66, 20.01. ESI-MS (MeOH) m/z $C_{19}H_{21}NO_3S$ $[M + H]^+$ calcd. for:344.13, found:344.16.

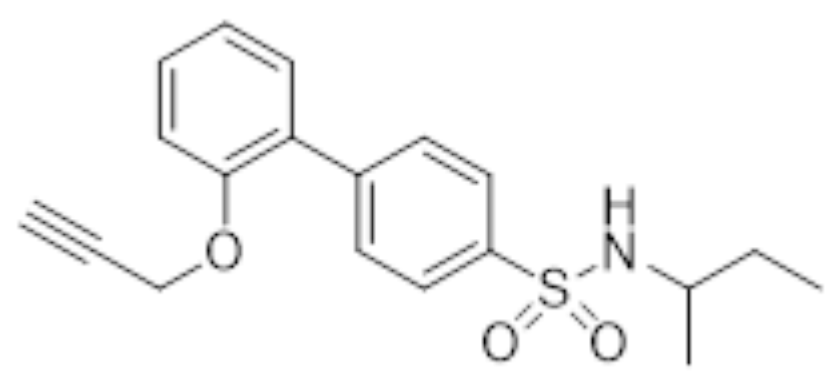

Fig. C6 The chemical structure of compound **4a-6**.

N-(sec-butyl)-2'-(prop-2-yn-1-yloxy)-[1,1'-biphenyl]-4-sulfonamide (**4a-6**). white solid, yield 28%. m.p. 82.6~86.4□. ^{1}H NMR (500 MHz, Chloroform-*d*) δ 7.91 (d, *J* = 7.6 Hz, 2H), 7.67 (d, *J* = 7.1 Hz, 2H), 7.38 (t, *J* = 7.8 Hz, 1H), 7.34 (d, *J* = 7.9 Hz, 1H), 7.14 (d, *J* = 8.1 Hz, 1H), 7.10 (d, *J* = 6.6 Hz, 1H), 4.67 (d, *J* = 2.5 Hz, 2H), 4.55 (dd, *J* = 23.4, 7.9 Hz, 1H), 3.32 (p, *J* = 7.0 Hz, 1H), 2.48 (t, *J* = 2.6 Hz, 1H), 1.44 (p, *J* = 7.7 Hz, 2H), 1.07 (d, *J* = 6.6 Hz, 3H), 0.83 (t, *J* = 7.3 Hz, 3H).^{13}C NMR (126 MHz, Chloroform-*d*) δ 154.55, 142.77, 139.60, 131.09, 130.27, 129.83, 129.65, 126.77, 122.23, 113.64, 78.52, 75.87, 56.43, 51.59, 30.47, 21.36, 10.04. ESI-MS (MeOH) m/z $C_{19}H_{21}NO_3S$ $[M + H]^+$ calcd. for:344.13, found:344.17.

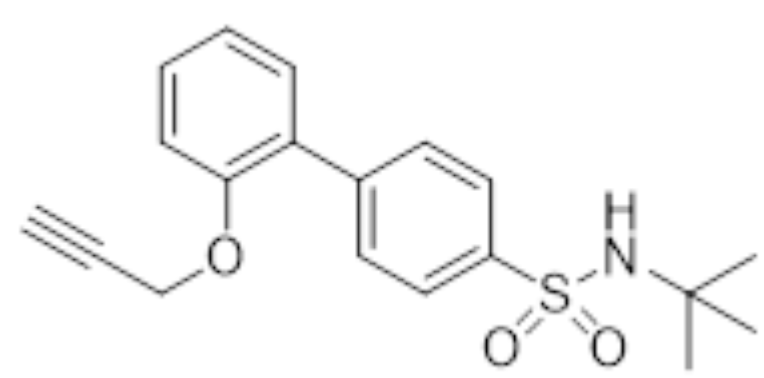

Fig. C7 The chemical structure of compound **4a-7**.

N-(tert-butyl)-2'-(prop-2-yn-1-yloxy)-[1,1'-biphenyl]-4-sulfonamide (**4a-7**). white solid, yield 30%. m.p.110.4~110.9□. ^{1}H NMR (500 MHz, Chloroform-*d*) δ 7.98 (d, *J* = 7.3 Hz, 2H), 7.70 (d, *J* = 7.6 Hz, 2H), 7.42 (t, *J* = 8.0 Hz, 1H), 7.39 (d, *J* = 7.7 Hz, 1H), 7.19 (d, *J* = 8.3 Hz, 1H), 7.14 (t, *J* = 7.0 Hz, 1H), 5.14 (s, 1H), 4.72 (d, *J* = 2.5 Hz, 2H), 2.53 (t, *J* = 2.5 Hz, 1H), 1.31 (s, 9H). ^{13}C NMR (126 MHz, Chloroform-*d*) δ 154.52, 142.40, 141.81, 131.08, 130.09, 129.84, 129.55, 126.67, 122.17, 113.60, 78.54, 75.86, 56.36, 54.79, 30.30. ESI-MS (MeOH) m/z $C_{19}H_{21}NO_3S$ $[M + H]^+$ calcd. for:344.13, found:344.18.

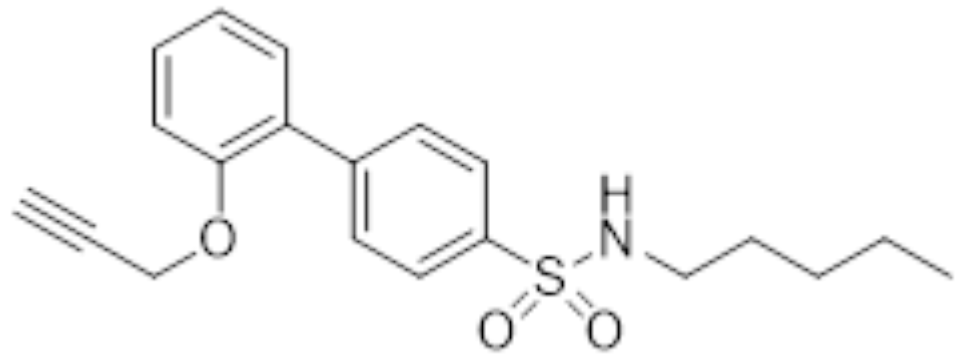

Fig. C8 The chemical structure of compound **4a-8**.
N-pentyl-2'-(prop-2-yn-1-yloxy)-[1,1'-biphenyl]-4-sulfonamide (**4a-8**). white solid, yield 27%. m.p.58.2~59.7□. ^{1}H NMR (500 MHz, Chloroform-*d*) δ 7.94 (d, *J* = 7.4 Hz, 2H), 7.72 (d, *J* = 7.9 Hz, 2H), 7.43 (t, *J* = 8.0 Hz, 1H), 7.38 (d, *J* = 7.6 Hz, 1H), 7.20 (d, *J* = 8.3 Hz, 1H), 7.15 (t, *J* = 7.5 Hz, 1H), 4.76 (t, *J* = 6.6 Hz, 1H), 4.73 (d, *J* = 2.9 Hz, 2H), 3.05 (q, *J* = 6.5 Hz, 2H), 2.54 (t, *J* = 2.9 Hz, 1H), 1.60 – 1.49 (m, 2H), 1.36 – 1.24 (m, 4H), 0.89 (t, *J* = 3.8, 2.7 Hz, 3H). ^{13}C NMR (126 MHz, Chloroform-*d*) δ 154.48, 142.90, 138.38, 131.10, 130.33, 129.70, 129.67, 126.84, 122.19, 113.47, 78.50, 75.93, 56.31, 43.42, 29.42, 28.77, 22.27, 13.99. ESI-MS (MeOH) m/z $C_{20}H_{23}NO_3S$ $[M + H]^+$ calcd. for:358.15, found:358.19.

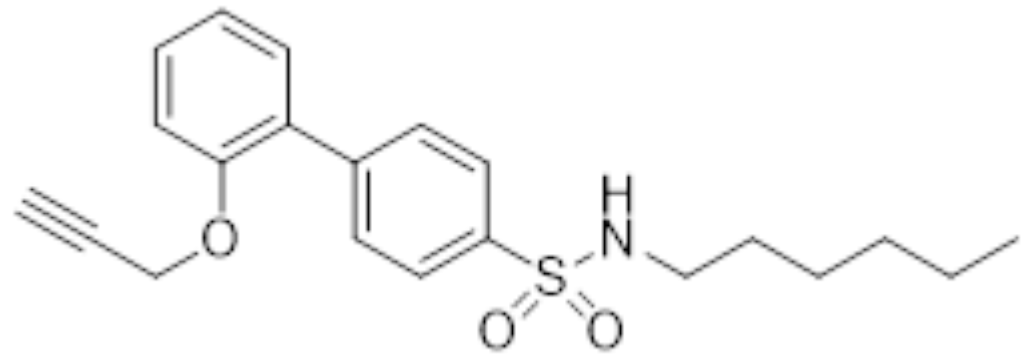

Fig. C9 The chemical structure of compound **4a-9**.
N-hexyl-2'-(prop-2-yn-1-yloxy)-[1,1'-biphenyl]-4-sulfonamide (**4a-9**). white solid, yield 29%. m.p.49.8~51.5□^{1}H NMR (400 MHz, Chloroform-*d*) δ 7.89 (d, *J* = 8.5 Hz, 2H), 7.68 (d, *J* = 8.5 Hz, 2H), 7.42 – 7.35 (m, 1H), 7.34 (dd, *J* = 7.6, 1.7 Hz, 1H), 7.15 (dd, *J* = 8.4, 1.0 Hz, 1H), 7.11 (td, *J* = 7.5, 1.1 Hz, 1H), 4.70 (d, *J* = 2.4 Hz, 3H), 3.00 (q, *J* = 6.8 Hz, 2H), 2.50 (t, *J* = 2.4 Hz, 1H), 1.56 – 1.38 (m, 2H), 1.35 – 1.17 (m, 6H), 0.85 (t, *J* = 6.8 Hz, 3H). ^{13}C NMR (101 MHz, Chloroform-*d*) δ 154.44, 142.89, 138.30, 131.11, 130.33, 129.68, 129.64, 126.85, 122.18, 113.39, 78.48, 75.95, 56.27, 43.45, 31.38, 29.70, 26.32, 22.58, 14.09. ESI-MS (MeOH) m/z $C_{21}H_{25}NO_3S$ $[M + H]^+$ calcd. for:372.16, found:372.21.

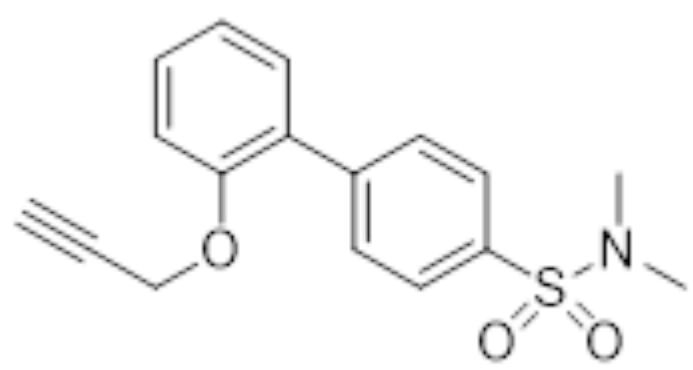

Fig. C10 The chemical structure of compound **4a-10**.
N,N-dimethyl-2'-(prop-2-yn-1-yloxy)-[1,1'-biphenyl]-4-sulfonamide (**4a-10**). white solid, yield 33%. m.p.91.5~93.8□. ^{1}H NMR (500 MHz, Chloroform-*d*) δ 7.85 (d, *J* = 7.2 Hz, 2H), 7.75 (d, *J* = 8.5 Hz, 2H), 7.43 (t, *J* = 8.0 Hz, 1H), 7.39 (d, *J* = 7.7 Hz, 1H), 7.20 (d, *J* = 9.0 Hz, 1H), 7.18 – 7.13 (m, 1H), 4.75 (d, *J* = 2.6 Hz, 2H), 2.80 (s, 6H), 2.55 (t, *J* = 2.5 Hz, 1H). ^{13}C NMR (126 MHz, Chloroform-*d*) δ 154.45, 142.99, 133.93, 131.10, 130.23, 129.71, 129.58, 127.53, 122.20, 113.45, 78.48, 75.94, 56.30, 38.09. ESI-MS (MeOH) m/z $C_{17}H_{17}NO_3S$ $[M + H]^+$ calcd. for:316.10, found:316.13.

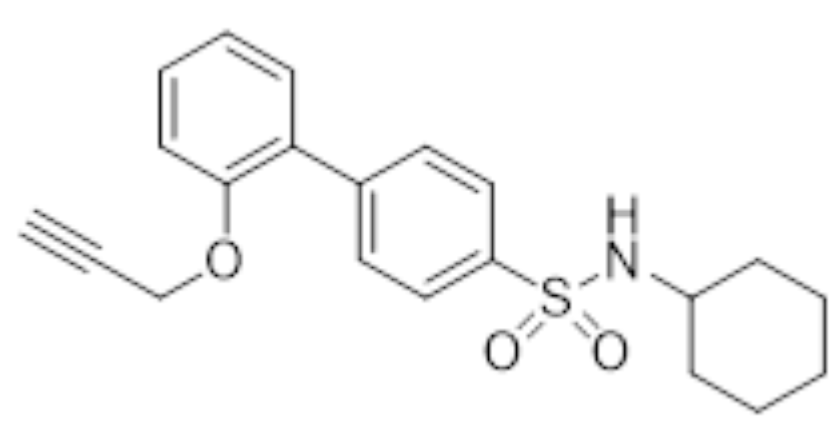

Fig. C11 The chemical structure of compound **4a-11**.
N-cyclohexyl-2'-(prop-2-yn-1-yloxy)-[1,1'-biphenyl]-4-sulfonamide (**4a-11**). white solid, yield 23%. m.p.66.7~67.9□. ^{1}H NMR (400 MHz, Chloroform-*d*) δ 7.91 (d, *J* = 8.4 Hz, 2H), 7.67 (d, *J* = 8.4 Hz, 2H), 7.41 – 7.36 (m, 1H), 7.36 – 7.33 (m, 1H), 7.15 (d, *J* = 8.2 Hz, 1H), 7.11 (td, *J* = 7.5, 1.1 Hz, 1H), 4.74 (d, *J* = 7.6 Hz, 1H), 4.69 (d, *J* = 2.4 Hz, 2H), 3.20 (tdt, *J* = 11.3, 7.9, 3.9 Hz, 1H), 2.49 (t, *J* = 2.4 Hz, 1H), 1.81 (dt, *J* = 12.1, 3.7 Hz, 2H), 1.66 (dq, *J* = 12.8, 3.8 Hz, 2H), 1.57 – 1.12 (m, 6H). ^{13}C NMR (101 MHz, Chloroform-*d*) δ 154.48, 142.68, 139.75, 131.12, 130.28, 129.72, 129.65, 126.67, 122.20, 113.50, 78.50, 75.91, 56.34, 52.78, 34.11, 25.28, 24.76. ESI-MS (MeOH) m/z $C_{21}H_{23}NO_3S$ $[M + H]^+$ calcd. for:370.15, found:370.20.

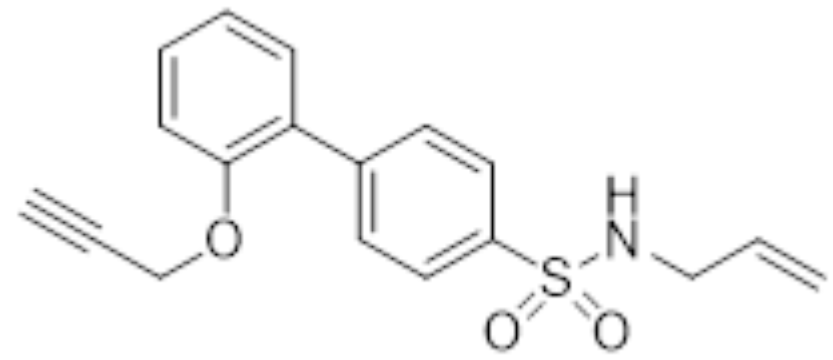

Fig. C12 The chemical structure of compound **4a-12**.

N-allyl-2'-(prop-2-yn-1-yloxy)-[1,1'-biphenyl]-4-sulfonamide (**4a-12**). white solid, yield 28%. m.p.120.7~125.1□. ^{1}H NMR (500 MHz, Chloroform-*d*) δ 7.94 (d, *J* = 8.5 Hz, 2H), 7.73 (d, *J* = 8.5 Hz, 2H), 7.47 – 7.40 (m, 1H), 7.38 (dd, *J* = 7.6, 1.8 Hz, 1H), 7.19 (d, *J* = 8.3 Hz, 1H), 7.15 (t, *J* = 7.3 Hz, 1H), 5.82 (ddt, *J* = 16.4, 10.1, 5.8 Hz, 1H), 5.30 – 5.13 (m, 2H), 4.80 (t, *J* = 6.3 Hz, 1H), 4.74 (d, *J* = 2.4 Hz, 2H), 3.70 (td, *J* = 5.2, 4.4, 3.0 Hz, 2H), 2.55 (t, *J* = 2.4 Hz, 1H).^{13}C NMR (126 MHz, Chloroform-*d*) δ 154.48, 143.07, 138.27, 133.17, 131.09, 130.39, 129.72, 129.64, 126.90, 122.21, 117.87, 113.47, 78.48, 75.95, 56.33, 45.96. ESI-MS (MeOH) m/z $C_{18}H_{17}NO_3S$ $[M + H]^+$ calcd. for:328.10, found:328.13.

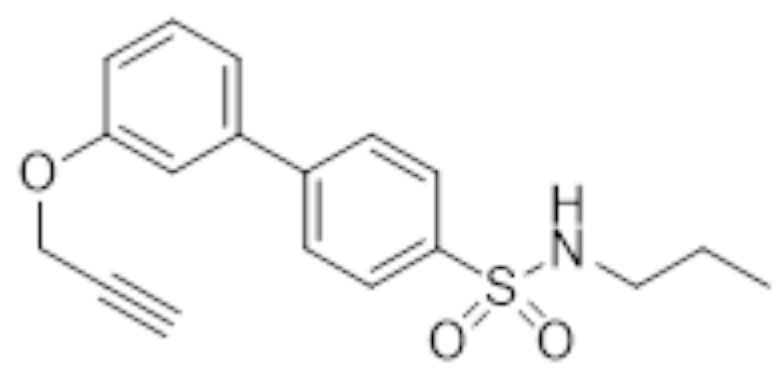

Fig. C13 The chemical structure of compound **4b-1**.

3'-(prop-2-yn-1-yloxy)-*N*-propyl-[1,1'-biphenyl]-4-sulfonamide (**4b-1**). white solid, yield 28%. m.p.49.8~51.5□. ^{1}H NMR (400 MHz, Chloroform-*d*) δ 7.93 (d, *J* = 8.6 Hz, 2H), 7.71 (d, *J* = 8.5 Hz, 2H), 7.41 (t, *J* = 7.9 Hz, 1H), 7.25 – 7.19 (m, 2H), 7.04 (ddd, *J* = 8.3, 2.6, 0.9 Hz, 1H), 4.77 (d, *J* = 2.4 Hz, 2H), 4.65 (q, *J* = 7.4, 6.2 Hz, 1H), 3.03 – 2.88 (m, 2H), 2.56 (t, *J* = 2.4 Hz, 1H), 1.52 (h, *J* = 7.3 Hz, 2H), 0.89 (t, *J* = 7.4 Hz, 3H). ^{13}C NMR (101 MHz, Chloroform-*d*) δ 158.14, 145.23, 140.98, 138.93, 130.26, 127.89, 127.70, 120.76, 114.77, 114.31, 78.46, 75.99, 56.03, 45.16, 23.12, 11.25. ESI-MS (MeOH) m/z $C_{18}H_{19}NO_3S$ $[M + H]^+$ calcd. for:330.12, found:330.15.

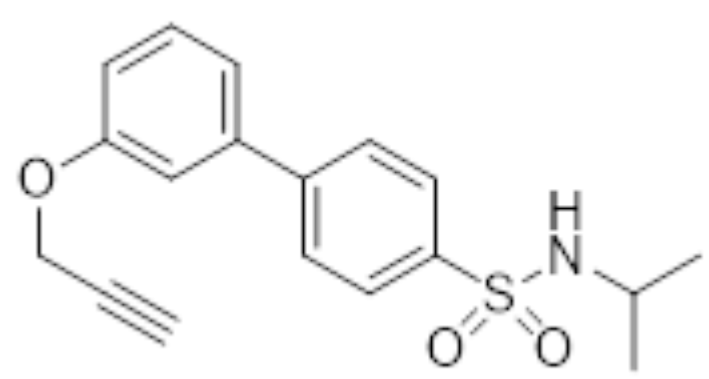

Fig. C14 The chemical structure of compound **4b-2**.

N-isopropyl-3'-(prop-2-yn-1-yloxy)-[1,1'-biphenyl]-4-sulfonamide (**4b-2**). white solid, yield 29%. m.p. 80.4~83.5□. ^{1}H NMR (500 MHz, Chloroform-*d*) δ 7.94 (dd, *J* = 8.2, 2.4 Hz, 2H), 7.74 – 7.67 (m, 2H), 7.41 (td, *J* = 8.0, 2.2 Hz, 1H), 7.23 (dd, *J* = 13.8, 5.2 Hz, 2H), 7.04 (dd, *J* = 8.3, 2.8 Hz, 1H), 4.76 (d, *J* = 2.5 Hz, 2H), 4.48 (d, *J* = 7.5 Hz, 1H), 3.52 (q, *J* = 7.0 Hz, 1H), 2.55 (t, *J* = 2.5 Hz, 1H), 1.12 (dd, *J* = 6.5, 2.3 Hz, 6H). ^{13}C NMR (126 MHz, Chloroform-*d*) δ 158.20, 145.14, 141.02, 140.13, 130.25, 127.85, 127.65, 120.78, 114.83, 114.37, 78.51, 75.97, 56.09, 46.33, 23.98. ESI-MS (MeOH) m/z $C_{18}H_{19}NO_3S$ $[M + H]^+$ calcd. for:330.12, found:330.12.

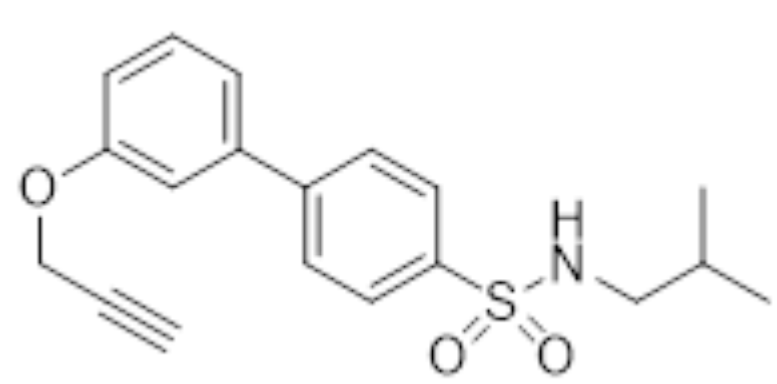

Fig. C15 The chemical structure of compound **4b-3**.

N-isobutyl-3'-(prop-2-yn-1-yloxy)-[1,1'-biphenyl]-4-sulfonamide （**4b-3**）. white solid, yield 26%. m.p.51.4~55.7□. ^{1}H NMR (500 MHz, Chloroform-*d*) δ 7.97 (d, *J* = 7.9 Hz, 2H), 7.75 (d, *J* = 6.1 Hz, 2H), 7.46 (td, *J* = 8.0, 2.3 Hz, 1H), 7.28 (dd, *J* = 15.6, 7.7 Hz, 2H), 7.09 (d, *J* = 8.1 Hz, 1H), 4.81 (d, *J* = 2.7 Hz, 2H), 4.66 (q, *J* = 10.0, 6.0 Hz, 1H), 2.86 (td, *J* = 6.7, 2.2 Hz, 2H), 2.60 (d, *J* = 2.6 Hz, 1H), 1.80 (dtt, *J* = 13.4, 6.8, 3.7 Hz, 1H), 0.94 (d, *J* = 5.7 Hz, 6H). ^{13}C NMR (126 MHz, Chloroform-*d*) δ 158.20, 145.24, 141.02, 139.08, 130.26, 127.89, 127.69, 120.78, 114.83, 114.37, 78.49, 75.97, 56.08, 50.78, 28.66, 20.01. ESI-MS (MeOH) m/z $C_{19}H_{21}NO_3S$ $[M + H]^+$ calcd. for:344.13, found:344.15.

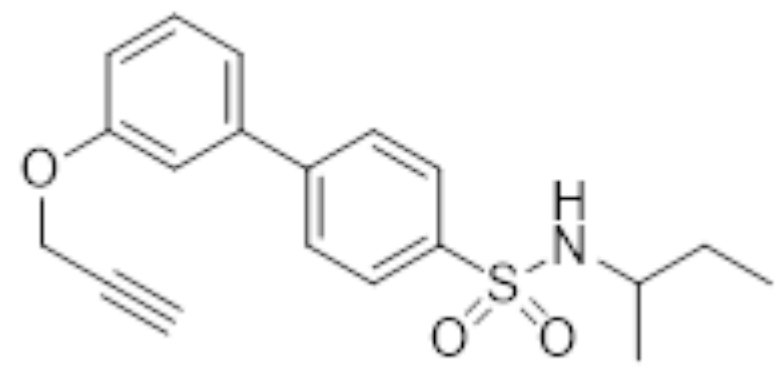

Fig. C16 The chemical structure of compound **4b-4**.

N-(sec-butyl)-3'-(prop-2-yn-1-yloxy)-[1,1'-biphenyl]-4-sulfonamide (**4b-4**). white solid, yield 27%. m.p.81.7~84.4□. ^{1}H NMR (500 MHz, Chloroform-*d*) δ 7.99 (d, *J* = 7.5 Hz, 2H), 7.74 (d, *J* = 7.7 Hz, 2H), 7.45 (t, *J* = 7.8 Hz, 1H), 7.29 (d, *J* = 8.1 Hz, 2H), 7.08 (d, *J* = 8.2 Hz, 1H), 4.80 (d, *J* = 2.7 Hz, 2H), 4.72 (t, *J* = 10.4 Hz, 1H), 3.35 (p, *J* = 7.9 Hz, 1H), 2.60 (t, *J* = 2.8 Hz, 1H), 1.48 (pd, *J* = 7.2, 2.2 Hz, 2H), 1.10 (d, *J* = 5.9 Hz, 3H), 0.87 (d, *J* = 7.1 Hz, 3H). ^{13}C NMR (126 MHz, Chloroform-*d*) δ 158.16, 145.01, 140.99, 140.25, 130.22, 127.76, 127.63, 120.74, 114.79, 114.33, 78.49, 75.96, 56.06, 51.63, 30.42, 21.32, 10.05. ESI-MS (MeOH) m/z $C_{19}H_{21}NO_3S$ $[M + H]^+$ calcd. for:344.13, found:344.20.

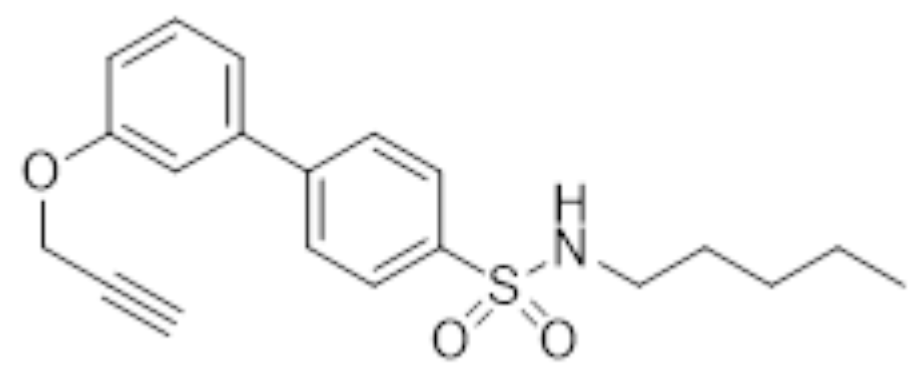

Fig. C17 The chemical structure of compound **4b-5**.

N-pentyl-3'-(prop-2-yn-1-yloxy)-[1,1'-biphenyl]-4-sulfonamide (**4b-5**). white solid, yield 23%. m.p.52.2~55.3□. ^{1}H NMR (500 MHz, Chloroform-*d*) δ 7.93 (d, *J* = 7.5 Hz, 2H), 7.71 (d, *J* = 7.4 Hz, 2H), 7.41 (t, *J* = 7.8 Hz, 1H), 7.27 – 7.19 (m, 2H), 7.04 (d, *J* = 7.7 Hz, 1H), 4.76 (d, *J* = 2.8 Hz, 2H), 4.58 (d, *J* = 6.5 Hz, 1H), 3.04 – 2.94 (m, 2H), 2.56 (d, *J* = 2.9 Hz, 1H), 1.56 – 1.42 (m, 2H), 1.26 (dt, *J* = 8.1, 4.0 Hz, 4H), 0.84 (h, *J* = 2.8 Hz, 3H). ^{13}C NMR (126 MHz, Chloroform-*d*) δ 158.19, 145.27, 141.03, 139.01, 130.26, 127.89, 127.72, 120.78, 114.81, 114.36, 78.49, 75.97, 56.07, 43.43, 29.44, 28.78, 22.27, 14.00. ESI-MS (MeOH) m/z $C_{20}H_{23}NO_3S$ $[M + H]^+$ calcd. for:358.15, found:358.21.

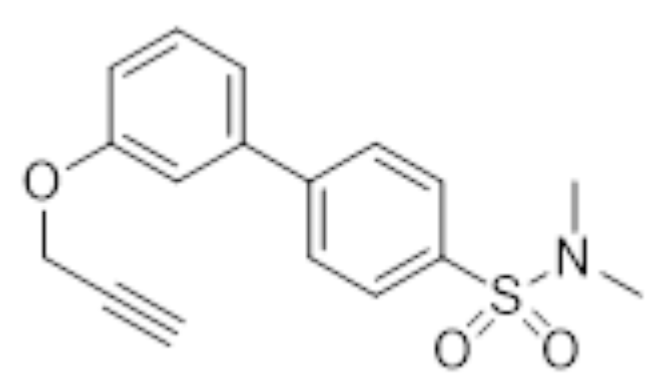

Fig. C18 The chemical structure of compound **4b-6**.

N,N-dimethyl-3'-(prop-2-yn-1-yloxy)-[1,1'-biphenyl]-4-sulfonamide (**4b-6**). white solid, yield 29%. m.p.93.3~93.8□. ^{1}H NMR (500 MHz, Chloroform-*d*) δ 7.88 (d, *J* = 8.4 Hz, 2H), 7.78 (d, *J* = 7.6 Hz, 2H), 7.46 (t, *J* = 7.4 Hz, 1H), 7.30 – 7.24 (m, 2H), 7.09 (d, *J* = 8.1 Hz, 1H), 4.81 (d, *J* = 2.6 Hz, 2H), 2.80 (s, 6H), 2.60 (t, *J* = 2.6 Hz, 1H). ^{13}C NMR (126 MHz, Chloroform-*d*) δ 158.21, 145.35, 140.97, 134.57, 130.28, 128.41, 127.81, 120.78, 114.85, 114.37, 78.49, 75.98, 56.08, 38.11. ESI-MS (MeOH) m/z $C_{17}H_{17}NO_3S$ $[M + H]^+$ calcd. for:316.10, found:316.14.

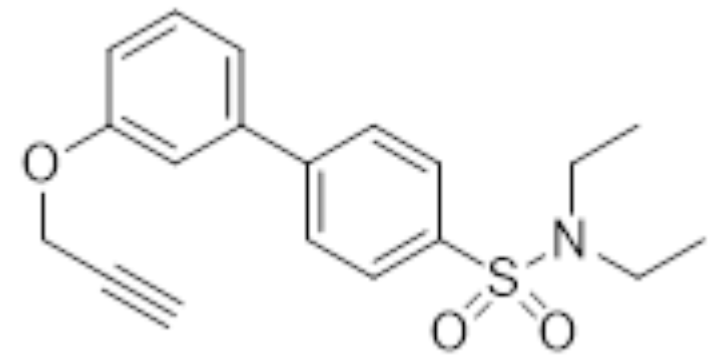

Fig. C19 The chemical structure of compound **4b-7**.

N,N-diethyl-3'-(prop-2-yn-1-yloxy)-[1,1'-biphenyl]-4-sulfonamide (**4b-7**). white solid, yield 29%. m.p.85.5~87.4□. ^{1}H NMR (500 MHz, Chloroform-*d*) δ 7.90 (d, *J* = 8.0 Hz, 2H), 7.72 (d, *J* = 6.8 Hz, 2H), 7.44 (t, *J* = 7.9 Hz, 1H), 7.30 – 7.23 (m, 2H), 7.07 (d, *J* = 8.1 Hz, 1H), 4.80 (d, *J* = 2.7 Hz, 2H), 3.32 (qd, *J* = 7.2, 2.4 Hz, 4H), 2.60 (t, *J* = 2.7 Hz, 1H), 1.20 (t, *J* = 6.7 Hz, 6H). ^{13}C NMR (126 MHz, Chloroform-*d*) δ 158.13, 144.82, 141.00, 139.34, 130.19, 127.74, 127.61, 120.70, 114.71, 114.27, 78.48, 75.94, 56.02, 42.23, 14.33. ESI-MS (MeOH) m/z $C_{19}H_{21}NO_3S$ $[M + H]^+$ calcd. for:344.13, found:344.18.

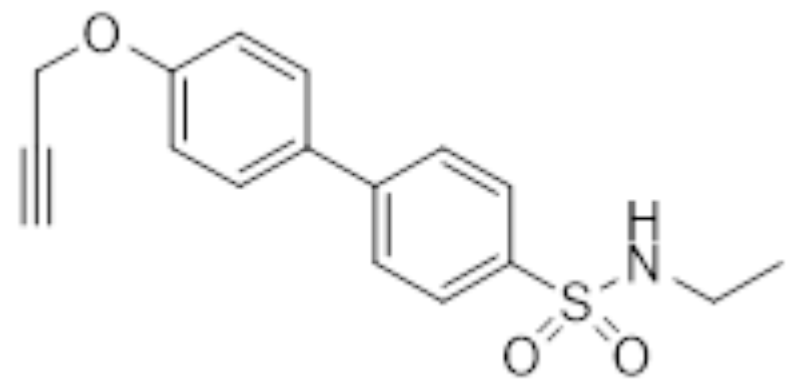

Fig. C20 The chemical structure of compound **4c-1**.

N-ethyl-4'-(prop-2-yn-1-yloxy)-[1,1'-biphenyl]-4-sulfonamide (**4c-1**). white solid, yield 28%. m.p.75.1~77.4□. ^{1}H NMR (500 MHz, Chloroform-*d*) δ 7.95 (d, *J* = 8.5 Hz, 2H), 7.72 (d, *J* = 8.5 Hz, 2H), 7.61 (d, *J* = 8.8 Hz, 2H), 7.13 (d, *J* = 8.8 Hz, 2H), 4.80 (d, *J* = 2.4 Hz, 2H), 4.64 (t, *J* = 6.0 Hz, 1H), 3.10 (qd, *J* = 7.2, 6.0 Hz, 2H), 2.61 (t, *J* = 2.4 Hz, 1H), 1.18 (t, *J* = 7.3 Hz, 3H). ^{13}C NMR (126 MHz, Chloroform-*d*) δ 158.07, 145.08, 138.08, 132.72, 128.58, 127.76, 127.34, 115.58, 78.39, 75.98, 56.00, 38.43, 15.25. ESI-MS (MeOH) m/z $C_{17}H_{17}NO_3S$ $[M + H]^+$ calcd. for:316.10, found:316.14.

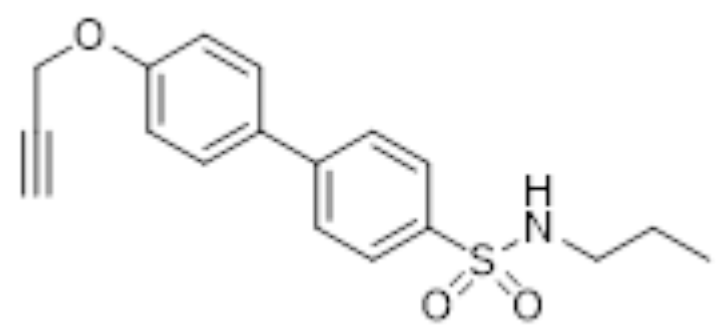

Fig. C21 The chemical structure of compound **4c-2**.

4'-(prop-2-yn-1-yloxy)-*N*-propyl-[1,1'-biphenyl]-4-sulfonamide (**4c-2**). white solid, yield 27%. m.p.65.1~66.4□. ^{1}H NMR (500 MHz, Chloroform-*d*) δ 7.95 (d, *J* = 8.5 Hz, 2H), 7.72 (d, *J* = 8.5 Hz, 2H), 7.61 (d, *J* = 8.8 Hz, 2H), 7.13 (d, *J* = 8.8 Hz, 2H), 4.80 (d, *J* = 2.4 Hz, 2H), 4.55 (t, *J* = 6.3 Hz, 1H), 3.01 (q, *J* = 6.8 Hz, 2H), 2.60 (t, *J* = 2.4 Hz, 1H), 1.56 (h, *J* = 7.3 Hz, 2H), 0.94 (t, *J* = 7.4 Hz, 3H). ^{13}C NMR (126 MHz, Chloroform-*d*) δ 158.08, 145.06, 138.17, 132.75, 128.59, 127.74, 127.34, 115.59, 78.40, 75.98, 56.01, 45.16, 23.14, 11.26. ESI-MS (MeOH) m/z $C_{18}H_{19}NO_3S$ $[M + H]^+$ calcd. for:330.12, found:330.16.

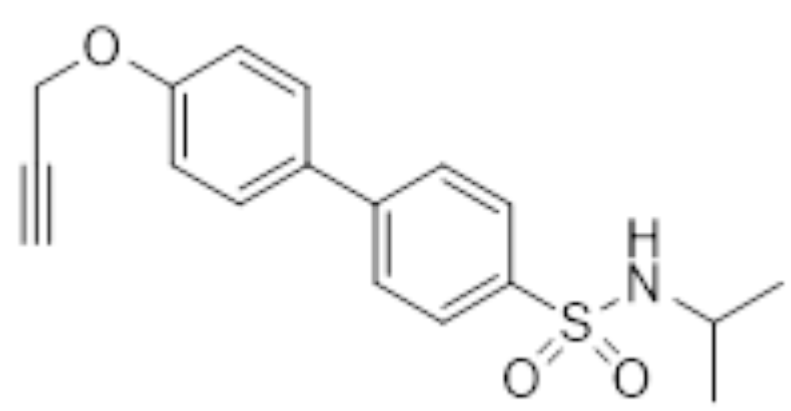

Fig. C22 The chemical structure of compound **4c-3**.

N-isopropyl-4'-(prop-2-yn-1-yloxy)-[1,1'-biphenyl]-4-sulfonamide (**4c-3**). white solid, yield 27%. m.p.83.4~83.9□. ^{1}H NMR (500 MHz, Chloroform-*d*) δ 7.96 (d, *J* = 8.4 Hz, 2H), 7.72 (d, *J* = 8.4 Hz, 2H), 7.61 (d, *J* = 8.8 Hz, 2H), 7.13 (d, *J* = 8.8 Hz, 2H), 4.80 (d, *J* = 2.4 Hz, 2H), 4.59 (d, *J* = 7.5 Hz, 1H), 3.62 – 3.47 (m, 1H), 2.60 (t, *J* = 2.4 Hz, 1H), 1.16 (d, *J* = 6.5 Hz, 6H). ^{13}C NMR (126 MHz, Chloroform-*d*) δ 158.10, 144.93, 139.34, 132.79, 128.58, 127.68, 127.28, 115.62, 78.43, 75.95, 56.05, 46.29, 23.97. ESI-MS (MeOH) m/z $C_{18}H_{19}NO_3S$ $[M + H]^+$ calcd. for:330.12, found:330.14.

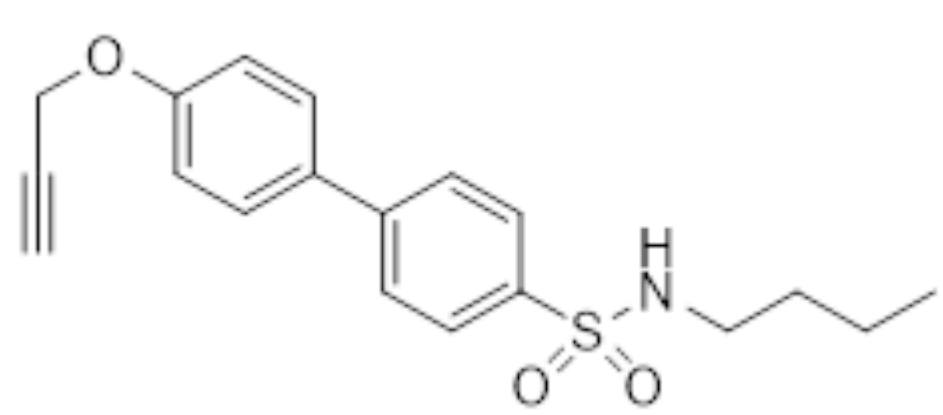

Fig. C23 The chemical structure of compound **4c-4**.

N-butyl-4'-(prop-2-yn-1-yloxy)-[1,1'-biphenyl]-4-sulfonamide (**4c-4**). white solid, yield 29%. m.p.56.8~59.2□. ^{1}H NMR (500 MHz, Chloroform-*d*) δ 7.98 – 7.92 (m, 2H), 7.72 (d, *J* = 8.4 Hz, 2H), 7.64 – 7.59 (m, 2H), 7.13 (d, *J* = 8.7 Hz, 2H), 4.80 (d, *J* = 2.4 Hz, 2H), 4.60 (t, *J* = 6.1 Hz, 1H), 3.04 (q, *J* = 6.8 Hz, 2H), 2.60 (t, *J* = 2.4 Hz, 1H), 1.51 (p, *J* = 7.2 Hz, 2H), 1.36 (h, *J* = 7.4 Hz, 2H), 0.91 (t, *J* = 7.3 Hz, 3H). ^{13}C NMR (126 MHz, Chloroform-*d*) δ 158.12, 145.07, 138.22, 132.79, 128.59, 127.75, 127.33, 115.63, 78.43, 75.96, 56.05, 43.13, 31.80, 19.84, 13.66. ESI-MS (MeOH) m/z $C_{19}H_{21}NO_3S$ $[M + H]^+$ calcd. for:344.13, found:344.15.

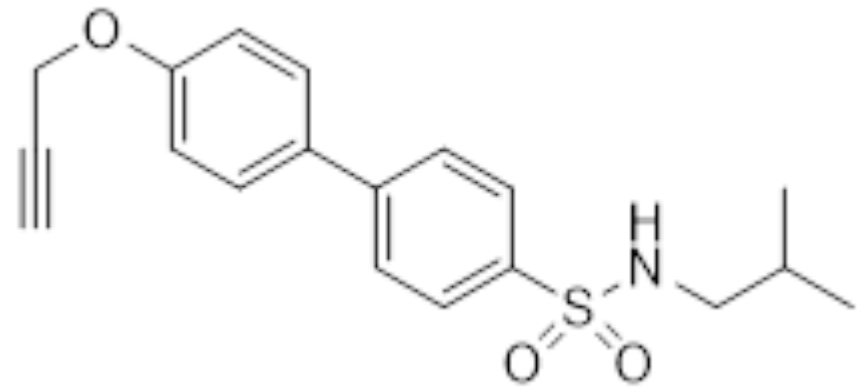

Fig. C24 The chemical structure of compound **4c-5**.

N-isobutyl-4'-(prop-2-yn-1-yloxy)-[1,1'-biphenyl]-4-sulfonamide (**4c-5**). white solid, yield 29%. m.p.66.4~66.9□. ^{1}H NMR (500 MHz, Chloroform-*d*) δ 7.95 (d, *J* = 8.4 Hz, 2H), 7.72 (d, *J* = 8.5 Hz, 2H), 7.61 (d, *J* = 8.8 Hz, 2H), 7.13 (d, *J* = 8.8 Hz, 2H), 4.80 (d, *J* = 2.4 Hz, 2H), 4.73 (t, *J* = 6.5 Hz, 1H), 2.85 (t, *J* = 6.6 Hz, 2H), 2.60 (t, *J* = 2.4 Hz, 1H), 1.79 (dp, *J* = 13.4, 6.7 Hz, 1H), 0.94 (d, *J* = 6.7 Hz, 6H). ^{13}C NMR (126 MHz, Chloroform-*d*) δ 158.12, 145.03, 138.30, 132.78, 128.59, 127.72, 127.33, 115.63, 78.43, 75.96, 56.05, 50.77, 28.65, 20.01. ESI-MS (MeOH) m/z $C_{19}H_{21}NO_3S$ $[M + H]^+$ calcd. for:344.13, found:344.17.

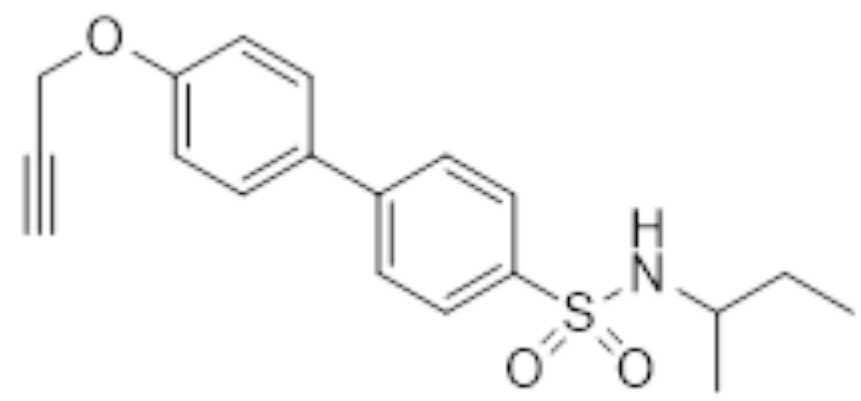

Fig. C25 The chemical structure of compound **4c-6**.

N-(sec-butyl)-4'-(prop-2-yn-1-yloxy)-[1,1'-biphenyl]-4-sulfonamide (**4c-6**). white solid, yield 27%. m.p.74.2~77.4□. ^{1}H NMR (500 MHz, Chloroform-*d*) δ 7.97 (d, *J* = 8.5 Hz, 2H), 7.72 (d, *J* = 8.5 Hz, 2H), 7.62 (d, *J* = 8.7 Hz, 2H), 7.13 (d, *J* = 8.8 Hz, 2H), 4.80 (d, *J* = 2.4 Hz, 2H), 4.64 – 4.49 (m, 1H), 3.34 (dp, *J* = 7.9, 6.5 Hz, 1H), 2.60 (t, *J* = 2.4 Hz, 1H), 1.53 – 1.40 (m, 2H), 1.10 (d, *J* = 6.6 Hz, 3H), 0.87 (t, *J* = 7.4 Hz, 3H). ^{13}C NMR (126 MHz, Chloroform-*d*) δ 158.06, 144.83, 139.45, 132.76, 128.56, 127.66, 127.21, 115.60, 78.41, 75.95, 56.03, 51.61, 30.44, 21.36, 21.35, 10.06. ESI-MS (MeOH) m/z $C_{19}H_{21}NO_3S$ $[M + H]^+$ calcd. for:344.13, found:344.18.

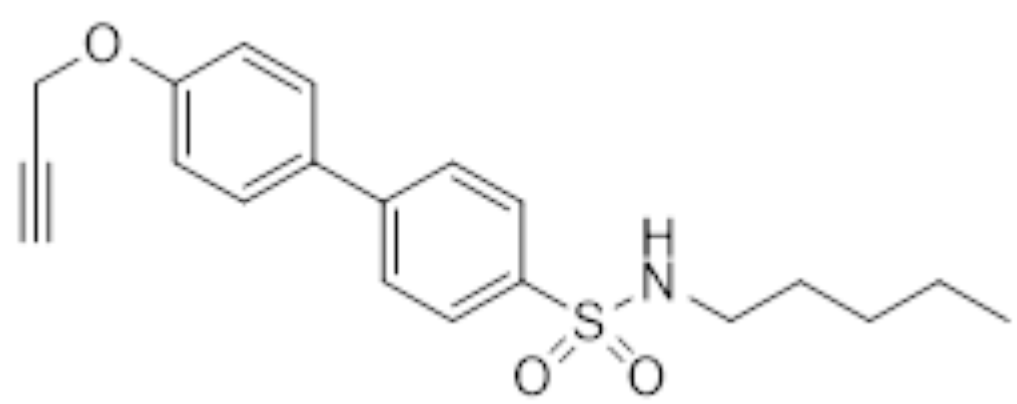

Fig. C26 The chemical structure of compound **4c-7**.

N-pentyl-4'-(prop-2-yn-1-yloxy)-[1,1'-biphenyl]-4-sulfonamide (**4c-7**). white solid, yield 28%. m.p.55.7~58.4□. ^{1}H NMR (500 MHz, Chloroform-*d*) δ 7.95 (d, *J* = 8.5 Hz, 2H), 7.72 (d, *J* = 8.5 Hz, 2H), 7.61 (d, *J* = 8.7 Hz, 2H), 7.13 (d, *J* = 8.8 Hz, 2H), 4.80 (d, *J* = 2.4 Hz, 2H), 4.52 (t, *J* = 6.2 Hz, 1H), 3.03 (q, *J* = 6.9 Hz, 2H), 2.60 (t, *J* = 2.4 Hz, 1H), 1.59 – 1.44 (m, 2H), 1.36 – 1.24 (m, *J* = 2.3 Hz, 5H), 0.96 – 0.82 (m, 3H). ^{13}C NMR (126 MHz, Chloroform-*d*) δ 158.08, 145.07, 138.15, 132.77, 128.59, 127.75, 127.34, 115.59, 78.40, 75.98, 56.01, 43.41, 29.44, 28.78, 22.28, 14.02. ESI-MS (MeOH) m/z $C_{20}H_{23}NO_3S$ $[M + H]^+$ calcd. for:358.12, found:358.19.

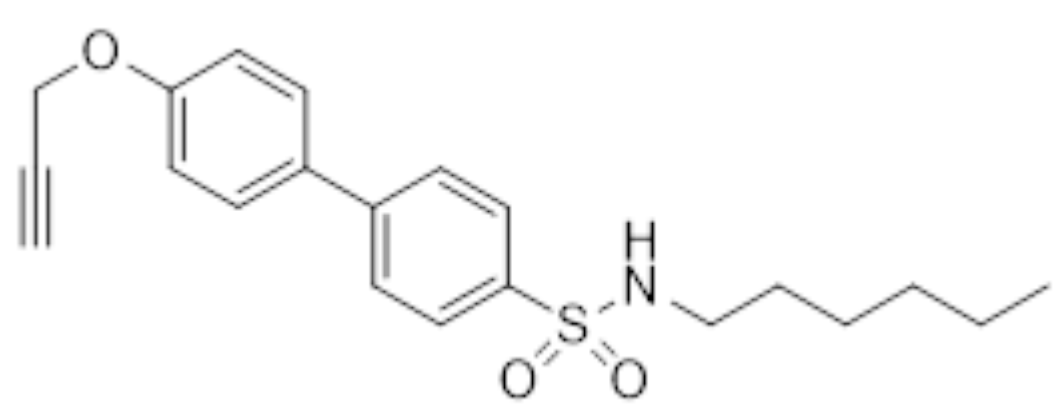

Fig. C27 The chemical structure of compound **4c-8**.

N-hexyl-4'-(prop-2-yn-1-yloxy)-[1,1'-biphenyl]-4-sulfonamide (**4c-8**). white solid, yield 26%. m.p.63.1~63.9□. ^{1}H NMR (400 MHz, Chloroform-*d*) δ 7.90 (d, *J* = 8.4 Hz, 2H), 7.68 (d, *J* = 8.4 Hz, 2H), 7.57 (d, *J* = 8.7 Hz, 2H), 7.09 (d, *J* = 8.7 Hz, 2H), 4.76 (d, *J* = 2.4 Hz, 2H), 4.61 (t, *J* = 6.2 Hz, 1H), 2.98 (q, *J* = 6.8 Hz, 2H), 2.56 (t, *J* = 2.4 Hz, 1H), 1.47 (p, *J* = 7.0 Hz, 2H), 1.31 – 1.15 (m, 6H), 0.84 (t, *J* = 6.8 Hz, 3H).^{13}C NMR (101 MHz, Chloroform-*d*) δ 158.05, 145.03, 138.11, 132.74, 128.57, 127.74, 127.32, 115.56, 78.38, 75.98, 55.99, 43.42, 31.36, 29.68, 26.31, 22.58, 14.08. ESI-MS (MeOH) m/z $C_{21}H_{25}NO_3S$ $[M + H]^+$ calcd. for:372.16, found:372.23.

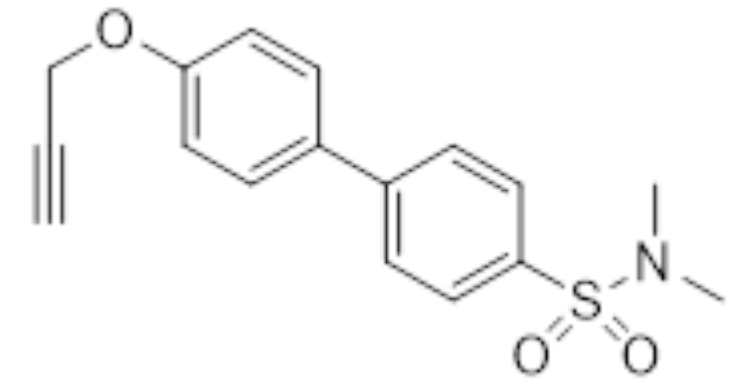

Fig. C28 The chemical structure of compound **4c-9**.

N,N-dimethyl-4'-(prop-2-yn-1-yloxy)-[1,1'-biphenyl]-4-sulfonamide (**4c-9**). white solid, yield 27%. m.p.95.1~96.6□. ^{1}H NMR (500 MHz, Chloroform-*d*) δ 7.86 (d, *J* = 8.5 Hz, 2H), 7.75 (d, *J* = 8.4 Hz, 2H), 7.62 (d, *J* = 8.8 Hz, 2H), 7.14 (d, *J* = 8.8 Hz, 2H), 4.80 (d, *J* = 2.4 Hz, 2H), 2.79 (s, 6H), 2.61 (t, *J* = 2.4 Hz, 1H). ^{13}C NMR (126 MHz, Chloroform-*d*) δ 158.11, 145.14, 135.65, 132.70, 128.60, 128.44, 127.24, 115.60, 78.39, 75.98, 56.01, 38.13. ESI-MS (MeOH) m/z $C_{17}H_{17}NO_3S$ $[M + H]^+$ calcd. for:316.10, found:316.13.

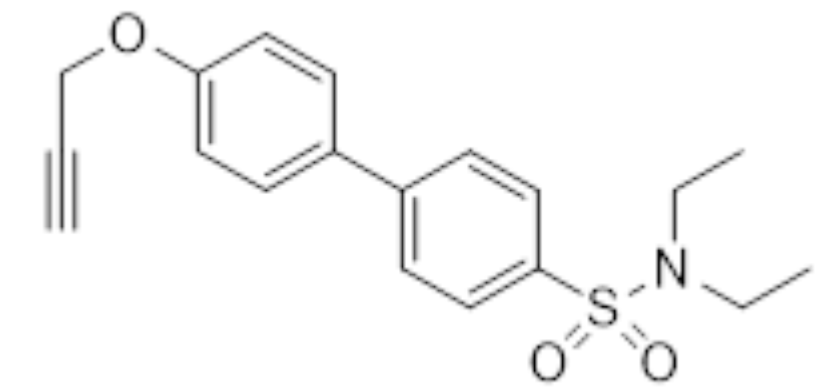

Fig. C29 The chemical structure of compound **4c-10**.

N,N-diethyl-4'-(prop-2-yn-1-yloxy)-[1,1'-biphenyl]-4-sulfonamide (**4c-10**). white solid, yield 27%. m.p.88.4~89.7□. ^{1}H NMR (500 MHz, Chloroform-*d*) δ 7.89 (d, *J* = 8.4 Hz, 2H), 7.70 (d, *J* = 8.5 Hz, 2H), 7.61 (d, *J* = 8.7 Hz, 2H), 7.12 (d, *J* = 8.8 Hz, 2H), 4.80 (d, *J* = 2.4 Hz, 2H), 3.31 (q, *J* = 7.2 Hz, 5H), 2.60 (t, *J* = 2.4 Hz, 1H), 1.20 (t, *J* = 7.2 Hz, 7H). ^{13}C NMR (126 MHz, Chloroform-*d*) δ 158.00, 144.64, 138.54, 132.78, 128.53, 127.66, 127.21, 115.54, 78.40, 75.95, 55.99, 42.22, 14.34. ESI-MS (MeOH) m/z $C_{19}H_{21}NO_3S$ $[M + H]^+$ calcd. for:344.13, found:344.17.

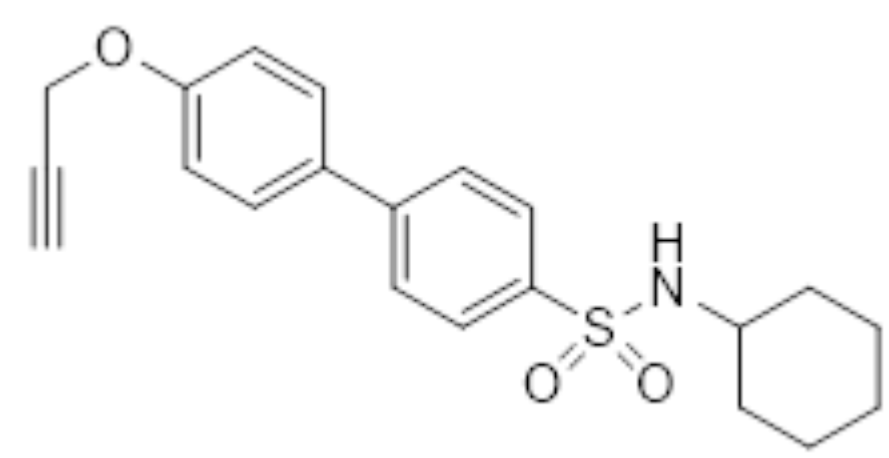

Fig. C30 The chemical structure of compound **4c-11**.

N-cyclohexyl-4'-(prop-2-yn-1-yloxy)-[1,1'-biphenyl]-4-sulfonamide (**4c-11**). white solid, yield 29%. m.p.55.7~59.2□. ^{1}H NMR (400 MHz, Chloroform-*d*) δ 7.93 (d, *J* = 8.1 Hz, 2H), 7.67 (d, *J* = 8.0 Hz, 2H), 7.62 – 7.50 (m, 2H), 7.08 (d, *J* = 8.3 Hz, 2H), 4.87 – 4.77 (m, 1H), 4.75 (d, *J* = 1.7 Hz, 2H), 3.18 (dp, *J* = 14.1, 4.3 Hz, 1H), 2.56 (d, *J* = 2.6 Hz, 1H), 1.84 – 1.46 (m, 5H), 1.18 (dtd, *J* = 25.4, 14.0, 6.4 Hz, 5H). ^{13}C NMR (101 MHz, Chloroform-*d*) δ 158.00, 144.75, 139.58, 132.71, 128.54, 127.55, 127.22, 115.53, 78.38, 75.97, 55.97, 52.78, 34.05, 25.24, 24.75. ESI-MS (MeOH) m/z $C_{21}H_{23}NO_3S$ $[M + H]^+$ calcd. for:370.12, found:370.20.

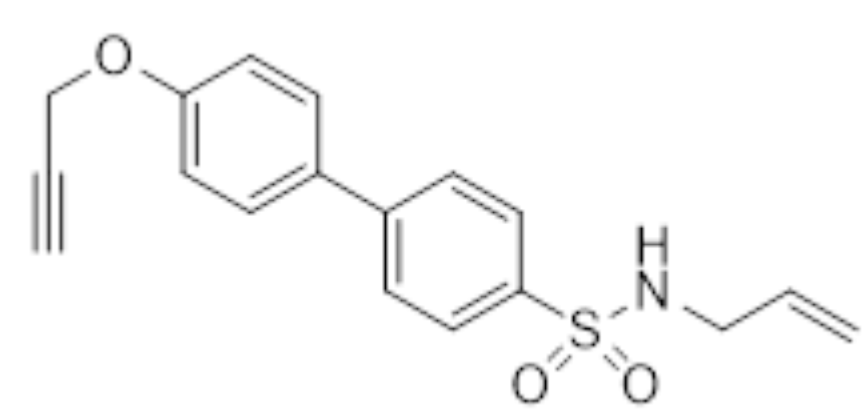

Fig. C31 The chemical structure of compound **4c-12**.

N-allyl-4'-(prop-2-yn-1-yloxy)-[1,1'-biphenyl]-4-sulfonamide (**4c-12**). white solid, yield 17%. m.p.114.5~117.7□. ^{1}H NMR (400 MHz, Chloroform-*d*) δ 7.95 – 7.87 (m, 2H), 7.70 – 7.67 (m, 2H), 7.61 – 7.53 (m, 2H), 7.13 – 7.02 (m, 2H), 5.75 (ddt, *J* = 16.4, 11.0, 5.9 Hz, 1H), 5.23 – 5.07 (m, 2H), 4.76 (d, *J* = 2.4 Hz, 2H), 4.66 (t, *J* = 6.3 Hz, 1H), 3.64 (t, *J* = 6.1 Hz, 2H), 2.56 (d, *J* = 2.4 Hz, 1H). ^{13}C NMR (101 MHz, Chloroform-*d*) δ 158.07, 145.19, 137.99, 133.05, 132.64, 128.59, 127.79, 127.36, 117.98, 115.57, 78.37, 76.00, 55.99, 45.96. ESI-MS (MeOH) m/z $C_{18}H_{17}NO_3S$ $[M + H]^+$ calcd. for:328.10, found:328.15.

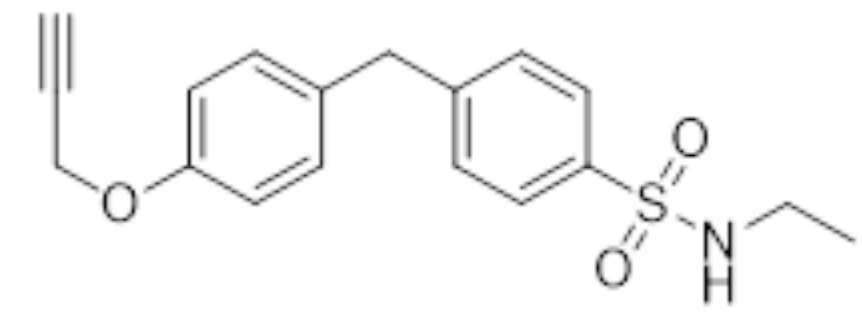

Fig. C32 The chemical structure of compound **6-1**.

N-ethyl-4-(4-(prop-2-yn-1-yloxy)benzyl)benzenesulfonamide (**6-1**) . white solid, yield 20%. m.p.46.8~47.2□. ^{1}H NMR (400 MHz, Chloroform-*d*) δ 7.77 (d, *J* = 8.4 Hz, 2H), 7.30 (d, *J* = 8.2 Hz, 2H), 7.10 (d, *J* = 8.6 Hz, 2H), 6.92 (d, *J* = 8.7 Hz, 2H), 4.67 (d, *J* = 2.4 Hz, 2H), 4.59 (t, *J* = 6.1 Hz, 1H), 3.98 (s, 2H), 2.99 (qd, *J* = 7.2, 6.0 Hz, 2H), 2.52 (t, *J* = 2.4 Hz, 1H), 1.09 (t, *J* = 7.2 Hz, 3H). ^{13}C NMR (101 MHz, Chloroform-*d*) δ 156.28, 146.69, 137.61, 132.71, 130.00, 129.47, 127.32, 115.12, 78.58, 75.58, 55.86, 40.86, 38.26, 15.08. ESI-MS (MeOH) m/z $C_{18}H_{19}NO_3S$ $[M + K]^+$ calcd. for:368.07, found:368.50.

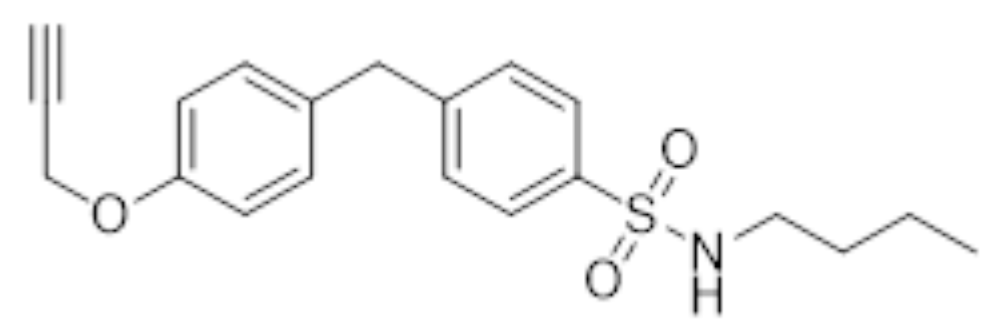

Fig. C33 The chemical structure of compound **6-2**.

N-butyl-4-(4-(prop-2-yn-1-yloxy)benzyl)benzenesulfonamide (**6-2**) . white solid, yield 23%. m.p.48.7~49.5□. ^{1}H NMR (400 MHz, Chloroform-*d*) δ 7.77 (d, *J* = 8.3 Hz, 2H), 7.30 (d, *J* = 8.0 Hz, 2H), 7.10 (d, *J* = 8.6 Hz, 2H), 6.92 (d, *J* = 8.6 Hz, 2H), 4.67 (d, *J* = 2.4 Hz, 2H), 4.51 (t, *J* = 6.2 Hz, 1H), 3.99 (s, 2H), 2.93 (q, *J* = 6.8 Hz, 2H), 2.51 (t, *J* = 2.4 Hz, 1H), 1.43 (m, 2H), 1.33 – 1.26 (m, 2H), 0.84 (t, *J* = 7.3 Hz, 3H).^{13}C NMR (101 MHz, Chloroform-*d*) δ 156.29, 146.63, 137.69, 132.72, 129.98, 129.46, 127.29, 115.13, 75.54, 55.87, 42.95, 40.86, 31.62, 19.68, 13.54. ESI-MS (MeOH) m/z $C_{20}H_{23}NO_3S$ $[M + H]^+$ calcd. for:358.12, found:358.19.

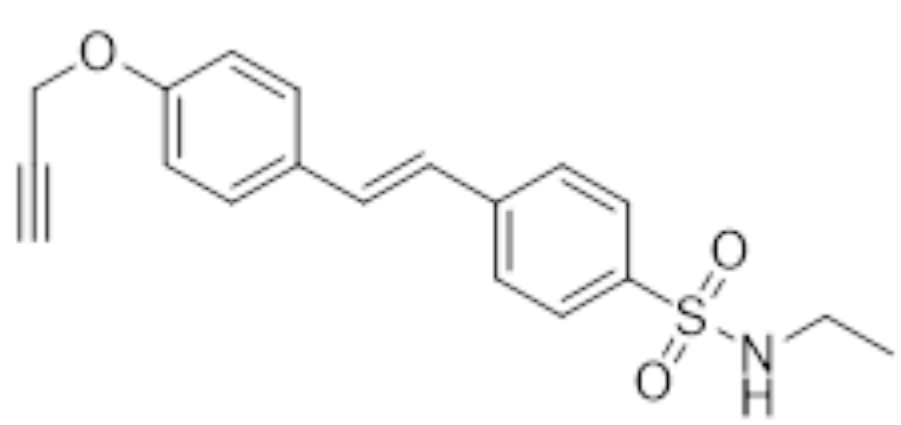

Fig. C34 The chemical structure of compound **8-1**.

(E)-*N*-ethyl-4-(4-(prop-2-yn-1-yloxy)styryl)benzenesulfonamide (**8-1**). white solid, yield 25%. m.p.89.6~93.7□. ^{1}H NMR (400 MHz, Chloroform-*d*) δ 7.71 (d, *J* = 8.4 Hz, 2H), 7.38 (d, *J* = 8.2 Hz, 2H), 7.15 (d, *J* = 8.6 Hz, 2H), 6.85 (d, *J* = 8.8 Hz, 2H), 6.67 (d, *J* = 12.2 Hz, 1H), 6.51 (d, *J* = 12.2 Hz, 1H), 4.68 (d, *J* = 2.4 Hz, 2H), 4.34 (t, *J* = 6.3 Hz, 1H), 3.03 (qd, *J* = 7.2, 6.1 Hz, 2H), 2.53 (t, *J* = 2.4 Hz, 1H), 1.11 (t, *J* = 7.2 Hz, 3H). ^{13}C NMR (101 MHz, Chloroform-*d*) δ 157.96, 142.13, 138.03, 131.51, 130.23, 128.30, 127.70, 126.76, 125.21, 115.38, 78.42, 75.92, 55.99, 38.42, 15.28. ESI-MS (MeOH) m/z $C_{19}H_{19}NO_3S$ $[M + H]^+$ calcd. for:342.12, found:342.10.

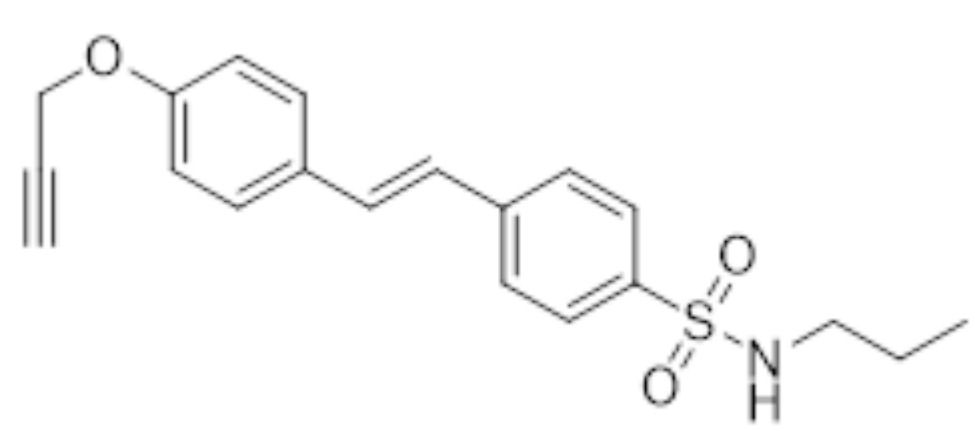

Fig. C35 The chemical structure of compound **8-2**.

(E)-4-(4-(prop-2-yn-1-yloxy)styryl)-*N*-propylbenzenesulfonamide (**8-2**). white solid, yield 22%. m.p.84.7~87.2□. ^{1}H NMR (400 MHz, Chloroform-*d*) δ 7.71 (d, *J* = 8.4 Hz, 2H), 7.38 (d, *J* = 8.5 Hz, 2H), 7.15 (d, *J* = 8.7 Hz, 2H), 6.84 (d, *J* = 8.7 Hz, 2H), 6.67 (d, *J* = 12.1 Hz, 1H), 6.51 (d, *J* = 12.2 Hz, 1H), 4.68 (d, *J* = 2.3 Hz, 2H), 4.39 (t, *J* = 6.2 Hz, 1H), 2.94 (q, *J* = 6.8 Hz, 2H), 2.53 (t, *J* = 2.4 Hz, 1H), 1.49 (h, *J* = 7.2 Hz, 2H), 0.87 (t, *J* = 7.4 Hz, 3H). ^{13}C NMR (101 MHz, Chloroform-*d*) δ 157.95, 142.08, 138.09, 131.49, 130.24, 128.29, 127.67, 126.75, 125.23, 115.38, 78.42, 75.92, 55.99, 45.13, 23.12, 11.26. ESI-MS (MeOH) m/z $C_{20}H_{21}NO_3S$ $[M + H]^+$ calcd. for:356.13, found:356.12.

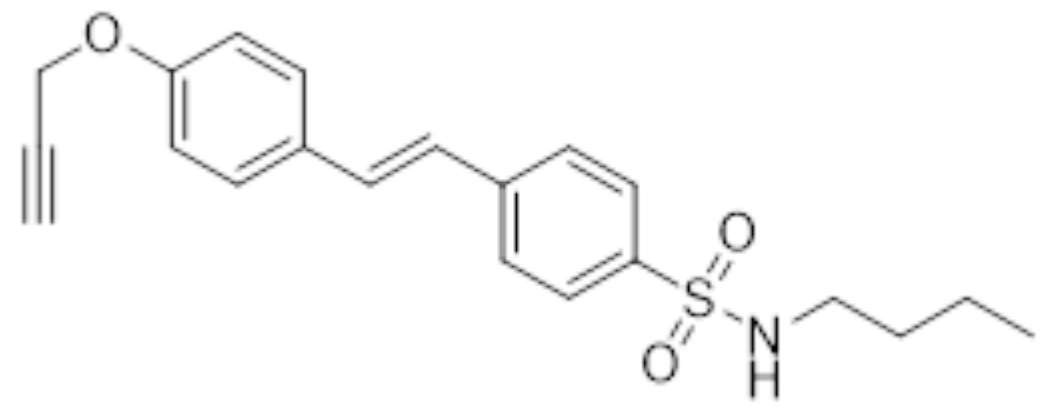

Fig. C36 The chemical structure of compound **8-3**.

(E)-*N*-butyl-4-(4-(prop-2-yn-1-yloxy)styryl)benzenesulfonamide (**8-3**). white solid, yield 21%. m.p.97.4~99.6□. ^{1}H NMR (400 MHz, Chloroform-*d*) δ 7.71 (d, *J* = 8.0 Hz, 2H), 7.38 (d, *J* = 7.9 Hz, 2H), 7.15 (d, *J* = 8.2 Hz, 2H), 6.93 – 6.77 (m, 2H), 6.67 (d, *J* = 12.2 Hz, 1H), 6.51 (d, *J* = 12.2 Hz, 1H), 4.68 (d, *J* = 2.4 Hz, 2H), 4.37 (t, *J* = 6.3 Hz, 1H), 2.96 (q, *J* = 6.6 Hz, 2H), 2.53 (d, *J* = 2.9 Hz, 1H), 1.44 (p, *J* = 7.1 Hz, 2H), 1.31 (q, *J* = 7.2 Hz, 2H), 0.86 (t, *J* = 7.3 Hz, 3H).^{13}C NMR (101 MHz, Chloroform-*d*) δ 157.95, 142.08, 138.02, 131.48, 130.24, 128.29, 127.67, 126.75, 125.23, 115.37, 78.42, 75.92, 55.99, 43.10, 31.76, 19.84, 13.68. ESI-MS (MeOH) m/z $C_{21}H_{23}NO_3S$ [M + H]$^{+}$ calcd. for:370.12, found:370.15.

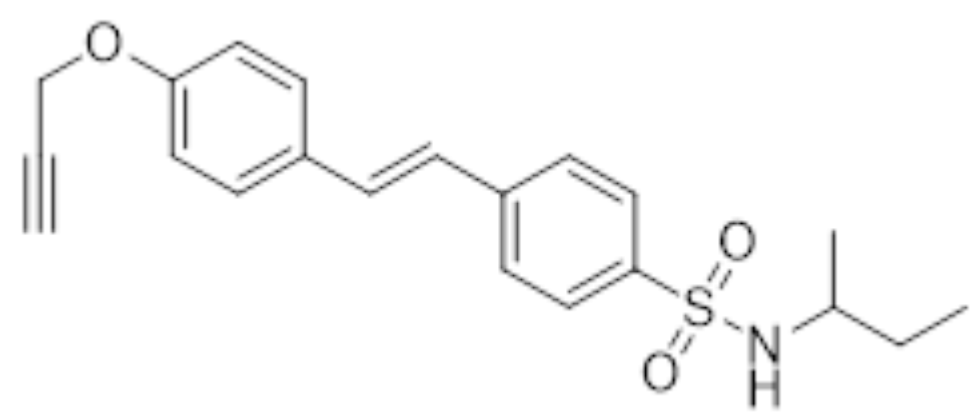

Fig. C37 The chemical structure of compound **8-4**.

(E)-*N*-(sec-butyl)-4-(4-(prop-2-yn-1-yloxy)styryl)benzenesulfonamide (**8-4**). white solid, yield 19%. m.p.90.6~93.2□. ^{1}H NMR (400 MHz, Chloroform-*d*) δ 7.73 (d, *J* = 8.4 Hz, 2H), 7.37 (d, *J* = 8.3 Hz, 2H), 7.13 (d, *J* = 8.6 Hz, 2H), 6.83 (d, *J* = 8.7 Hz, 2H), 6.66 (d, *J* = 12.2 Hz, 1H), 6.53 (d, *J* = 12.2 Hz, 1H), 4.68 (d, *J* = 2.4 Hz, 2H), 4.18 (d, *J* = 8.3 Hz, 1H), 3.27 (dq, *J* = 8.3, 6.5 Hz, 1H), 2.53 (t, *J* = 2.4 Hz, 1H), 1.41 – 1.34 (m, 2H), 1.04 (d, *J* = 6.5 Hz, 4H), 0.79 (d, *J* = 7.5 Hz, 3H). ^{13}C NMR (101 MHz, Chloroform-*d*) δ 157.96, 142.07, 138.13, 131.49, 130.25, 128.29, 127.65, 126.76, 125.24, 115.38, 78.44, 75.93, 55.99, 51.69, 30.42, 21.28, 10.24. ESI-MS (MeOH) m/z $C_{21}H_{23}NO_3S$ [M + H]$^{+}$ calcd. for:370.12, found:370.16.

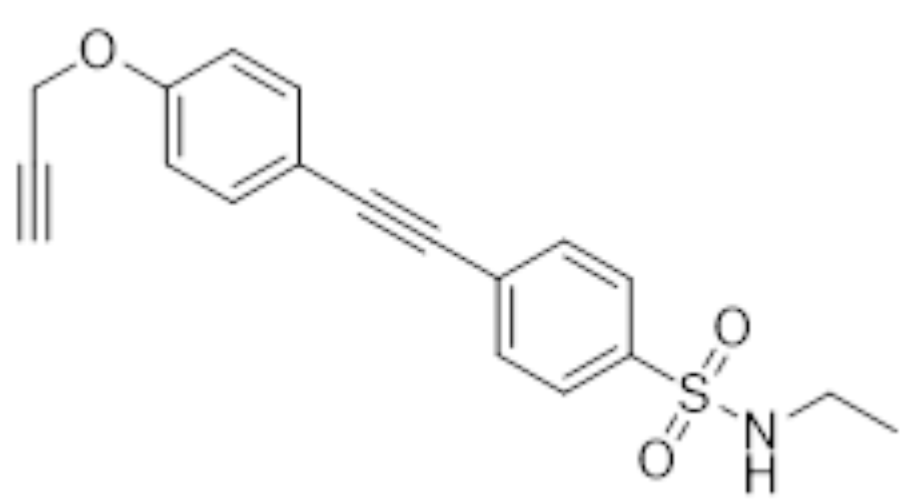

Fig. C38 The chemical structure of compound **10-1**.

N-ethyl-4-((4-(prop-2-yn-1-yloxy)phenyl)ethynyl)benzenesulfonamide (**10-1**). white solid, yield 21%. m.p.59.7~62.5□. ^{1}H NMR (500 MHz, Chloroform-*d*) δ 7.88 (d, *J* = 8.4 Hz, 2H), 7.67 (d, *J* = 8.5 Hz, 2H), 7.54 (d, *J* = 8.8 Hz, 2H), 7.02 (d, *J* = 8.8 Hz, 2H), 4.77 (d, *J* = 2.5 Hz, 2H), 4.53 (t, *J* = 6.0 Hz, 1H), 3.08 (qd, *J* = 7.3, 6.0 Hz, 2H), 2.60 (t, *J* = 2.4 Hz, 1H), 1.16 (t, *J* = 7.3 Hz, 3H). ^{13}C NMR (126 MHz, Chloroform-*d*) δ 158.07, 138.91, 133.33, 131.95, 128.29, 127.09, 115.43, 115.11, 92.79, 87.00, 78.03,76.01, 55.87, 38.33, 15.17. ESI-MS (MeOH) m/z $C_{19}H_{17}NO_3S$ [M + H]$^{+}$ calcd. for:340.10, found:340.16.

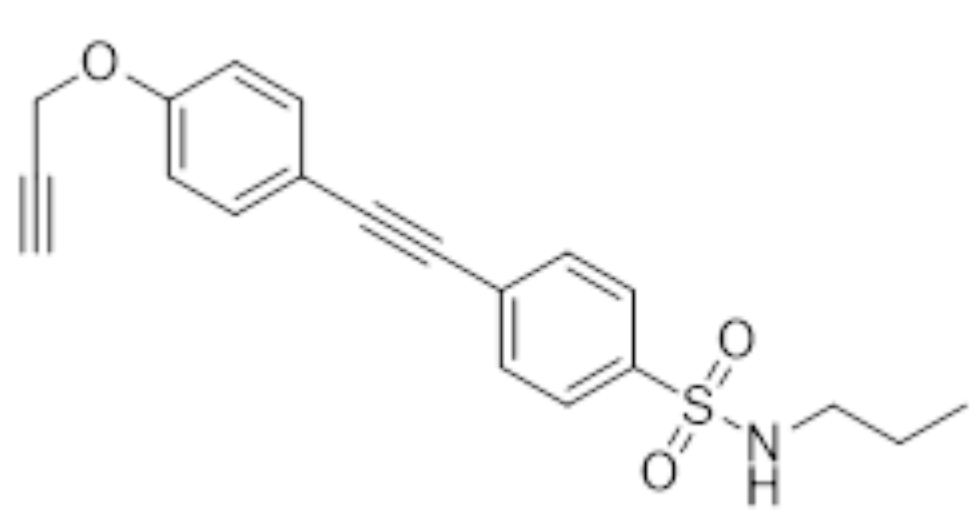

Fig. C39 The chemical structure of compound **10-2**.

4-((4-(prop-2-yn-1-yloxy)phenyl)ethynyl)-*N*-propylbenzenesulfonamide (**10-2**). white solid, yield 18%. m.p.66.3~67.6□. ^{1}H NMR (400 MHz, Chloroform-*d*) δ 7.84 (d, *J* = 8.6 Hz, 2H), 7.62 (d, *J* = 8.5 Hz, 2H), 7.50 (d, *J* = 8.8 Hz, 2H), 6.98 (d, *J* = 8.9 Hz, 2H), 4.73 (d, *J* = 2.5 Hz, 2H), 4.40 (d, *J* = 7.7 Hz, 1H), 3.56 – 3.44 (m, 1H), 2.55 (t, *J* = 2.4 Hz, 1H), 1.09 (d, *J* = 6.5 Hz, 6H). ^{13}C NMR (101 MHz, Chloroform-*d*) δ 158.03, 140.01, 133.31, 131.92, 128.11, 126.98, 115.42, 115.08, 92.69, 87.01,78.03, 76.00, 55.85, 46.23, 23.82. ESI-MS (MeOH) m/z $C_{20}H_{19}NO_3S$ [M + H]$^{+}$ calcd. for:354.12, found:354.12.

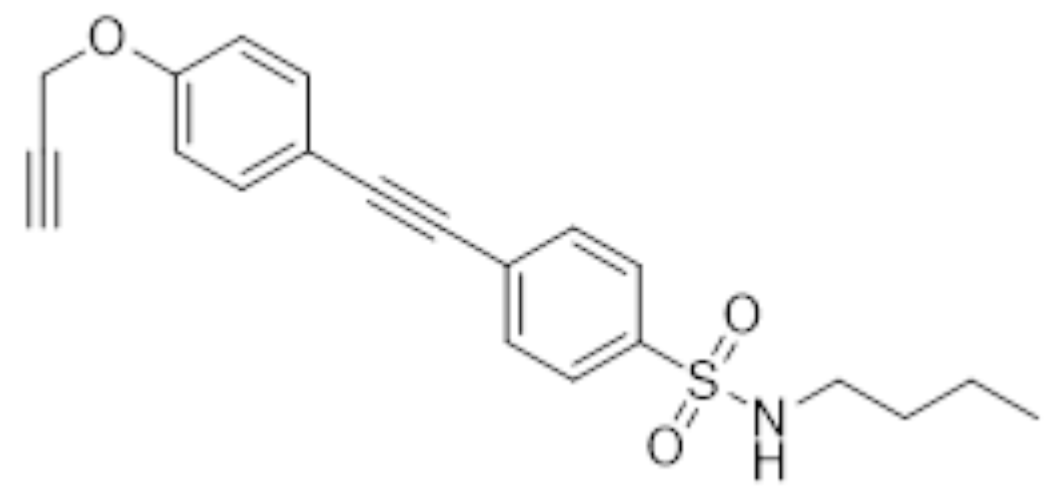

Fig. C40 The chemical structure of compound **10-3**.

N-butyl-4-((4-(prop-2-yn-1-yloxy)phenyl)ethynyl)benzenesulfonamide (**10-3**). white solid, yield 17%. m.p.70.8~72.4□. ^{1}H NMR (400 MHz, Chloroform-*d*) δ 7.82 (d, *J* = 8.0 Hz, 2H), 7.62 (d, *J* = 8.0 Hz, 2H), 7.50 (d, *J* = 8.4 Hz, 2H), 6.98 (d, *J* = 8.4 Hz, 2H), 4.73 (d, *J* = 1.8 Hz, 2H), 4.39 (t, *J* = 6.2 Hz, 1H), 2.97 (q, *J* = 6.7 Hz, 2H), 2.59 – 2.52 (m, 1H), 1.45 (p, *J* = 7.2 Hz, 2H), 1.31 (dt, *J* = 15.0, 7.2 Hz, 2H), 0.86 (t, *J* = 7.3 Hz, 4H). ^{13}C NMR (101 MHz, Chloroform-*d*) δ 158.05, 138.86, 133.31, 131.92, 128.25, 127.05, 115.42, 115.09, 92.76, 86.99, 78.03,75.99, 55.85, 42.99, 31.64, 19.69, 13.53. ESI-MS (MeOH) m/z $C_{21}H_{21}NO_3S$ $[M + H]^+$ calcd. for:368.13, found:368.47.

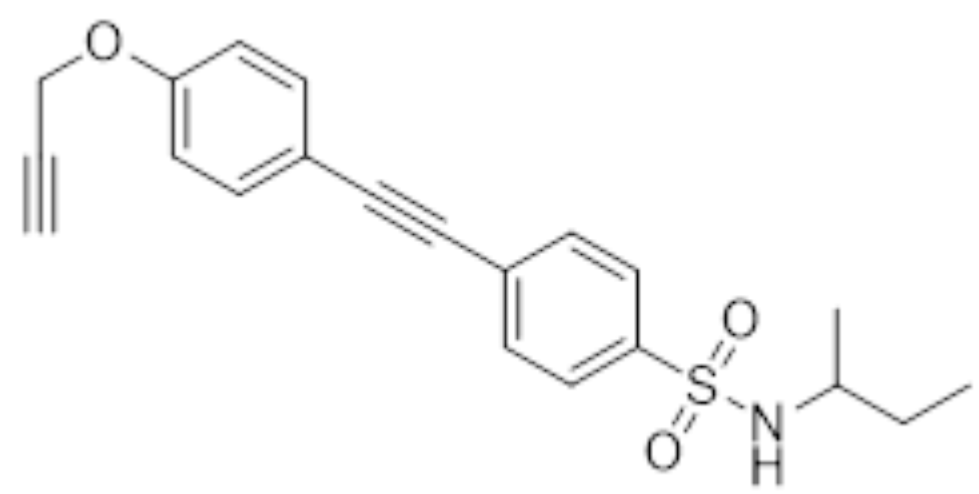

Fig. C41 The chemical structure of compound **10-4**.

N-(sec-butyl)-4-((4-(prop-2-yn-1-yloxy)phenyl)ethynyl)benzenesulfonamide (**10-4**). white solid, yield 20%. m.p.71.7~74.3□. ^{1}H NMR (400 MHz, Chloroform-*d*) δ 7.85 (d, *J* = 8.5 Hz, 2H), 7.61 (d, *J* = 8.5 Hz, 2H), 7.49 (d, *J* = 8.8 Hz, 2H), 6.97 (d, *J* = 8.8 Hz, 2H), 4.72 (d, *J* = 2.4 Hz, 2H), 4.55 (d, *J* = 8.2 Hz, 1H), 2.55 (t, *J* = 2.4 Hz, 1H), 1.40 (p, *J* = 7.3 Hz, 2H), 1.03 (d, *J* = 6.5 Hz, 3H), 0.90 – 0.84 (m, 1H), 0.80 (t, *J* = 7.4 Hz, 3H).^{13}C NMR (101 MHz, Chloroform-*d*) δ 158.15, 140.30, 133.40, 131.98, 128.13, 127.08, 115.55, 115.20, 92.75, 87.17, 78.15, 76.10, 55.96, 51.68, 30.39, 21.36, 10.07. ESI-MS (MeOH) m/z $C_{21}H_{21}NO_3S$ $[M + H]^+$ calcd. for:368.13, found:368.14.

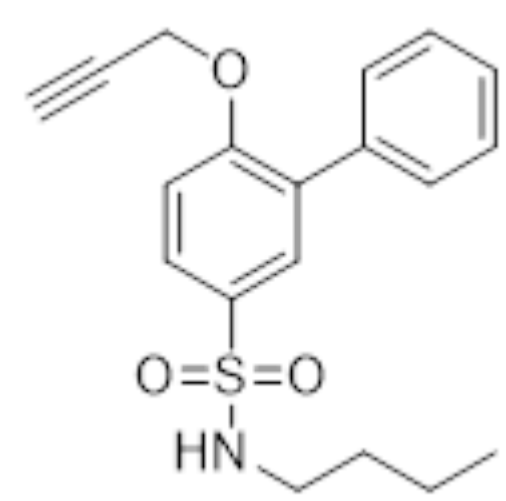

Fig. C42 The chemical structure of compound **S4-1**.

N-butyl-6-(prop-2-yn-1-yloxy)-[1,1'-biphenyl]-3-sulfonamide (**S4-1**). white solid, yield 47%. m.p. 55.4~57.2□. ^{1}H NMR (400 MHz, Chloroform-*d*) δ 8.07 (d, *J* = 2.2 Hz, 1H), 7.81 (dd, *J* = 8.7, 2.3 Hz, 1H), 7.45 – 7.39 (m, 2H), 7.32 (qd, *J* = 8.9, 7.5, 3.3 Hz, 3H), 7.24 (d, *J* = 8.8 Hz, 1H), 5.08 (s, 2H), 4.37 (t, *J* = 6.2 Hz, 1H), 2.96 (q, *J* = 6.7 Hz, 2H), 1.46 (p, *J* = 7.1 Hz, 2H), 1.29 (dp, *J* = 14.5, 7.3 Hz, 3H), 0.86 (t, *J* = 7.3 Hz, 3H). ^{13}C NMR (101 MHz, Chloroform-*d*) δ 157.53, 133.75, 132.56, 131.98, 129.25, 128.54, 127.98, 121.78, 113.46, 112.87, 88.82, 82.22, 58.04, 43.10, 31.73, 19.81, 13.66. ESI-MS (MeOH) m/z $C_{19}H_{21}NO_3S$ $[M + H]^+$ calcd. for:344.13, found:344.14.

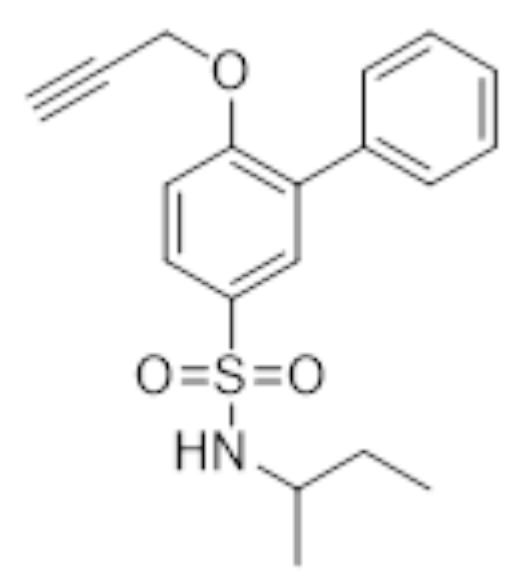

Fig. C43 The chemical structure of compound **S4-2**.

N-(sec-butyl)-6-(prop-2-yn-1-yloxy)-[1,1'-biphenyl]-3-sulfonamide (**S4-2**). white solid, yield 45%. m.p.73.5~76.1□. ^{1}H NMR (400 MHz, CDCl3) δ 8.02 (d, J = 2.3 Hz, 1H), 7.76 (dd, J = 8.7, 2.3 Hz, 1H), 7.37 – 7.31 (m, 1H), 7.30 – 7.19 (m, 1H), 7.15 (d, J = 8.7 Hz, 1H), 5.00 (s, 1H), 4.54 (d, J = 8.1 Hz, 1H), 3.29 – 3.07 (m, 1H), 1.43 – 1.26 (m, 1H), 0.97 (d, J = 6.6 Hz, 1H), 0.73 (t, J = 7.4 Hz, 1H).^{13}C NMR (101 MHz, Chloroform-*d*) δ 157.35, 135.04, 132.47, 131.93, 129.19, 128.50, 127.81, 121.77, 113.46, 112.72, 88.78, 82.23, 58.04, 51.64, 30.35, 21.33, 10.07. ESI-MS (MeOH) m/z $C_{19}H_{21}NO_3S$ $[M + H]^+$ calcd. for:344.13, found:344.15.

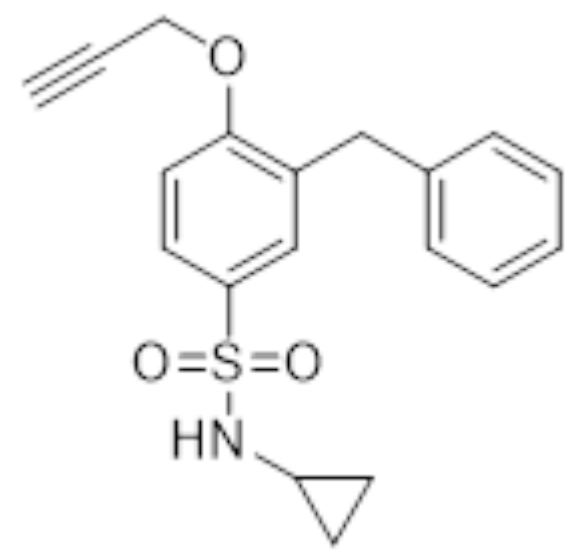

Fig. C44 The chemical structure of compound **S6-1**.

3-benzyl-*N*-cyclopropyl-4-(prop-2-yn-1-yloxy)benzenesulfonamide (**S6-1**). white solid, yield 55%. m.p. 65.1~68.0□. ^{1}H NMR (400 MHz, Chloroform-*d*) δ 7.69 (dd, *J* = 8.6, 2.4 Hz, 1H), 7.56 (d, *J* = 2.4 Hz, 1H), 7.23 – 7.15 (m, 2H), 7.13 (d, *J* = 7.2 Hz, 3H), 6.99 (d, *J* = 8.6 Hz, 1H), 4.79 (s, 1H), 4.69 (d, *J* = 2.4 Hz, 2H), 3.94 (s, 2H), 2.48 (t, *J* = 2.4 Hz, 1H), 2.10 (tt, *J* = 6.8, 3.6 Hz, 1H), 0.45 (ddt, *J* = 11.8, 5.0, 2.3 Hz, 5H). ^{13}C NMR (101 MHz, Chloroform-*d*) δ 158.59, 139.48, 131.89, 131.40, 129.77, 128.95, 128.50, 127.47, 126.31, 111.66, 77.64, 76.34, 56.21, 35.82, 24.32, 6.12. ESI-MS (MeOH) m/z $C_{19}H_{19}NO_3S$ $[M + H]^+$ calcd. for:342.12, found:342.17.

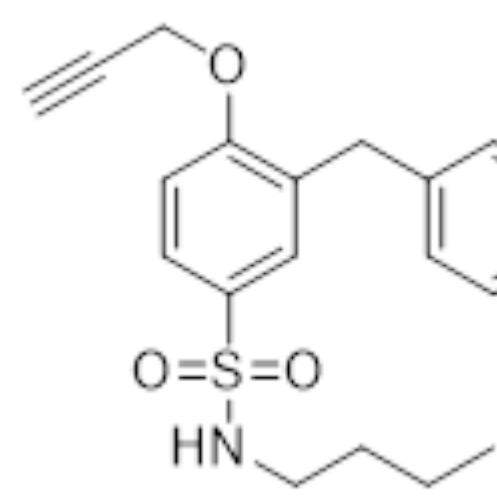

Fig. C45 The chemical structure of compound **S6-2**.

3-benzyl-*N*-butyl-4-(prop-2-yn-1-yloxy)benzenesulfonamide **(S6-2**). white solid, yield 49%. m.p. 72.6~72.9□. ^{1}H NMR (400 MHz, Chloroform-*d*) δ 7.66 (dd, *J* = 8.6, 2.4 Hz, 1H), 7.53 (d, *J* = 2.4 Hz, 1H), 7.22 – 7.16 (m, 2H), 7.12 (d, *J* = 7.2 Hz, 3H), 6.97 (d, *J* = 8.6 Hz, 1H), 4.68 (d, *J* = 2.4 Hz, 2H), 4.41 (t, *J* = 6.3 Hz, 1H), 3.92 (s, 2H), 2.80 (q, *J* = 6.2 Hz, 2H), 2.47 (t, *J* = 2.4 Hz, 1H), 1.31 (dtd, *J* = 8.4, 7.6, 7.1, 6.1 Hz, 2H), 1.23 – 1.12 (m, 2H), 0.75 (t, *J* = 7.3 Hz, 3H). ^{13}C NMR (101 MHz, CDCl3) δ 158.59 (s), 139.62 (s), 132.45 (s), 131.71 (s), 129.54 (s), 129.08 (s), 128.65 (s), 127.34 (s), 126.46 (s), 112.87 – 111.99 (m), 77.82 (s), 76.45 (s), 56.34 (s), 43.06 (s), 36.03 (s), 31.68 (s), 19.85 (s), 13.70 (s).ESI-MS (MeOH) m/z $C_{20}H_{23}NO_3S$ $[M + H]^+$ calcd. for:358.12, found:358.20.

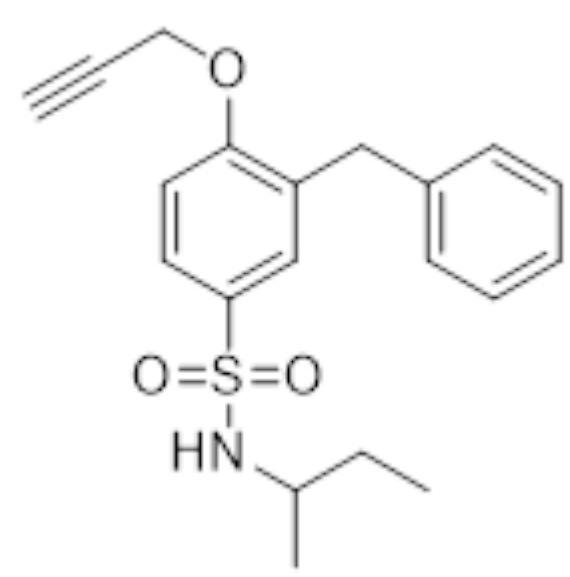

Fig. C46 The chemical structure of compound **S6-3**.

3-benzyl-*N*-(sec-butyl)-4-(prop-2-yn-1-yloxy)benzenesulfonamide (**S6-3**). white solid, yield 45%. m.p. 81.5~82.0□. ^{1}H NMR (400 MHz, Chloroform-*d*) δ 7.77 (dd, *J* = 8.6, 2.4 Hz, 1H), 7.63 (d, *J* = 2.5 Hz, 1H), 7.27 (d, *J* = 7.2 Hz, 2H), 7.24 – 7.17 (m, 3H), 7.06 (d, *J* = 8.7 Hz, 1H), 4.78 (d, *J* = 2.2 Hz, 2H), 4.46 (s, 1H), 4.02 (s, 2H), 3.19 (p, *J* = 6.7 Hz, 1H), 2.57 (t, *J* = 2.4 Hz, 1H), 1.42 – 1.31 (m, 2H), 1.00 (d, *J* = 6.6 Hz, 3H), 0.77 (t, *J* = 7.4 Hz, 3H). ^{13}C NMR (101 MHz, CDCl3) δ 158.32 (s), 139.52 (s), 133.51 (s), 131.40 (s), 129.30 (s), 128.93 (s), 128.48 (s), 127.13 (s), 126.28 (s), 111.58 (s), 77.68 (s), 76.28 (s), 56.21 (s), 51.32 (s), 35.83 (s), 30.22 (s), 21.18 (s), 9.95 (s).ESI-MS (MeOH) m/z $C_{20}H_{23}NO_3S$ $[M + H]^+$ calcd. for:358.12, found:358.20.

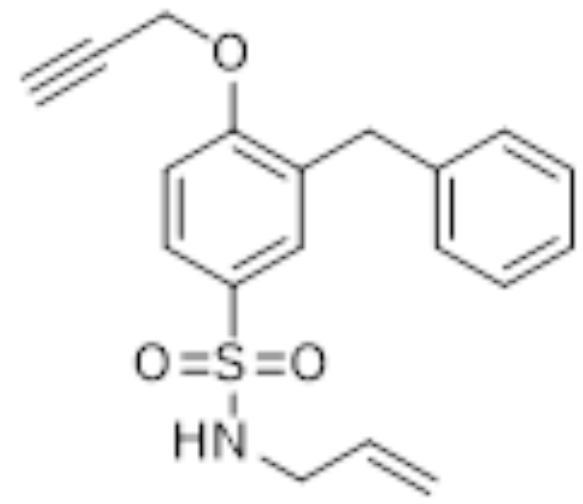

Fig. C47 The chemical structure of compound **S6-4**.

N-allyl-3-benzyl-4-(prop-2-yn-1-yloxy)benzenesulfonamide (**S6-4**). white solid, yield 36%. m.p. 105.2~108..4□. ^{1}H NMR (400 MHz, CDCl3) δ 7.73 (dd, J = 8.6, 2.2 Hz, 1H), 7.60 (d, J = 2.1 Hz, 1H), 7.31 – 7.15 (m, 5H), 7.05 (d, J = 8.7 Hz, 1H), 5.67 (dq, J = 10.6, 5.8 Hz, 1H), 5.08 (dd, J = 24.0, 13.7 Hz, 2H), 4.76 (d, J = 2.2 Hz, 2H), 4.52 (t, J = 6.2 Hz, 1H), 4.00 (s, 2H), 3.53 (t, J = 6.0 Hz, 2H), 2.55 (t, J = 2.2 Hz, 1H).^{13}C NMR (101 MHz, CDCl3) δ 158.55 (s), 139.42 (s), 133.00 (s), 132.20 (s), 131.52 (s), 129.40 (s), 128.95 (s), 128.52 (s), 127.28 (s), 126.34 – 125.64 (m), 117.67 – 117.24 (m), 111.71 (s), 77.64 (s), 76.36 (s), 56.19 (s), 45.77 (s), 35.88 (s).ESI-MS (MeOH) m/z $C_{19}H_{19}NO_3S$ $[M + H]^+$ calcd. for:342.12, found:342.17.

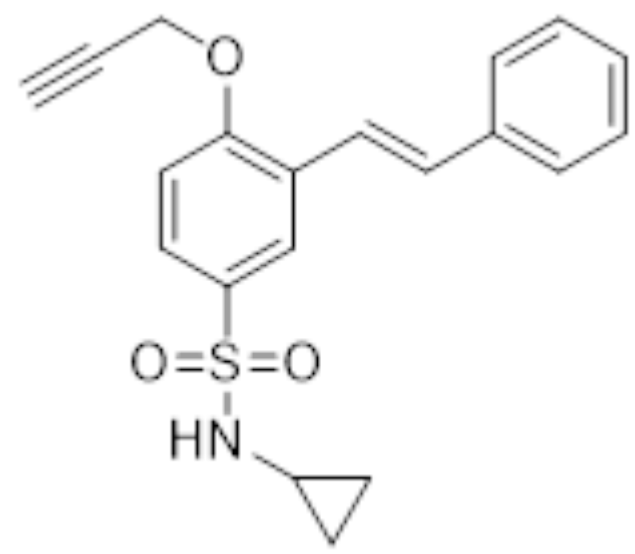

Fig. C48 The chemical structure of compound **S8-1**.

(E)-*N*-cyclopropyl-4-(prop-2-yn-1-yloxy)-3-styrylbenzenesulfonamide（E:Z=1.47:1）(**S8-1**). white solid, yield 23%. m.p. 92.4~93.1□. ^{1}H NMR (400 MHz, Chloroform-*d*) δ 8.25 (d, *J* = 2.3 Hz, 1H), 7.89 (ddd, *J* = 8.7, 6.2, 2.4 Hz, 2H), 7.70 (d, *J* = 2.4 Hz, 1H), 7.68 – 7.62 (m, 1H), 7.49 (dd, *J* = 13.6, 6.1 Hz, 2H), 7.44 – 7.36 (m, 1H), 7.28 (d, *J* = 5.6 Hz, 1H), 7.25 – 7.19 (m, 3H), 6.88 (d, *J* = 12.1 Hz, 1H), 6.76 (d, *J* = 12.0 Hz, 1H), 5.04 (s, 1H), 4.96 (d, *J* = 2.4 Hz, 1H), 4.90 (d, *J* = 2.4 Hz, 2H), 4.68 (s, 1H), 2.70 (d, *J* = 2.4 Hz, 1H), 2.68 (t, *J* = 2.6 Hz, 1H), 2.39 (tt, *J* = 6.7, 3.6 Hz, 1H), 1.96 (tt, *J* = 6.8, 3.4 Hz, 1H), 0.80 – 0.67 (m, 3H), 0.63 (td, *J* = 4.6, 4.1 Hz, 2H), 0.50 (td, *J* = 7.1, 4.9 Hz, 2H). ^{13}C NMR (101 MHz, Chloroform-*d*) δ 158.63, 157.80, 137.16, 136.83, 132.56, 132.50, 131.84, 131.69, 130.14, 129.04, 128.87, 128.36, 128.25, 128.06, 127.97, 127.92, 127.33, 126.97, 125.87, 124.58, 121.46, 112.47, 112.18, 77.72, 77.66, 76.75, 76.64, 56.56, 56.47, 24.48, 24.33, 6.34, 6.23. ESI-MS (MeOH) m/z $C_{20}H_{19}NO_3S$ $[M + H]^+$ calcd. for:354.12, found:354.18.

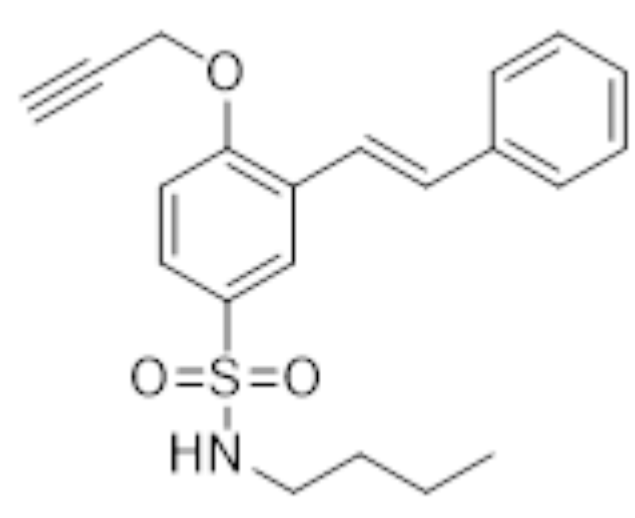

Fig. C49 The chemical structure of compound **S8-2**.

(E)-*N*-butyl-4-(prop-2-yn-1-yloxy)-3-styrylbenzenesulfonamide（E:Z=1.8:1）（**S8-2**）. white solid, yield 26%. m.p. 82.6~83.9□. ^{1}H NMR (400 MHz, Chloroform-*d*) δ 7.83 (td, *J* = 8.7, 2.4 Hz, 1H), 7.71 – 7.60 (m, 2H), 7.47 (dd, *J* = 13.1, 5.8 Hz, 1H), 7.38 (d, *J* = 7.3 Hz, 1H), 7.32 – 7.26 (m, 3H), 7.24 (t, *J* = 3.8 Hz, 1H), 7.19 (dd, *J* = 8.7, 6.1 Hz, 1H), 6.85 (d, *J* = 12.1 Hz, 1H), 6.73 (d, *J* = 12.1 Hz, 1H), 4.93 (d, *J* = 2.4 Hz, 1H), 4.85 (d, *J* = 2.4 Hz, 2H), 4.69 (t, *J* = 6.2 Hz, 1H), 4.26 (t, *J* = 6.3 Hz, 1H), 3.06 (q, *J* = 6.8 Hz, 1H), 2.76 (q, *J* = 6.7 Hz, 2H), 2.66 (t, *J* = 2.4 Hz, 1H), 1.71 (s, 1H), 1.62 – 1.26 (m, 8H), 0.95 (t, *J* = 7.2 Hz, 5H). ^{13}C NMR (101 MHz, Chloroform-*d*) δ 158.38, 157.63, 137.17, 136.79, 132.86, 132.52, 132.25, 131.64, 129.68, 128.96, 128.85, 128.39, 128.23, 128.07, 127.90, 127.83, 127.54, 127.49, 126.96, 125.53, 124.33, 121.45, 112.51, 112.29, 77.74, 77.69, 76.70, 76.59, 56.53, 56.45, 43.09, 42.88, 31.71, 19.84, 19.80, 13.73, 13.69. ESI-MS (MeOH) m/z $C_{21}H_{23}NO_3S$ $[M + H]^+$ calcd. for:370.15, found:370.22.

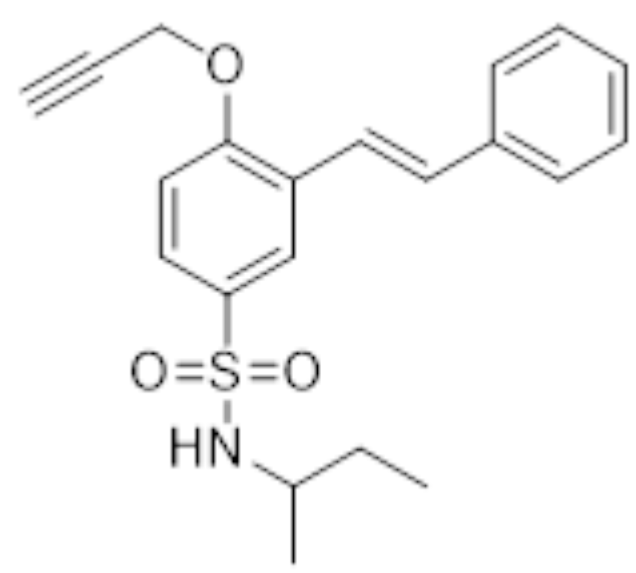

Fig. C50 The chemical structure of compound **S8-3**.

(E)-*N*-(sec-butyl)-4-(prop-2-yn-1-yloxy)-3-styrylbenzenesulfonamide（E:Z=1.2:1）（**S8-3**）. white solid, yield 30%. m.p. 96.1~99.4□. ^{1}H NMR (400 MHz, Chloroform-*d*) δ 8.23 (d, *J* = 2.3 Hz, 1H), 7.84 (ddd, *J* = 11.7, 8.7, 2.4 Hz, 2H), 7.73 (d, *J* = 2.4 Hz, 1H), 7.66 – 7.58 (m, 2H), 7.46 (dd, *J* = 13.5, 6.0 Hz, 2H), 7.41 – 7.32 (m, 1H), 7.27 (s, 1H), 7.25 (s, 1H), 7.18 (d, *J* = 3.8 Hz, 1H), 7.16 (d, *J* = 3.7 Hz, 1H), 6.83 (d, *J* = 12.2 Hz, 1H), 6.71 (d, *J* = 12.2 Hz, 1H), 4.92 (d, *J* = 2.4 Hz, 2H), 4.83 (d, *J* = 2.5 Hz, 2H), 4.71 (d, *J* = 8.0 Hz, 1H), 4.29 (d, *J* = 7.9 Hz, 1H), 3.35 (dq, *J* = 8.0, 6.4 Hz, 1H), 3.17 – 3.03 (m, 1H), 2.67 (t, *J* = 2.4 Hz, 1H), 2.64 (t, *J* = 2.4 Hz, 1H), 1.50 (q, *J* = 7.1 Hz, 2H), 1.41 (p, *J* = 7.2 Hz, 2H), 1.14 (d, *J* = 6.6 Hz, 3H), 1.01 (d, *J* = 6.5 Hz, 3H), 0.91 (t, *J* = 7.4 Hz, 3H), 0.85 (t, *J* = 7.4 Hz, 3H). ^{13}C NMR (101 MHz, Chloroform-*d*) δ 158.24, 157.51, 137.19, 136.76, 134.17, 133.59, 132.48, 131.53, 129.59, 128.85, 128.83, 128.41, 128.19, 127.95, 127.80, 127.70, 127.51, 127.44, 126.94, 125.46, 124.21, 121.50, 112.47, 112.36, 77.74, 77.69, 76.66, 76.53, 56.54, 56.46, 51.53, 51.22, 30.39, 30.32, 21.34, 21.23, 10.09, 10.01. ESI-MS (MeOH) m/z $C_{21}H_{23}NO_3S$ $[M + H]^+$ calcd. for:370.15, found:370.21.

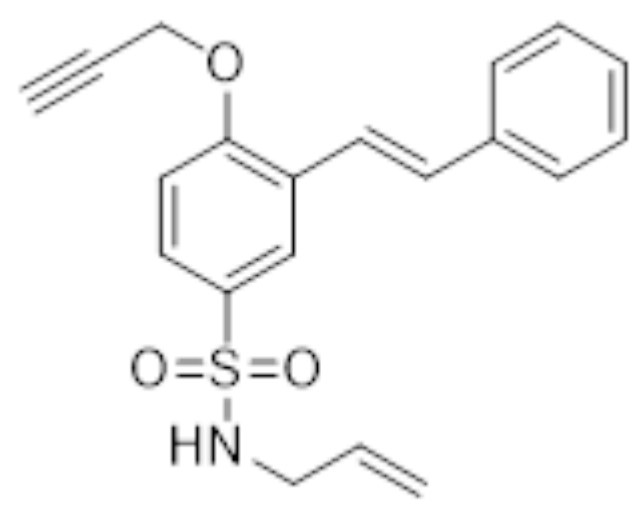

Fig. C51 The chemical structure of compound **S8-4**.

(E)-*N*-allyl-4-(prop-2-yn-1-yloxy)-3-styrylbenzenesulfonamide（E:Z=1.6:1）（**S8-4**）. white solid, yield 22%. m.p. 126.1~130.7□. ^{1}H NMR (400 MHz, Chloroform-*d*) δ 8.23 (d, *J* = 2.3 Hz, 1H), 7.83 (ddd, *J* = 10.9, 8.7, 2.4 Hz, 2H), 7.67 (d, *J* = 2.4 Hz, 1H), 7.63 (d, *J* = 7.3 Hz, 1H), 7.47 (dd, *J* = 13.3, 5.8 Hz, 2H), 7.42 – 7.34 (m, 1H), 7.28 (d, *J* = 14.1 Hz, 2H), 7.25 (d, *J* = 3.2 Hz, 1H), 7.19 (dd, *J* = 8.7, 3.5 Hz, 2H), 6.85 (d, *J* = 12.1 Hz, 1H), 6.73 (d, *J* = 12.1 Hz, 1H), 5.84 (ddt, *J* = 16.2, 10.2, 5.8 Hz, 1H), 5.74 (ddt, *J* = 16.2, 10.3, 5.7 Hz, 1H), 4.97 (t, *J* = 6.3 Hz, 1H), 4.93 (d, *J* = 2.4 Hz, 1H), 4.85 (d, *J* = 2.4 Hz, 2H), 4.49 (t, *J* = 6.3 Hz, 1H), 3.70 (tt, *J* = 6.2, 1.7 Hz, 1H), 3.38 (tt, *J* = 6.2, 1.7 Hz, 2H), 2.68 (t, *J* = 2.4 Hz, 1H), 2.67 (t, *J* = 2.4 Hz, 1H).^{13}C NMR (101 MHz, Chloroform-*d*) δ 158.47, 157.68, 137.11, 136.73, 133.08, 132.75, 132.55, 132.03, 131.64, 129.74, 128.91, 128.81, 128.41, 128.20, 128.06, 127.88, 127.78, 127.58, 127.51, 126.93, 125.53, 124.25, 121.35, 117.83, 117.59, 112.51, 112.35, 77.69, 77.65, 76.72, 76.62, 56.50, 56.43, 45.89, 45.68. ESI-MS (MeOH) m/z $C_{20}H_{19}NO_3S$ $[M + H]^+$ calcd. for:354.12, found:354.18.

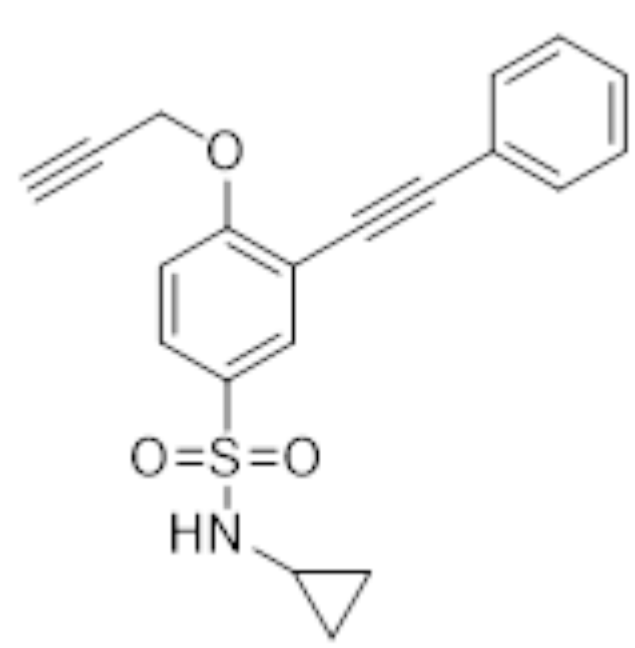

Fig. C52 The chemical structure of compound **S10-1**.

N-cyclopropyl-3-(phenylethynyl)-4-(prop-2-yn-1-yloxy)benzenesulfonamide（**S10-1**）. white solid, yield 19%. m.p. 53.1~52.6□. ^{1}H NMR (400 MHz, Chloroform-*d*) δ 8.05 (d, *J* = 2.4 Hz, 1H), 7.84 (dd, *J* = 8.8, 2.4 Hz, 1H), 7.56 (dq, *J* = 7.1, 2.6, 2.2 Hz, 2H), 7.40 – 7.33 (m, 3H), 7.16 (d, *J* = 8.8 Hz, 1H), 4.96 (s, 1H), 4.88 (d, *J* = 2.4 Hz, 2H), 2.60 (t, *J* = 2.4 Hz, 1H), 2.28 (dddd, *J* = 7.1, 5.7, 4.4, 2.2 Hz, 1H), 0.62 (dt, *J* = 7.3, 1.9 Hz, 4H). ^{13}C NMR (101 MHz, Chloroform-*d*) δ 160.92, 133.01, 132.42, 131.88, 129.01, 128.88, 128.51, 122.84, 114.46, 112.57, 95.61, 83.80, 77.40, 77.04, 56.86, 24.43, 6.33. ESI-MS (MeOH) m/z $C_{20}H_{17}NO_3S$ $[M + H]^+$ calcd. for:352.10, found:352.19.

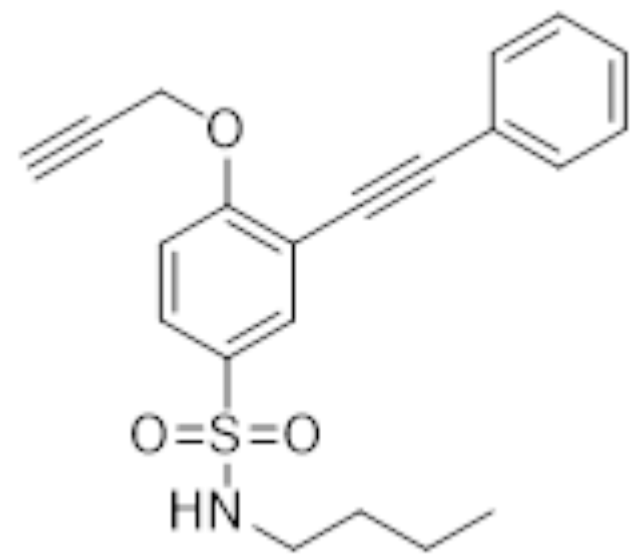

Fig. C53 The chemical structure of compound **S10-2**.

N-butyl-3-(phenylethynyl)-4-(prop-2-yn-1-yloxy)benzenesulfonamide (**S10-2**). white solid, yield 20%. m.p.47.6~49.2□. ^{1}H NMR (400 MHz, Chloroform-*d*) δ 8.01 (d, *J* = 2.4 Hz, 1H), 7.81 (dd, *J* = 8.8, 2.3 Hz, 1H), 7.56 (dq, *J* = 7.9, 3.1, 2.4 Hz, 2H), 7.37 (q, *J* = 2.9 Hz, 3H), 7.15 (d, *J* = 8.8 Hz, 1H), 4.87 (d, *J* = 2.4 Hz, 2H), 4.56 (t, *J* = 6.2 Hz, 1H), 2.96 (q, *J* = 6.8 Hz, 2H), 2.59 (t, *J* = 2.4 Hz, 1H), 1.46 (dq, *J* = 8.6, 7.1 Hz, 2H), 1.37 – 1.25 (m, 2H), 0.87 (t, *J* = 7.3 Hz, 3H). ^{13}C NMR (101 MHz, Chloroform-*d*) δ 160.76, 132.83, 132.70, 131.88, 128.87, 128.63, 128.51, 122.86, 114.47, 112.65, 95.58, 83.83, 77.44, 76.99, 56.85, 43.09, 31.72, 19.83, 13.68.ESI-MS (MeOH) m/z $C_{21}H_{21}NO_3S$ $[M + H]^+$ calcd. for:368.13, found:368.22.

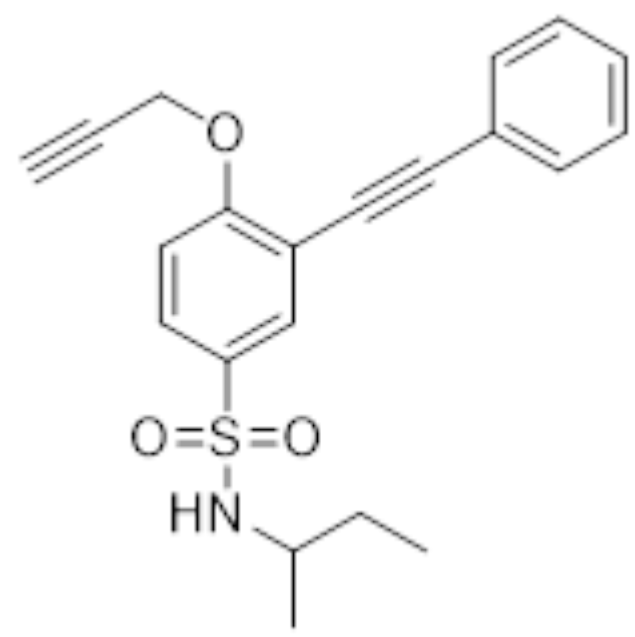

Fig. C54 The chemical structure of compound **S10-3**.

N-(sec-butyl)-3-(phenylethynyl)-4-(prop-2-yn-1-yloxy)benzenesulfonamide (**S10-3**). white solid, yield 25%. m.p.61.5~62.7□. ^{1}H NMR (400 MHz, Chloroform-*d*) δ 8.05 (d, *J* = 2.4 Hz, 1H), 7.83 (dd, *J* = 8.8, 2.4 Hz, 1H), 7.61 – 7.50 (m, 2H), 7.35 (p, *J* = 3.9, 3.4 Hz, 3H), 7.13 (d, *J* = 8.8 Hz, 1H), 4.87 – 4.80 (m, 3H), 3.26 (dq, *J* = 8.0, 6.4 Hz, 1H), 2.59 (t, *J* = 2.4 Hz, 1H), 1.42 (p, *J* = 7.2 Hz, 2H), 1.05 (d, *J* = 6.6 Hz, 3H), 0.82 (t, *J* = 7.4 Hz, 3H). ^{13}C NMR (101 MHz, Chloroform-*d*) δ 160.59, 134.11, 132.54, 131.82, 128.80, 128.49, 128.46, 122.83, 114.26, 112.59, 95.42, 83.90, 77.40, 76.94, 56.81, 51.55, 30.30, 21.24, 10.06. ESI-MS (MeOH) m/z $C_{21}H_{21}NO_3S$ $[M + H]^+$ calcd. for:368.13, found:368.21.

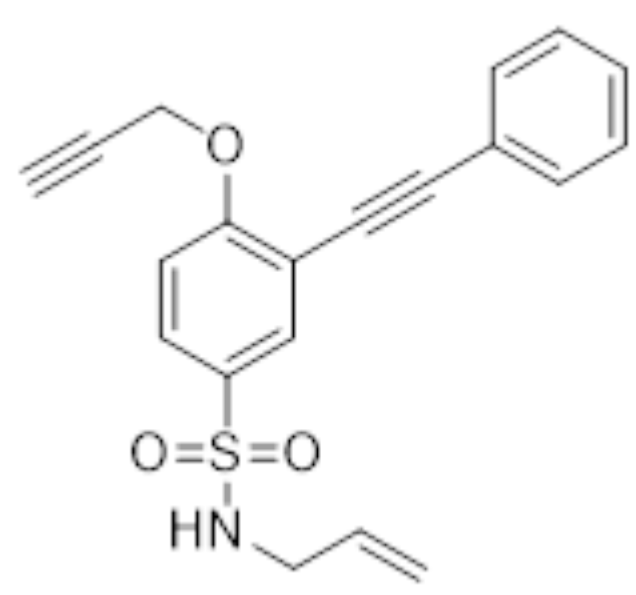

Fig. C55 The chemical structure of compound **S10-4**.

N-allyl-3-(phenylethynyl)-4-(prop-2-yn-1-yloxy) benzene-sulfonamide (**S10-4**). white solid, yield 20%. m.p.74.6~77.4□. ^{1}H NMR (400 MHz, Chloroform-*d*) δ 8.02 (d, *J* = 2.3 Hz, 1H), 7.81 (dd, *J* = 8.9, 2.4 Hz, 1H), 7.55 (dd, *J* = 6.6, 3.0 Hz, 2H), 7.41 – 7.30 (m, 3H), 7.15 (d, *J* = 8.8 Hz, 1H), 5.74 (ddt, *J* = 16.3, 10.9, 5.8 Hz, 1H), 5.25 – 5.08 (m, 2H), 4.87 (d, *J* = 2.4 Hz, 2H), 4.78 (t, *J* = 6.3 Hz, 1H), 3.61 (t, *J* = 6.1 Hz, 2H), 2.64 – 2.56 (m, 1H). ^{13}C NMR (101 MHz, Chloroform-*d*) δ 160.86, 132.99, 132.75, 132.73, 131.88, 128.89, 128.70, 128.52, 122.82, 118.02, 114.53, 112.66, 95.64, 83.77, 77.40, 77.04, 56.85, 45.93. ESI-MS (MeOH) m/z $C_{20}H_{17}NO_3S$ $[M + H]^+$ calcd. for:352.10, found:352.15.

2. Copies of NMR Spectra of Products

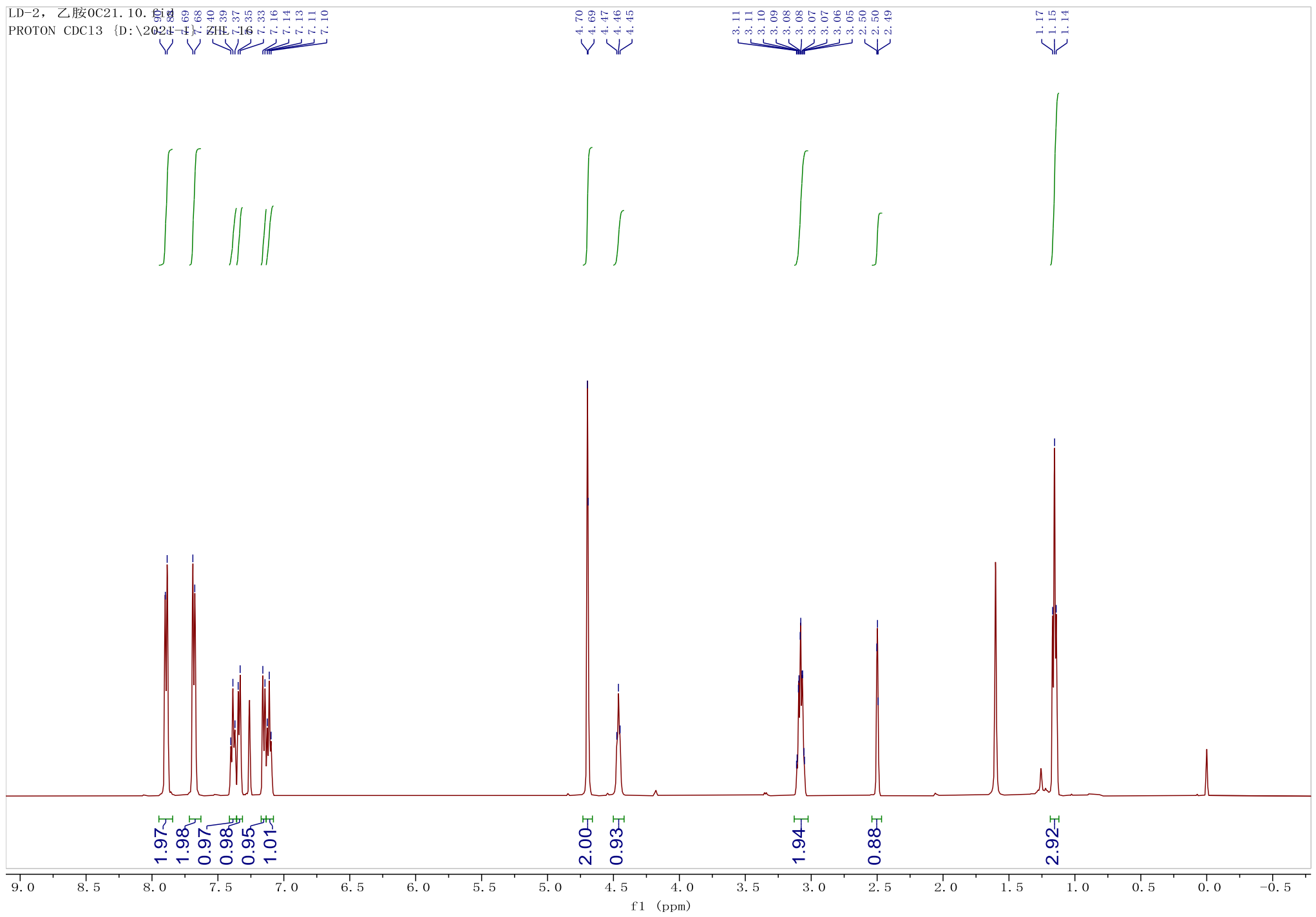

Fig. N1 ^{1}H NMR (500 MHz, CDCl3) spectra of product **4a-1**

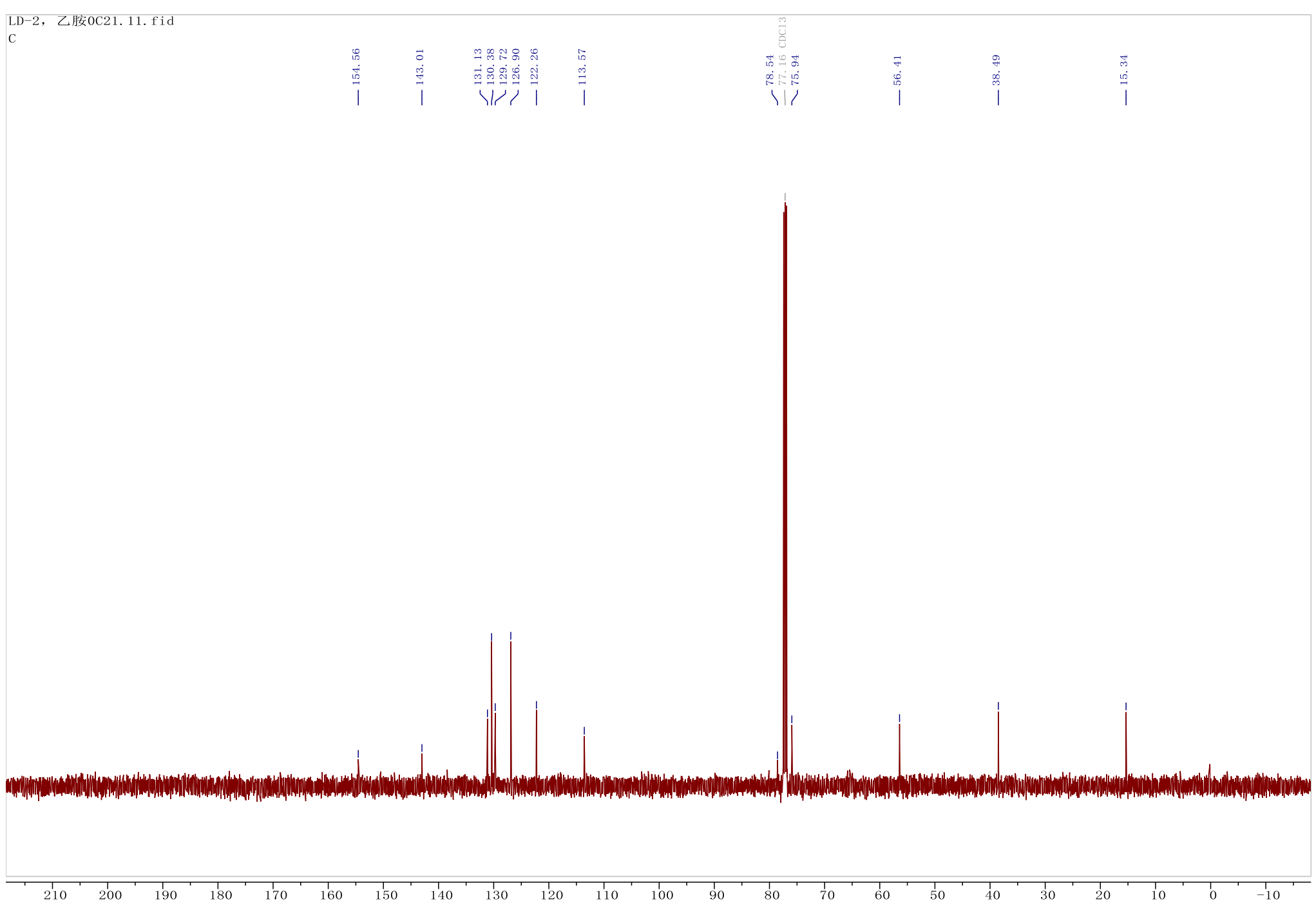

Fig. N2 ^{13}C NMR (126 MHz, CDCl3) spectra of product **4a-1**

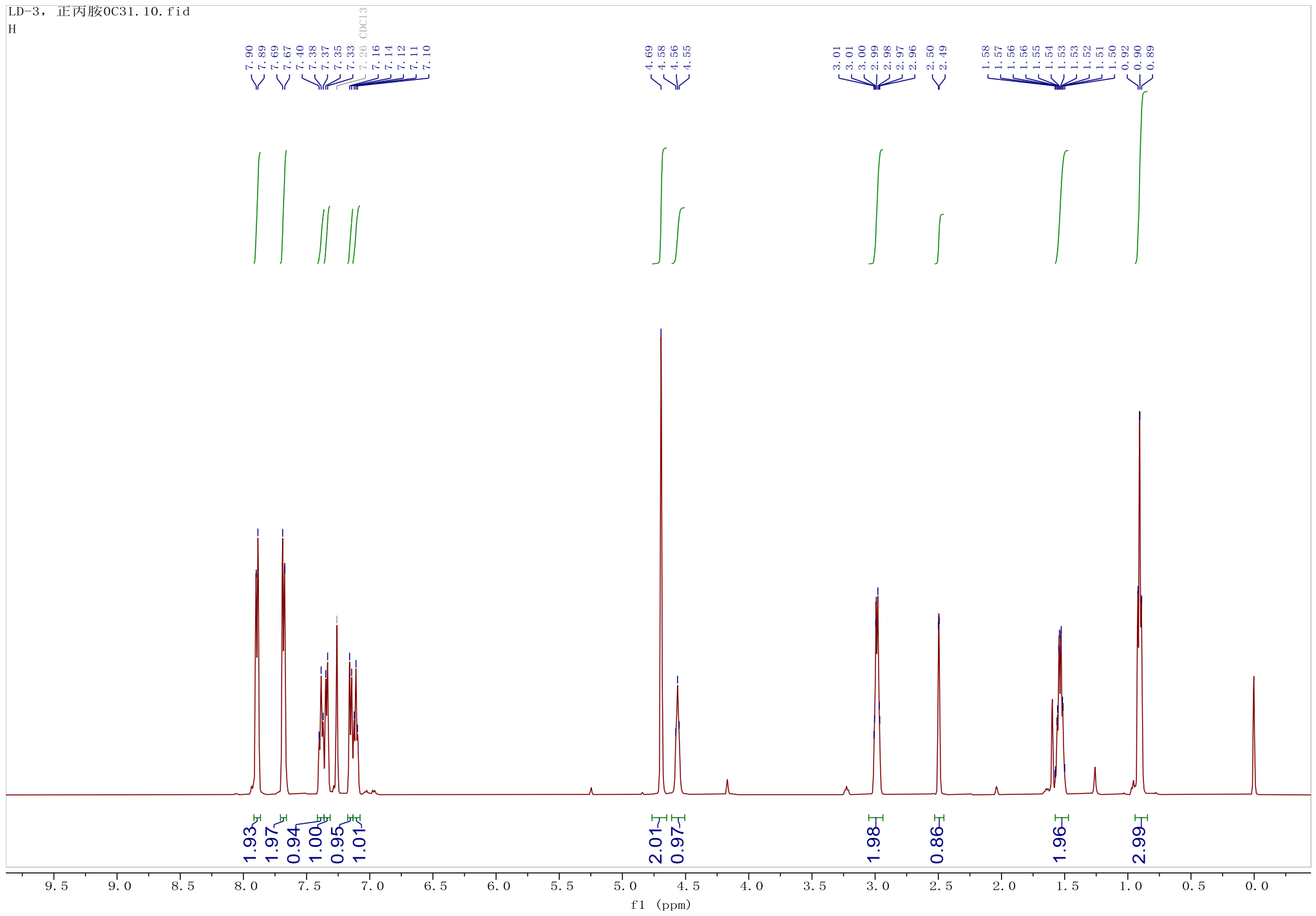

Fig. N3 ^{1}H NMR (500 MHz, CDCl3) spectra of product **4a-2**

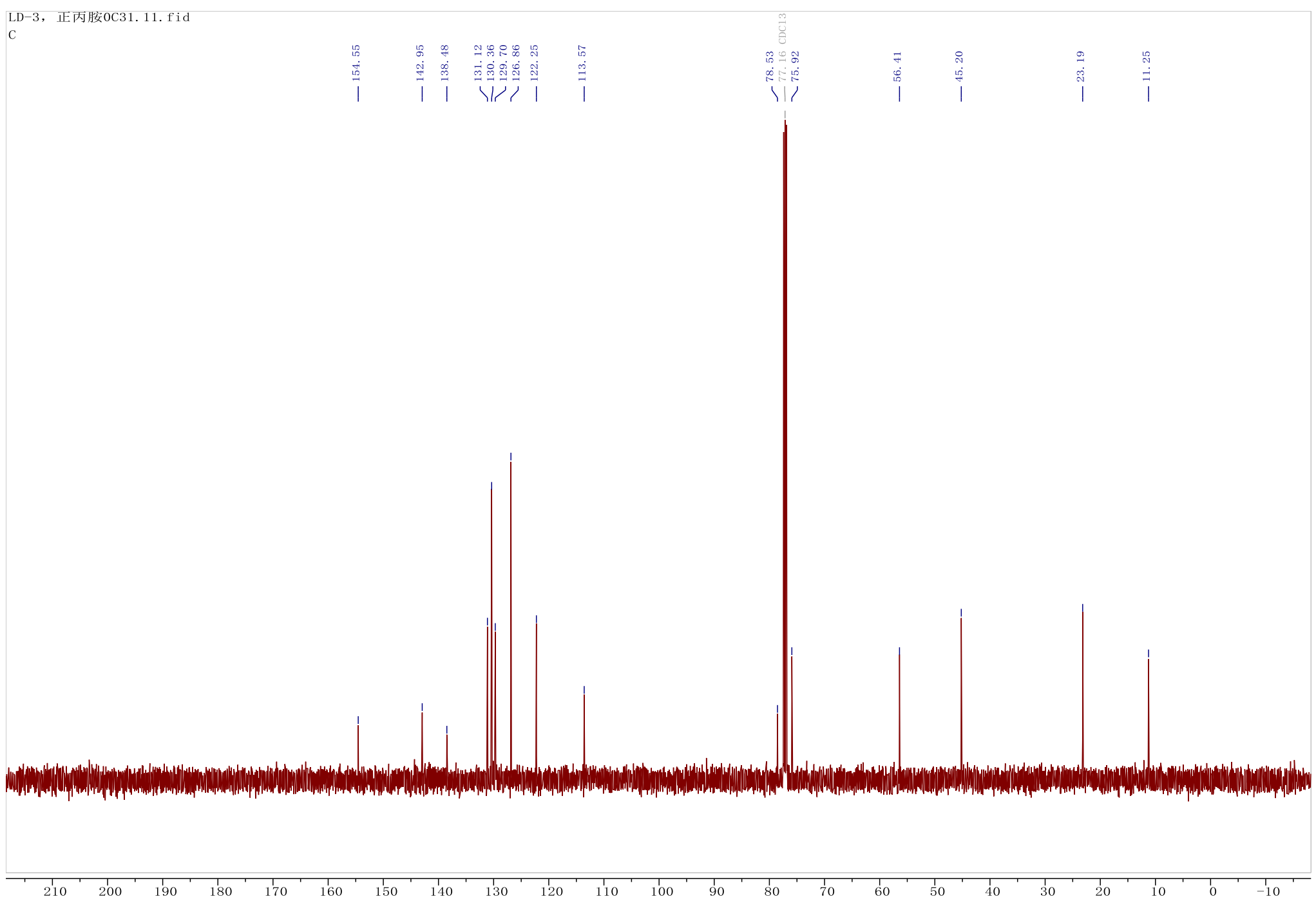

Fig. N4 ^{13}C NMR (126 MHz, CDCl3) spectra of product **4a-2**

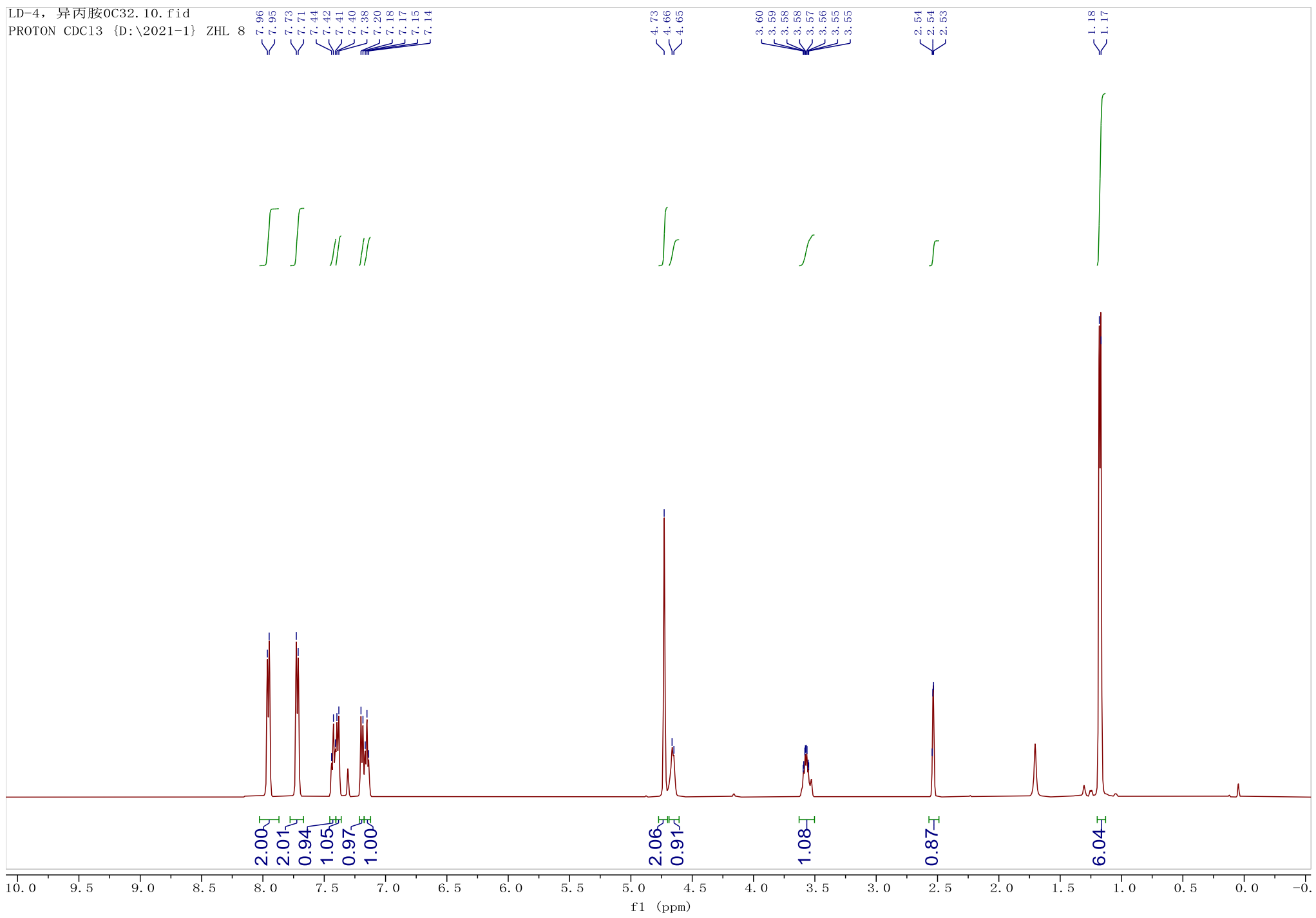

Fig. N5 ^{1}H NMR (500 MHz, CDCl3) spectra of product **4a-3**

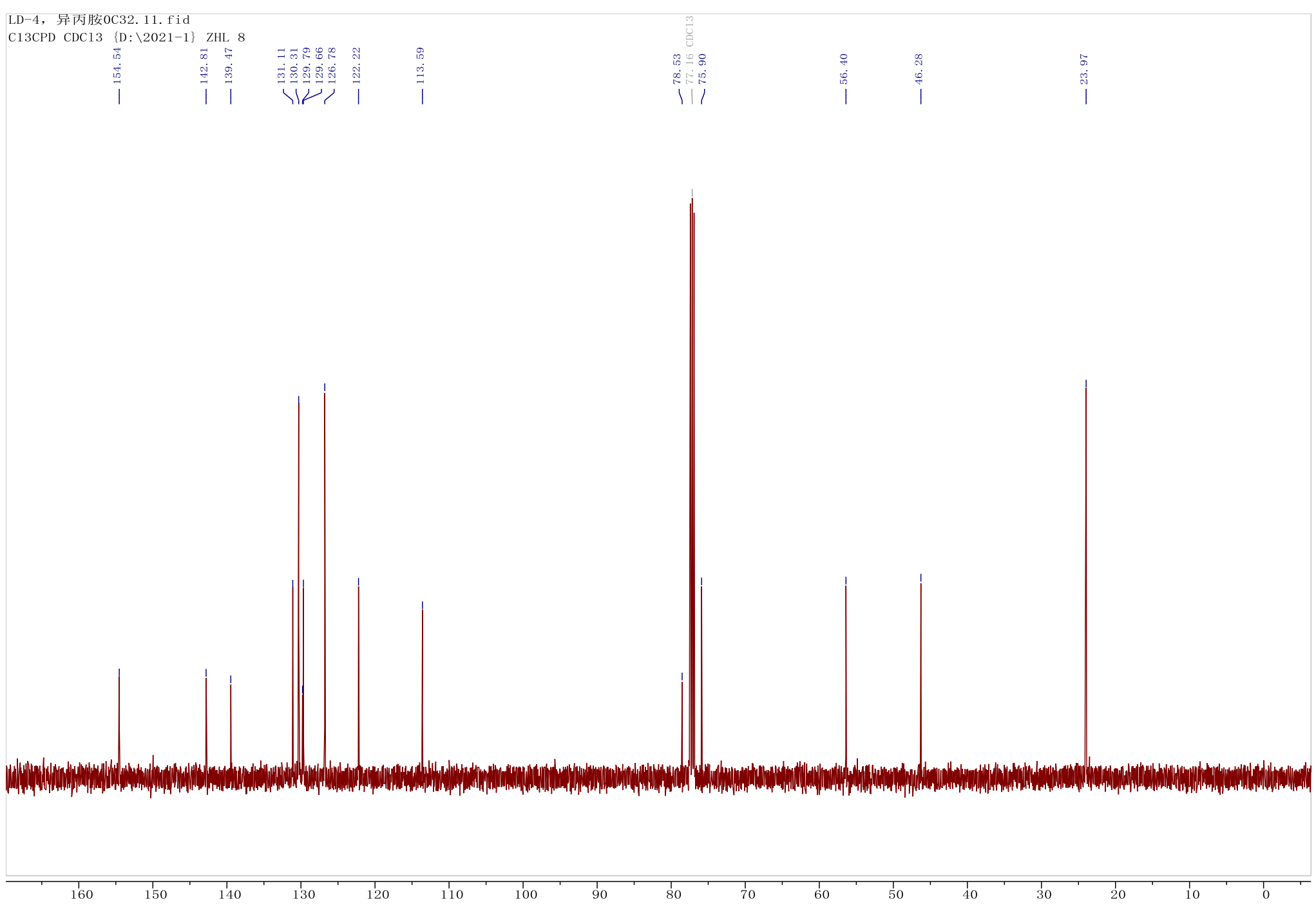

Fig. N6 ^{13}C NMR (126 MHz, CDCl3) spectra of product **4a-3**

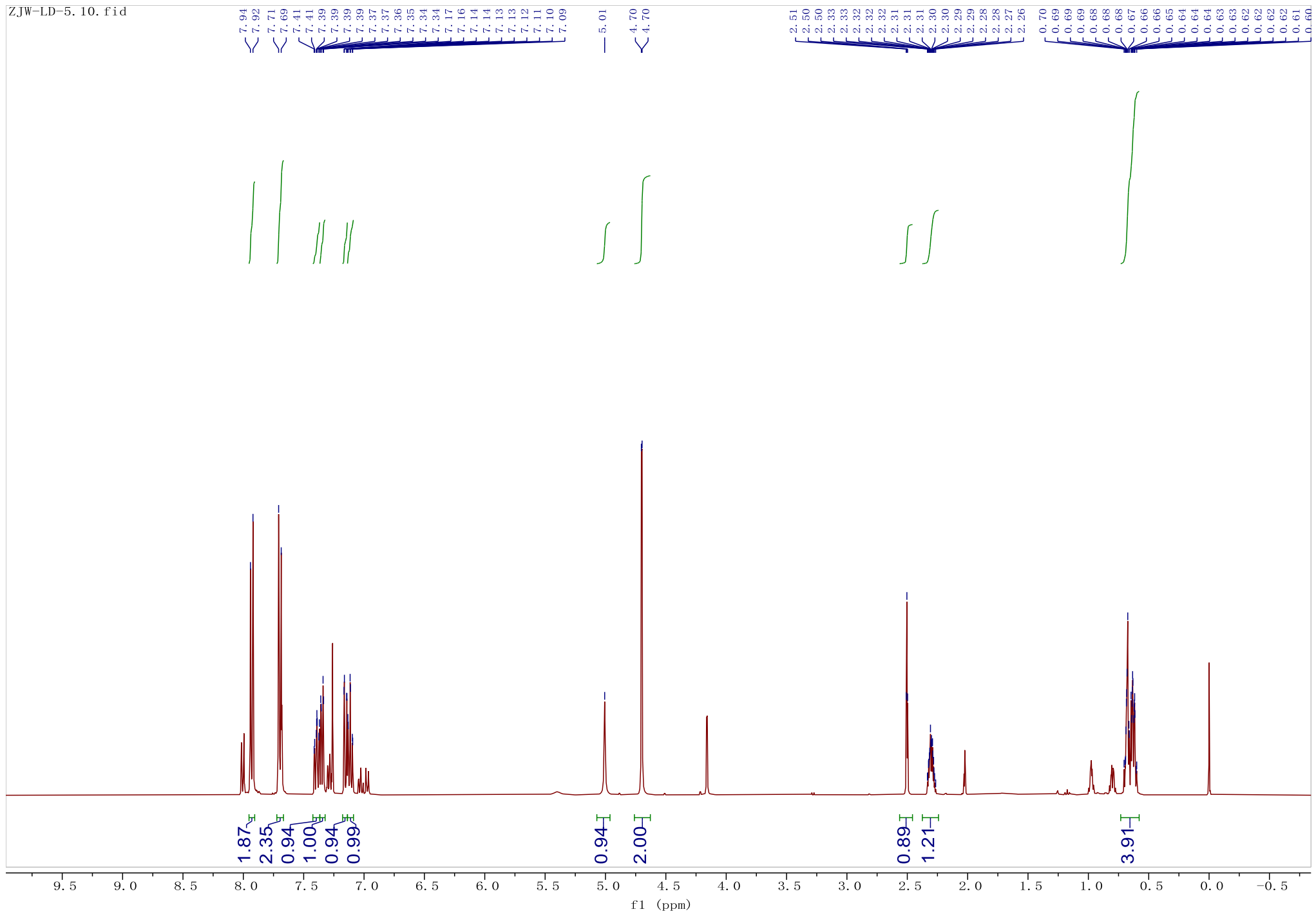

Fig. N7 ^{1}H NMR (400 MHz, CDCl3) spectra of product **4a-4**

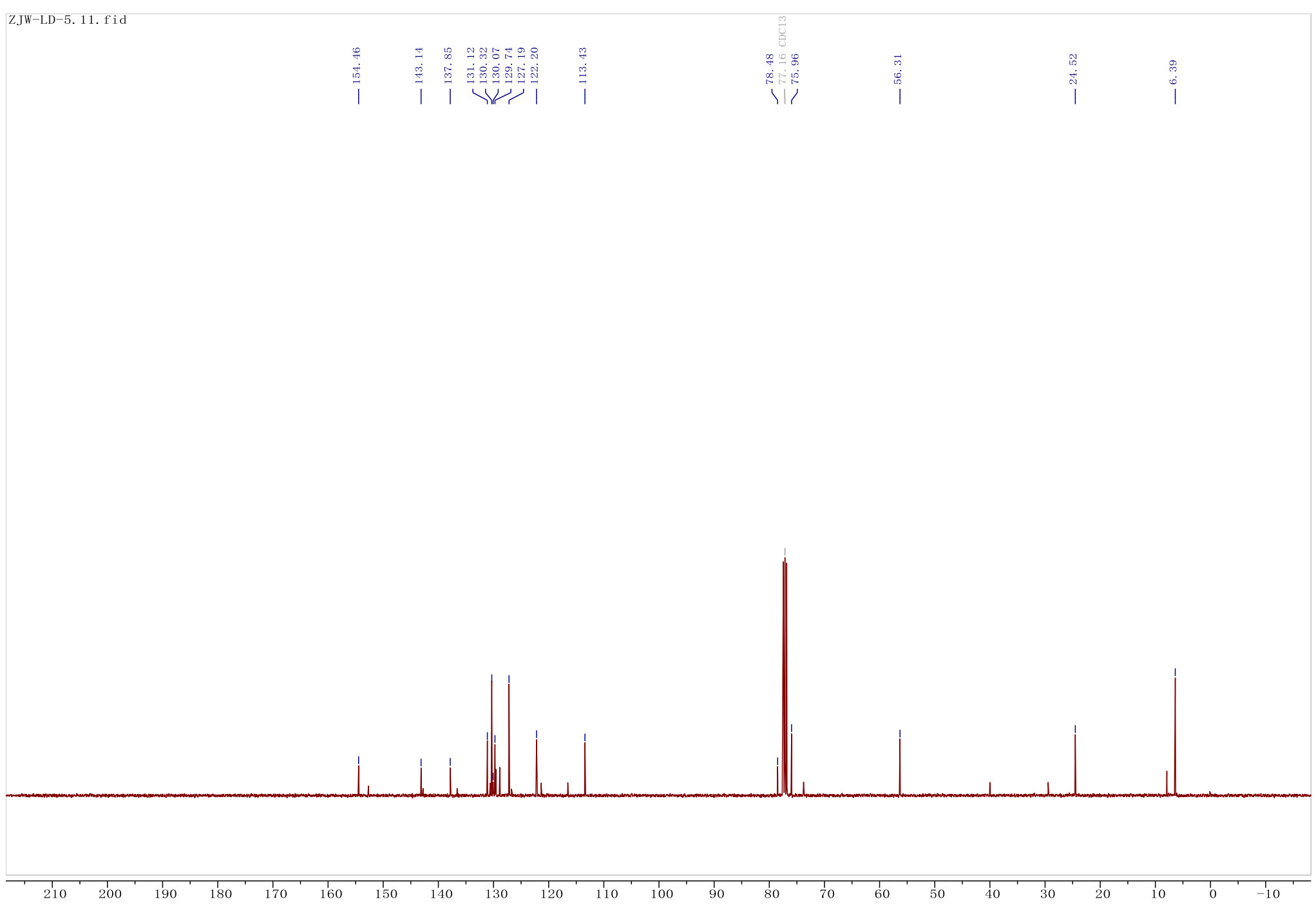

Fig. N8 ^{13}C NMR (101 MHz, CDCl3) spectra of product **4a-4**

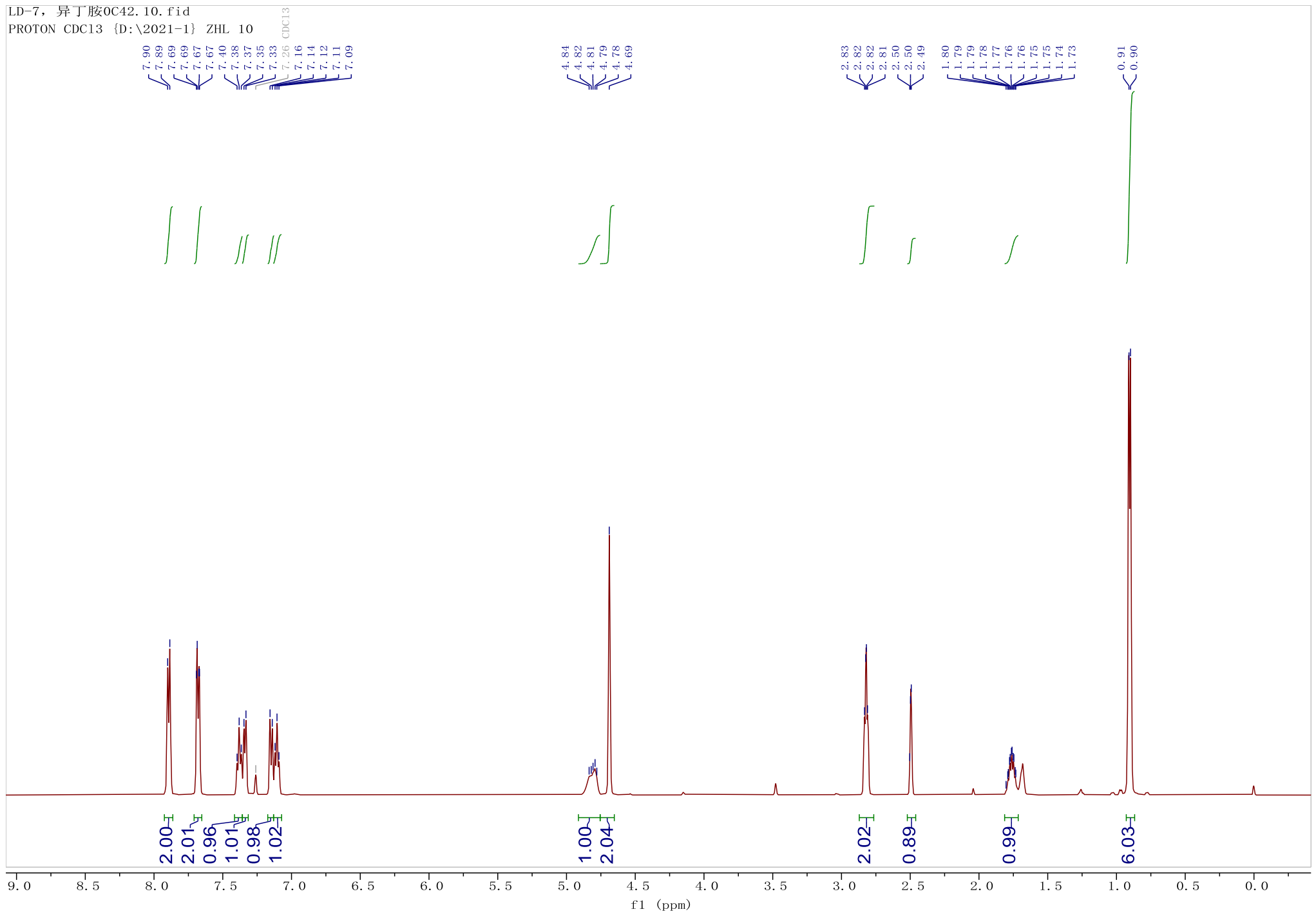

Fig. N9 ^{1}H NMR (500 MHz, CDCl3) spectra of product **4a-5**

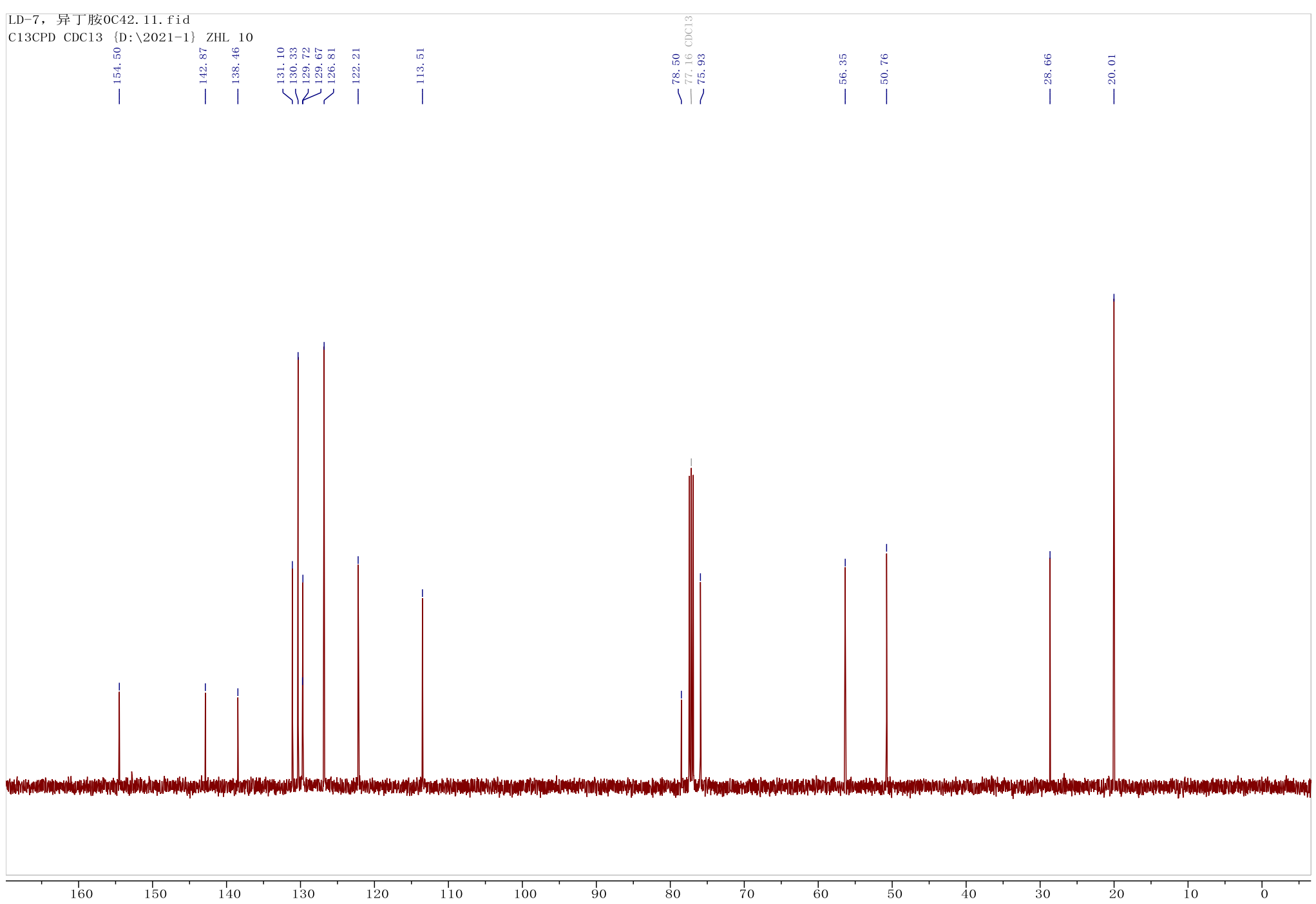

Fig. N10 ^{13}C NMR (126 MHz, CDCl3) spectra of product **4a-5**

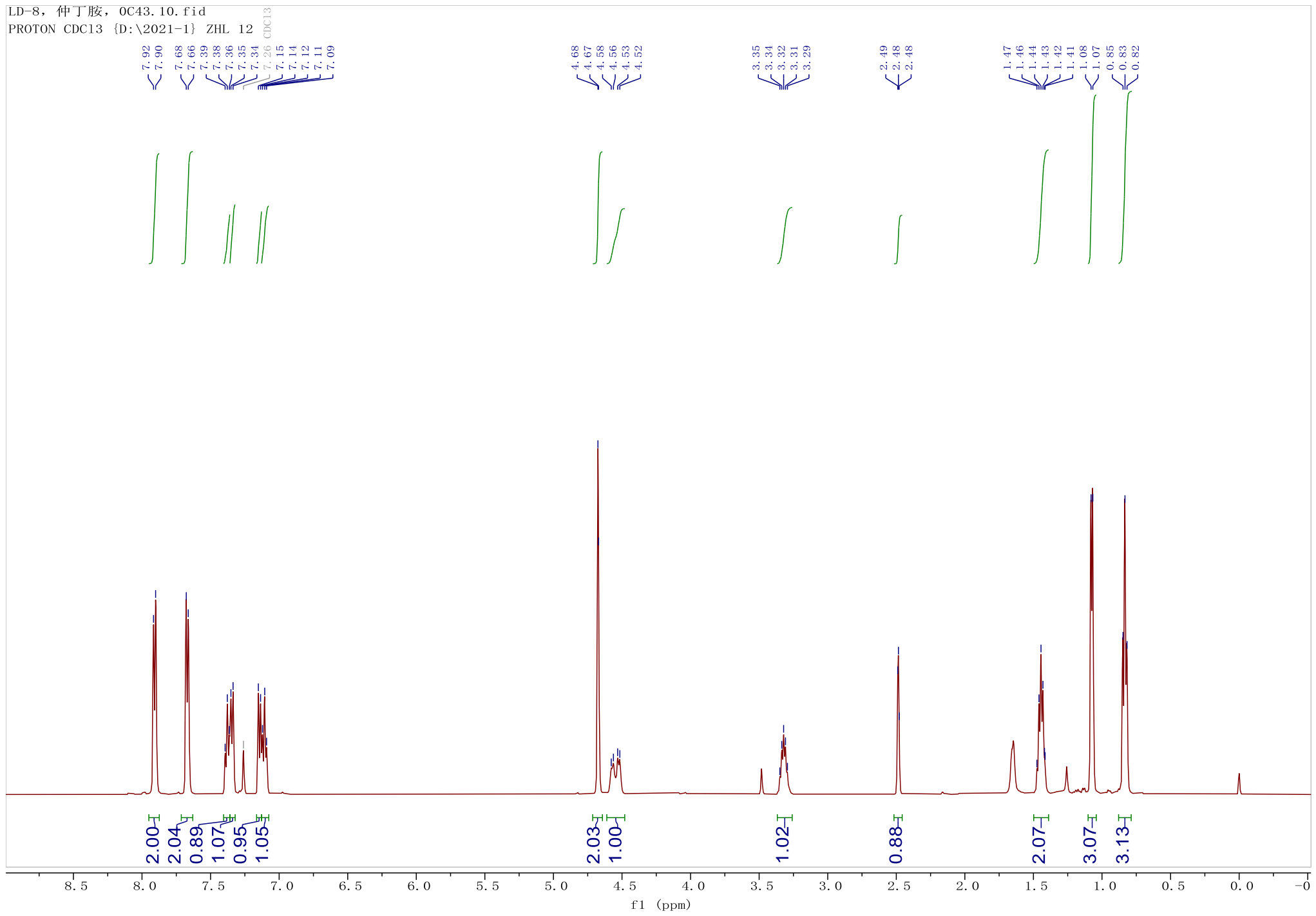

Fig. N11 ^{1}H NMR (500 MHz, CDCl3) spectra of product **4a-6**

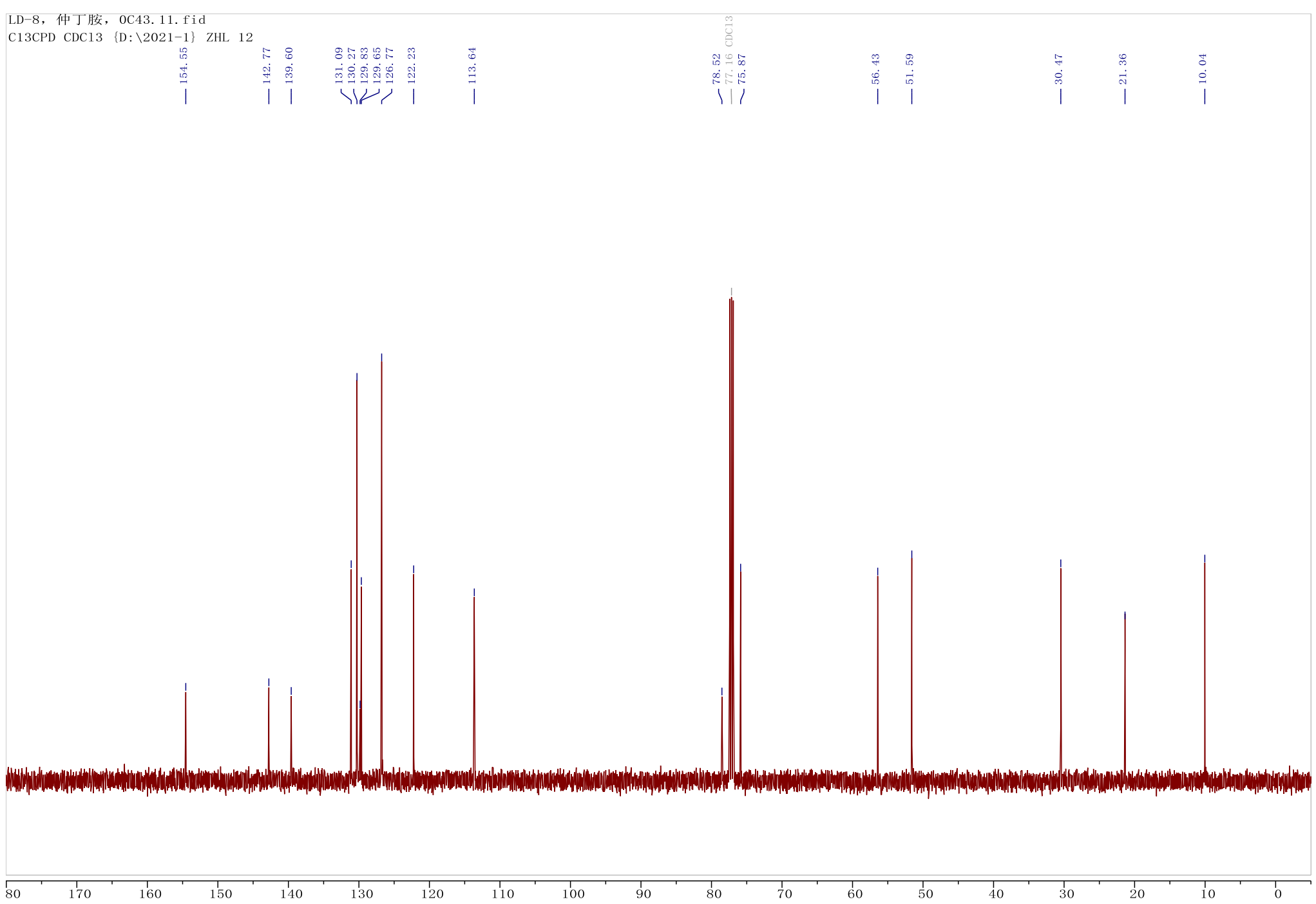

Fig. N12 ^{13}C NMR (126 MHz, CDCl3) spectra of product **4a-6**

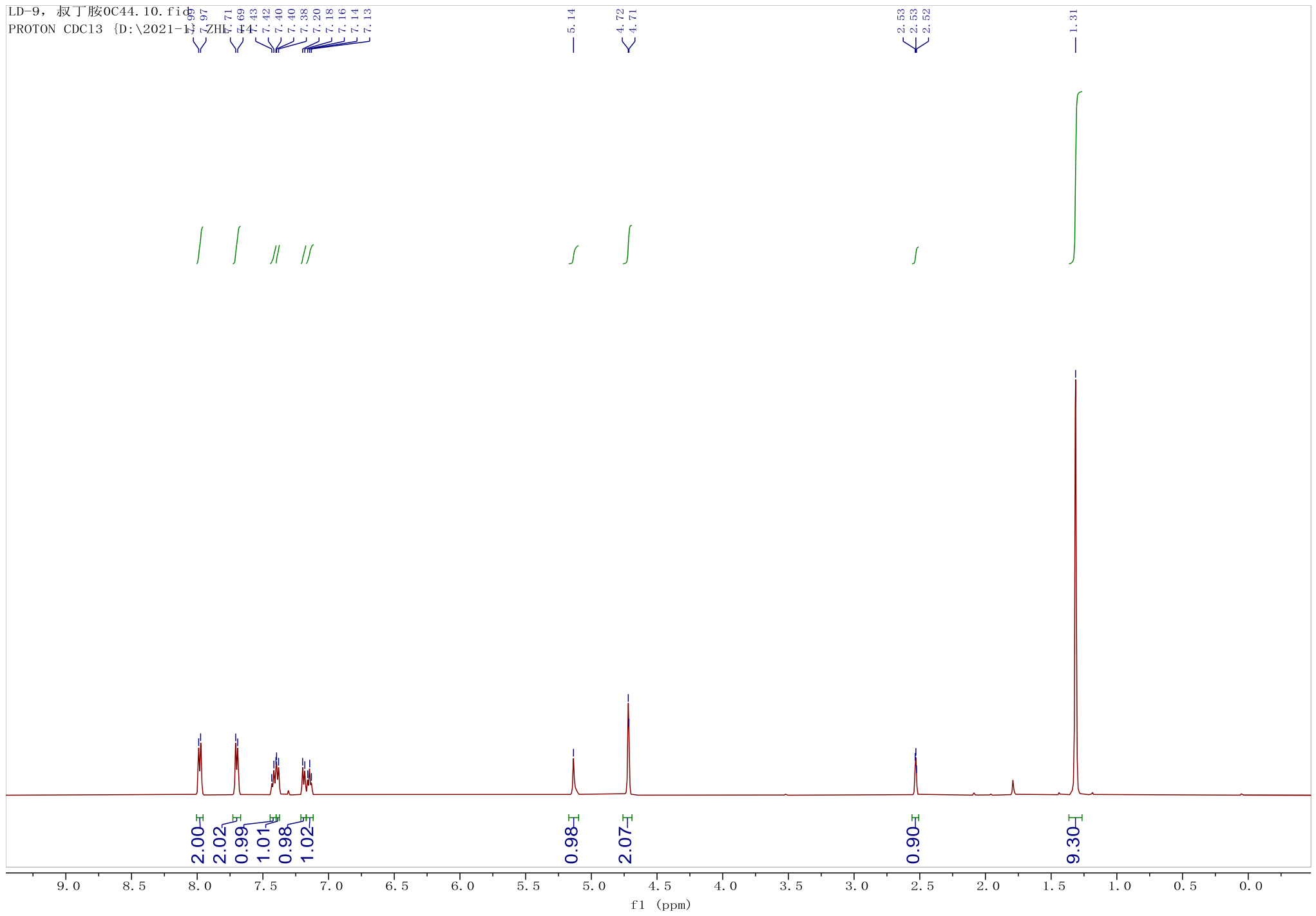

Fig. N13 ^{1}H NMR (500 MHz, CDCl3) spectra of product **4a-7**

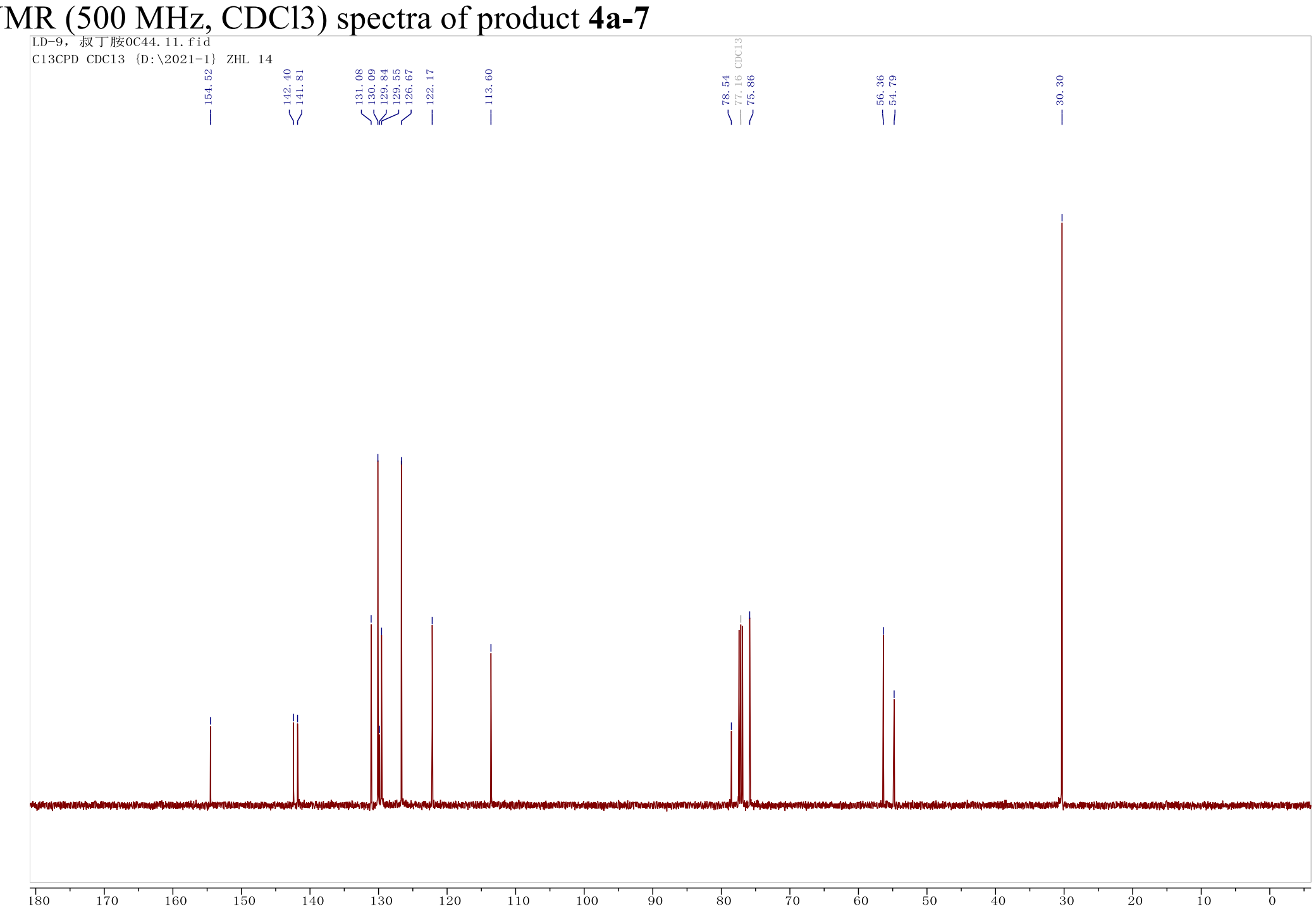

Fig. N14 ^{13}C NMR (126 MHz, CDCl3) spectra of product **4a-7**

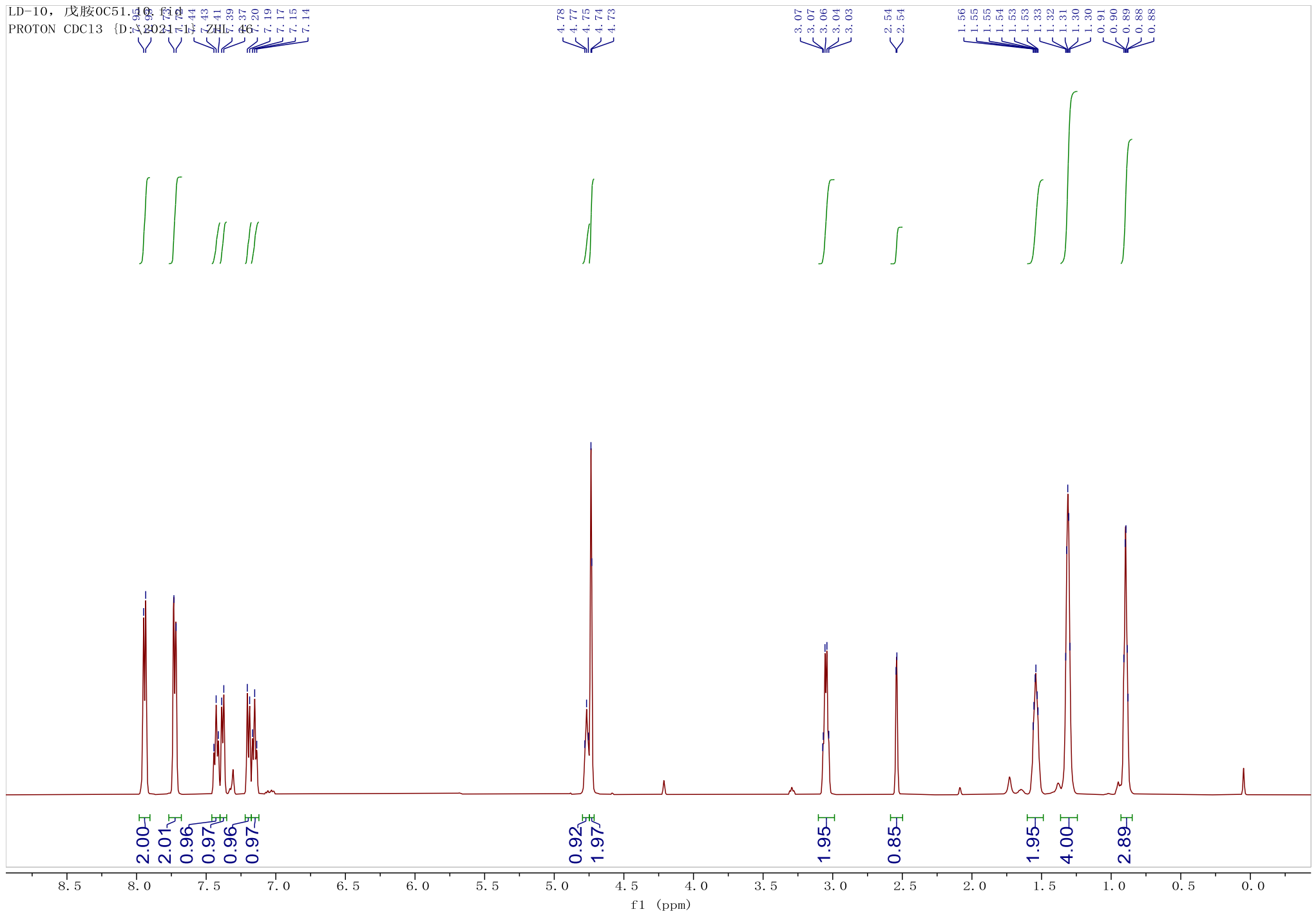

Fig. N15 ^{1}H NMR (500 MHz, CDCl3) spectra of product **4a-8**

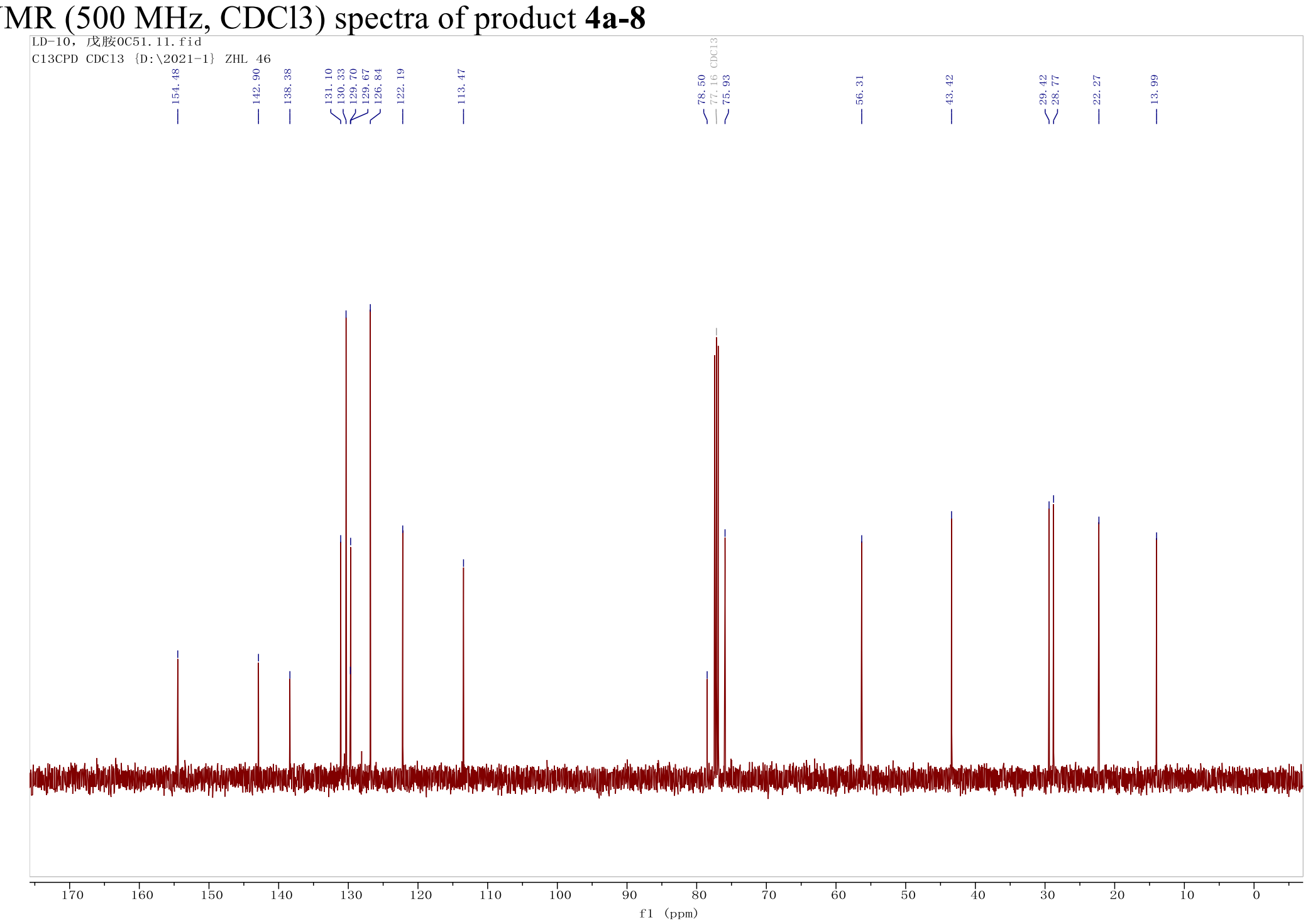

Fig. N16 ^{13}C NMR (126 MHz, CDCl3) spectra of product **4a-8**

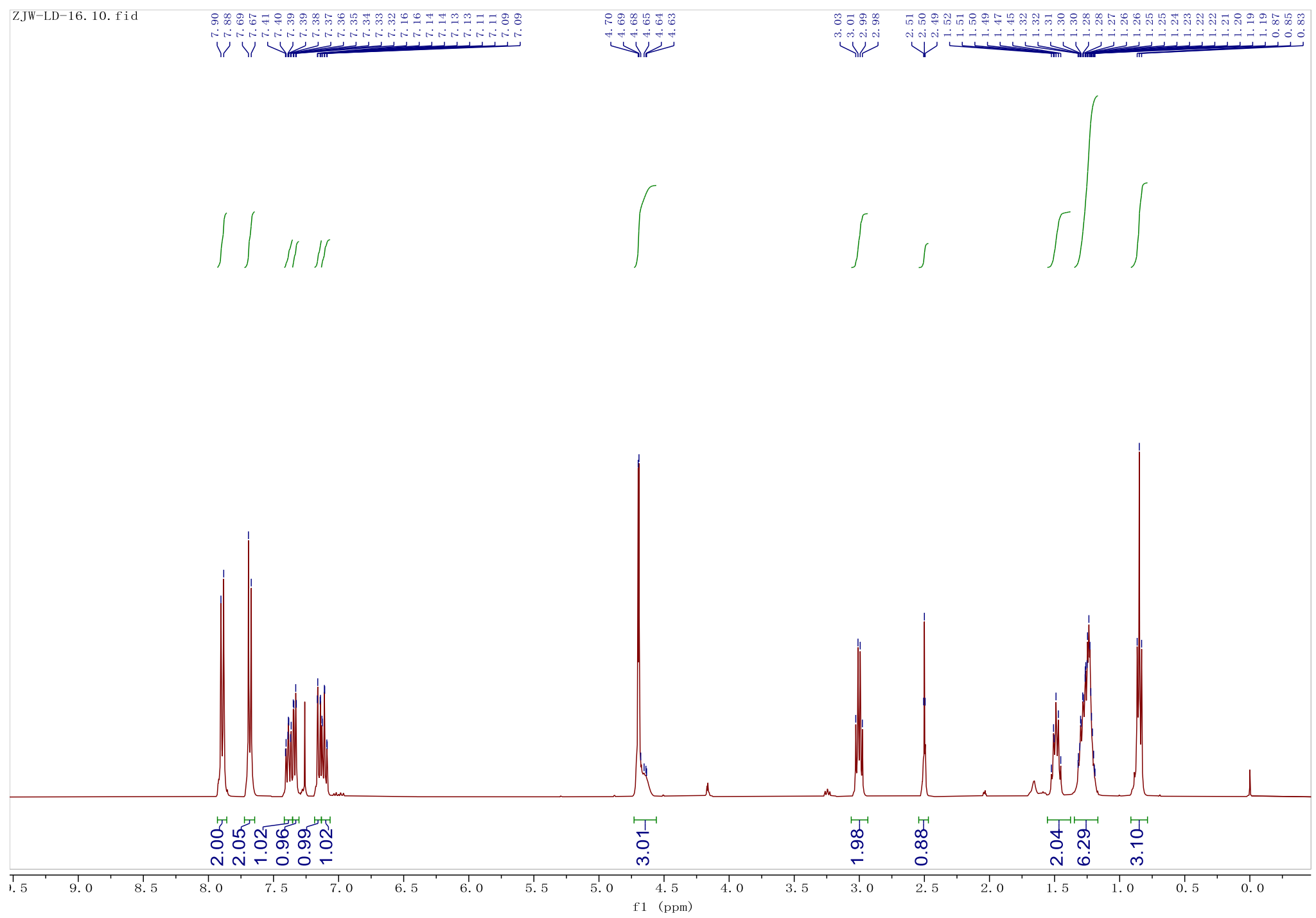

Fig. N17 ^{1}H NMR (400 MHz, CDCl3) spectra of product **4a-9**

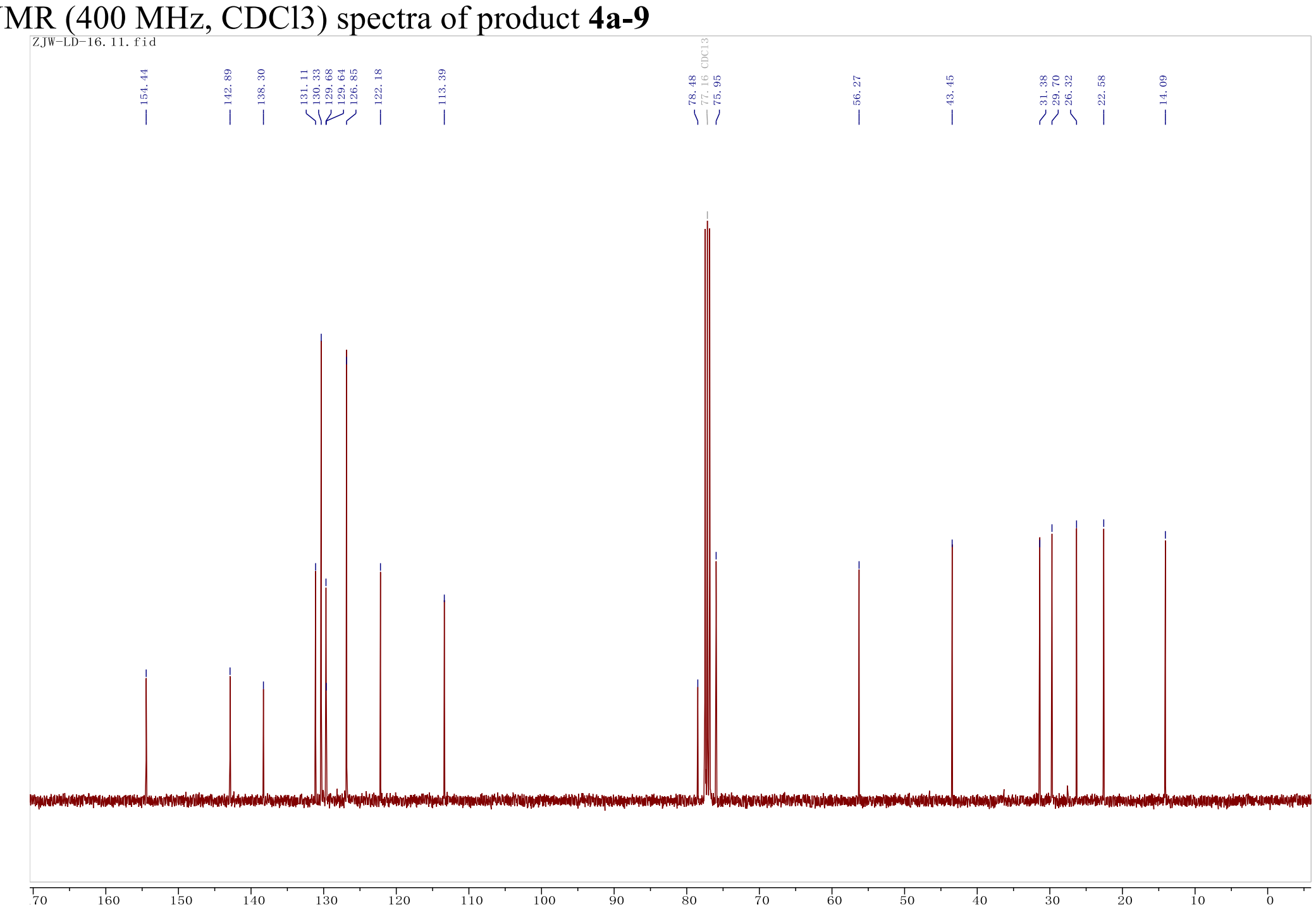

Fig. N18 ^{13}C NMR (101 MHz, CDCl3) spectra of product **4a-9**

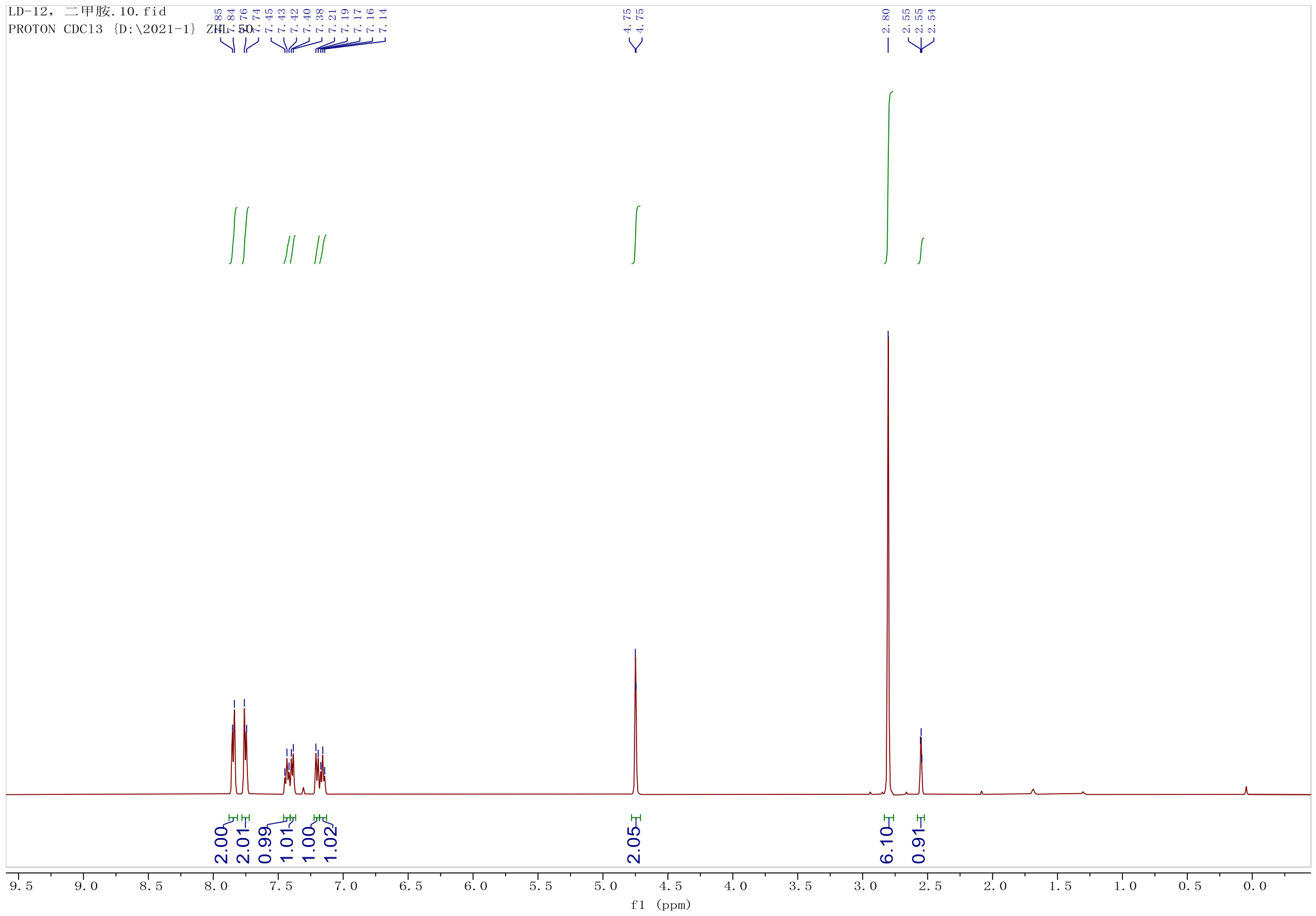

Fig. N19 ^{1}H NMR (500 MHz, CDCl3) spectra of product **4a-10**

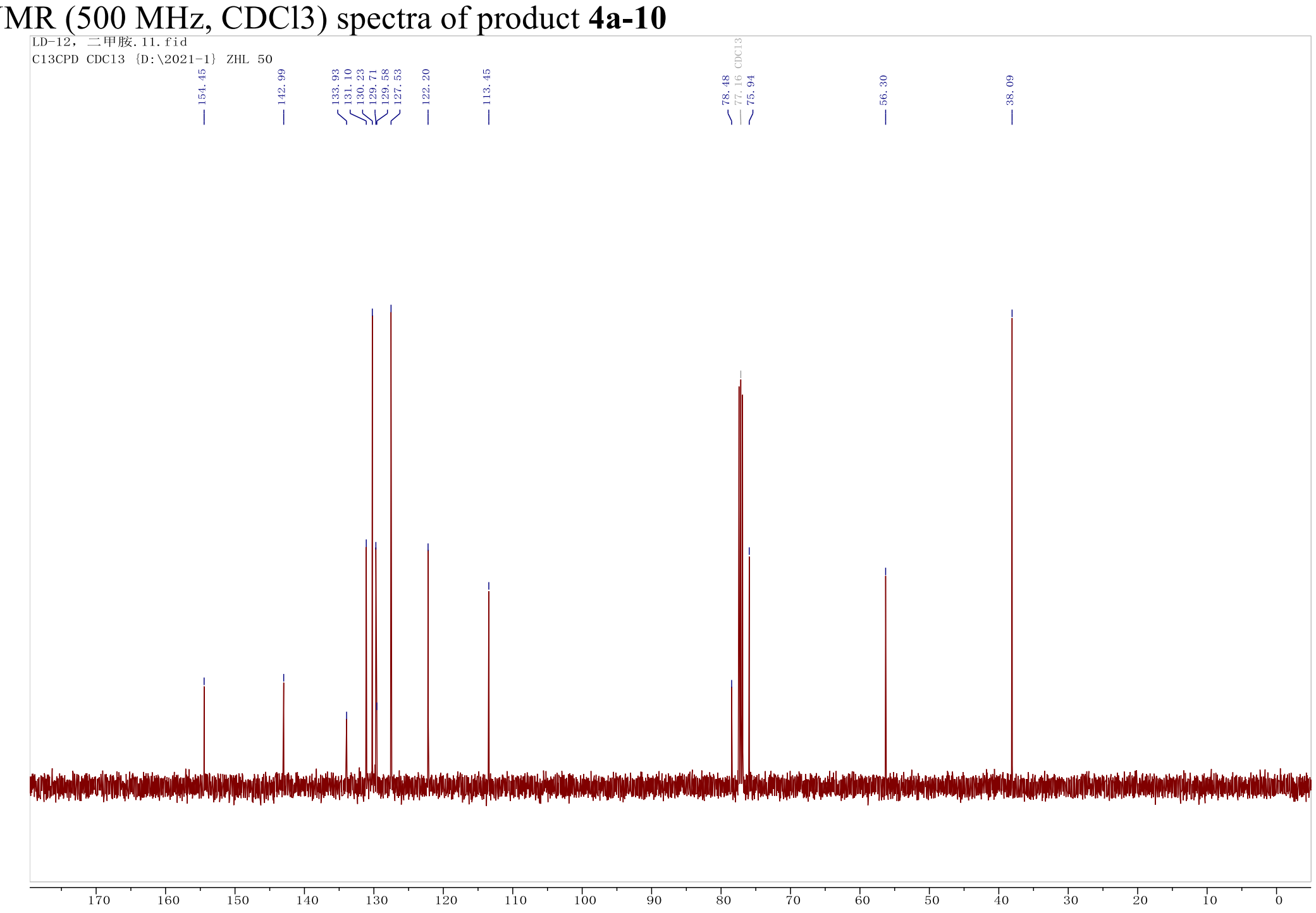

Fig. N20 ^{13}C NMR (126 MHz, CDCl3) spectra of product **4a-10**

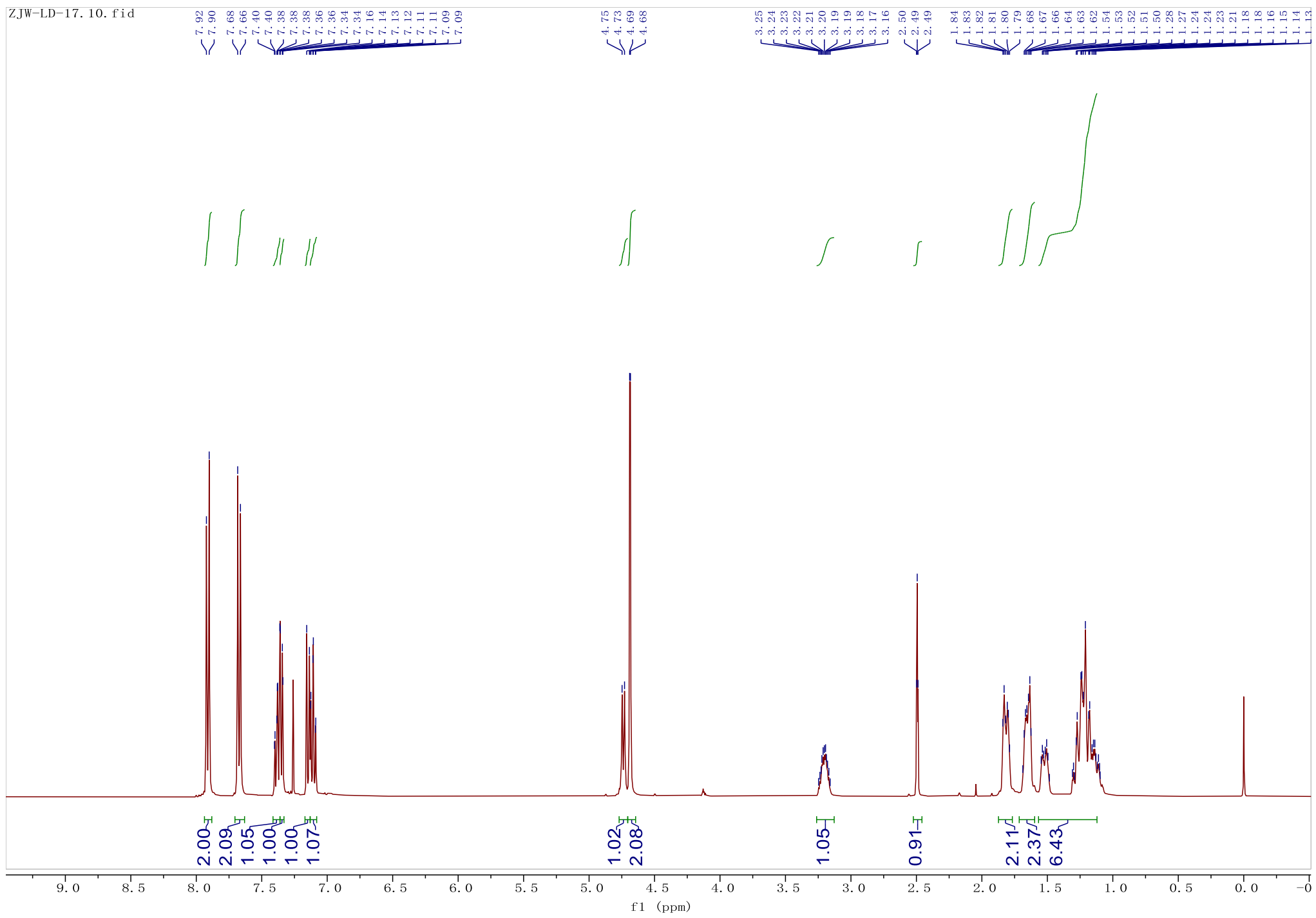

Fig. N21 ^{1}H NMR (400 MHz, CDCl3) spectra of product **4a-11**

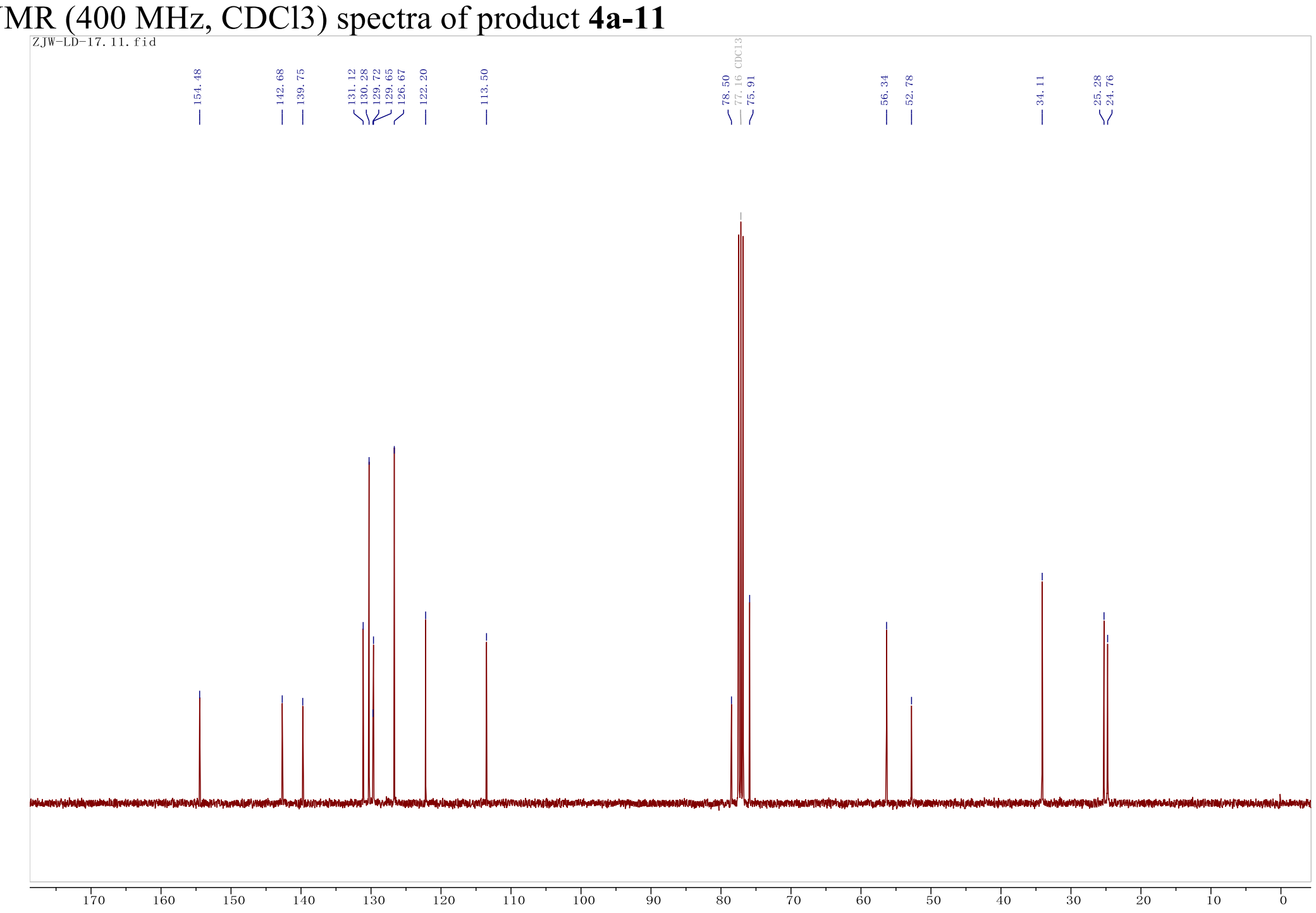

Fig. N22 ^{13}C NMR (101 MHz, CDCl3) spectra of product **4a-11**

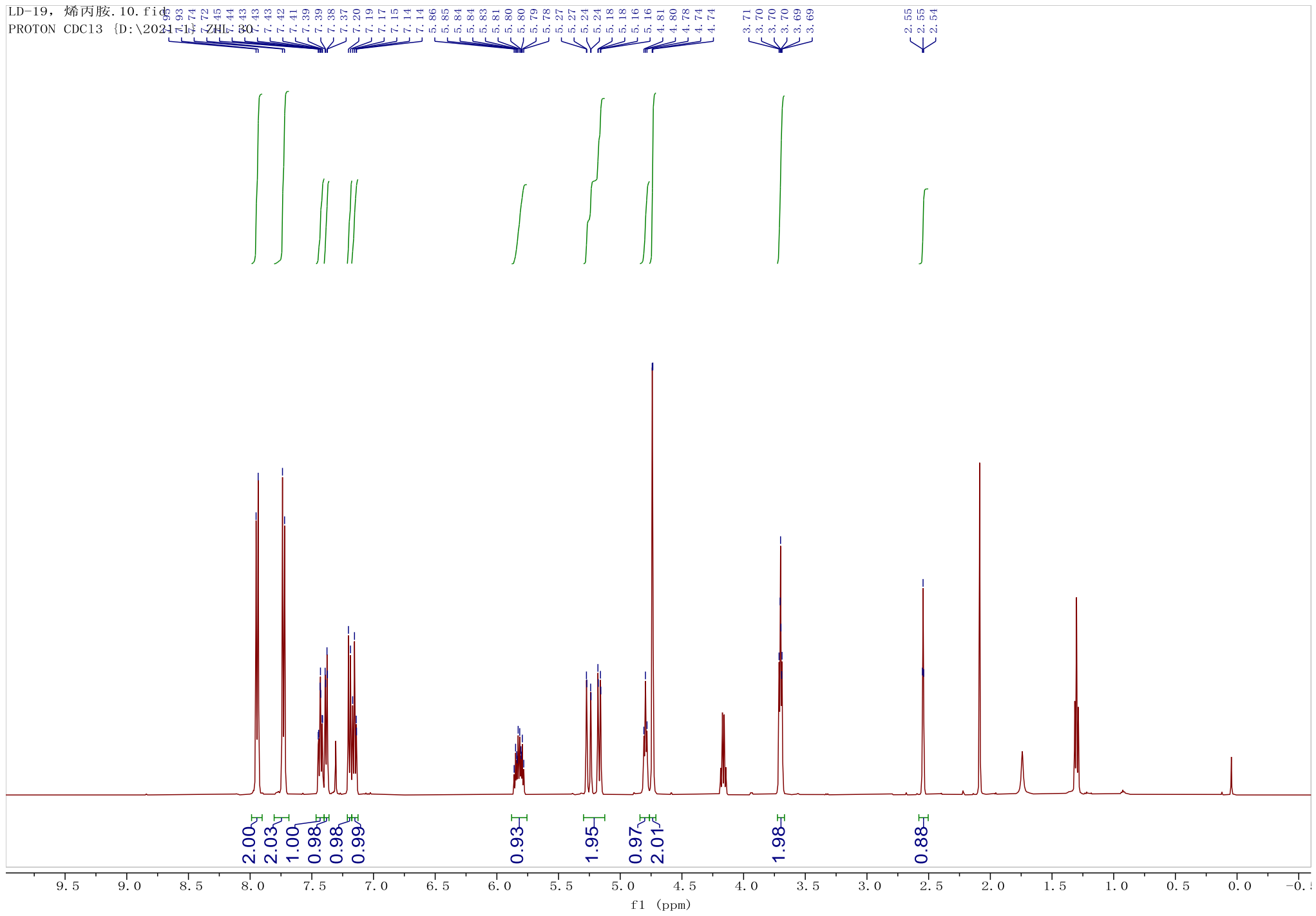

Fig. N23 ^{1}H NMR (500 MHz, CDCl3) spectra of product **4a-12**

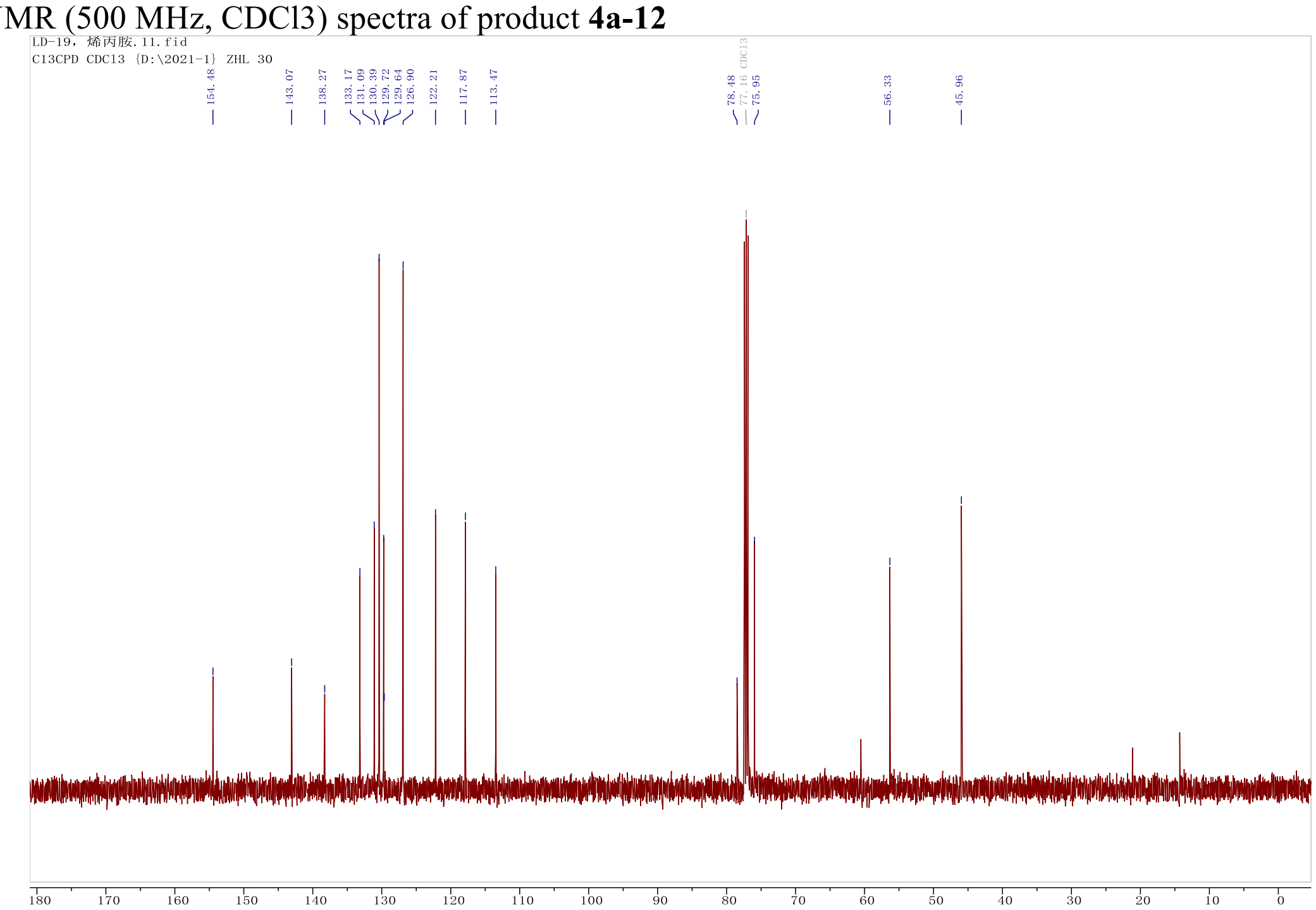

Fig. N24 ^{13}C NMR (126 MHz, CDCl3) spectra of product **4a-12**

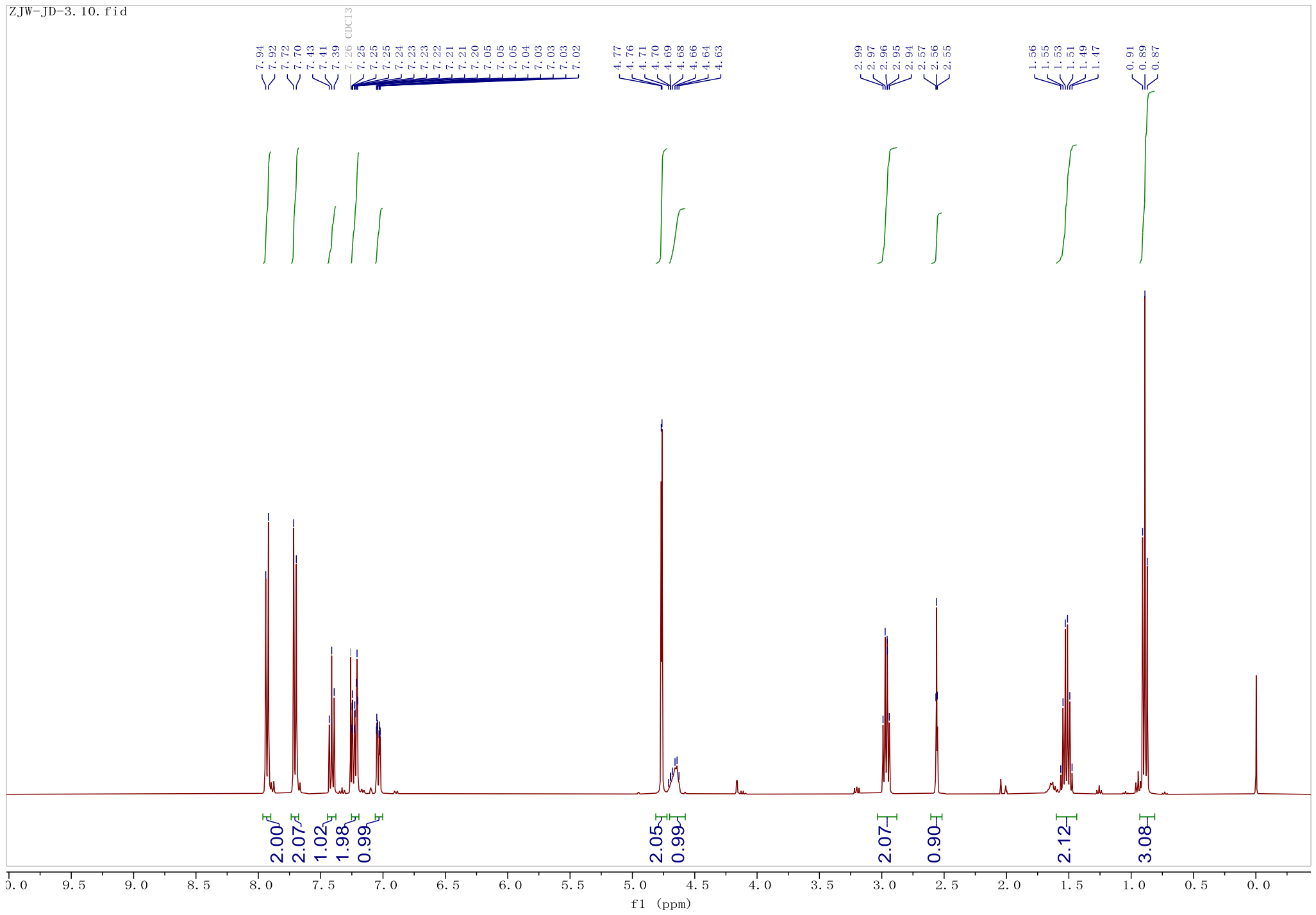

Fig. N25 ^{1}H NMR (400 MHz, CDCl3) spectra of product **4b-1**

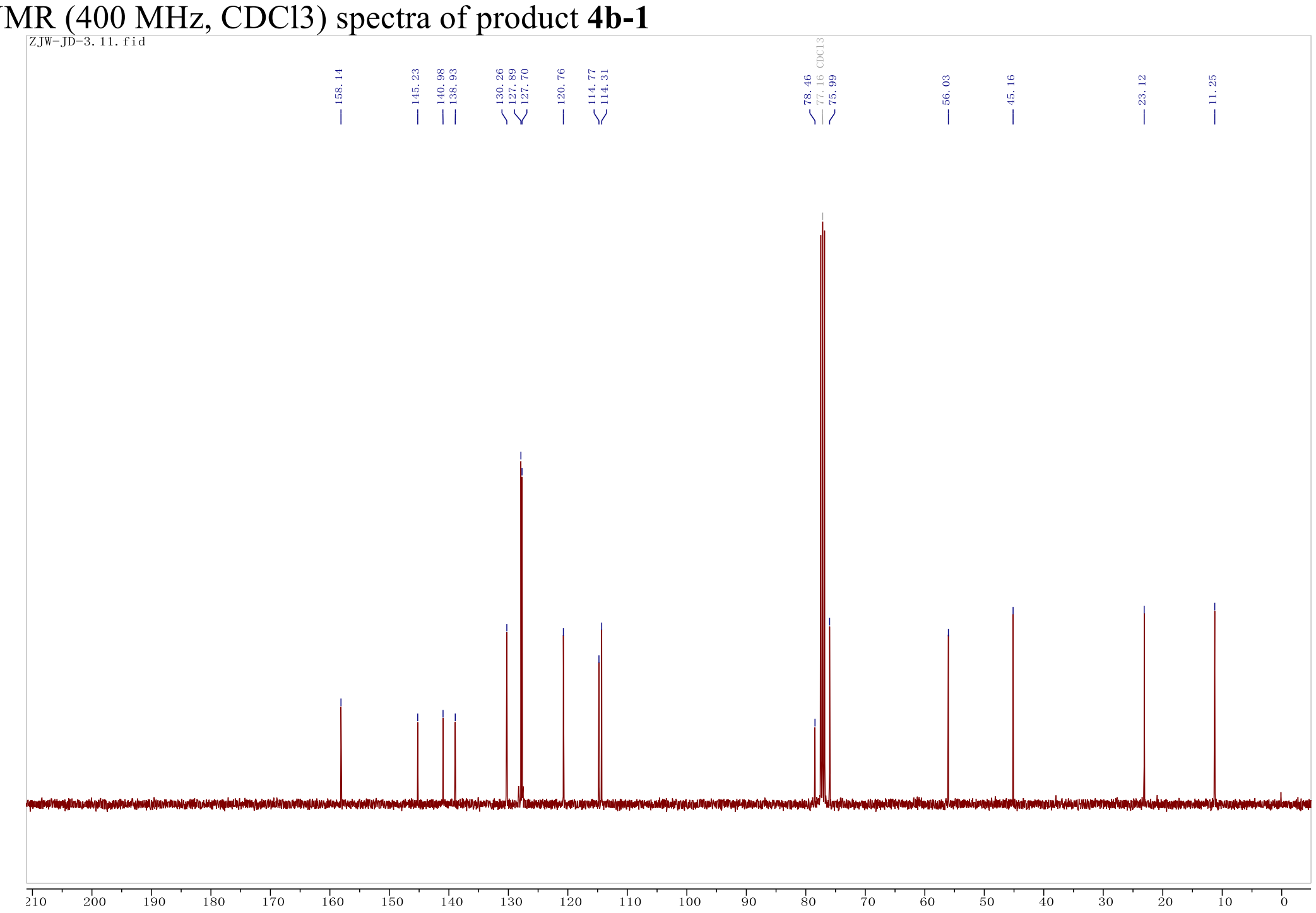

Fig. N26 ^{13}C NMR (101 MHz, CDCl3) spectra of product **4b-1**

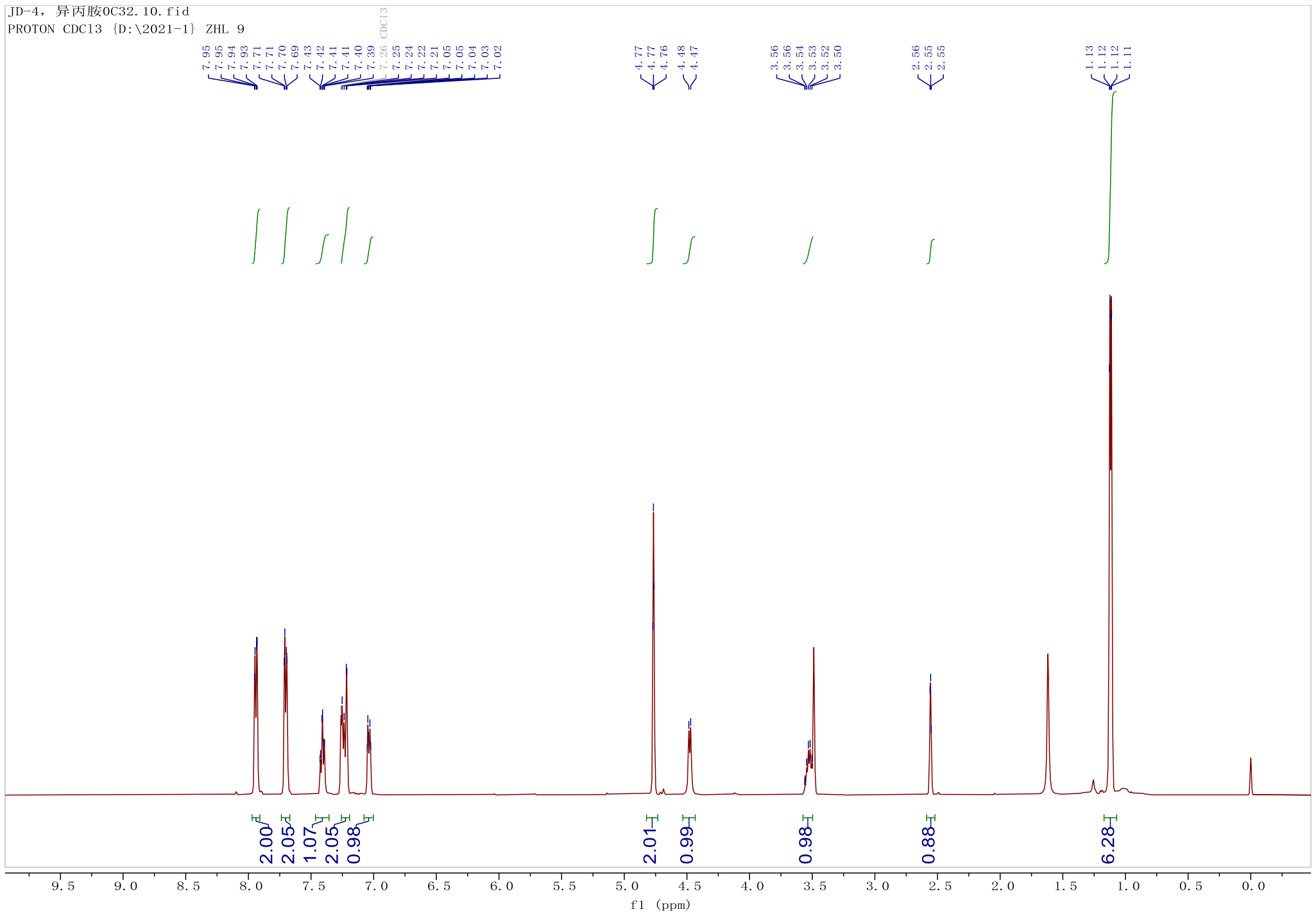

Fig. N27 ^{1}H NMR (500 MHz, CDCl3) spectra of product **4b-2**

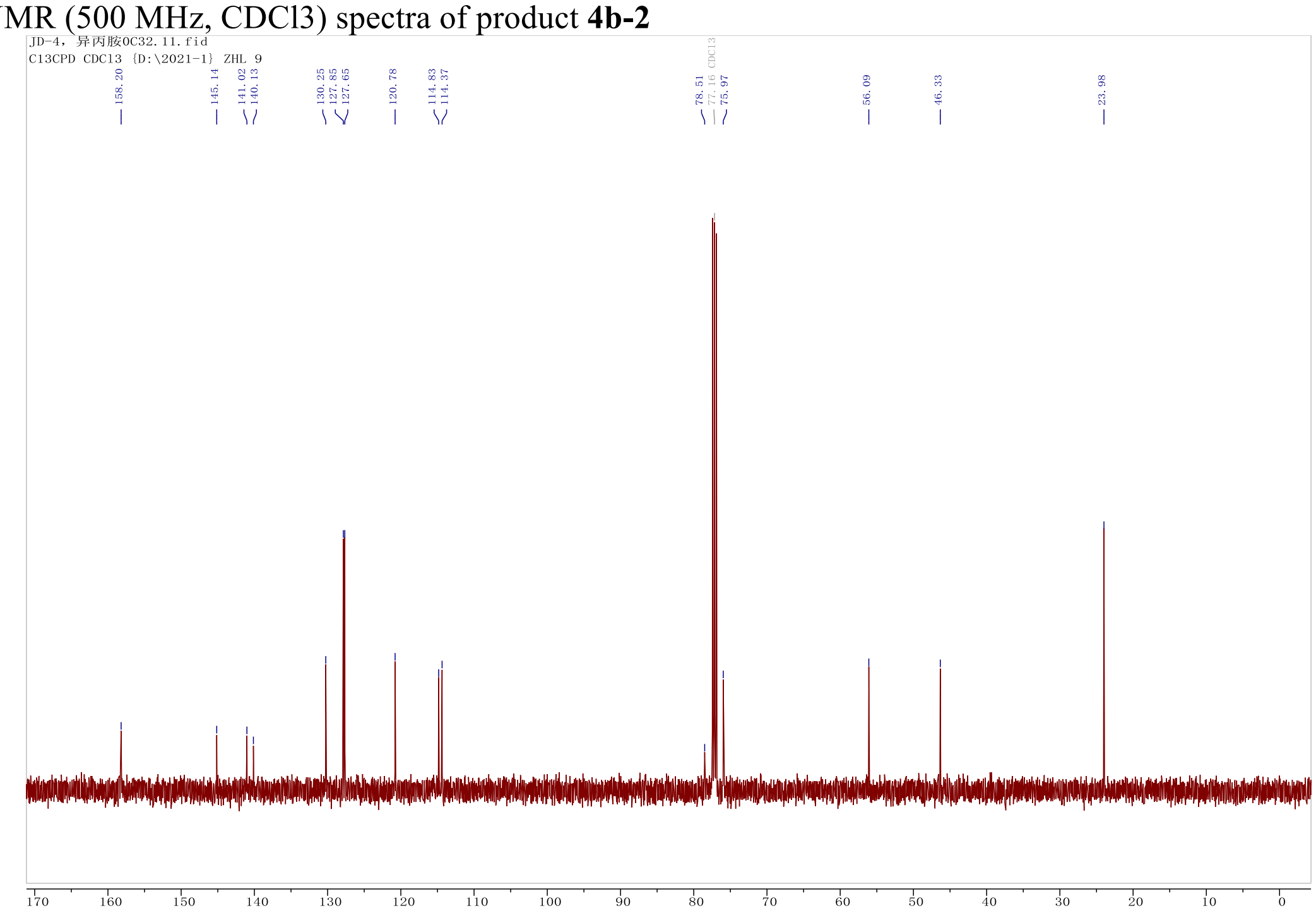

Fig. N28 ^{13}C NMR (126 MHz, CDCl3) spectra of product **4b-2**

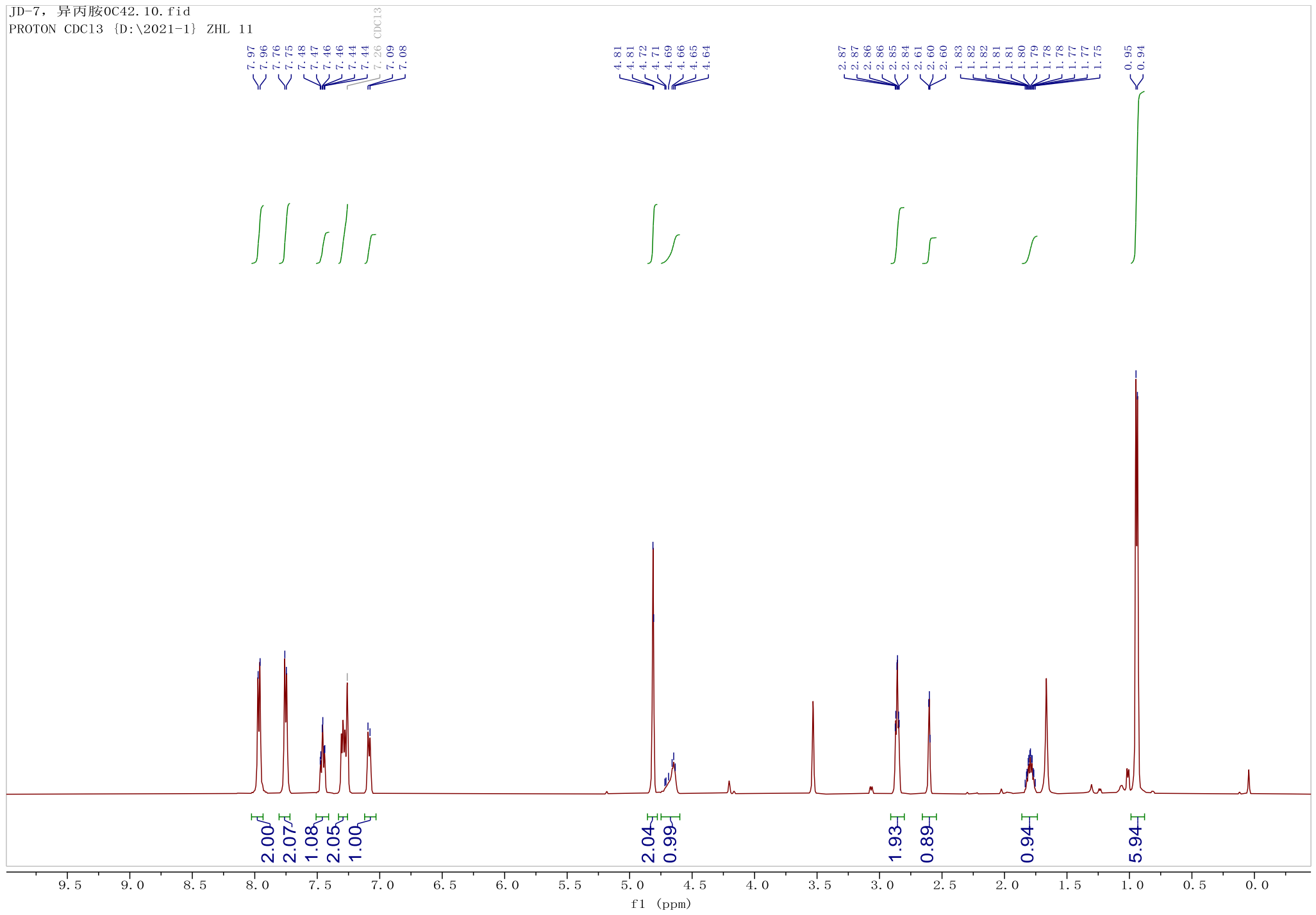

Fig. N29 ^{1}H NMR (500 MHz, CDCl3) spectra of product **4b-3**

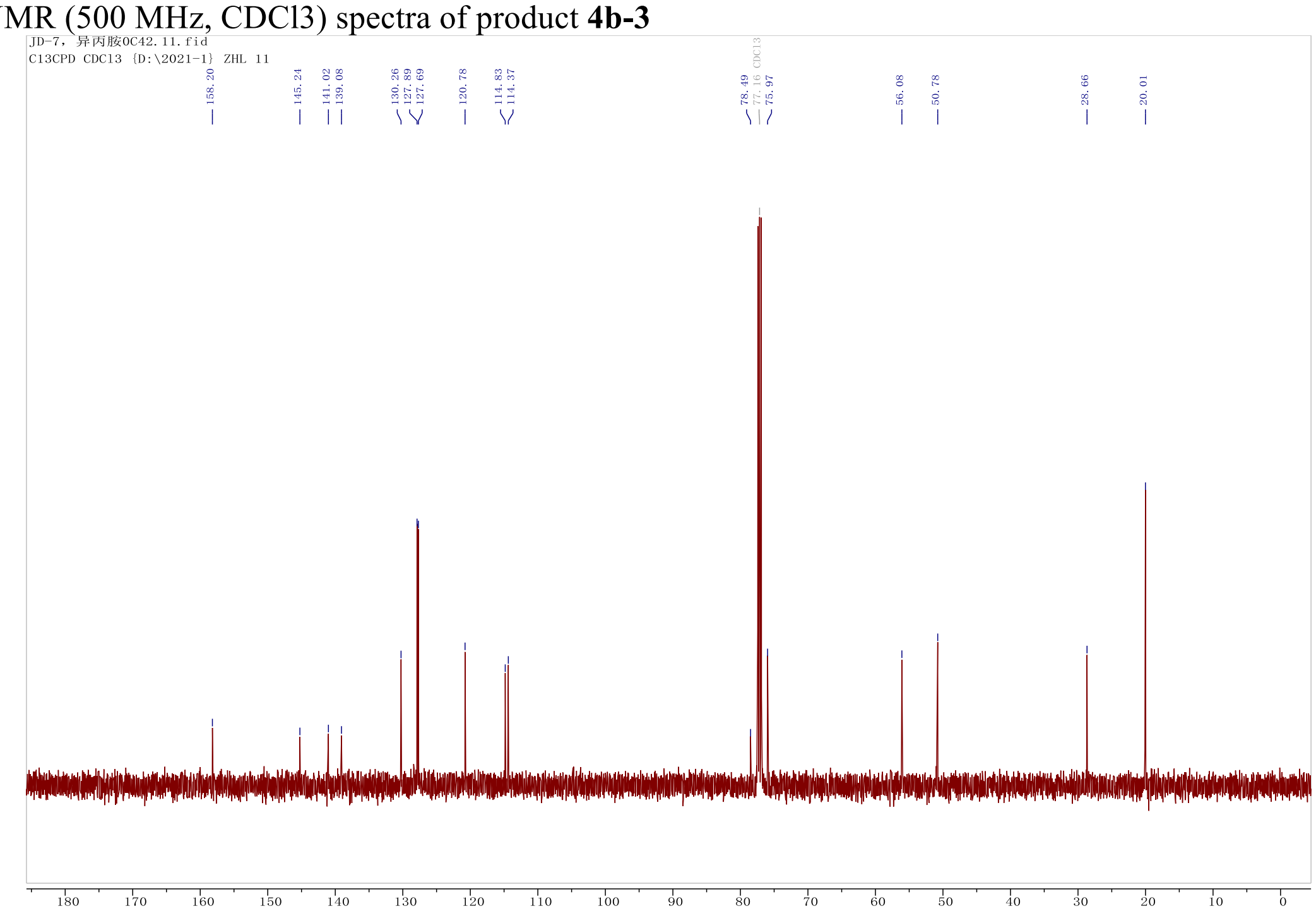

Fig. N30 ^{13}C NMR (126 MHz, CDCl3) spectra of product **4b-3**

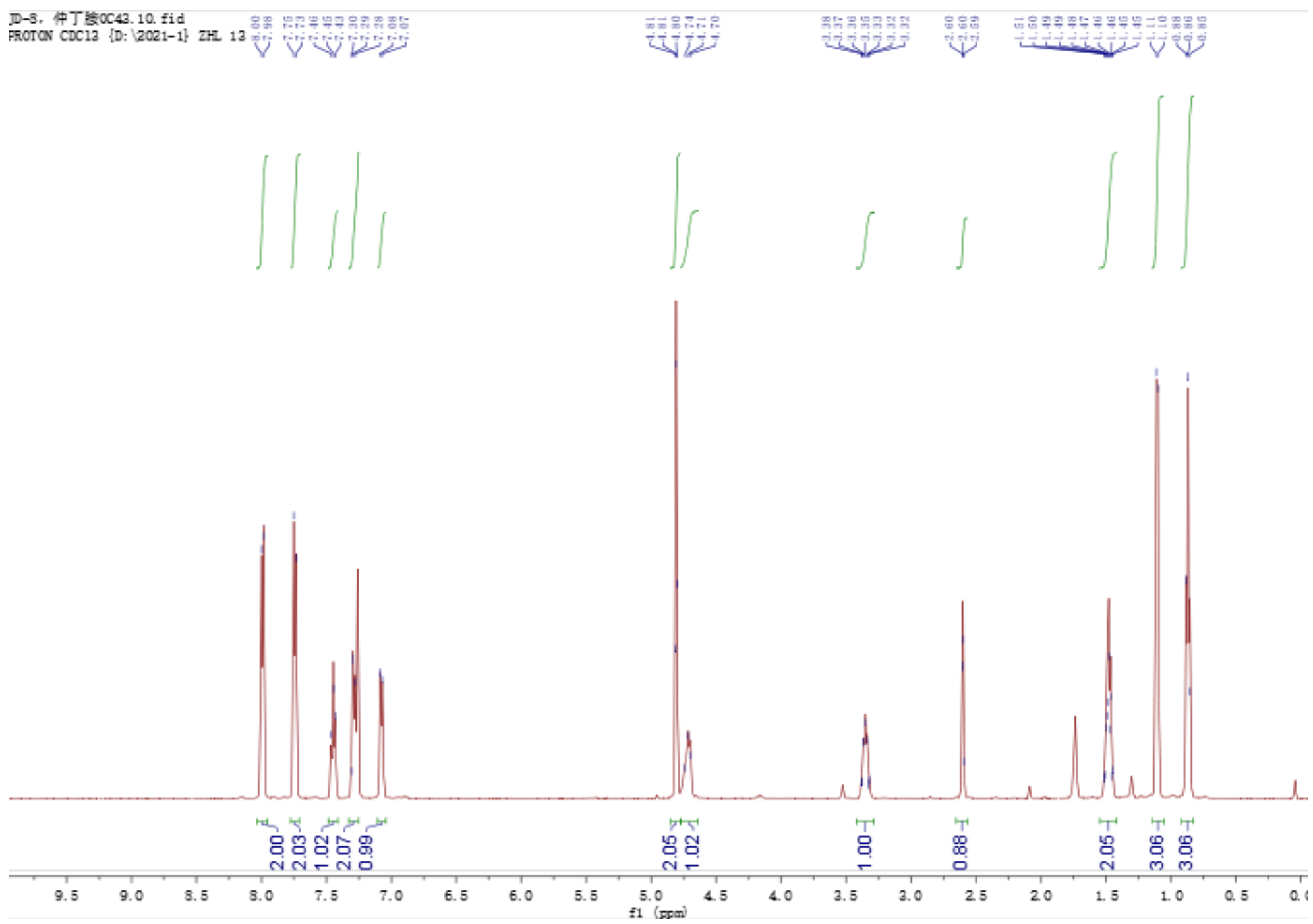

Fig. N31 ^{1}H NMR (500 MHz, CDCl3) spectra of product **4b-4**

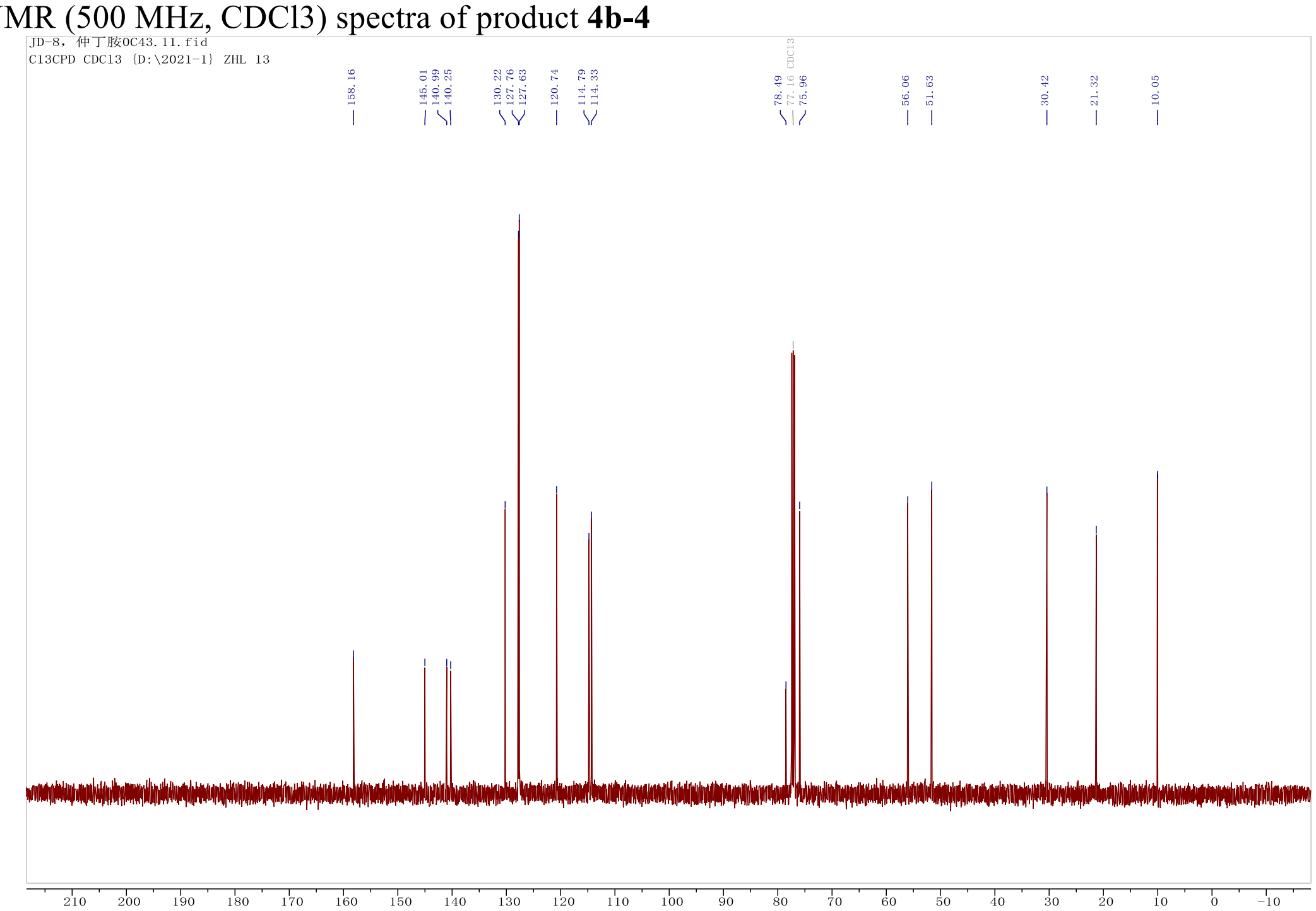

Fig. N32 ^{13}C NMR (126 MHz, CDCl3) spectra of product **4b-4**

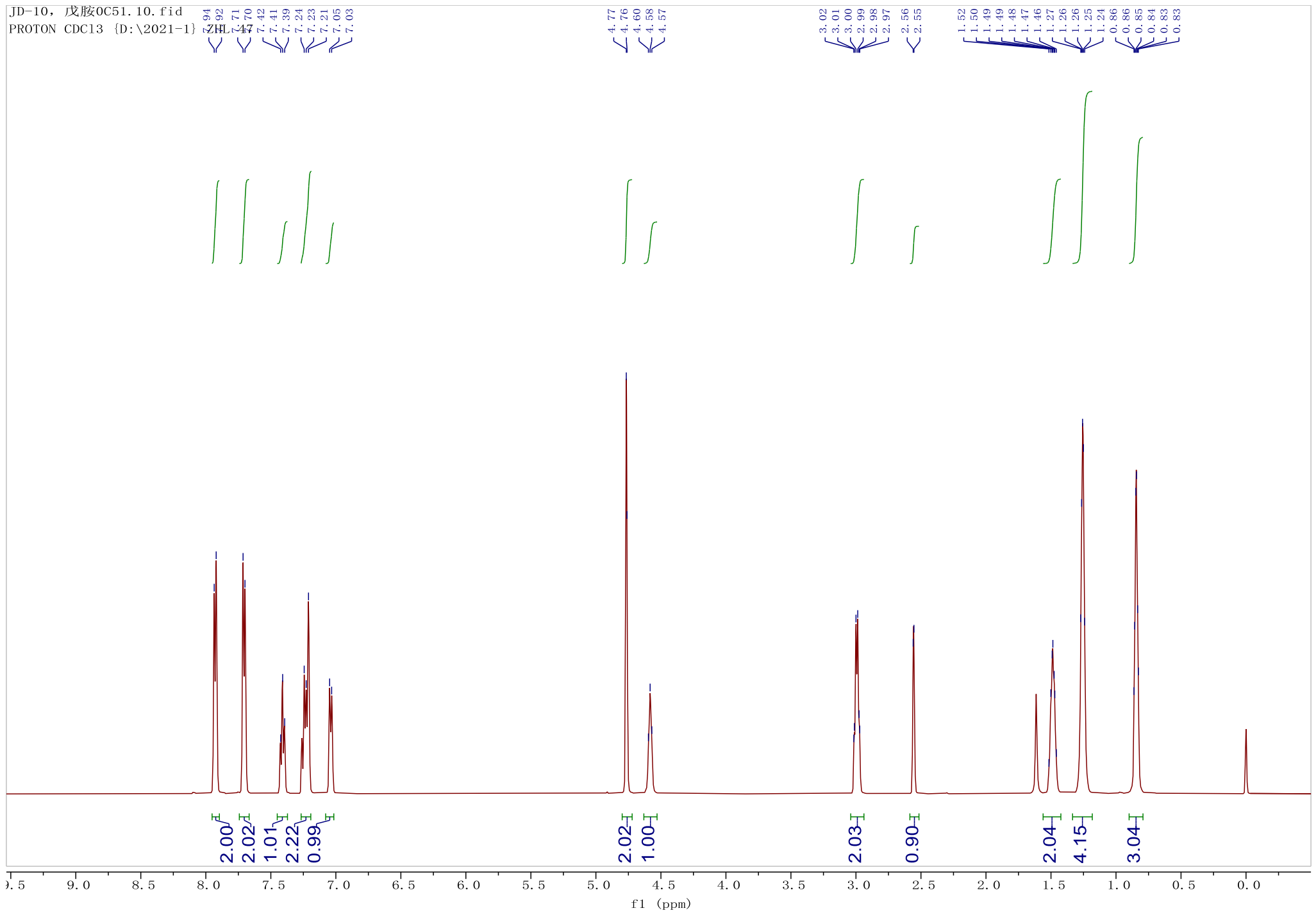

Fig. N33 ^{1}H NMR (500 MHz, CDCl3) spectra of product **4b-5**

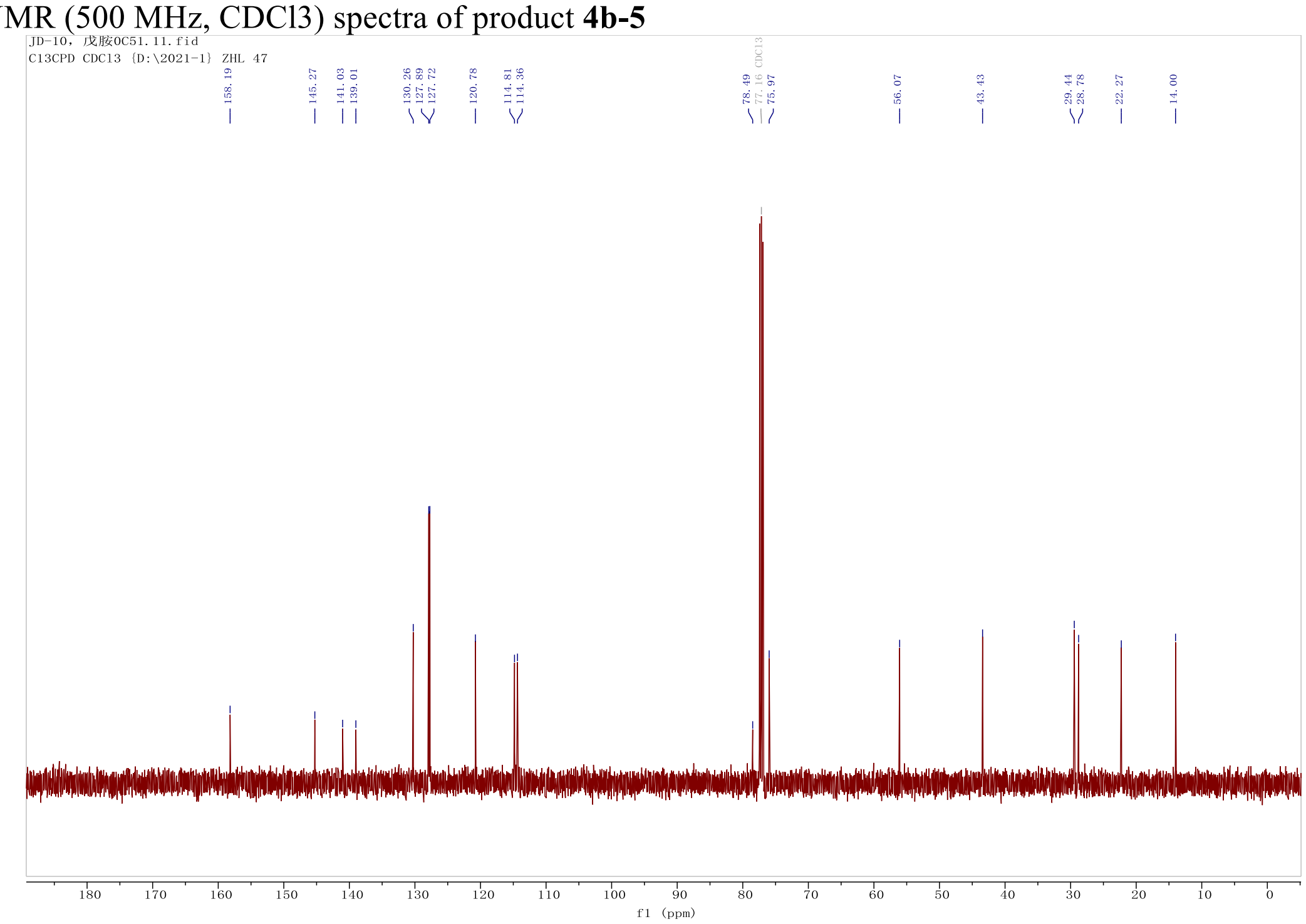

Fig. N34 ^{13}C NMR (126 MHz, CDCl3) spectra of product **4b-5**

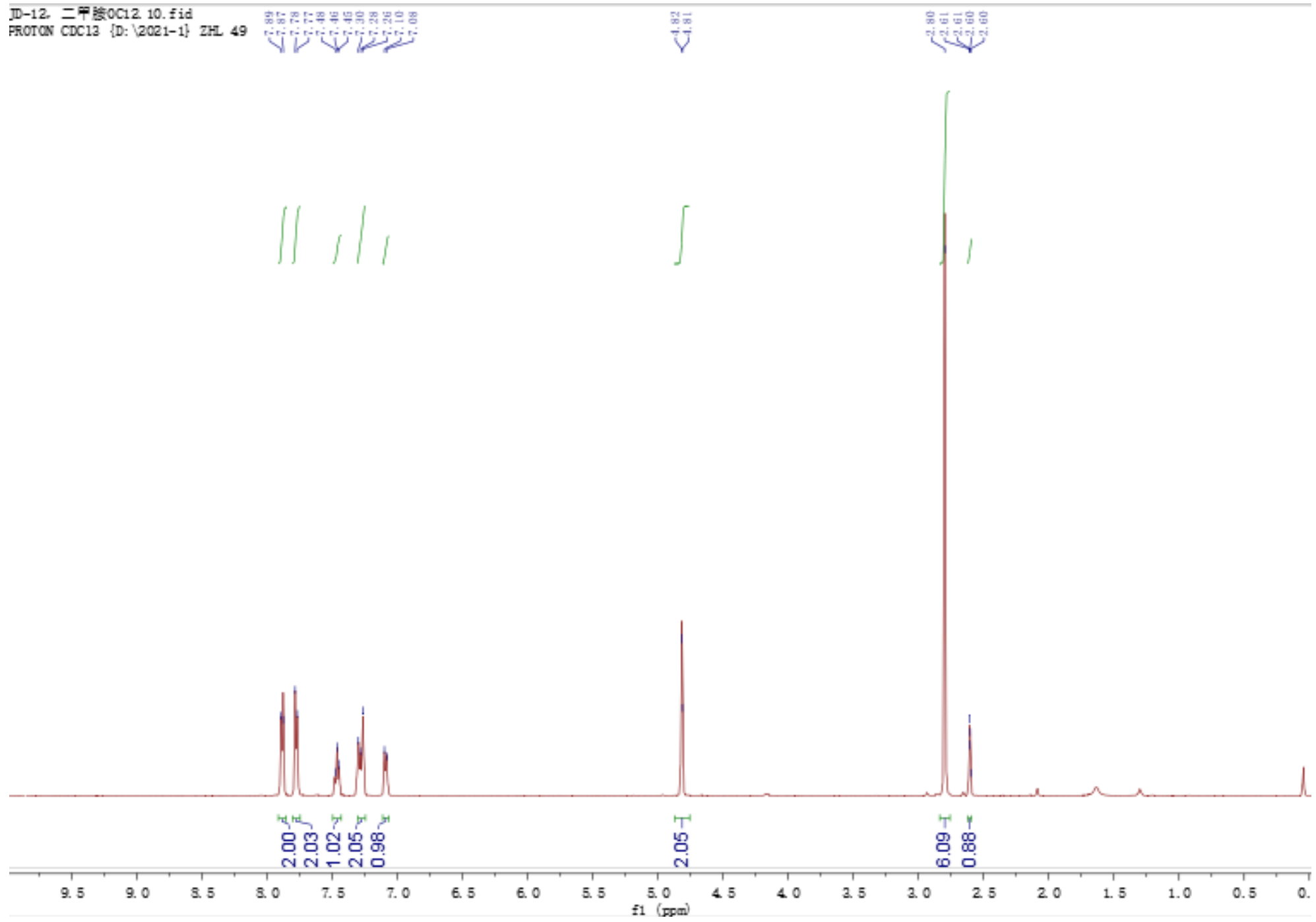

Fig. N35 ^{1}H NMR (500 MHz, CDCl3) spectra of product **4b-6**

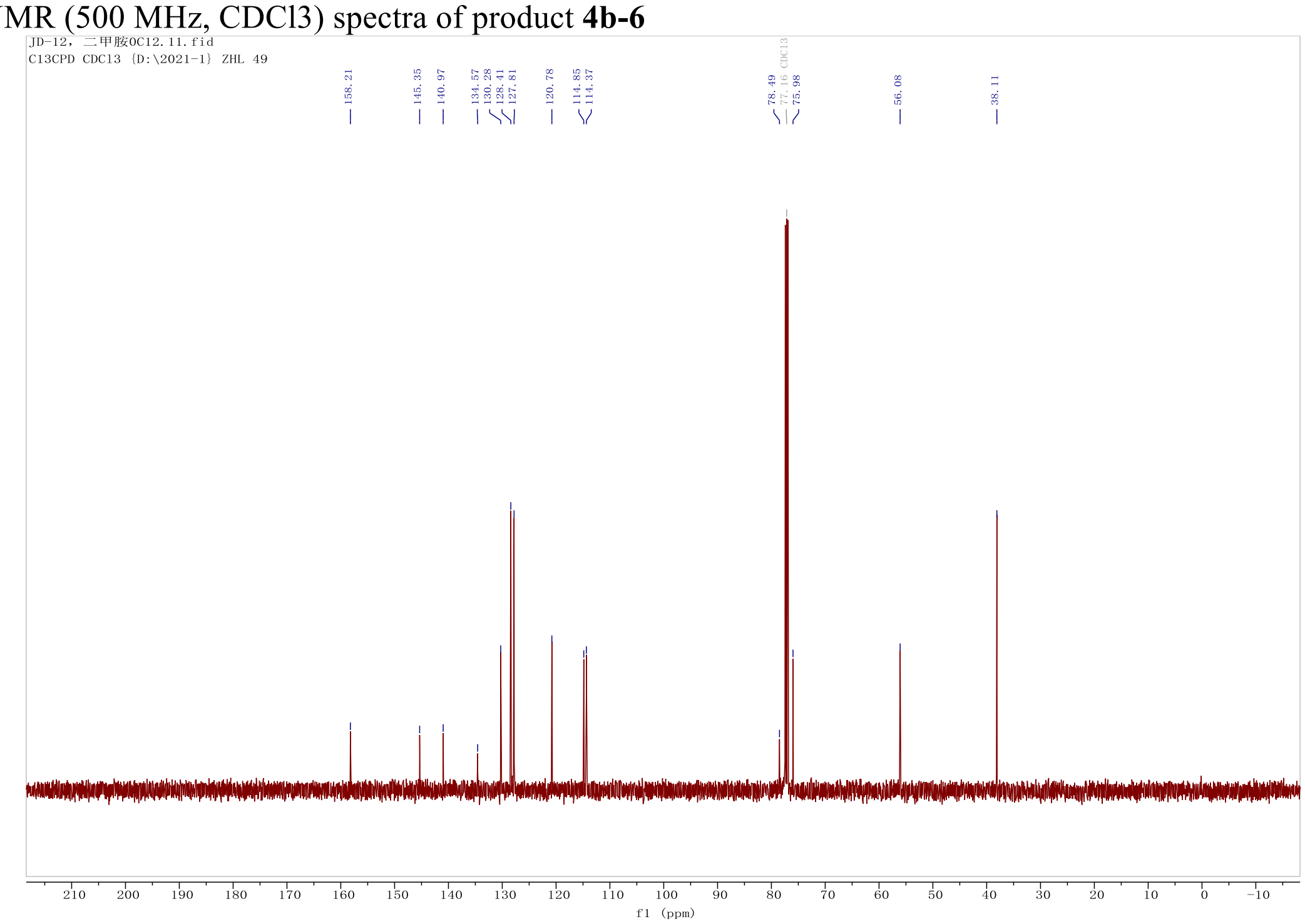

Fig. N36 ^{13}C NMR (126 MHz, CDCl3) spectra of product **4b-6**

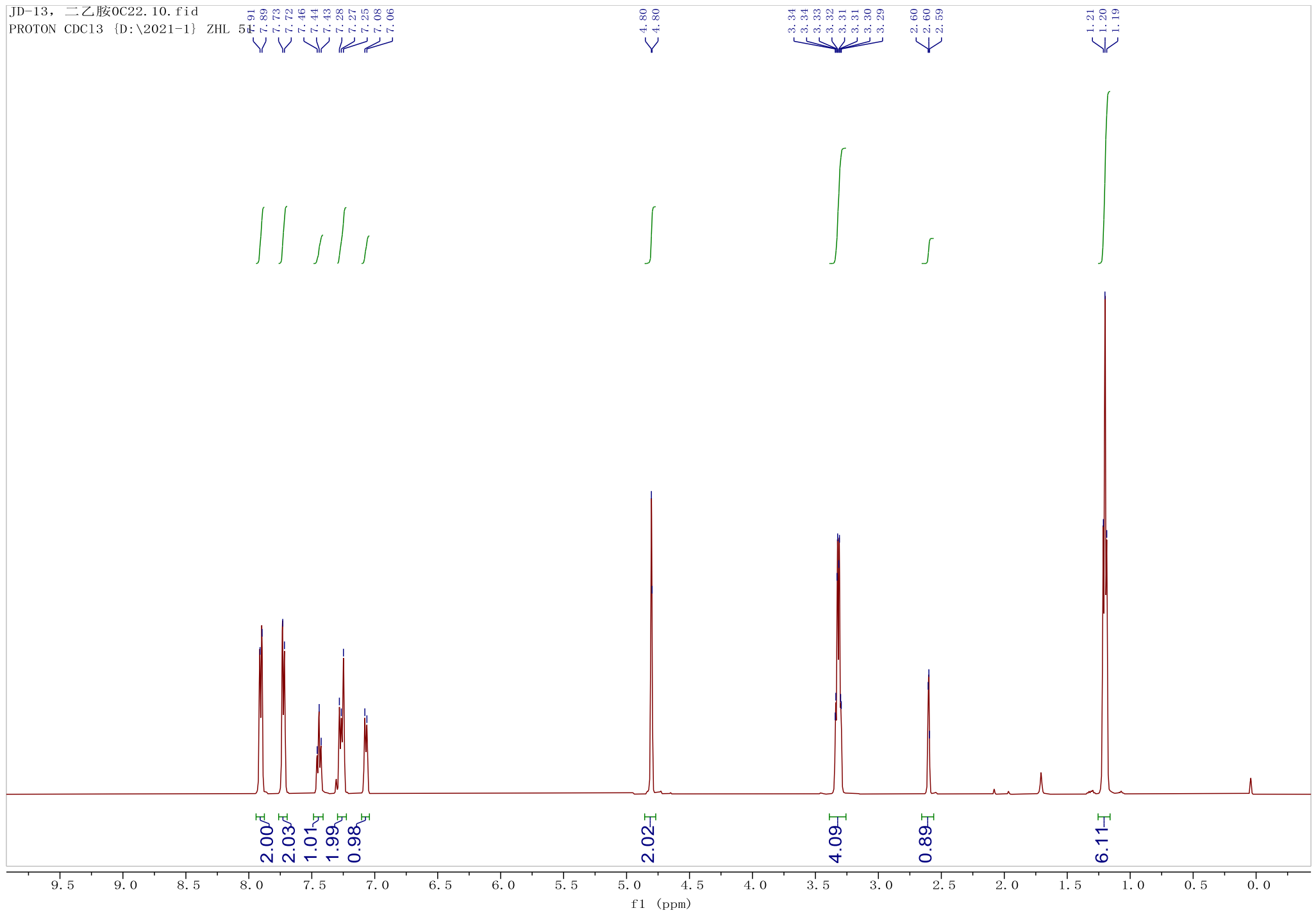

Fig. N37 ^{1}H NMR (500 MHz, CDCl3) spectra of product **4b-7**

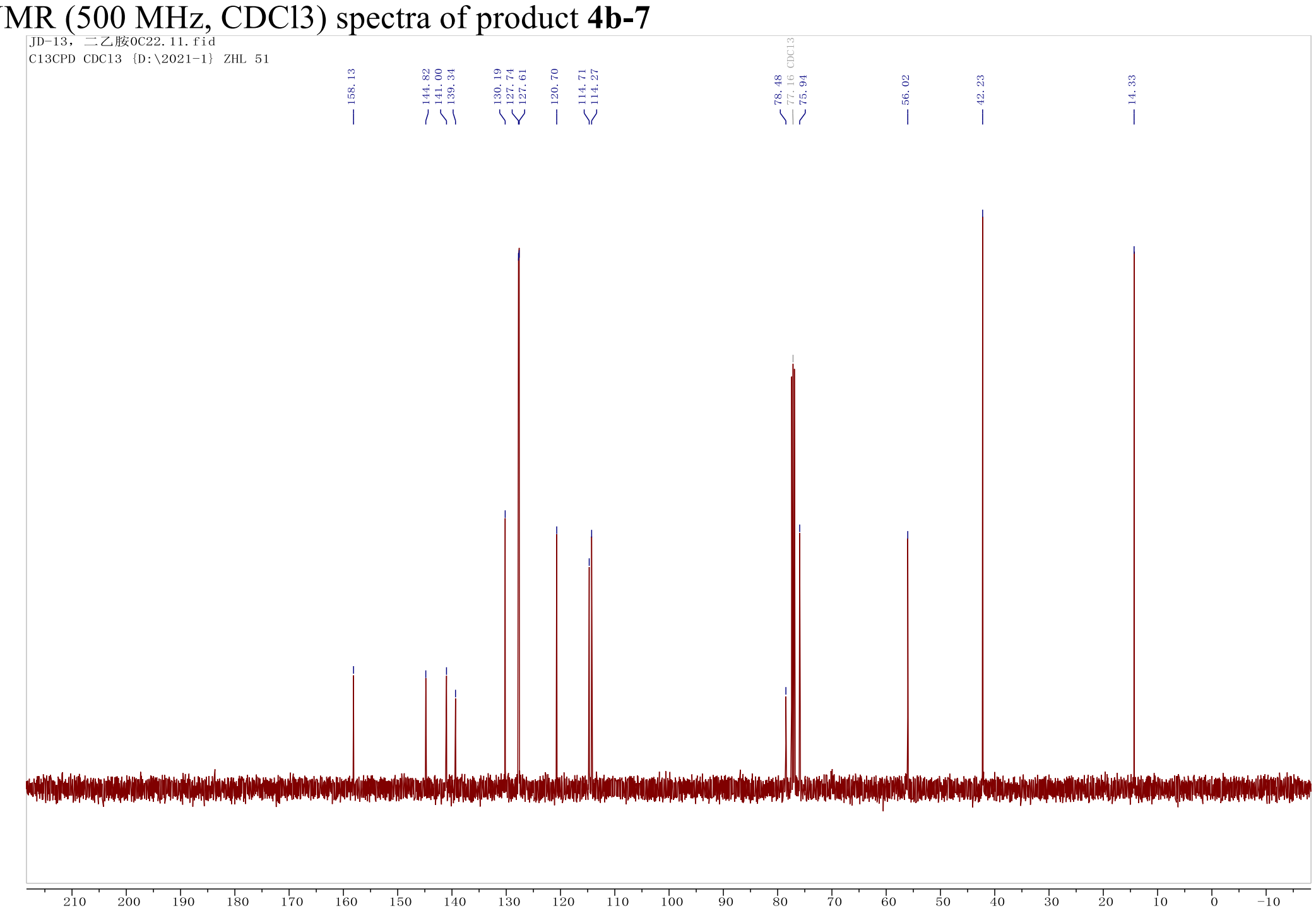

Fig. N38 ^{13}C NMR (126 MHz, CDCl3) spectra of product **4b-7**

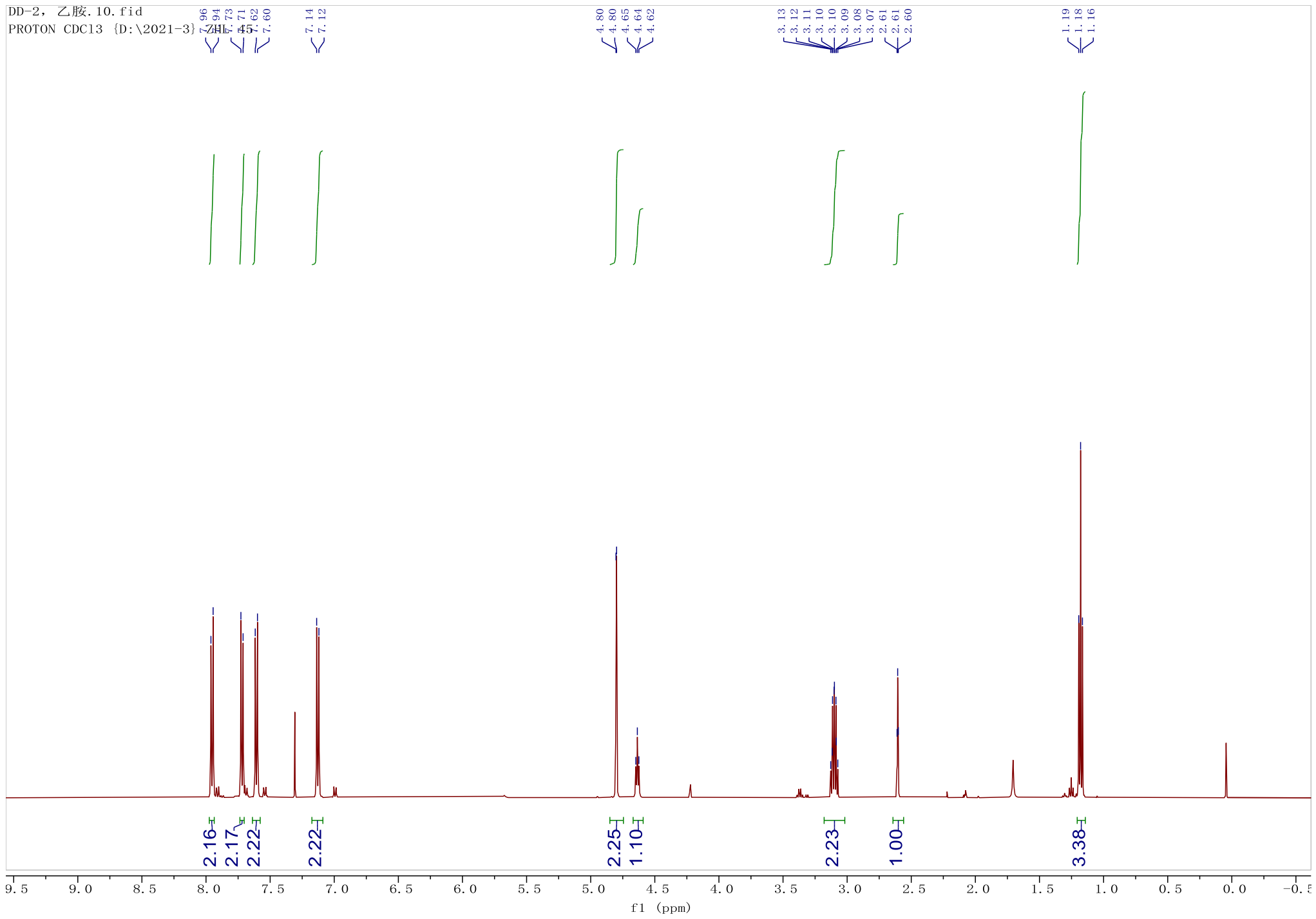

Fig. N39 ^{1}H NMR (500 MHz, CDCl3) spectra of product **4c-1**

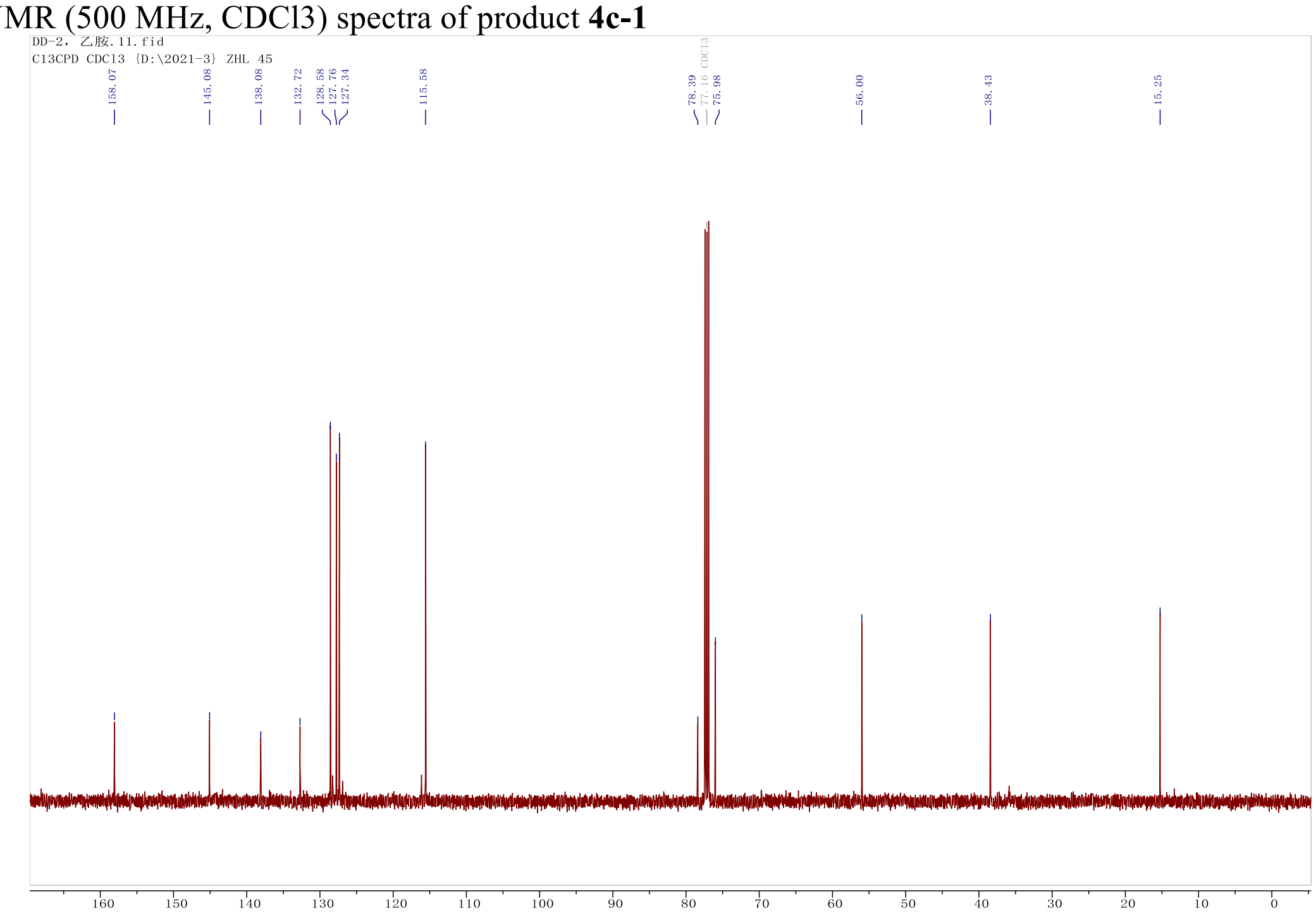

Fig. N40 ^{13}C NMR (126 MHz, CDCl3) spectra of product **4c-1**

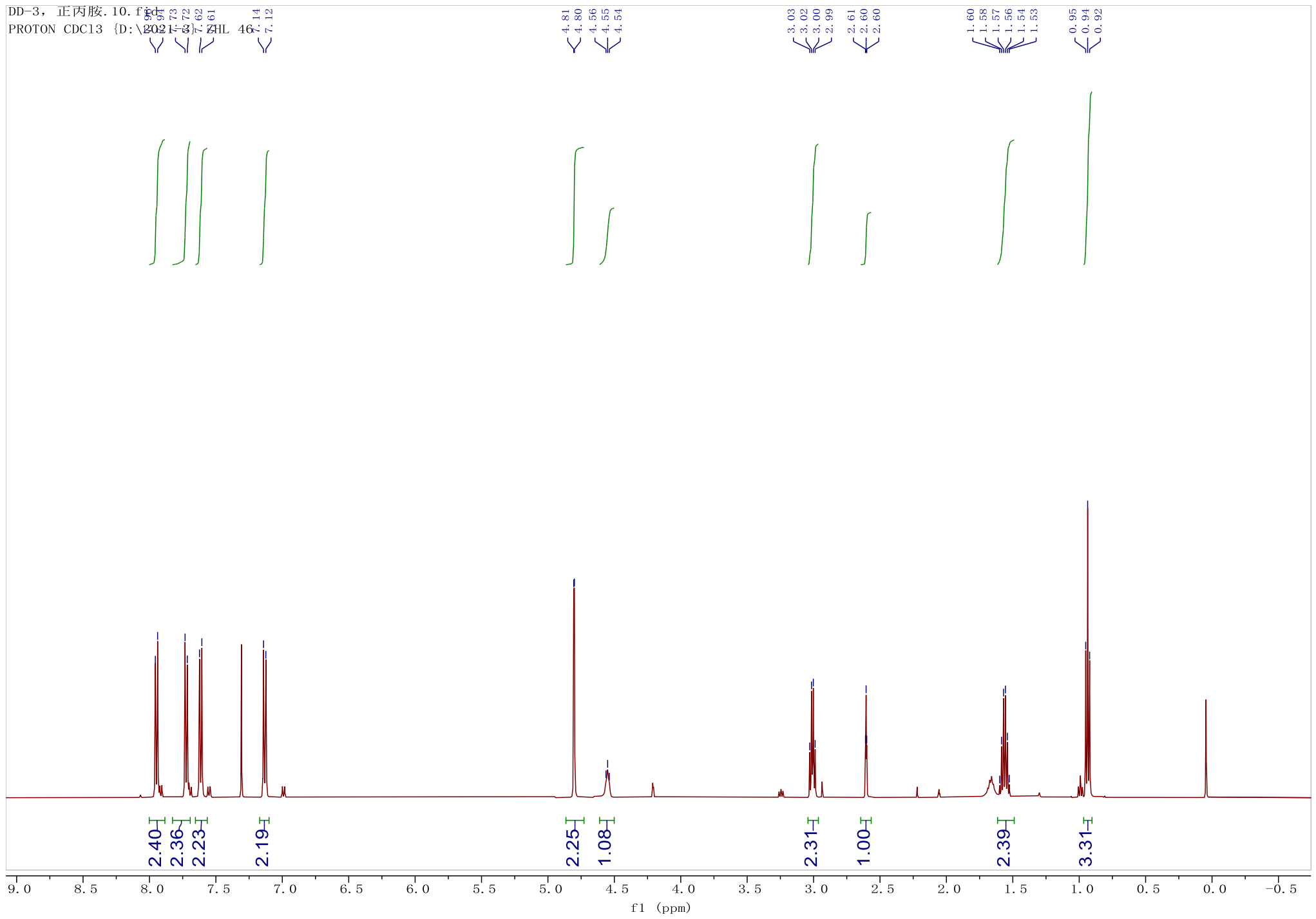

Fig. N41 ^{1}H NMR (500 MHz, CDCl3) spectra of product **4c-2**

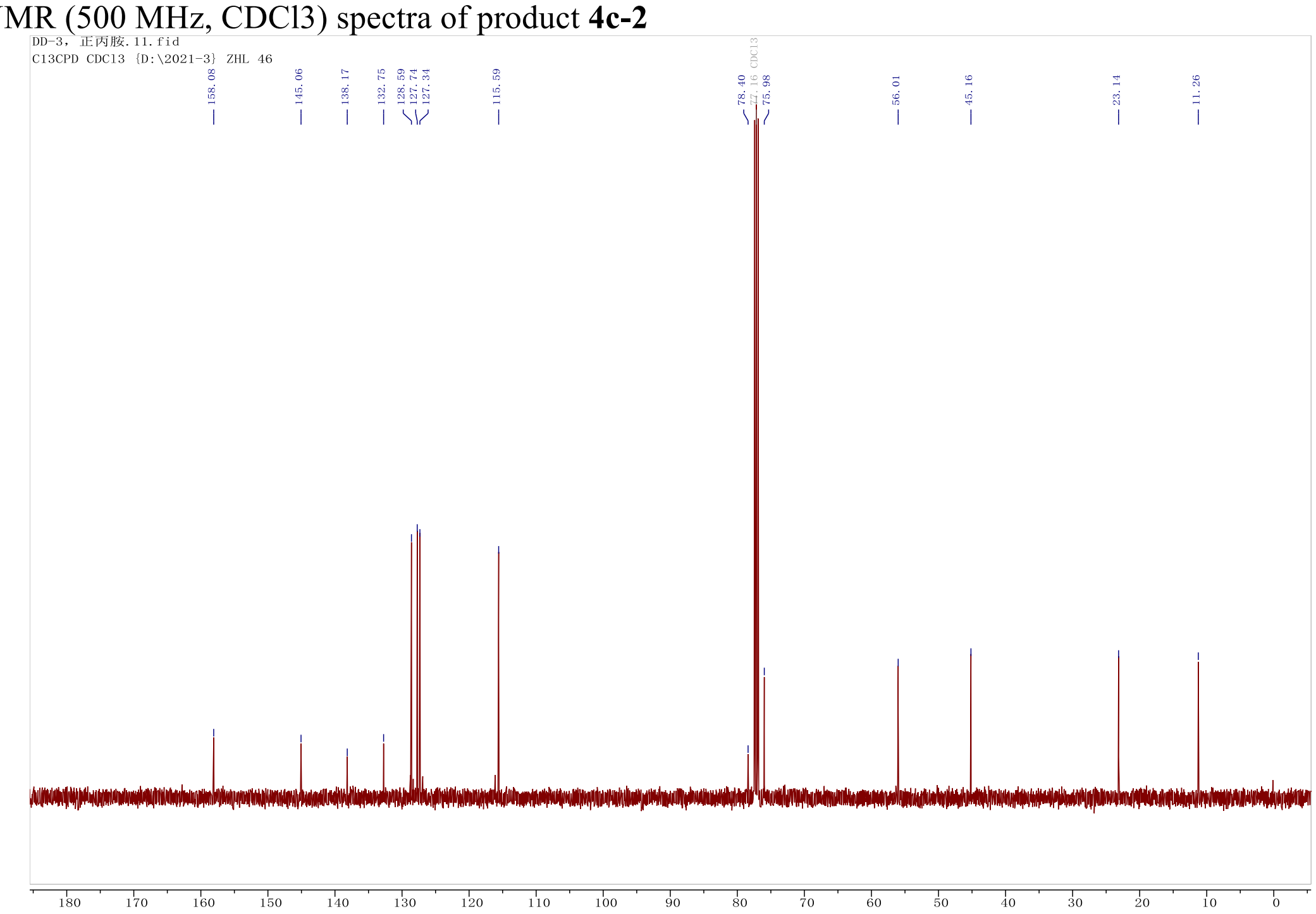

Fig. N42 ^{13}C NMR (126 MHz, CDCl3) spectra of product **4c-2**

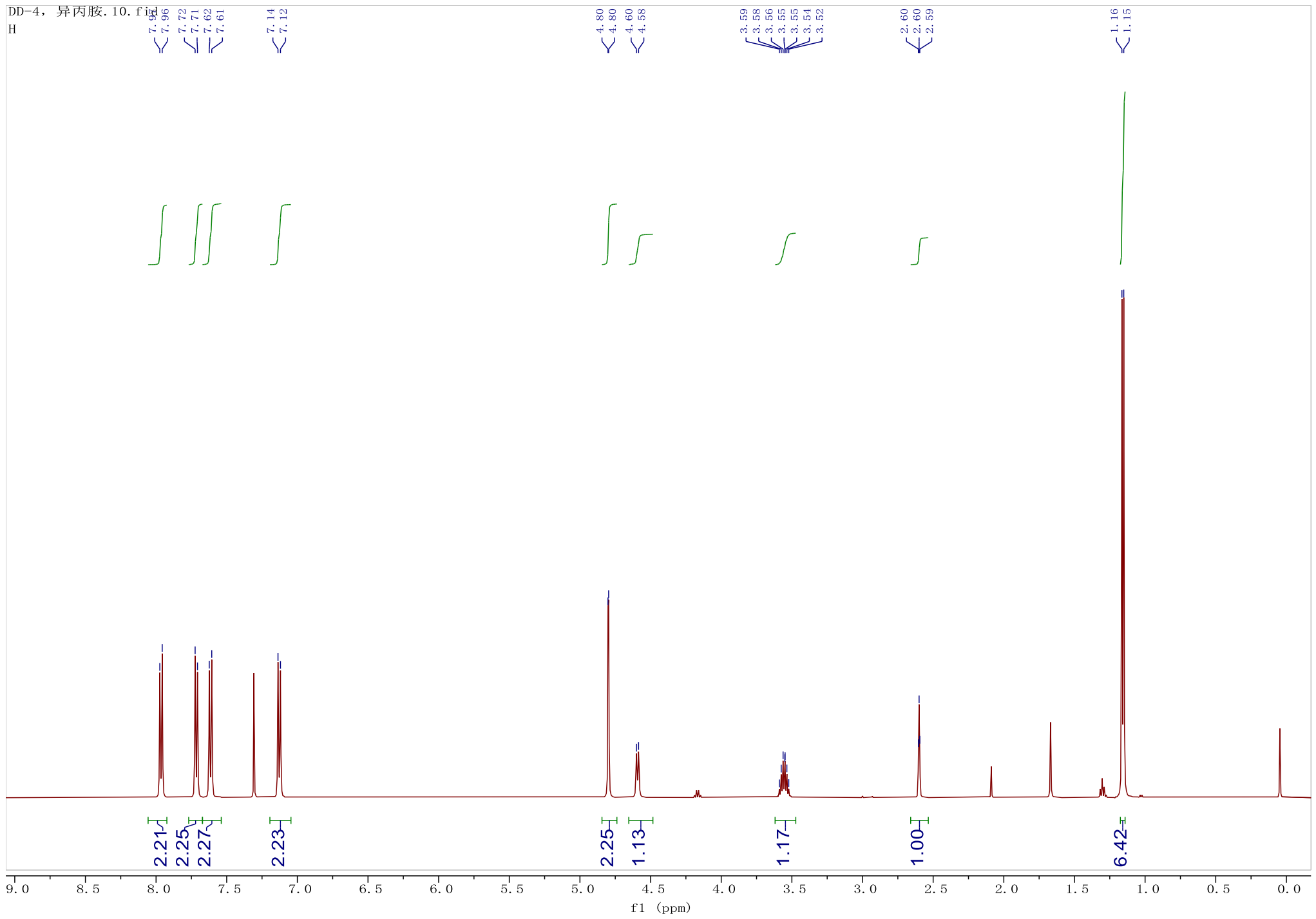

Fig. N43 ^{1}H NMR (500 MHz, CDCl3) spectra of product **4c-3**

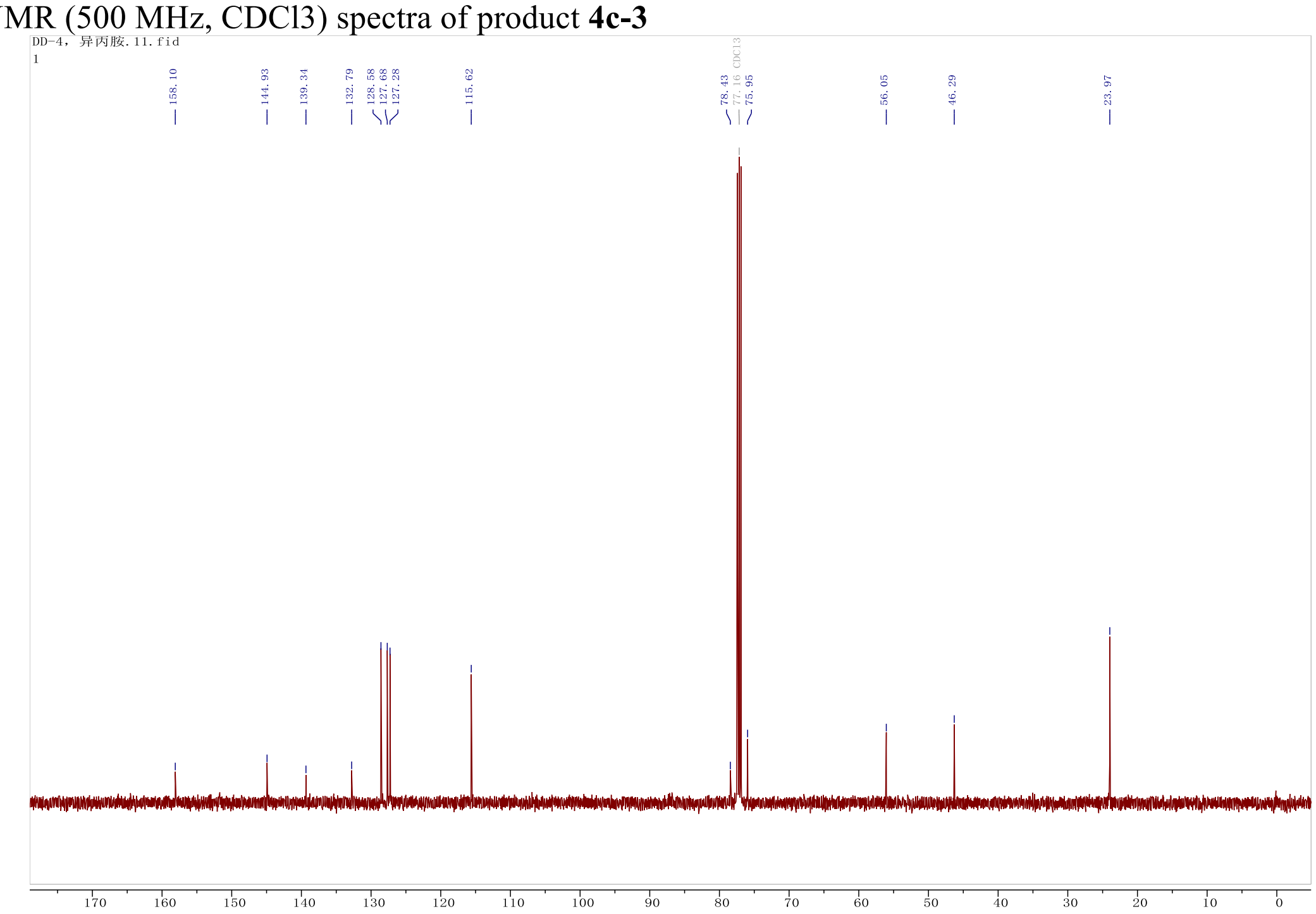

Fig. N44 ^{13}C NMR (126 MHz, CDCl3) spectra of product **4c-3**

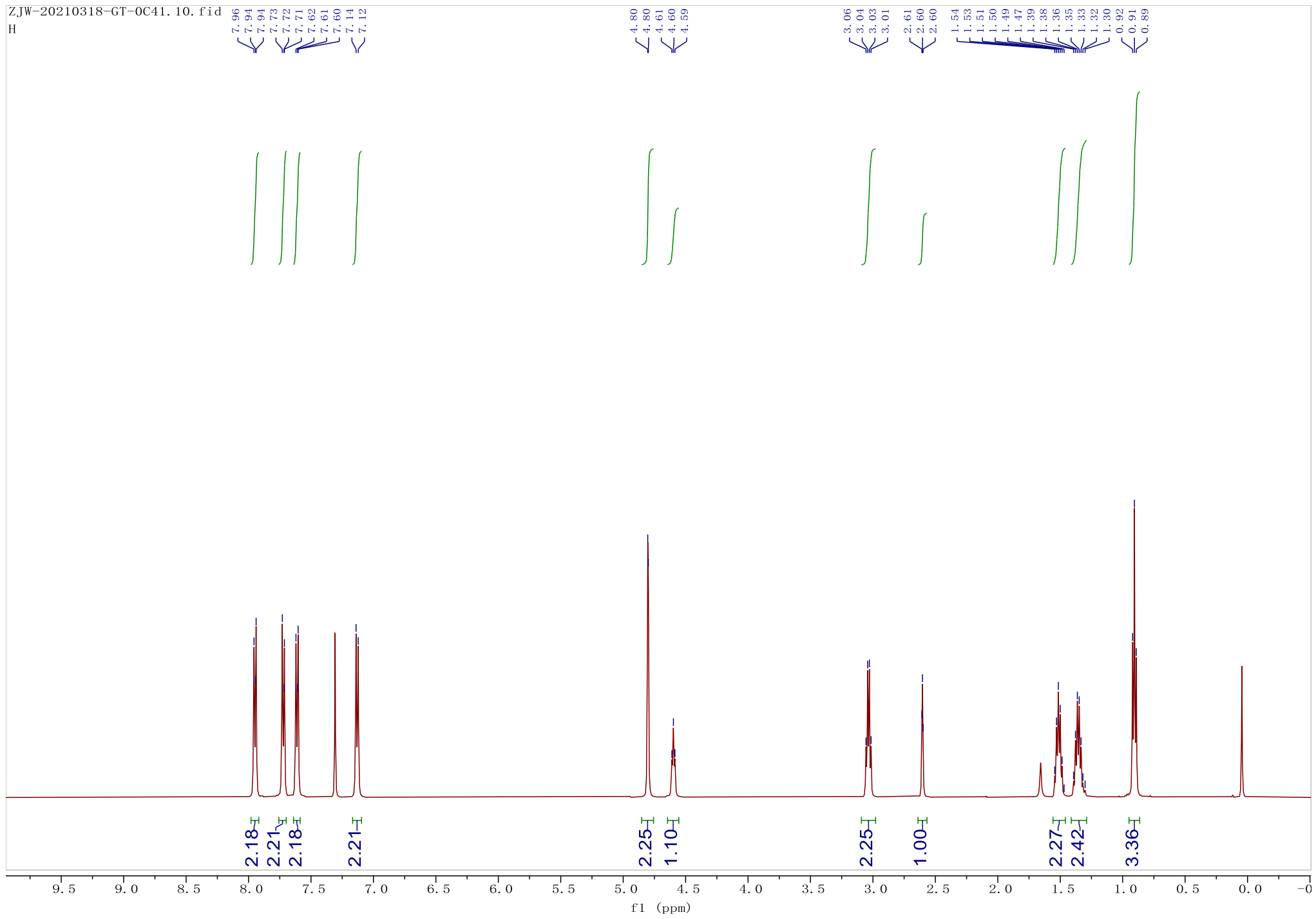

Fig. N45 ^{1}H NMR (500 MHz, CDCl3) spectra of product **4c-4**

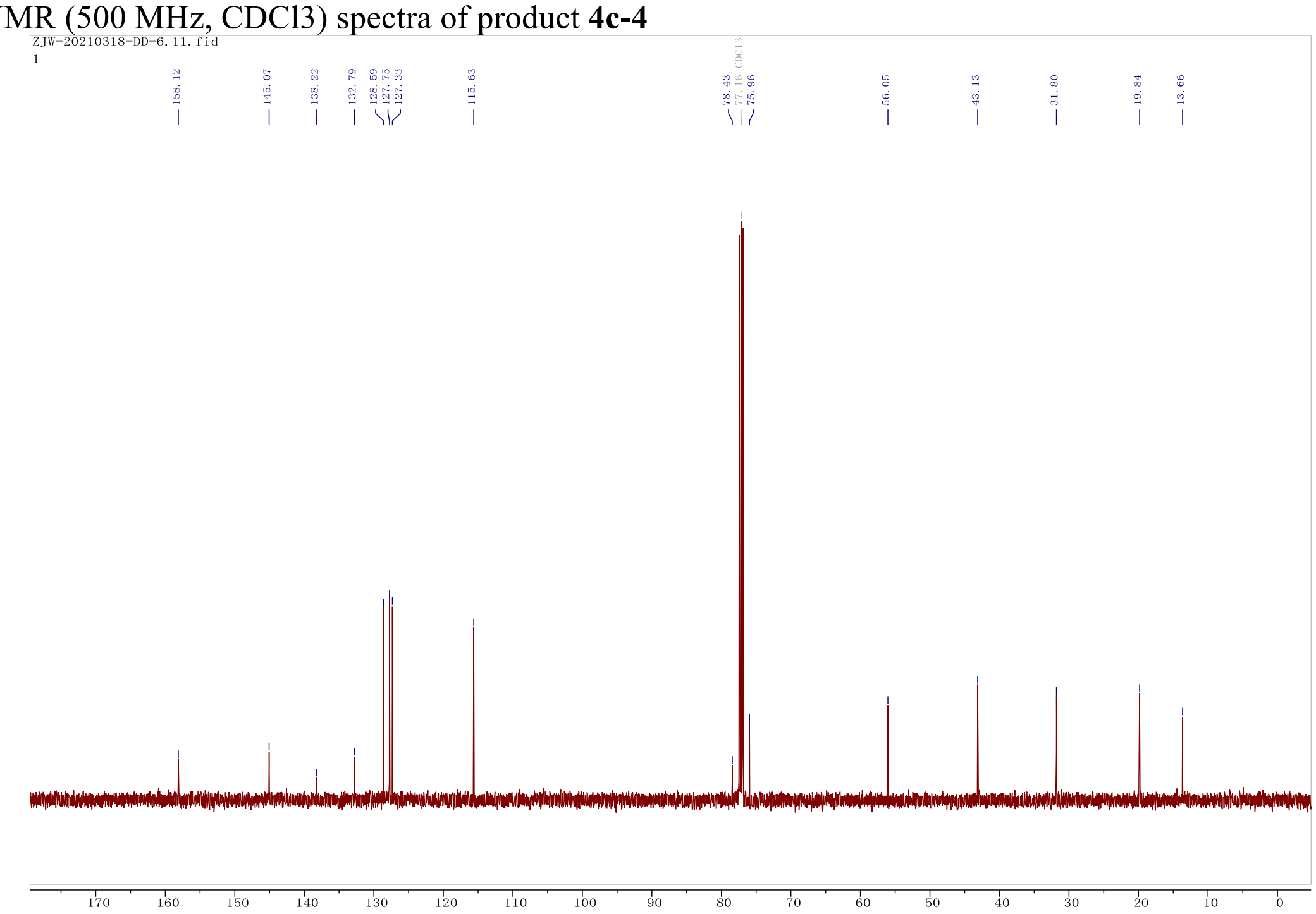

Fig. N46 ^{13}C NMR (126 MHz, CDCl3) spectra of product **4c-4**

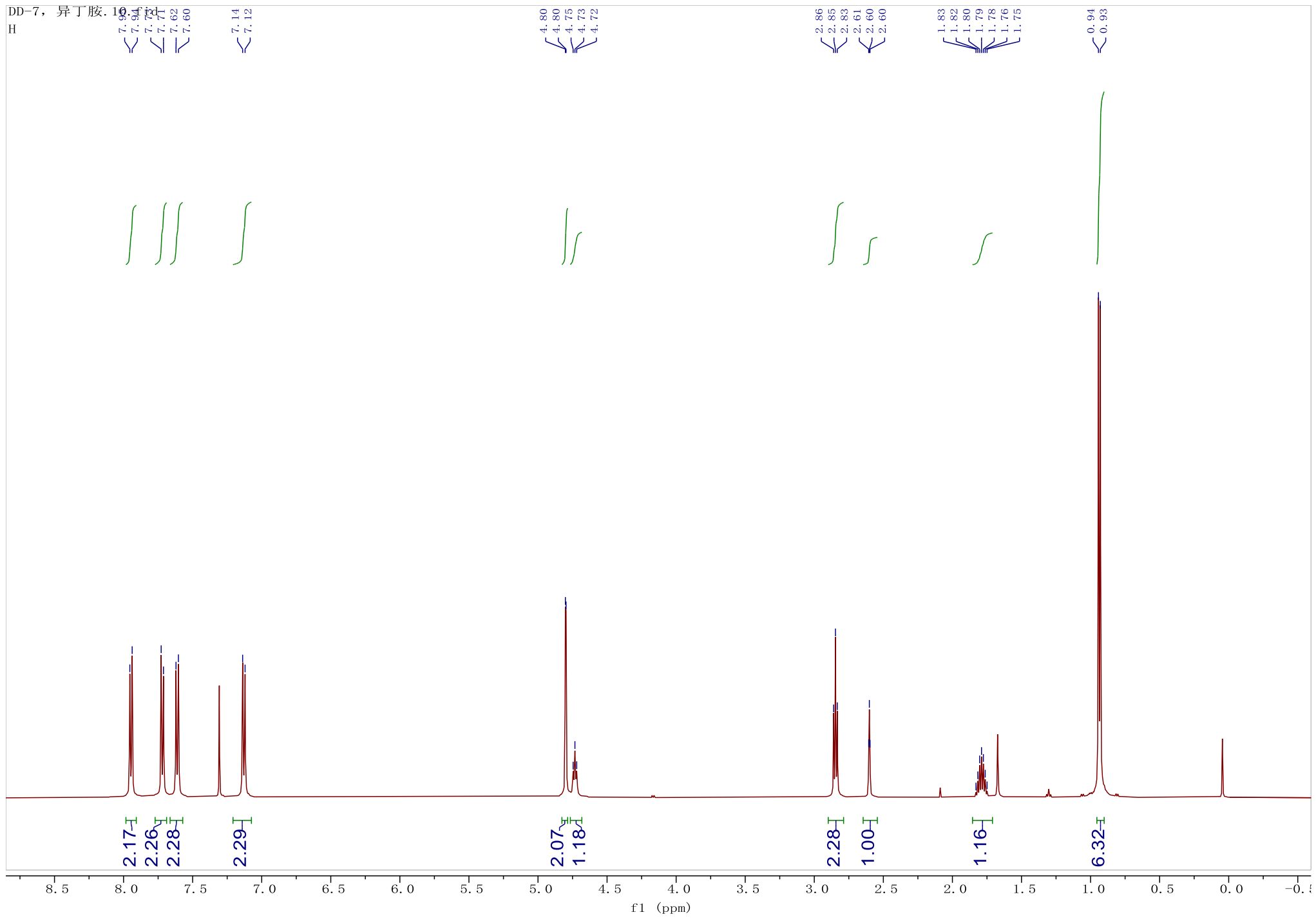

Fig. N47 ^{1}H NMR (500 MHz, CDCl3) spectra of product **4c-5**

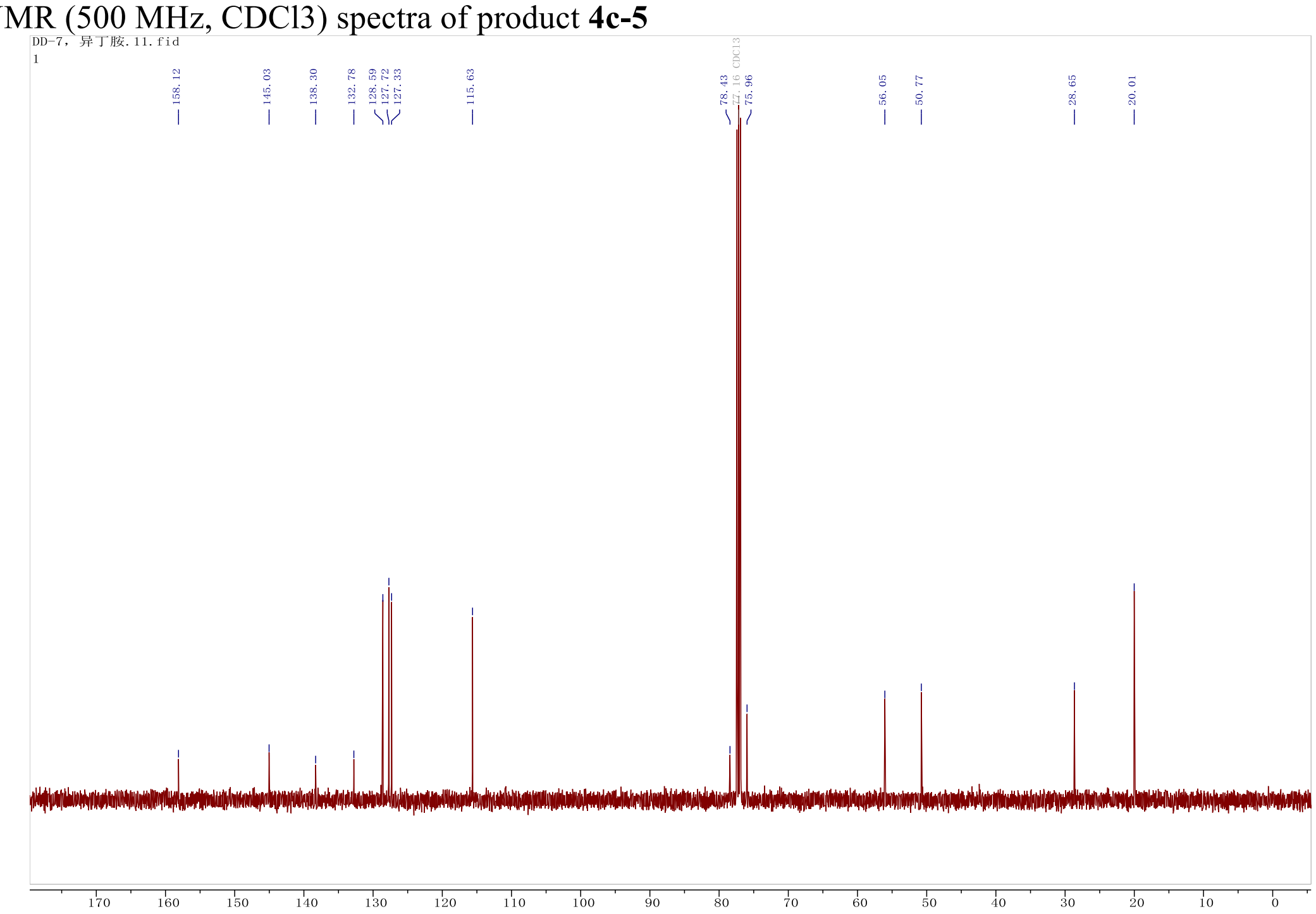

Fig. N48 ^{13}C NMR (126 MHz, CDCl3) spectra of product **4c-5**

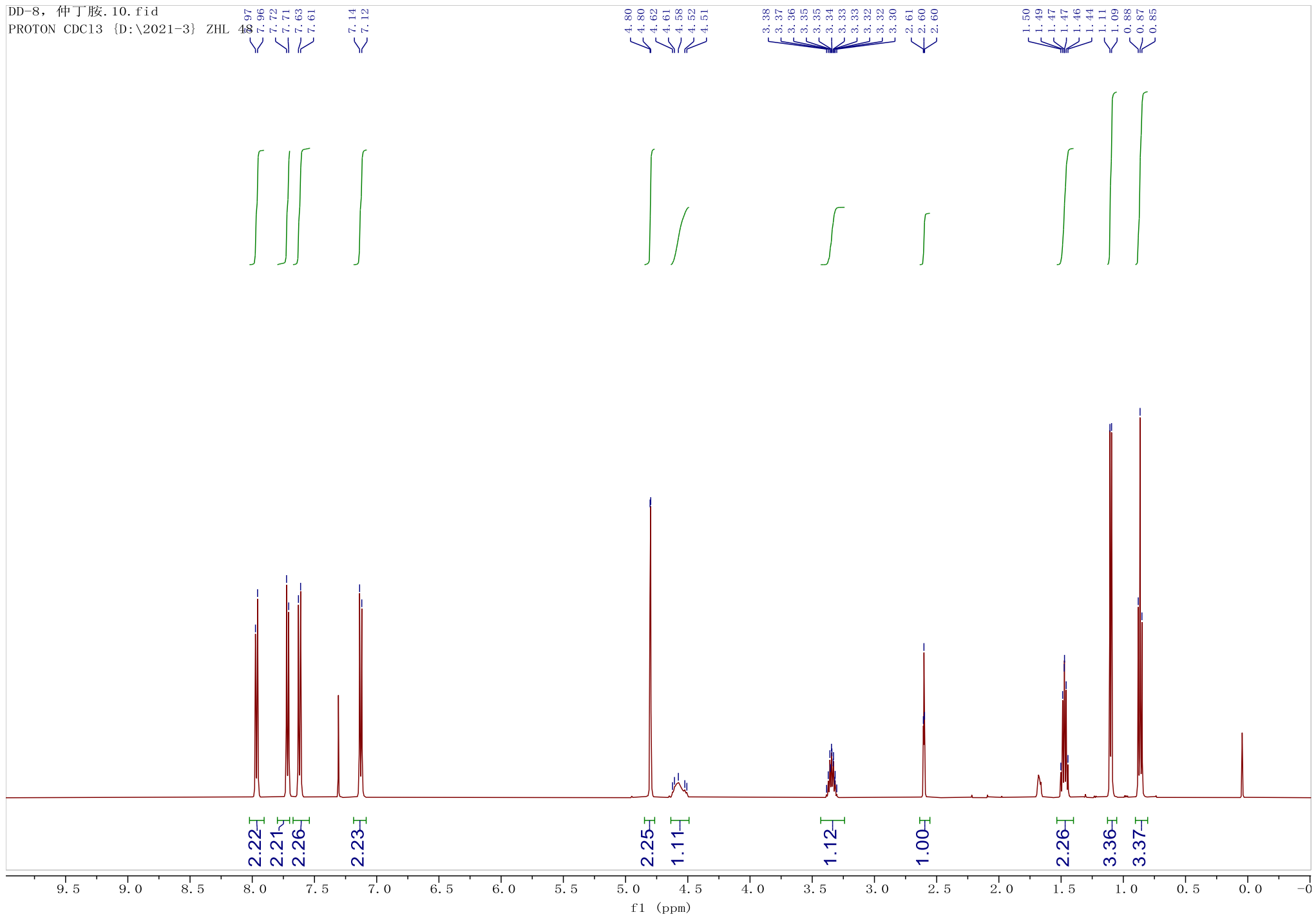

Fig. N49 ^{1}H NMR (500 MHz, CDCl3) spectra of product **4c-6**

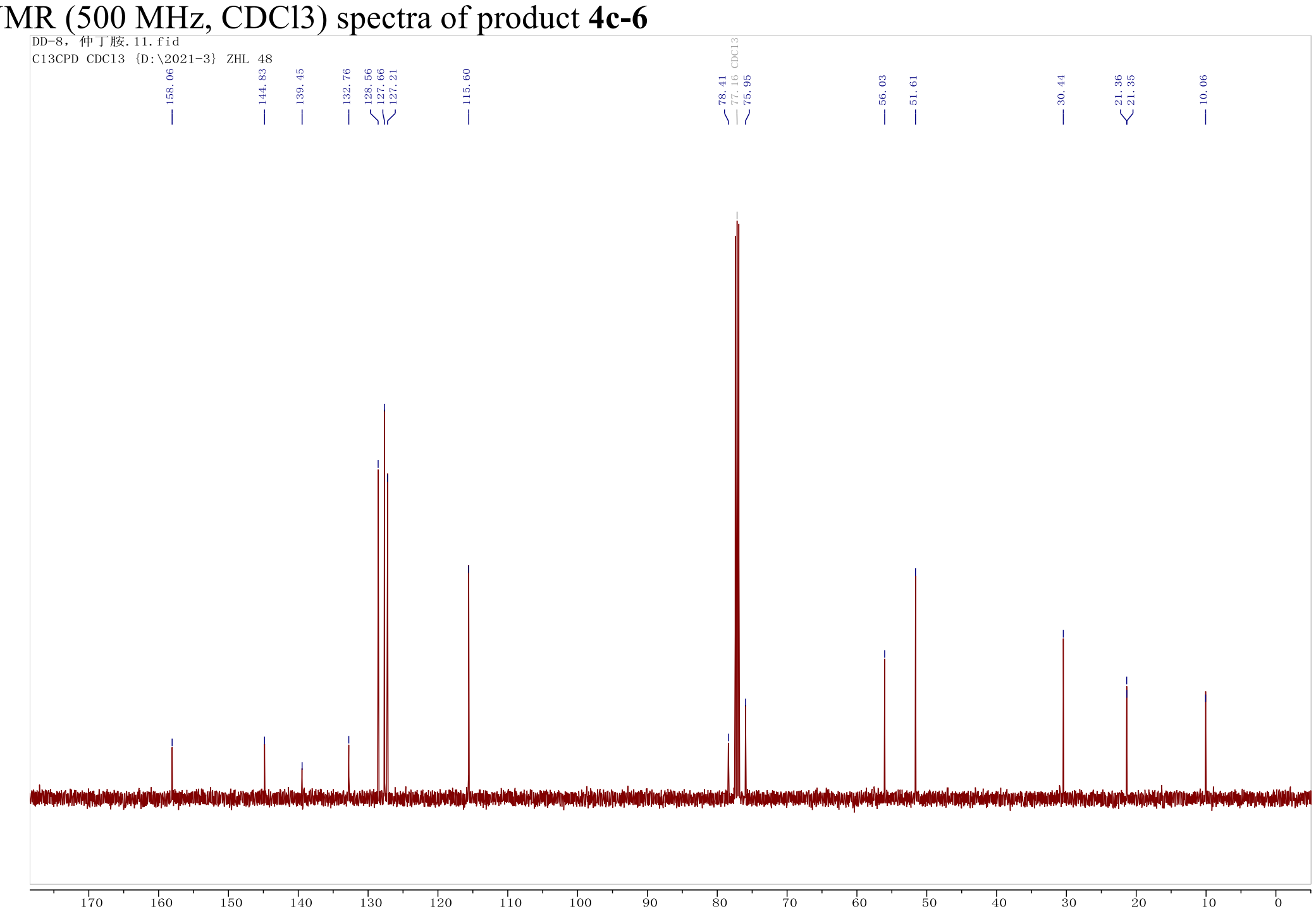

Fig. N50 ^{13}C NMR (126 MHz, CDCl3) spectra of product **4c-6**

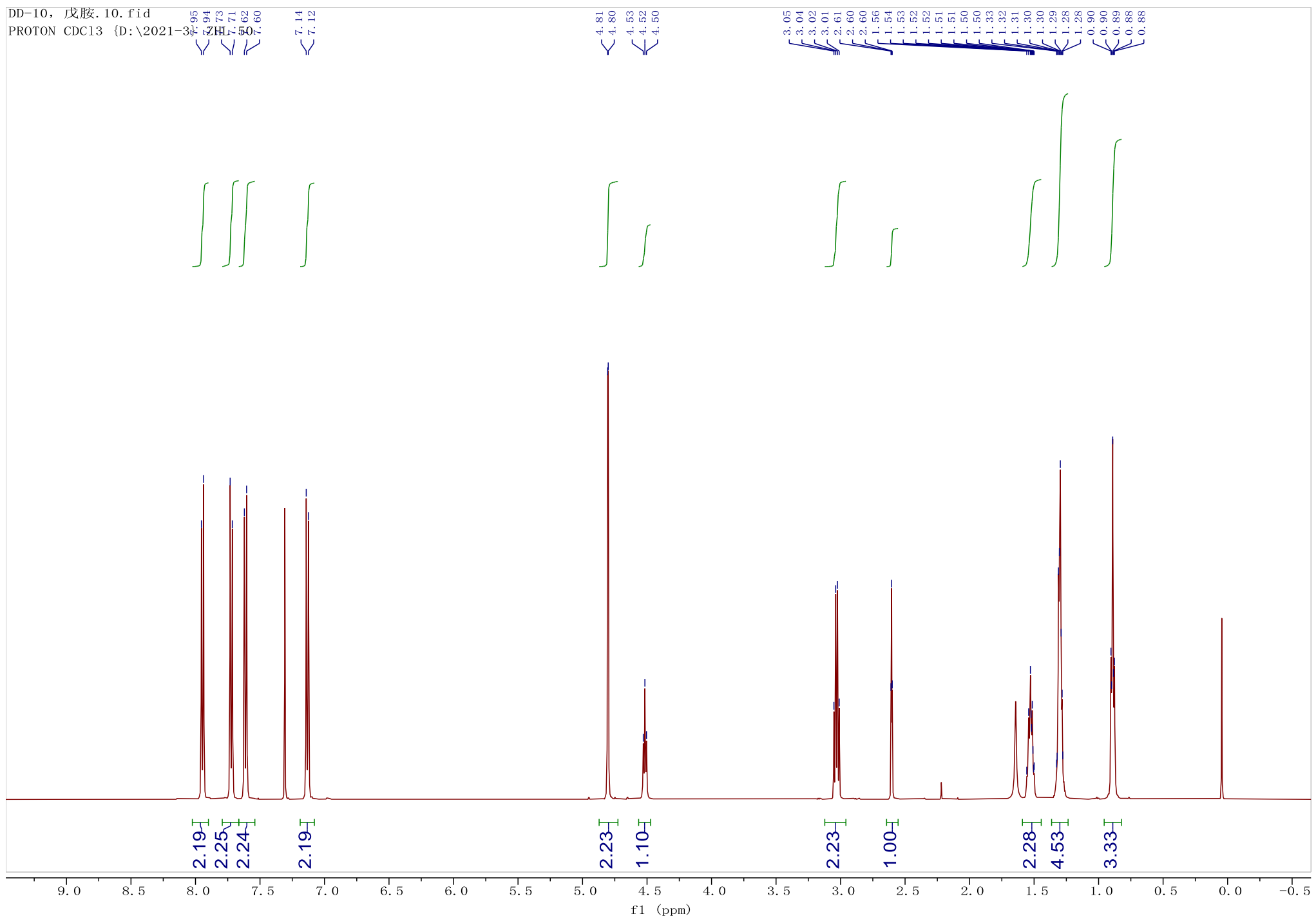

Fig. N51 ^{1}H NMR (500 MHz, CDCl3) spectra of product **4c-7**

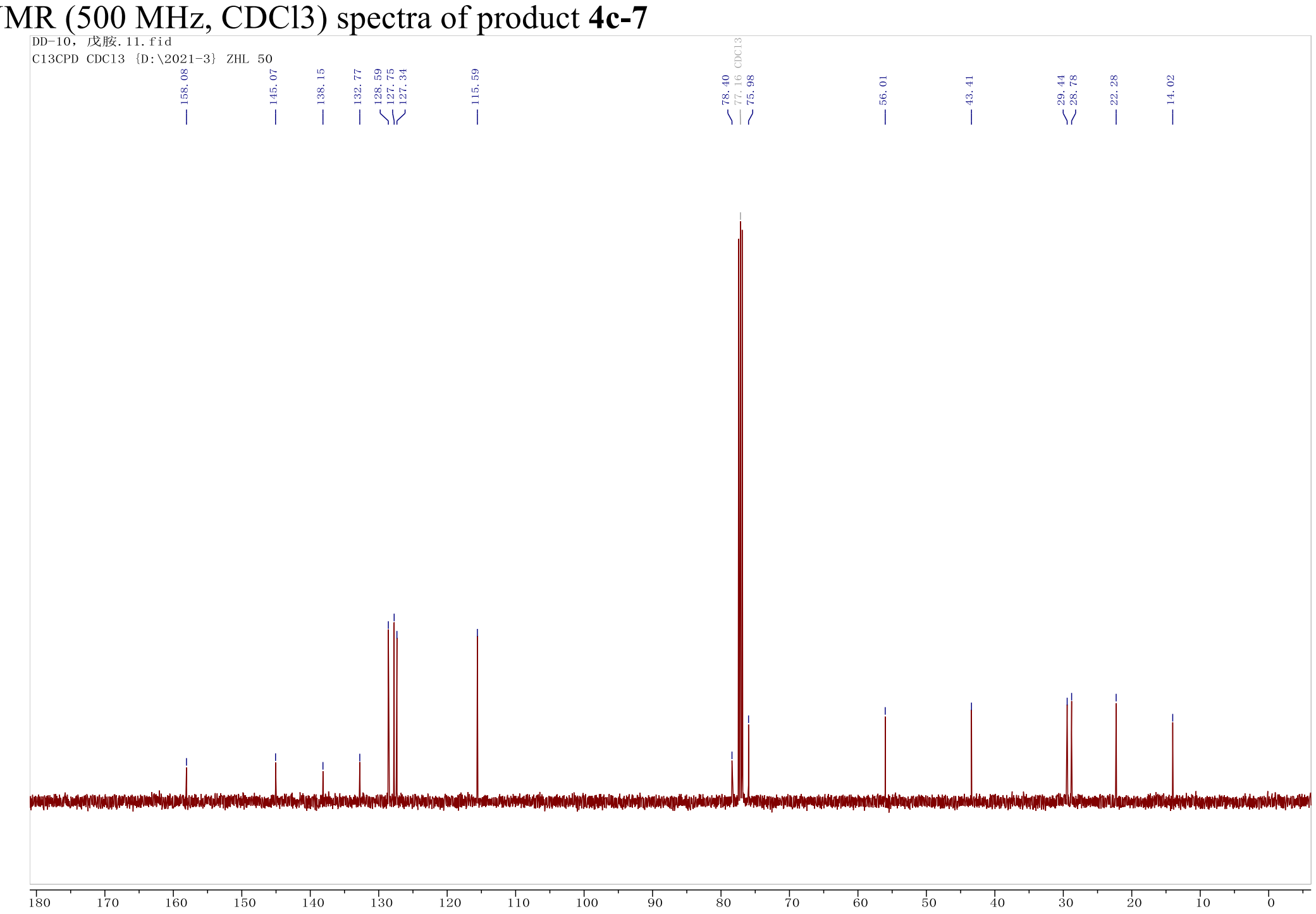

Fig. N52 ^{13}C NMR (126 MHz, CDCl3) spectra of product **4c-7**

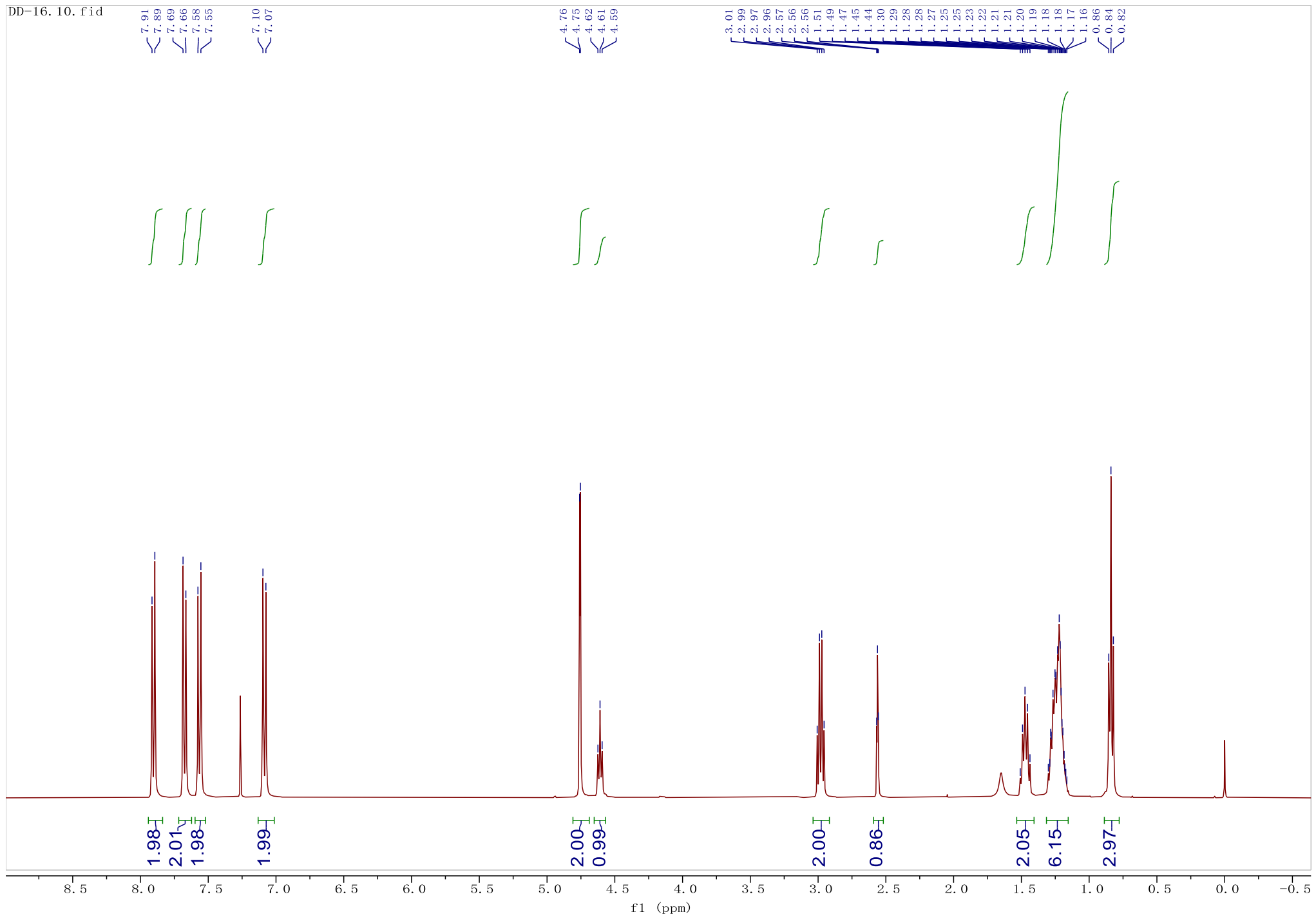

Fig. N53 ^{1}H NMR (400 MHz, CDCl3) spectra of product **4c-8**

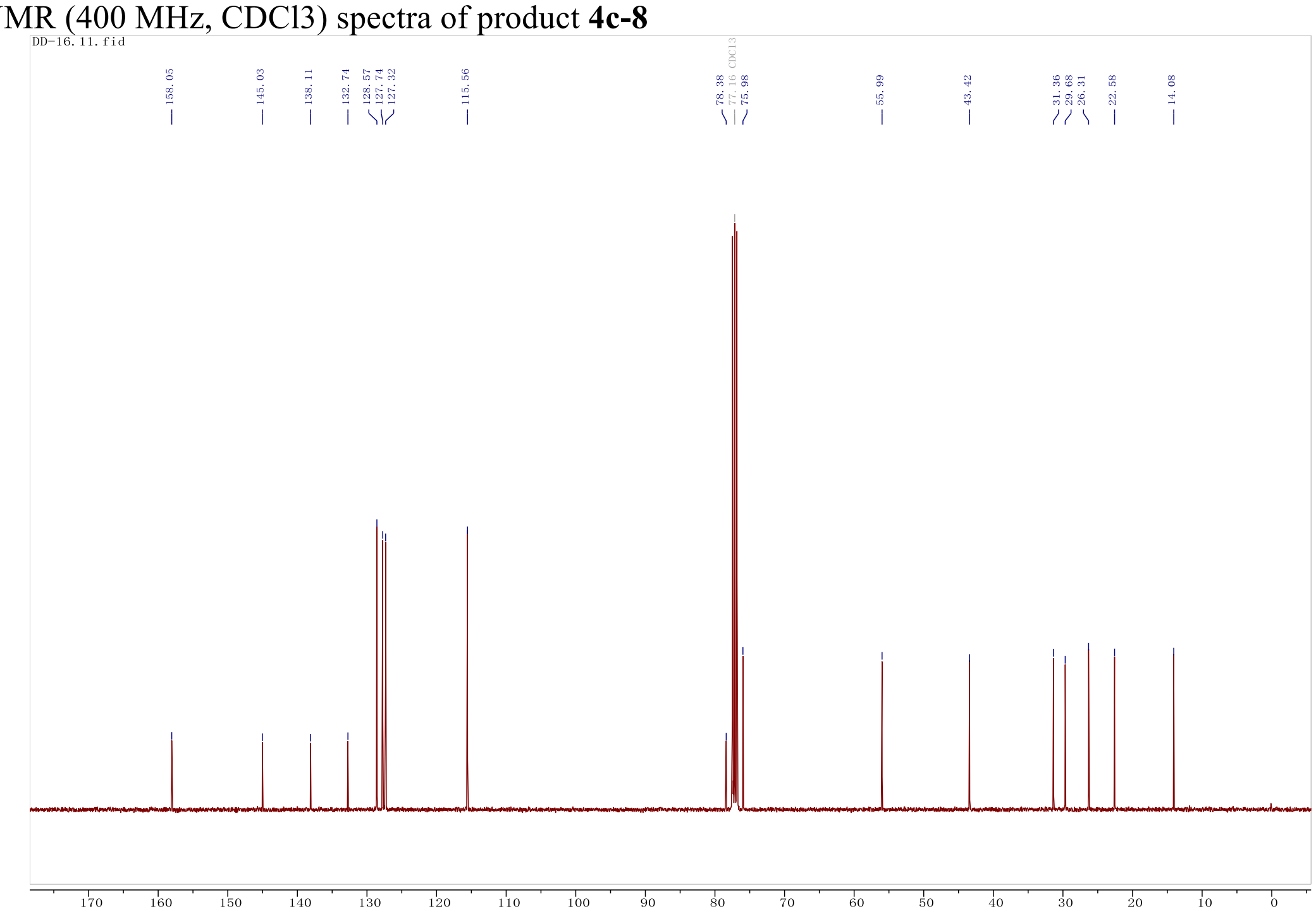

Fig. N54 ^{13}C NMR (101 MHz, CDCl3) spectra of product **4c-8**

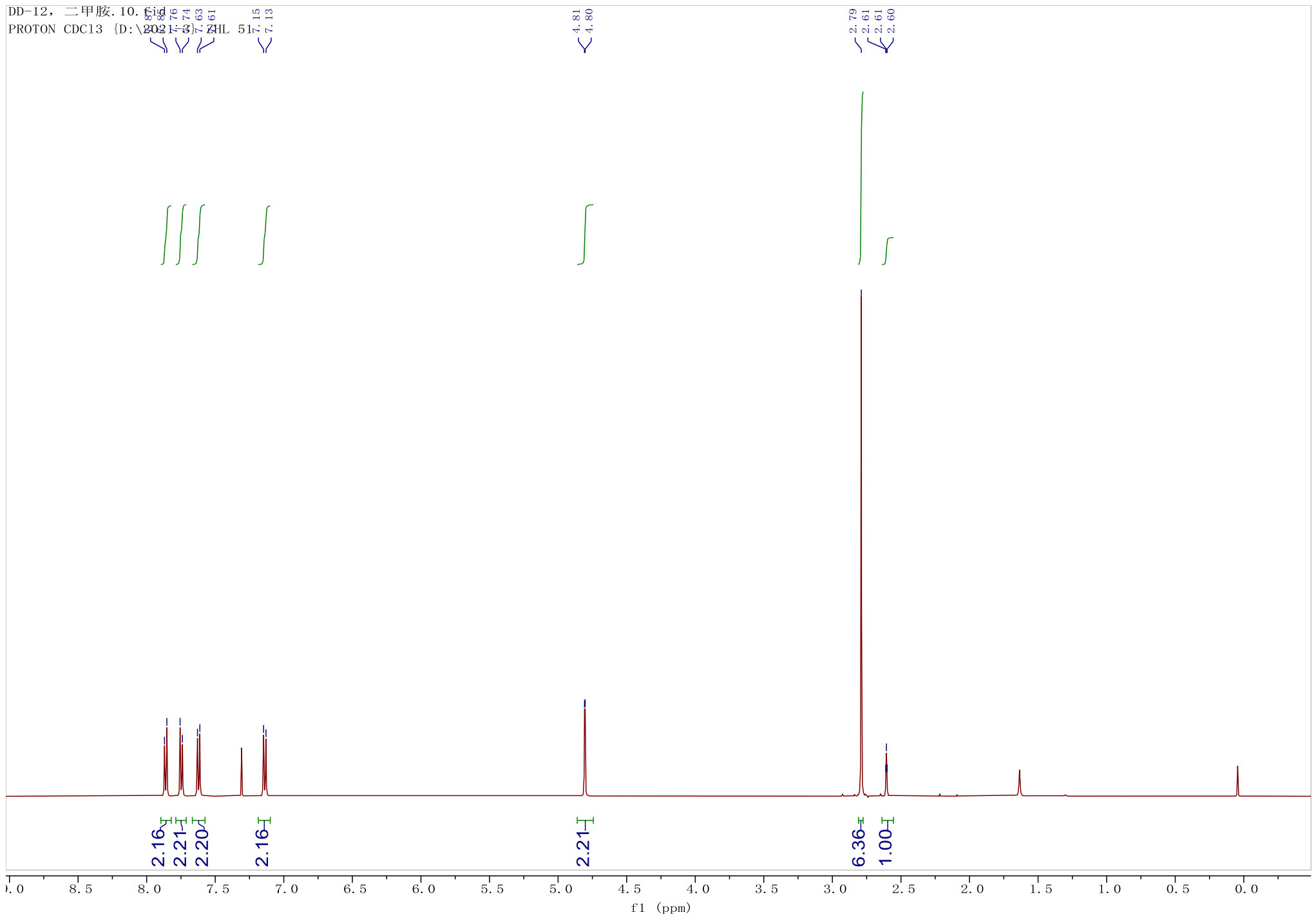

Fig. N55 ^{1}H NMR (500 MHz, CDCl3) spectra of product **4c-9**

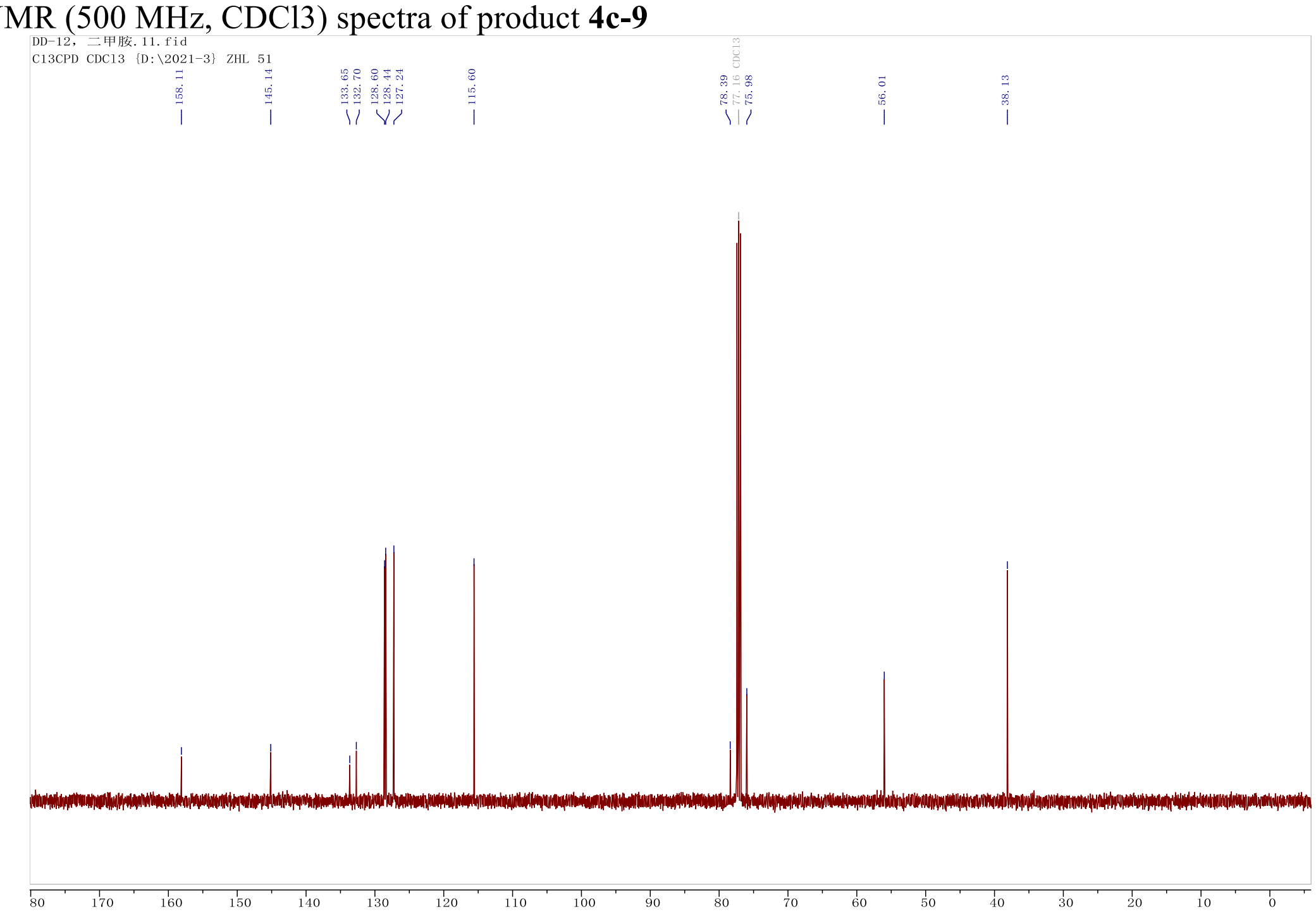

Fig. N56 ^{13}C NMR (126 MHz, CDCl3) spectra of product **4c-9**

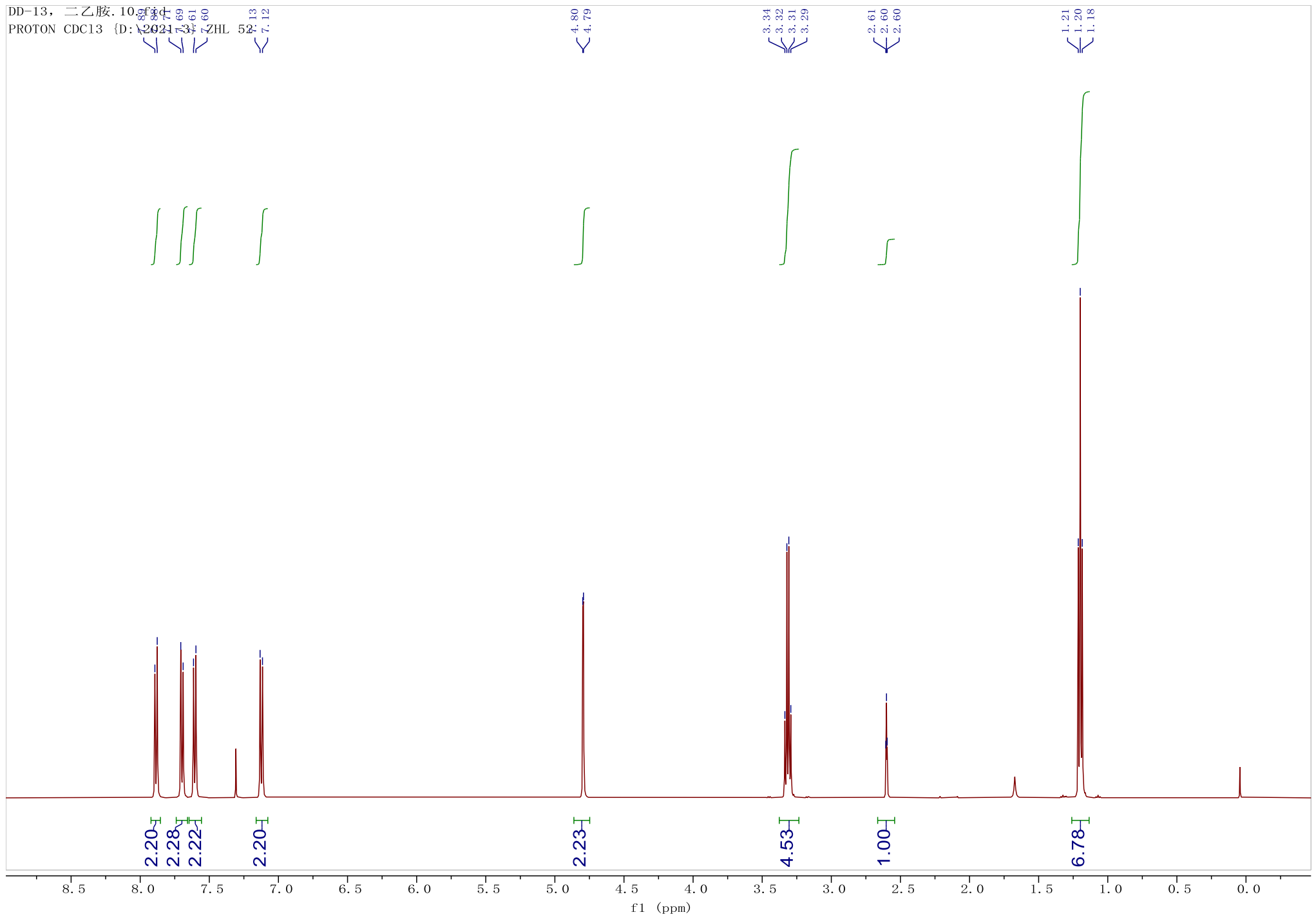

Fig. N57 ^{1}H NMR (500 MHz, CDCl3) spectra of product **4c-10**

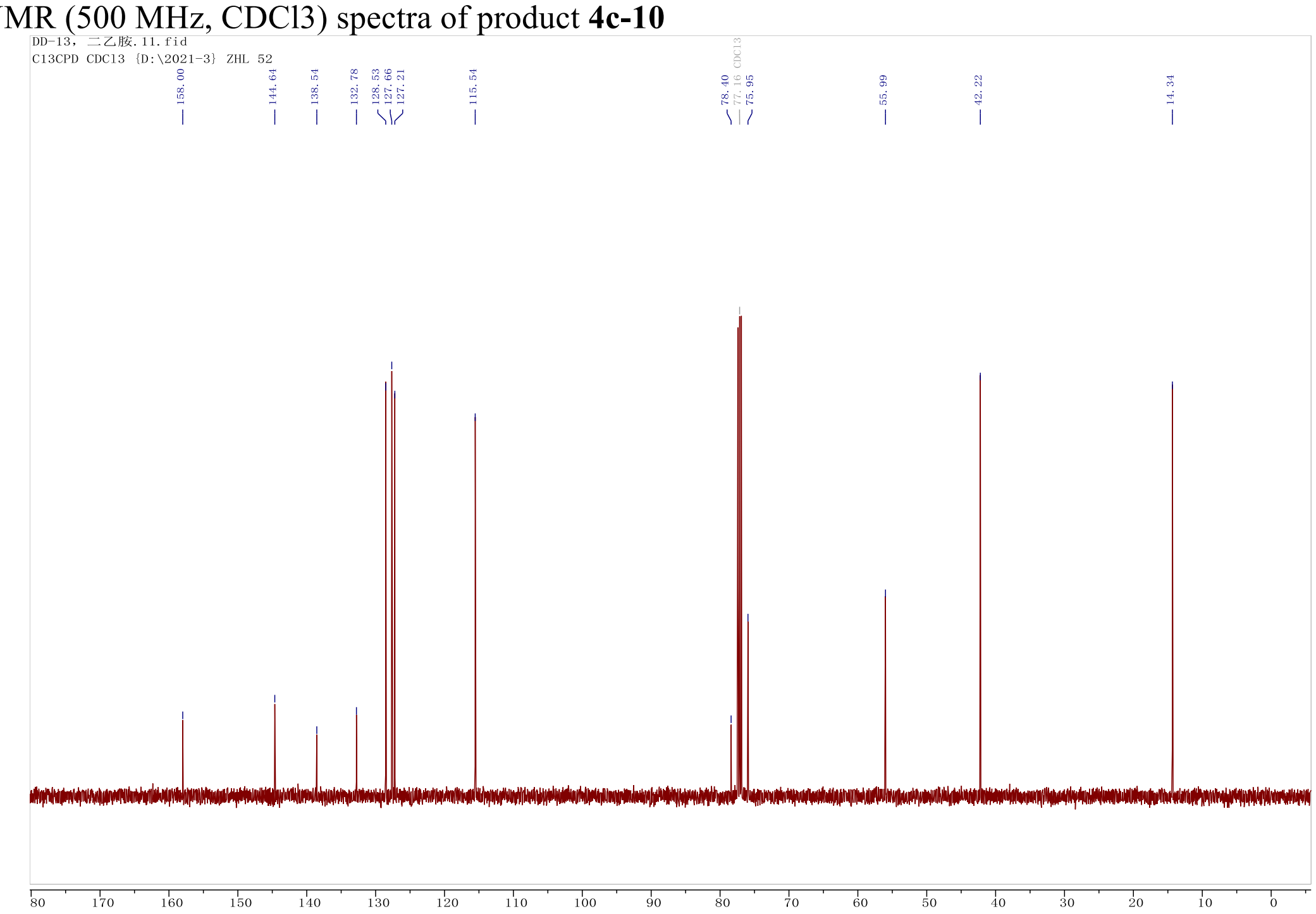

Fig. N58 ^{13}C NMR (126 MHz, CDCl3) spectra of product **4c-10**

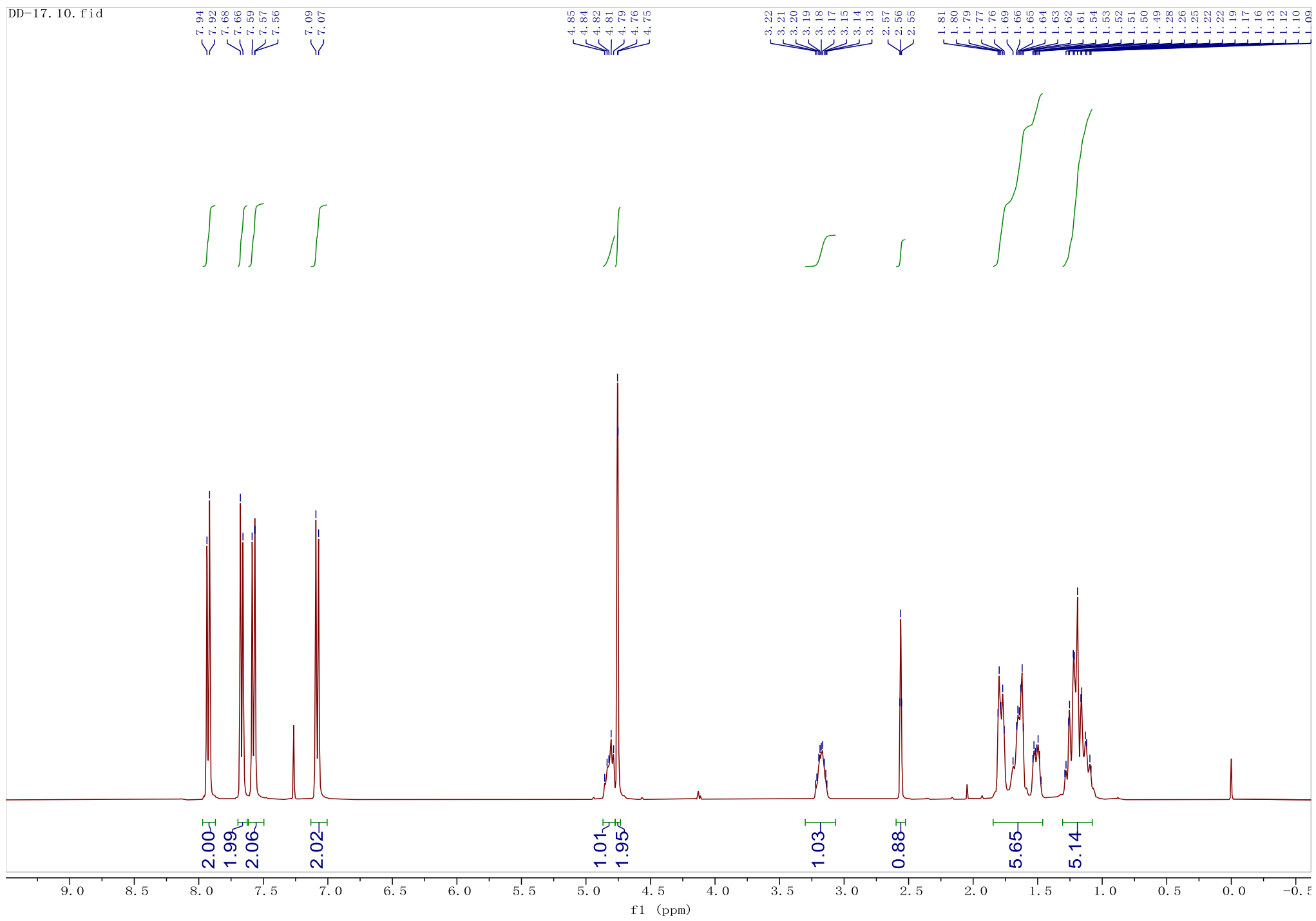

Fig. N59 ^{1}H NMR (400 MHz, CDCl3) spectra of product **4c-11**

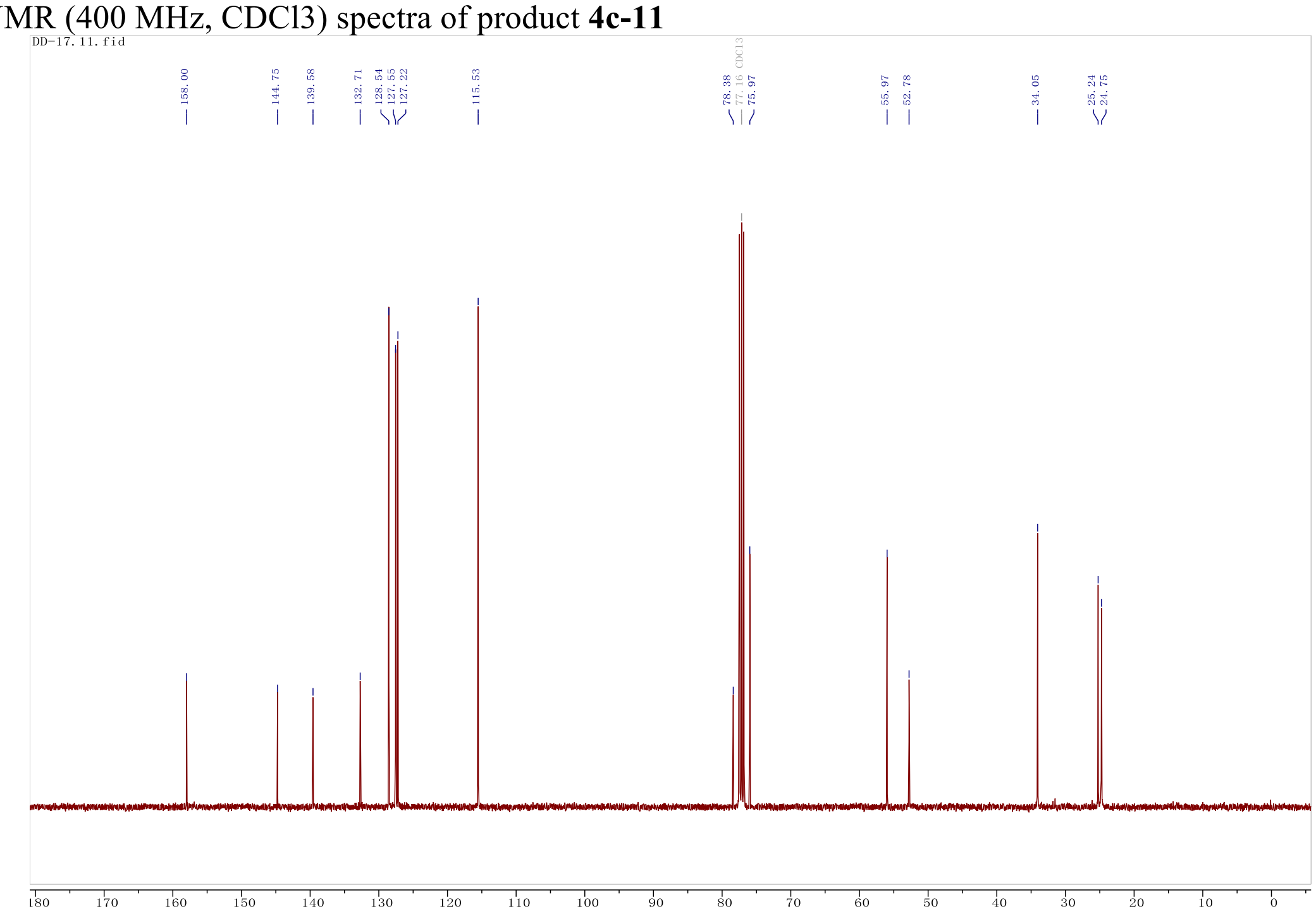

Fig. N60 ^{13}C NMR (101 MHz, CDCl3) spectra of product **4c-11**

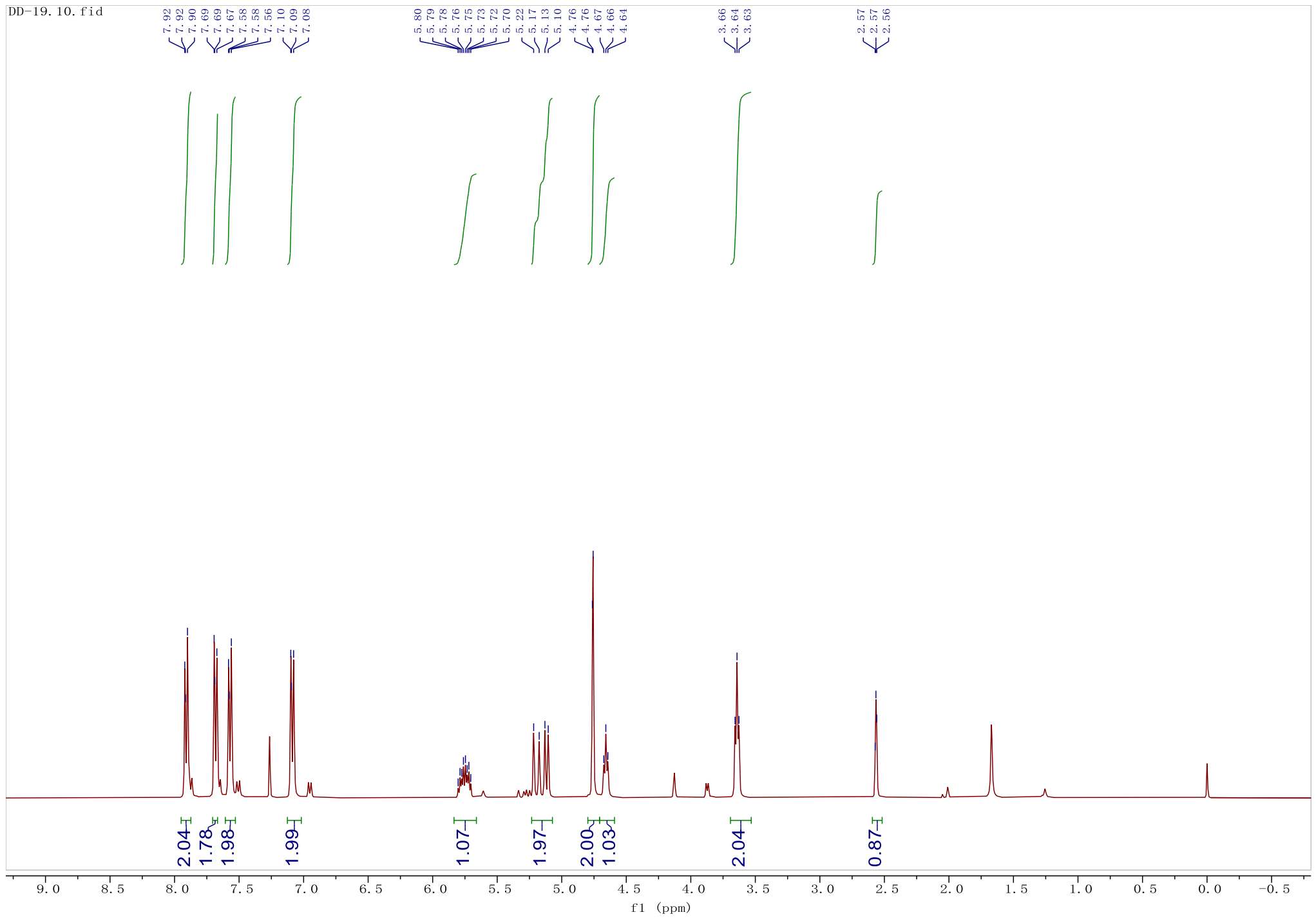

Fig. N61 ^{1}H NMR (400 MHz, CDCl3) spectra of product **4c-12**

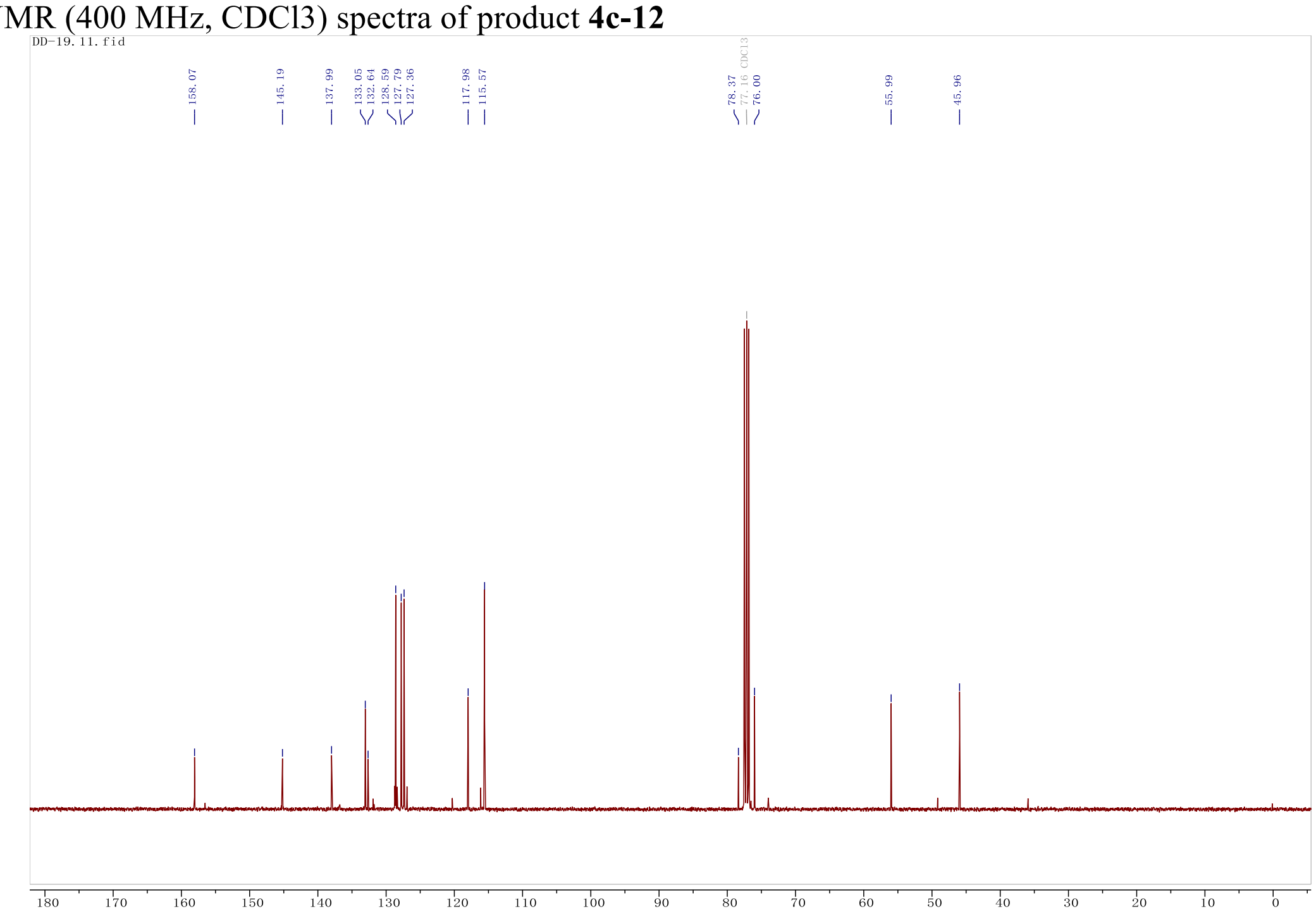

Fig. N62 ^{13}C NMR (101 MHz, CDCl3) spectra of product **4c-12**

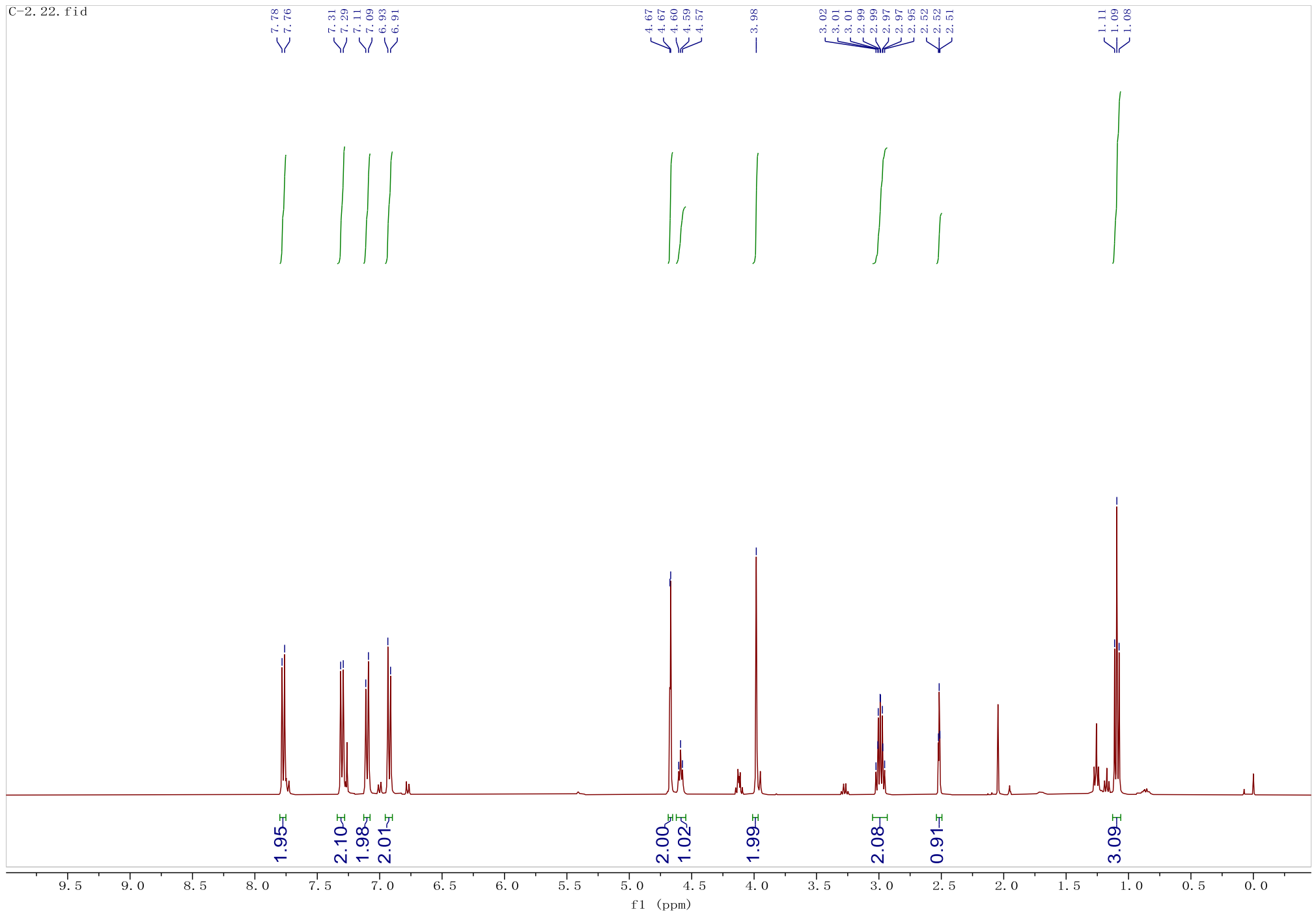

Fig. N63 ^{1}H NMR (400 MHz, CDCl3) spectra of product **6-1**

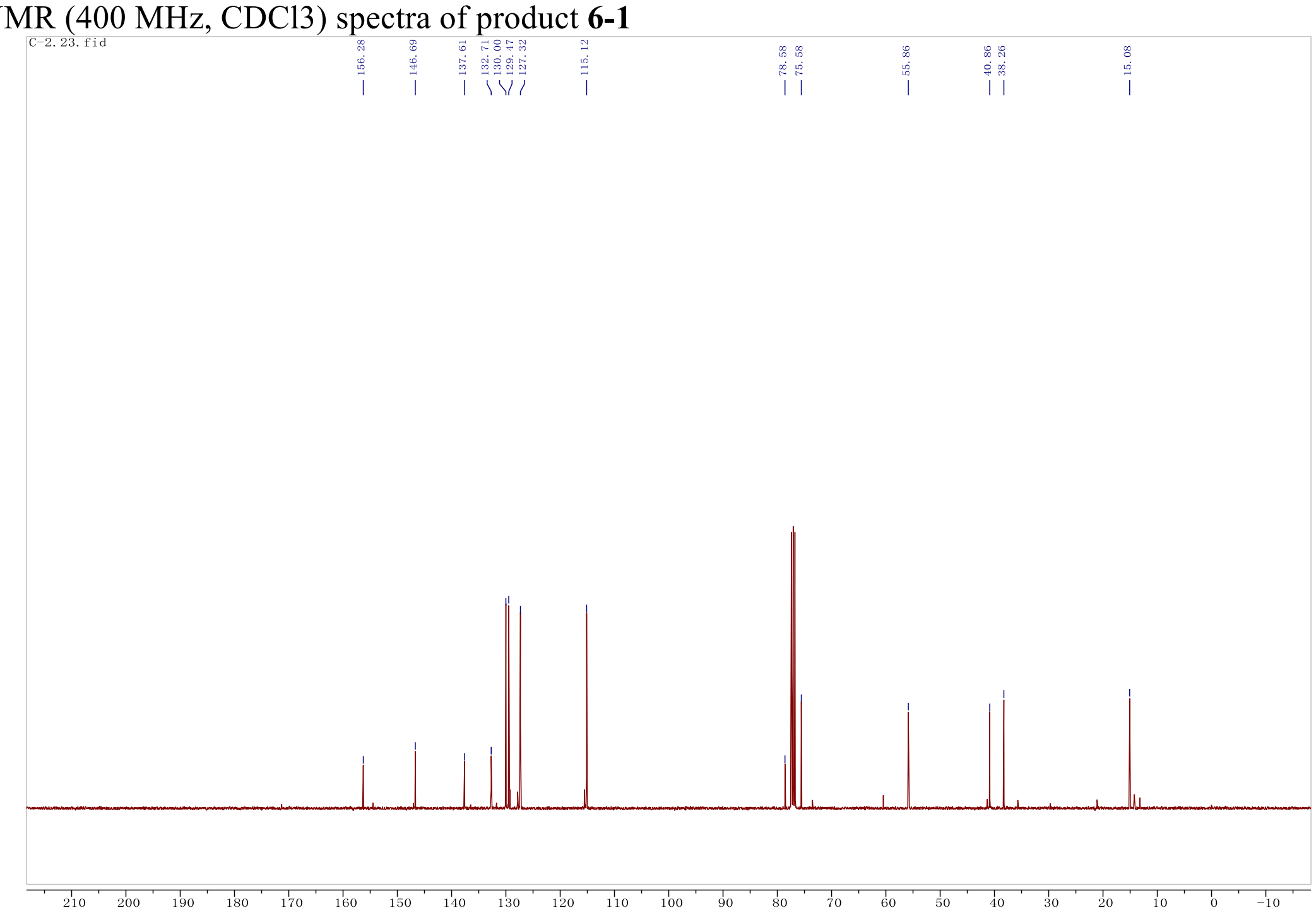

Fig. N64 ^{13}C NMR (101 MHz, CDCl3) spectra of product **6-1**

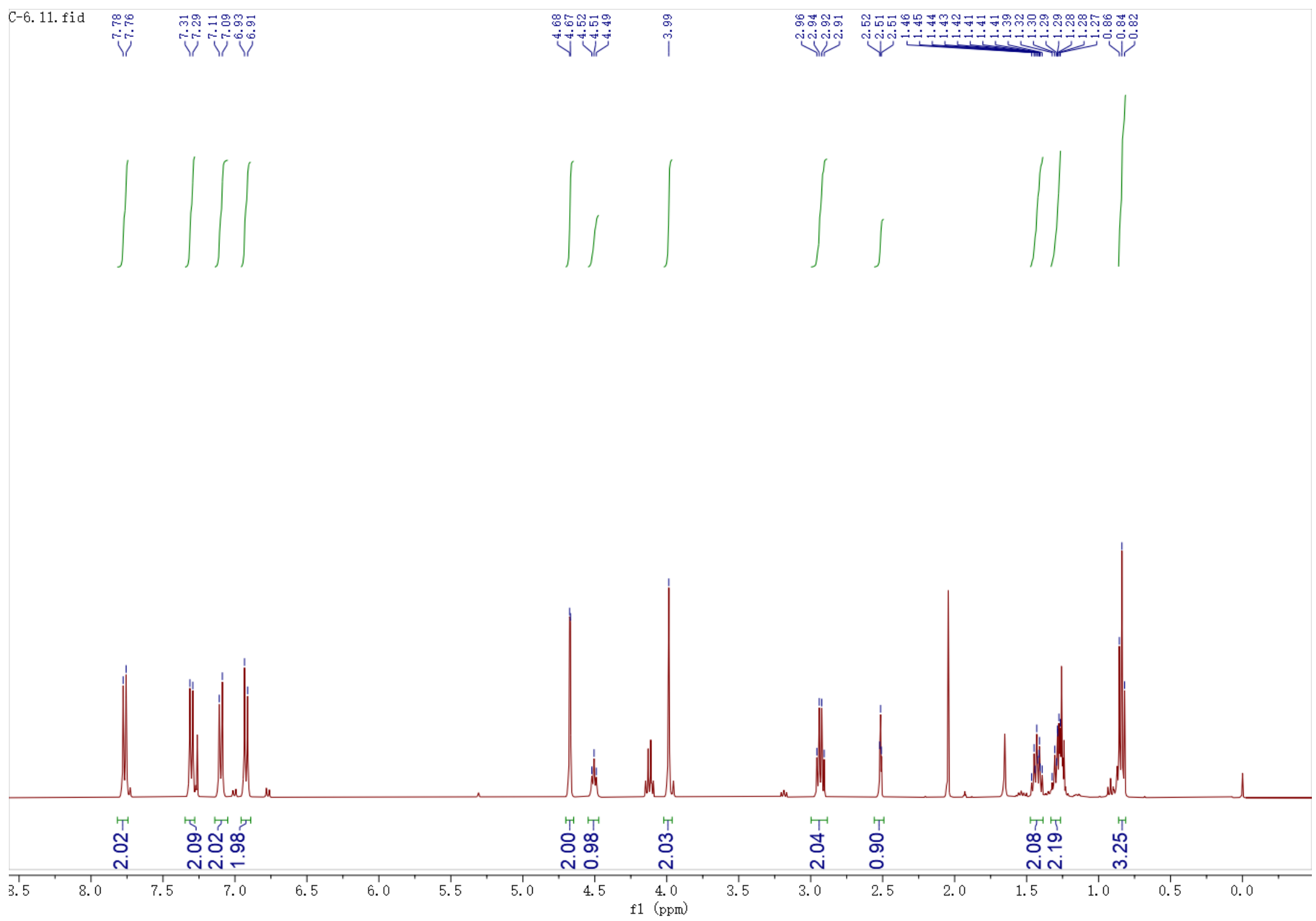

Fig. N65 ^{1}H NMR (400 MHz, CDCl3) spectra of product **6-2**

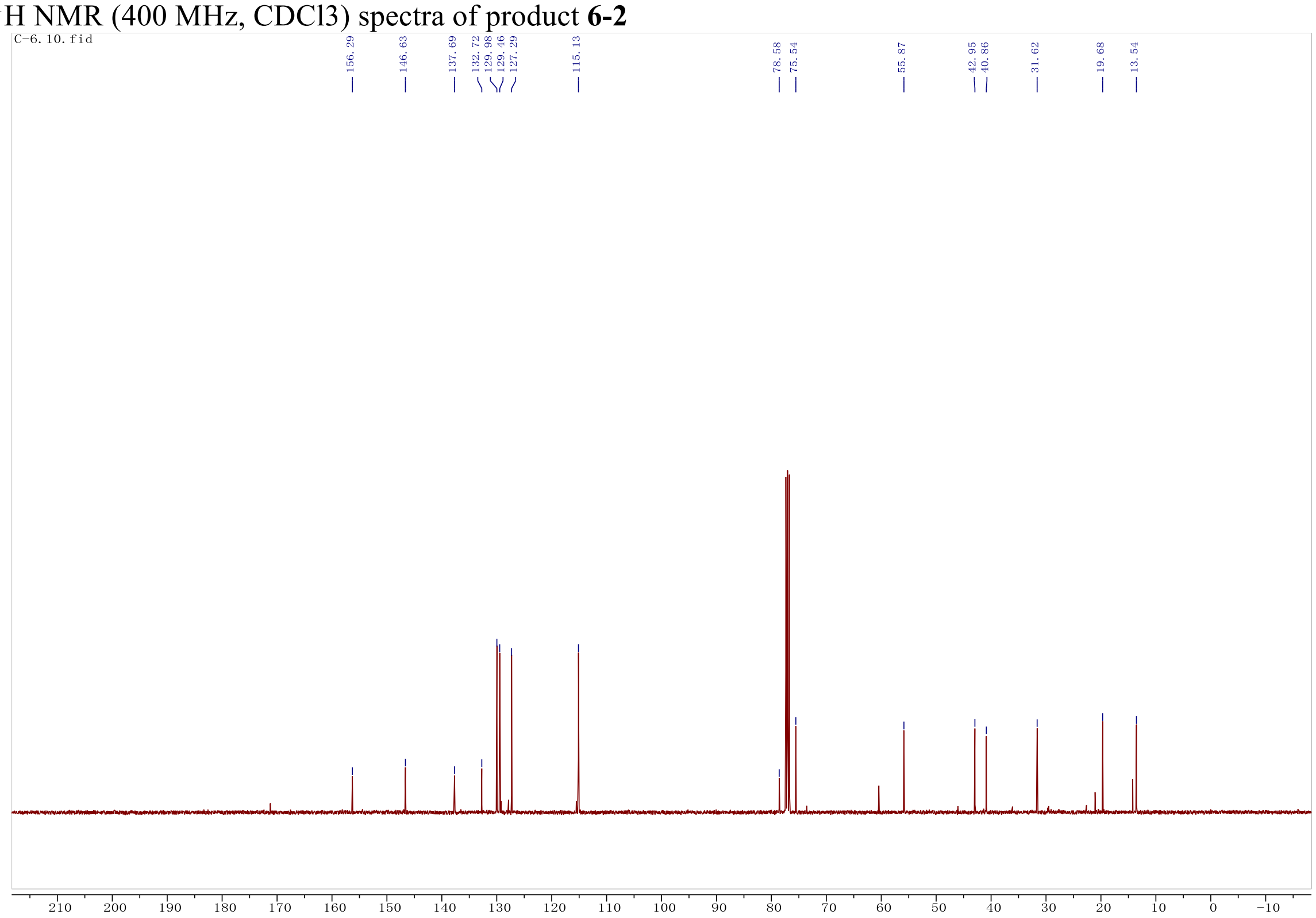

Fig. 66 ^{13}C NMR (101 MHz, CDCl3) spectra of product **6-2**

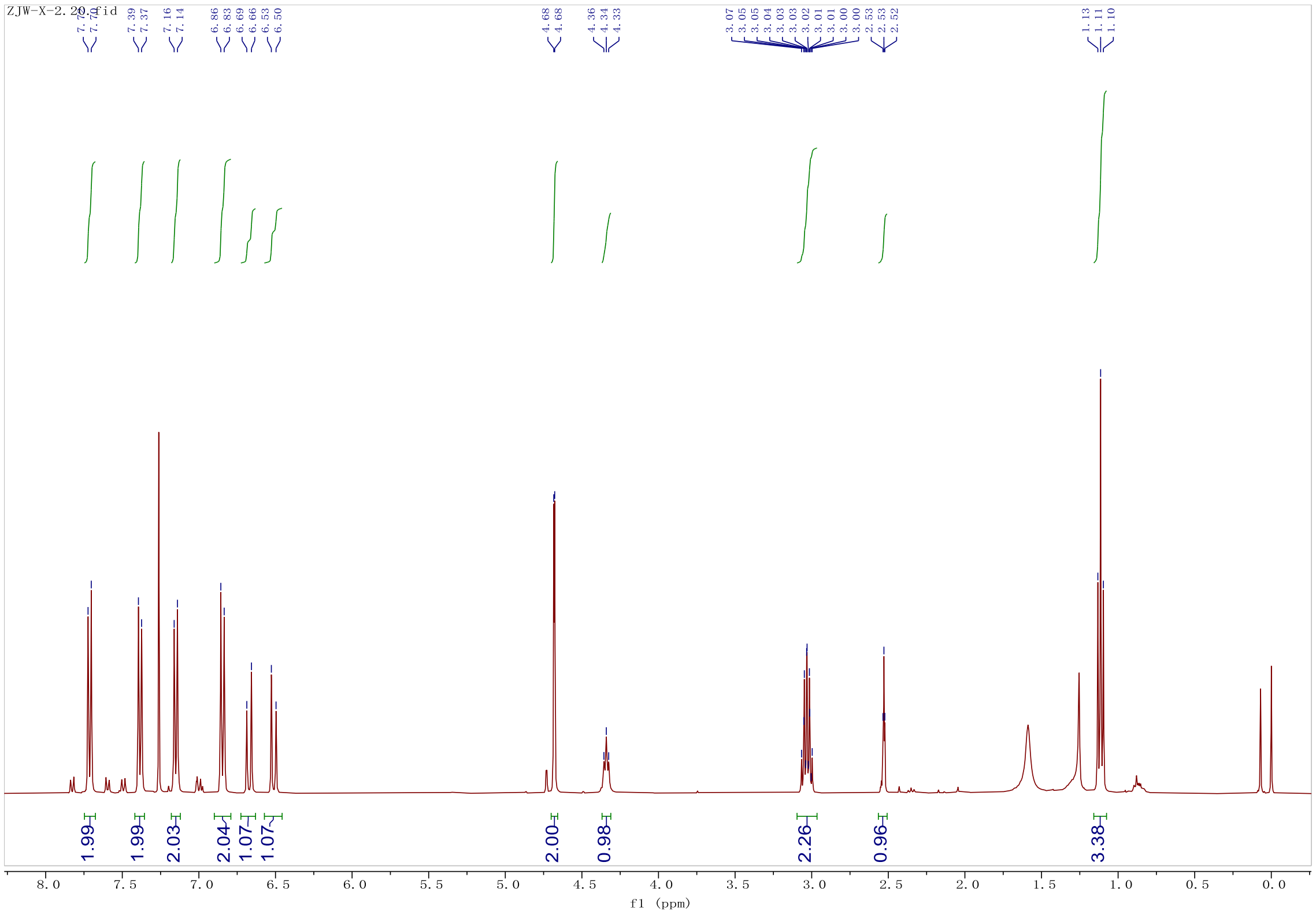

Fig. N67 ^{1}H NMR (400 MHz, CDCl3) spectra of product **8-1**

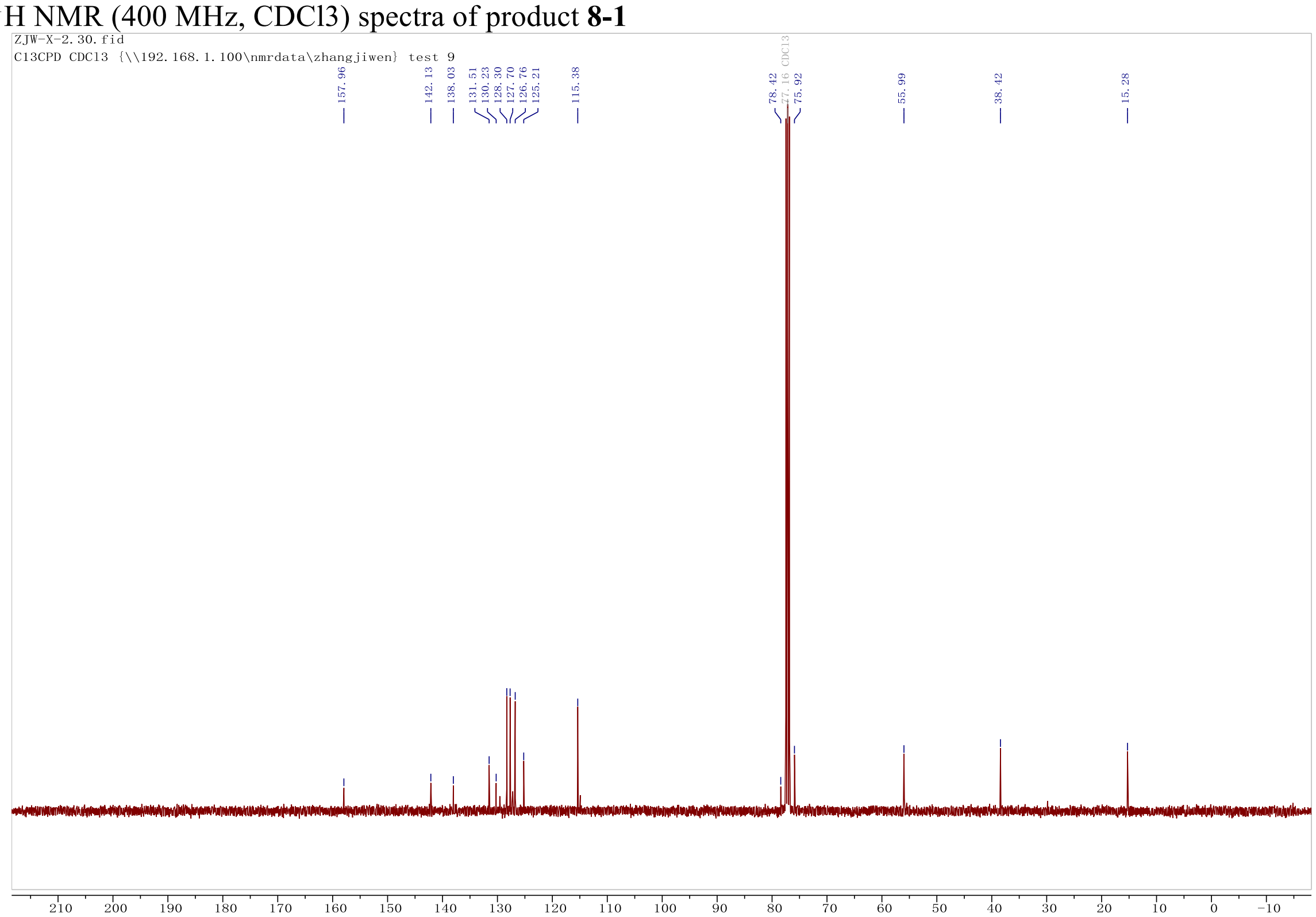

Fig. N68 ^{13}C NMR (101 MHz, CDCl3) spectra of product **8-1**

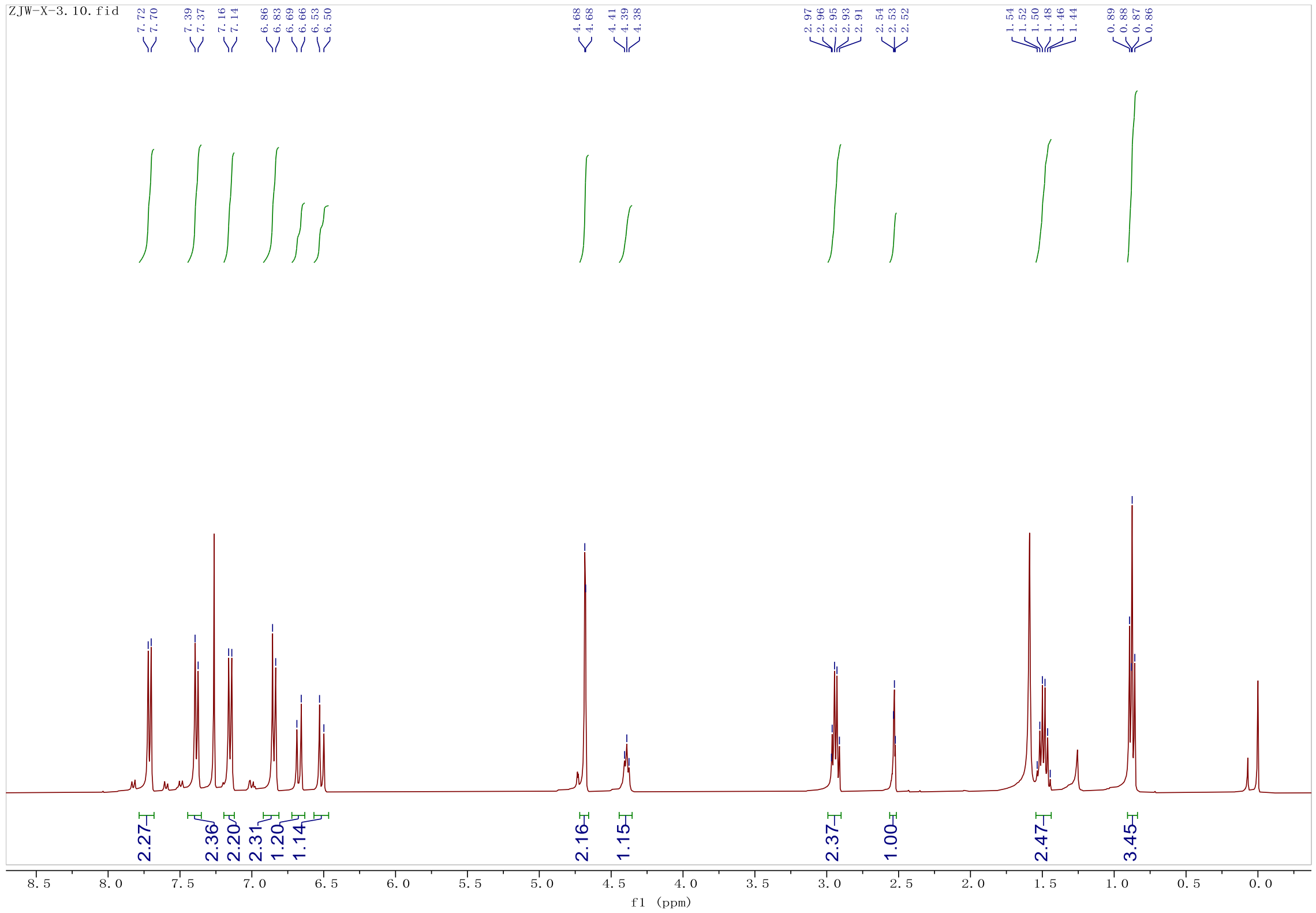

Fig. 69 ^{1}H NMR (400 MHz, CDCl3) spectra of product **8-2**

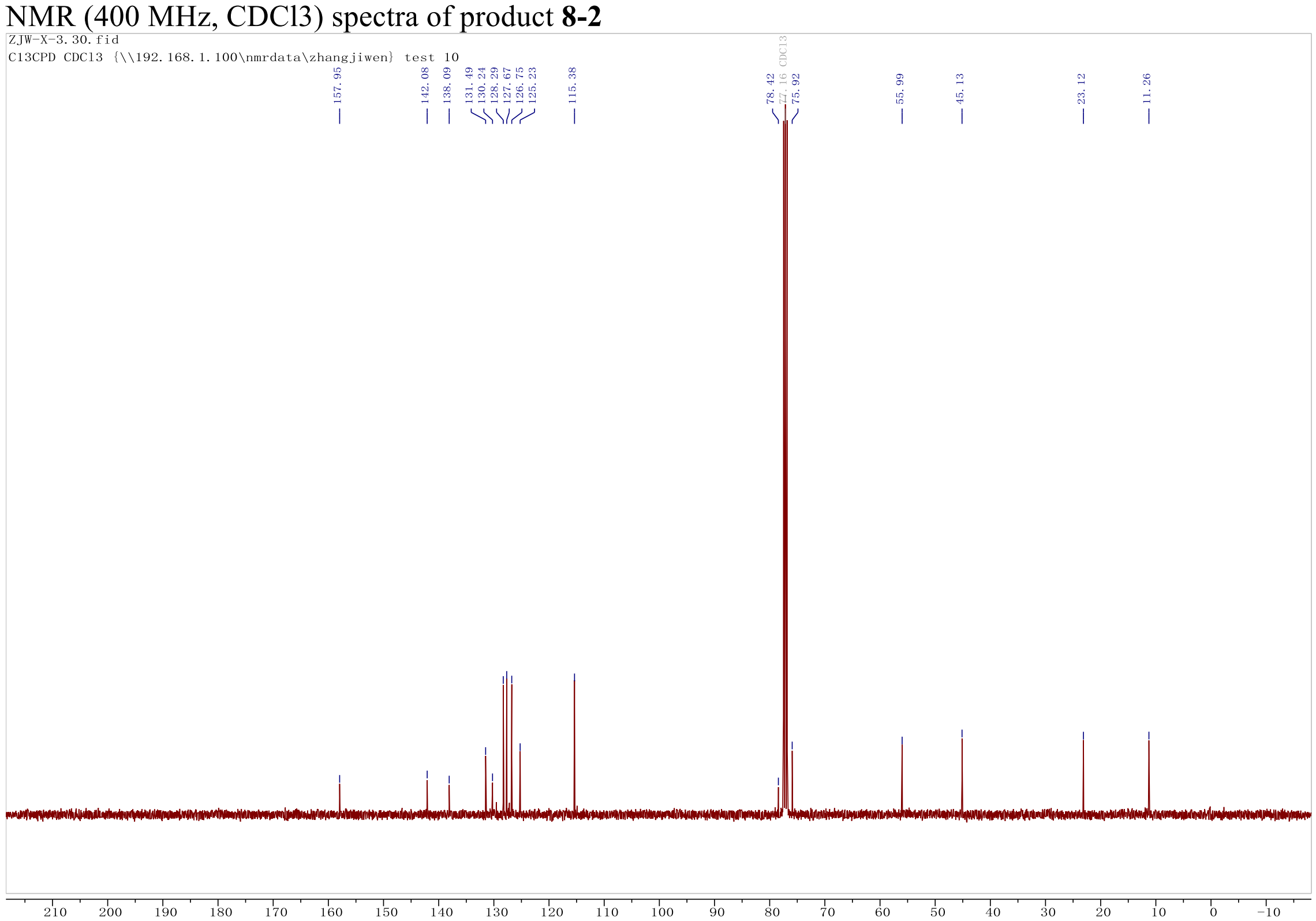

Fig. N70 ^{13}C NMR (101 MHz, CDCl3) spectra of product **8-2**

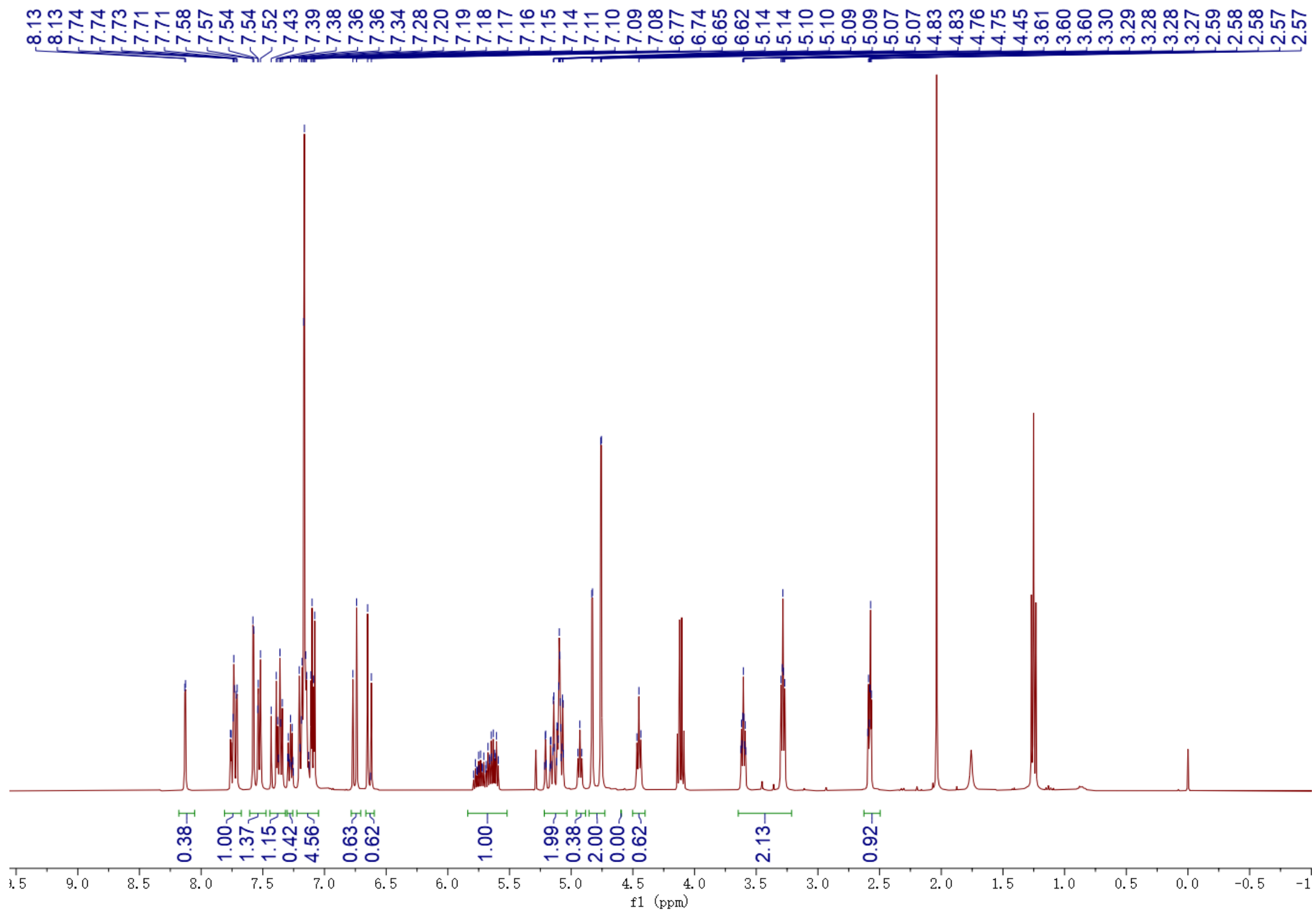

Fig. N71 ^{1}H NMR (400 MHz, CDCl3) spectra of product **8-3**

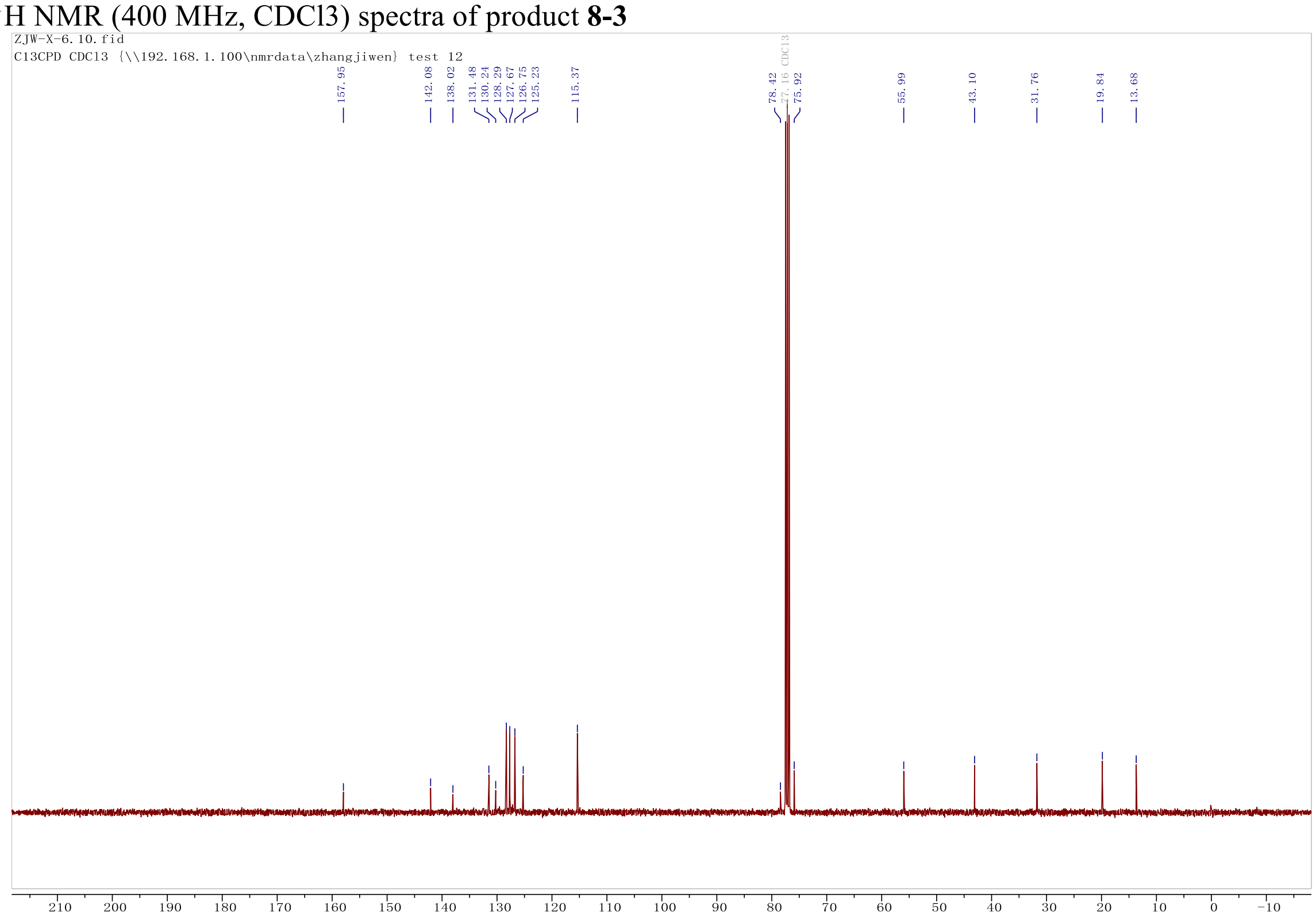

Fig. N72 ^{13}C NMR (101 MHz, CDCl3) spectra of product **8-3**

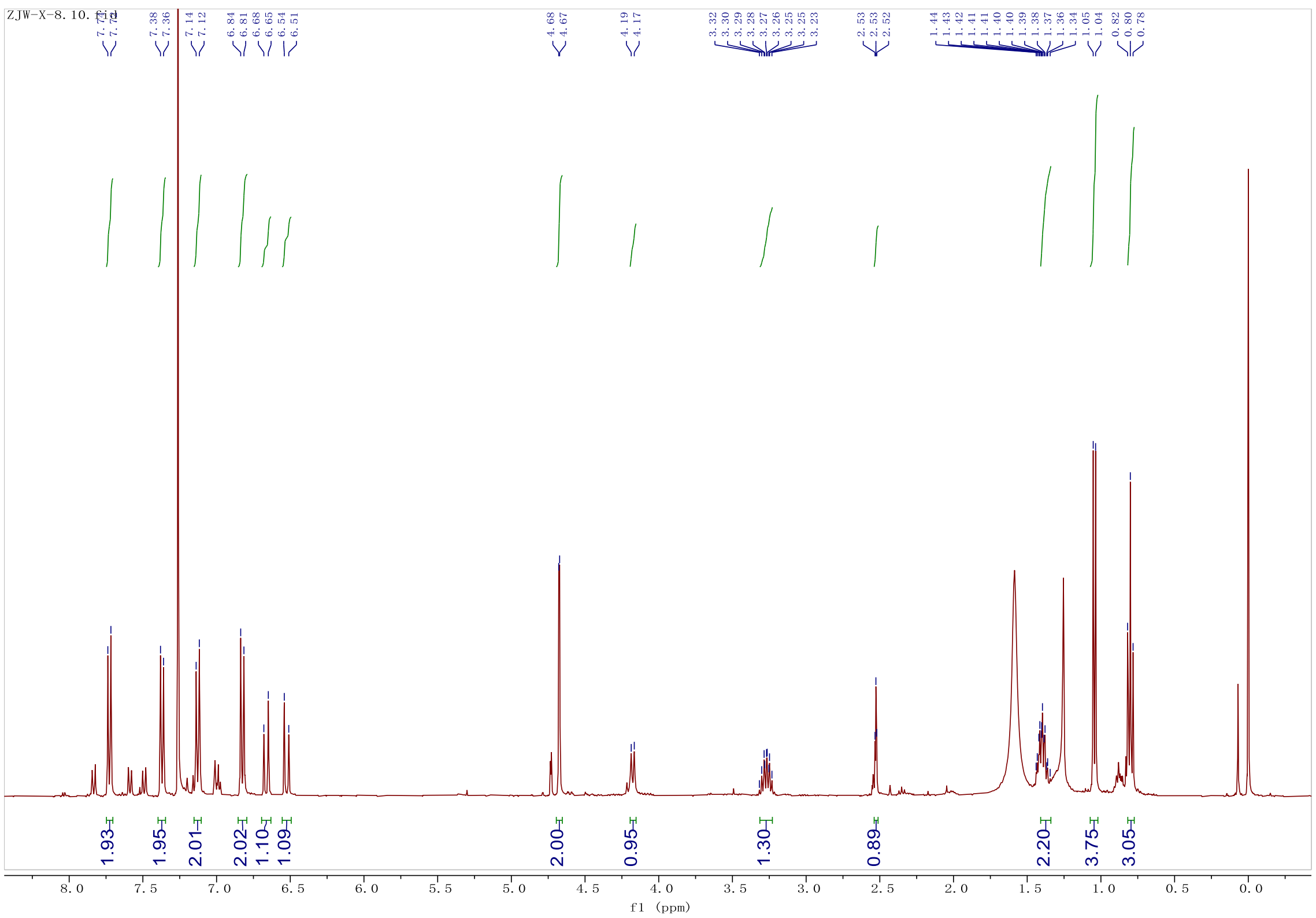

Fig. N73 ^{1}H NMR (400 MHz, CDCl3) spectra of product **8-4**

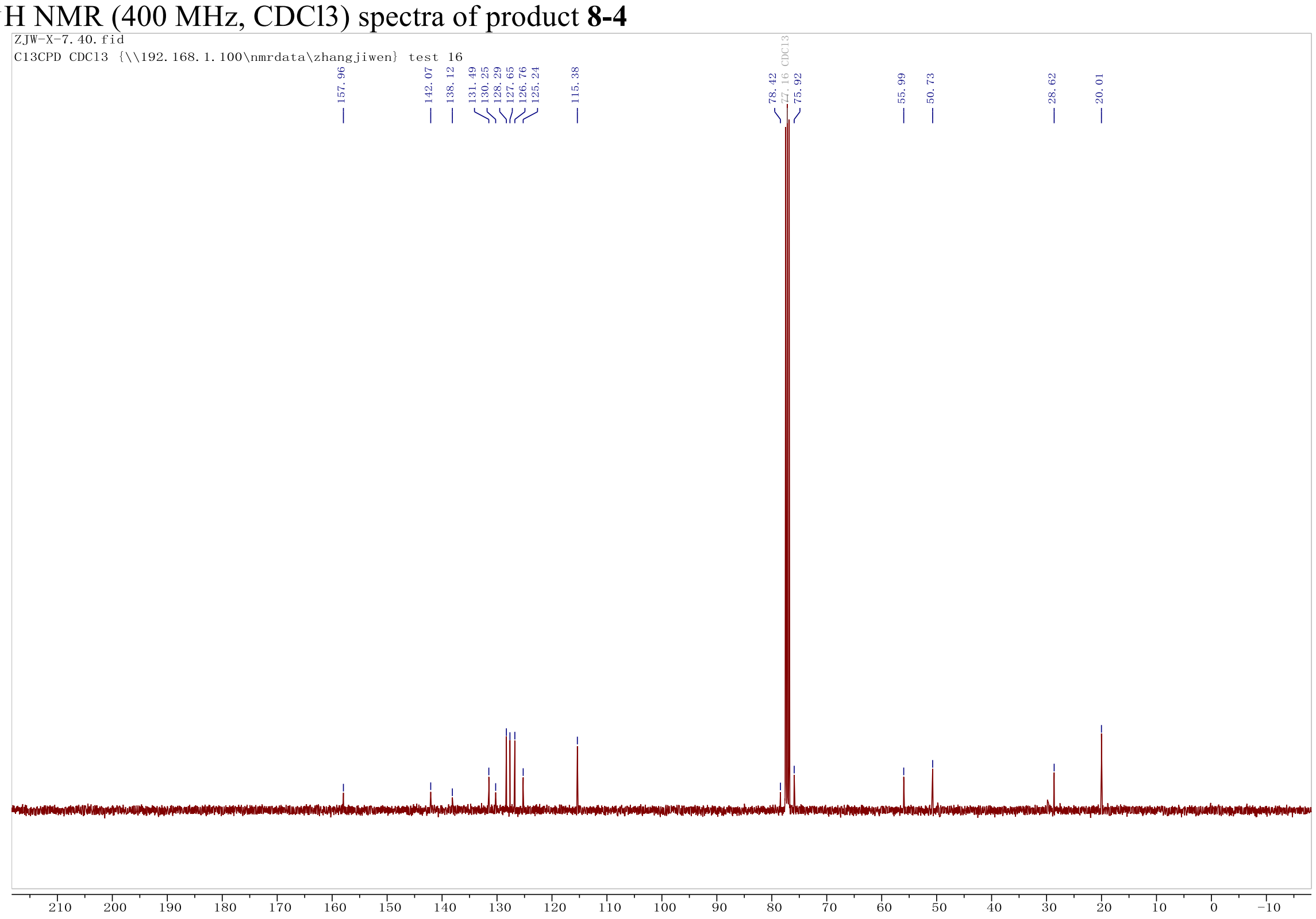

Fig. N74 ^{13}C NMR (101 MHz, CDCl3) spectra of product **8-4**

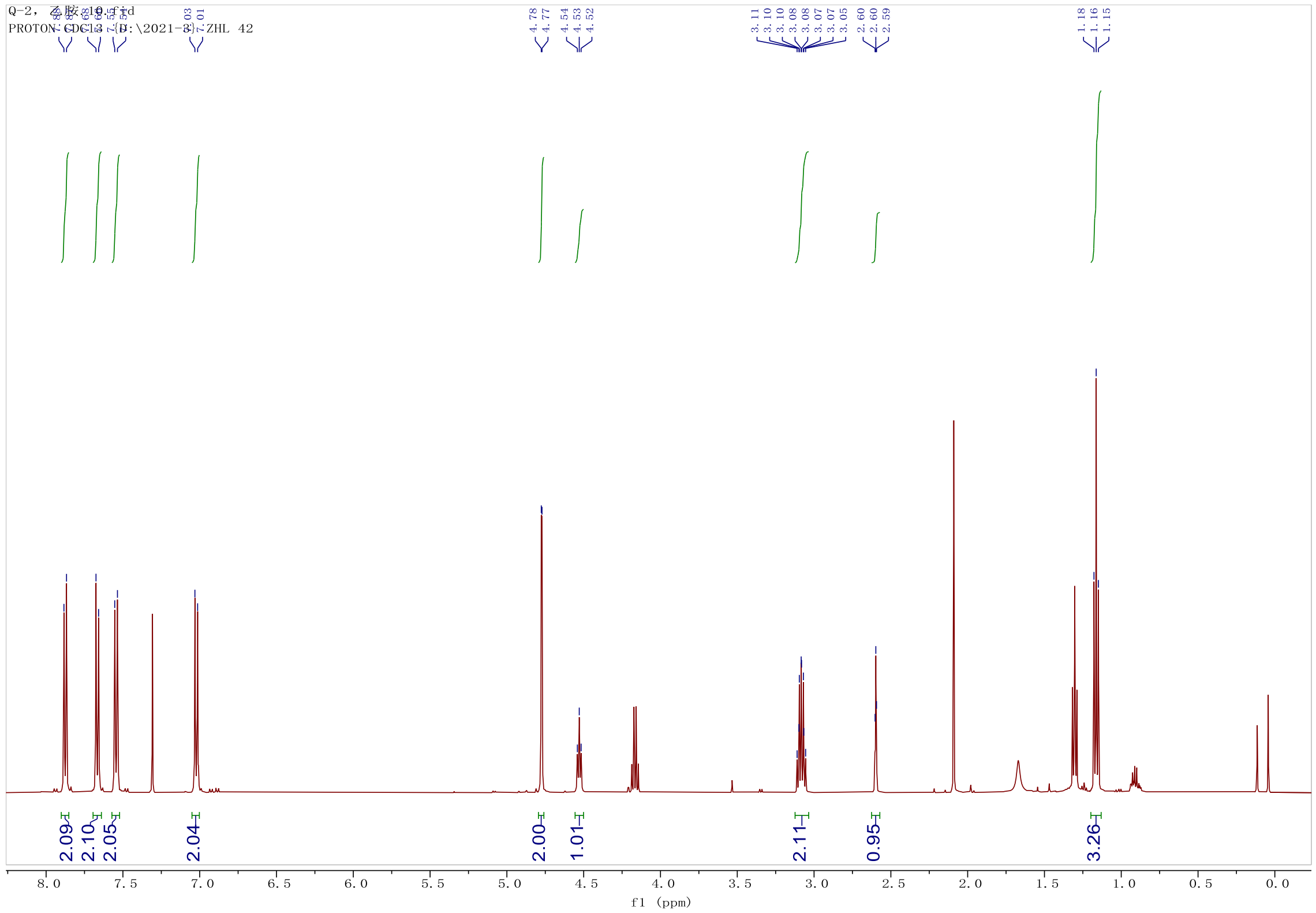

Fig. N75 ^{1}H NMR (500 MHz, CDCl3) spectra of product **10-1**

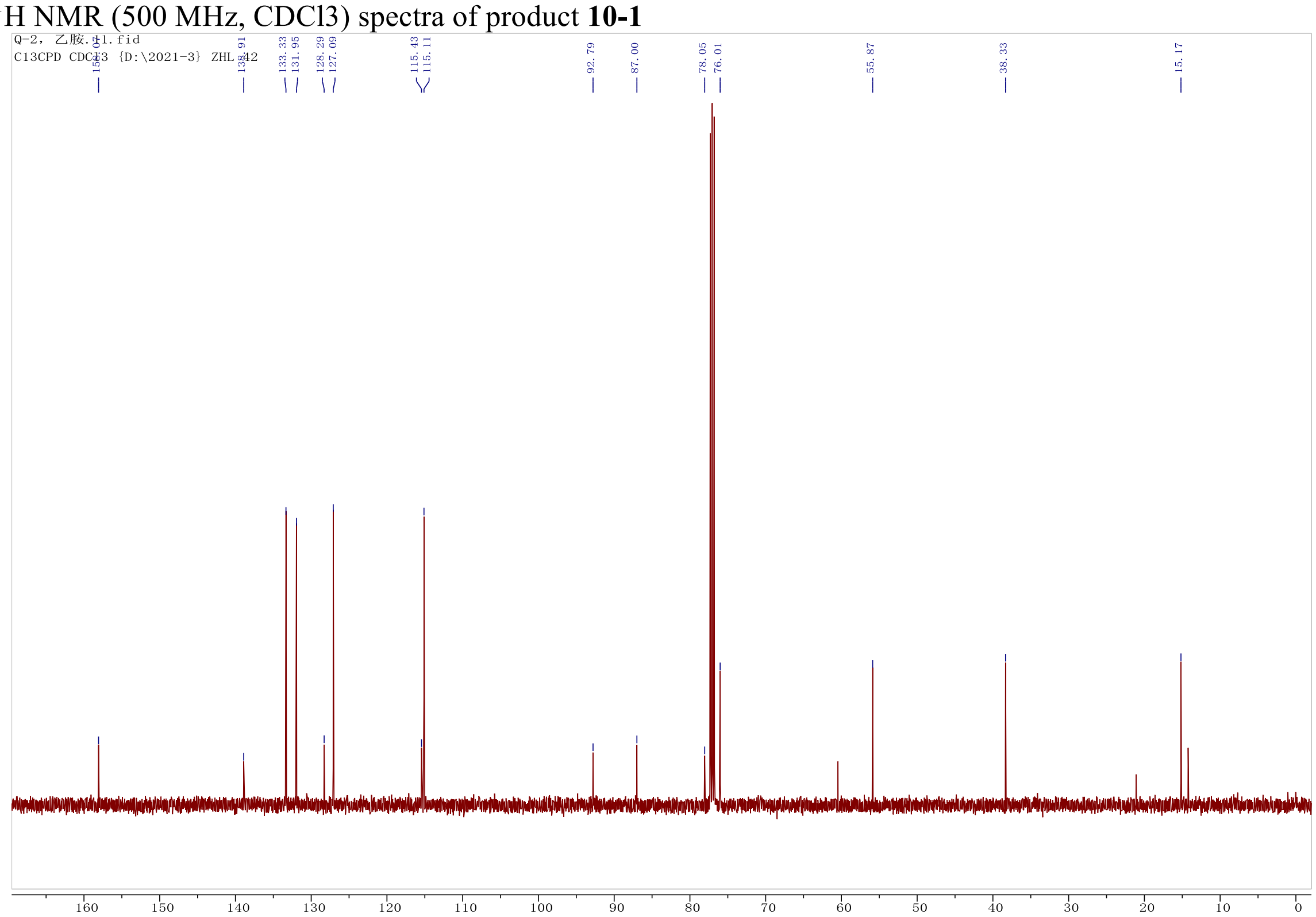

Fig. N76 ^{13}C NMR (126 MHz, CDCl3) spectra of product **10-1**

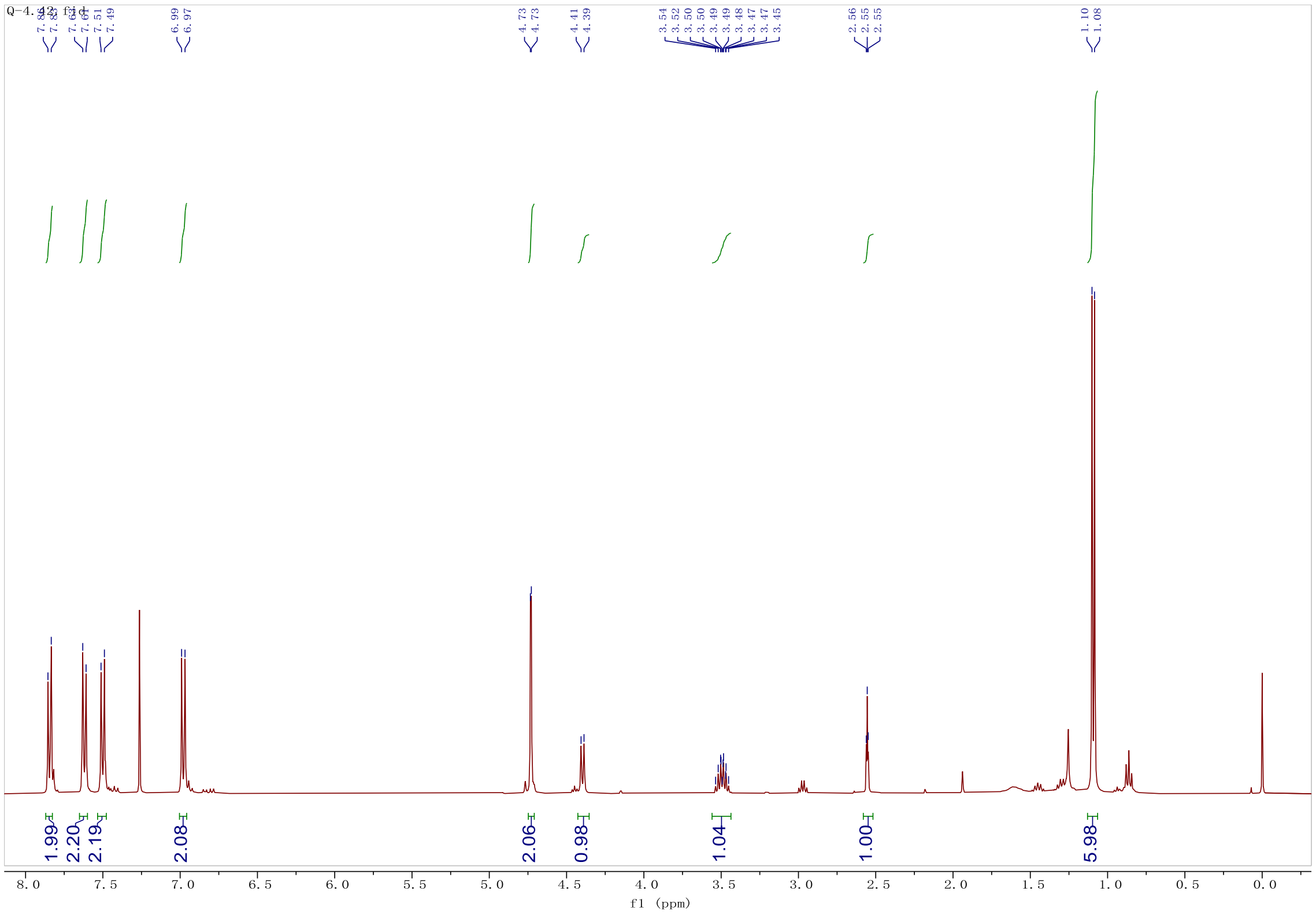

Fig. N77 ^{1}H NMR (400 MHz, CDCl3) spectra of product **10-2**

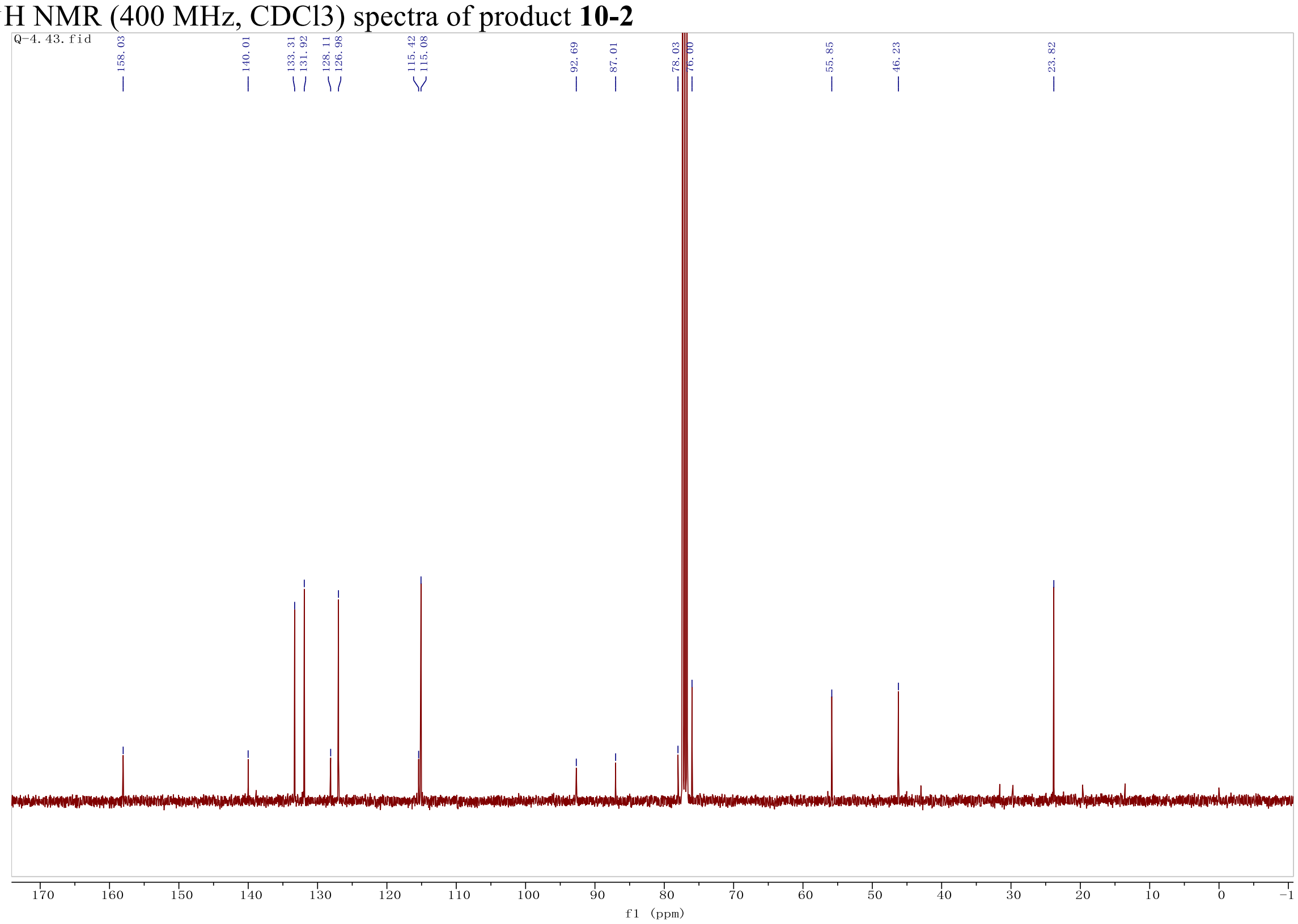

Fig. N78 ^{13}C NMR (101 MHz, CDCl3) spectra of product **10-2**

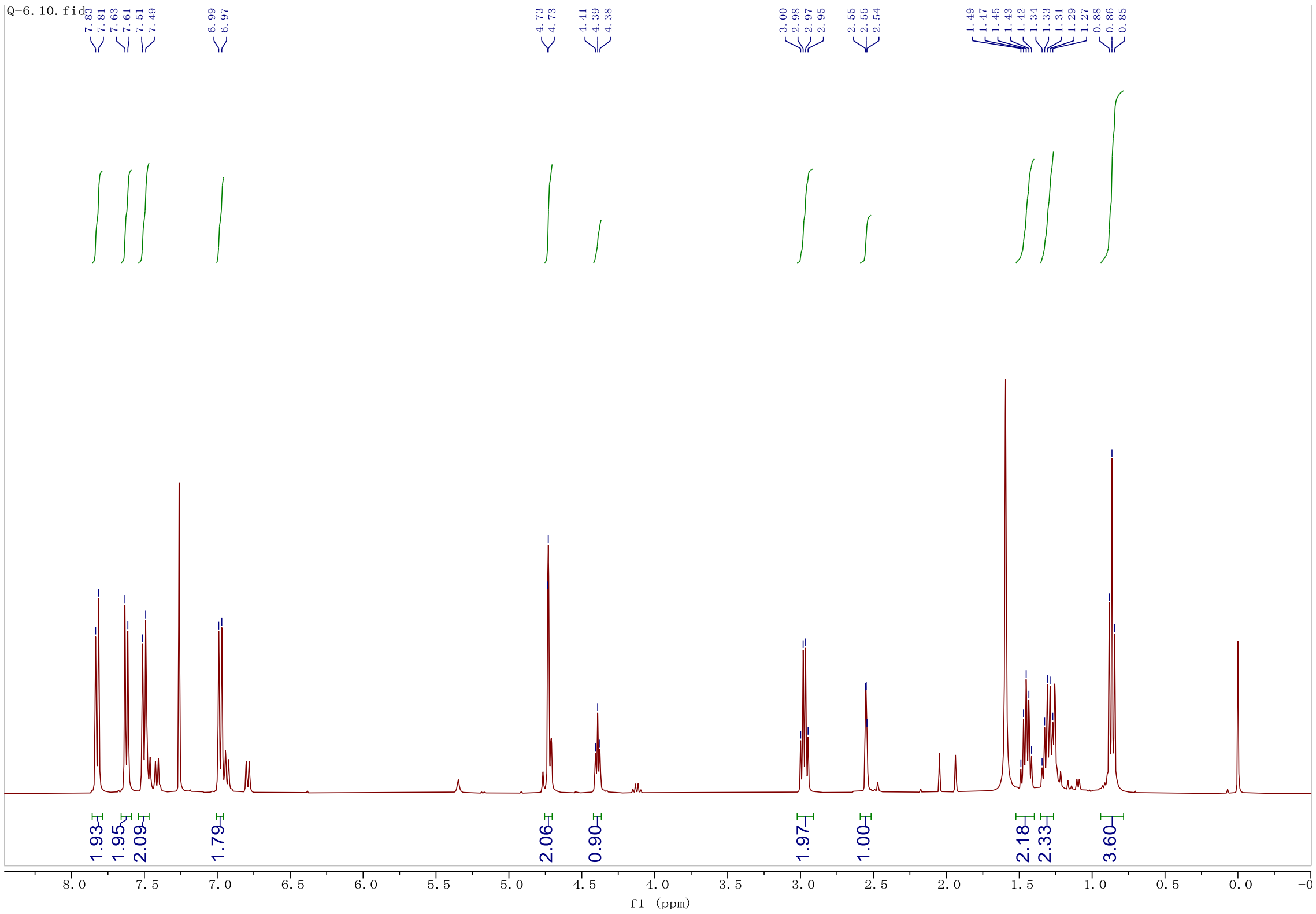

Fig. N79 ^{1}H NMR (400 MHz, CDCl3) spectra of product **10-3**

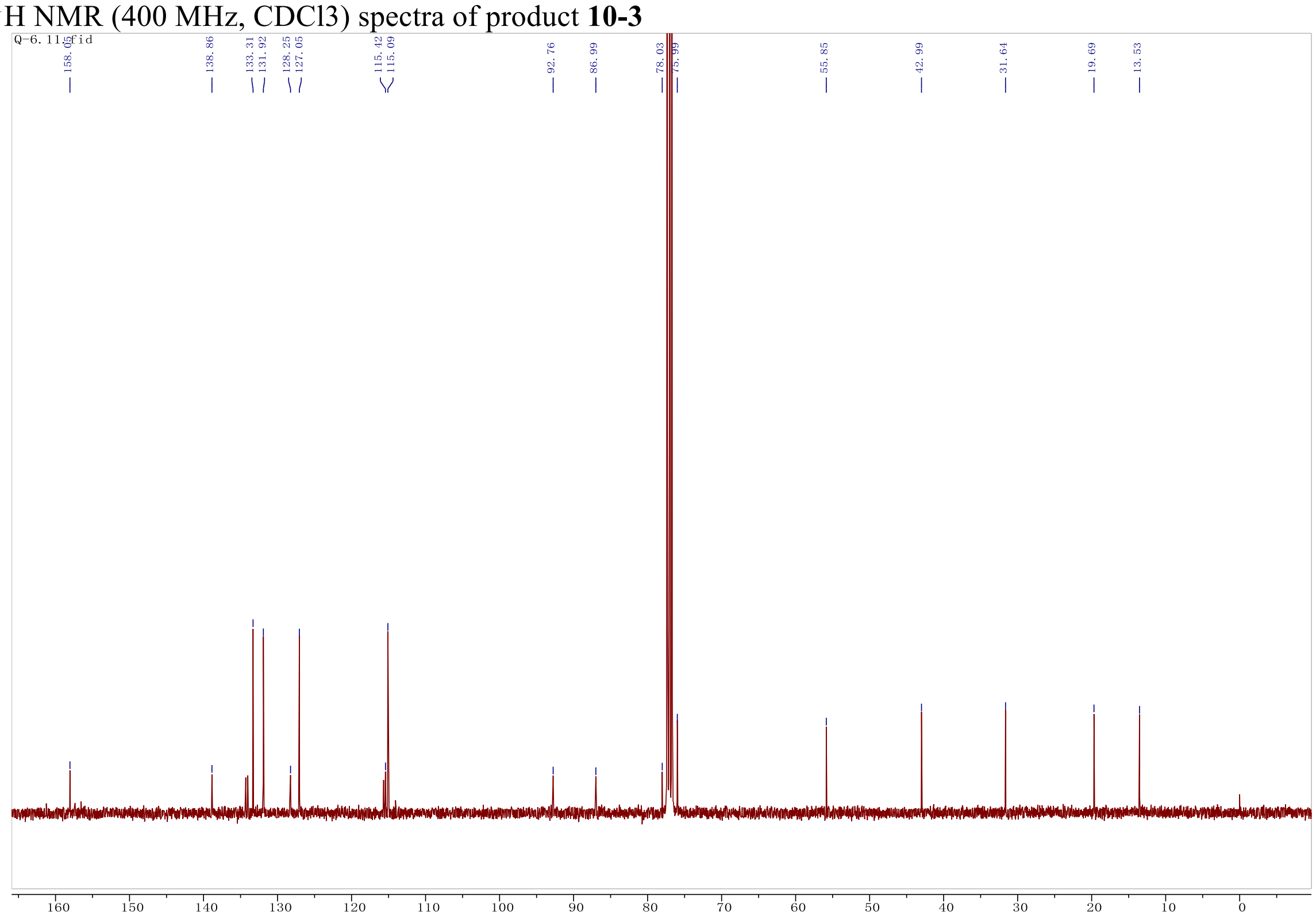

Fig. N80 ^{13}C NMR (101 MHz, CDCl3) spectra of product **10-3**

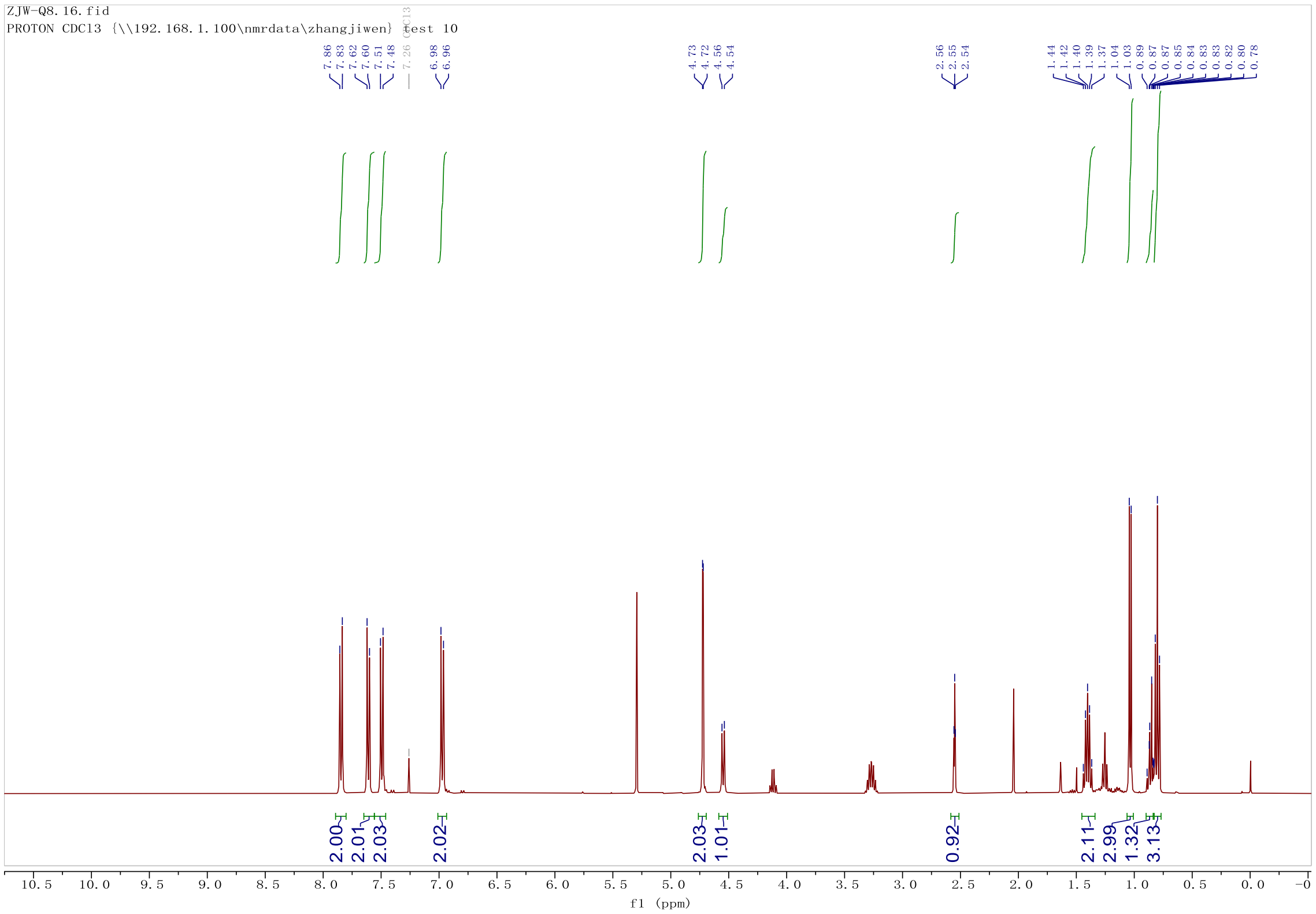

Fig. N81 ^{1}H NMR (400 MHz, CDCl3) spectra of product **10-4**

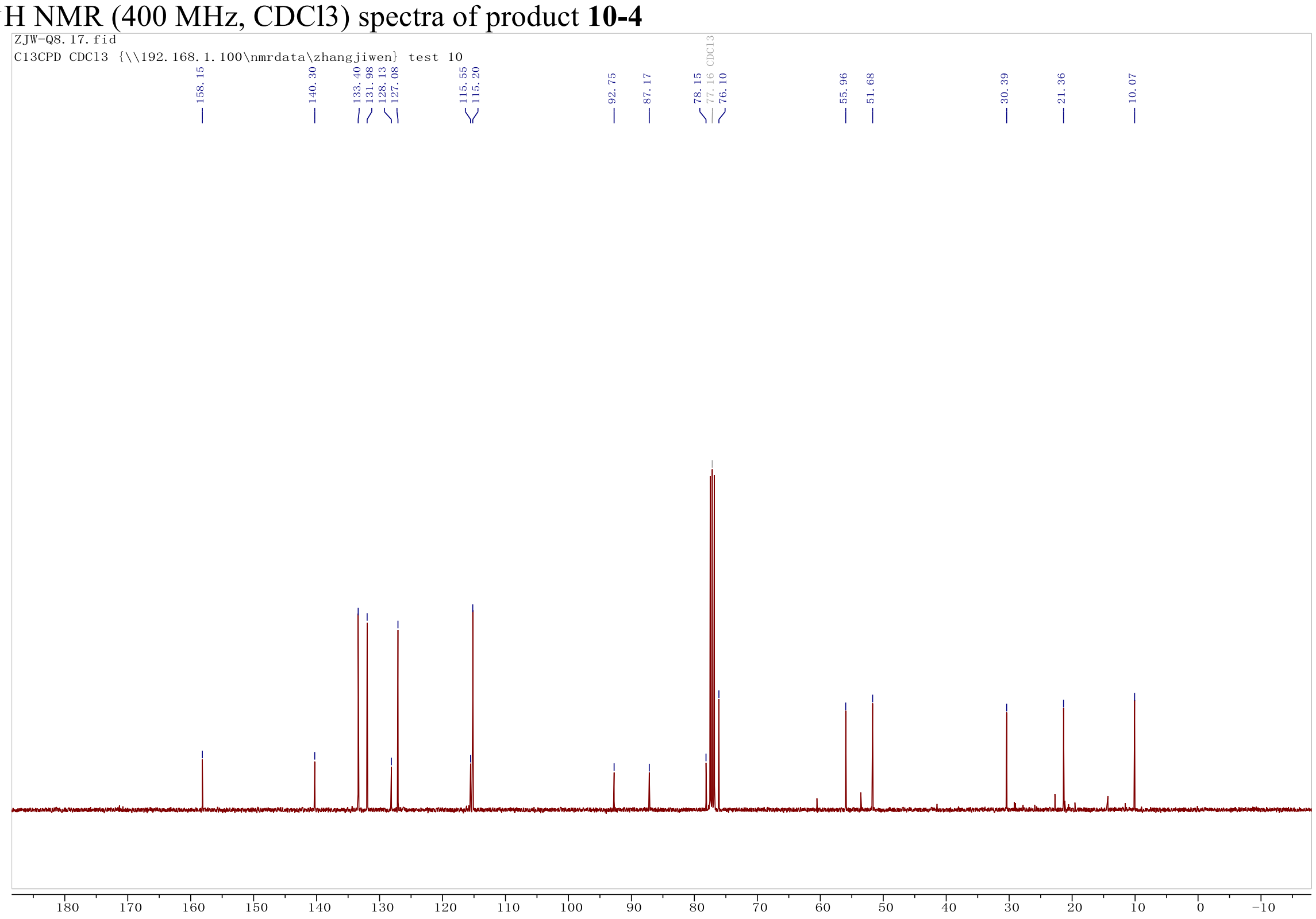

Fig. N82 ^{13}C NMR (101 MHz, CDCl3) spectra of product **10-4**

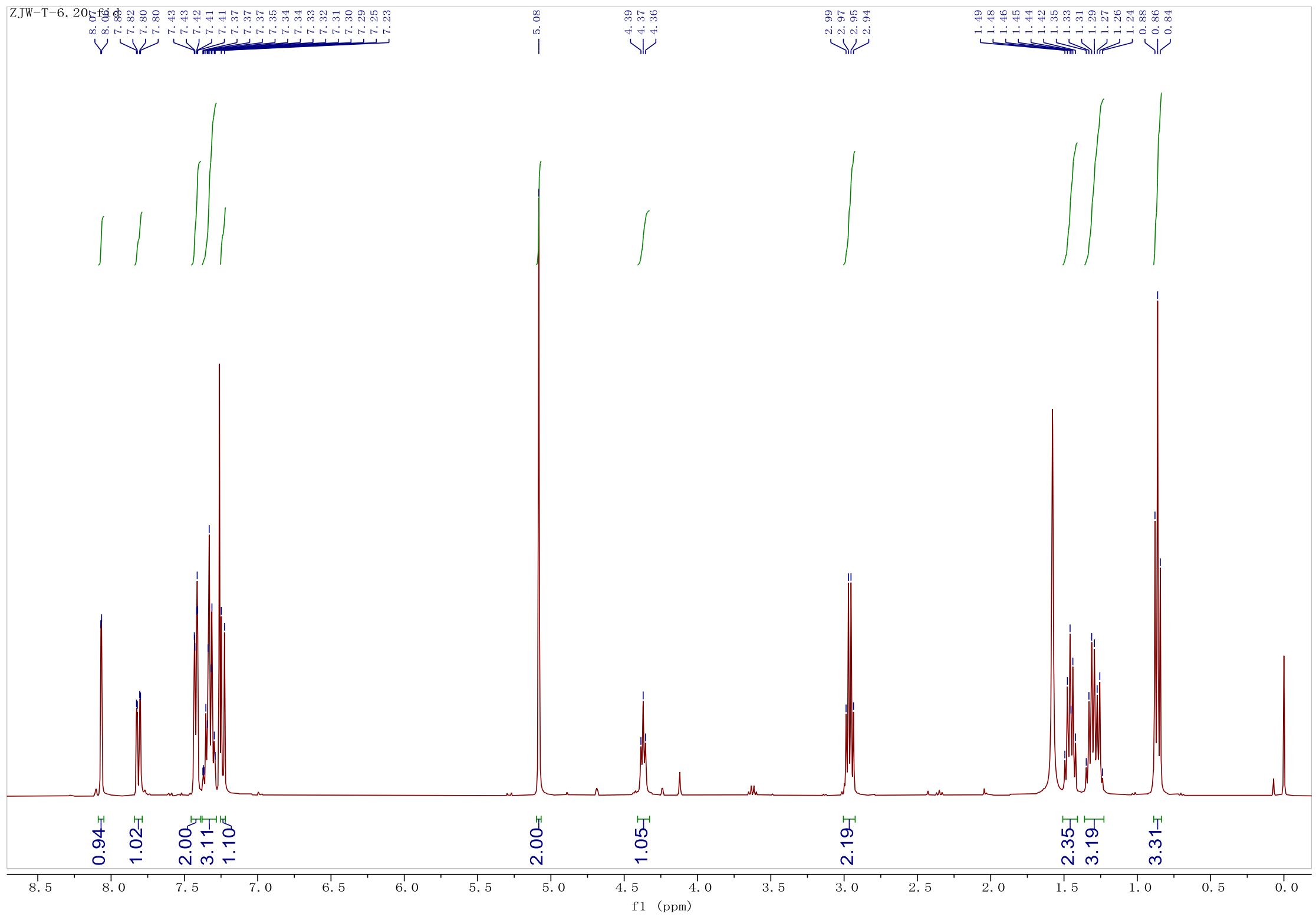

Fig. N83 ^{1}H NMR (400 MHz, CDCl3) spectra of product **S4-1**

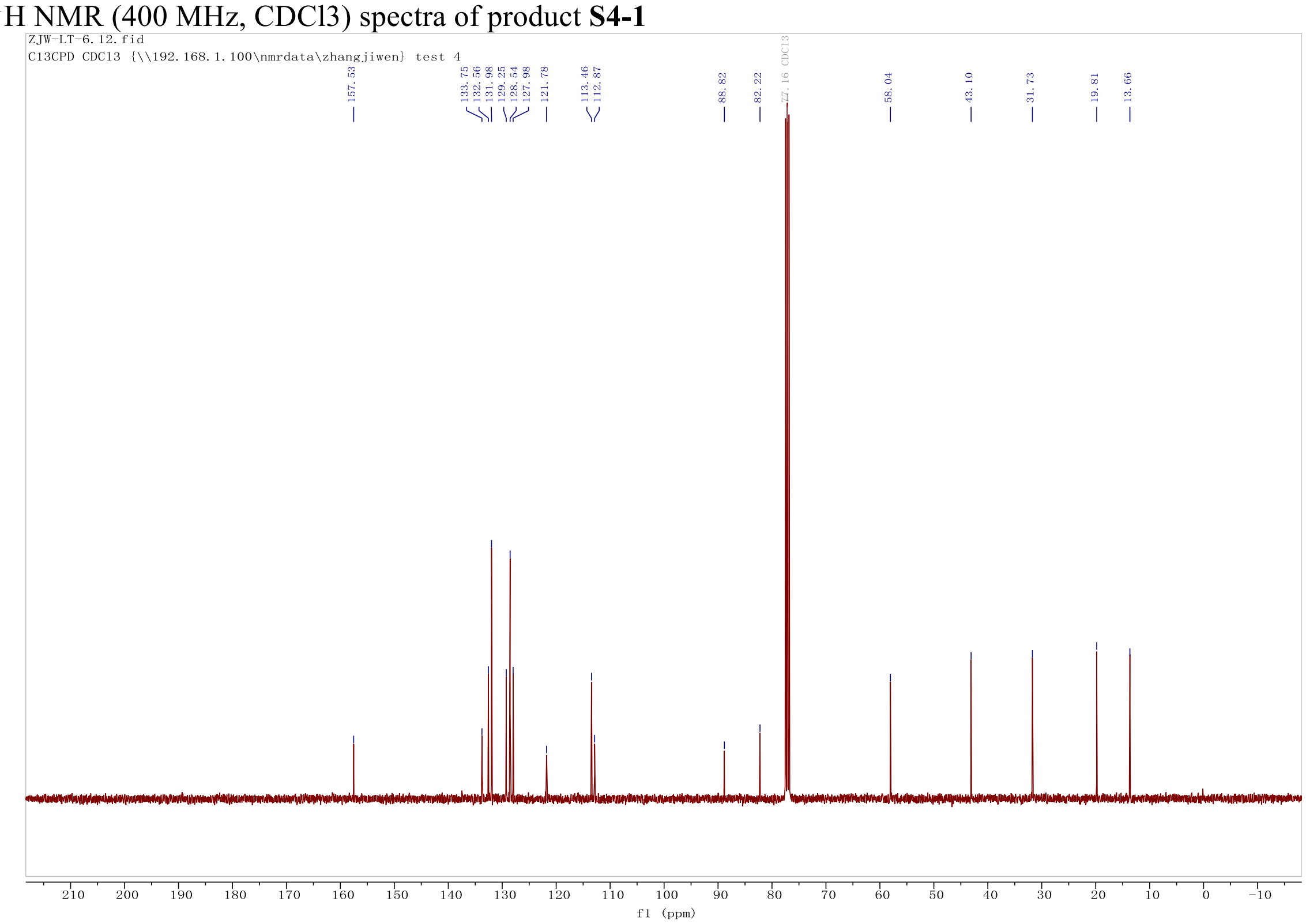

Fig. N84 ^{13}C NMR (101 MHz, CDCl3) spectra of product **S4-1**

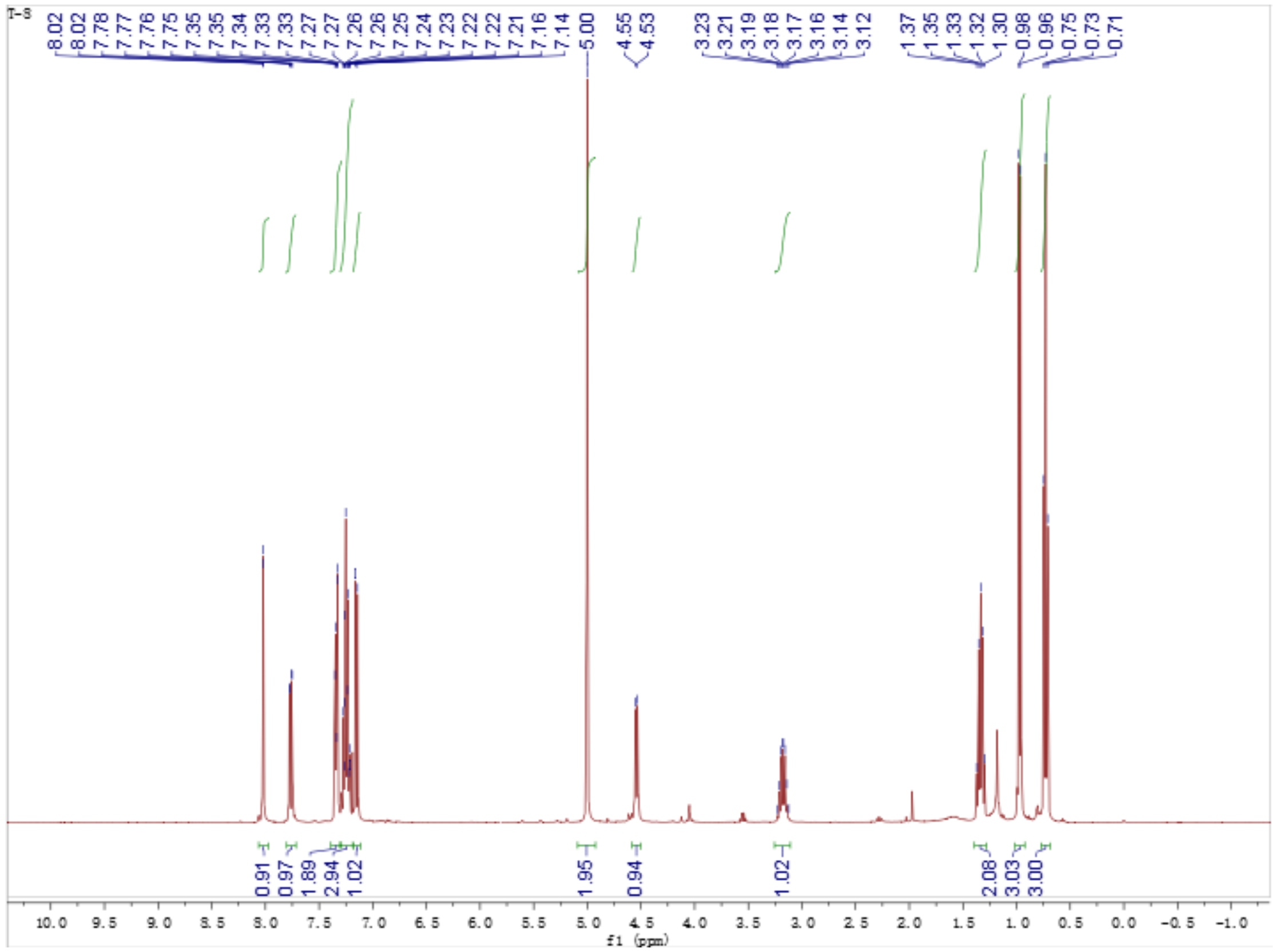

Fig. N85 ^{1}H NMR (400 MHz, CDCl3) spectra of product **S4-2**

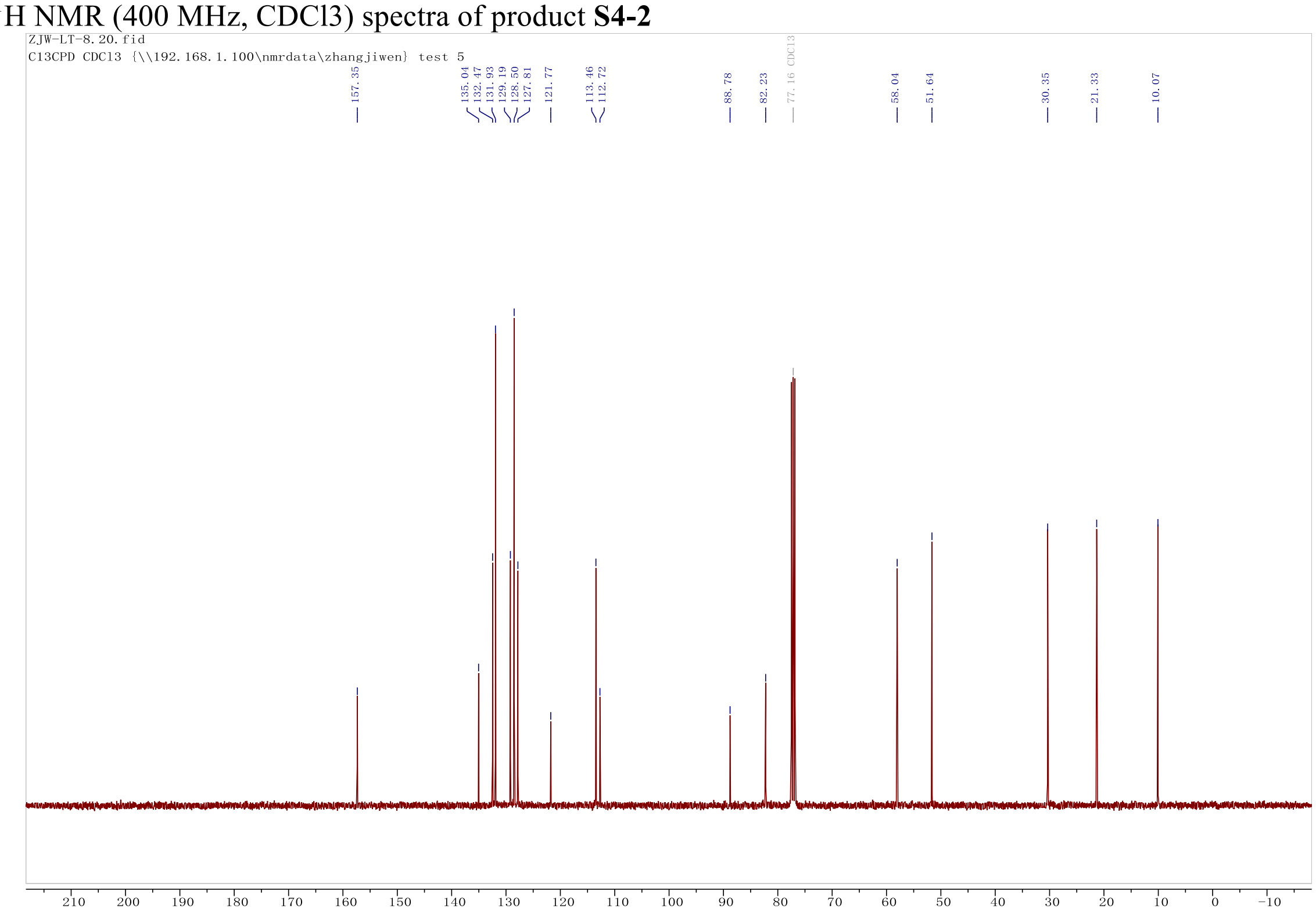

Fig. N86 ^{13}C NMR (101 MHz, CDCl3) spectra of product **S4-2**

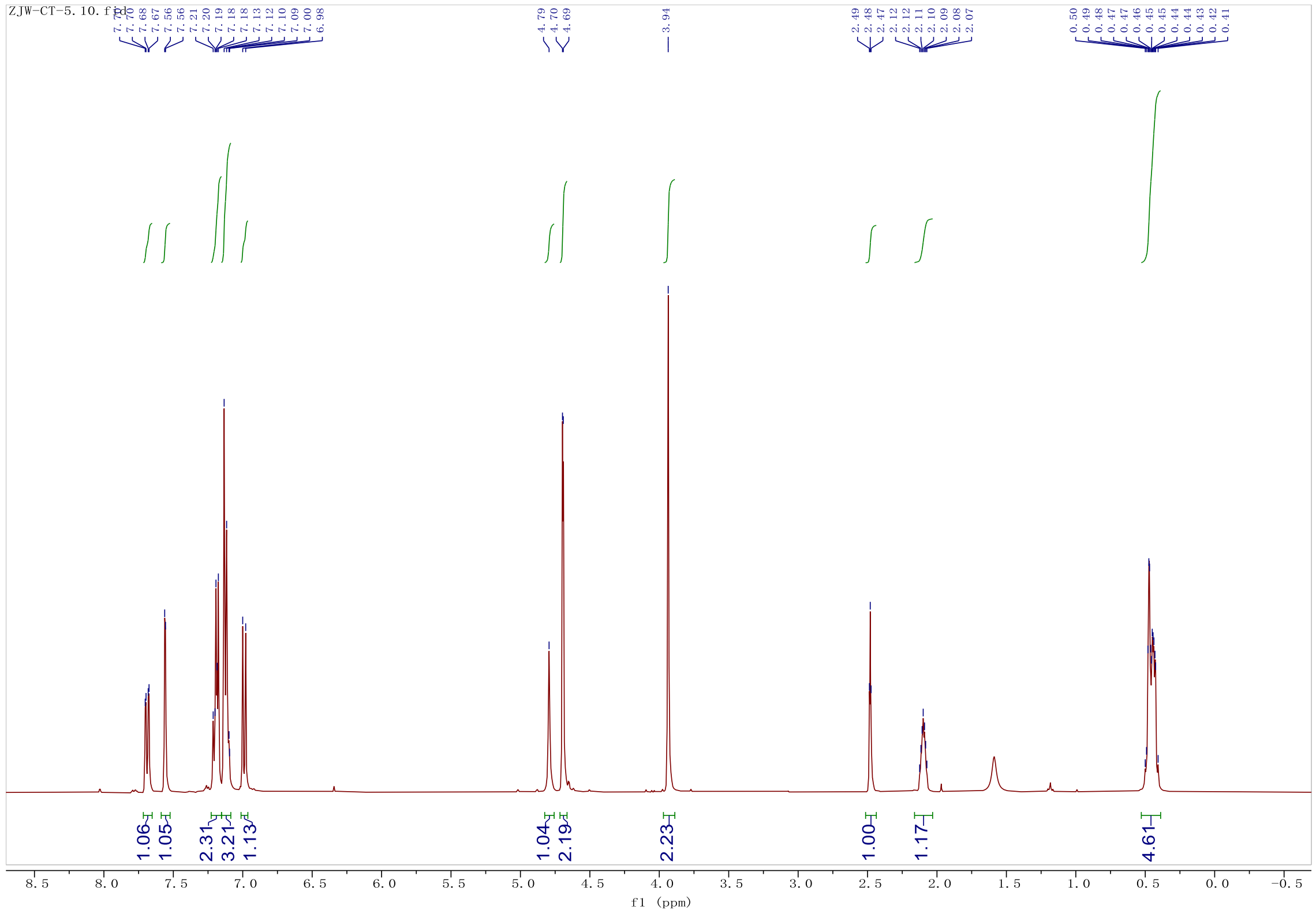

Fig. N87 ^{1}H NMR (400 MHz, CDCl3) spectra of product **S6-1**

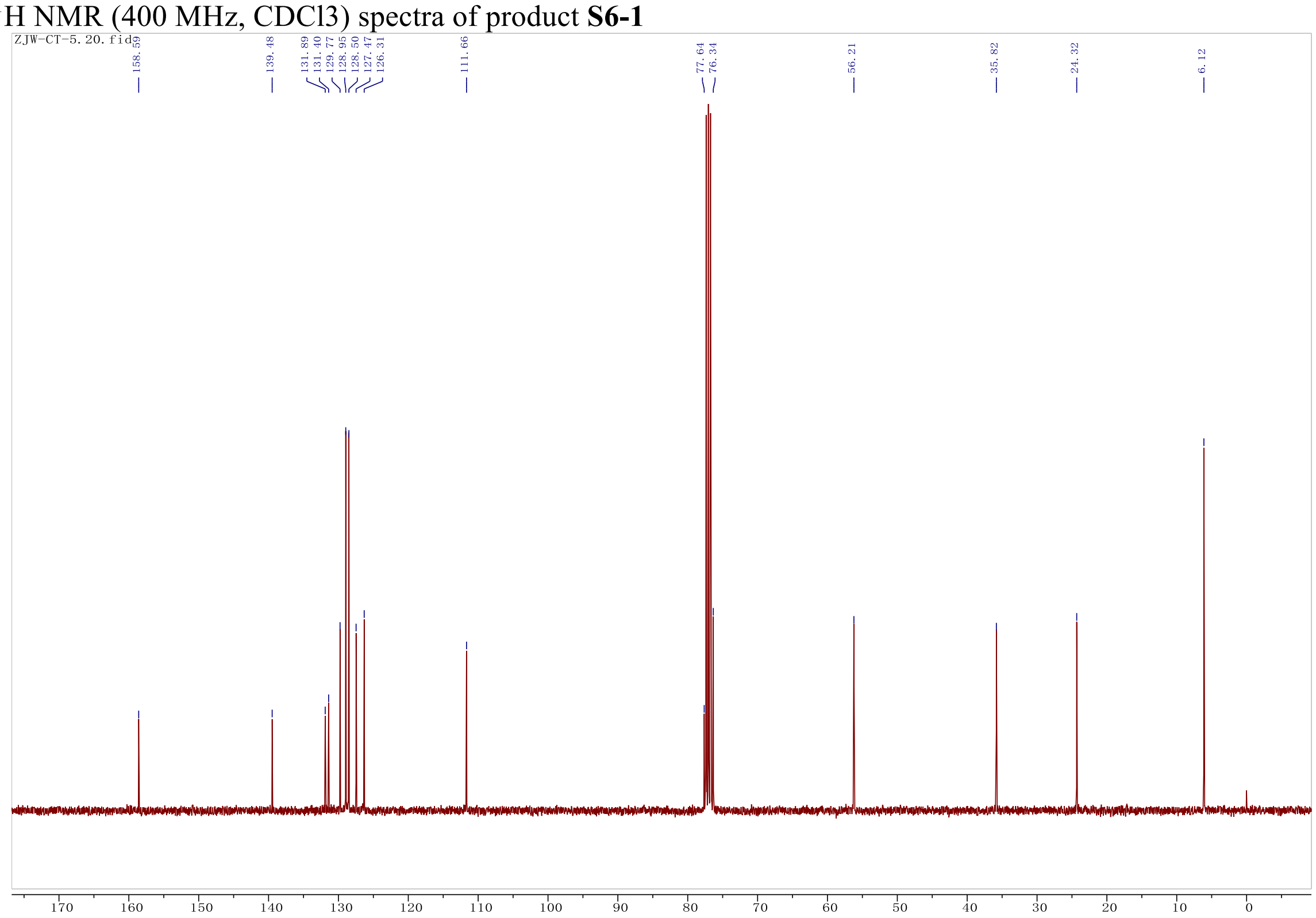

Fig. N88 ^{13}C NMR (101 MHz, CDCl3) spectra of product **S6-1**

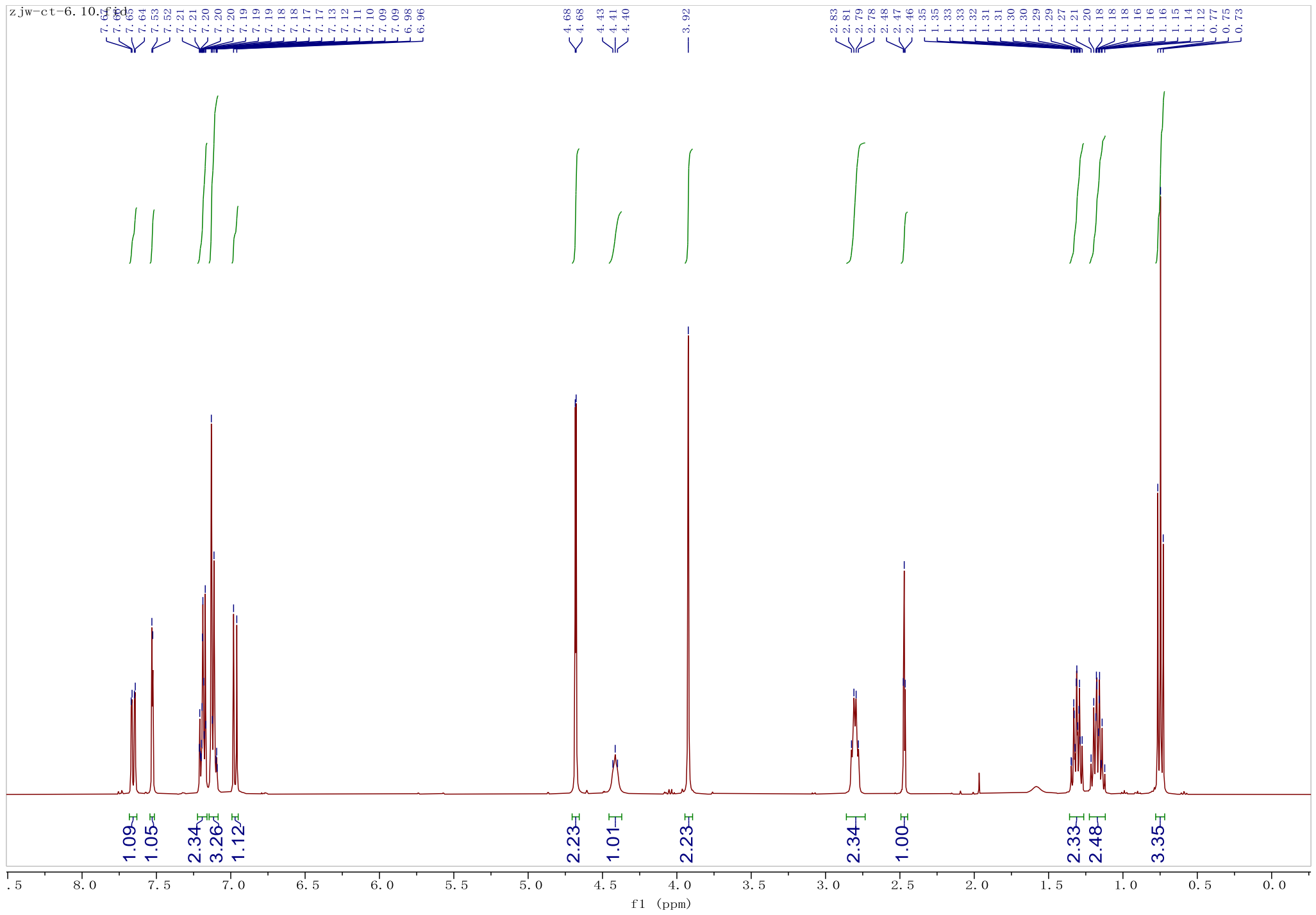

Fig. N89 ^{1}H NMR (400 MHz, CDCl3) spectra of product **S6-2**

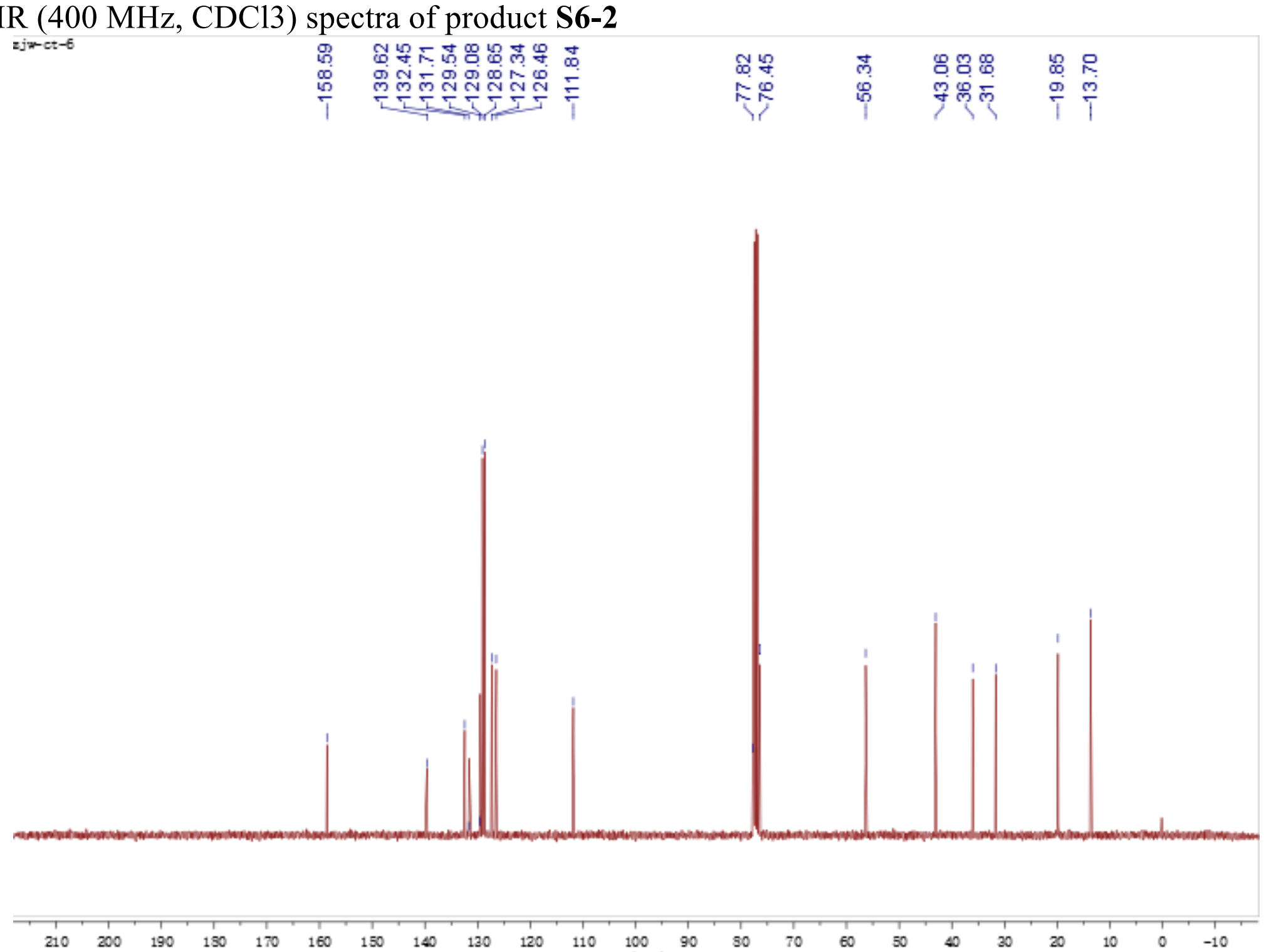

Fig. N90 ^{13}C NMR (101 MHz, CDCl3) spectra of product **S6-2**

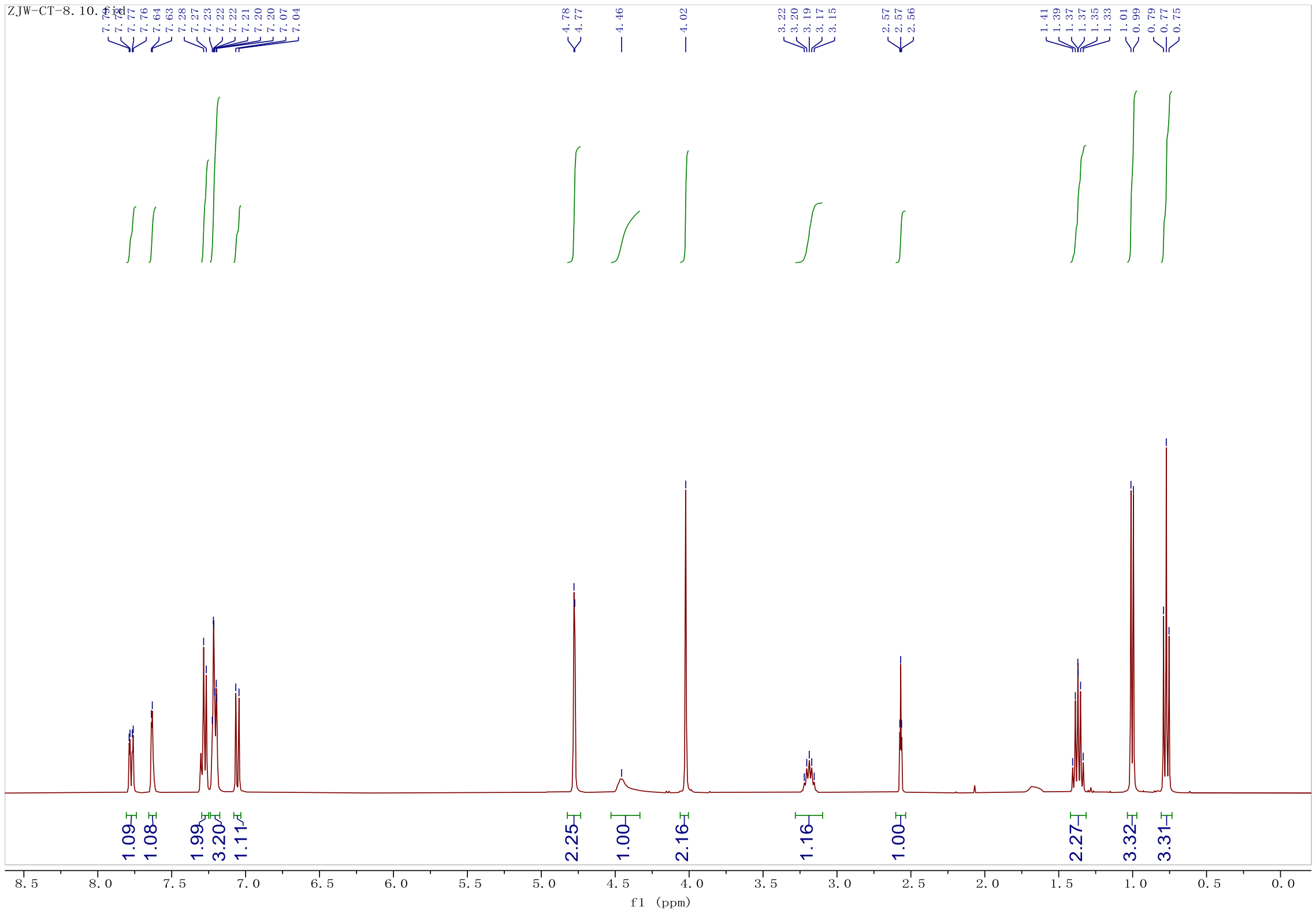

Fig. 91 ^{1}H NMR (400 MHz, CDCl3) spectra of product **S6-3**

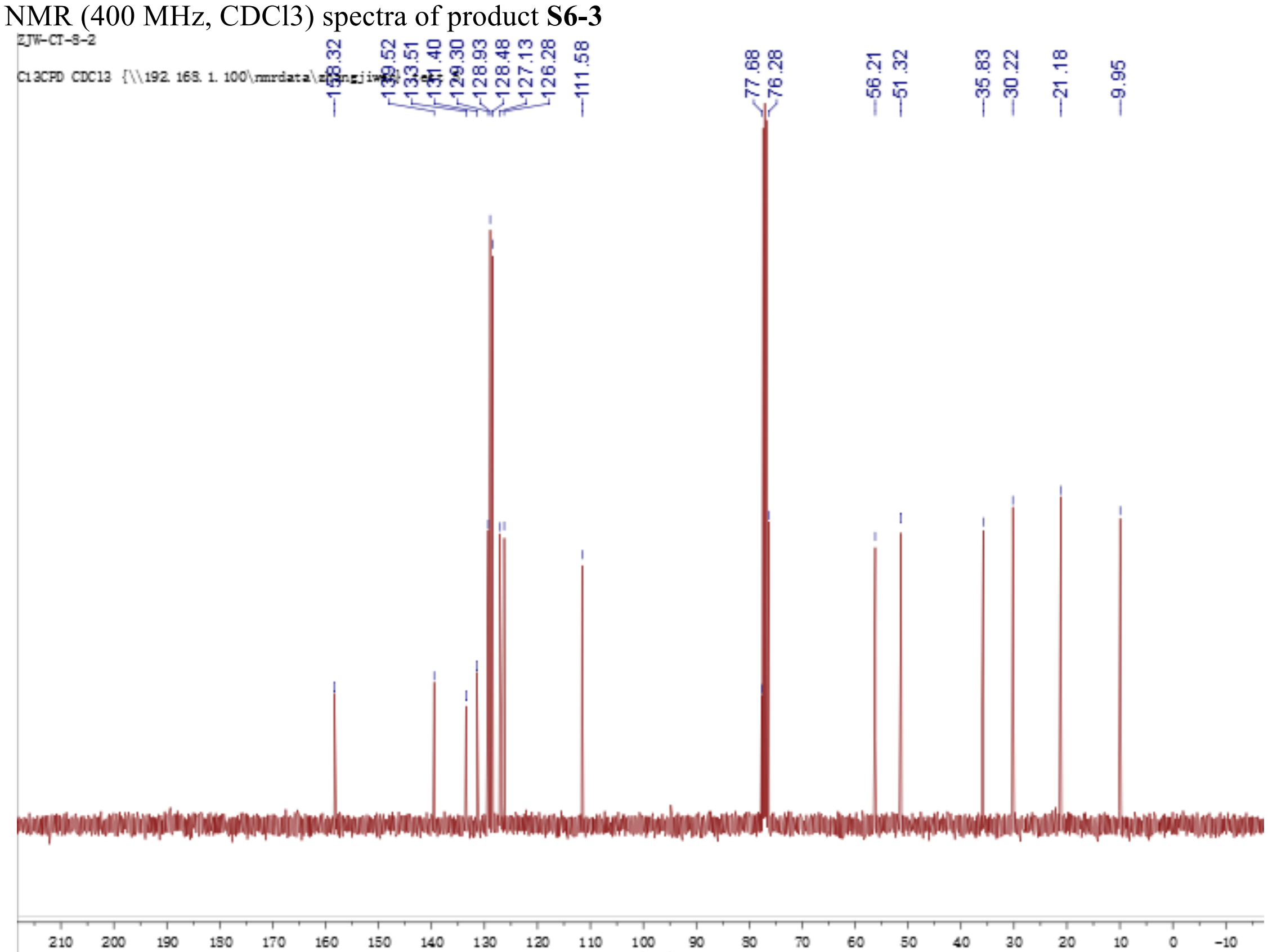

Fig. N92 ^{13}C NMR (101 MHz, CDCl3) spectra of product **S6-3**

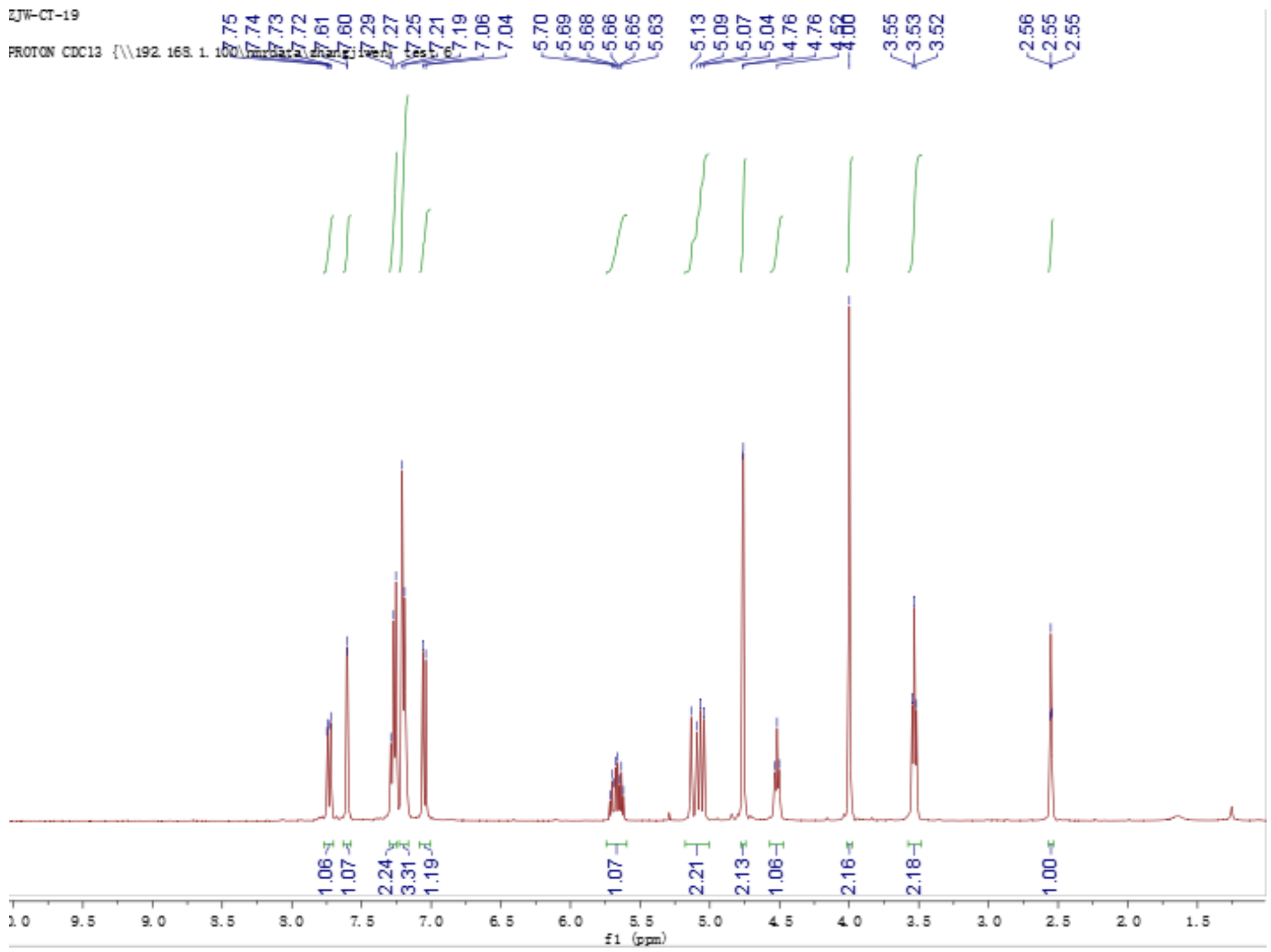

Fig. N93 ^{1}H NMR (400 MHz, CDCl3) spectra of product **S6-4**

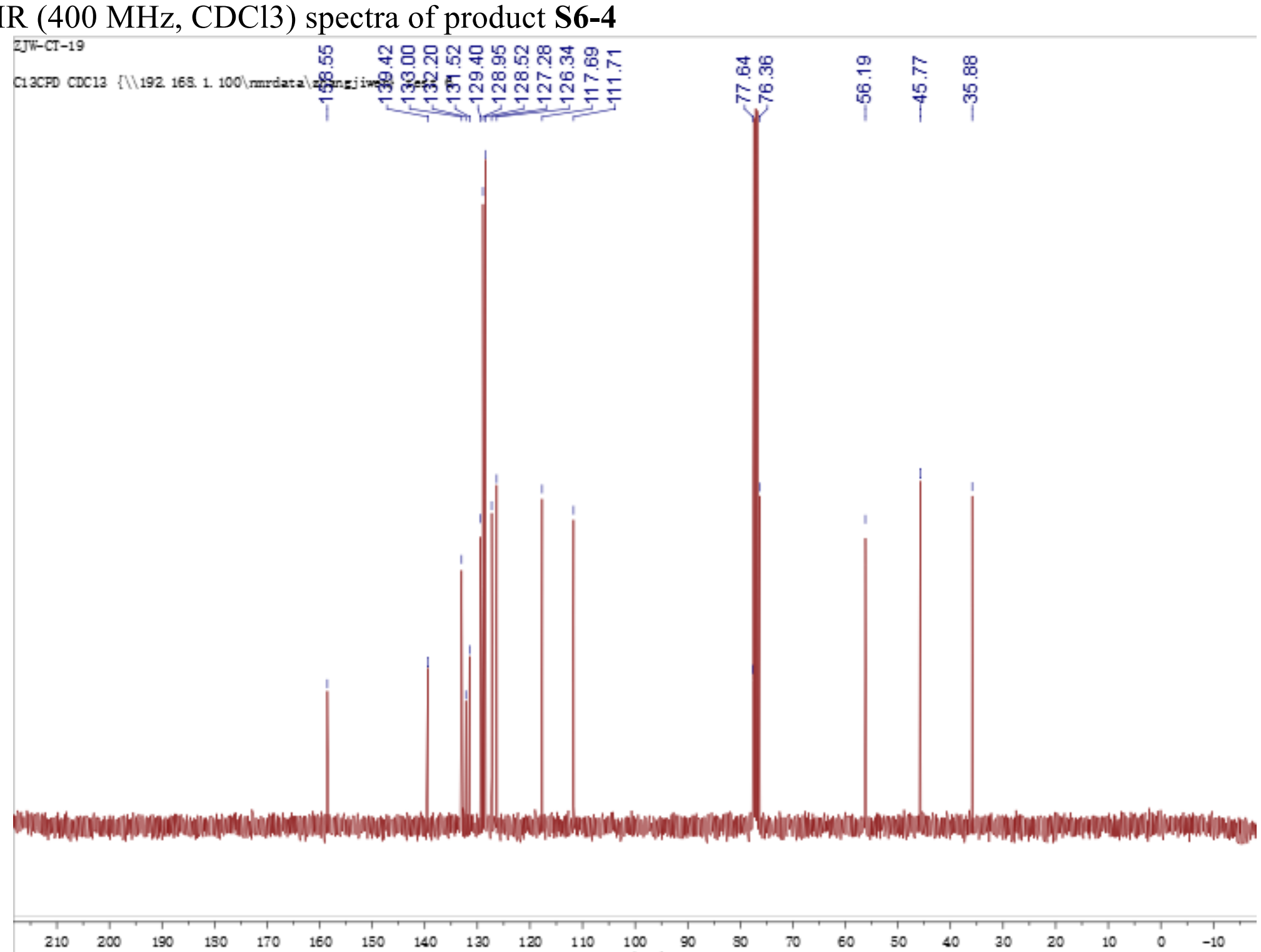

Fig. N94 ^{13}C NMR (101 MHz, CDCl3) spectra of product **S6-4**

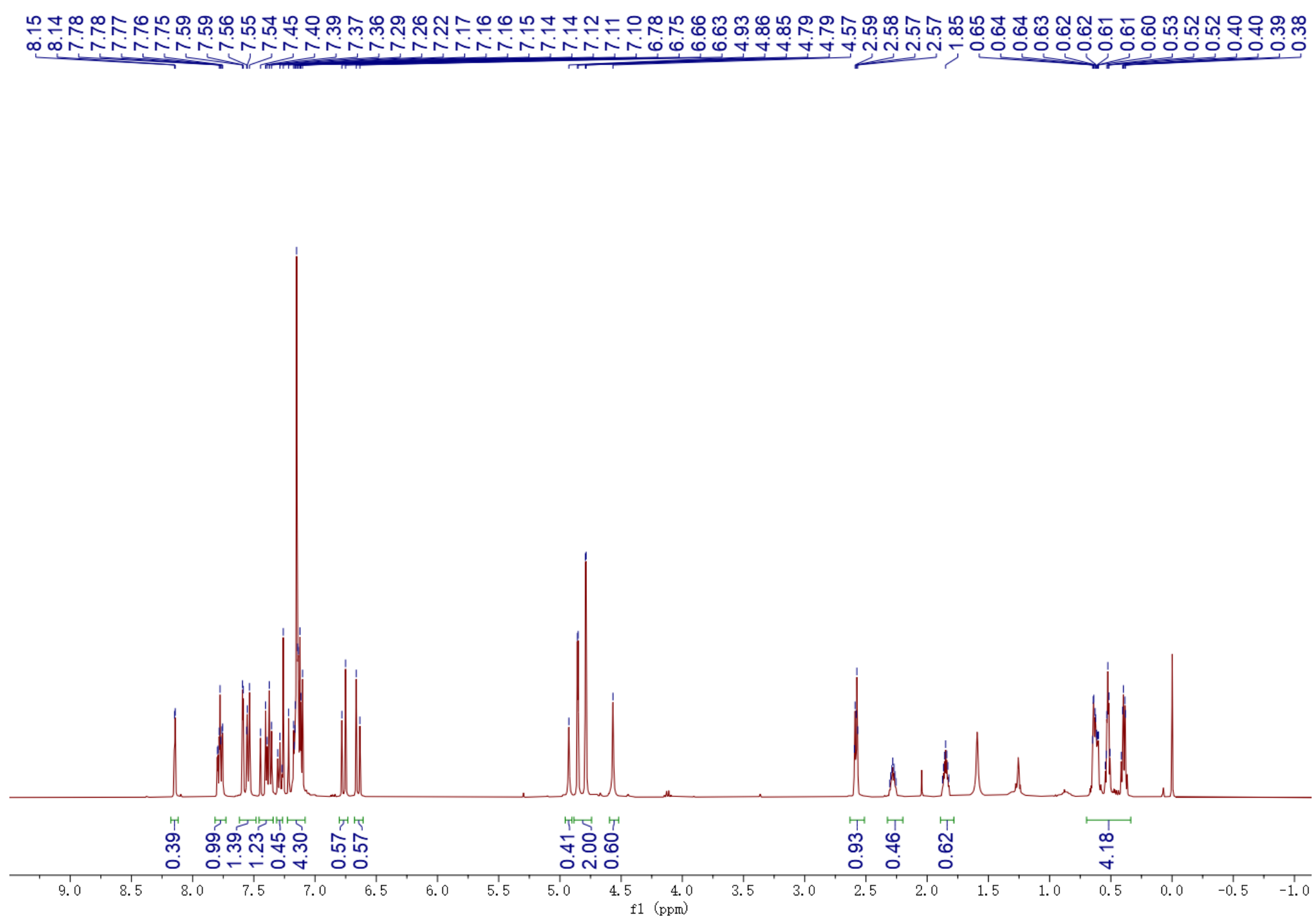

Fig. N95 ^{1}H NMR (400 MHz, CDCl3) spectra of product **S8-1**

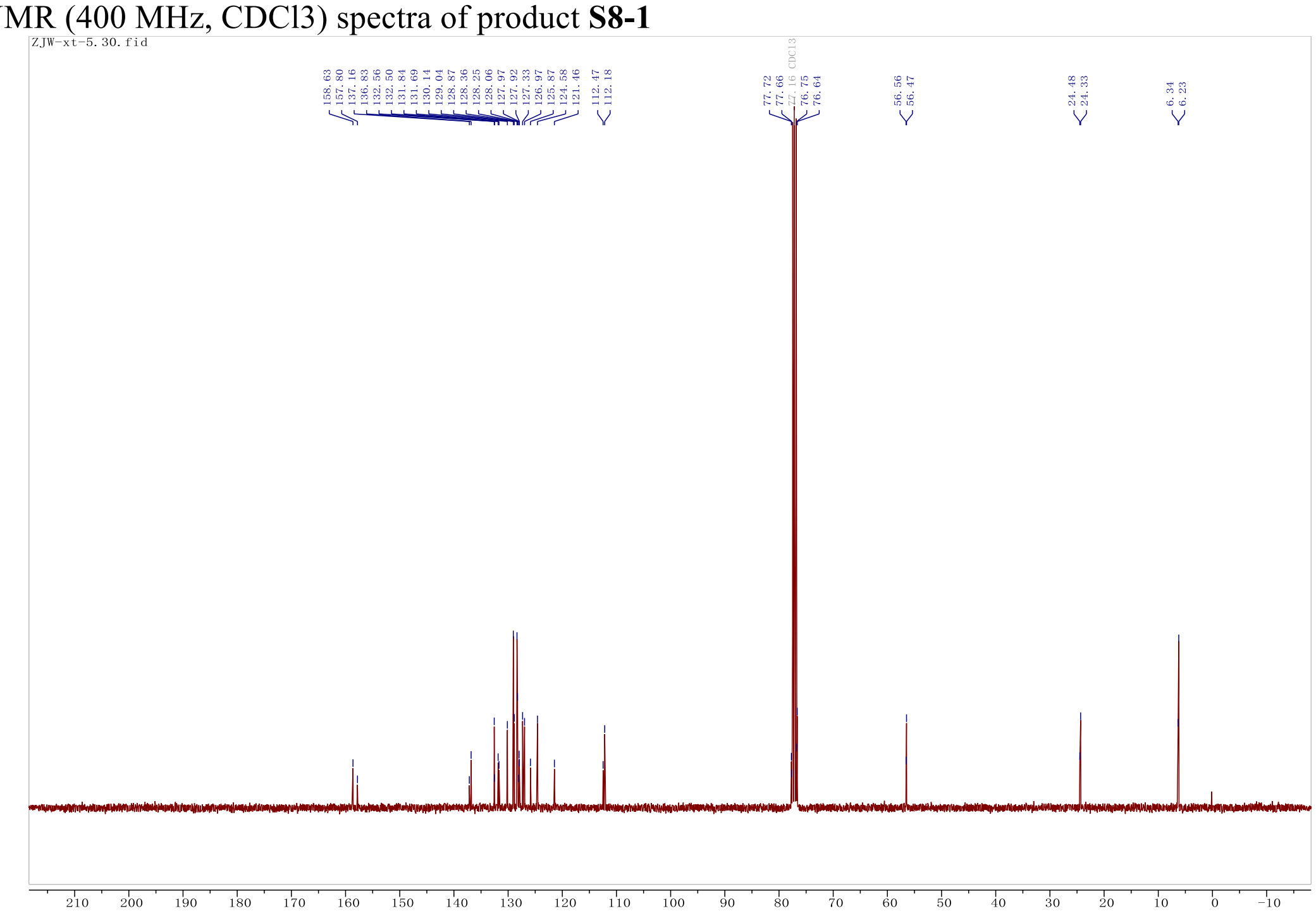

Fig. N96 ^{13}C NMR (101 MHz, CDCl3) spectra of product **S8-1**

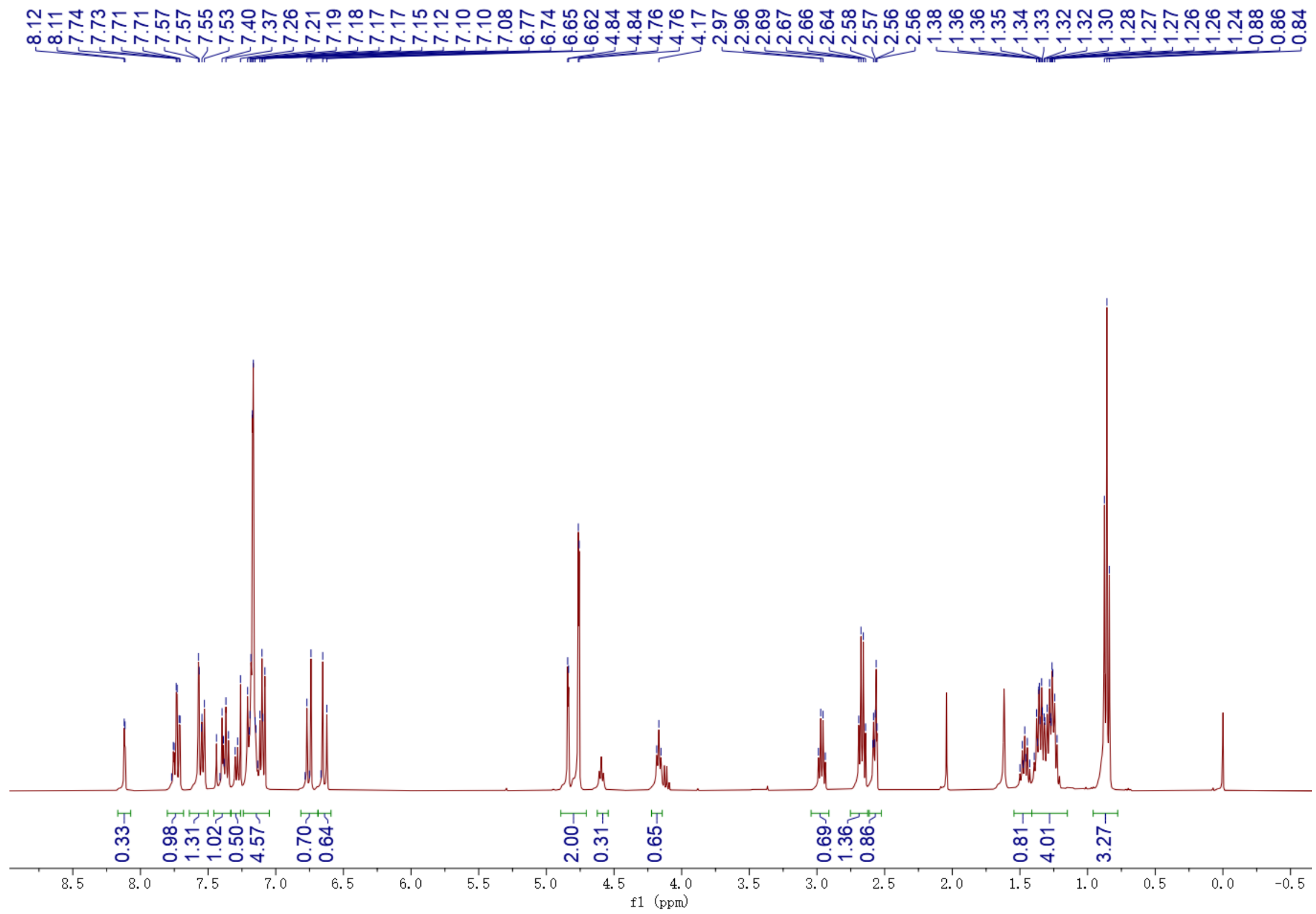

Fig. N97 ^{1}H NMR (400 MHz, CDCl3) spectra of product **S8-2**

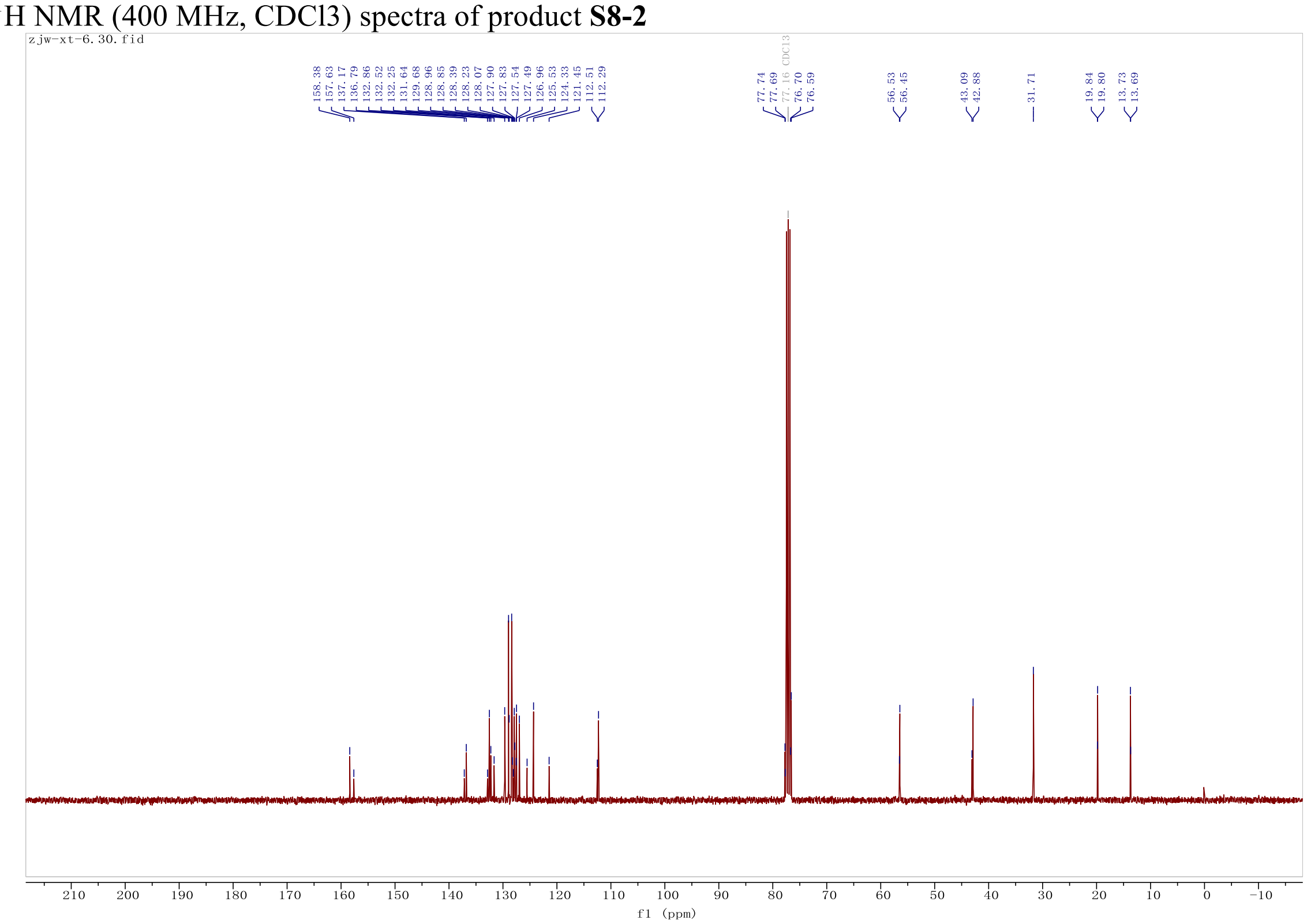

Fig. N98 ^{13}C NMR (101 MHz, CDCl3) spectra of product **S8-2**

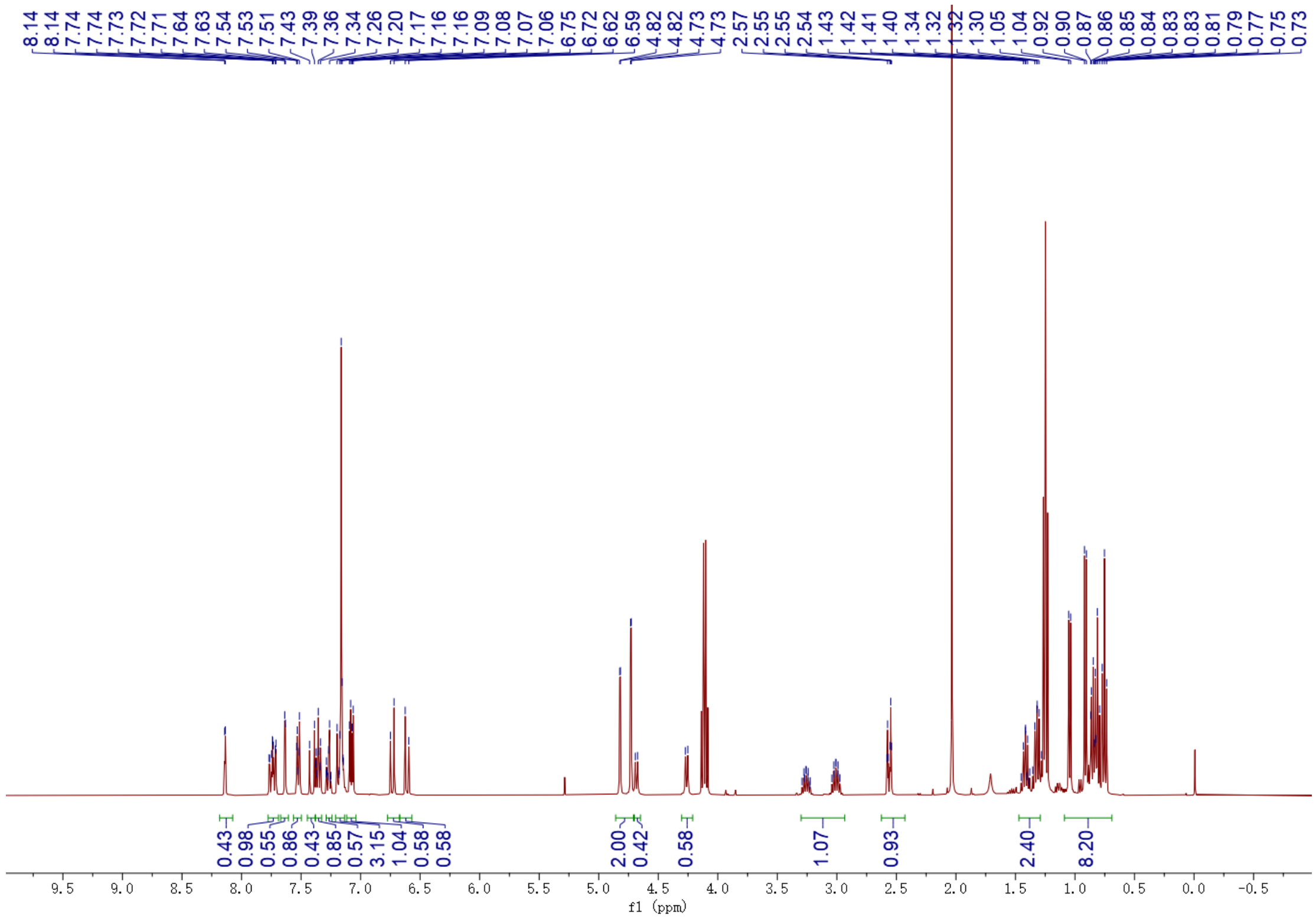

Fig. N99 ^{1}H NMR (400 MHz, CDCl3) spectra of product **S8-3**

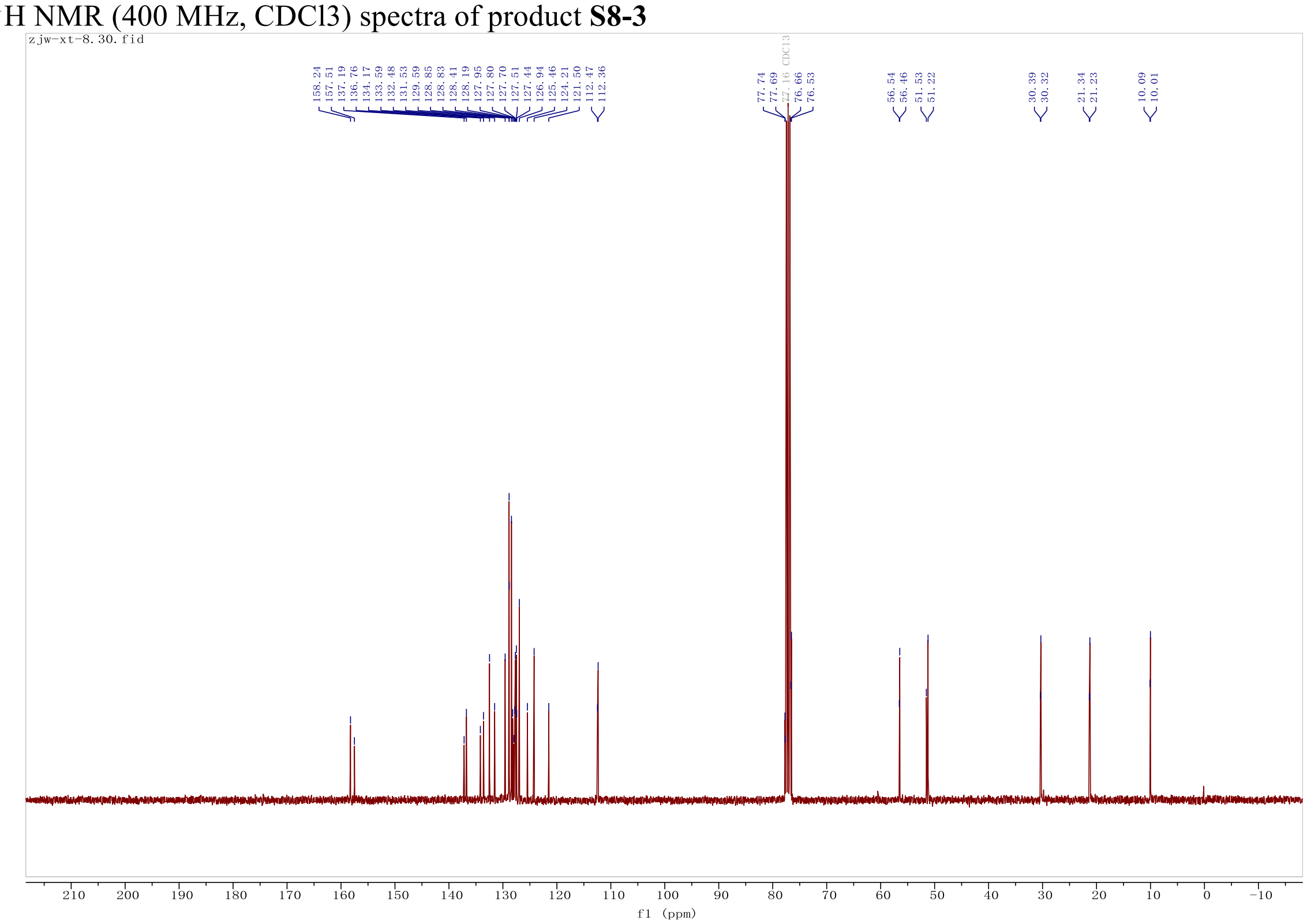

Fig. N100 ^{13}C NMR (101 MHz, CDCl3) spectra of product **S8-3**

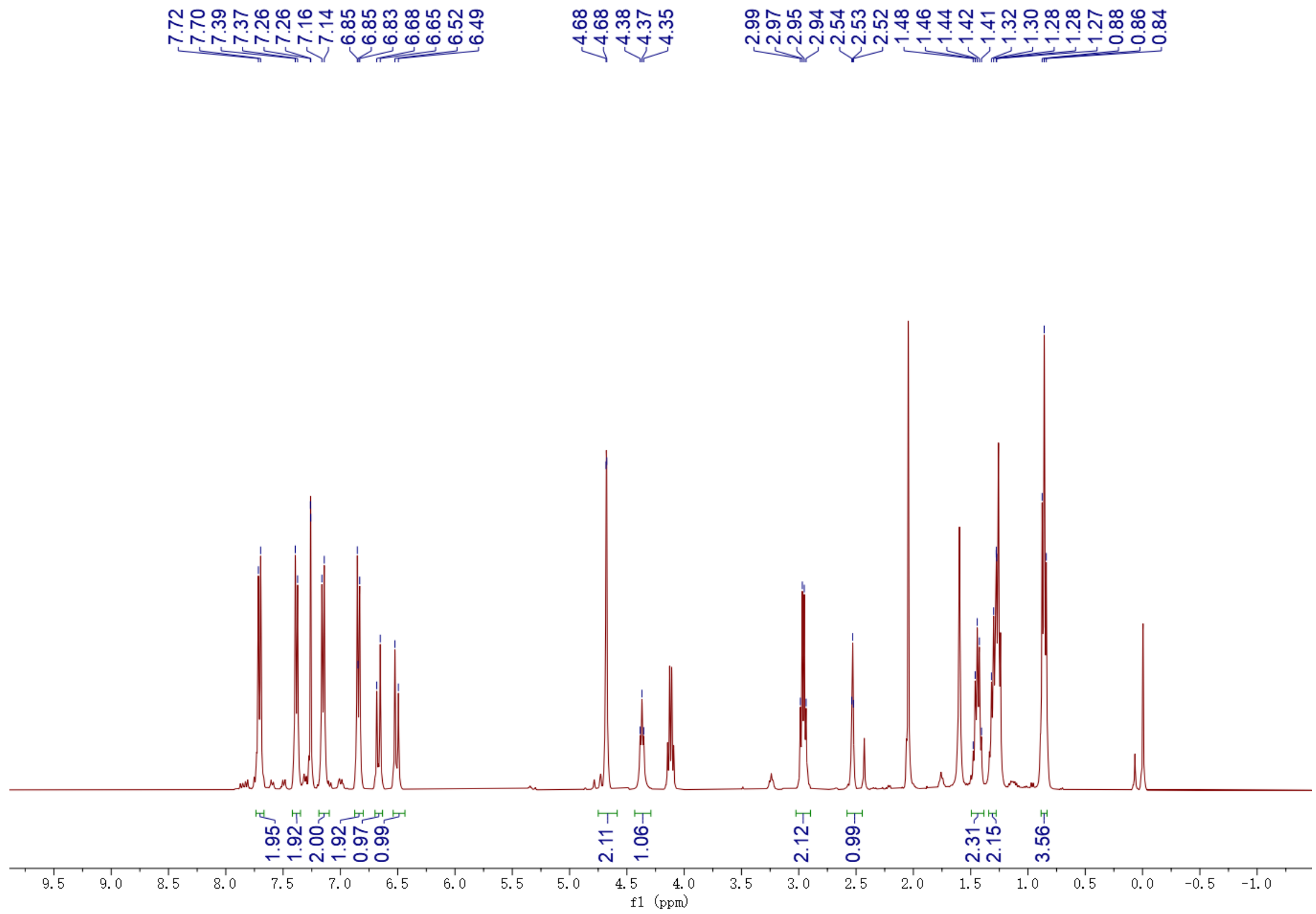

Fig. N101 ^{1}H NMR (400 MHz, CDCl3) spectra of product **S8-4**

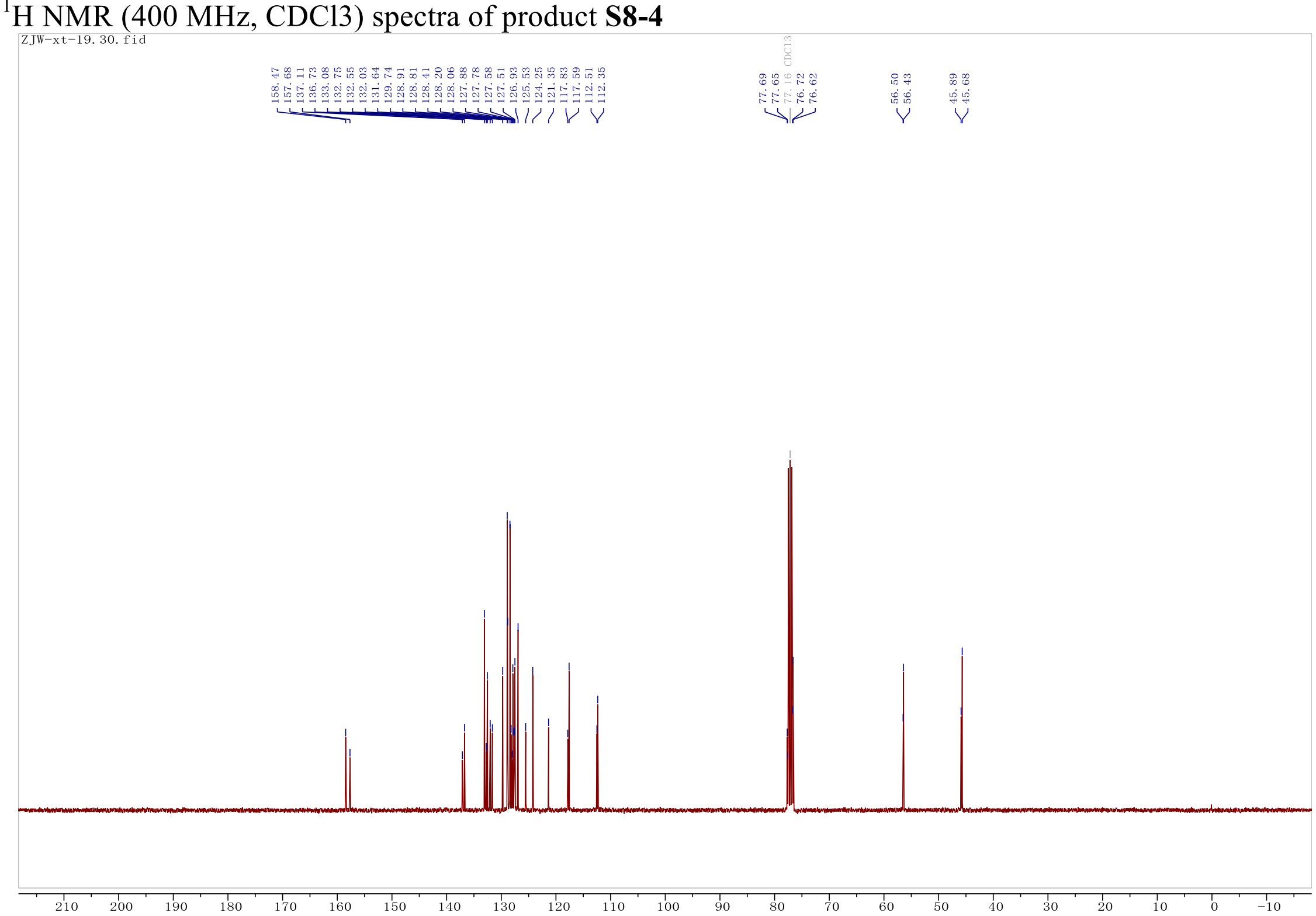

Fig. N102 ^{13}C NMR (101 MHz, CDCl3) spectra of product **S8-4**

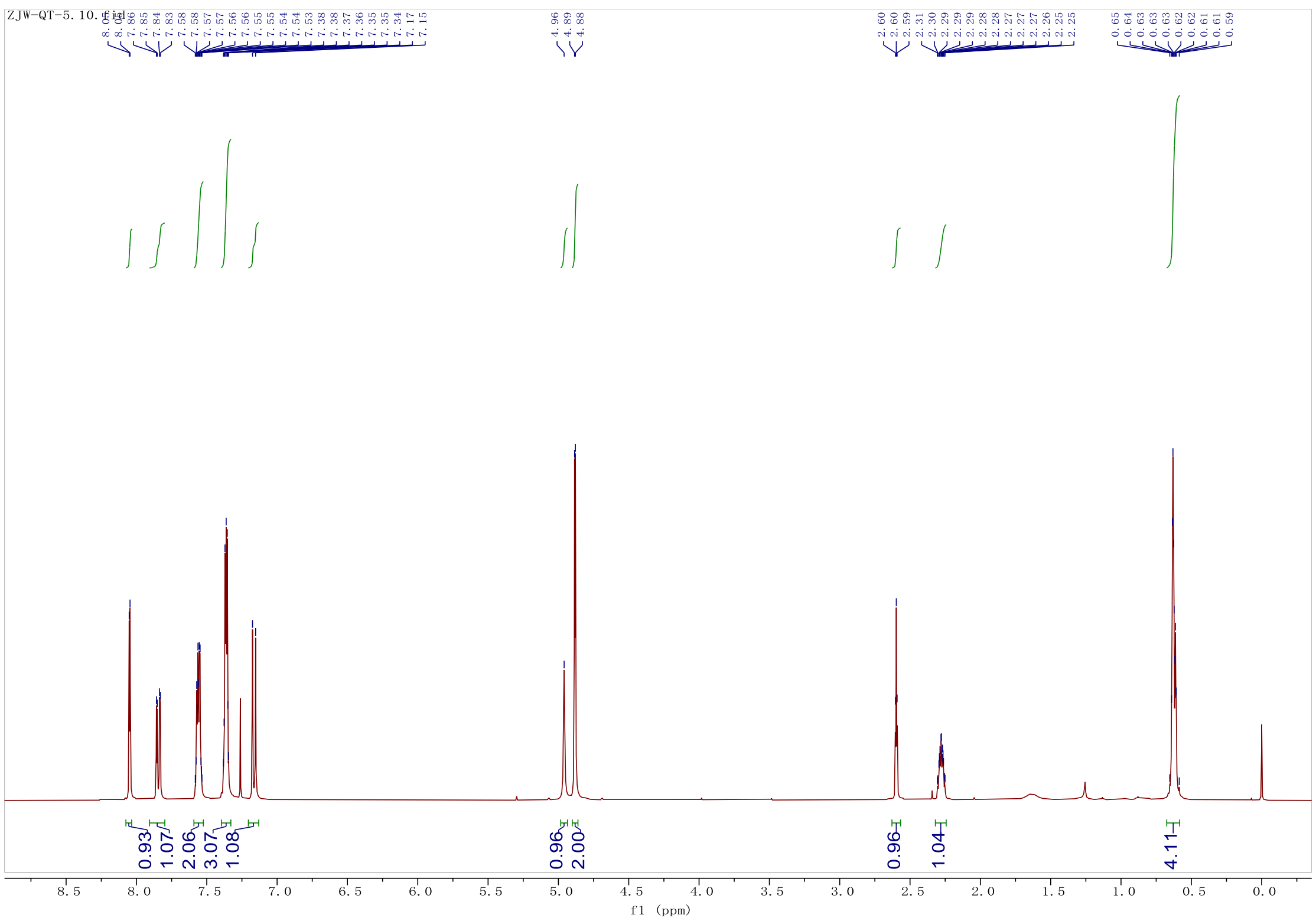

Fig. N103 ^{1}H NMR (400 MHz, CDCl3) spectra of product **S10-1**

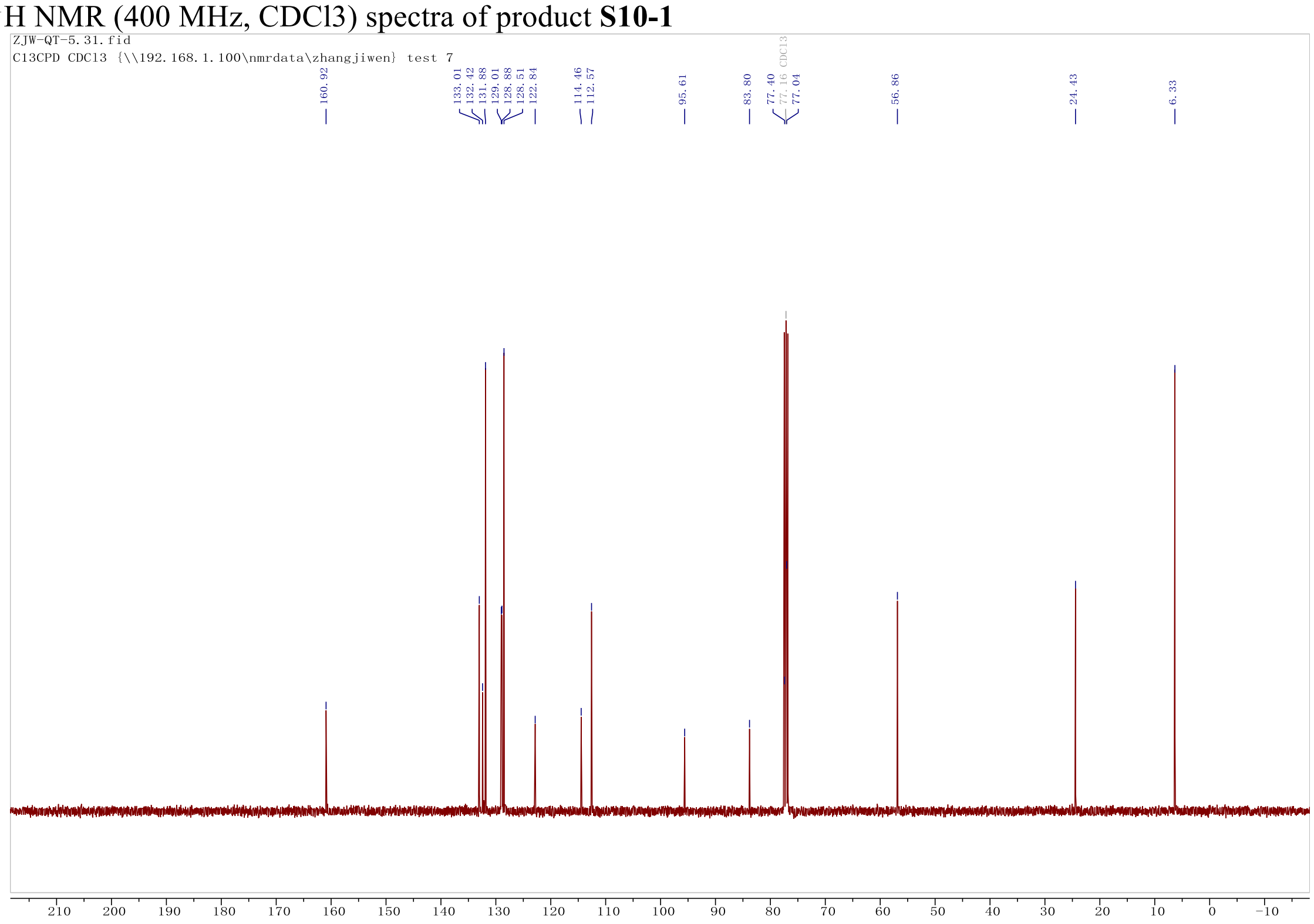

Fig. N104 ^{13}C NMR (101 MHz, CDCl3) spectra of product **S10-1**

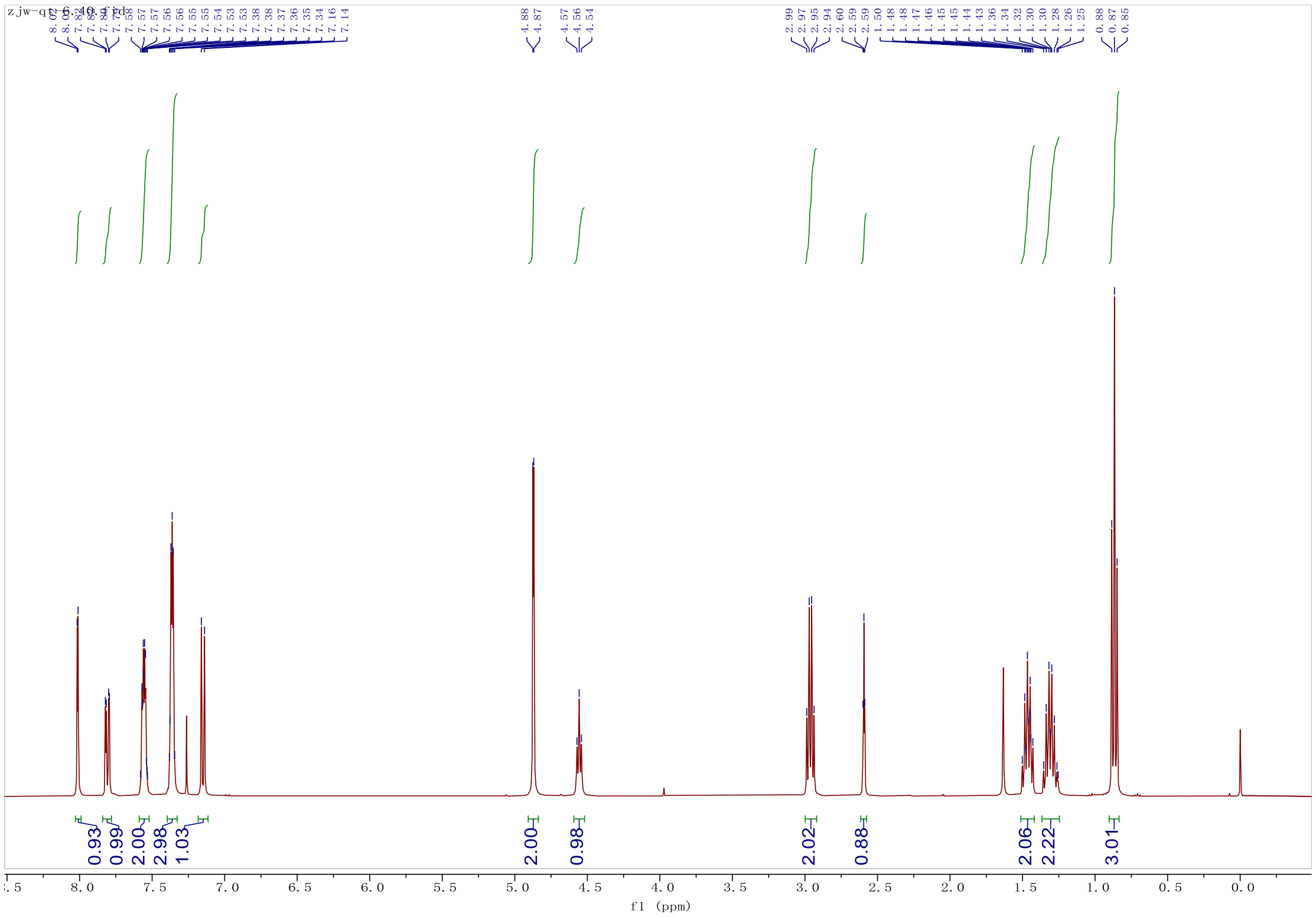

Fig. N105 ^{1}H NMR (400 MHz, CDCl3) spectra of product **S10-2**

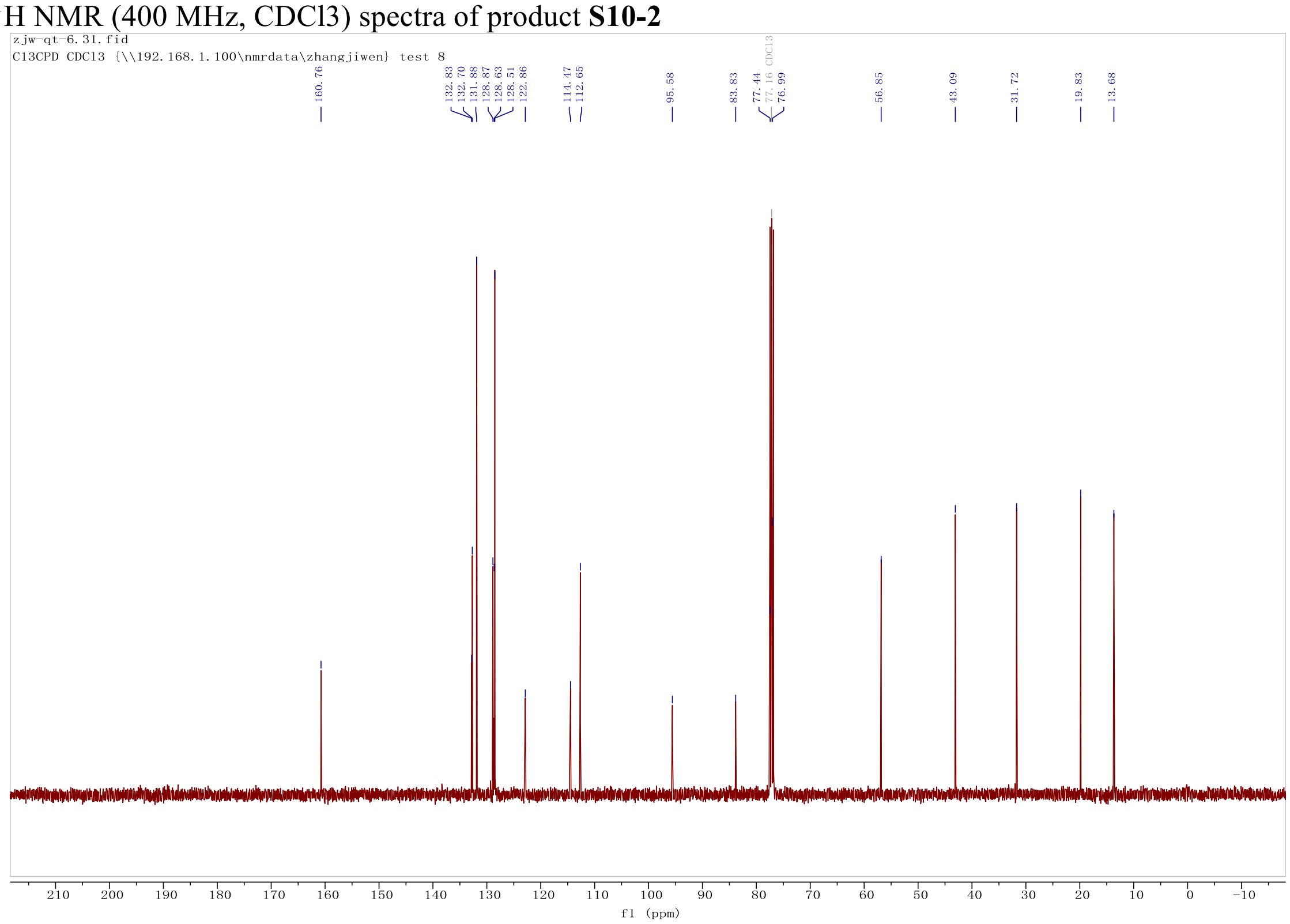

Fig. N106 ^{13}C NMR (101 MHz, CDCl3) spectra of product **S10-2**

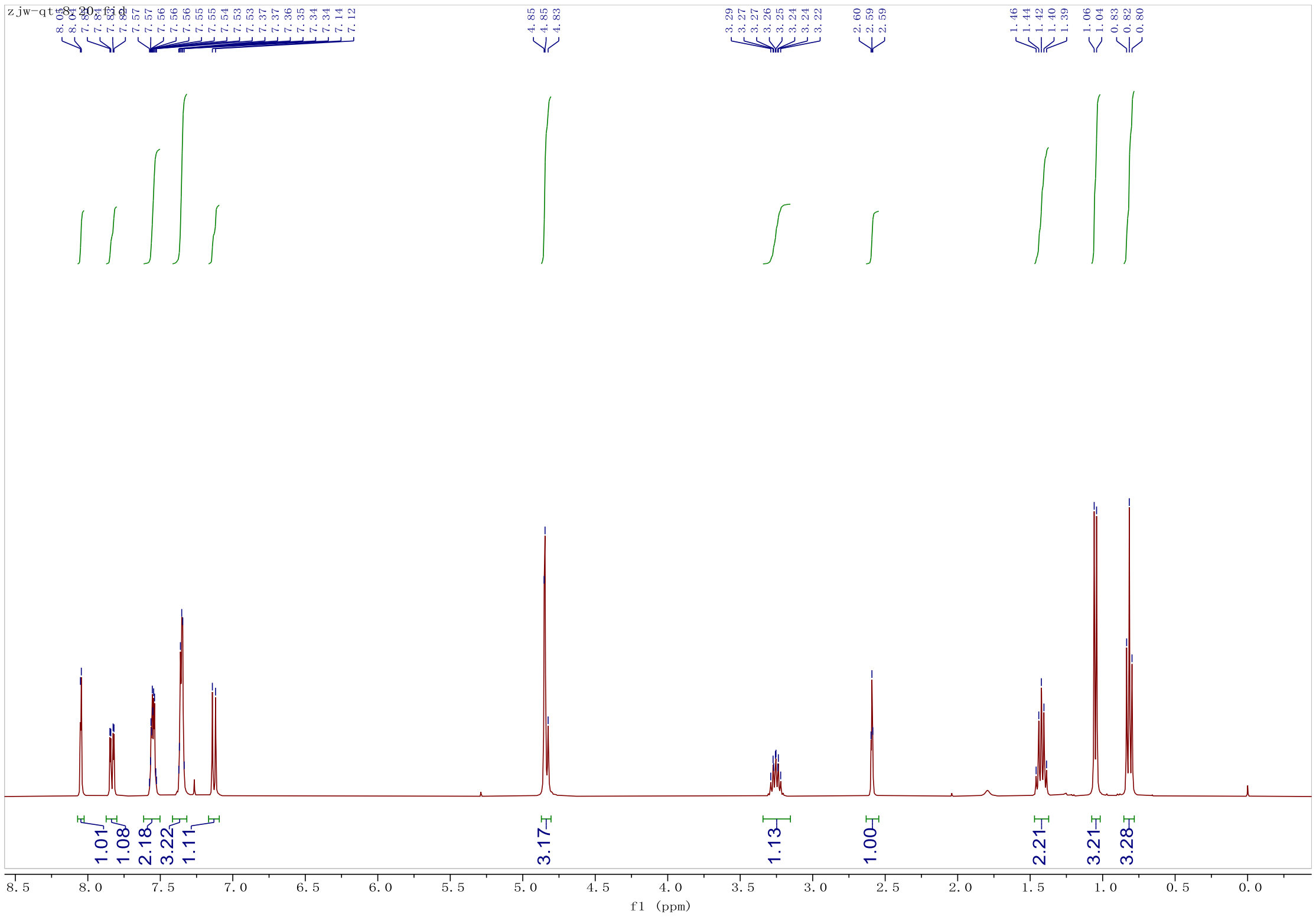

Fig. N107 ^{1}H NMR (400 MHz, CDCl3) spectra of product **S10-3**

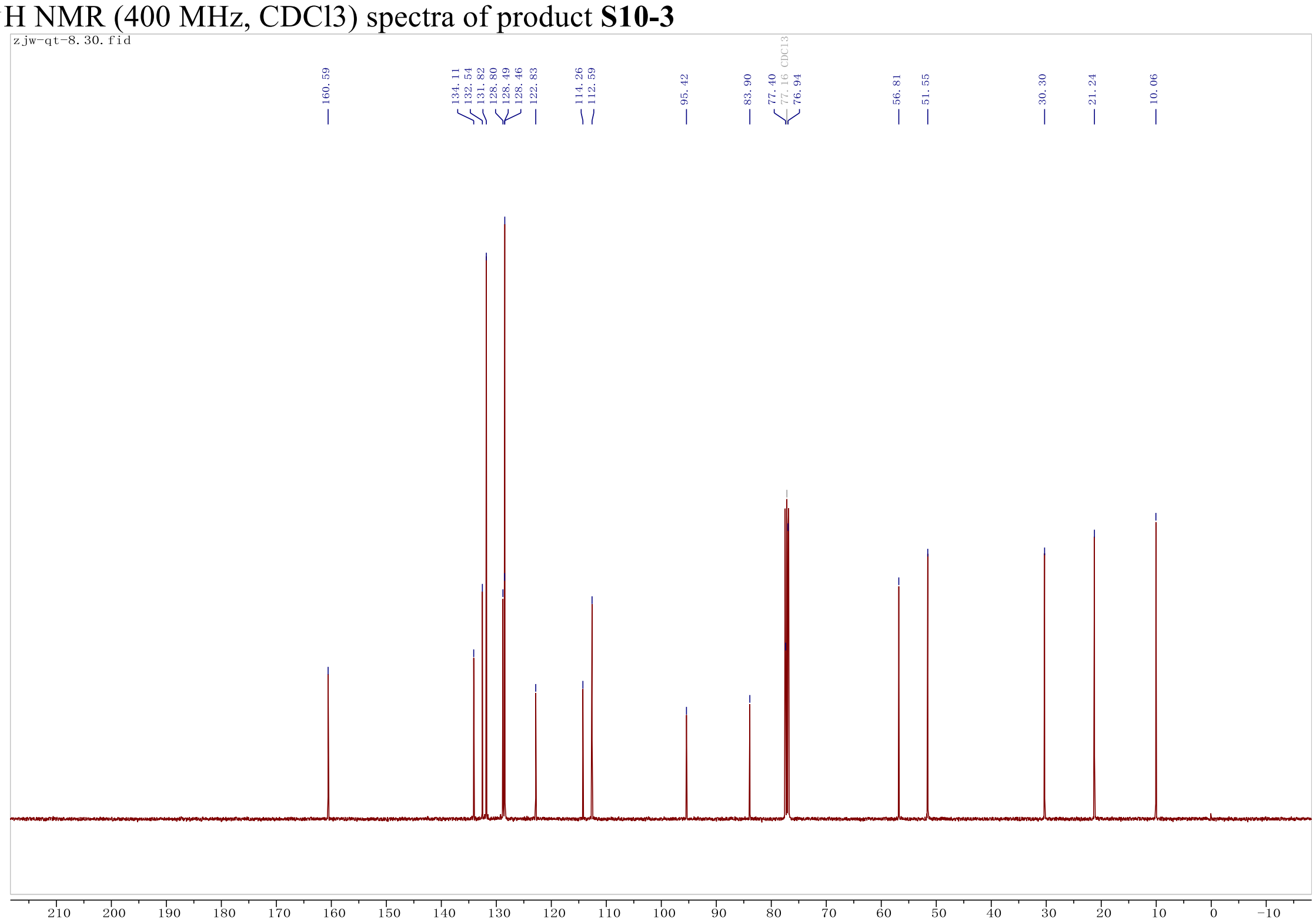

Fig. N108 ^{13}C NMR (101 MHz, CDCl3) spectra of product **S10-3**

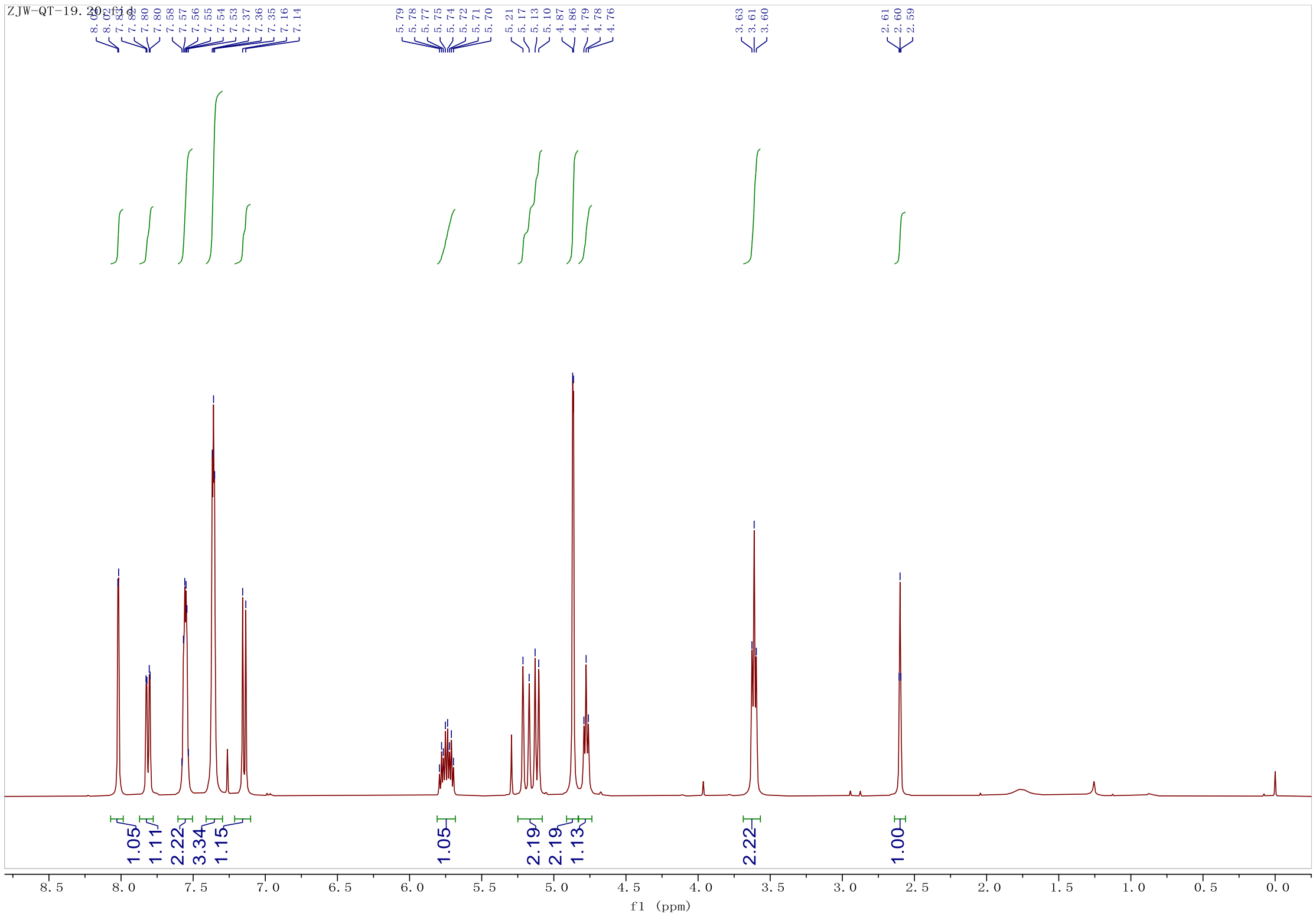

Fig. N109 ^{1}H NMR (400 MHz, CDCl3) spectra of product **S10-4**

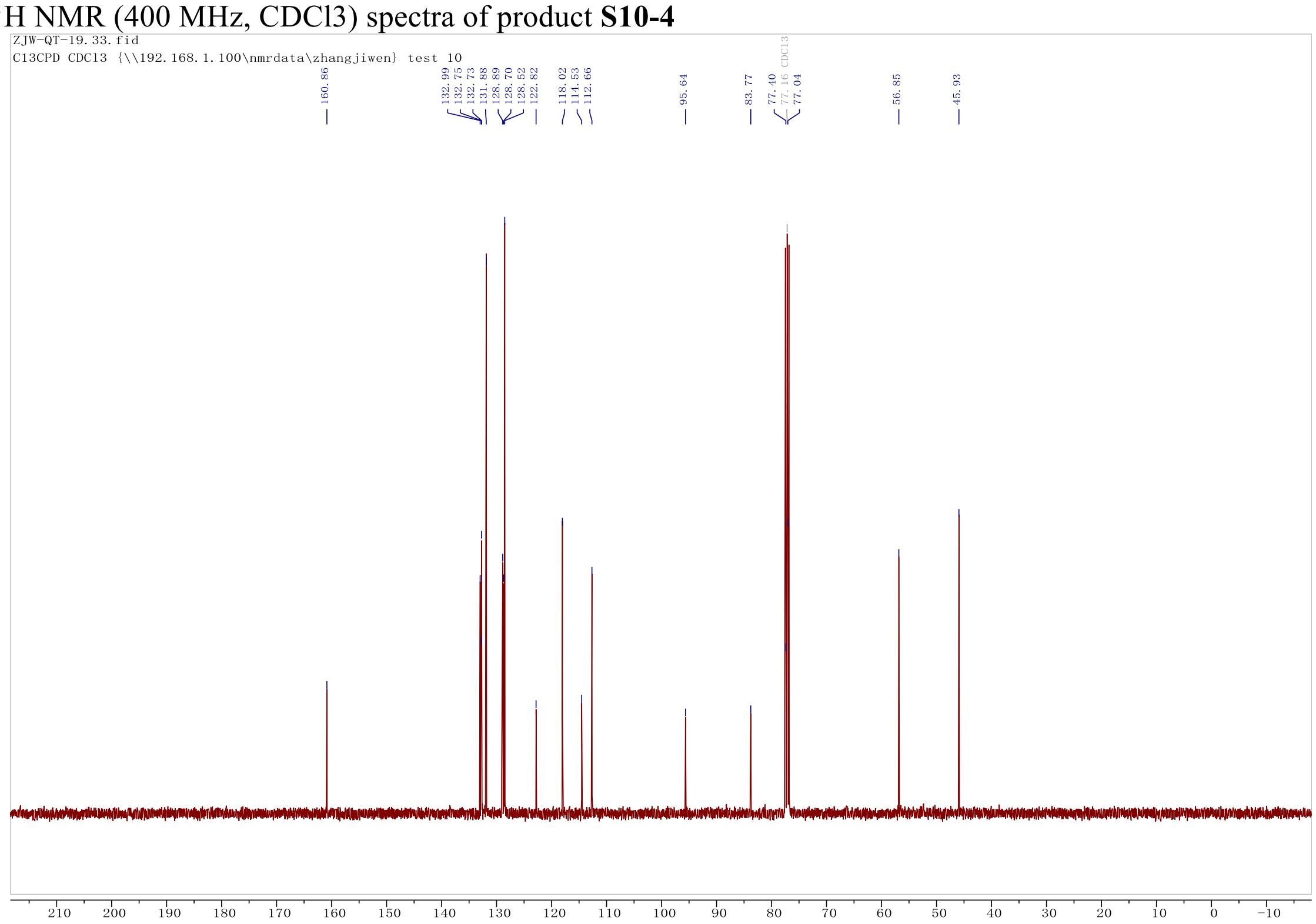

Fig. N110 ^{13}C NMR (101 MHz, CDCl3) spectra of product **S10-4**

3. Mass spectrum of the target product

Fig. M1 The mass spectra of target compound **4a-1**

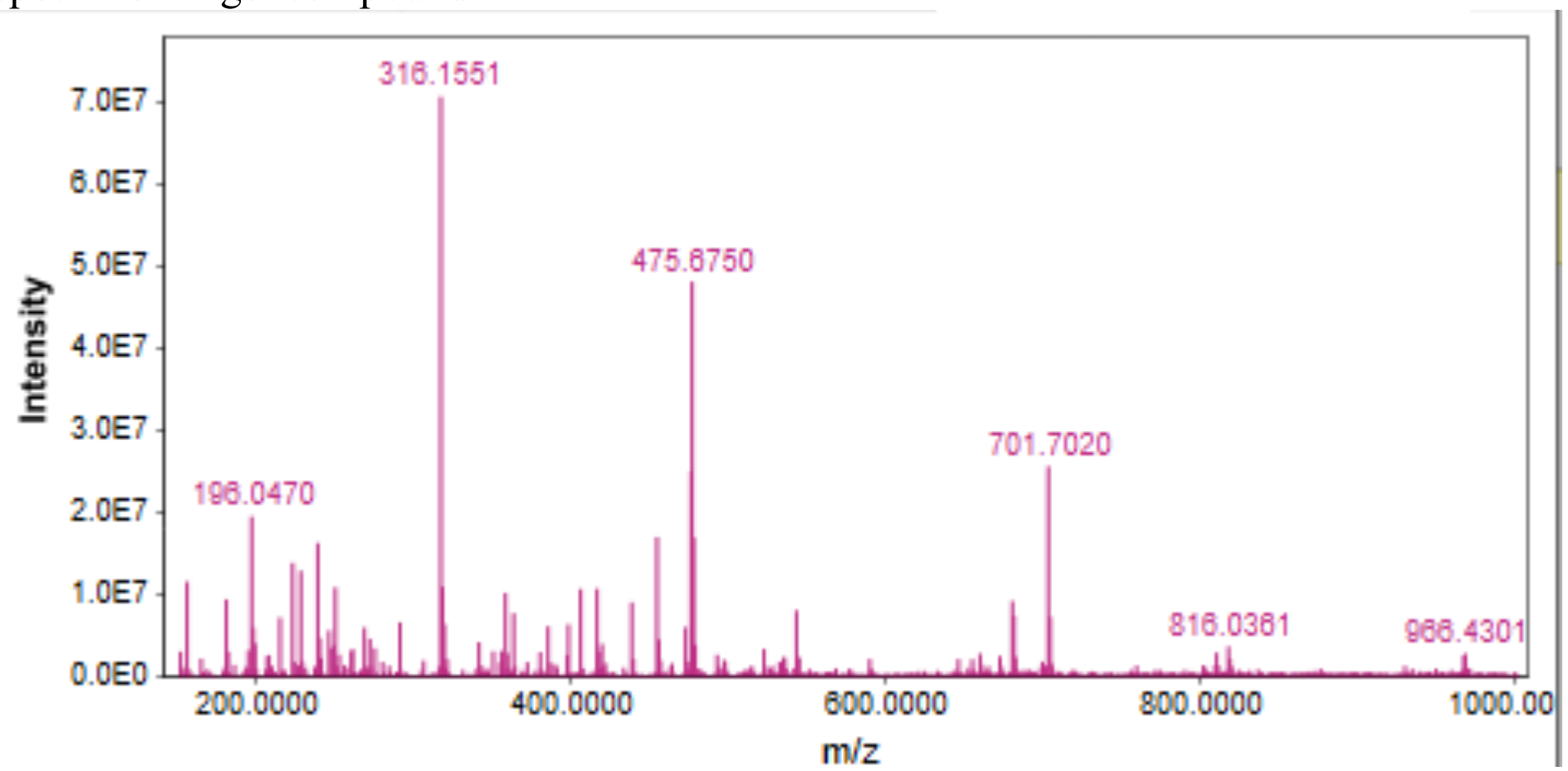

Fig. M2 The mass spectra of target compound **4a-2**

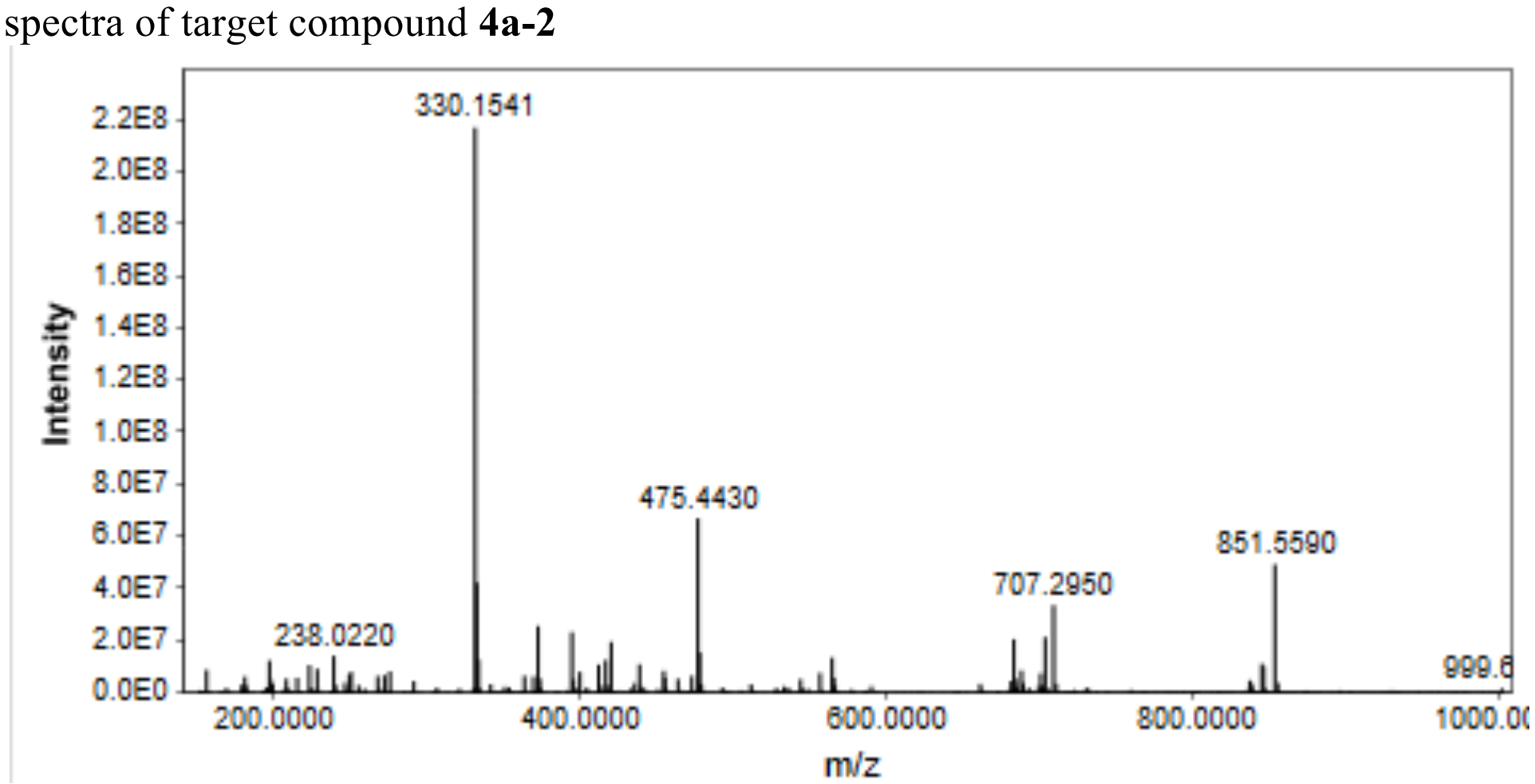

Fig. M3 The mass spectra of target compound **4a-3**

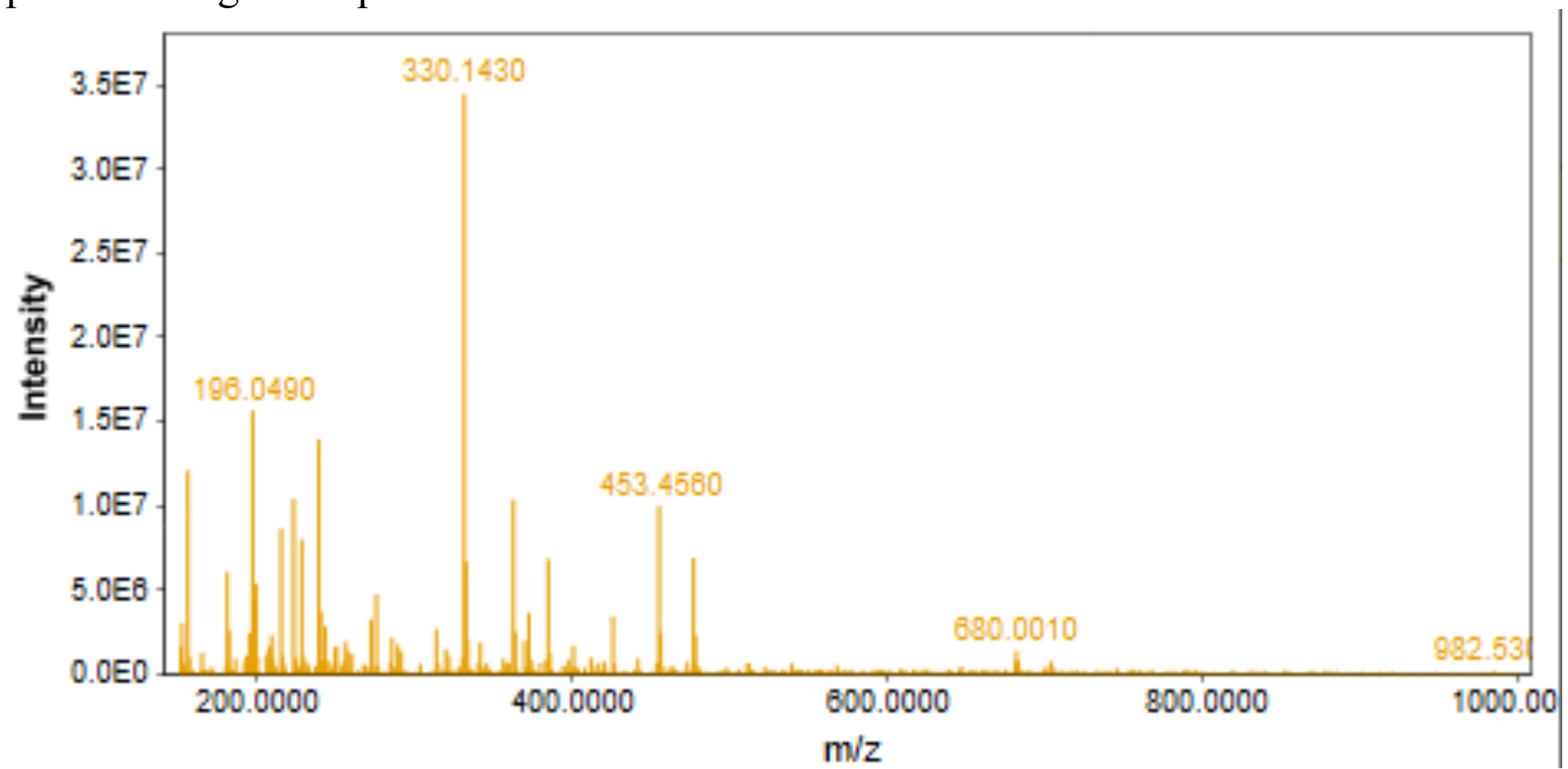

Fig. M4 The mass spectra of target compound **4a-4**

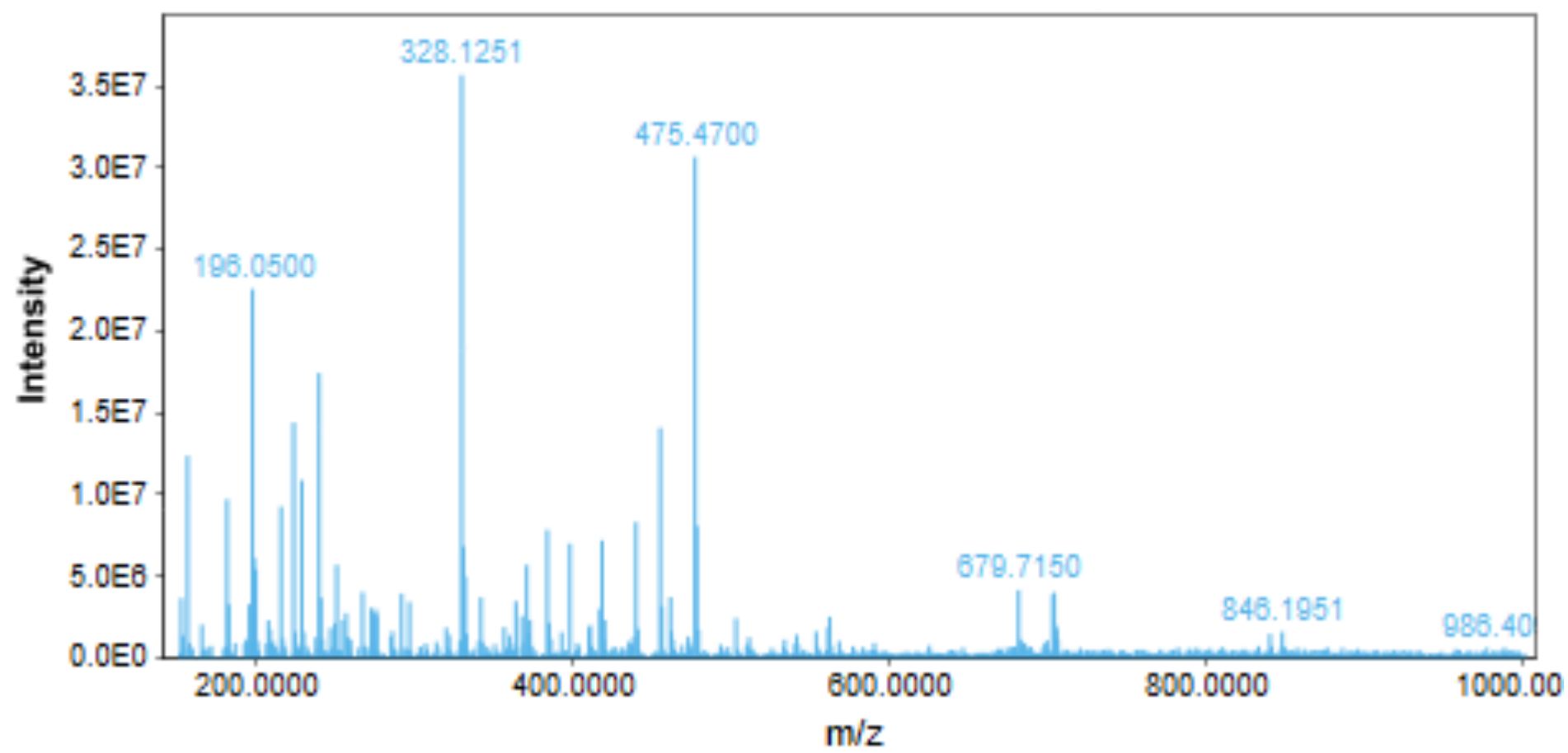

Fig. M5 The mass spectra of target compound **4a-5**

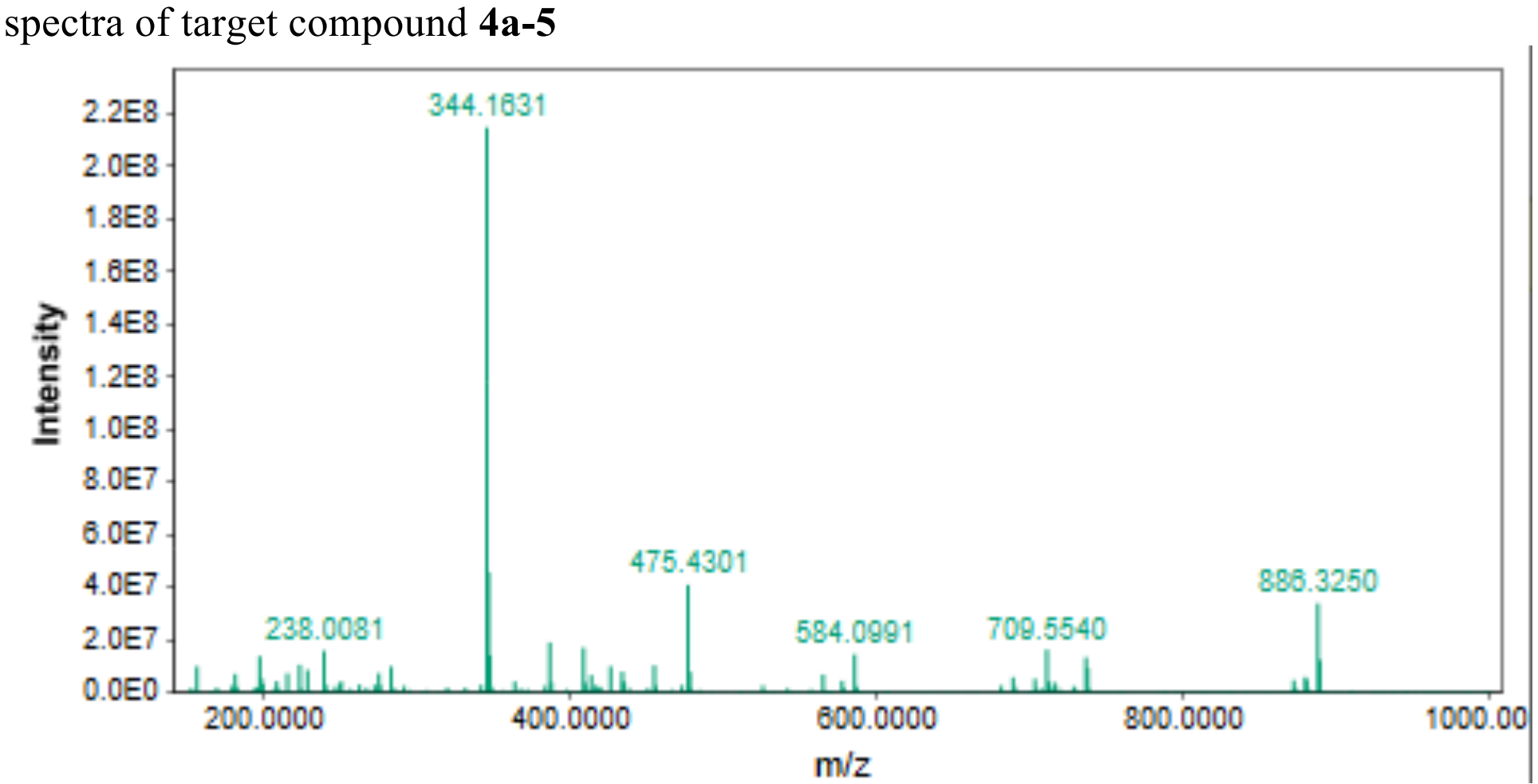

Fig. M6 The mass spectra of target compound **4a-6**

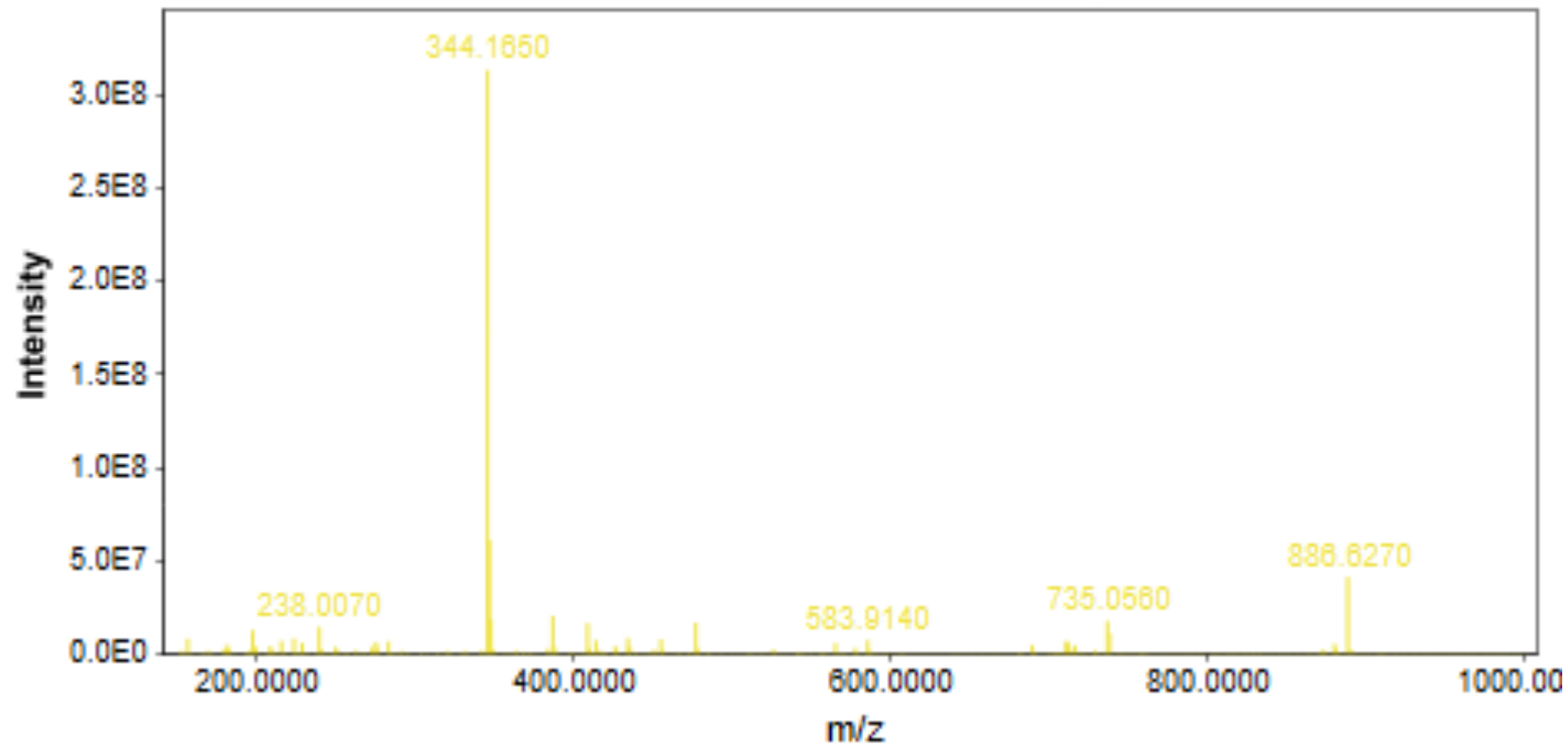

Fig. M7 The mass spectra of target compound **4a-7**

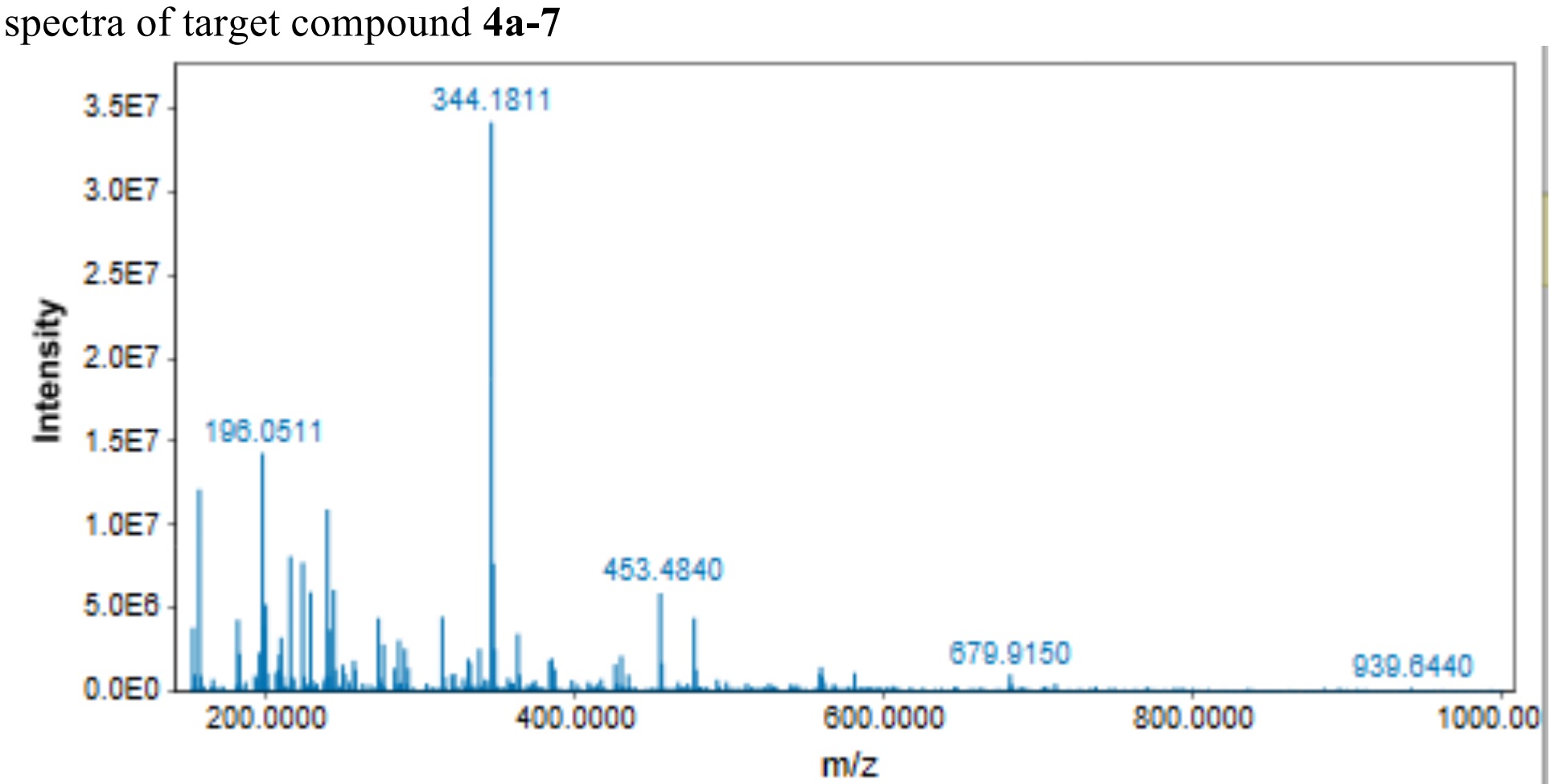

Fig. M8 The mass spectra of target compound **4a-8**

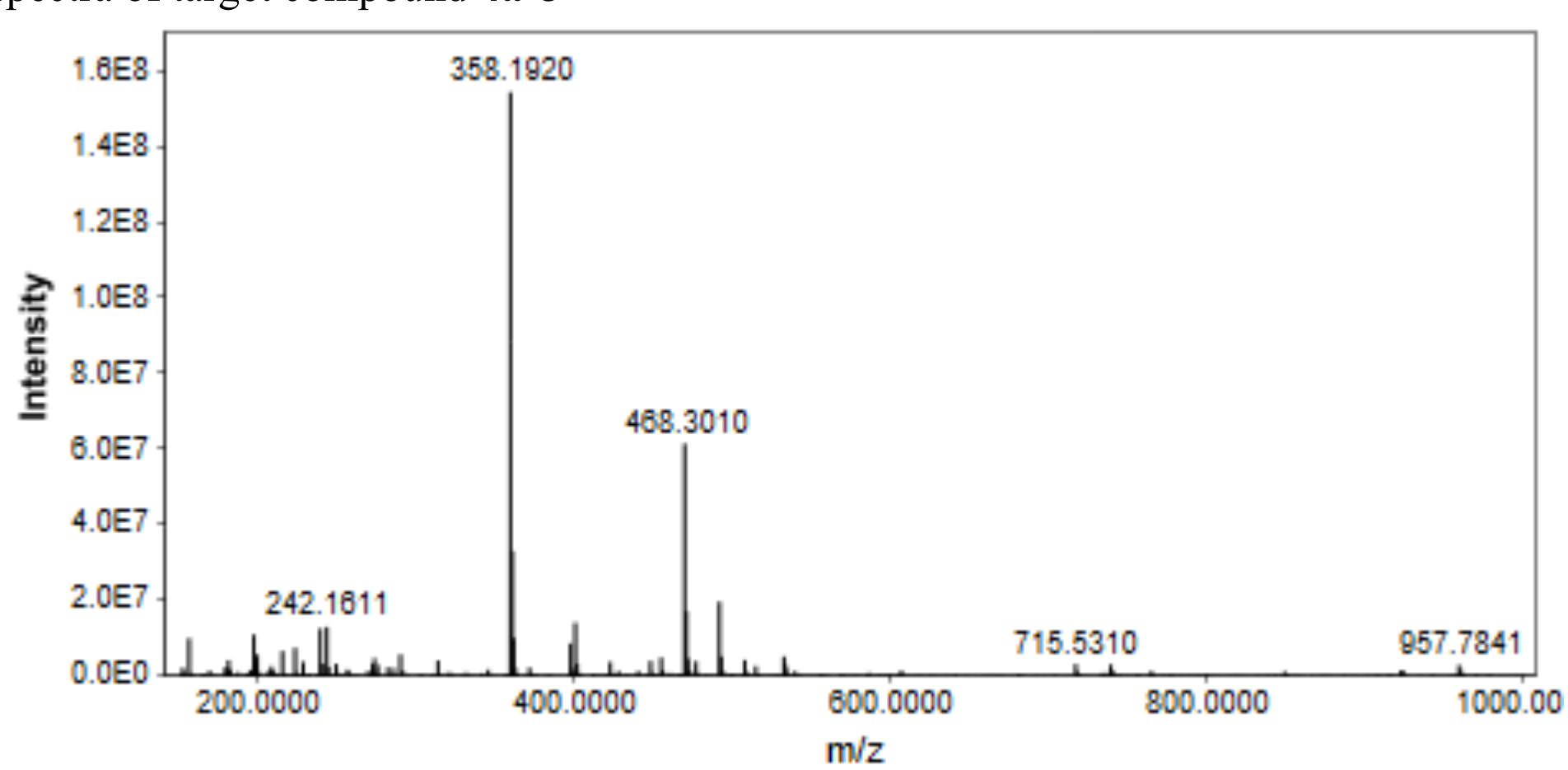

Fig. M9 The mass spectra of target compound **4a-9**

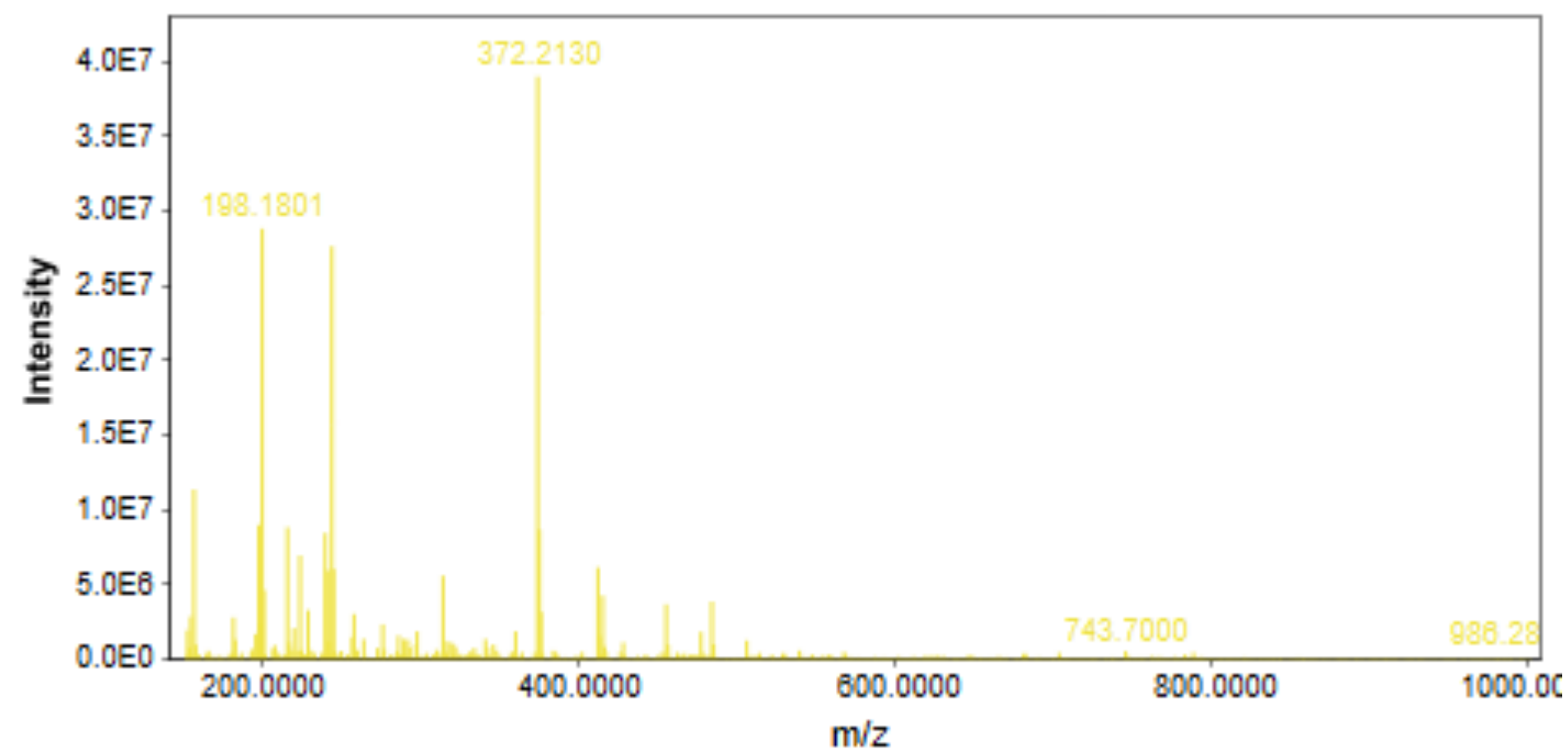

Fig. M10 The mass spectra of target compound **4a-10**

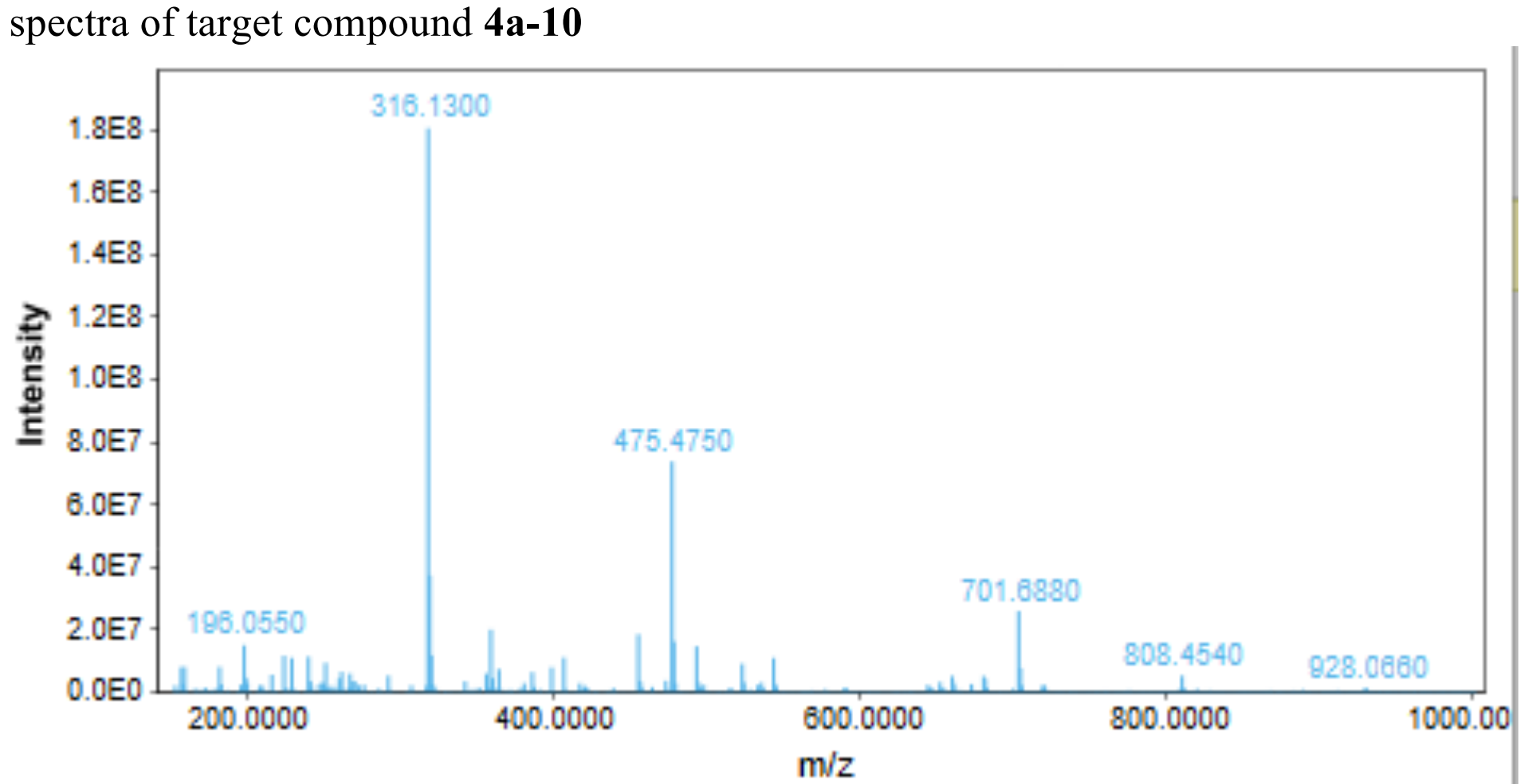

Fig. M11 The mass spectra of target compound **4a-11**

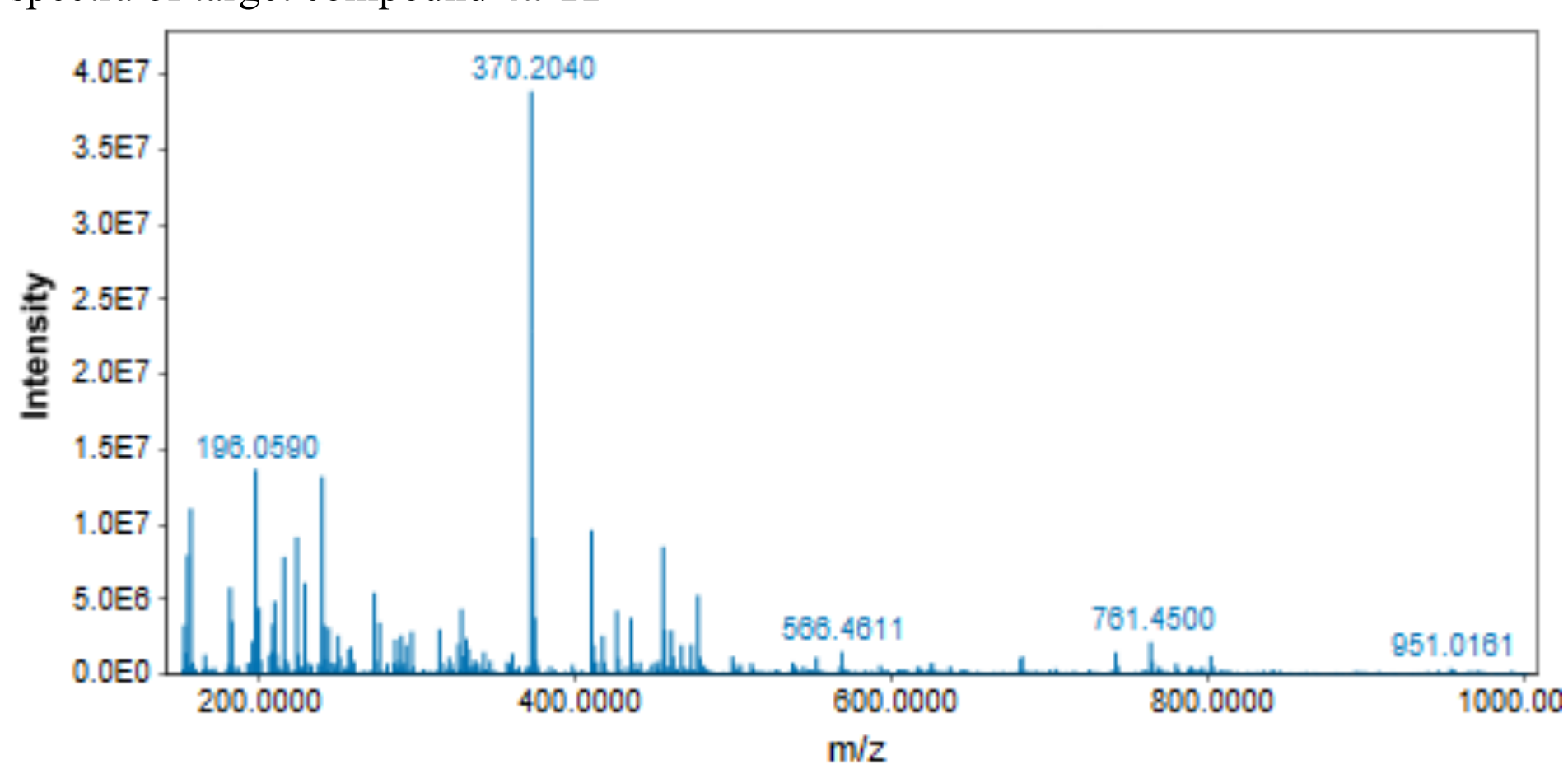

Fig. M12 The mass spectra of target compound **4a-12**

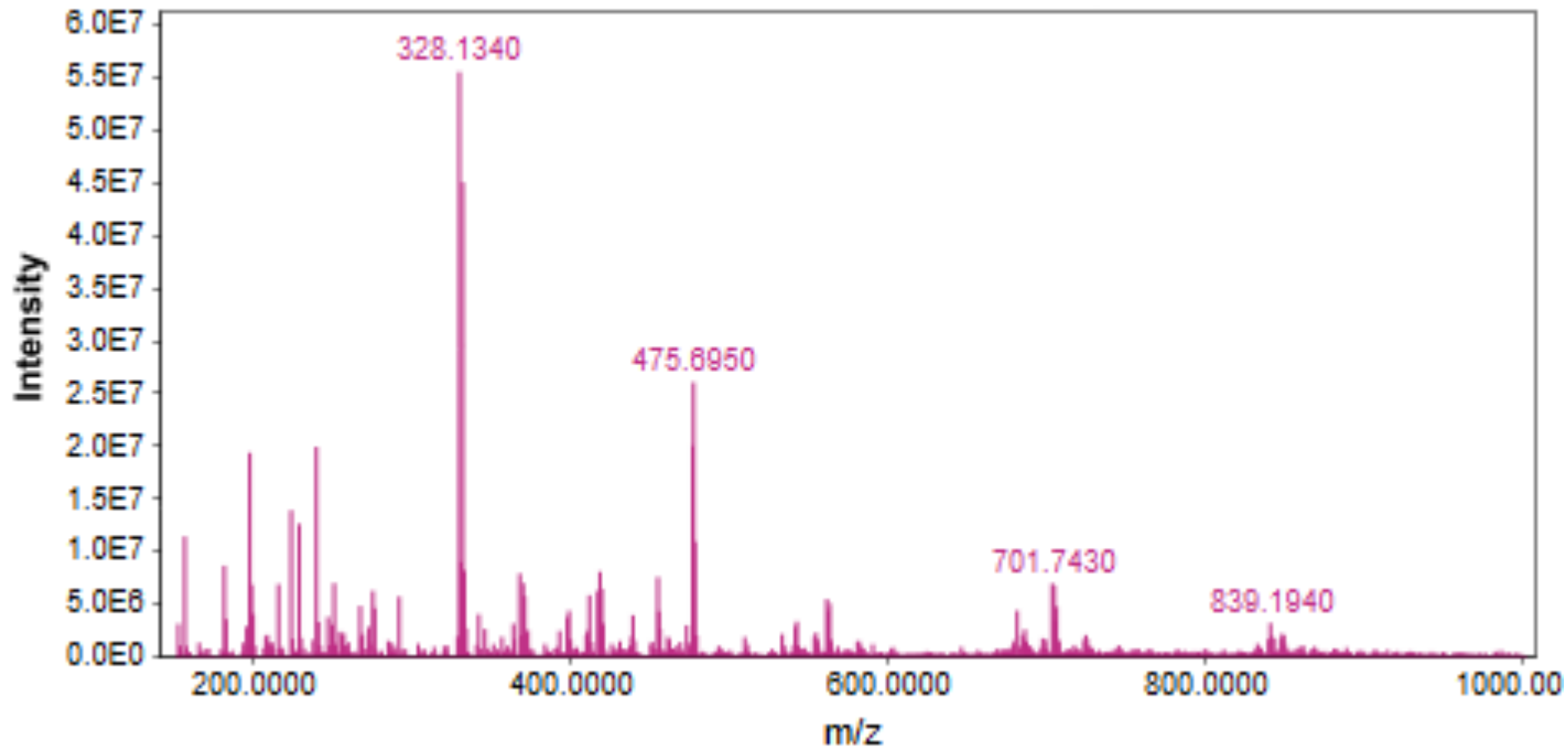

Fig. M13 The mass spectra of target compound **4b-1**

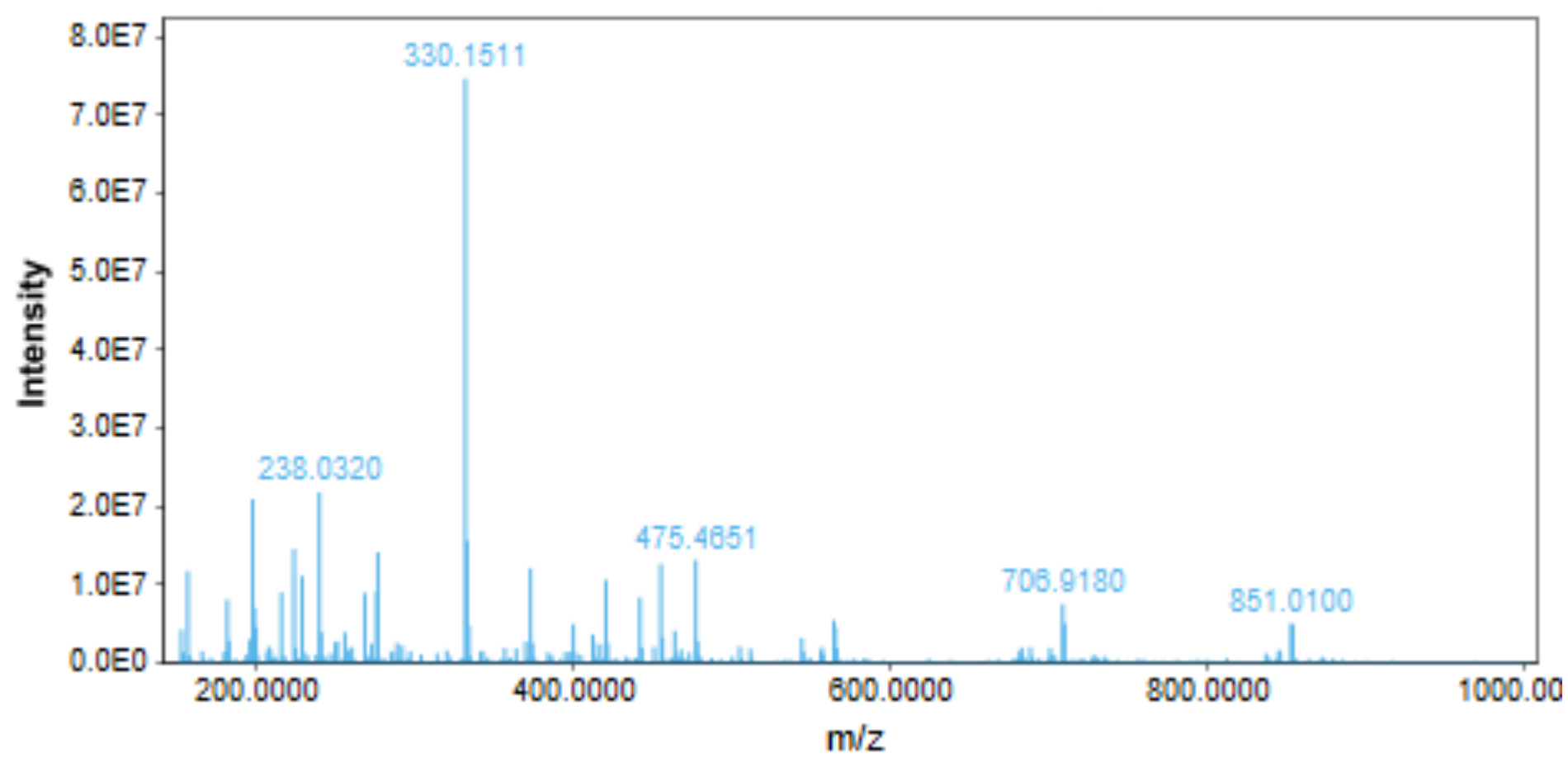

Fig. M14 The mass spectra of target compound **4b-2**

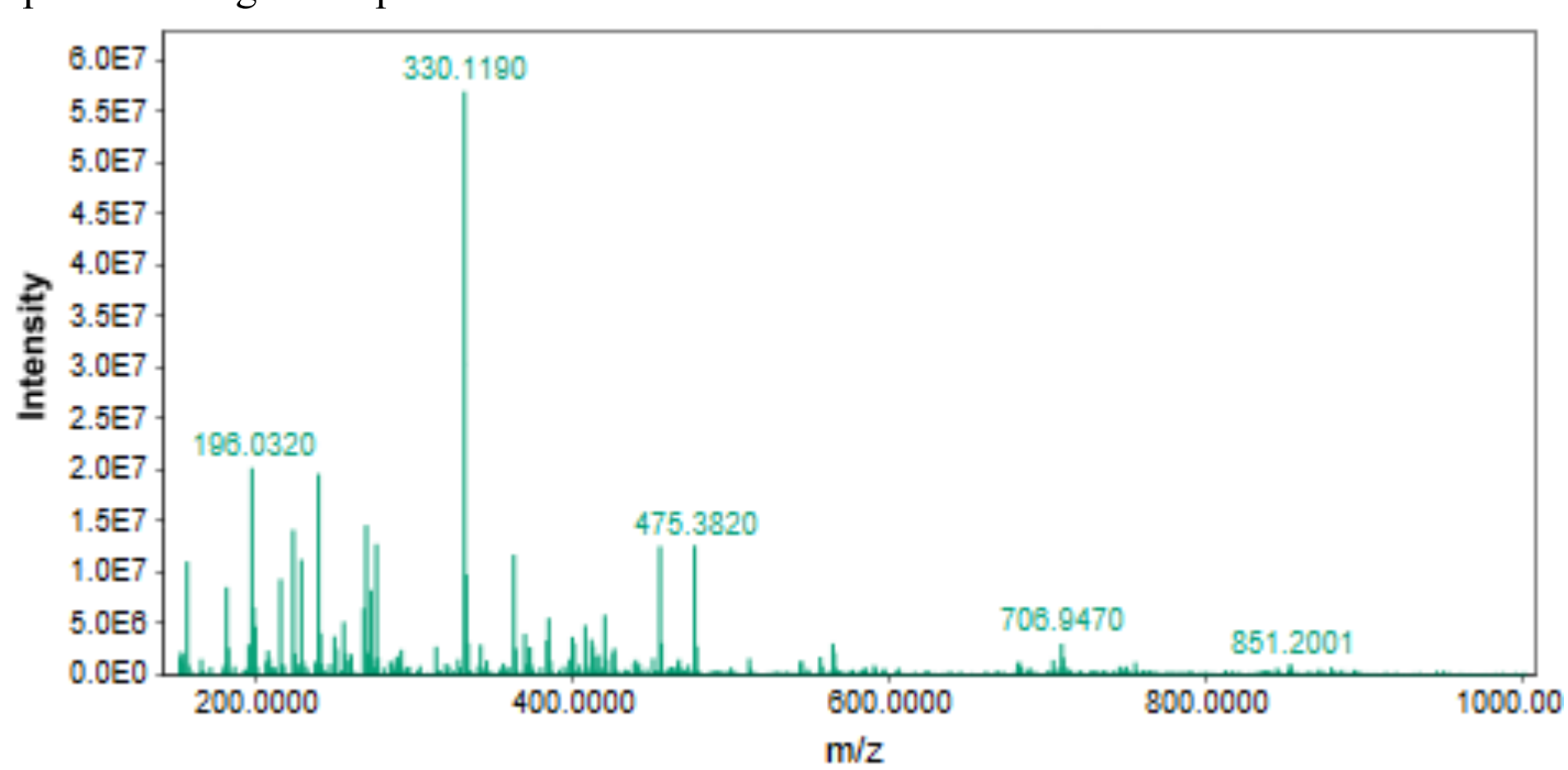

Fig. M15 The mass spectra of target compound **4b-3**

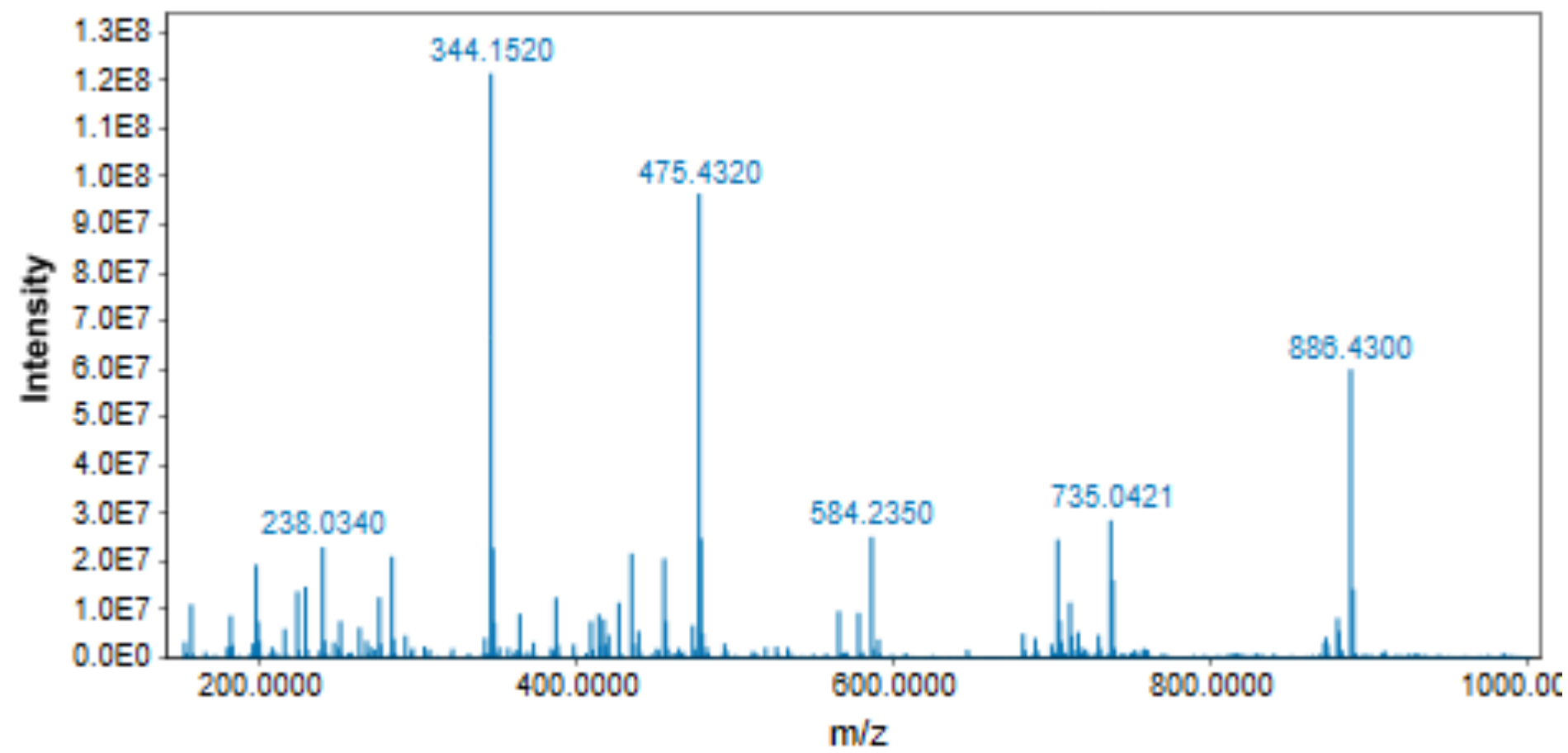

Fig. M16 The mass spectra of target compound **4b-4**

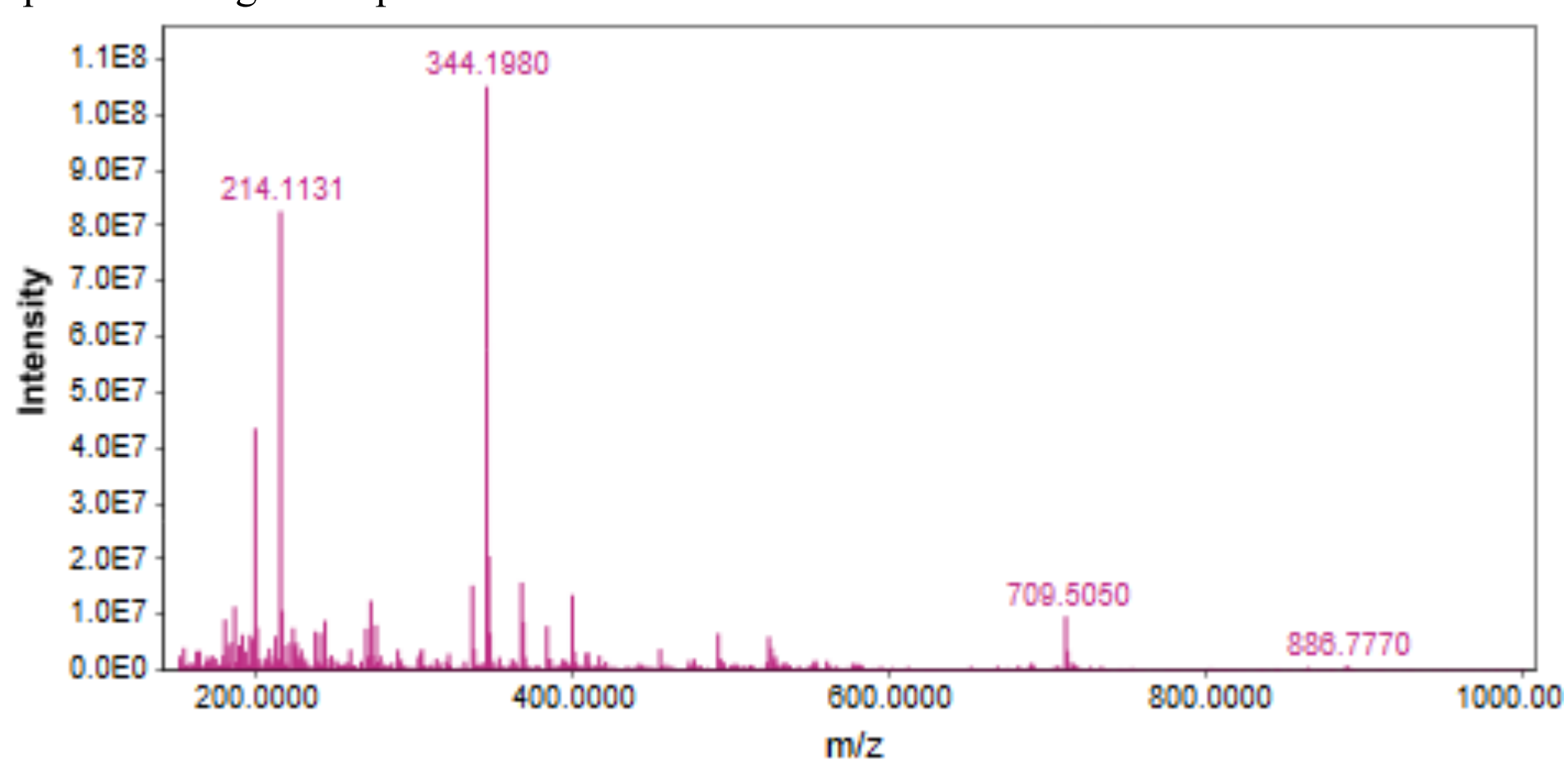

Fig. M17 The mass spectra of target compound **4b-5**

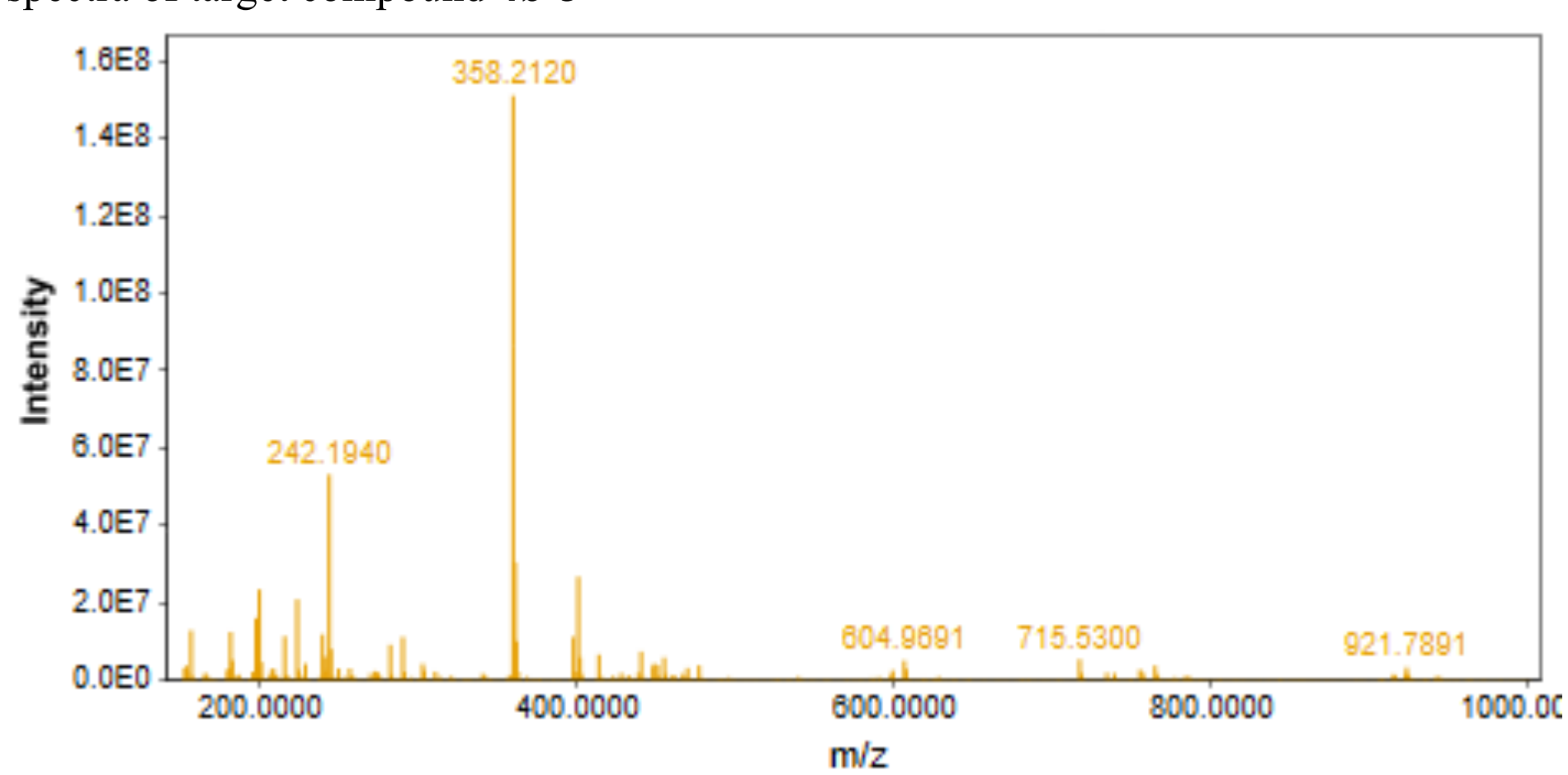

Fig. M18 The mass spectra of target compound **4b-6**

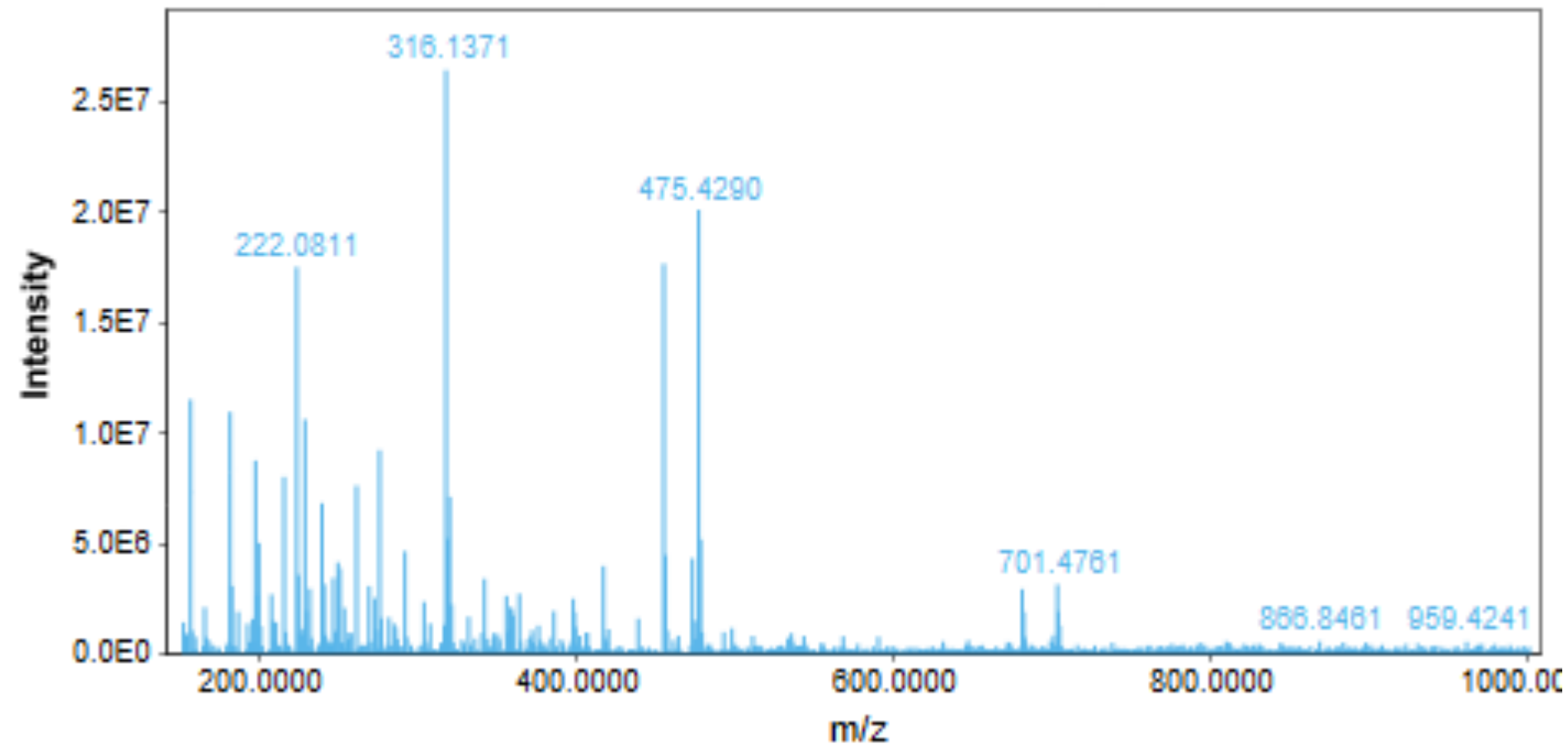

Fig. M19 The mass spectra of target compound **4b-7**

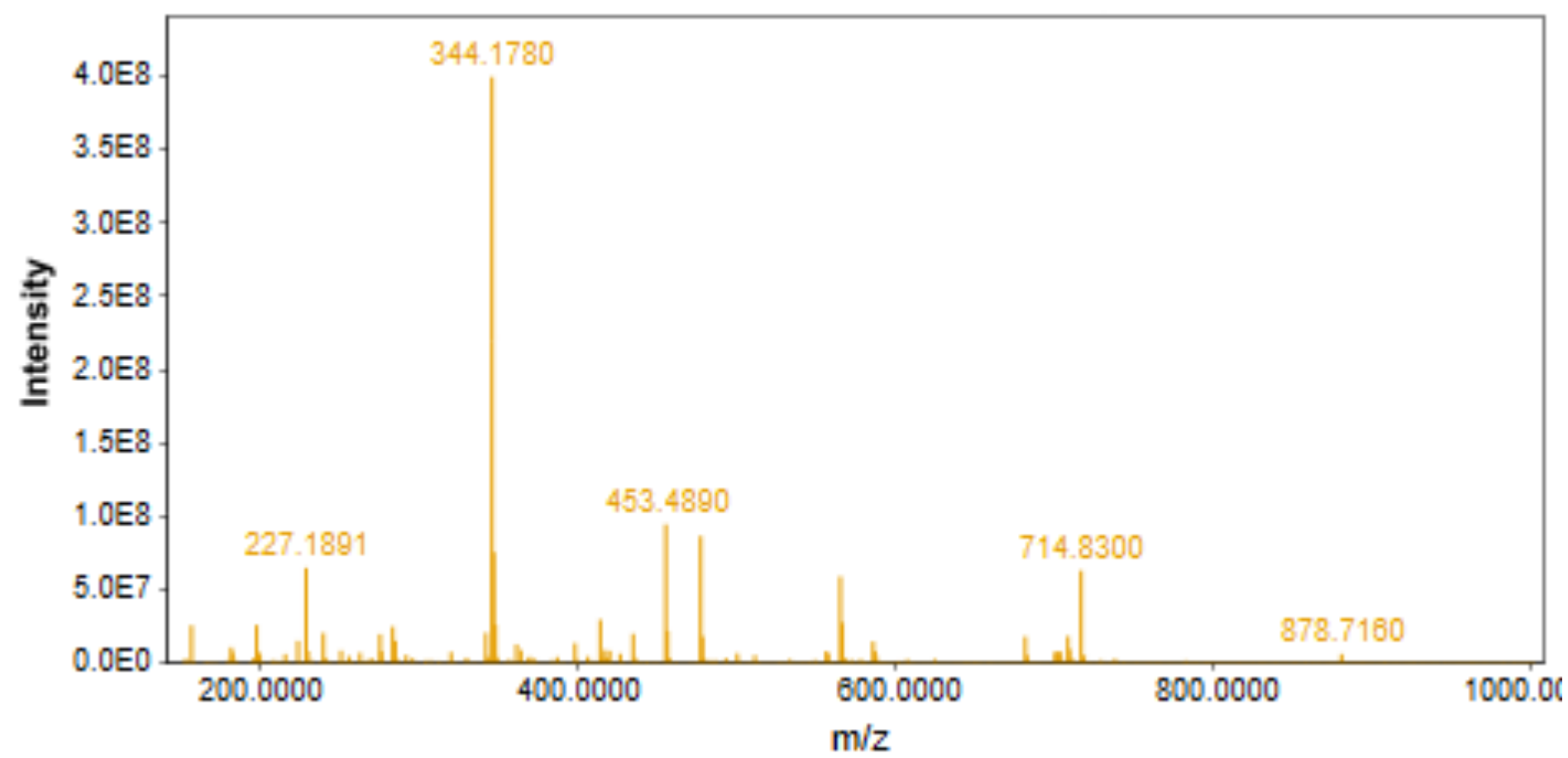

Fig. M20 The mass spectra of target compound **4c-1**

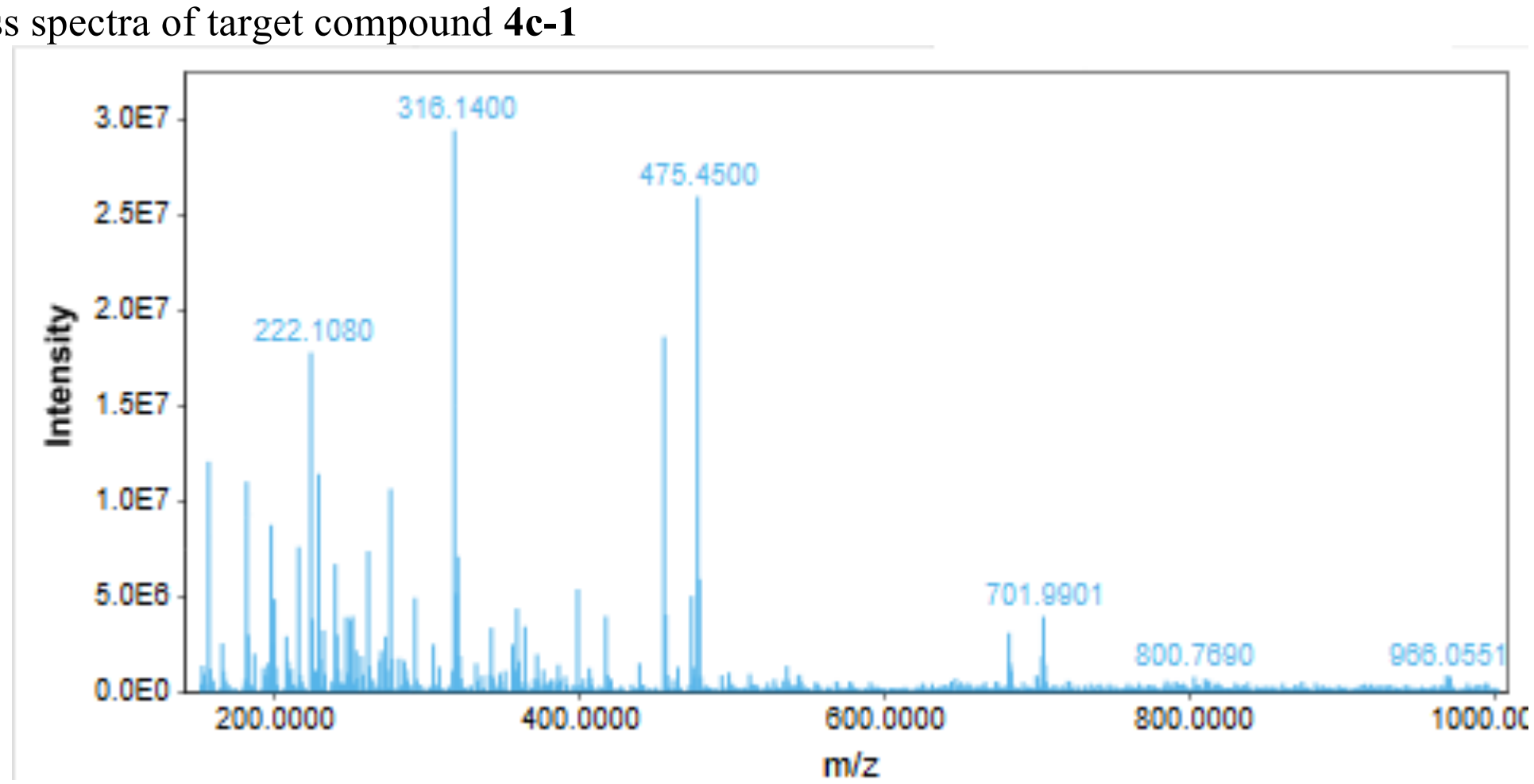

Fig. M21 The mass spectra of target compound **4c-2**

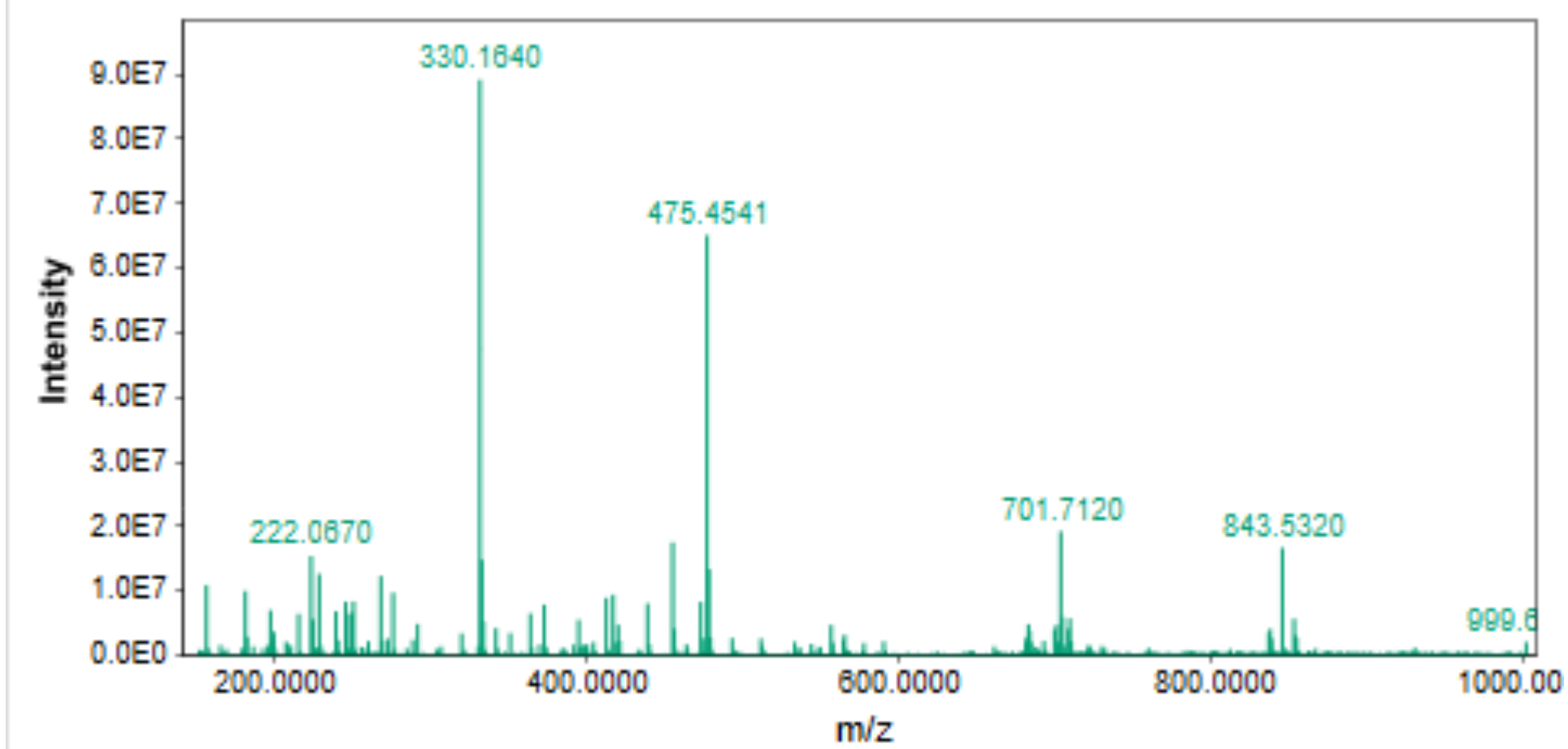

Fig. M22 The mass spectra of target compound **4c-3**

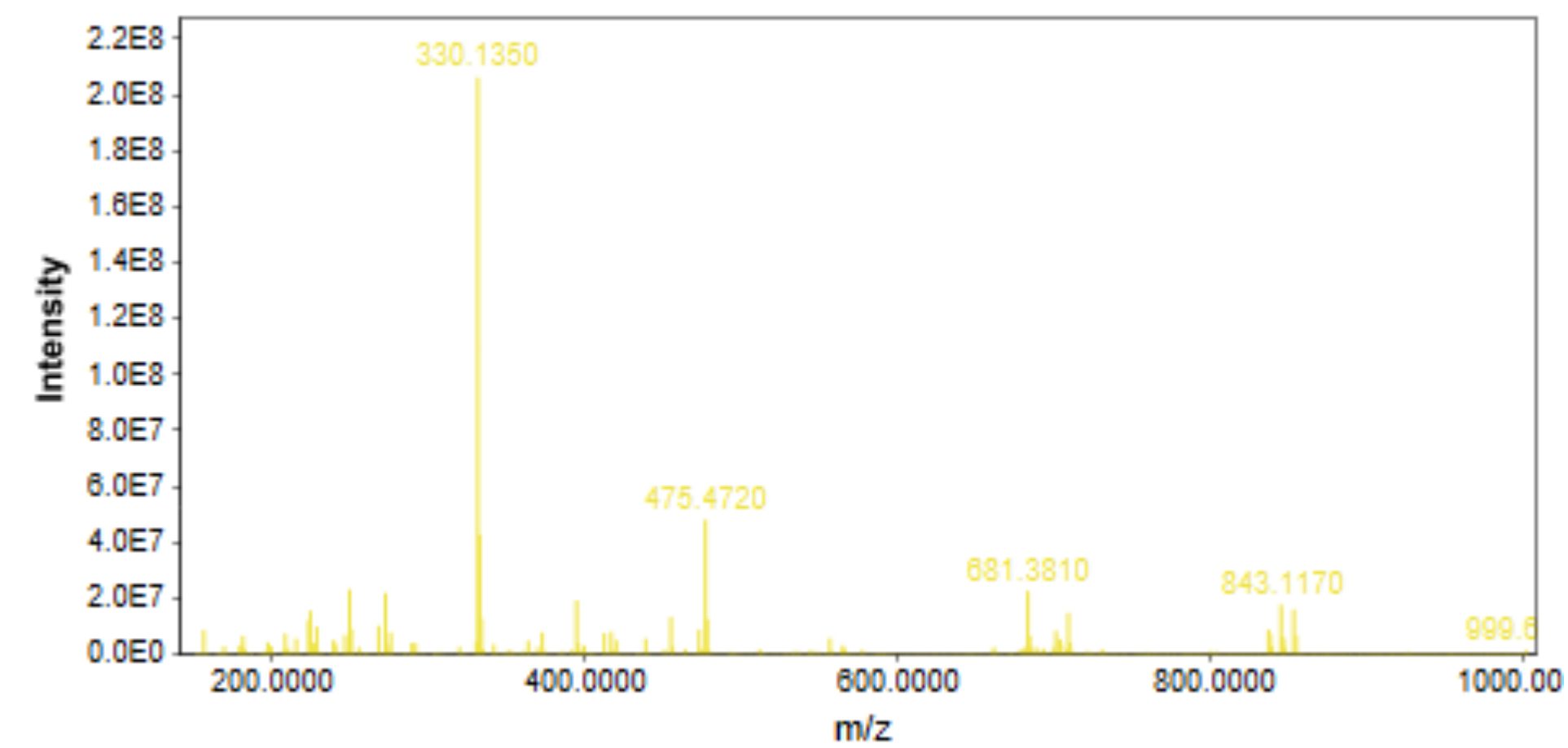

Fig. M23 The mass spectra of target compound **4c-4**

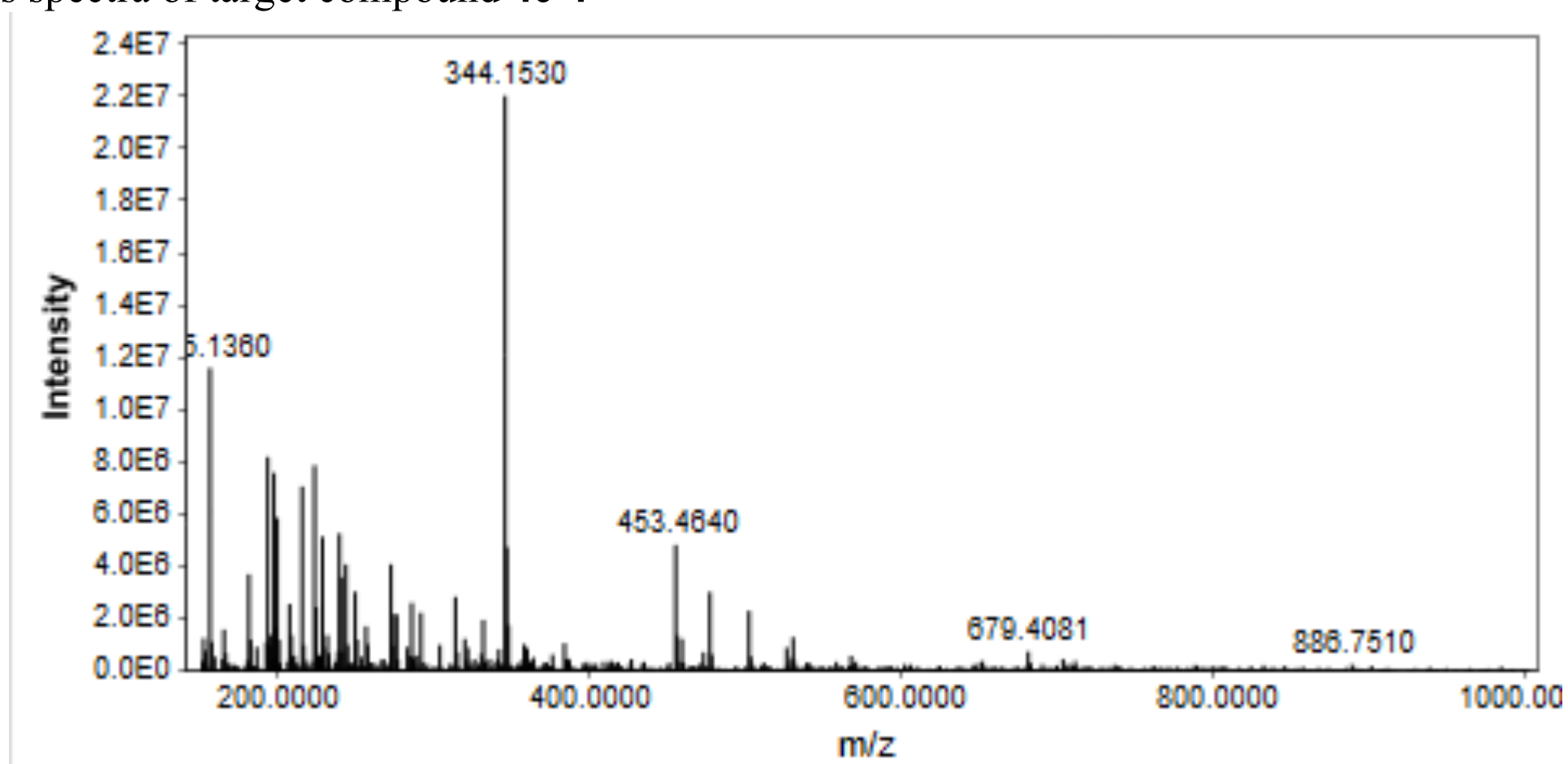

Fig. M24 The mass spectra of target compound **4c-5**

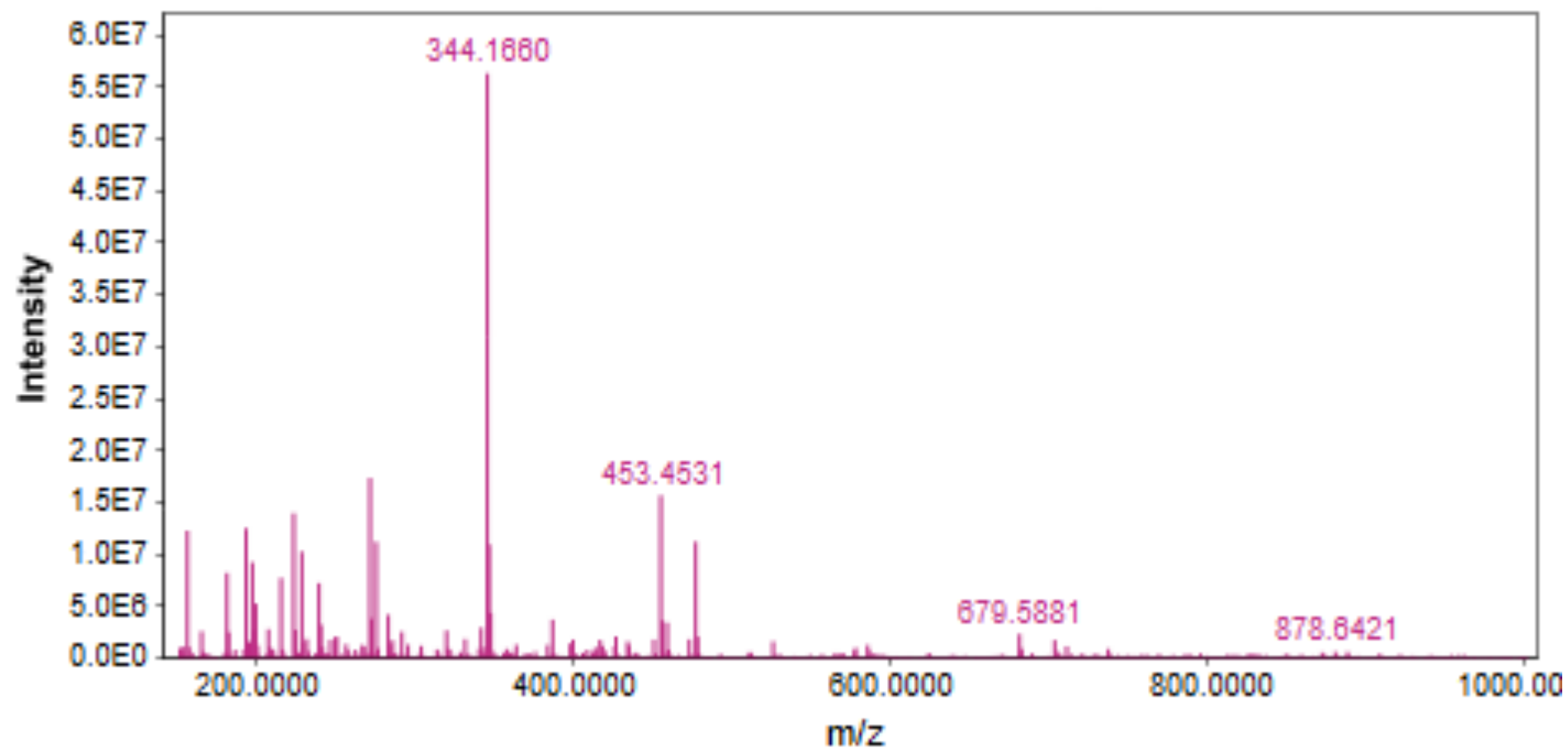

Fig. M25 The mass spectra of target compound **4c-6**

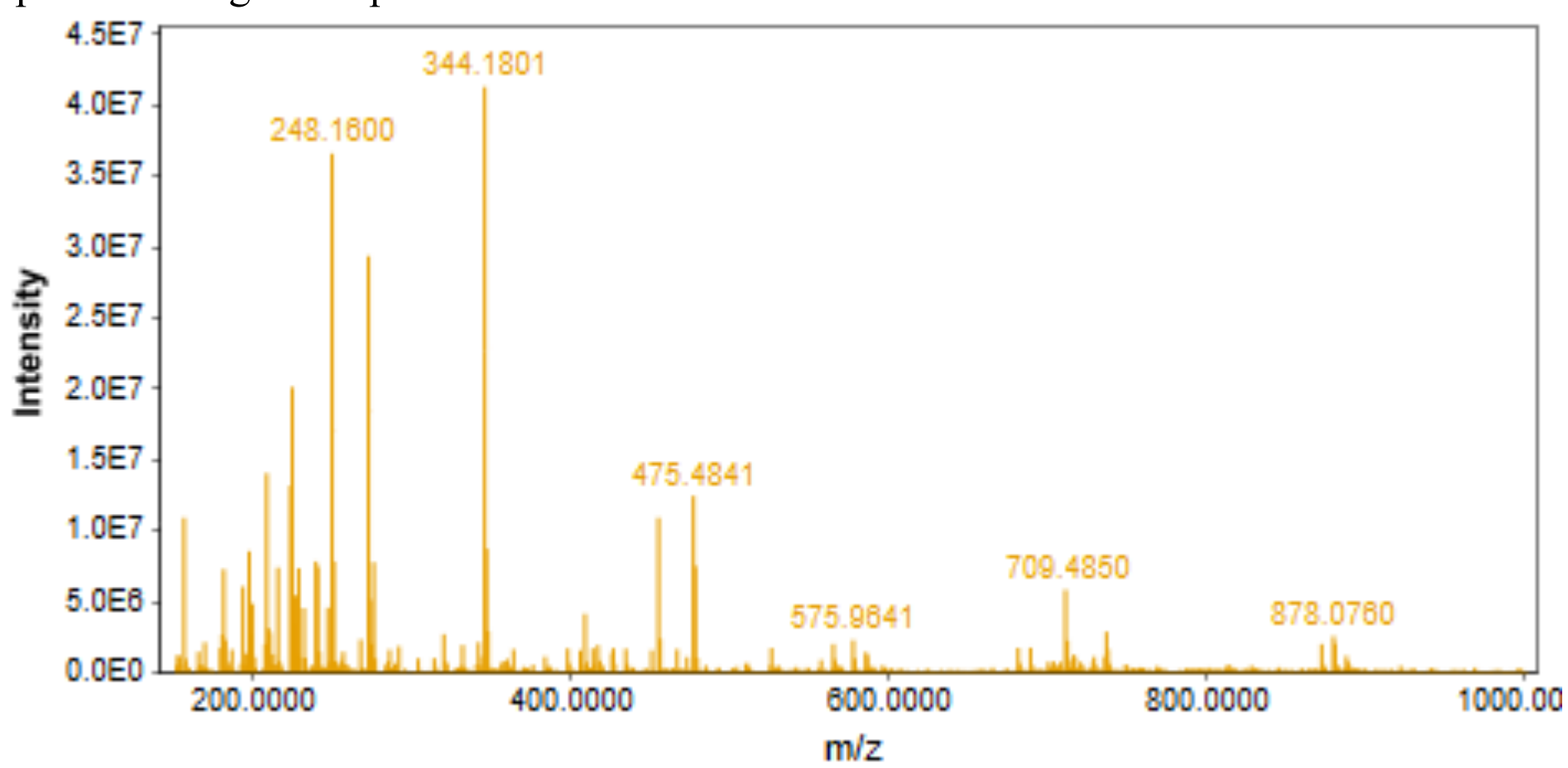

Fig. M26 The mass spectra of target compound **4c-7**

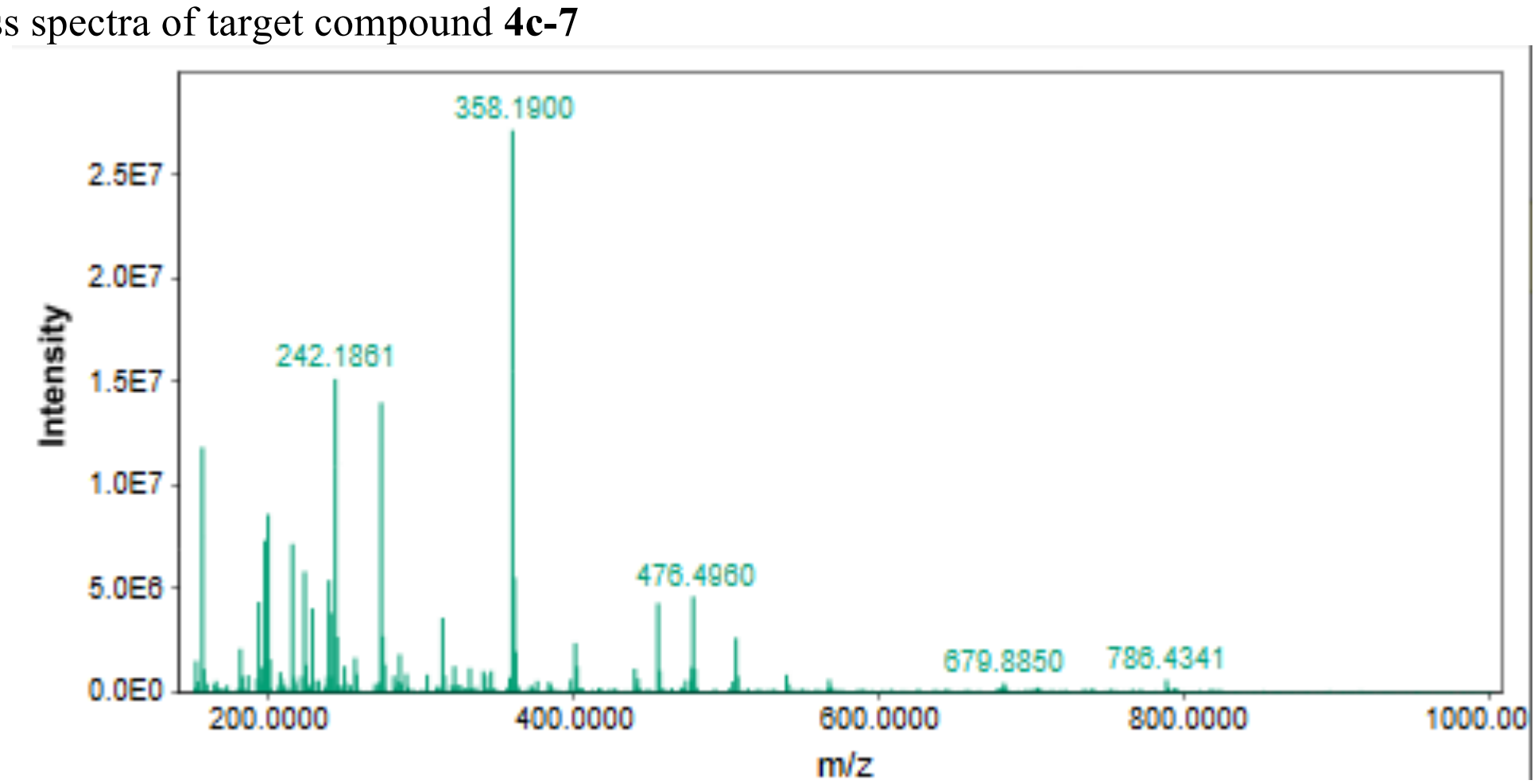

Fig. M27 The mass spectra of target compound **4c-8**

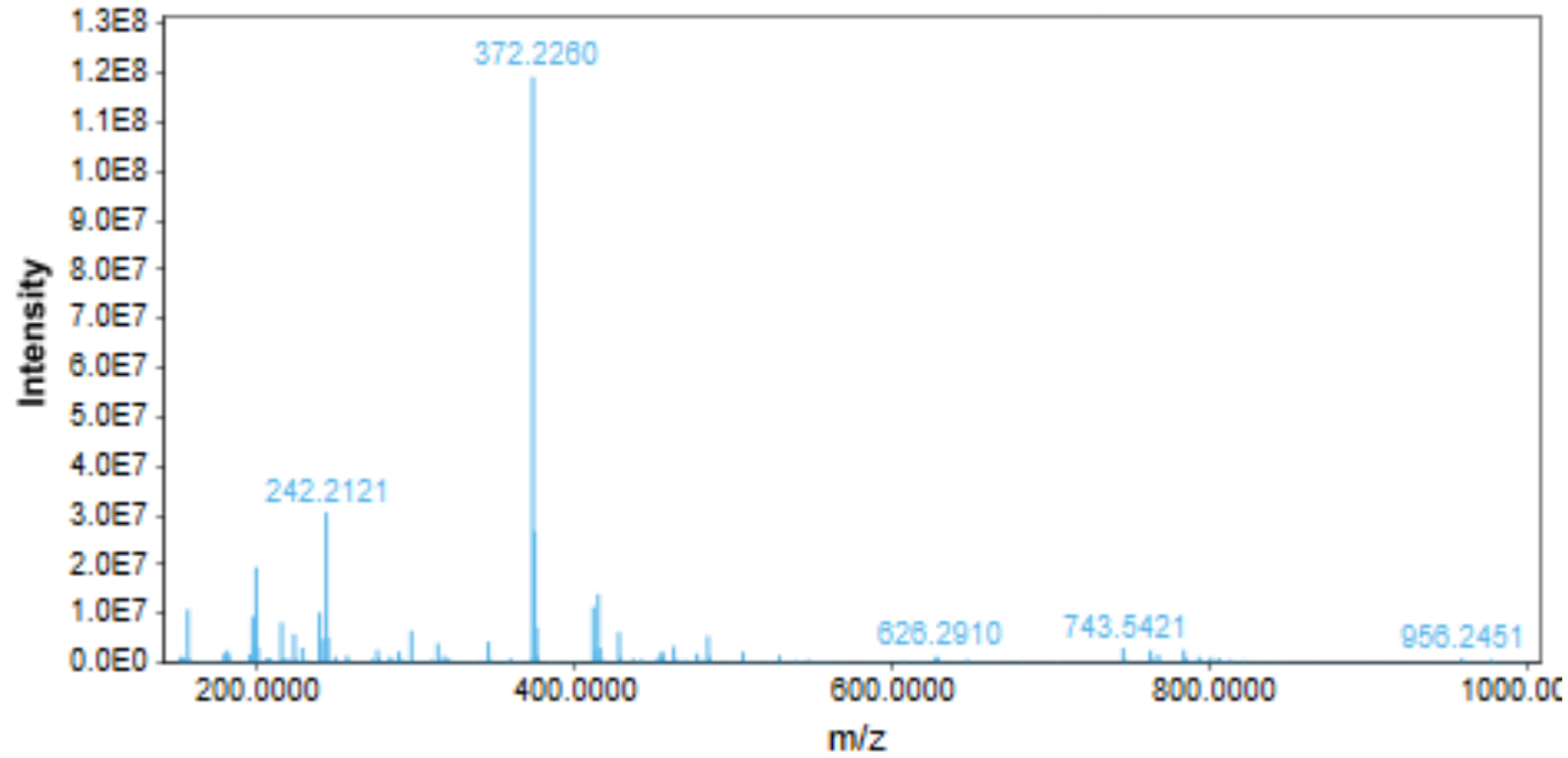

Fig. M28 The mass spectra of target compound **4c-9**

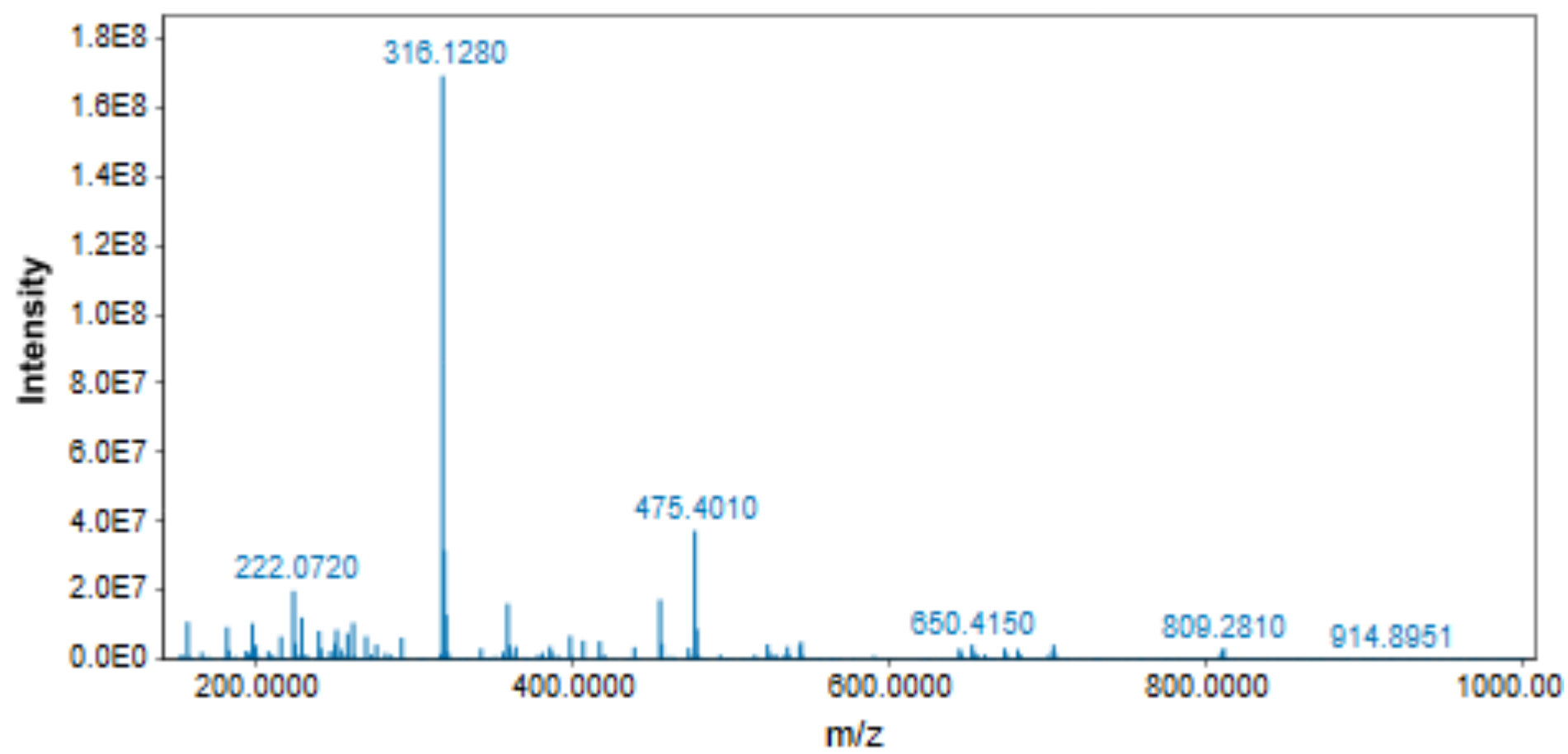

Fig. M29 The mass spectra of target compound **4c-10**

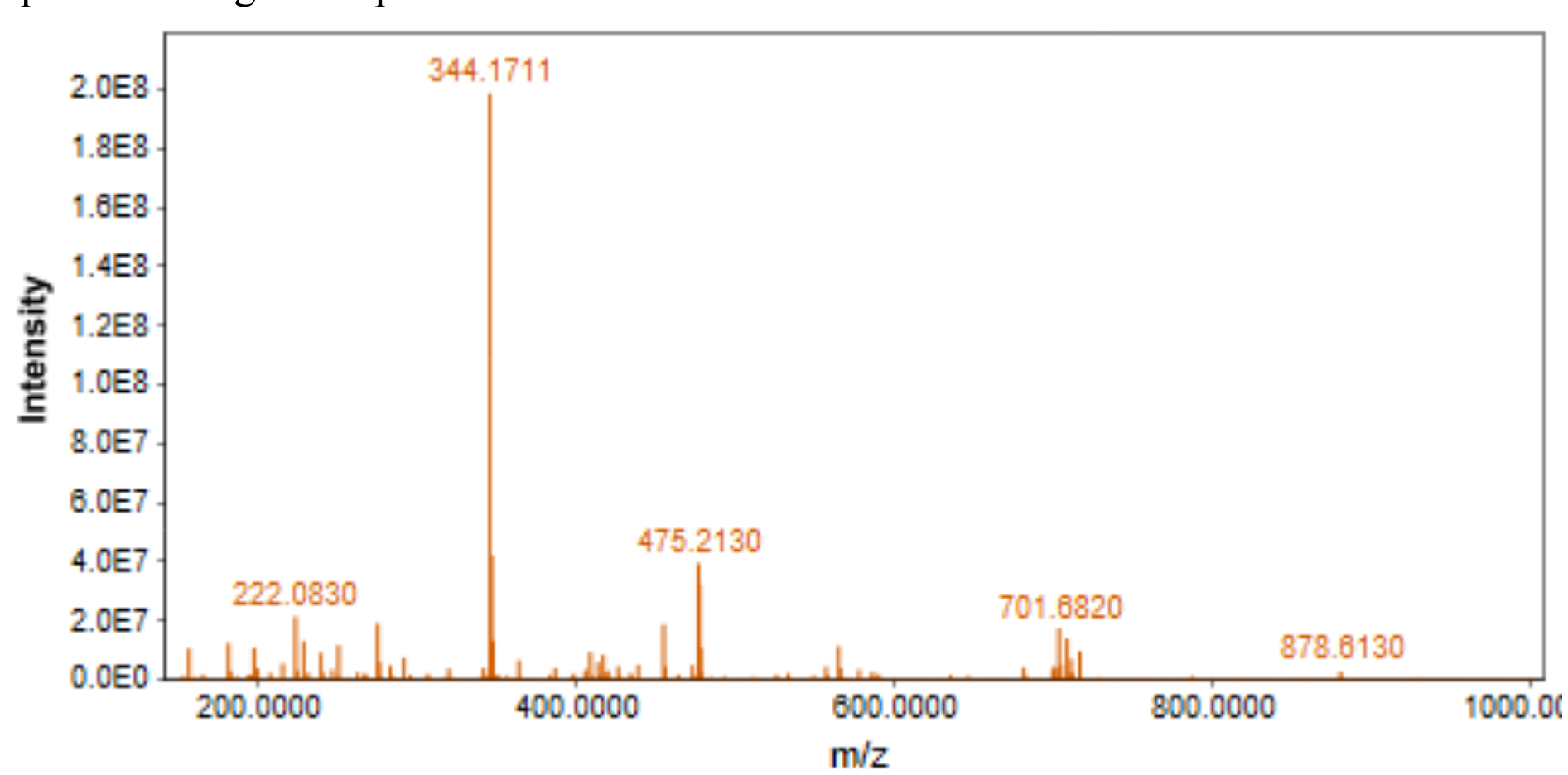

Fig. M30 The mass spectra of target compound **4c-11**

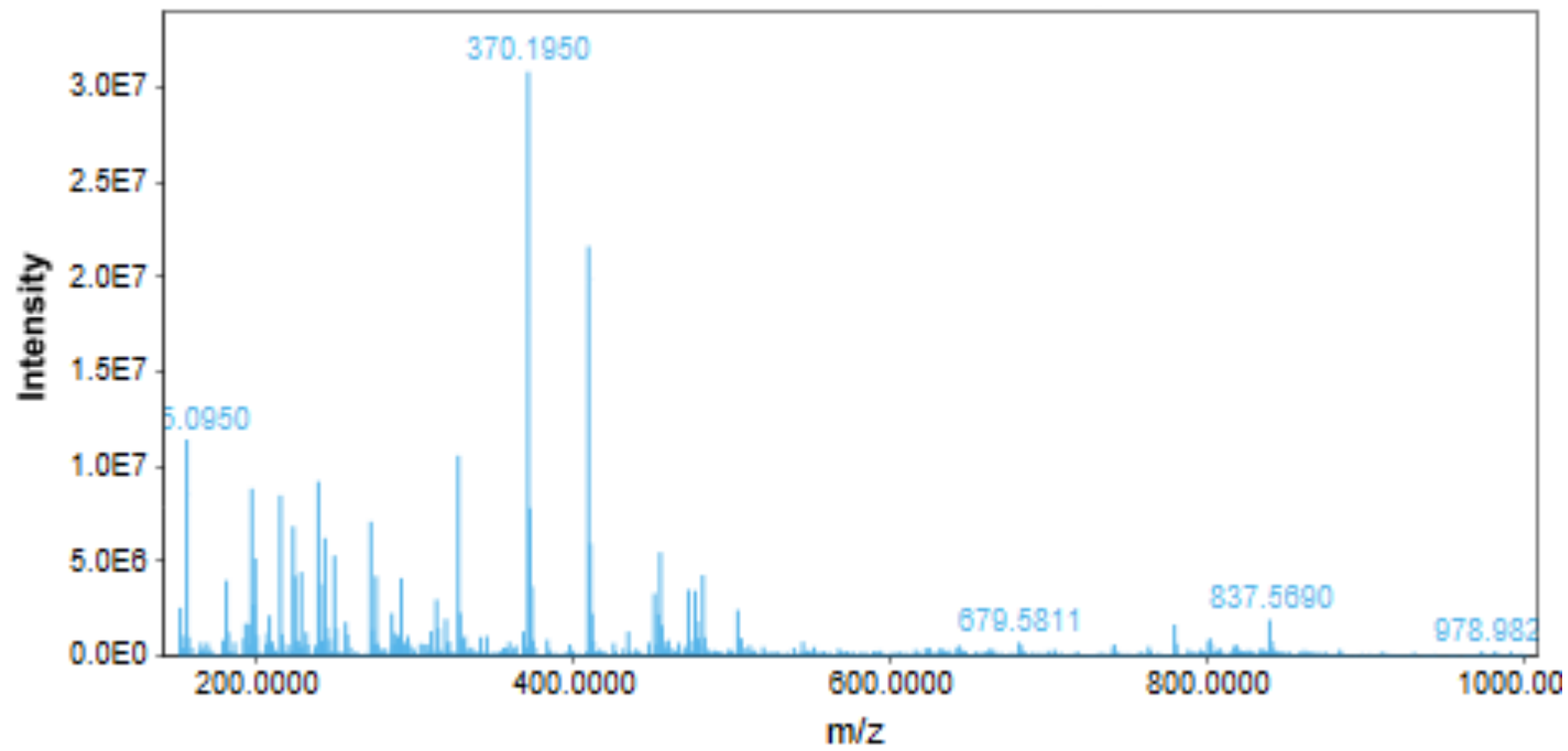

Fig. M31 The mass spectra of target compound **4c-12**

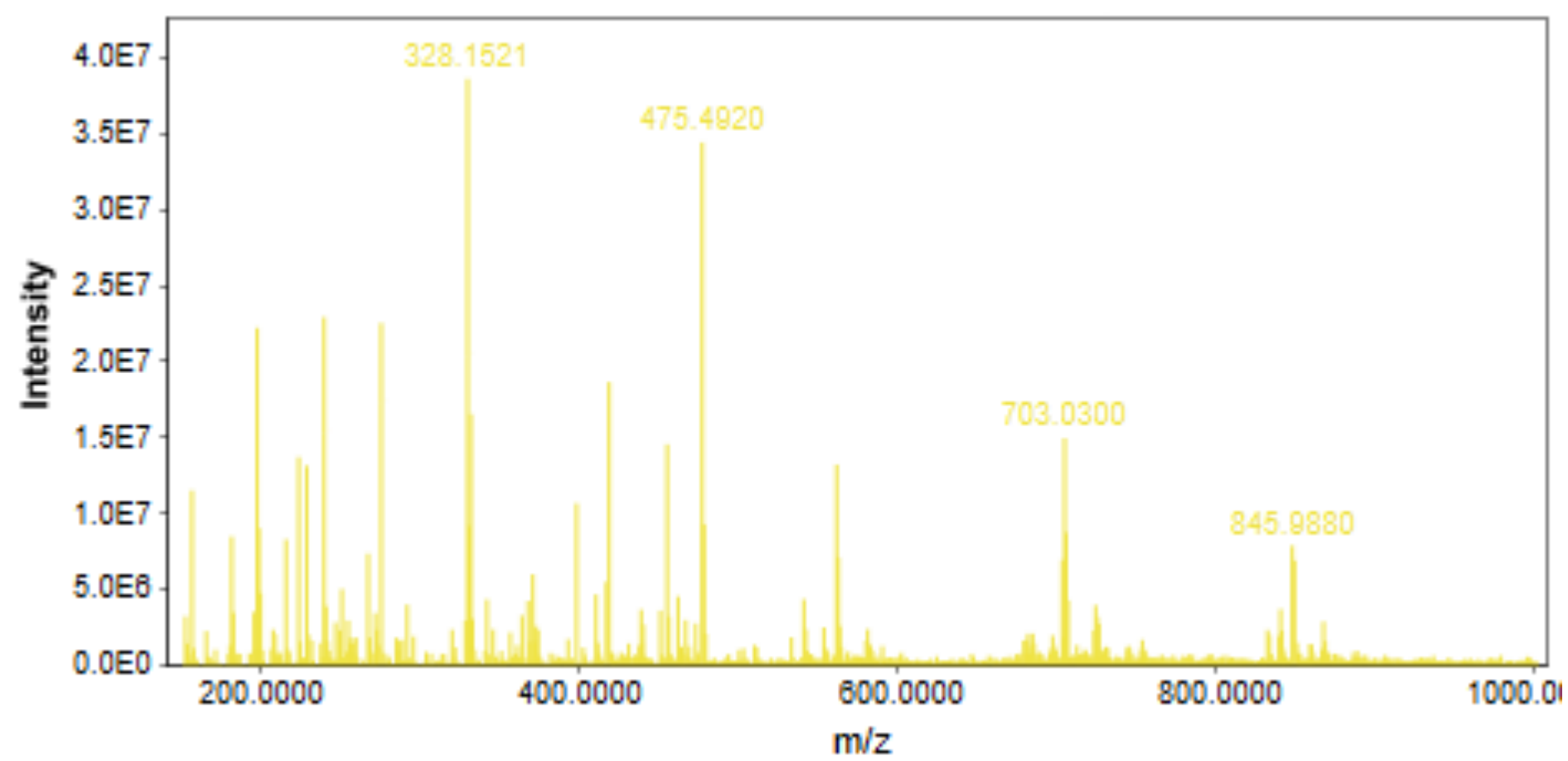

Fig. M32 The mass spectra of target compound **6-1**

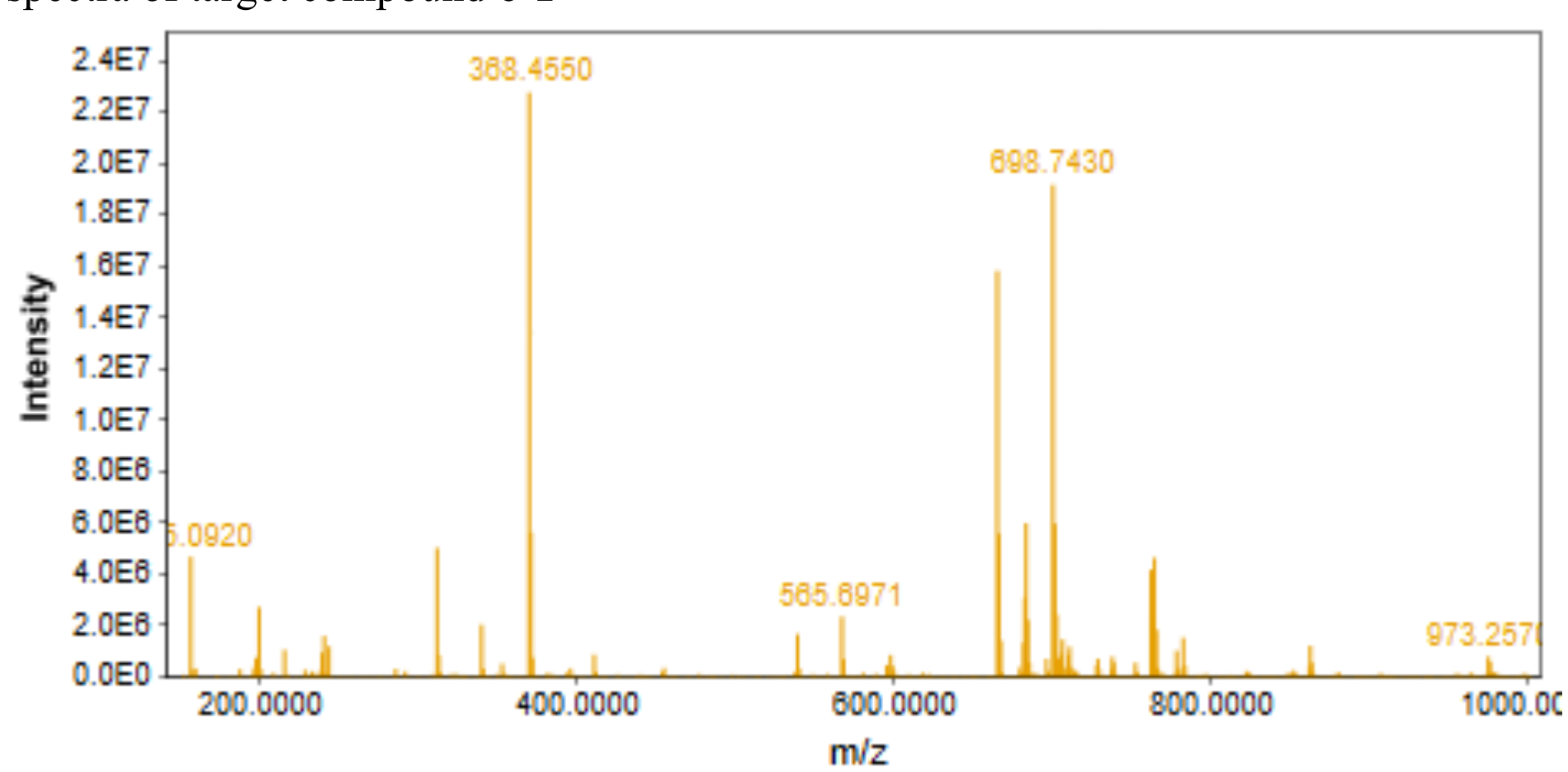

Fig. M33 The mass spectra of target compound **6-2**

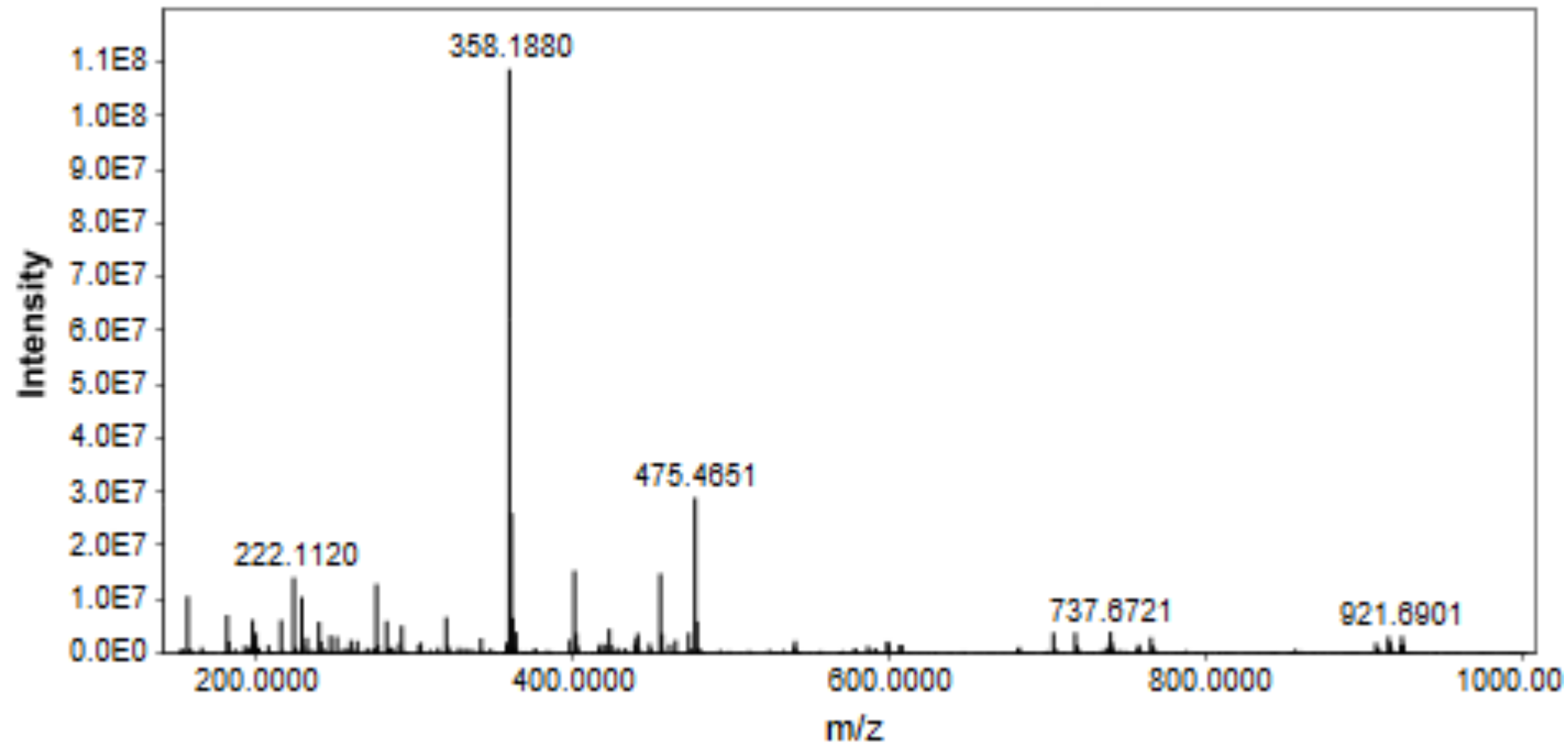

Fig. M34 The mass spectra of target compound **8-1**

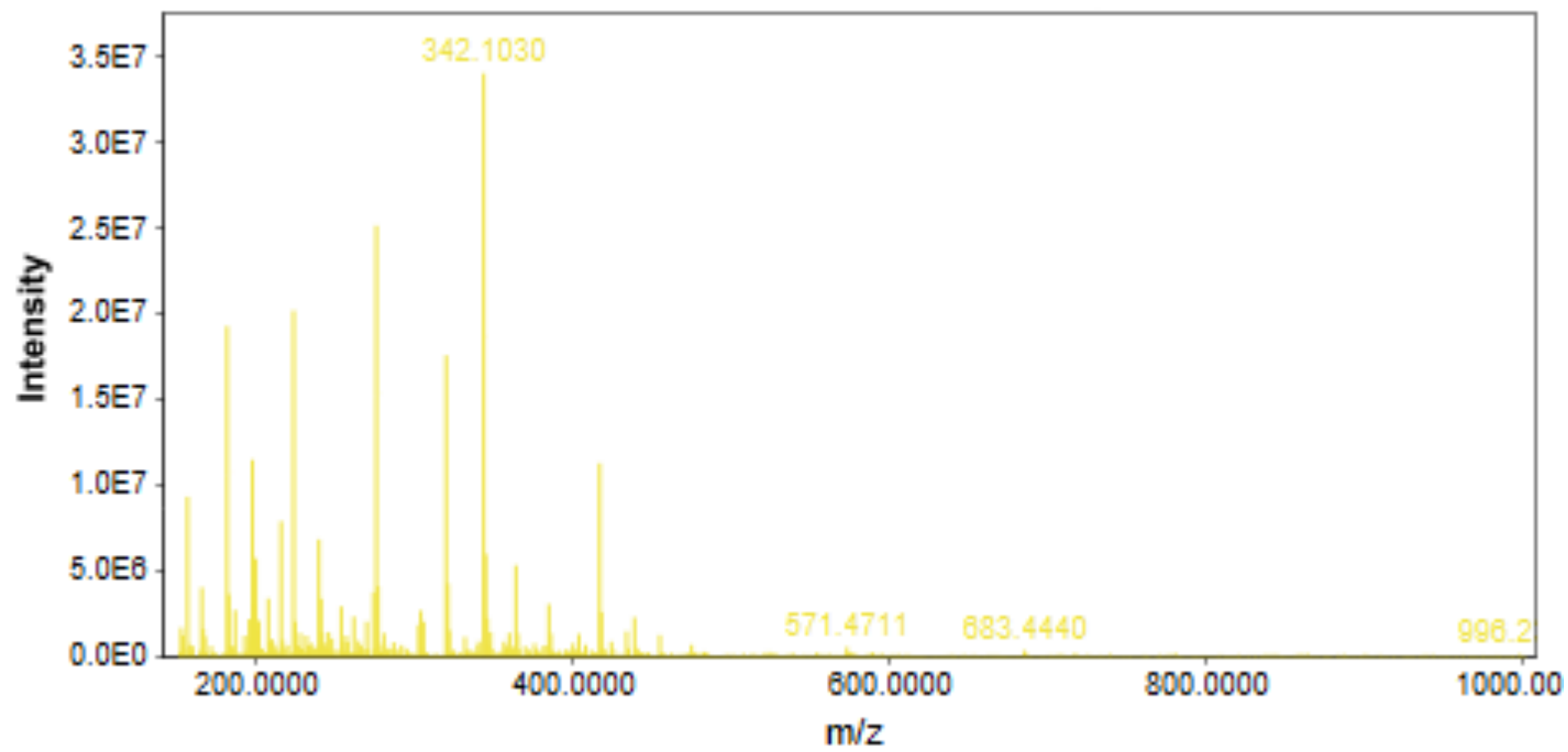

Fig. M35 The mass spectra of target compound **8-2**

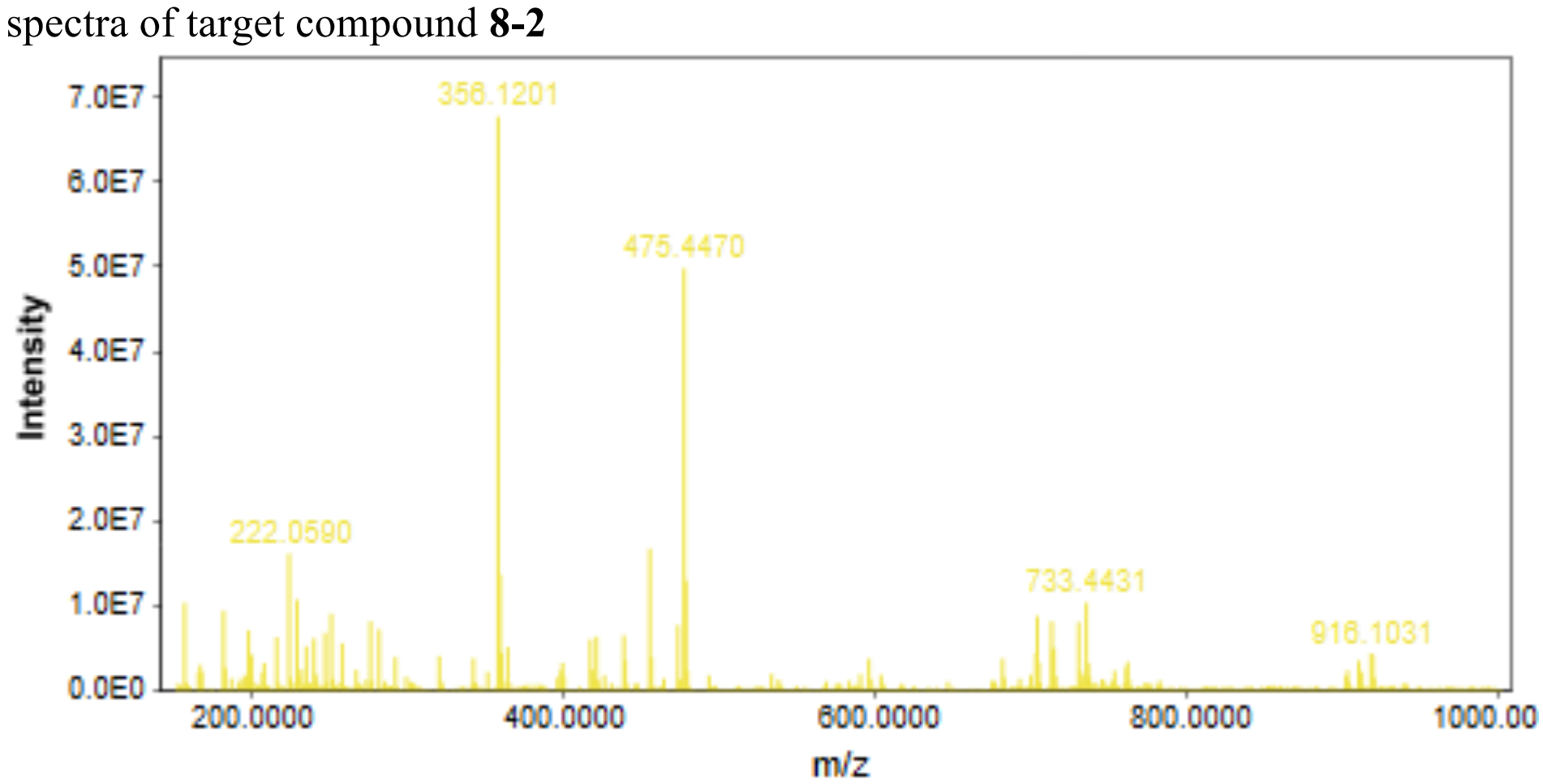

Fig. M36 The mass spectra of target compound **8-3**

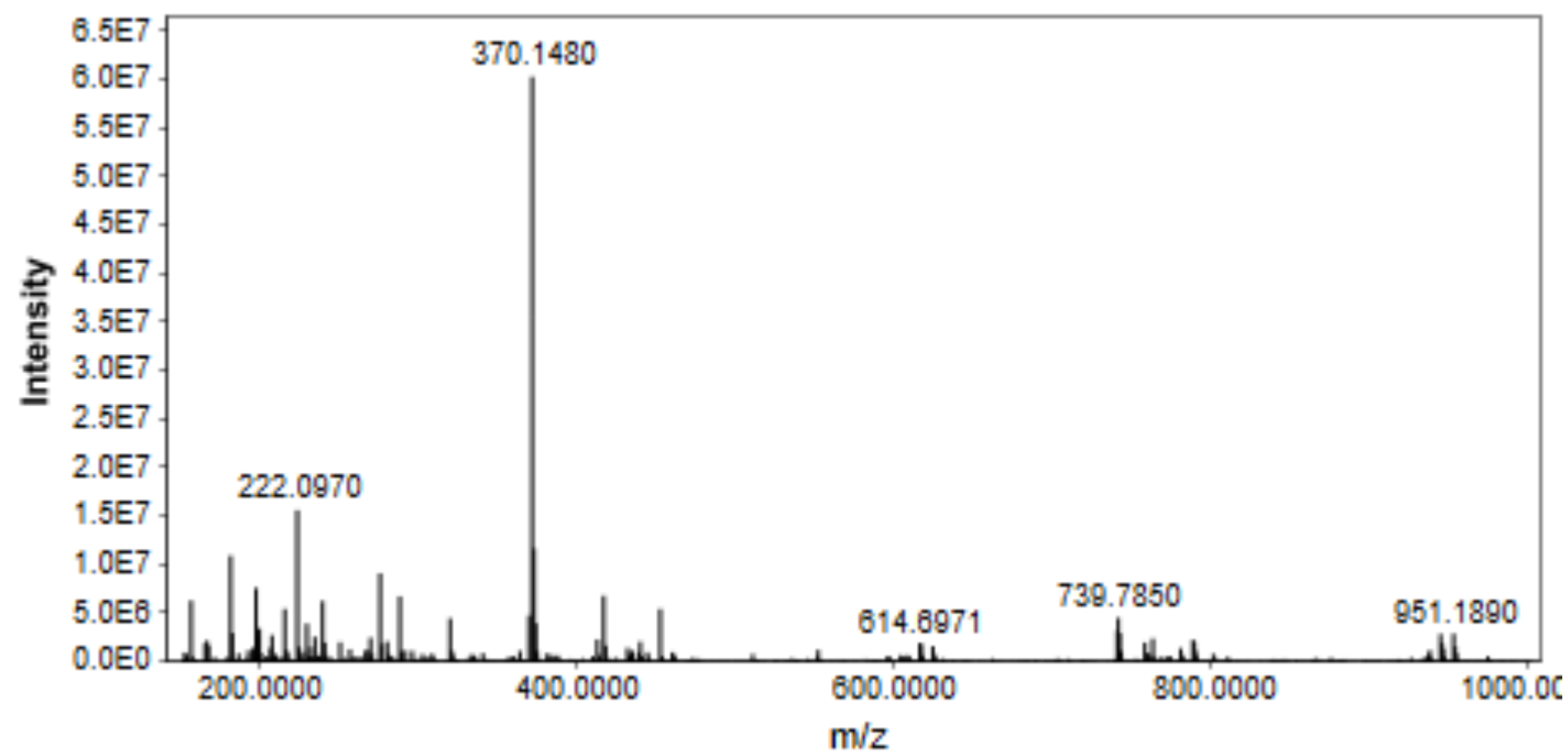

Fig. M37 The mass spectra of target compound **8-4**

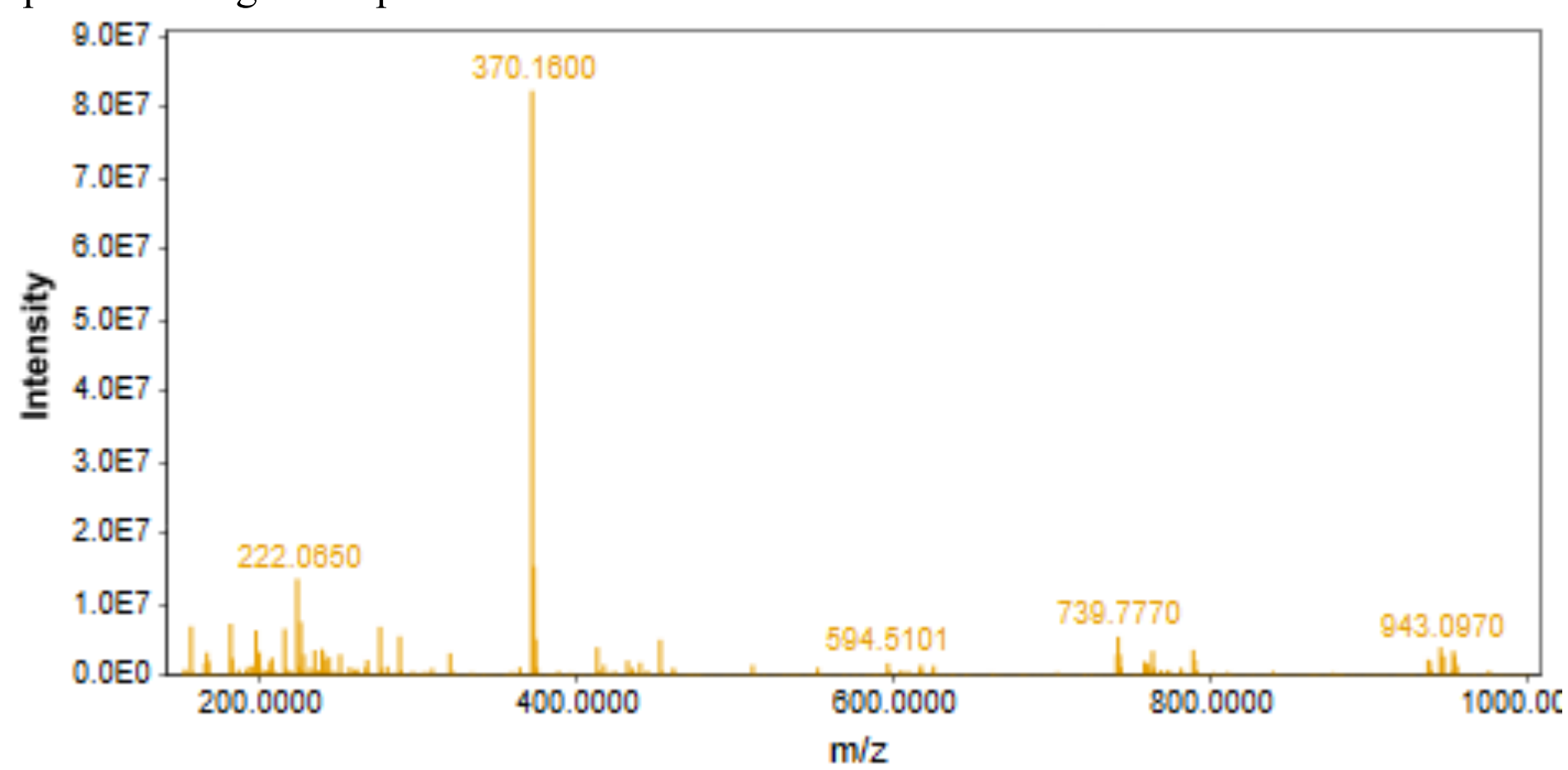

Fig. M38 The mass spectra of target compound **10-1**

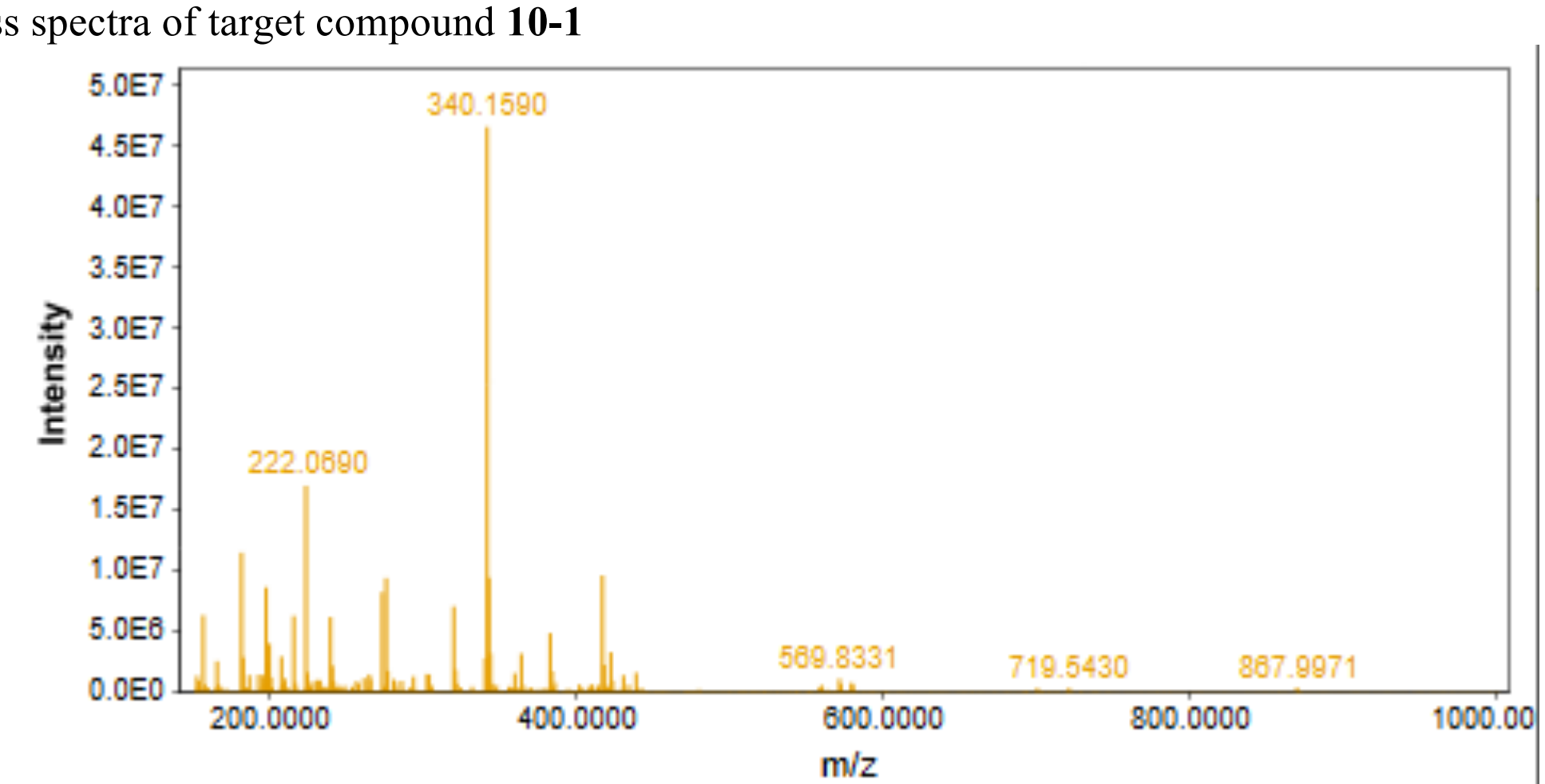

Fig. M39 The mass spectra of target compound **10-2**

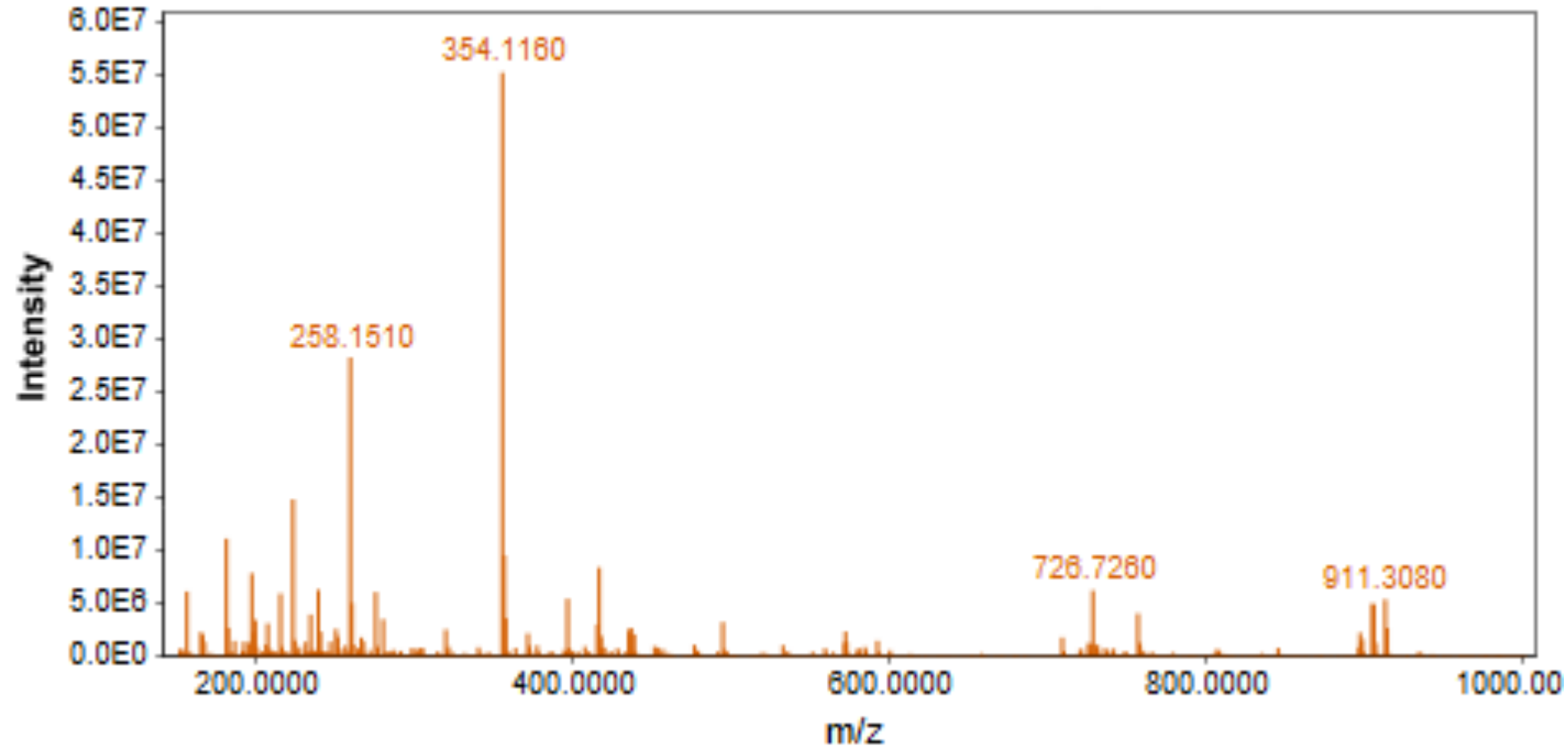

Fig. M40 The mass spectra of target compound **10-3**

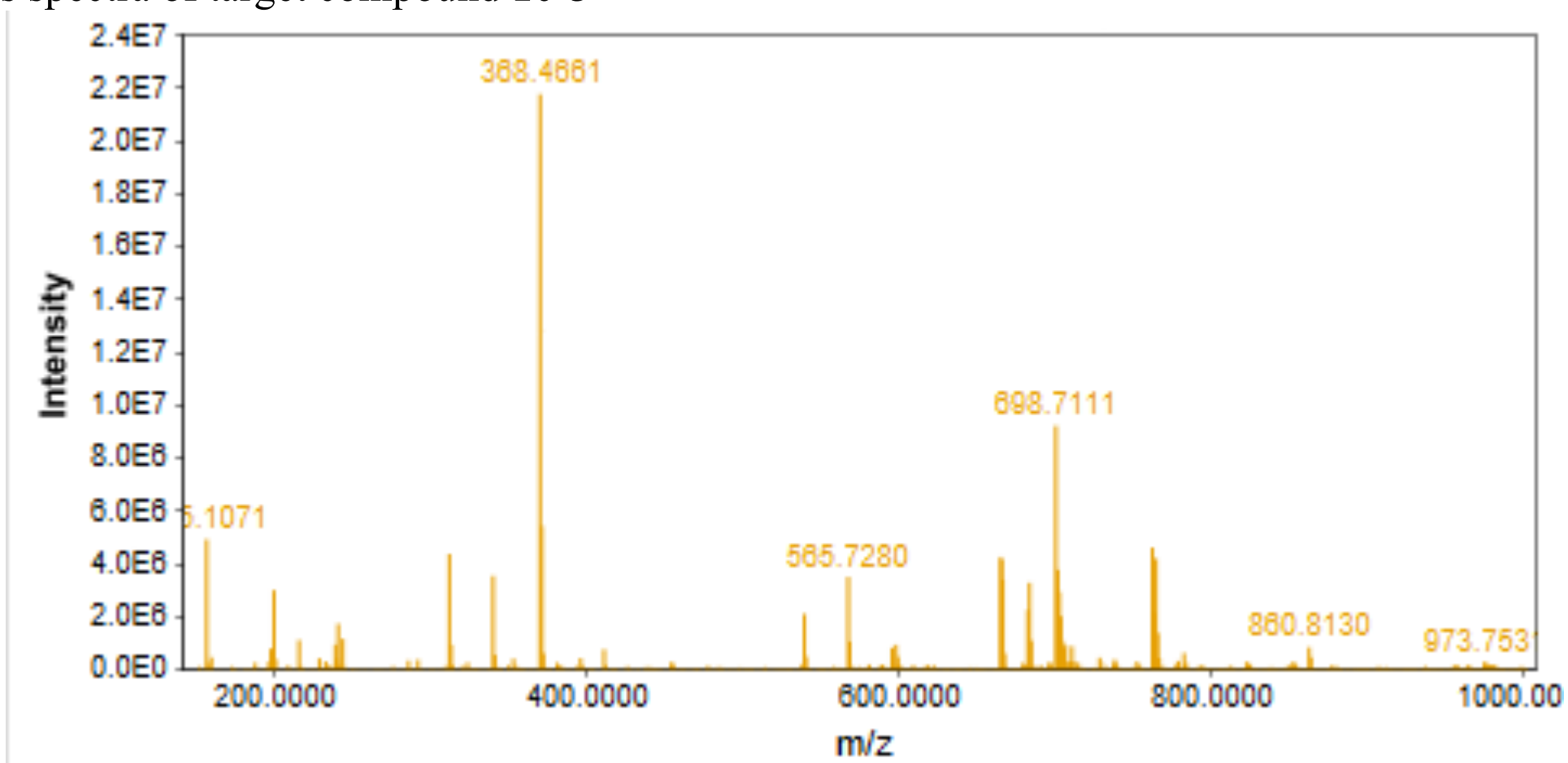

Fig. M41 The mass spectra of target compound **10-4**

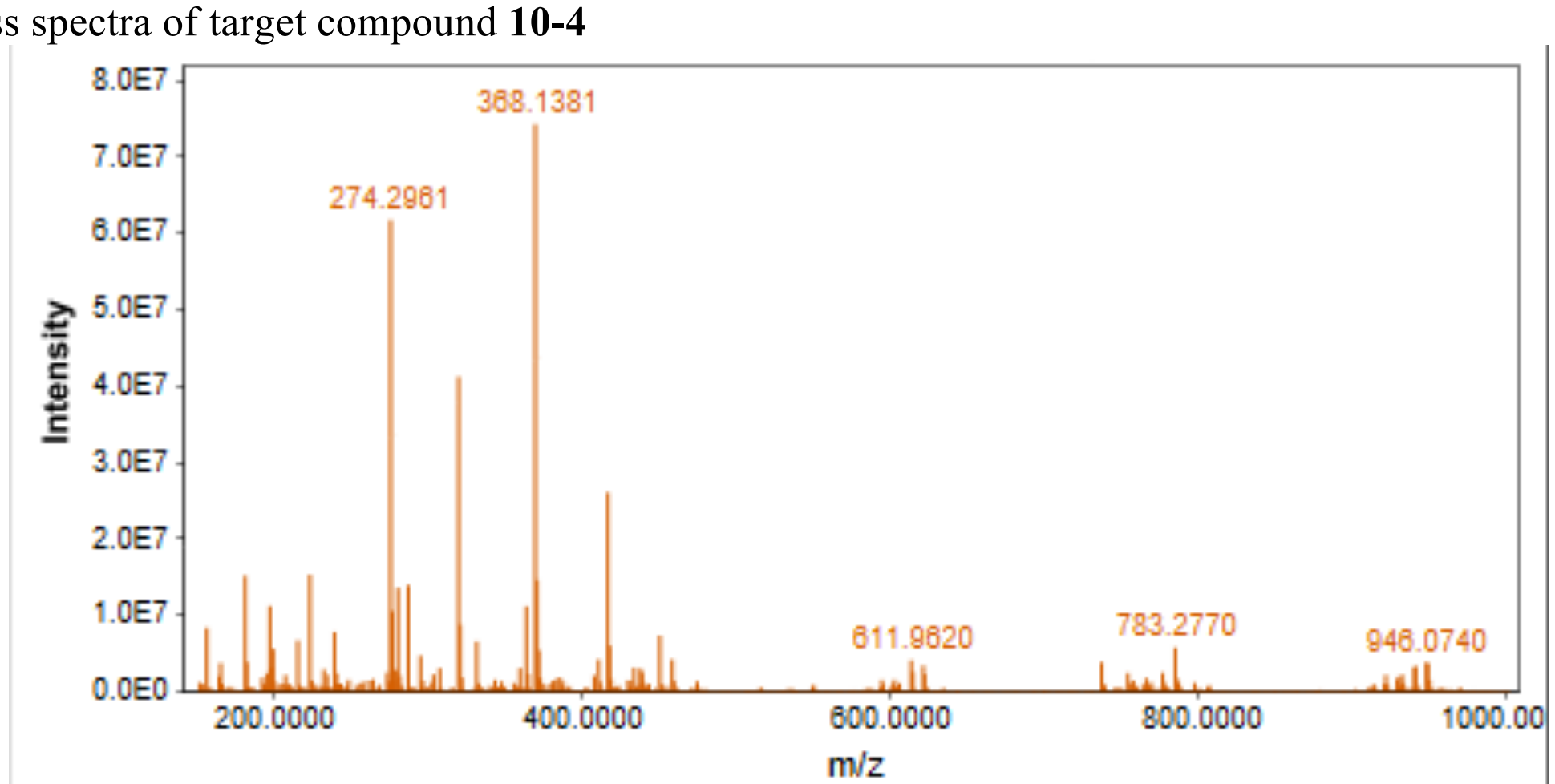

Fig. M42 The mass spectra of target compound **S4-1**

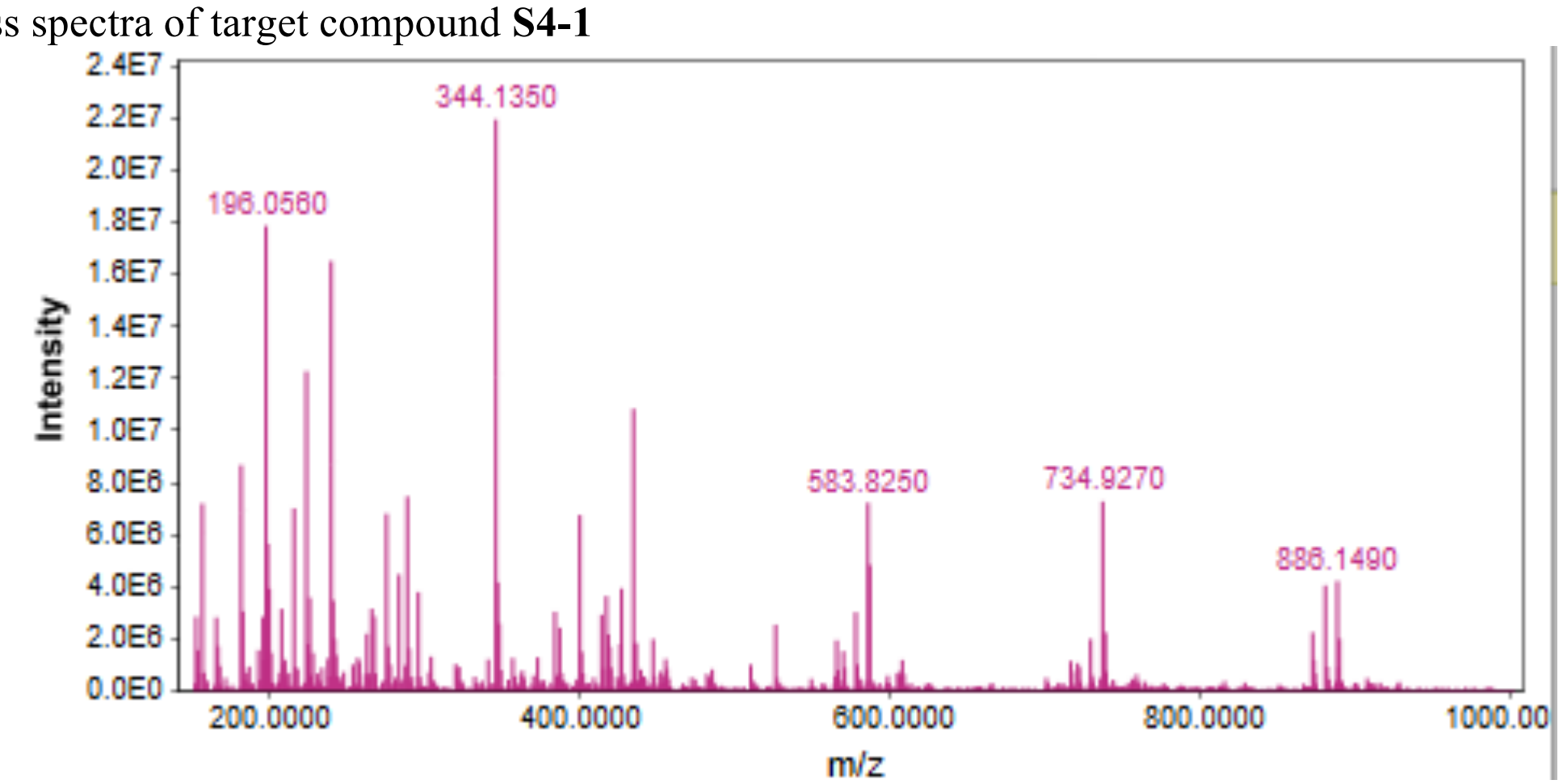

Fig. M43 The mass spectra of target compound **S4-2**

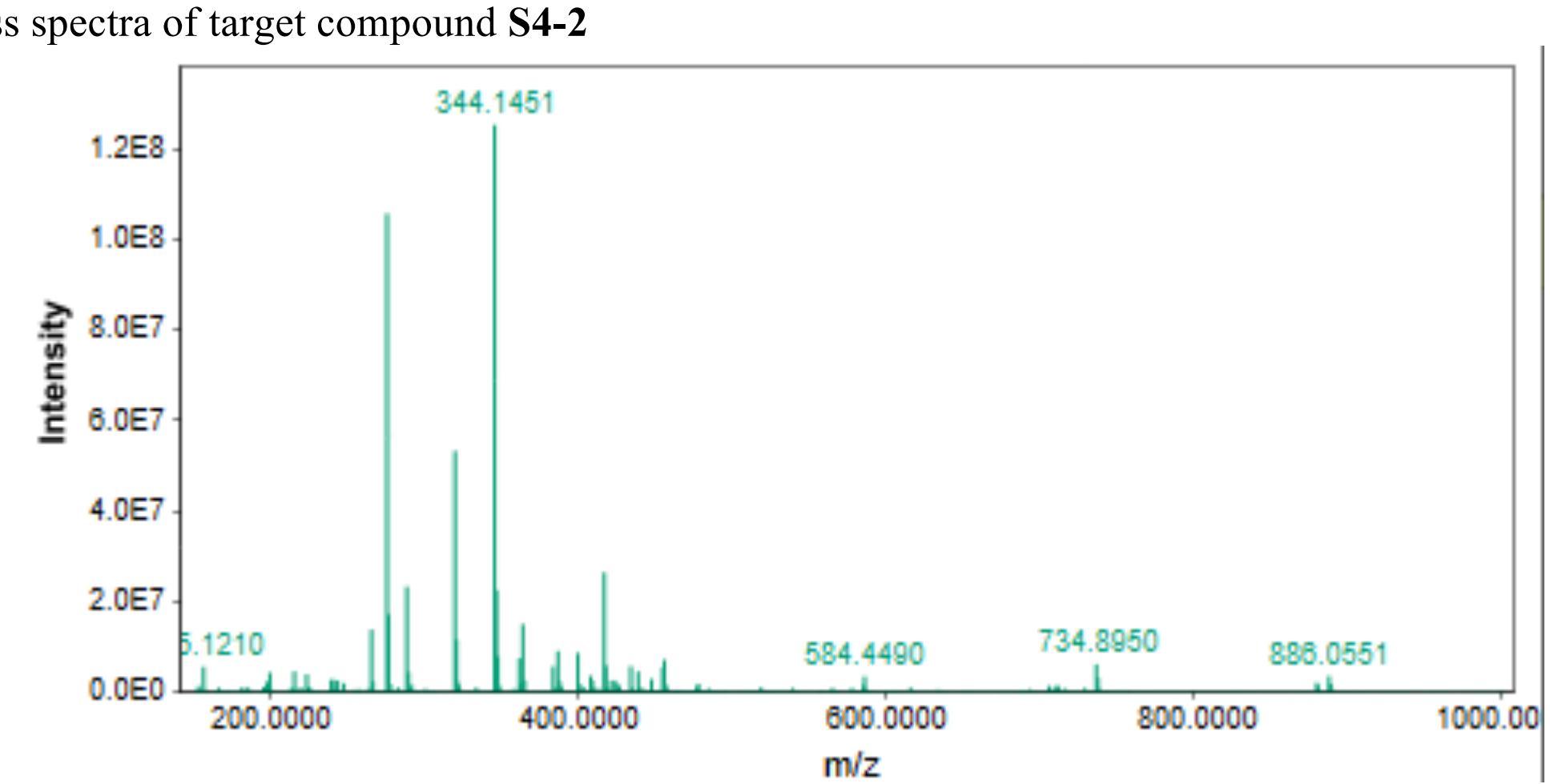

Fig. M44 The mass spectra of target compound **S6-1**

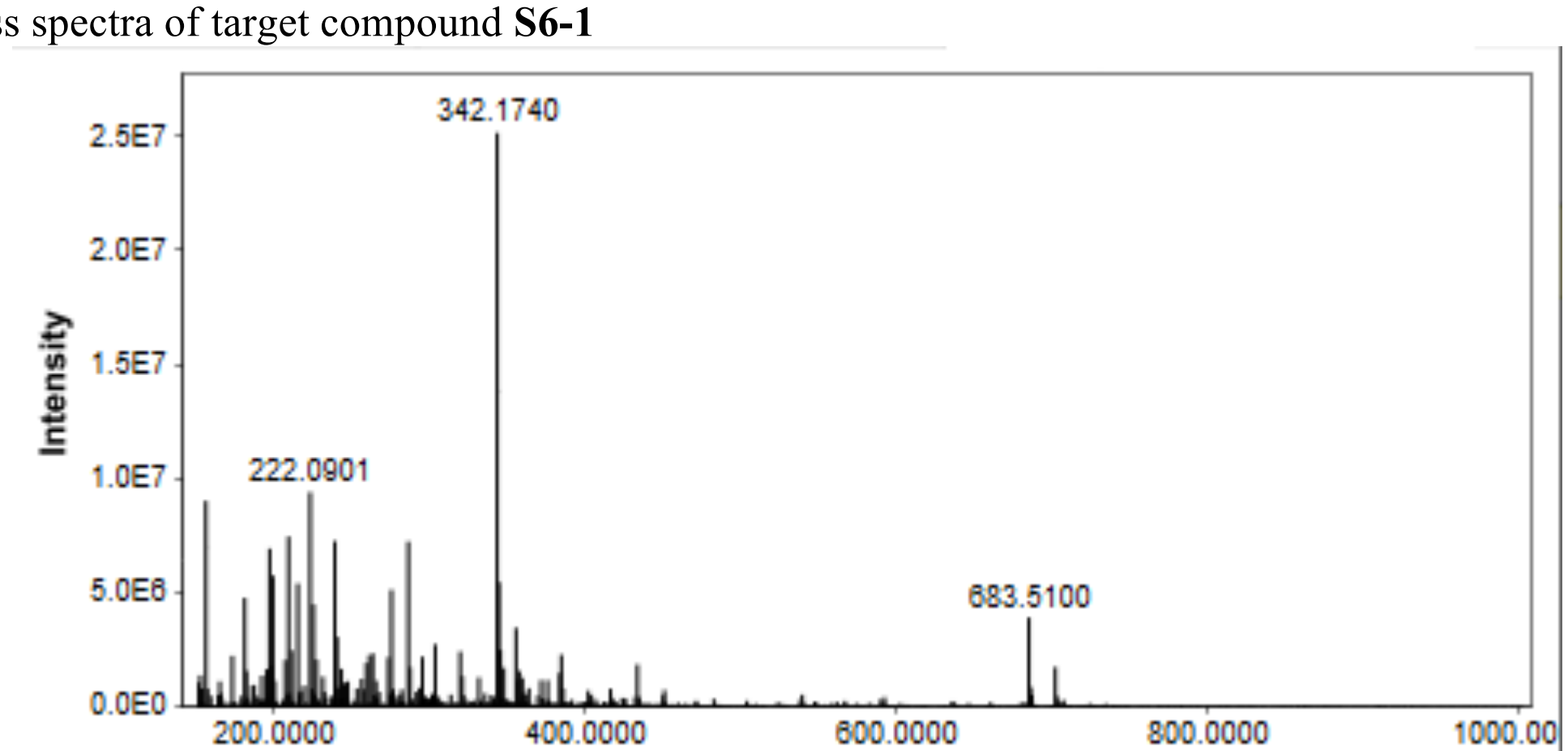

Fig. M45 The mass spectra of target compound **S6-2**

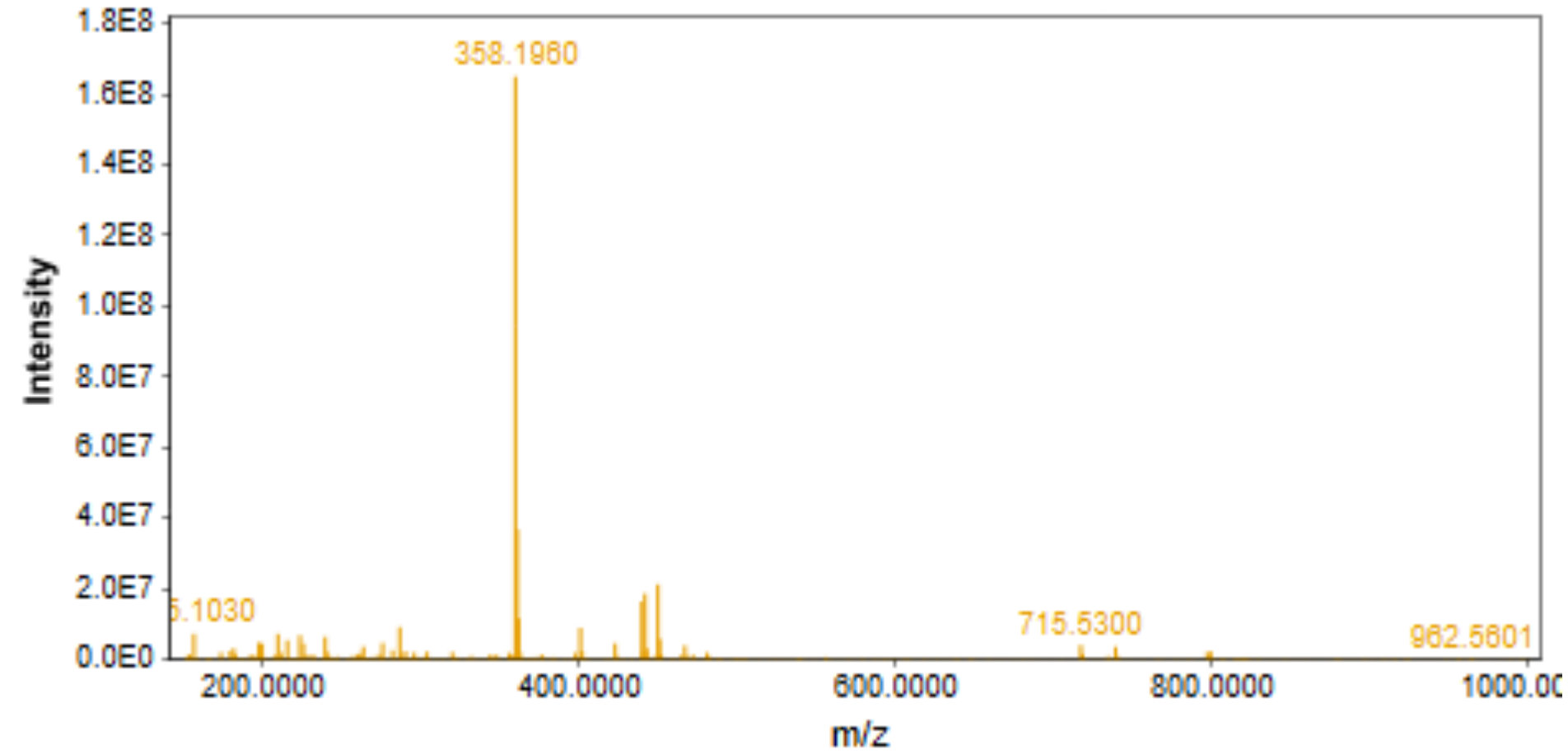

Fig. M46 The mass spectra of target compound **S6-3**

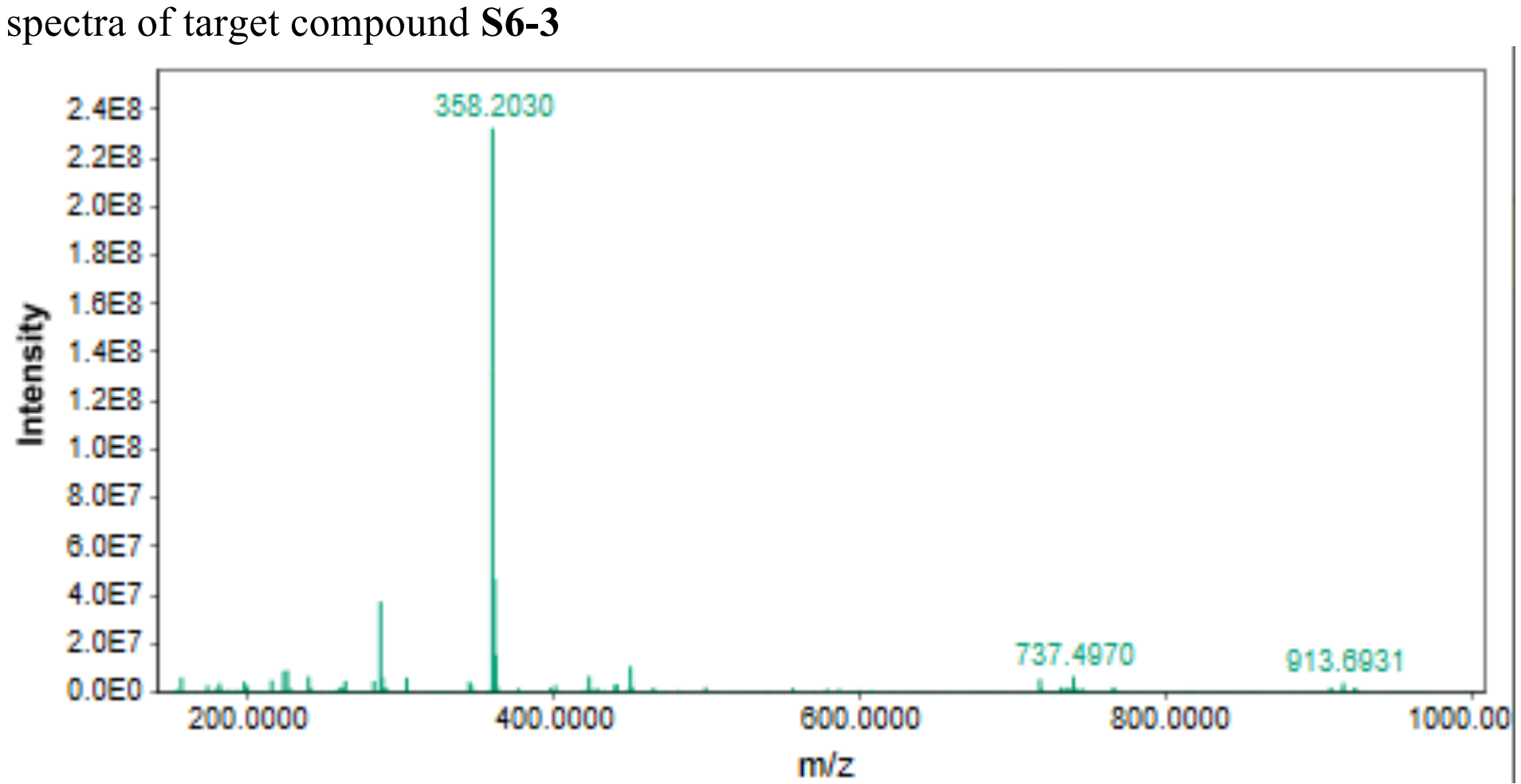

Fig. M47 The mass spectra of target compound **S6-4**

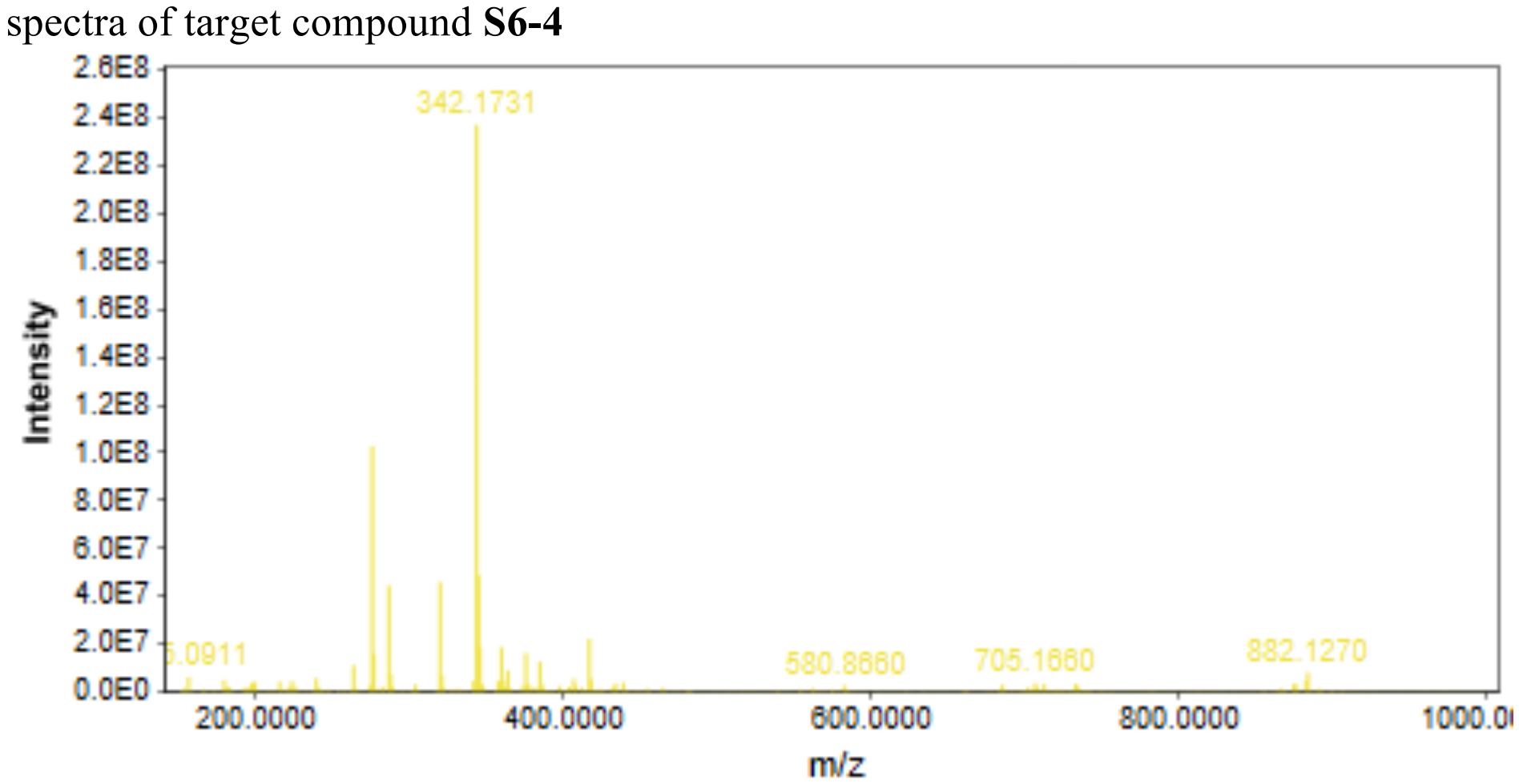

Fig. M48 The mass spectra of target compound **S8-1**

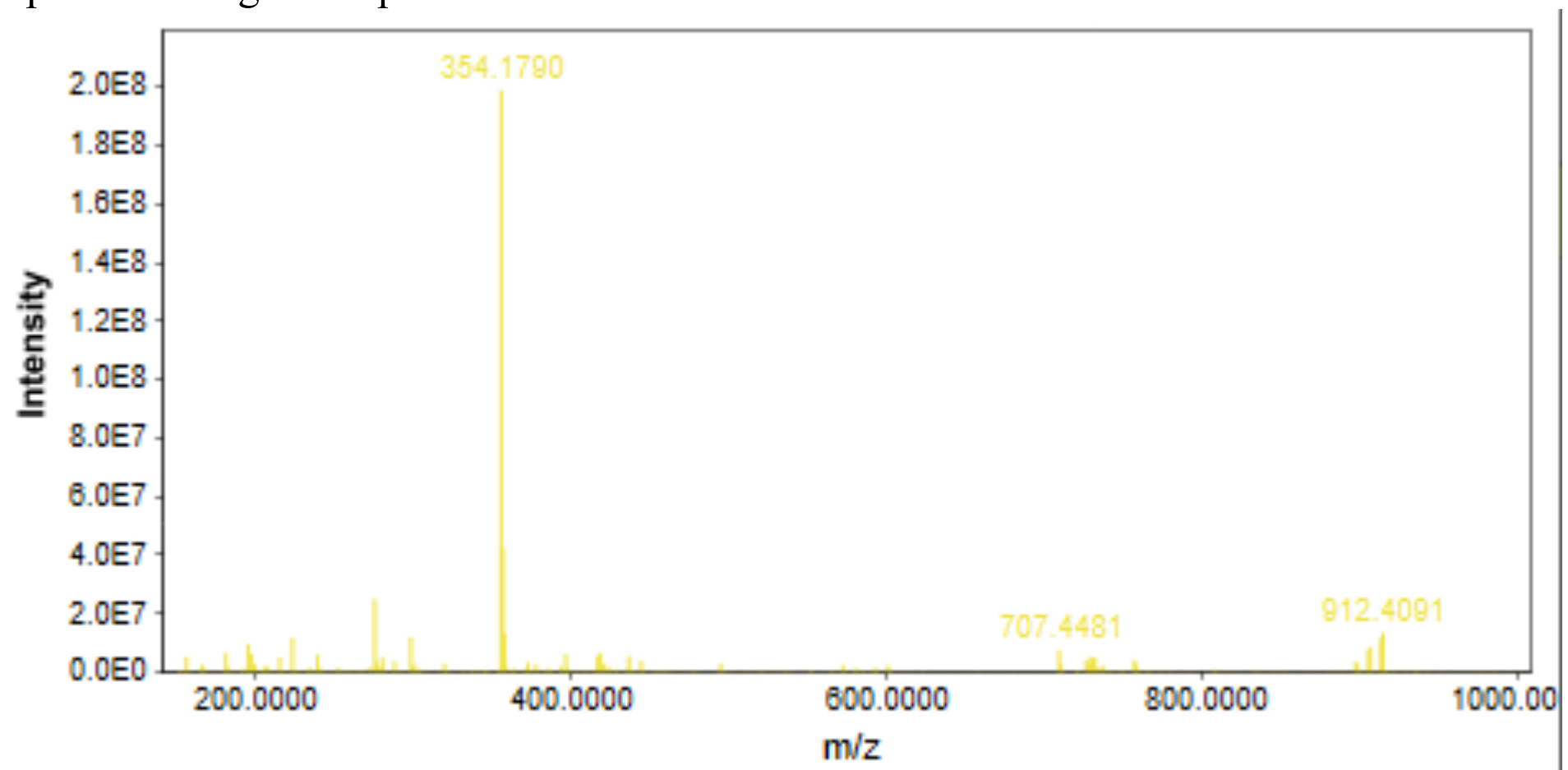

Fig. M49 The mass spectra of target compound **S8-2**

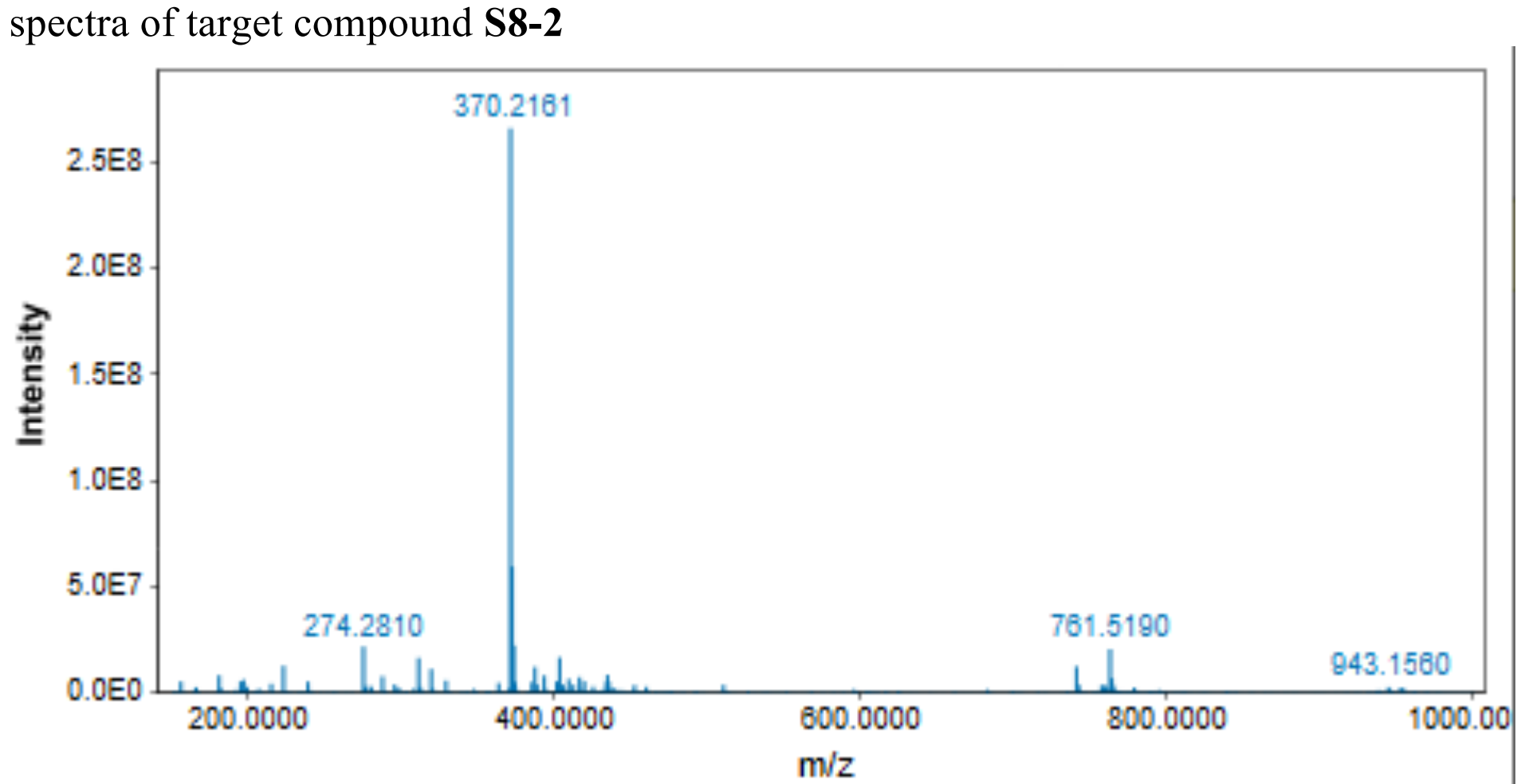

Fig. M50 The mass spectra of target compound **S8-3**

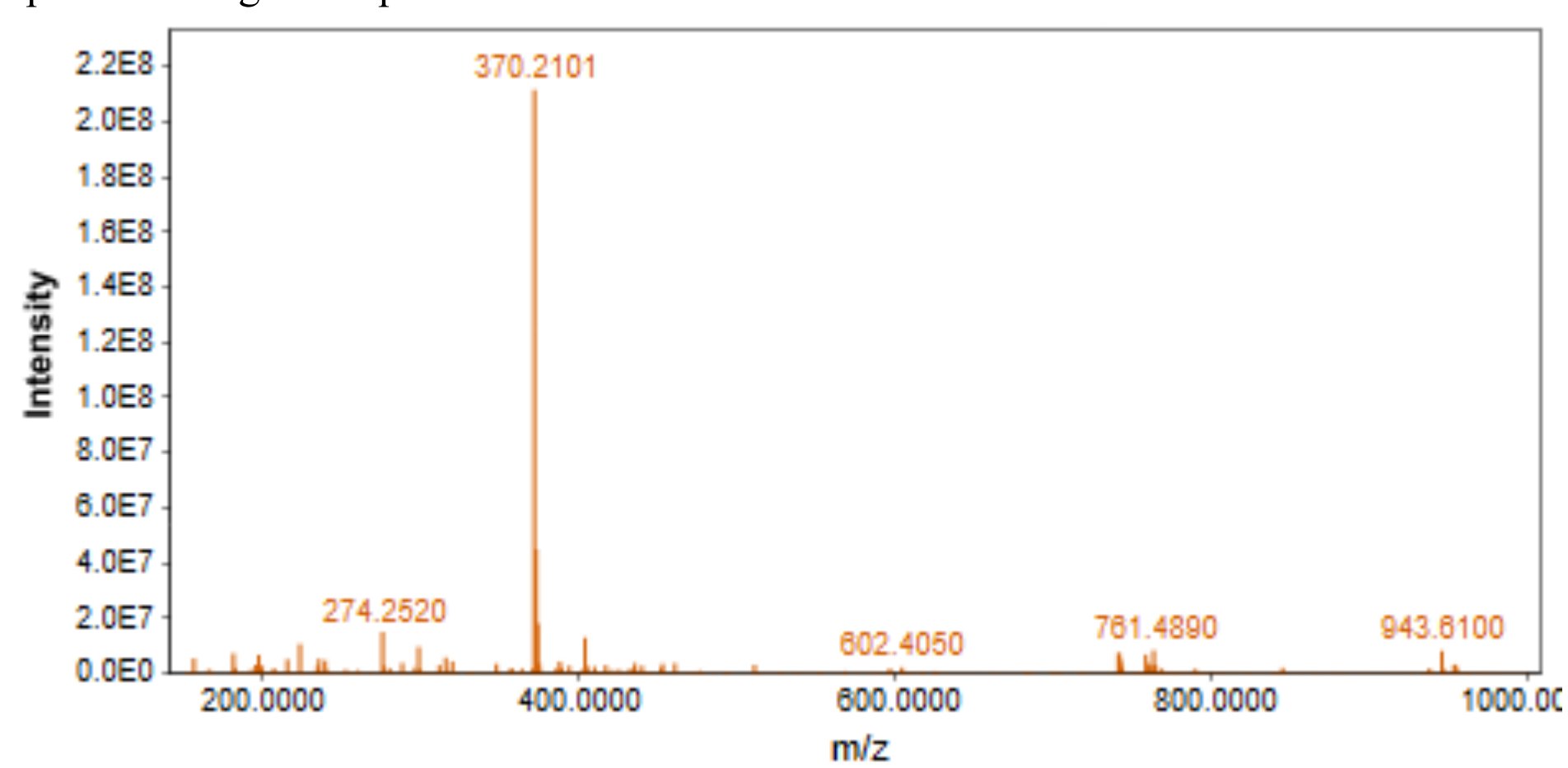

Fig. M51 The mass spectra of target compound **S8-4**

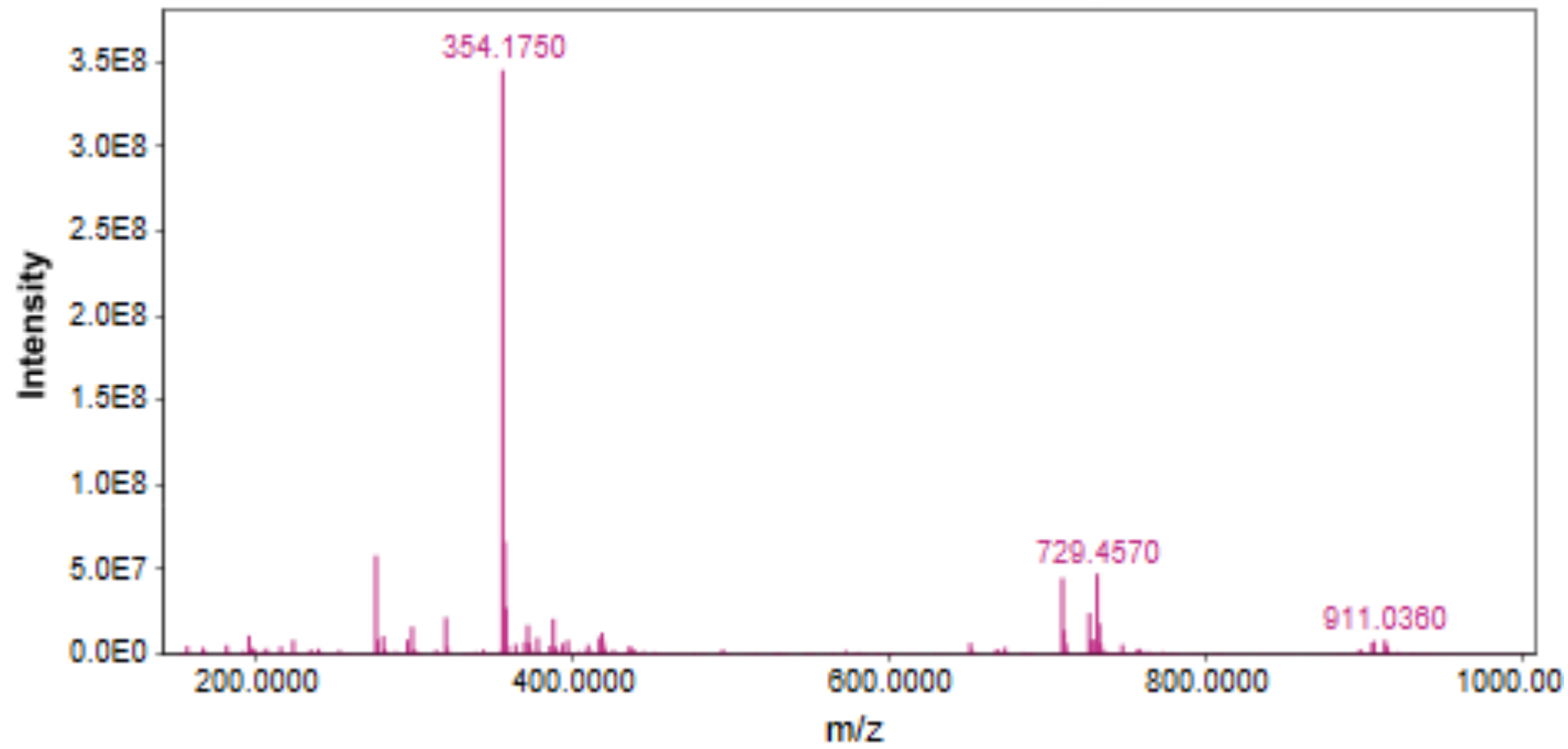

Fig. M52 The mass spectra of target compound **S10-1**

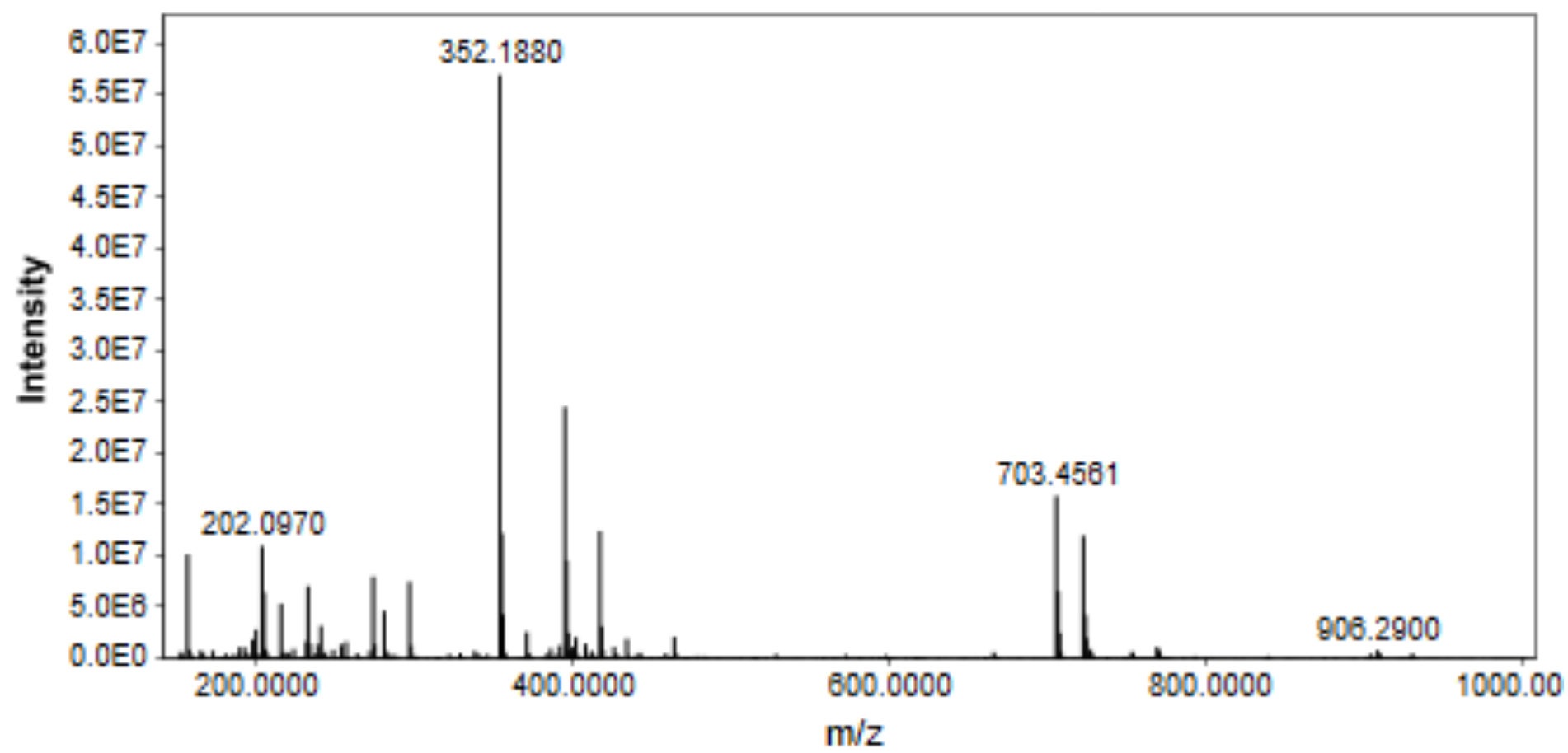

Fig. M53 The mass spectra of target compound **S10-2**

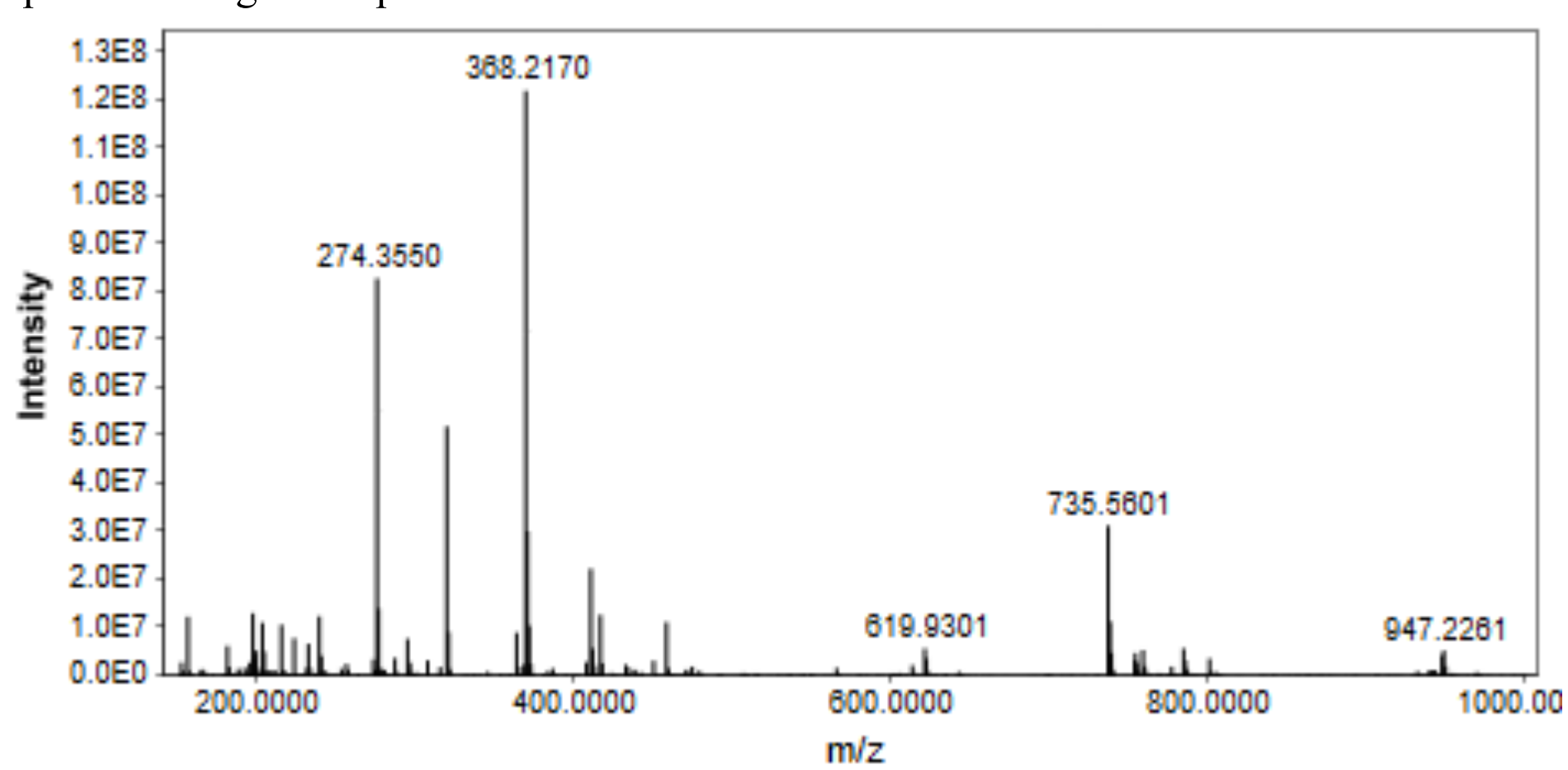

Fig. M54 The mass spectra of target compound **S10-3**

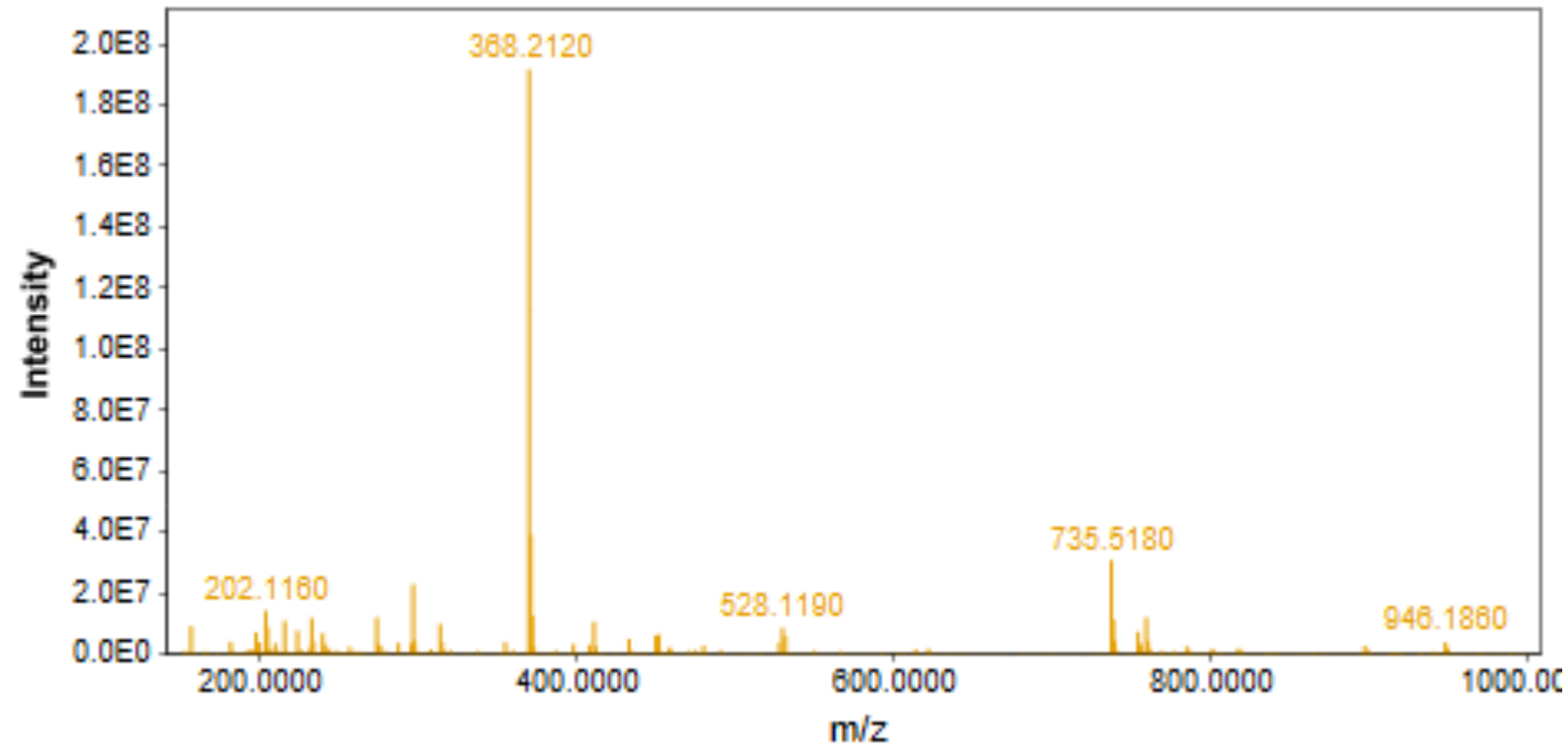

Fig. M55 The mass spectra of target compound **S10-4**

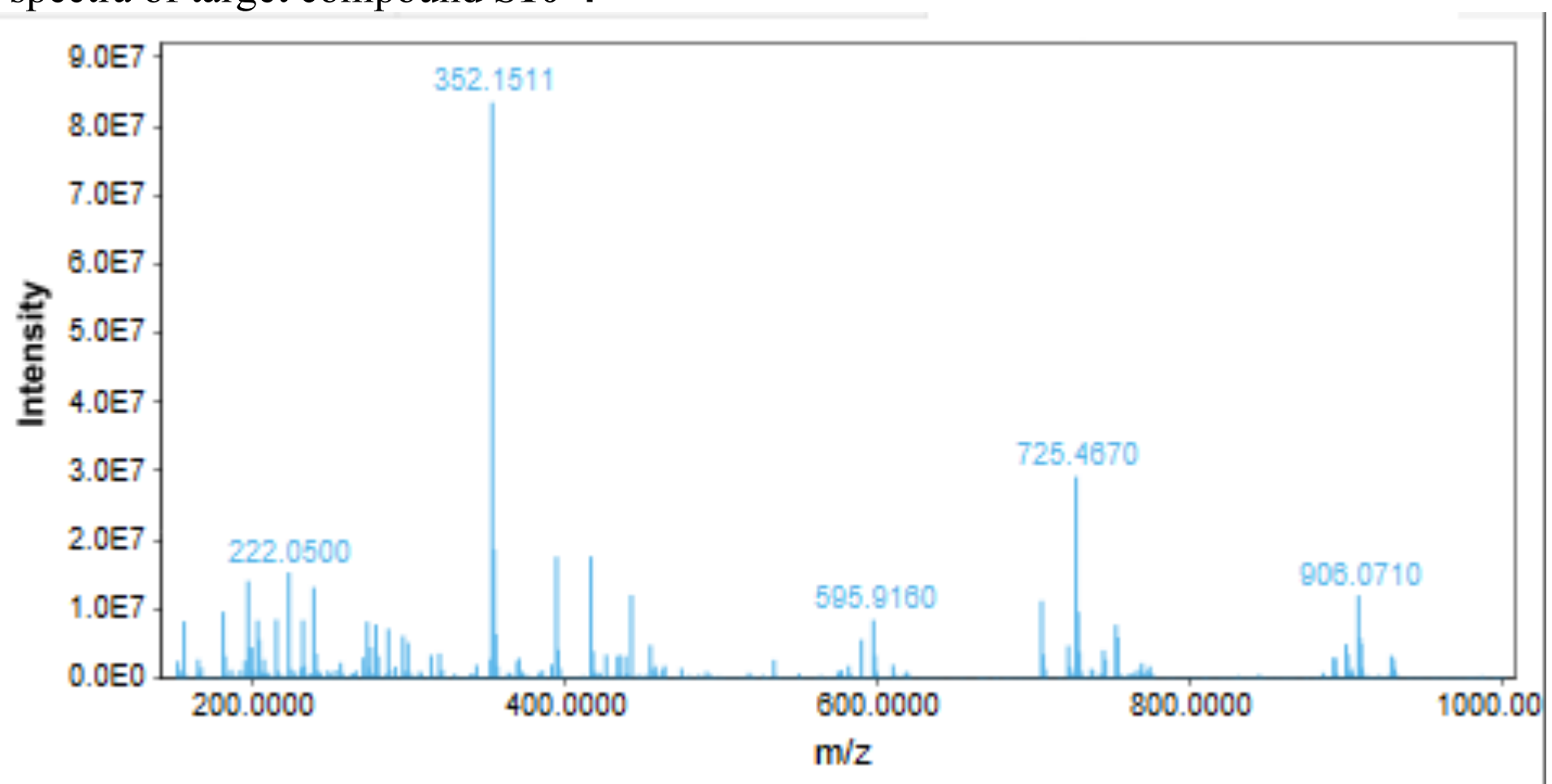

Supplement: Supplementary file 1 [file COS-23-3-193_SD1.pdf]
